# Comparative LCMS2 Analysis of Synthetic Ether- and Ester-Linked Polyacids Refines Structural Boundaries for Small Molecules in Dissolved Organic Matter

**DOI:** 10.1021/acs.est.6c01032

**Published:** 2026-04-28

**Authors:** Agnes D. Flygare, Lindon W. K. Moodie, Jeffrey A. Hawkes, Alexander J. Craig

**Affiliations:** † Department of Chemistry for Life Sciences, 8097 Uppsala University, Uppsala 752 37, Sweden; ‡ Department of Medicinal Chemistry, Uppsala University, Uppsala 752 37, Sweden

**Keywords:** dissolved organic matter, mass spectrometry, liquid chromatography, tandem mass spectrometry, chemical synthesis

## Abstract

Dissolved organic matter (DOM) is a ubiquitous component of aqueous systems that contributes to both nutrient transport and carbon sequestration. However, its complexity limits accurate analysis, restricting insight into how it affects and is affected by environmental processes. The bulk of DOM contains molecular signatures indicative of chemical structures extensively functionalized with carboxyl groups, yet few similar molecules have been isolated from either natural product or synthetic chemistry. Recently, we showed that carbocyclic carboxylate-rich alicyclic molecules (CRAM) were inconsistent with the tandem mass spectrometry (MS2) data of DOM, proposing instead that ethers and esters are likely to link subunits of common scaffolds in natural samples. Here, we have synthesized 38 ether- or ester-linked polycarboxylic acids with systematically varied functional group compositions and orientations to test whether their liquid chromatography (LC) and MS2 properties better match those of DOM than carbocyclic CRAM analogues. LC data showed that the relative placement of chemical functionality around central cores is important in determining retention times of natural isomers but is still secondary to functional group composition. MS2 experiments highlight that aliphatic polyacids linked by ethers and esters show features consistent with DOM. Conversely, aromatic carboxylic acids were found to be poor matches to DOM’s MS2 data, contradicting common paradigms in the field that ascribe elemental ratios to lignin or tannin derivatives. This work reiterates the need for compounds that accurately represent the functional group composition and carbon skeletons of not only DOM, but also theoretical proxies such as CRAM.

## Graphical abstract

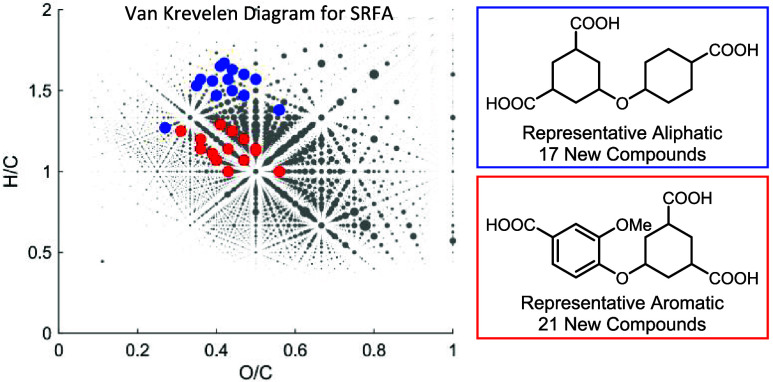

## Introduction

Dissolved organic matter (DOM) is an abundant and complex mixture of myriad organic molecules that exists in all of Earth’s waters. It plays key roles in nutrient transport and carbon sequestration but also acts as an active mediator of chemical processes within the environment. The current gold standards for DOM analysis include nuclear magnetic resonance spectroscopy (NMR),^1,2^ high-resolution mass spectrometry (HRMS),^3,4^ and tandem mass spectrometry (MS2),^5–8^ all of which can be coupled online or offline to liquid chromatography (LC). Ultimately, the current resolution of these (combined) techniques is still insufficient to truly deconvolute DOM, and typical conclusions attempt to cluster data and attribute them to speculative biomolecular classes (HRMS) or poorly defined functional group regions (NMR). As a result, understanding remains limited for how the chemistry of DOM relates to the environmental processes it governs, and much of the work in the field relies on correlations between average data metrics rather than strict chemical evidence.

DOM is often conceptually split into two major pools: labile DOM (LDOM), which constitutes around 5% of the static DOM pool, and recalcitrant DOM (RDOM), which comprises the other 95%.^9^ These groups are ambiguously defined, but generally, LDOM is considered to consist of known biometabolites and natural products, which have a high environmental turnover, while RDOM consists of a massively diverse mixture of long-term stable carboxyl-rich molecules derived from biogeochemical processing.^10–13^ These carboxyl-rich molecules have very few known parallels within biology and are poorly represented in synthetic and isolative (natural product) chemistry.^14^ As a result, the chemistry of carboxyl-rich molecules is only known through their bulk behavior in different types of natural organic matter. However, we have no ways to accurately track individual compounds in these mixtures, and few appropriate molecular comparisons exist that can be used to assist in the deconvolution of DOM data.^15^ This disconnect is problematic in understanding DOM fluxes, as chemical structure both indicates molecular origin, and determines the removal of DOM through biological transformation, sorption, and photooxidation.^16^

Many models have attempted to explain this carboxyl-rich fraction of DOM,^10–13,17–19^ but all suffer from biases based on instrumentation, fractionation, or chemical derivatization. Furthermore, analytical limitations mean that they cannot identify molecular connectivity within individual isomers, and as a result, these models hypothesize molecules based only on the averages of DOM’s prominent analytical features. In conjunction with a lack of isolated natural molecules (see above), all of these models remain unverified, and as a result, implications about biogeochemical origin or environmental relevance are speculative and should be treated with caution. Recently, our group has focused on the synthetic preparation of one proposed class of carboxyl-rich DOM, carboxyl-rich alicyclic molecules (CRAM) to investigate their chemical properties^7,14^ and tracked their behavior in analytical^20^ and applied environmental tests.^16^ Notably, these structure-based investigations showed that these fused all-carbon polycarboxylic acids are poor fits for the negative-mode LC-MS2 data of DOM, casting doubt on the validity of the carbocylic-only model of CRAM.^10^ In response to this, we proposed that the more frequent incorporation of esters and ethers between carbon subunits and within the rings of molecules is necessary to explain the MS fragmentation data of DOM in multiple environments, in line with other recent proposals in the field.^11^

To investigate this hypothesis, as well as further opportunities for characterization and reactivity studies, we have developed a straightforward synthetic approach to prepare 38 previously unreported carboxyl-rich molecule analogues. These compounds all contain a cyclic subunit linked through an ether or carboxylic ester to another carbon subunit. Both aromatic and aliphatic molecules were synthesized to probe how MS fragmentation differs between these functionalities, and to investigate whether aromatic functionalities can be detected in riverine samples, which have been generally shown to be higher in aromatic content than marine DOM by NMR.^1,10,11,21,22^ Generally, we found that aromatic carboxylic acids linked through ethers and esters are poor fits for the LCMS2 data of DOM. Conversely, certain structural features of the aliphatic polyacids prepared here are consistent with DOM’s analytical data, providing useful refinement of the likely chemical space and structure occupied by the small molecules in DOM. Notably, these results indicate that aromatic carboxylic acids, which are often proposed as tannin or lignin derivatives, are unlikely to be significant contributors to the negative-mode ionizable pool of molecules within freshwater DOM.

## Methods

Full synthetic procedures for final compounds **1–38**, and all intermediates **39–77** are reported in the Supporting Information (SI, page S4–S42), as well as NMR spectra for **1–77** (page S144–S219), and LCMS and LCMS2 data for **1–38** (page S70–S129).

### Reference Materials

The reference standards Suwannee River Fulvic Acid (SRFA) and Nordic reservoir natural organic matter (NRNOM) were used as received and dissolved in 5% acetonitrile in water to concentrations of 10 and 1 mg mL^–1^, respectively, for injection by LCMS. LC and MS2 data for SRFA are reported at *m*/*z* values corresponding to those of synthetic compounds **1–38** within the SI (Page S43–S61, S131–S143).

### DOM Sample Sites

The lake sample (Plåten) was taken by hand from a brown water lake, Plåten, Sweden (59.8627°N, 18.5426°E; ∼20 ppm DOC). The sample was concentrated by a factor of about 11 to a final concentration of 225 ppm DOC by reverse osmosis, and then further concentrated on PPL sorbent after acidification, to a final measured concentration in methanol of 3990 ppm DOC.^23^ A river sample (CJ11) was taken by hand from 10 cm depth from Munkedal, West Sweden (58.4615 N, 11.6857 E) on the 27th April, 2017.^24^ Site D1 was taken by hand at the end of the Fyrisån (59.4714°N, 17. 3944°E; ∼18 ppm DOC), a river in Uppsala just before the mouth to a large lake (Ekoln), and site D11 was taken by hand from a long water residence time lake, Långsjö (59.4656°N, 17.3733°E; ∼11 ppm DOC).^25^

### Liquid-Chromatography Mass-Spectrometry

Liquid chromatography was conducted with a Thermo Vanquish UPLC using a C18 column at a flow rate of 0.5 mL min^–1^ from 5 to 100% acetonitrile in water with 0.1% formic acid as an additive (pH ∼ 2.7). Mass spectrometry was performed using an Orbitrap Q Exactive (Thermo Fisher), with electrospray ionization conducted in negative mode at −3.0 kV, and masses detected over 150–1000 Da. MS2 experiments were conducted at normalized collision energies of 35 and 75 V by higher energy collision dissociation (HCD) using the PRM method. Synthetic compounds and DOM mixtures were analyzed individually. Code used to process MS data is available at doi: 10.5281/zenodo.19823396, raw data is available on request.

### Synthetic Scope

Synthesis focused on a simplified strategy in comparison with our previous approaches,^7,14^ in which we used the Mitsunobu reaction to prepare intermediate ethers or esters that could be hydrolyzed to form aromatic ether-linked carboxylic acid targets (Figure 1, **1–20**), hydrogenated to form ester-linked carboxylic acid targets (Figure 1, **21**, **36–38**), or hydrogenated and hydrolyzed to form aliphatic ether-linked targets (Figure 1, **22–28, 30–34**). Additionally, **29** was prepared by alkylation and hydrolysis, while **35** was prepared via the in situ acetylation of alcohol **34**. Substrates focused on varying the number of carboxylic acid groups (i.e., diacid **1**, triacid **3**, tetraacid **10**), varying the relative positions of the carboxylic acids (i.e., **1** vs **2**, comparisons between **3–7**), modifying cyclic substituents (i.e., cyclopentane **8**, perillic acid-derived ether **9**), adjusting lengths of linear alkyl chains (i.e., **11–14**), and incorporation of other functionalities (i.e., ketone **15**, alcohol **16**, diol **17**, dimethylphenylcarboxylic acid **18**, methoxy ethers **19** and **20**). Not all aromatics readily underwent hydrogenation and typically required two aromatic carboxylic ester functionalities to proceed. Additionally, triacid **6**, ketone **15**, and diol **17** underwent side reactions during hydrogenation and did not afford the desired aliphatic equivalents, but appropriate protecting groups are being investigated.

**Figure 1:**
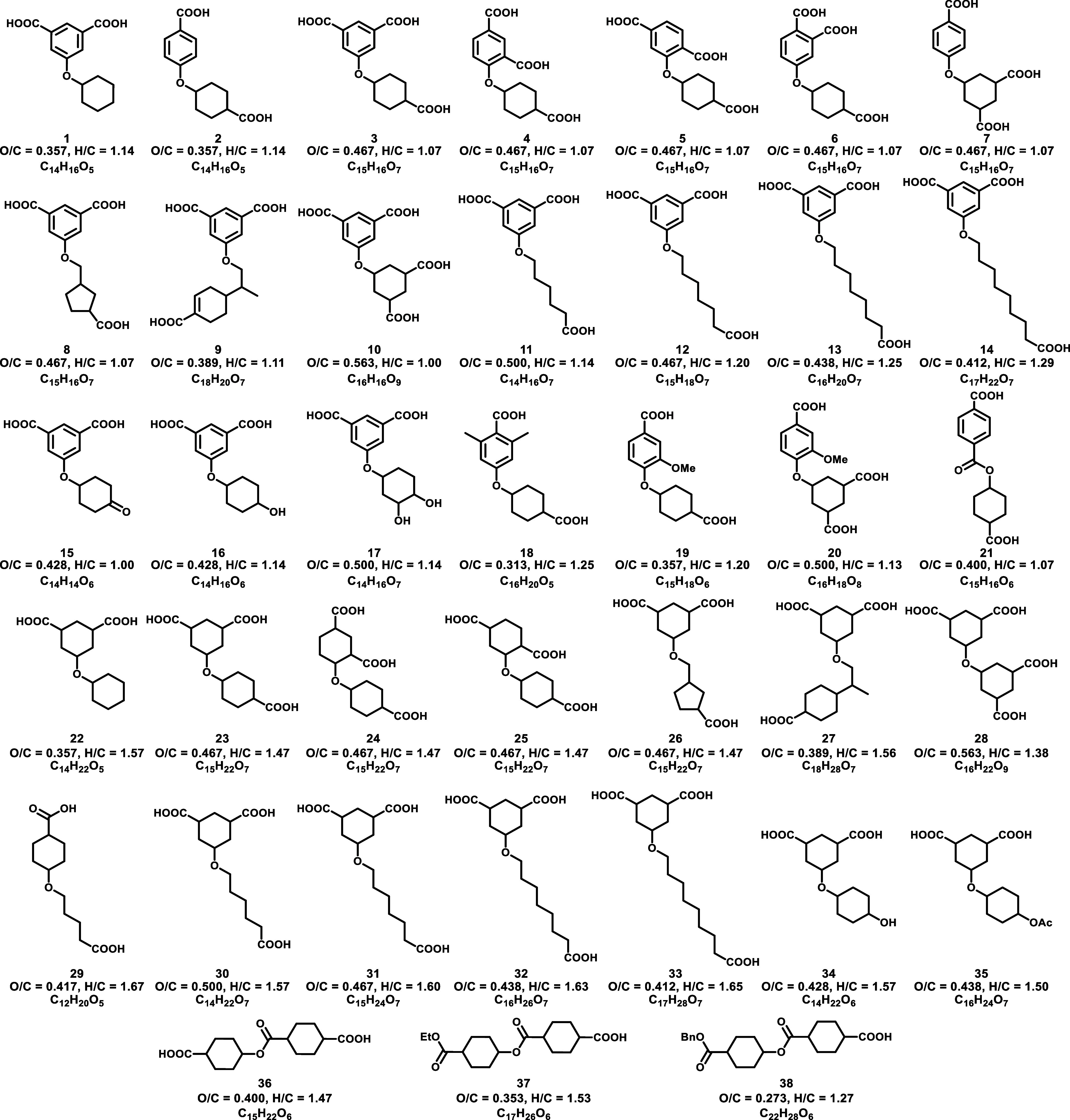
Compounds prepared in this work, including aromatic carboxylic acids **1–21**, and aliphatic carboxylic acids **22–38**.

### Retention Metrics

To analyze the retention behavior of molecules **1–38**, we employed the % cumulative intensity (%CI) metric we recently proposed (Figure 2),^7^ where %CI is determined as the percentage of summed intensity that has eluted for the equivalent molecular formula in DOM (in this case, SRFA) at the retention time of the compound (See Figures S2–S38 for examples). Samples were run using a 5–100% acetonitrile in water gradient with 0.1% formic acid as an additive on a C18 column. Comparisons to DOM were initially made against SRFA, as the use of marine standards such as Tjärnö Reference Material (TRM-0522),^26^ or North Equatorial Pacific Intermediate Water (NEqPIW)^27^ would be unlikely to provide useful comparisons for aromatic molecules. For the average retention times of aromatic compounds **1–21**, increasing the O/C ratio typically led to earlier elution, and we found that the %CI of these molecules fell between 63 and 96%. Notably, in our previous work,^7^ we found that alcohols and diols consistently eluted early (i.e., sub 40% CI), but here, aromatic alcohol **16** and diol **17** eluted at 64 and 78% CI, respectively. For aliphatic compounds **22–38**, no clear trend was observed between differences in functional groups and %CI, in contrast to our previously disclosed bicyclic compounds.^7^ The reliably high %CI values for all prepared aromatic compounds strongly indicate that aromatic polyacids within DOM are restricted to late-eluting isomers within reverse-phase LC methods, and that the hydrophobicity of aromatic functionalities outweighs the hydrophilicity of functionalities such as alcohols and diols in determining the C18 chromatography retention behavior of polycarboxylic acid molecules.

**Figure 2:**
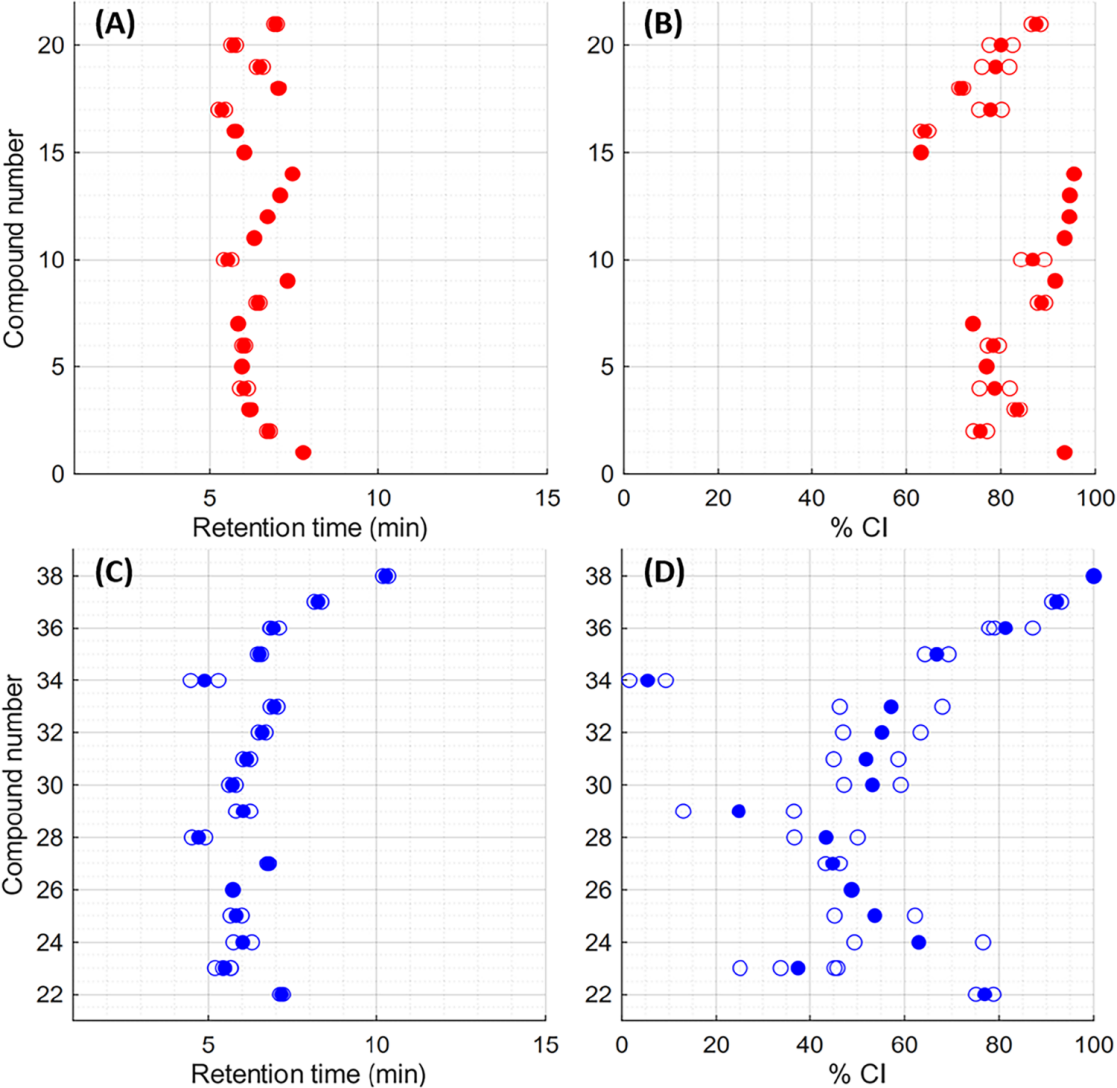
Retention times (A) and %CI values (B) for aromatic compounds **1–21** in red, and retention times (C) and %CI values (D) for aliphatic compounds **22–38** in blue. Solid circles represent average values, while hollow circles represent individual diastereomers. Compounds with %CI values of 50% elute at exactly half the intensity of the SRFA peak for the same molecular formula, while those with %CI values of 100% elute either at the very end or entirely after the elution of the same formula.

Aliphatic molecules **22–38** also typically displayed decreasing retention times as the O/C ratio increased. In %CI comparisons, a clearer trend was observed compared to the aromatics, with earlier-eluting aliphatic carboxylic acids showing a more even distribution of functional groups at the edges of their structures. Molecules of this type with rigid alkyl functionalities (two-ring containing compounds **23**, **28**, **34**, 6–43% CI) had lower %CIs than those with some flexibility (**26** and **27**, 45 and 49% CI), while compounds with fully linear alkyl functionalities had slightly higher %CIs again (**30–33**, 52–57% CI). At high %CI values, compounds tended to have larger hydrophobic domains, with triacids **25** (54% CI) and **24** (63% CI) having their oxygen containing functionalities closer together than triacid **23**, while acetyl ester **35** (67% CI), cyclohexyl ether **22** (76% CI), cyclohexyl ester **36** (81% CI), ethyl ester **37** (92% CI) and benzyl ester **38** (100% CI) all contain extensive aliphatic regions with no H-bond donors at the pH of the chromatography method (pH 2.7). This hydrophobic domain explanation may provide additional context for the %CI profiles observed in bicyclic polycarboxylic acids, where we previously reported^7^ that increasing carboxylic acid functionality led to higher %CI metrics for this type of scaffold (discussion provided below, with structural comparisons shown in Figure 8).

### Fragmentation Data

Fragmentation studies used higher energy collisional dissociation (HCD) at 35 (low energy) and 75 V (high energy). To parametrize compounds for comparison with DOM, we investigated several metrics based on ion intensity within MS2 spectra at low and high energy for both individual compounds and nominal masses in SRFA. Of course, SRFA (or indeed any DOM sample) consists of an undefined but large number of isomers for any molecular formula at any retention time. As a result, the comparisons to single synthetic isomers are done to test for common features within the many isomers in DOM. Tables of these fragmentation metrics are provided in the SI:

(1) **%Parent**—The % of intensity remaining as the parent ion, for the exact mass of a given synthetic compound, or the sum intensity of all parent masses at a given nominal mass in SRFA.
(2) **%FG**—The % intensity of peaks that correspond to CO_2_, H_2_O, CH_4_O, or sequential CO_2_, H_2_O, and CH_4_O losses from parent ions, related to carboxylic acid, alcohol, and aliphatic methyl ether^7^ fragmentations. This was calculated from the known molecular formulas of individual synthetic standards, while for SRFA, it was derived from all precursors in the MS1 spectrum to ensure that parent ions completely labile to fragmentation were still accounted for.
(3) **Avg Frag**—The intensity weighted average fragment mass.
(4) **%Radical**—The % intensity of peaks that could be assigned to a radical ion.

For the %Parent data at low fragmentation energy, aromatic acids **1–21** all had between 0 and 10% intensity remaining for their corresponding parent ions (Figure 3A). Qualitative analysis of the fragmentation spectra of these compounds at 35 V showed that this is primarily due to the high fragmentation-lability of the aromatic ether bonds in these molecules. Exceptions were cyclohexane functionalized diacid **1** (Figure S39), ketone **15** (Figure S53), and alcohol **16** (Figure S54), which showed intense single CO_2_ losses and little ether fragmentation at low energy. This is almost certainly due to the aliphatic portions of these molecules having poor capacity to stabilize negative charge (Figure 3, **i**), and as a result being unable to undergo charge migration or charge retention fragmentations.^28^ In contrast, all other aromatic molecules had an ionizable carboxylic acid on their aliphatic subunits (Figure 3, **ii**), with the one exception being diol **17** (Figure 3, **iii**), where a cyclic species would still provide charge stabilization.

**Figure 3:**
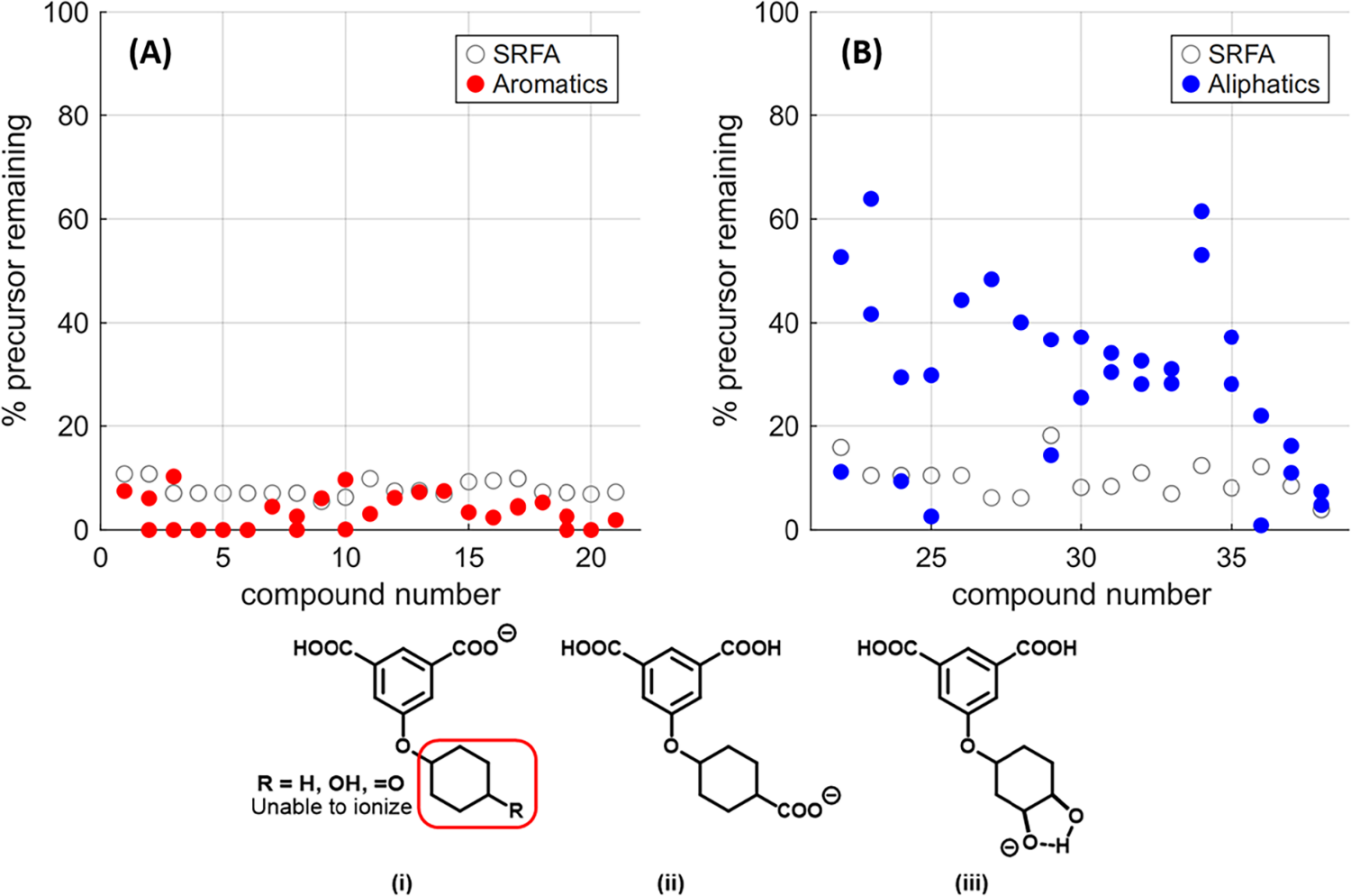
Top %Parent values at 35 V HCD Fragmentation for aromatic compounds 1–21 in red (A), and for aliphatic compounds 22–38 in blue (B). Hollow circles represent %Parent values for equivalent nominal masses in SRFA. Bottom: Subunits of cyclohexane 1, ketone 15, and alcohol 16 that are unable to stabilize a negative charge (i), and subunits that can stabilize a charge for carboxylic acids (ii) and diol (iii).

For aliphatic molecules **22–38** (Figure 3B), parent ion intensities were far more diverse, ranging from 1 to 64%. Notably, diastereomers showed remarkably different parent ion stability, with, for example, cyclohexane ether **22** having isomers retaining 11 and 53% parent intensity, and dicarboxylic acid **29** having isomers with 14 and 37% remaining intensity. In comparison, SRFA %Parent intensities at low energy were relatively consistent across all tested nominal masses and occurred between 4 and 18% from nominal masses between *m*/*z* 243 and 387. On average, these %Parent intensities were slightly higher in SRFA than for the aromatic compounds, and much lower in SRFA than for the aliphatic compounds. High-energy comparison provided no additional insight, as all single compounds and all tested SRFA masses showed no remaining parent ion intensity.

To further investigate the differences between this set of synthetic compounds and DOM, %FG and Avg Frag metrics were examined (Figure 4). Focusing first on low-energy fragmentation, SRFA again delivered relatively consistent values, with all %FG intensities ranging from 38 to 69%, and all Avg Frag values occurring between *m*/*z* 161 and 219. Aromatic compounds typically delivered far lower %FG intensity compared to SRFA, with 19 of 21 compounds occurring below 30%, and the majority occurring below 10% (Figure 4A). The exceptions were again cyclohexane functionalized diacid **1** and ketone **15**, which exhibited greater ether MS fragmentation stability than the other model compounds (see above). Avg Frag values were also lower than the average for SRFA, with only seven isomers falling between *m*/*z* 160 and 200, while twenty-two isomers fell below *m*/*z* 160, which was again attributed to the relative lability of the aromatic ether functionalities (Figure 4C). Aliphatic compounds showed a more varied %FG intensity distribution, with five compounds with isomers between 40 and 60%, five compounds with isomers between 20 and 40%, and 11 compounds with isomers between 0 and 20% intensity (Figure 4B). Aliphatic Avg Frag masses occurred over a wider range, with the lowest compound showing an avg frag of *m*/*z* 140, and the highest showing an Avg Frag of *m*/*z* 307 (Figure 4D).

**Figure 4:**
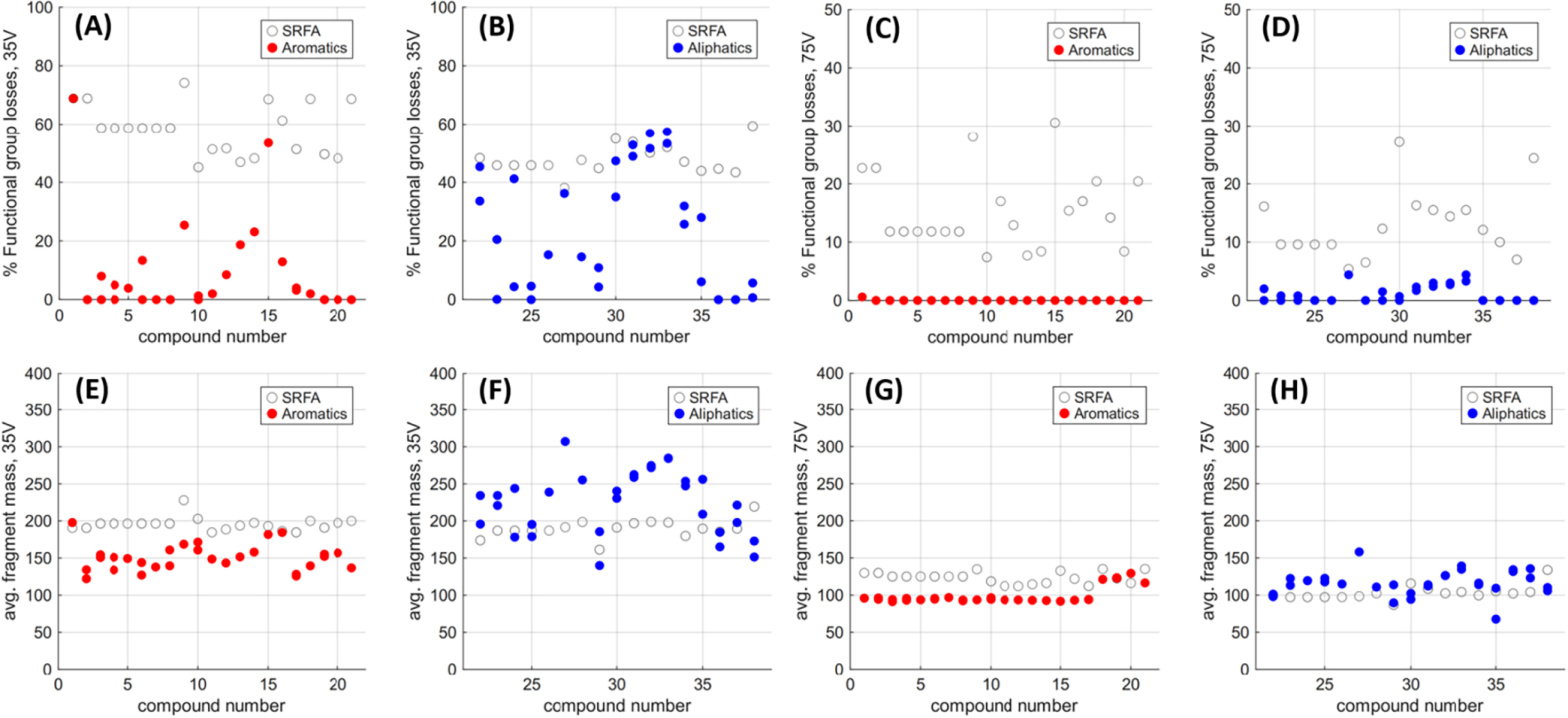
%FG at 35 V HCD Fragmentation for aromatic compounds (A) 1–21 in red, and for aliphatic compounds (B) 22–38 in blue, %FG at 75 V HCD Fragmentation for aromatic compounds (C) 1–21 in red, and for aliphatic compounds (D) 22–38 in blue, Avg Frag values at 35 V for aromatic compounds (E) 1–21 in red, and for aliphatic compounds (F) 22–38 in blue, and Avg Frag values at 75 V for aromatic compounds (G) 1–21 in red, and for aliphatic compounds (H) 22–38 in blue. Hollow circles represent %FG and Avg Frag values for equivalent nominal masses in SRFA.

For %FG analysis in higher energy fragmentation experiments (see SI), all but one aromatic compound had 0% remaining intensity, with the exception being cyclohexane derivative **1** at 1%. Aliphatic compounds also had few functional group fragments at high energy, with all compounds falling between 0 and 10%FG intensity. Here, SRFA had slightly higher %FG intensities than those for all single aromatic compounds, with values between 5 and 31%. Avg Frag masses for aromatic compounds at high energy were remarkably consistent, as compounds **1–17** converged on a single dominant fragment, based on ether cleavage and one or two decarboxylations (Figure 5, **iv**). Aliphatic avg frag metrics at higher energy were again more varied, falling between *m*/*z* 67 and 158. Low mass Avg Frag outlier acetyl ester **35** (Figure S73) had an isomer dominated by the presence of acetate anion fragments (Figure 5, **v**), while high avg mass outlier perillic acid-derived ether **27** (Figure S65) had an intense peak corresponding to a fragment where the remaining oxygenated functionality would be conformationally restricted from being able to interact and fragment further (Figure 5, **vi**). SRFA high energy Avg Frag masses all occurred between *m*/*z* 87 and 134, but had a far wider variation between lowest and highest fragment *m*/*z* than for any of the prepared synthetic analogues.

**Figure 5:**
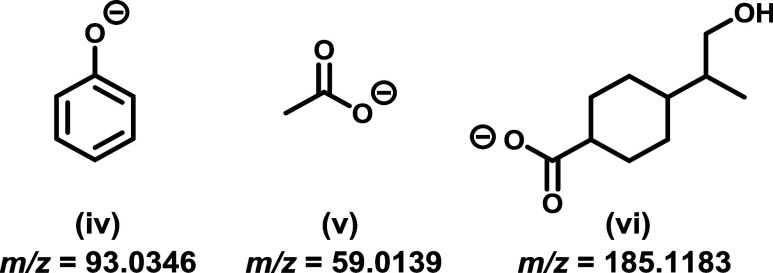
Common fragment (iv) found for aromatic carboxylic acid ethers **1–17**, acetyl anion fragment (v) derived from acetyl ester **35**, and fragment (vi) derived from perillic acid ether **27**.

During qualitative analysis of the MS2 spectra of these new synthetic compounds, it became obvious that radical anion fragments were being formed for aromatic compounds containing an aliphatic subunit that could not strongly stabilize a negative charge (Figure 6A). The relative proportion of these radical fragments strongly increased at higher energy fragmentation, had higher intensity as the acidity of these functional groups decreased, and occurred even if a different subunit had a carboxylic acid (i.e., methoxy ethers **19** and **20**). Methoxy ether **19** showed the highest %Radical, with one isomer at 36% at low energy and 98% at high energy. Conversely, across all fragmented nominal masses, SRFA showed the highest %Radical intensity of 7% at low energy, and 13% at high energy. No synthetic aliphatic compounds showed any %Radical intensity at low or high energy, with the exception of benzyl-protected ester **38** (Figure 6B).

**Figure 6:**
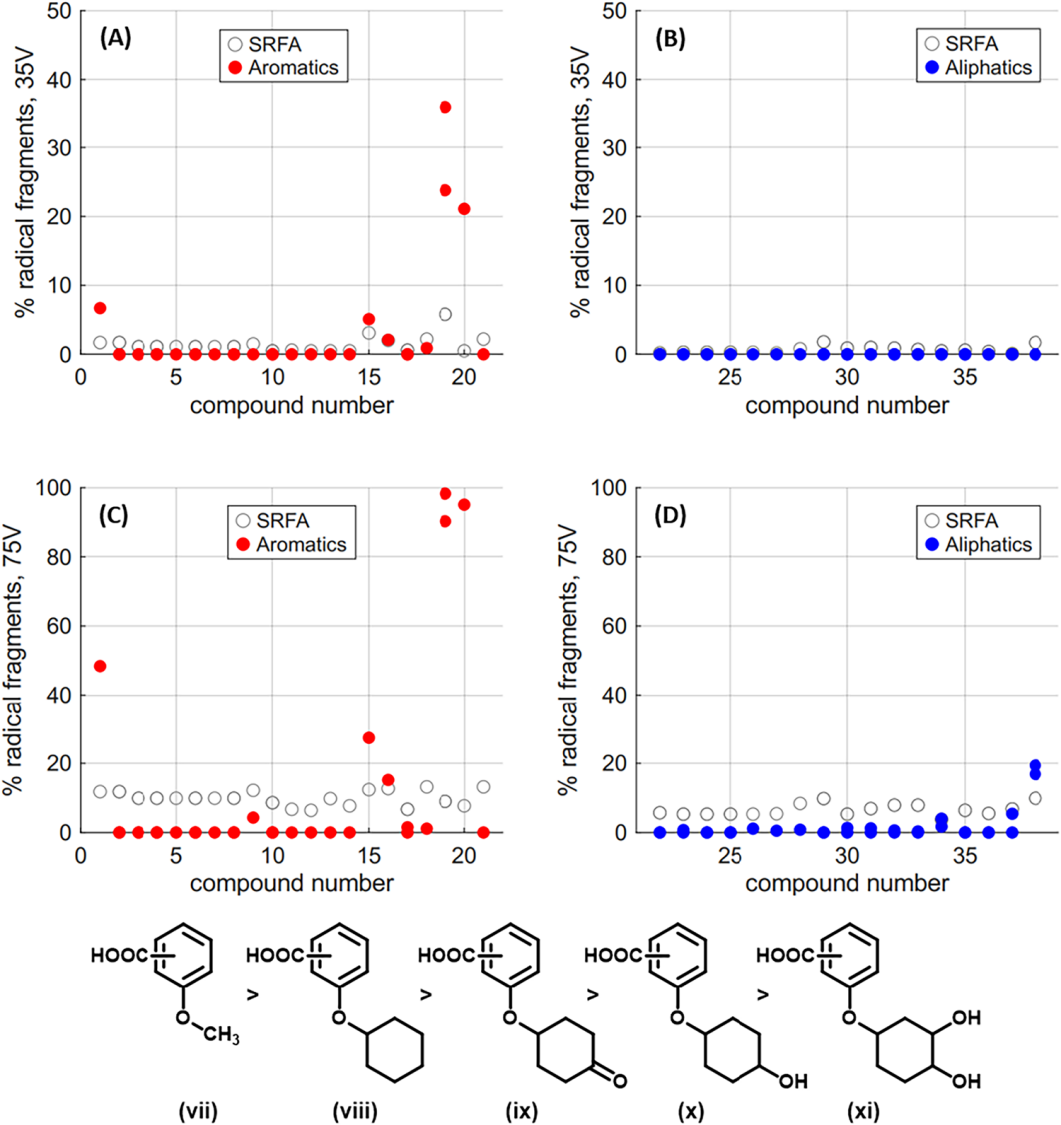
Top: %Radical at 35 V HCD Fragmentation for aromatic compounds (A) **1–21** in red, and for aliphatic compounds (B) **22–38** in blue, and %Radical at 75 V HCD Fragmentation for aromatic compounds (C) **1–21** in red, and for aliphatic compounds (D) **22–38** in blue. Hollow circles represent %FG and Avg Frag values for equivalent nominal masses in SRFA. **Bottom**: Relative intensities of radical anion fragmentation based on functional group differences (**vii-xi**).

### Comparison to Other Freshwater DOM Samples

To check how broadly our conclusions apply to DOM across a range of freshwater environments, we investigated both %CI and fragmentation metrics for five other DOM samples. These included four in-house archived terrestrial DOM samples, along with NRNOM from the IHSS. In increasing order of degradation from the original source, the extra four samples were from a humic lake (Plåten, SRFA similar degradation),^23^ site “D1” from the end of Fyrisån river in Uppsala,^25^ site “CJ11” from the end of the Munkedal river in west Sweden,^24^ and site “D11” from a long water residence time lake (Långsjö, NRNOM similar degradation) near Uppsala, Sweden.^25,29^

Unfortunately, the column we used to calculate %CI values for compounds **1–38** and SRFA initially was degraded and needed to be replaced, and the replacement column (with the same specifications) required a slightly lower flow rate (0.4 compared with 0.5 mL min^–1^) to operate at appropriate pressure. To compare %CI values, we ran all six DOM samples on this replacement column with the new flow rate and calculated the 5, 10, 25, 50, 75, 90, and 95%CI values for each DOM sample at all *m*/*z* values correlating to synthetic compounds **1–38** (shown in Figure S248, with XICs for all masses and samples shown in Figures S249–S276). Comparing %CI values for each mass across the six samples, the 50% CI value for 26 of the 28 masses varied by less than 1 min in elution time. Furthermore, for the 10, 25, 50, 75, and 90% CI intervals of all 28 masses, 98 of 140 values occurred within 1 min of each other, 33 of 140 values occurred between 1 and 2 min of each other, and 9 values fell above 2 min. Significant variability between samples almost exclusively occurred for *m*/*z* values where significant XIC intensity was present for both very hydrophilic material (i.e., elution times between 0.5 and 3 min) and hydrophobic material (i.e., elution times between 4 and 10 min), for example, at *m*/*z* 277.0717 (Figure S252) or 295.0827 (Figure S258). Overall, elution profiles across all *m*/*z* values remained similar, and on average, SRFA represented slightly more hydrophobic isomers than the other five samples.

Due to limited sample quantity, we chose 10 nominal masses to fragment at low and high energy for fragmentation metric comparison between the six freshwater DOM samples (Figure 7). These focused on masses corresponding to synthetic compounds that produced high %Radical metrics (e.g., methoxy ethers **19** and **20**), or that we had several regioisomers of (e.g., triacids **22–26**), with the remainder chosen to evenly cover our investigated mass range (i.e., between *m*/*z* 263 and 357). Notably, some of these DOM samples displayed high-intensity MS2 peaks correlating to halogenated compounds (either as parent or fragment ions), which we presumed to be anthropogenic pollutants. As a result, Figure 7 shows %Parent, %FG, Avg Frag, and %Radical data for the six freshwater DOM samples at low and high energy, for MS2 peaks with mass defects between x.0 and x.5, while excluding those that are highly likely to be halogenated (i.e., between x.5 and x.0). Corresponding metrics for all mass defects are shown in Figure S277, and tables for both are available in Tables S5–S8, with the main differences corresponding to outliers (e.g., %Radical for NRNOM at *m*/*z* 343).

**Figure 7:**
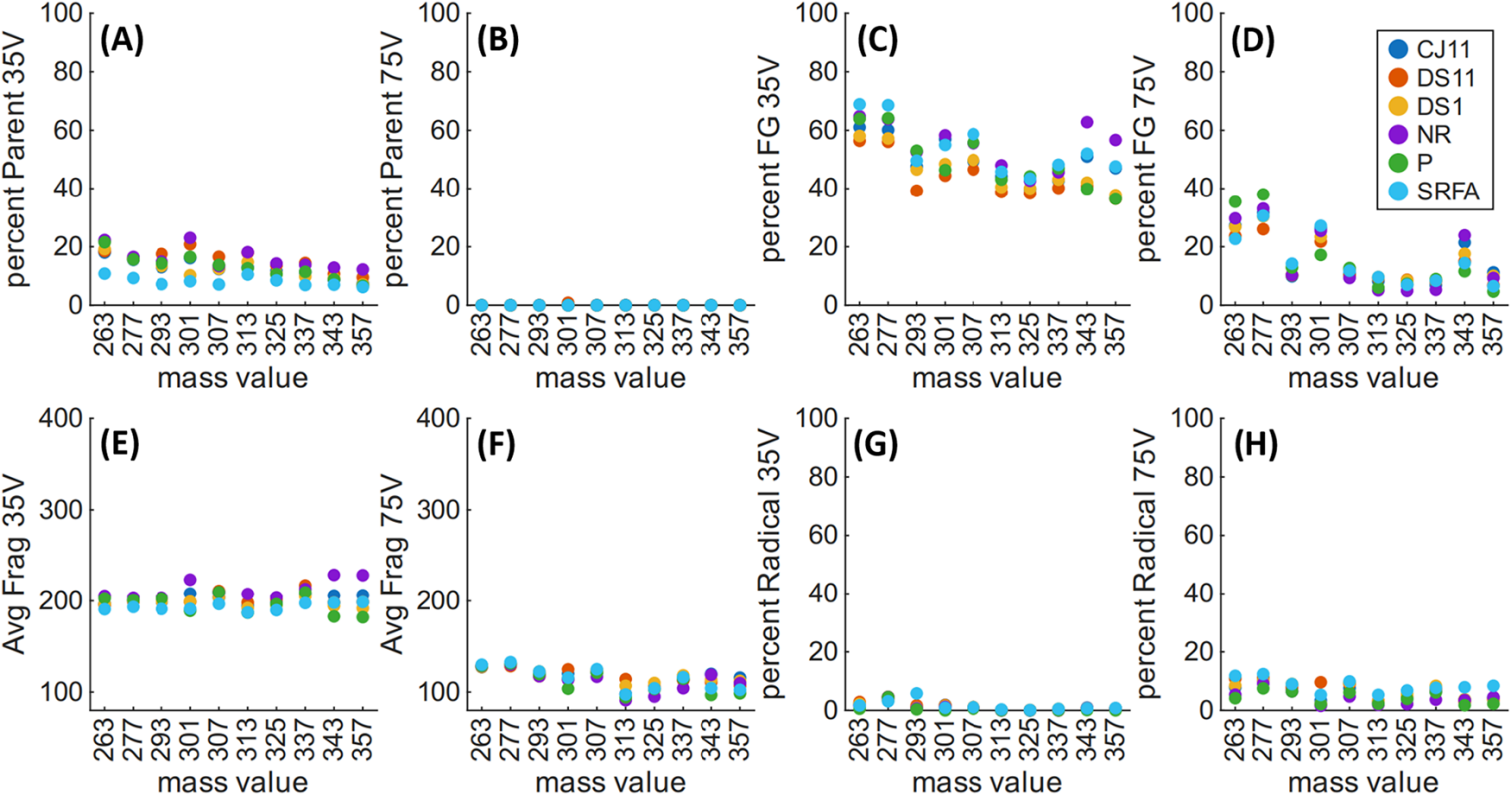
35 V %Parent, (A), 75 V %Parent (B), 35 V %FG (C), 75 V %FG (D), 35 V Avg Frag (E), 75 V Avg Frag (**F**), 35 V %Radical (G), and 75 V %Radical (H) for DOM samples CJ11 (dark blue), DS11 (orange), DS1 (yellow), NRNOM (purple), Plåten (green), and SRFA (light blue).

Analysis of these metrics across the six samples highlighted that trends in fragmentation data show little variability among freshwater environments. Under low-energy fragmentation, the largest difference for these ten nominal masses between SRFA and the five other DOM samples was observed in %Parent (SRFA = 6–11%, other samples = 7–21%), while %FG (SRFA = 43–69%, other samples = 36–64%), Avg Frag (SRFA = 187–198, other samples = 182–217), and %Radical (SRFA = 0–6%, other samples = 0–5%) showed relatively minor differences. At high energy, little variation was seen between all six examined samples; %Parent (SRFA = 0–0%, other samples = 0–0%), %FG (SRFA = 7–31%, other samples = 5–38%), Avg Frag (SRFA = 97–133, other samples = 91–131), and %Radical (SRFA = 5–12%, other samples = 2–11%).

### Discussion and Environmental Implications

LC data indicated that for polycarboxylic acids, aromatic functionalities dominated the C18 retention characteristics of other functional groups, with all prepared aromatic molecules eluting late in comparison to their equivalent molecular formulas in freshwater DOM, based on our %CI metric. Conversely, aliphatic ethers and esters showed a larger range of %CI values, where the presence of hydrophobic regions (specifically those lacking H-bond donors at the pH, which chromatography was performed at) tended to increase the %CI of individual molecules in comparison with freshwater DOM (Figure 8). This offers an additional nuance to the variation in %CI between our previously published bicyclic compounds,^7^ where diacid **78** had a %CI of 45%, while triacids **79** had %CI values of 63–87%, and tetraacids **80** had %CI values between 76% and 91%. These tri- and tetra carboxylic acids have a similar clustering of their functionality that can participate in H-bonding, and it is easy to envision how regions of these molecules would associate with nonpolar C18 resins, while their hydrophilic portions are simultaneously exposed to aqueous mobile phases. It still holds true that for molecules with similar backbones and functional group distributions, increasing the number of carboxylic acids decreases %CI, with triacid **23** having a %CI of 37% and tetraacid **28** having a %CI of 43%. It is difficult to envision a way to “cover” the hydrophobic portions of **28** with carboxylic acids in a more evenly distributed way, and as a result, it is highly likely that early eluting DOM isomers (i.e., sub 50% CI) contain other H-bond donors such as alcohols, reinforcing the conclusions in our previous work.^7^

**Figure 8:**
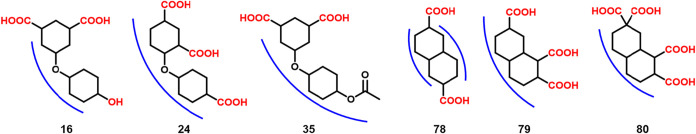
Retention comparisons for compound **16**, **24**, **35**, and diacid **78**, triacids **79**, and tetraacids **80**, based on H-bonding donation capacity (red) and hydrophobic regions (blue).

Fragmentation experiments at low energy highlighted the lability of the ether and ester functionalities within the synthesized aromatic carboxylic acids. This led to universally decreased parent ion intensities (**1–21**, %Parent = 0–10%), and provided low intensity of ions corresponding to sequential CO_2_ and H_2_O losses from parent ions (**2–14**, **16–21** %FG = 0–25%), with exceptions for compounds **1** and **15**, which contain subunits unable to stabilize a negative charge. In contrast, while freshwater DOM had very depleted parent ion intensities at a low fragmentation energy (6–23%), it showed universally higher %FG intensities (36–69%). At high energy, only one synthetic aromatic ether (cyclohexane **1**) retained any functional group fragments, while all examined masses in freshwater DOM had %FG intensities between 5 and 38%. Furthermore, freshwater DOM %Radical intensities did not exceed 6% at low-energy fragmentation and did not exceed 12% at high energy, while for aromatic methoxy ethers **19** and **20**, low-energy experiments showed significant production of radical anions (**19** = 24 and 36%, **20** = 21%), and radical anions were the dominant feature at high energy (**19** = 90 and 98%, **20** = 95%).

The combination of these fragmentation metrics with the reliably late %CI values of the aromatic compounds prepared here means that a significant contribution of small-molecule ether- and ester-containing aromatic carboxylic acids to freshwater DOM is unlikely. Furthermore, the high %Radical values of methoxy ethers **19** and **20** are vital when considering the near-ubiquitous discussion of the lignin and tannin compound classes in the HRMS analysis of freshwater DOM. Hydrolyzable tannins contain frequent carboxylic acids and aromatic ester linkages, and lignin is defined by the presence of aromatic subunits with ether bonds to aliphatic subunits, alongside extensive incorporation of aromatic methoxy-ether functionality. This data ultimately casts doubt on the definitions of ‘lignin-like’ or ‘tannin-like’ components based on the MS analysis of freshwater DOM, and limits the potential for these molecular formulas to be structurally related to the speculative parent biomolecules. While compounds derived from the extensive reworking of lignin and tannin essentially must contribute to freshwater DOM, the types of structures that relate to this reworking must be reconsidered moving forward. This is especially true in the context of HRMS analysis, where parent biochemical molecular formulas must be decoupled from quantitative analysis.^30,31^

MS2 metrics for the aliphatic polyacids in this work were generally more similar to those for DOM than those for the aromatic polyacids. Notably, semilinear compounds such as perillic acid-derived ether **27** (Figure S65) and aliphatic ethers **30–33** (Figures S68–S71) showed similarities to DOM, with sequential CO_2_ and H_2_O losses from their parent ion, as well as low intensity ether fragmentations. Conversely, dicyclohexyl compounds (e.g., **23–25**) tended to show increased backbone fragmentation relative to functional group fragments in comparison with DOM, and predominantly led to single CO_2_ losses and few H_2_O losses from their parent ions. Proximity of functional groups promoted fragmentation, observable with the significantly depleted %Parent values for 1-carboxylic acid-2-ethers **24** and **25** when compared to 1-carboxylic acid-3-ethers **23** and **34**. Furthermore, both isomers of acetyl ester **35** produced acetate fragments even at low energy, which were widely observable across all fragmented nominal masses within freshwater DOM.

Taken together, the fragmentation data for the aliphatic polyacids prepared here and for freshwater DOM suggest several likely features for the common small molecules in freshwater DOM. First, functionalities capable of promoting H_2_O and CO_2_ losses must be clustered to promote their ubiquitous sequential losses observed in the low-energy HCD fragmentation spectra of natural samples. This is reinforced by the relatively high intensity of H_2_O, CO_2_, and H_2_O+CO_2_ losses for the 1,2-diacids prepared in our previous work.^7,14^ Notably, fragmentation spectra of DOM show significantly increased intensity of fragments related to carboxylic acid and alcohol fragmentations (e.g., 3× CO_2_ at *m*/*z* 269, 4× CO_2_ at *m*/*z* 307, 5× CO_2_ at *m*/*z* 357, with all corresponding *x*_*n*–1_CO_2_ + H_2_O losses observed) in comparison to any of the synthetic polyacids from our studies, either here or previously.^7,14^ These high numbers of CO_2_ and H_2_O may relate to some portion of isomers in DOM with many closely clustered carboxylic acids and alcohols, but further investigation is required.

Additionally, small ether and ester functionalities (i.e., sub 100 Da) almost certainly contribute to substructures within the ionizable small molecule pool in freshwater DOM. Plausible functional group precursors for these peaks were inferred from the presence of a high-intensity acetate ion in fragmentation experiments on acetyl ester **35**, together with our previous work demonstrating the high lability of aliphatic methoxy ethers on polyacids.^7^ Notably, for **35**, neutral loss of this fragment was directly observed from the parent ion *(m*/*z* 327 to 267), whereas in freshwater DOM, this loss was only observed following sequential losses of CO_2_ and H_2_O. This is possibly explained by the apparent clustering of functionality that leads to sequential CO_2_ and H_2_O losses in DOM, but verification is required through appropriate analogue synthesis. Finally, larger rigid aliphatic units with distant oxygen-based functionality such as those derived from perillic acid-derived ether **27** remain stable even under high-energy fragmentation conditions. This suggests that the larger *m*/*z* ions remaining in high energy fragmentation experiments on DOM are also likely to be those where remaining functionalities are distant from one another on large and rigid aliphatic subunits.

While it cannot be ignored that oxygen-functionalized aromatics are significant contributors to the aggregate NMR data of freshwater DOM samples, the distribution of these functionalities between ionizable and nonionizable molecules (e.g., larger polymers) must be further investigated if molecular origin and environmental fluxes are to be quantified using NMR and HRMS. Phenols remain as potential contributors to this small molecule pool, although lignin-like phenols would almost certainly lead to the extensive generation of radical fragments within MS2 experiments.^32^ Future work is underway to prepare a more extensive set of lignin and tannin derivatives to more accurately define their likely contributions to environmental samples. Conversely, aliphatic ester and ether-linked polycarboxylic acids show several structural consistencies with the MS2 data of DOM. While further investigation is required to refine the exact subunits that lead to DOM’s common smaller *m*/*z* fragments, this work has shown that the fragmentation of larger subunits into smaller fragments based on internal oxygen functionality is generally consistent with DOM’s MS2 data. As a result, subsequent work will aim to prepare a wider range of carbon-based subunits within these systems in order to refine potential biosynthetic inputs to these structures in the environment. Furthermore, the preparation of polyacid cyclic ethers (e.g., tetrahydrofurans)^33,34^ and cyclic esters (i.e., lactones) is required to fully explore our hypotheses around the distribution of ethers and esters in DOM’s small organic molecules, and spirocyclic equivalents of these compounds must be investigated to interrogate other speculative structures proposed within the field.^11^

## Supplementary Material



## Notes

Synthetic procedures; plots for cumulative intensity data; tabulated fragmentation metrics; liquid-chromatography tandem mass spectrometry data for final compounds and corresponding SRFA masses; NMR data for all synthetic intermediates and final compounds; and %CI and XIC data for the six investigated freshwater DOM samples (PDF)
