# Supplementary material for: Comparative LCMS2 Analysis of Synthetic Ether- and Ester-Linked Polyacids Refines Structural Boundaries for Small Molecules in Dissolved Organic Matter
Source: Environ Sci Technol. 2026 Apr 28;60(18):13427–36. doi: 10.1021/acs.est.6c01032 (PMC13173522; doi:10.1021/acs.est.6c01032)
Supplement: Supplementary file 1 [file es6c01032_si_001.pdf]

Supporting Information

Comparative LCMS2 Analysis of Synthetic Ether- and Ester-Linked Polyacids Refines Structural Boundaries for Small Molecules in Dissolved Organic Matter

Agnes D. Flygare,[1] Lindon W. K. Moodie,[2] Jeffrey A. Hawkes,[1] and Alexander J. Craig[1,2]*

[1.] Department of Chemistry for Life Sciences, Uppsala University, Uppsala 752 37, Sweden

[2.] Department of Pharmacy, Uppsala University, Uppsala 752 37, Sweden

*Corresponding author: alexander.craig@uu.se

Summary: 230 pages, 277 figures, 8 tables

Contents:

Abbreviations List	**S2**
General Methods	**S3**
Synthetic Procedures for the preparation of **1**-**77**	**S4-42**
% Cumulative Intensity Plots for **1**-**38**	**S43-S61**
Tabulated Fragmentation Metrics for **1**-**38**	**S62-S65**
Tabulated Fragmentation Metrics for DOM Samples	**S66-S69**
Liquid Chromatography Tandem Mass Spectrometry Data for **1**-**38**	**S70-S129**
Tandem Mass Spectrometry Data for SRFA	**S130-S143**
NMR Data for **1**-**77**	**S144-S219**
% Cumulative intensity comparisons for DOM samples	**S220**
XIC plots for DOM samples for % cumulative intensity comparisons	**S221-S230**
Unadjusted fragmentation metric plots for freshwater DOM samples	**S230**

Abbreviations List

CI	Cumulative intensity
CRAM	Carboxylate rich alicyclic molecule
d	Doublet
dd	Doublet of doublets
ddd	Doublet of doublet of doublets
dddd	Doublet of doublet of doublets of doublets
dt	Doublet of triplets
DIAD	Diisopropyl azodicarboxylate
ESI	Electrospray ionization
Et_2O	Diethyl ether
EtOAc	Ethyl acetate
FG	Functional group
HCD	Higher energy collisional dissociation
HRMS	High-resolution mass spectrometry
LCMS	Liquid chromatography mass spectrometry
m	Multiplet
MeOD	Deuterated methanol
MS2	Tandem mass spectrometry
NMR	Nuclear magnetic resonance
Pd/C	Palladium on carbon
PET	Petroleum ether
q	Quartet
Rh/C	Rhodium on carbon
s	Singlet
sept	Septet
SRFA	Suwannee River Fulvic Acid
THF	Tetrahydrofuran
TIC	Total ion chromatogram
TLC	Thin-layer chromatography
t	Triplet
UV	Ultraviolet
XIC	Extracted Ion Chromatogram

General Methods

Thin-layer chromatography (TLC) was performed on 0.2 mm aluminium plates precoated with silica gel 60 F_{254} (Merck). Compounds were visualized with an ultraviolet (UV)-light, and stained with potassium permanganate. Column chromatography was performed with silica gel (40-63 µM). High resolution mass-spectra to obtain accurate masses of intermediate and final compounds were recorded with an Orbitrap Q Exactive (Thermo Fisher) using liquid chromatography mass spectrometry (LCMS) with electrospray ionisation in positive or negative mode. Liquid chromatography was conducted at a flow rate of 500 µl/min on C18 (Phenomenex Kinetex 150x2.1 mm, 1.7 µm pore size), in a linear gradient running from 5-100% mobile phase B, where A was 0.1% formic acid in deionised water (milliQ, Millipore), and B was acetonitrile (LiChrosolv, Merck) with 0.1% formic acid. Data were collected at 35,000 resolution (at *m/z* 400). ^{1}H NMR spectra were recorded at 400 MHz on a Varian Mercury Plus spectrometer. All spectra were recorded from samples in either $CDCl_3$ or MeOD, at room temperature in 5 mm nuclear magnetic resonance (NMR) tubes. Chemical shifts are reported relative to the residual solvent peak at δ 7.26 for $CDCl_3$ or δ 3.31 for MeOD. Resonances were assigned as follows: chemical shift (multiplicity, number of protons, coupling constant(s)). Multiplicity abbreviations are reported by the conventions: s (singlet), d (doublet), dd (doublet of doublets), ddd (doublet of doublet of doublets) dt (doublet of triplets), t (triplet), tt (triplet of triplets), q (quartet), sept (septet), m (multiplet). Proton decoupled ^{13}C NMR spectra were recorded at 101 MHz on a Varian Mercury Plus spectrometer under the same conditions as for the ^{1}H NMR spectra. Chemical shifts have reported relative to the residual solvent peak at δ 77.16 for $CDCl_3$ or δ 49.90 for MeOD. All solvents and reagents were used as received.

Analytical Liquid Chromatography

Liquid chromatography was conducted with a Thermo Vanquish UPLC using a Phenomenex Kinetex C18 column (2.1x150 mm, 2.6 µm) at a flow rate of 0.5 ml/min. Mobile phase A was 0.1% formic acid (AnalaR Normapur, VWR) in deionized grade water (Millipore MilliQ), B was 0.1% formic acid in LCMS grade acetonitrile (Lichrosolv, Supelco, Merck). A linear gradient started at 5% B then increased at 1 min from 5 to 100% B at 10 min, followed by a 2.1 min washout phase at 100% B, a decrease to 5% B and a 2.7 min equilibration phase at 5% B (method length = 15 min). The column oven was set to 40 °C to decrease back pressure, and the autosampler chamber to 5 °C.

Analytical Mass Spectrometry

Mass spectrometry was conducted with an Orbitrap Q Exactive (Thermo Fisher). Electrospray ionization was used as the ionization source, using a heated unit running at 200 °C and -3.5 kV (negative mode). Sheath gas and auxiliary gas were set to 20 and 2 units, respectively, S-Lens was set to 50, and capillary temperature was set to 300 °C. For the experiments, resolution was set to 35,000, and a maximum injection time of 200 ms was chosen, aiming to trap 3×10^{6} ions in the orbitrap. MS2 experiments were conducted at normalized collision energies of 35 and 75 by higher energy collision dissociation (HCD) experiments using the PRM method, and 2×10^{5} ions were targeted using automatic gain control for these experiments, with a maximum trapping time of 100 ms. An isolation window of 1 Da was selected, centered on the deprotonated mass of the compound being investigated.

General Procedure A

An aromatic alcohol (1 equiv.), an aliphatic alcohol (1 equiv.) and PPh_3 (1.1 equiv.) were combined into a round-bottom flask and pump-purged with nitrogen twice. The mixture was then dissolved in dry THF (2.5 mL per 1.5 mmol) before DIAD was added (1.5 equiv.). The reaction was stirred at room temperature overnight. At completion as monitored by LCMS, the mixture was diluted with ethyl acetate, before being washed with distilled water (×2), 1M aqueous NaOH (×1), and brine (×1). The solution was then dried over Na_2SO_4 before the solvent was evaporated in vacuo.

Diester **39**

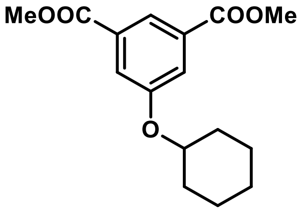

Following General Procedure A: a mixture of 3,5-dimethoxyphenol (0.251 g, 1.62 mmol), cyclohexanol (0.17 mL, 0.16 g, 1.6 mmol), PPh_3 (0.467 g, 1.78 mol) and DIAD (0.48 mL, 0.49 g, 2.4 mmol) were subjected to reaction. The crude mixture was subjected to silica gel column chromatography (1:19 to 1:4, EtOAc: PET) to afford the title compound (0.090 g, 19%) as a clear oil.

^{1}H NMR (400MHz, $CDCl_3$) δ 8.23 (t, 1H, *J* = 1.5 Hz), 7.73 (d, J = 1.5 Hz, 2H), 4.37 (tt, 1H, *J* = 8.7, 4.3 Hz), 3.93 (s, 6H), 2.06-1.91 (m, 2H), 1.89-1.72 (m, 2H), 1.66-1.49 (m, 3H), 1.47-1.28 (m, 3H). ^{13}C NMR (101 MHz, $CDCl_3$) δ 166.4, 158.1, 131.9, 122.8, 121.4, 76.0, 52.5, 31.6, 25.6, 23.6. LRMS (ESI-MS) calculated for **39** [M + H]$^+$: $C_{18}H_{24}O_5^+$: 293.1; not found.

Diester **40**

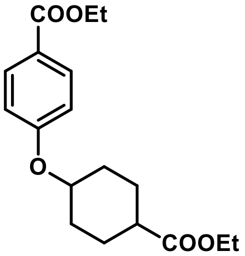

Following General Procedure A: a mixture of 4-hydroxybenzoate (0.235 g, 1.41 mmol), ethyl 4-hydroxycyclohexanecarboxylate (0.23 mL, 0.25 g, 1.4 mmol), PPh_3 (0.407 g, 1.55 mol) and DIAD (0.42 mL, 0.43 g, 2.1 mmol) were subjected to reaction. The crude mixture was subjected to silica gel column chromatography (0:1 to 2:3, EtOAc: PET) to afford the title compound as a mixture of diastereomers (0.137 g, 23%) as colourless amorphous crystals.

^{1}H NMR (400 MHz, $CDCl_3$) δ 7.98-7.93 (m, 2H), 6.91-6.85 (m, 2H), 4.56-4.24 (m, 3H), 4.16-4.09 (m, 2H), 2.43-2.28 (m, 1H), 2.21-1.89 (m, 4H), 1.77-1.40 (m, 4H), 1.40-1.33 (m, 3H), 1.28-1.22 (m, 3H). ^{13}C NMR (101 MHz, $CDCl_3$) δ 175.4, 166.5, 161.54, 161.45, 131.73, 131.70, 122.9, 115.4, 115.2, 75.1, 42.2, 41.9, 30.8, 28.9, 26.8, 23.7, 14.5, 14.4. LRMS (ESI-MS) calculated for **40** $C_{18}H_{24}O_5^+$ [M + H]$^+$: 321.2, found; 321.2.

Triester **41**

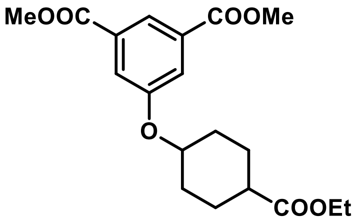

Following General Procedure A: a mixture of dimethyl 5-hydroxyisophthalate (0.200 g, 0.952 mmol), ethyl 4-hydroxycyclohexanecarboxylate (0.15 g, 1.4 mL, 0.88 mmol), PPh_3 (0.275 g, 1.05 mol) and DIAD (0.21 mL, 0.21 g, 1.1 mmol) were subjected to reaction. The crude mixture was subjected to silica gel column chromatography (3:17 to 2:3, EtOAc: PET) to afford the title compound as a 3:17 mixture of diastereomers (0.059 g, 17%) as a pale-yellow oil.

Major: ^{1}H NMR (400 MHz, $CDCl_3$) δ 8.27 (t, 1H, *J* = 1.4 Hz), 7.75 (d, 2H, *J* = 1.4 Hz), 4.35 (tt, 1H, *J* = 10.0, 3.9 Hz), 4.17 (q, 2H, *J* = 7.1 Hz), 3.96 (s, 6H), 2.47-2.34 (m, 1H), 2.25-2.17 (m, 2H), 2.16-2.09 (m, 2H), 1.75-1.60 (m, 2H), 1.59-1.47 (m, 2H), 1.29 (t, 3H, *J* = 7.2 Hz). ^{13}C NMR (101 MHz, $CDCl_3$) δ 175.0, 166.0, 157.6, 131.7, 122.9, 121.0, 75.4, 60.2, 52.2, 41.8, 30.4, 26.4, 14.1. *Minor:* ^{1}H NMR (400 MHz, $CDCl_3$) δ 8.27 (t, 1H, *J* = 1.4 Hz), 7.77 (d, 2H, *J* = 1.4 Hz), 4.65-4.60 (m, 1H), 4.18 (q, 2H, *J* = 7.1 Hz), 3.96 (s, 6H), 2.47-2.34 (m, 1H), 2.06-1.93 (m, 4H), 1.86-1.76 (m, 2H), 1.75-1.60 m, 2H), 1.29 (t, 3H, *J* = 7.2 Hz). ^{13}C NMR (101 MHz, $CDCl_3$) δ 175.0, 166.0, 157.4, 131.6, 122.7, 121.1, 72.0, 60.2, 52.4, 41.6, 28.5, 23.3, 14.1. HRMS (ESI-MS) calculated for **41** $C_{19}H_{25}O_7^+$ $[M + H]^+$: 365.1595, found; 365.1597.

Triester **42**

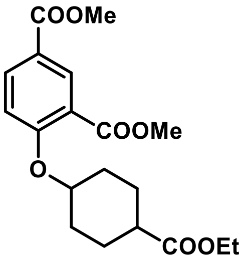

Following General Procedure A: a mixture of dimethyl 4-hydroxyisophthalate (0.276 g, 1.31 mmol), ethyl 4-hydroxycyclohexanecarboxylate (0.21 mL, 0.23 g, 1.3 mmol), PPh_3 (0.408 g, 1.44 mol) and DIAD (0.39 mL, 0.40 g, 2.0 mmol) were subjected to reaction. The crude mixture was subjected to silica gel column chromatography (1:19 to 1:4, EtOAc: PET) to afford the title compound as a 1:2 mixture of diastereomers (0.098 g, 50%) as a colourless oil.

Major ^{1}H NMR (400 MHz, $CDCl_3$) δ 8.45-8.44 (d, 1H, *J* = 2.3 Hz), 8.10-8.06 (m, 1H), 7.00-6.95 (m, 1H), 4.74-4.67 (m, 1H), 4.13 (q, 2H, *J* = 7.1 Hz), 3.92 (s, 3H), 3.88 (s, 3H), 2.43 (m, 1H), 2.22-1.91 (m, 4H), 1.82-1.56 (m, 4H), 1.25 (t, 3H, *J* = 7.1 Hz). ^{13}C NMR (101 MHz, $CDCl_3$) δ 175.4, 166.7, 166.2, 160.3, 134.7, 134.0, 120.1, 121.6, 113.7, 72.5, 60.4, 52.3, 52.2, 42.3, 28.9, 23.4. *Minor* ^{1}H NMR (400 MHz, $CDCl_3$) δ 8.43 (d, 1H, *J* = 2.3 Hz), 8.10-8.06 (m, 1H), 7.00-6.95 (m, 1H), 4.44-4.35 (m, 1H), 4.13 (q, 2H, *J* = 7.1 Hz), 3.88 (s, 3H), 3.87 (s, 3H), 2.43 (m, 1H), 2.22-1.91 (m, 4H), 1.82-1.56 (m, 4H), 1.25 (t, 3H, *J* = 7.1 Hz). ^{13}C NMR (101 MHz, $CDCl_3$) δ 175.3, 166.2, 166.0, 161.0, 134.7, 133.8, 122.1, 121.3, 114.2, 76.4, 60.5, 52.19, 52.16, 41.7, 30.2, 26.3. HRMS (ESI-MS) calculated for **42** $C_{19}H_{25}O_7^+$ $[M + H]^+$: 365.1595, found; 365.1594.

Triester **43**

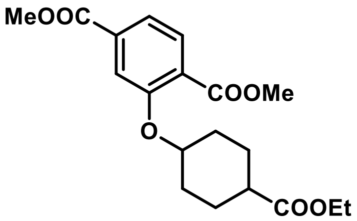

Following General Procedure A: a mixture of 1,4-dimethyl 2-hydroxy-1,4-benzenedicarboxylate (0.465 g, 2.10 mmol), ethyl 4-hydroxycyclohexanecarboxylate (0.34 mL, 0.36 g, 2.1 mmol), PPh_3 (0.606 g, 2.31 mol) and DIAD (0.62 mL, 0.64 g, 3.2 mmol) were subjected to reaction. The crude mixture was subjected to silica gel column chromatography (0:1 to 3:7, EtOAc: PET) to afford the title compound as a 1:4 mixture of diastereomers (0.383 g, 50%) as a colourless oil.

Major ^{1}H NMR (400 MHz, $CDCl_3$) δ 7.77-7.73 (m, 1H), 7.63-7.56 (m, 2H), 4.41-4.32 (m, 1H), 4.12 (q, 2H, *J* = 7.1 Hz), 3.91 (s, 3H), 3.87 (s, 3H), 2.41-2.30 (m, 1H), 2.19-2.12 (m, 2H), 2.11-2.03 (m, 2H), 1.68-1.50 (m, 4H), 1.24 (t, 3H, *J* = 7.2 Hz). ^{13}C NMR (101 MHz, $CDCl_3$) δ 175.4, 166.4, 166.3, 156.9, 134.2, 131.4, 126.1, 121.5, 116.3, 76.7, 60.4, 52.6, 52.2, 41.8, 30.4, 26.4, 14.3. *Minor* ^{1}H NMR (400 MHz, $CDCl_3$) δ 7.77-7.73 (m, 1H), 7.63-7.56 (m, 2H), 4.73-4.68 (m, 1H), 4.12 (q, 2H, *J* = 7.1 Hz), 3.91 (s, 3H), 3.90 (s, 3H), 2.41-2.30 (m, 1H), 2.11-2.03 (m, 2H), 2.00-1.89 (m, 2H), 1.78-1.69 (m, 2H), 1.68-1.50 (m, 2H), 1.23 (t, 3H, *J* = 7.1 Hz). ^{13}C NMR (101 MHz, $CDCl_3$) δ 175.4, 167.1, 166.4, 156.3, 134.1, 131.7, 126.1, 121.2, 115.4, 77.3, 60.3, 52.5, 52.4, 42.3, 28.8, 23.3, 14.3. HRMS (ESI-MS) calculated for **43** $C_{19}H_{25}O_7^+$ $[M + H]^+$: 365.1595, found; 365.1596.

Triester **44**

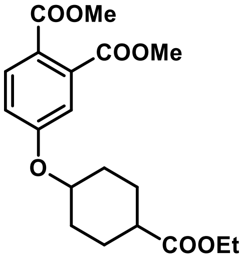

Following General Procedure A: a mixture of dimethyl 4-hydroxyphthalate (0.314 g, 1.49 mmol), ethyl 4-hydroxycyclohexanecarboxylate (0.24 mL, 0.26 g, 1.49 mmol), PPh_3 (0.440 g, 1.64 mol) and DIAD (0.44 mL, 0.45 g, 2.24 mmol) were subjected to reaction. The crude mixture was subjected to silica gel column chromatography (1:19 to 1:4, EtOAc: PET) to afford the title compound as a single diastereomer (pure: 0.032 g, 6%, contaminated with 25% reduced DIAD: 0.183 g, 25% adjusted for contamination) as a colourless oil.

^{1}H NMR (400 MHz, $CDCl_3$) δ 7.79 (d, 1H, *J* = 8.7 Hz), 7.03 (d, 1H, *J* = 2.5 Hz), 6.96 (dd, 1H, *J* = 8.6, 2.6 Hz), 4.30 (tt, 1H, *J* = 10.0, 4.0 Hz), 3.91 (s, 3H), 3.86 (s, 3H), 2.35 (tt, 1H, *J* = 11.0, 3.8 Hz), 2.21-2.13 (m, 2H), 2.12-2.04 (m, 2H), 1.67-1.56 (m, 2H), 1.56-1.42 (m, 2H), 1.26 (t, 3H, *J* = 7.1 Hz). ^{13}C NMR (101 MHz, $CDCl_3$) δ 175.3, 169.0, 166.9, 160.4, 136.0, 131.8, 122.0, 117.2, 115.1, 75.5, 60.6, 52.9, 52.5, 42.1, 30.6, 26.7, 14.4. HRMS (ESI-MS) calculated for **44** $C_{19}H_{25}O_7^+$ $[M + H]^+$: 365.1595, found; 365.1596.

Triester **45**

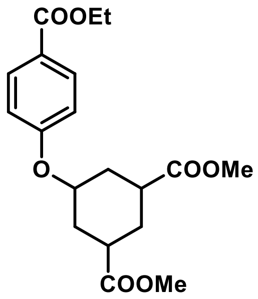

Following General Procedure A: a mixture of ethyl 4-hydroxybenzoate (0.124 g, 0.746 mmol) and dimethyl 5-hydroxycyclohexane-1,3-dicarboxylate (0.160 g, 0.739 mmol), PPh_3 (0.214 g, 0.816 mol) and DIAD (0.22, 0.22 g, 1.11 mmol) were subjected to reaction. The crude mixture was subjected to silica gel column chromatography (2:100:1 to 20:80:1, EtOAc:Toluene:Et_3N) to afford the title compound as a mixture of diastereomers (0.096 g, 35%) as a white solid.

^{1}H NMR (400 MHz, $CDCl_3$) δ 7.99-7.94 (m, 2H), 6.90-6.86 (m, 2H), 4.33 (q, 2H, *J* = 7.1 Hz), 3.68 (s, 6H), 2.56-2.41 (m, 4H), 2.36-2.20 (m, 2H), 1.63-1.50 (m, 3H), 1.36 (t, 3H, *J* = 7.1 Hz). ^{13}C NMR (101 MHz, $CDCl_3$) δ 174.0, 166.3, 161.1, 131.7, 123.4, 115.3, 74.4, 60.8, 52.1, 40.7, 33.8, 30.5, 14.5. LRMS (ESI-MS) calculated for **45** $[M + H]^+$: $C_{19}H_{25}O_7^+$: 365.2; found; 365.2.

Triester **46**

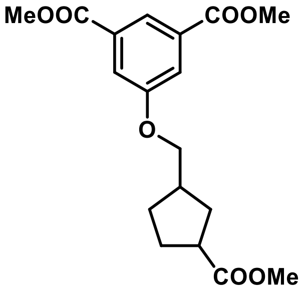

Following General Procedure A: a mixture of dimethyl 5-hydroxyisophthalate (0.181 g, 0.83 mmol), ethyl 3-(hydroxymethyl)cyclopentane-1-carboxylate (0.139 g, 0.83 mmol), PPh_3 (0.246 g, 0.91 mol) and DIAD (0.25 mL, 0.25 g, 1.25 mmol) were subjected to reaction. The crude mixture was subjected to silica gel column chromatography (1:9 to 3:1, Et_2O: PET) to afford the title compound as a 2:3 mixture of diastereomers (0.059 g, 20%) as a colourless oil.

Major ^{1}H NMR (400 MHz, $CDCl_3$) δ 8.26 (t, 1H, *J* = 1.4 Hz), 7.73 (d, 2H, *J* = 1.4 Hz), 3.99-3.90 (m, 8H), 3.68 (s, 3H), 2.93-2.81 (m, 1H), 2.63-2.41 (m, 1H), 2.25-1.73 (m, 5H), 1.70-1.42 (m, 1H). ^{13}C NMR (101 MHz, $CDCl_3$) δ 176.7, 166.3, 159.3, 131.9, 123.1, 112.0, 72.3, 52.5, 51.9, 42.9, 39.4, 33.5, 30.0, 29.3. *Minor* ^{1}H NMR (400 MHz, $CDCl_3$) δ 8.26 (t, 1H, *J* = 1.4 Hz), 7.72 (d, 2H, *J* = 1.4 Hz), 3.99-3.90 (m, 8H), 3.69 (s, 3H), 2.93-2.81 (m, 1H), 2.63-2.41 (m, 1H), 2.25-1.73 (m, 5H), 1.70-1.42 (m, 1H). ^{13}C NMR (101 MHz, $CDCl_3$) δ 176.8, 166.3, 159.3, 113.9, 123.1, 120.0, 72.1, 52.6, 51.9, 43.3, 38.7, 32.9, 29.4, 29.0. HRMS (ESI-MS) calculated for **46** $C_{18}H_{23}O_7^+$ $[M + H]^+$: 351.1438, found; 351.1439.

(-)-Perillic acid methyl ester **47**

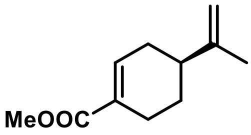

To a solution of (S)-(-)perillic acid (0.464 g, 2.79 mmol) in acetone (10 mL) was added Cs_2CO_3 (1.27 g, 3.90 mmol) followed by methyl iodide (0.70 mL, 1.6 g, 11.2 mmol). The mixture was heated to reflux and stirred for 6 hours. At completion, as tracked by TLC, the mixture was cooled to room temperature and diluted into ethyl acetate. The mixture was washed with 0.1 N sodium hydroxide (x1), distilled water (x2) and brine (x1), before being dried over Na_2SO_4, filtered, and concentrated *in vacuo* to afford the title compound (0.478 g, 95%) as a colourless oil.

^{1}H NMR (400 MHz, $CDCl_3$) δ 7.01-6.98 (m, 1H), 4.77-4.74 (m, 1H), 4.73-4.70 (m, 1H), 3.73 (s, 3H), 2.50-2.43 (m, 1H), 2.43-2.38 (m, 1H) 2.38-2.04 (m, 3H), 1.93-1.84 (m, 1H), 1.77-1.72 (m, 3H), 1.51-1.41 (m, 1H). HRMS (ESI-MS) calculated for **47** $C_{11}H_{17}O_2^+$ $[M + H]^+$: 181.1223, found; 181.1222.

Alcohol **48**

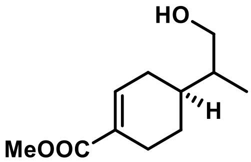

To a solution of perillic acid methyl ester **47** (0.244 g, 1.35 mmol) in dry THF (10 mL) was added 9-borabicyclo[3.3.1]nonane (5.4 mL, 0.5 M in THF, 2.7 mmol) at 0 °C under nitrogen. The mixture was warmed to room temperature and stirred overnight, before 2 M aqueous NaOH (3.7 mL) and 60% hydrogen peroxide (1.85 mL) were added, and the mixture stirred for a further 30 minutes. At completion, the mixture was extracted with EtOAc (x2), before the organic portions were combined, washed with brine, dried over Na_2SO_4, filtered, and concentrated *in vacuo*. The crude mixture was subjected to silica gel column chromatography (1:19 to 3:1 PET:EtOAc) to afford the title compound as a 2:3 mixture of diastereomers (0.138 g, 51%) as a clear oil.

Major ^{1}H NMR (400 MHz, $CDCl_3$) δ 6.99-6.94 (m, 1H), 3.72 (s, 3H), 3.67-3.62 (m, 1H), 3.56-3.51 (m, 1H), 2.47-2.42 (m, 1H), 2.31-2.10 (m, 2H), 2.09-1.91 (m, 1H), 1.88-1.77 (m, 1H), 1.69-1.54 (m, 2H), 1.37-1.16 (m, 1H), 0.95 (d, 3H, J = 6.7 Hz). ^{13}C NMR (101 MHz, $CDCl_3$) δ 168.1, 139.6, 130.3, 66.2, 51.7, 39.7, 34.6, 30.4, 26.5, 24.8, 13.6. *Minor* ^{1}H NMR (400 MHz, $CDCl_3$) δ 6.99-6.94 (m, 1H), 3.72 (s, 3H), 3.67-3.62 (m, 1H), 3.56-3.51 (m, 1H), 2.51-2.47 (m, 1H), 2.31-2.10 (m, 2H), 2.09-1.91 (m, 1H), 1.88-1.77 (m, 1H), 1.69-1.54 (m, 2H), 1.37-1.16 (m, 1H), 0.93 (d, 3H, J = 6.7 Hz). ^{13}C NMR (101 MHz, $CDCl_3$) δ 168.1, 139.5, 130.3, 66.3, 51.7, 39.9, 34.5, 28.4, 25.0, 24.7, 13.3. HRMS (ESI-MS) calculated for **48** $C_{11}H_{19}O_3^+$ $[M + H]^+$: 199.1329, not found.

Triester **49**

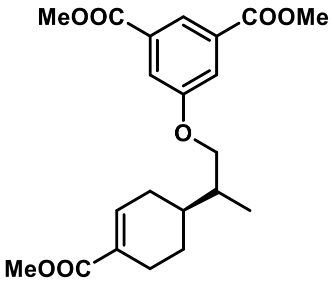

Following General Procedure A: a mixture of dimethyl 5-hydroxyisophthalate (0.112 g, 0.502 mmol), perillic acid derived alcohol **48** (0.100 g, 0.502 mmol), PPh_3 (0.174 g, 0.55 mol) and DIAD (0.15 mL, 0.15 g, 0.75 mmol) were subjected to reaction. The crude mixture was subjected to silica gel column chromatography (1:9 to 1:1, EtOAc: PET) to afford the title compound as 2:3 mixture of diastereomers (0.145 g, 74%) as a colourless oil.

Major ^{1}H NMR (400 MHz, $CDCl_3$) δ 8.26 (t, 1H, *J* = 1.4 Hz), 7.73 (d, 2H, *J* = 1.4 Hz), 7.00-6.94 (m, 1H), 4.02-3.89 (m, 8H), 3.72 (s, 3H), 2.48-2.45 (m, 1H), 2.35-2.12 (m, 2H), 2.12-2.00 (m, 1H), 2.00-1.82 (m, 2H), 1.82-1.64 (m, 1H), 1.42-1.25 (m, 3H), 1.07 (d, 3H, *J* = 6.9 Hz). ^{13}C NMR (101 MHz, $CDCl_3$) δ 168.0, 166.3, 159.3, 139.2, 131.9, 130.4, 123.0, 119.9, 71.7, 52.5, 51.6, 37.2, 35.0, 30.3, 25.0, 22.1, 14.1. *Minor* ^{1}H NMR (400 MHz, $CDCl_3$) δ 8.26 (t, 1H, *J* = 1.4 Hz), 7.73 (d, 2H, *J* = 1.4 Hz), 7.00-6.94 (m, 1H), 4.02-3.89 (m, 8H), 3.72 (s, 3H), 2.53-2.49 (m, 1H), 2.35-2.12 (m, 2H), 2.12-2.00 (m, 1H), 2.00-1.82 (m, 2H), 1.82-1.64 (m, 1H), 1.42-1.25 (m, 3H), 1.04 (d, 3H, *J* = 6.9 Hz). ^{13}C NMR (101 MHz, $CDCl_3$) δ 168.0, 166.3, 159.3, 139.2, 131.9, 130.3, 123.0, 119.9, 71.7, 52.5, 51.6, 37.2, 34.9, 28.5, 26.4, 24.8, 13.7. HRMS (ESI-MS) calculated for **49** $C_{21}H_{27}O_7^+$ [M + H]$^+$: 391.1751, found; 391.1750.

Tetraester **50**

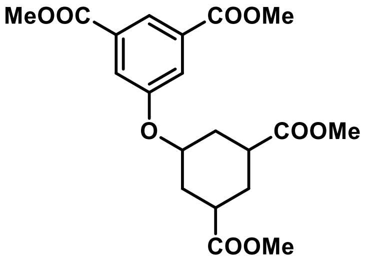

Following General Procedure A: a mixture of dimethyl 5-hydroxyisophthalate (0.133 g, 0.633 mmol), dimethyl 5-hydroxycyclohexane-1,3-dicarboxylate (0.137 g, 0.634 mmol), PPh_3 (0.183 g, 0.698 mol) and DIAD (0.14 mL, 0.14 g, 0.70 mmol) were subjected to reaction. The crude mixture was subjected to silica gel column chromatography (3:17 to 1:1, EtOAc: PET) to afford the title compound as a single diastereomer (0.144 g, 22%, yield is adjusted for contamination with reduced DIAD) as a yellow oil.

^{1}H NMR (400 MHz, $CDCl_3$) δ 8.26 (t, 1H, *J* = 1.5 Hz), 7.72 (d, 2H, *J* = 1.5 Hz), 4.35 (tt, 1H, *J* = 11.1, 4.2 Hz), 3.93 (s, 6H), 3.68 (s, 6H), 2.51 (tt, 2H, *J* = 12.7, 3.5 Hz), 2.46-2.38 (m, 2H), 2.34-2.26 (m, 1H), 1.62-1.51 (m, 3H). ^{13}C NMR (101 MHz, $CDCl_3$) δ 174.1, 166.2, 157.5, 132.1, 123.6, 121.5, 75.1, 52.6, 52.2, 40.6, 33.8, 30.6. HRMS (ESI-MS) calculated for **50** $C_{20}H_{25}O_9^+$ [M + H]$^+$: 409.1493, found; 409.1496.

Triester **51**

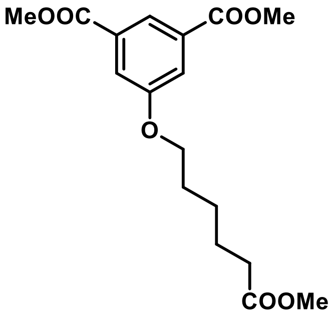

Following General Procedure A: a mixture of dimethyl 5-hydroxyisophthalate (0.200 g, 0.952 mmol), 6-hydroxy hexanoic acid ethyl ester (0.139 g, 0.952 mmol), PPh_3 (0.275 g, 1.05 mol) and DIAD (0.21 mL, 0.21 g, 1.1 mmol) were subjected to reaction. The crude mixture was subjected to silica gel column chromatography (3:17 to 3:7, EtOAc: PET) to afford the title compound (0.136 g, 42%) as a colourless oil.

^{1}H NMR (400 MHz, $CDCl_3$) δ 8.24 (t, 1H, *J* = 1.4 Hz), 7.71 (d, 2H, *J* = 1.5 Hz), 4.02 (t, 2H, *J* = 6.4 Hz), 3.92 (s, 6H), 3.66 (s, 3H), 2.34 (t, 2H, *J* = 7.6 Hz), 1.85-1.78 (m, 2H), 1.74-1.66 (m, 2H), 1.54-1.47 (m, 2H). ^{13}C NMR (101 MHz, $CDCl_3$) δ 174.1, 166.3, 159.2, 131.8, 122.9, 119.9, 68.4, 52.5, 51.6, 34.0, 28.9, 25.7, 24.7. HRMS (ESI-MS) calculated for **51** $C_{17}H_{23}O_7^+$ $[M + H]^+$: 339.1438, found; 339.1436.

Triester **52**

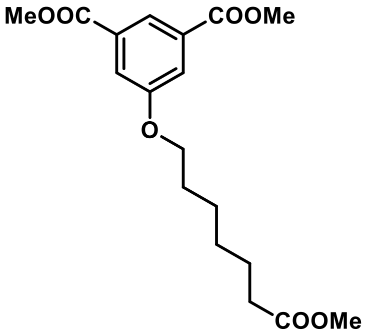

Following General Procedure A: a mixture of dimethyl 5-hydroxyisophthalate (0.200 g, 0.952 mmol), 7-hydroxy heptanoic acid ethyl ester (0.152 g, 0.952 mmol), PPh_3 (0.275 g, 1.05 mol) and DIAD (0.21 mL, 0.21 g, 1.1 mmol) were subjected to reaction. The crude mixture was subjected to silica gel column chromatography (3:17 to 1:3, EtOAc: PET) to afford the title compound (0.136 g, 40%) as a colourless oil.

^{1}H NMR (400 MHz, $CDCl_3$) δ 8.24 (t, 1H, *J* = 1.4 Hz), 7.72 (d, 2H, *J* = 1.4 Hz), 4.02 (t, 2H, *J* = 6.4 Hz), 3.92 (s, 6H), 3.66 (s, 3H), 2.32 (t, 2H, *J* = 7.4 Hz), 1.83-1.76 (m, 2H), 1.69-1.62 (m, 2H), 1.52-1.45 (m, 2H), 1.43-1.35 (m, 2H). ^{13}C NMR (101 MHz, $CDCl_3$) δ 174.3, 166.3, 159.3, 131.8, 122.9, 119.9, 68.5, 52.5, 51.6, 34.1, 29.0, 28.9, 25.8, 24.9. HRMS (ESI-MS) calculated for **52** $C_{18}H_{25}O_7^+$ $[M + H]^+$: 353.1595, found; 353.1598.

Triester **53**

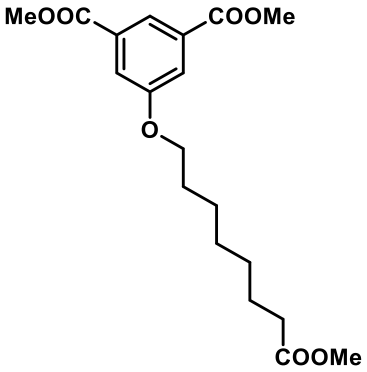

Following General Procedure A: a mixture of dimethyl 5-hydroxyisophthalate (0.200 g, 0.952 mmol), 8-hydroxy octanoic acid ethyl ester (0.166 g, 0.952 mmol), PPh_3 (0.275 g, 1.05 mol) and DIAD (0.21 mL, 0.21 g, 1.1 mmol) were subjected to reaction. The crude mixture was subjected to silica gel column chromatography (3:17 to 1:3, EtOAc: PET) to afford the title compound (0.178 g, 51%) as a pale-yellow oil.

^{1}H NMR (400 MHz, $CDCl_3$) δ 8.26 (t, 1H, *J* = 1.4 Hz), 7.73 (d, 2H, *J* = 1.4 Hz), 4.03 (t, 2H, *J* = 6.4 Hz), 3.93 (s, 6H), 3.67 (s, 3H), 2.32 (t, 2H, *J* = 7.5 Hz), 1.83-1.77 (m, 2H), 1.68-1.61 (m, 2H), 1.53-1.44 (m, 2H), 1.43-1.29 (m, 4H). ^{13}C NMR (101 MHz, $CDCl_3$) δ 174.4, 166.4, 159.3, 131.9, 122.9, 120.0, 68.7, 52.5, 51.6, 34.2, 29.2, 29.18, 29.17, 26.0, 25.0. HRMS (ESI-MS) calculated for **53** $C_{19}H_{27}O_7^+$ [M + H]$^+$: 367.1751, found; 367.1750.

Triester **54**

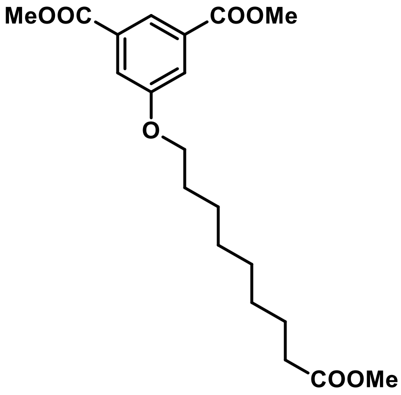

Following General Procedure A: a mixture of dimethyl 5-hydroxyisophthalate (0.200 g, 0.952 mmol), 9-hydroxy nonanoic acid ethyl ester (0.179 g, 0.952 mmol), PPh_3 (0.275 g, 1.05 mol) and DIAD (0.21 mL, 0.21 g, 1.1 mmol) were subjected to reaction. The crude mixture was subjected to silica gel column chromatography (3:17 to 1:3, EtOAc: PET) to afford the title compound (0.097 g, 27%) as a colourless oil.

^{1}H NMR (400 MHz, $CDCl_3$) δ 8.24 (t, 1H, *J* = 1.4 Hz), 7.72 (d, 2H, *J* = 1.4 Hz), 4.02 (t, 2H, *J* = 6.4 Hz), 3.92 (s, 6H), 3.65 (s, 3H), 2.30 (t, 2H, *J* = 7.6 Hz), 1.82-1.75 (m, 2H), 1.65-1.58 (m, 2H), 1.49-1.41 (m, 2H), 1.40-1.28 (m, 6H). ^{13}C NMR (101 MHz, $CDCl_3$) δ 174.4, 166.3, 159.3, 131.8, 122.9, 119.9, 68.7, 52.5, 51.6, 34.2, 29.3, 29.24, 29.17, 29.16, 26.0, 25.0. HRMS (ESI-MS) calculated for **54** $C_{20}H_{29}O_7^+$ [M + H]$^+$: 381.1908, found; 381.1904.

Ketone **55**

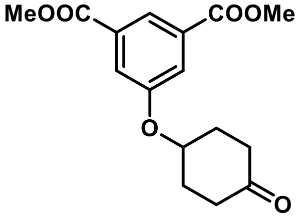

Following General Procedure A: a mixture of dimethyl 5-hydroxyisophthalate (3.28 g, 15.2 mmol), 4-hydroxycyclohexanone (1.74 g, 15.2 mmol), PPh_3 (4.36 g, 16.7 mol) and DIAD (4.5 mL, 4.6 g, 22.8 mmol) were subjected to reaction. The crude mixture was subjected to silica gel column chromatography (1:9 to 1:0, Et_2O: PET) to afford the title compound (1.10 g, 24%) as a yellow oil.

^{1}H NMR (400 MHz, $CDCl_3$) δ 8.30 (t, 1H, *J* = 1.4 Hz), 7.81 (d, 2H, *J* = 1.4 Hz), 4.87-4.79 (m, 1H), 3.94 (s, 6H), 2.68 (ddd, 2H, *J* = 15.8, 11.4, 5.9 Hz), 2.39-2.26 (m, 4H), 2.17-2.06 (m, 2H). ^{13}C NMR (101 MHz, $CDCl_3$) δ 210.2, 166.1, 157.4, 132.2, 123.6, 121.3, 71.6, 52.6, 36.8, 20.2. HRMS (ESI-MS) calculated for **55** $C_{16}H_{19}O_6^+$ $[M + H]^+$: 307.1176, found; 307.1178.

Alcohol **56**

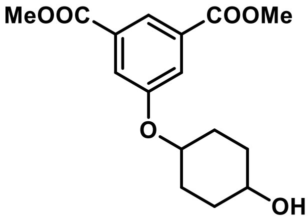

Following General Procedure A: a mixture of dimethyl 5-hydroxyisophthalate (0.912 g, 4.18 mmol), 1,4-cyclohexanediol (0.500 g, 4.18 mmol), PPh_3 (1.208 g, 4.60 mmol) and DIAD (1.23 mL, 1.27 g, 6.27 mmol) were subjected to reaction. The crude mixture was subjected to silica gel column chromatography (1:3 to 3:1, EtOAc: PET) to afford the title compound as a 1:2 mixture of diastereomers (0.200 g, 15%) as a colourless oil.

Major ^{1}H NMR (400 MHz, $CDCl_3$) δ 8.25-8.22 (m, 1H), 7.76-7.71 (m, 2H), 4.43-4.35 (m, 1H), 3.93 (s, 6H), 3.87-3.76 (m, 1H), 2.19-1.95 (m, 4H), 1.80-1.40 (m, 4H). ^{13}C NMR (101 MHz, $CDCl_3$) δ 166.3, 157.9, 131.9, 123.0, 121.3, 75.1, 68.8, 52.5, 31.7, 28.2. *Minor* ^{1}H NMR (400 MHz, $CDCl_3$) δ 8.25-8.22 (m, 1H), 7.76-7.71 (m, 2H), 4.53-4.47 (m, 1H), 4.14-4.08 (m, 1H), 3.92 (s, 6H), 2.19-1.95 (m, 4H), 1.80-1.40 (m, 4H). ^{13}C NMR (101 MHz, $CDCl_3$) δ 166.3, 157.7, 131.9, 123.0, 121.4, 72.7, 68.3, 52.5, 30.3, 27.4. HRMS (ESI-MS) calculated for **56** $C_{16}H_{21}O_6^+$ $[M + H]^+$: 309.1333, found; 309.1335.

Alkene **57**

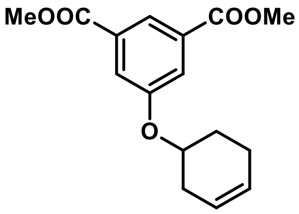

Following General Procedure A: a mixture of dimethyl 5-hydroxyisophthalate (1.05 g, 4.80 mmol), cyclohex-3-en-1-ol (0.496 g, 4.80 mmol), PPh_3 (1.38 g, 5.28 mol) and DIAD (1.42 mL, 1.46 g, 7.20 mmol) were subjected to reaction. The crude mixture was subjected to silica gel column chromatography (1:19 to 1:9, EtOAc: PET) to afford the title compound (0.105 g, 4%, contaminated with 50% DIAD) as a colourless oil.

^{1}H NMR (400 MHz, $CDCl_3$) δ 8.25 (t, 1H, *J* = 1.4 Hz), 7.76 (d, 2H, *J* = 1.4 Hz), 5.77-5.70 (m, 1H), 5.65-5.58 (m, 1H), 4.68-4.62 (m, 1H), 3.93 (s, 6H), 2.56-2.45 (m, 1H), 2.31-2.12 (m, 3H), 2.05-1.99 (m, 1H), 1.89-1.80 (m, 1H). ^{13}C NMR (101 MHz, $CDCl_3$) δ 166.4 158.0, 131.9, 127.1, 123.7, 123.0, 121.3, 73.2, 52.5, 31.0, 27.3, 23.4. HRMS (ESI-MS) calculated for **57** $C_{16}H_{19}O_5^+$ $[M + H]^+$: 291.1227, found; 291.1230.

Diol **58**

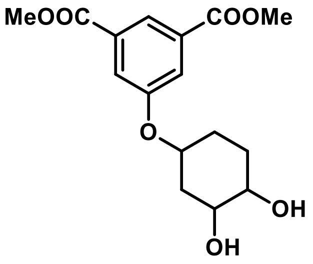

To a solution of alkene **57** (0.055 g, 0.19 mmol), in ^{t}BuOH (5 mL) and H_2O (5 mL) was added $K_2Os(OH)_4$ (0.008 g, 0.02 mmol), $K_3[Fe(CN)_6]$ (0.150 g, 0.38 mmol), and K_2CO_3 (0.046 g, 0.38 mmol) at room temperature. The mixture was vigorously stirred overnight, before being extracted with EtOAc (x3). The organic portions were combined, washed with distilled water (x2) followed by brine (x1), dried over Na_2SO_4, filtered, and concentrated *in vacuo*. The crude mixture was subjected to silica gel column chromatography (1:1 to 1:0, EtOAc:PET) to afford the title compound as a 7:3 mixture of diastereomers (0.014 g, 22%) as a clear oil contaminated with triphenylphosphine.

Major ^{1}H NMR (400 MHz, $CDCl_3$) δ 8.24 (t, *J* = 1.5 Hz, 1H), 7.73 (d, *J* = 1.5 Hz, 2H), 4.75 (tt, *J* = 6.7, 3.4 Hz, 1H), 4.14-4.05 (m, 1H), 3.92 (s, 6H), 3.87-3.79 (m, 1H), 2.49 (s, 2H), 2.19-2.10 (m, 1H), 2.06-1.71 (m, 4H), 1.65-1.56 (m, 1H). *Minor* ^{1}H NMR (400 MHz, $CDCl_3$) δ 8.28 (t, *J* = 1.5 Hz, 1H), 7.74 (d, *J* = 1.4 Hz, 2H), 4.52-4.47 (m, 1H), 4.14-4.05 (m, 1H), 3.93 (s, 6H), 3.87-3.79 (m, 1H), 2.49 (s, 2H), 2.19-2.10 (m, 1H), 2.06-1.71 (m, 4H), 1.65-1.56 (m, 1H). HRMS (ESI-MS) calculated for **58** $C_{16}H_{21}O_7^+$ $[M + H]^+$: 325.1282, found; 325.1289.

Diester **59**

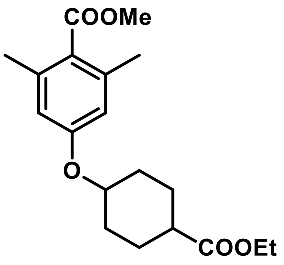

Following General Procedure A: a mixture of methyl 4-hydroxy-2,6-dimethylbenzoate (0.178 g, 0.99 mmol), ethyl 4-hydroxycyclohexanecarboxylate (0.15 mL, 0.17 g, 0.99 mmol), PPh_3 (0.296 g, 1.49 mmol) and DIAD (0.29 mL, 0.30 g, 1.49 mmol) were subjected to reaction. The crude mixture was subjected to silica gel column chromatography (1:19 to 1:4, EtOAc: PET) to afford the title compound as a 2:3 mixture of diastereomers (0.086 g, 26%) as a colourless oil.

Major ^{1}H NMR (400 MHz, $CDCl_3$) δ 6.55 (s, 2H), 4.21 (tt, 1H, *J* = 10.0, 4.3 Hz), 4.14 (q, 2H, *J* = 7.2 Hz), 3.88 (s, 3H), 2.46-2.26 (m, 7H), 2.20-1.88 (m, 4H), 1.79-1.40 (m, 5H), 1.26 (t, 3H, *J* = 7.1 Hz). ^{13}C NMR (101 MHz, $CDCl_3$) δ 175.5, 170.4,158.2, 137.9, 126.3, 115.1, 74.9, 60.5, 51.8, 42.3, 30.9, 26.9, 20.5, 14.4. *Minor* ^{1}H NMR (400 MHz, $CDCl_3$) δ 6.57 (s, 2H), 4.51-4.45 (m, 1H), 4.14 (q, 2H, *J* = 7.2 Hz), 3.88 (s, 3H), 2.46-2.26 (m, 7H), 2.20-1.88 (m, 4H), 1.79-1.40 (m, 5H), 1.26 (t, 3H, *J* = 7.1 Hz). ^{13}C NMR (101 MHz, $CDCl_3$) δ 175.5, 170.5, 158.1, 137.8, 126.1, 115.3, 71.4, 60.4, 51.8, 42.0, 29.0, 23.7, 20.5, 14.4. HRMS (ESI-MS) calculated for **59** $C_{19}H_{27}O_5^+$ $[M + H]^+$: 335.1853, found; 335.1856.

Methoxy ether **60**

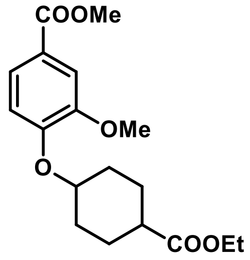

Following General Procedure A: a mixture of methyl vanillate (0.239 g, 1.31 mmol) and ethyl 4-hydroxycyclohexanecarboxylate (0.21 mL, 0.23 g, 1.3 mmol), PPh_3 (0.378 g, 1.44 mmol) and DIAD (0.39 mL, 0.39 g, 2.0 mmol) were subjected to reaction. The crude mixture was subjected to silica gel column chromatography (MeOH:DCM:Et_3N, 0:99:1 to 4:95:1) to afford the title compound as a 4:1 mixture of diastereomers (0.114 g, 26%) as a colourless oil.

^{1}H NMR (400 MHz, $CDCl_3$) δ 7.65-7.57 (m, 1H), 7.54-7.52 (m, 1H), 6.98-6.76 (m, 1H), 4.53-4.24 (m, 1H), 4.15-4.08 (m, 2H), 3.92-3.84 (m, 6H), 2.44-2.29 (m, 1H), 2.29-1.92 (m, 4H), 1.77-1.49 (m, 4H), 1.30-1.22 (m, 3H). ^{13}C NMR (101 MHz, $CDCl_3$) δ 175.4, 175.2, 167.0, 151.3, 151.2, 150.3, 149.8, 123.5, 123.1, 123.0, 114.8, 113.9, 113.3, 112.9, 76.5, 60.5, 60.4, 56.3, 56.2, 52.1, 52.1, 42.3, 41.6, 30.9, 29.1, 27.1, 24.0, 14.4, 14.4. LRMS (ESI-MS) calculated for **60** $[M + H]^+$: $C_{15}H_{19}O_6^+$: 295.1; found 295.3.

Methoxy ether **61**

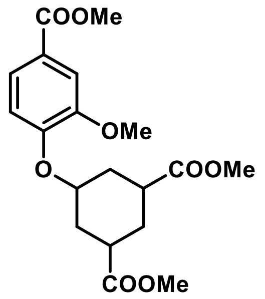

Following General Procedure A: a mixture of methyl vanillate (0.239 g, 1.31 mmol), dimethyl 5-hydroxycyclohexane-1,3-dicarboxylate (0.21 mL, 0.23 g, 1.3 mmol), PPh_3 (0.378 g, 1.44 mmol) and DIAD (0.38 mL, 0.39 g, 2.0 mmol) were subjected to reaction. The crude mixture was subjected to silica gel column chromatography (1:19 to 3:20, EtOAc:Toluene) to afford the title compound as a mixture of diastereomers (0.090 g, 19%) as a white amorphous solid.

^{1}H NMR (400 MHz, $CDCl_3$) δ 7.61 (dd, 1H, *J* = 8.4, 2.0 Hz), 7.54 (d, 1H, *J* = 2.0 Hz), 6.90 (d, 1H, *J* = 8.5 Hz), 4.82 (d, 1H, *J* = 12.1 Hz), 3.87 (s, 3H), 3.87 (s, 3H) 3.65 (s, 6H), 2.94 (tt, 2H, J = 12.5, 3.6 Hz), 2.39-2.27 (m, 3H), 1.59 (m, 3H). ^{13}C NMR (101 MHz, $CDCl_3$) δ = 175.4, 166.9, 150.6, 150.4, 123.7, 123.4, 115.2, 113.2, 72.5, 56.1, 53.5, 52.1, 51.9, 37.0, 31.5, 30.9. LRMS (ESI-MS) calculated for **61** $[M + H]^+$: $C_{16}H_{19}O_8^+$: 339.1; not found.

Triester **62**

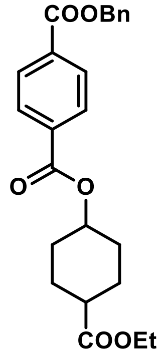

Following General Procedure A: a mixture of 4-((benzyloxy)carbonyl)benzoic acid (0.224 g, 0.87 mmol), ethyl 4-hydroxycyclohexanecarboxylate (0.14 mL, 0.15 g, 0.87 mmol), PPh_3 (0.258 g, 0.96 mmol) and DIAD (0.26 mL, 0.26 g, 1.31 mmol) were subjected to reaction. The crude mixture was subjected to silica gel column chromatography (1:19 to 1:4, EtOAc:PET) to afford the title compound as a 7:3 ratio of diastereomers (0.262 g, 73%) as a white amorphous solid.

Major ^{1}H NMR (400 MHz, $CDCl_3$) δ 8.15-8.06 (m, 4H), 7.47-7.33 (m, 5H), 5.38-5.25 (s, 2H), 4.98 (tt, J = 10.1, 4.2 Hz, 1H), 4.19-4.12 (m, 2H), 2.35 (tt, J = 11.1, 3.7 Hz, 1H), 2.23-2.05 (m, 3H), 2.05-1.81 (m, 2H), 1.76-1.51 (m, 3H), 1.31-1.23 (m, 3H). *Minor* ^{1}H NMR (400 MHz, $CDCl_3$) δ 8.15-8.06 (m, 4H), 7.47-7.33 (m, 5H), 5.38-5.25 (s, 2H), 5.25-5.20 (m, 1H), 4.19-4.12 (m, 2H), 2.44 (tt, J = 9.8, 4.1 Hz, 1H), 2.23-2.05 (m, 3H), 2.05-1.81 (m, 2H), 1.76-1.51 (m, 3H), 1.31-1.23 (m, 3H).

Alcohols **63** and **64**

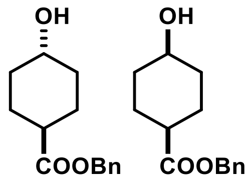

To a solution of benzyl 4-oxocyclohexane-1-carboxylate (0.109 g, 0.47 mmol) in methanol (2 mL) was added $NaBH_4$ (0.043 g, 0.94 mmol) at room temperature. The mixture was stirred overnight, before distilled water was added, and the mixture acidified to pH 1 using 2 M aqueous HCl. The mixture was extracted with EtOAc (2x), before the organic portions were combined, dried over Na_2SO_4, filtered, and concentrated *in vacuo*. The crude mixture was subjected to silica gel column chromatography (1:3 to 1:0, Et_2O:PET) to afford the *anti*-title compound **63** (0.004 g, 3%) as an opaque oil, the *syn*-title compound **64** (0.017 g, 15%) as an opaque oil, and a mixture of **63** and **64** (0.067 g, 61%) as a yellow oil.

syn: ^1H NMR (400 MHz, $CDCl_3$) δ 7.39-7.29 (m, 5H), 5.13 (s, 2H), 3.93-3.86 (m, 1H), 2.50-2.38 (m, 1H), 2.06-1.91 (m, 2H), 1.76-1.59 (m, 6H). ^{13}C NMR (101 MHz, $CDCl_3$) δ 175.2, 136.3, 128.7, 128.3, 128.2, 67.0, 66.2, 41.5, 32.2, 23.8. HRMS (ESI-MS) calculated for **64** $C_{14}H_{19}O_3^+$ [M + H]$^+$: 235.1329, found; 235.1332.

anti: ^1H NMR (400 MHz, $CDCl_3$) δ 7.39-7.29 (m, 5H), 5.11 (s, 2H), 3.61 (tt, 1H, *J* = 10.6, 4.0 Hz), 2.29 (tt, 1H, *J* = 12.0, 3.5 Hz), 2.08-1.97 (m, 4H), 1.54-1.47 (m, 2H), 1.33-1.23 (m, 2H). ^{13}C NMR (101 MHz, $CDCl_3$) δ 175.5, 136.2, 128.7, 128.3, 128.2, 70.0, 66.3, 42.4, 34.6, 27.3. HRMS (ESI-MS) calculated for **63** $C_{14}H_{19}O_3^+$ [M + H]$^+$: 235.1329, found; 235.1331.

Dibenzyl ester **65**

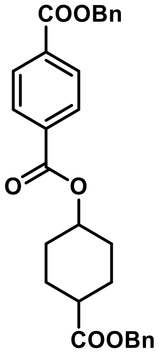

Following General Procedure A: a mixture of 4-((benzyloxy)carbonyl)benzoic acid (0.13 g, 0.51 mmol), benzyl 4-hydroxycyclohexane-1-carboxylate (0.13 g, 0.56 mmol), PPh_3 (0.17 g, 0.62 mol) and DIAD (0.17 mL, 0.17 g, 0.62 mmol) were subjected to reaction. The crude mixture was subjected to silica gel column chromatography (1:19 to 1:3, EtOAc: PET) to afford the title compound as a 2:3 mixture of diastereomers (0.022 g, 9%) as a colourless oil.

Major ^{1}H NMR (400 MHz, $CDCl_3$) δ 8.17-8.07 (m, 4H), 7.48-7.30 (m, 10H), 5.40 (s, 2H), 6.27-5.21 (m, 1H), 5.15 (s, 2H), 2.52 (tt, 1H, *J* = 9.8, 4.1 Hz), 2.22-2.08 (m, 1H), 2.07-1.83 (m, 4H), 1.78-1.63 (m, 2H), 1.63-1.48 (m, 1H). ^{13}C NMR (101 MHz, $CDCl_3$) δ 175.1, 165.8, 166.2, 136.2, 135.8, 134.7, 134.0, 129.8, 129.7, 128.8, 128.7, 128.56, 128.55, 128.4, 128.2, 70.6, 67.3, 66.4, 41.7, 29.2, 24.1. *Minor* ^{1}H NMR (400 MHz, $CDCl_3$) δ 8.17-8.07 (m, 4H), 7.48-7.30 (m, 10H), 5.39 (s, 2H), 5.14 (s, 2H), 5.02-4.90 (m, 1H), 2.42 (tt, 1H, *J* = 11.1, 3.5 Hz), 2.22-2.08 (m, 2H), 2.07-1.83 (m, 2H), 1.78-1.63 (m, 2H), 1.63-1.48 (m, 1H), 1.12 (dd, 1H, *J* = 6.2, 4.5 Hz). ^{13}C NMR (101 MHz, $CDCl_3$) δ 174.9, 165.8, 165.2, 136.1, 135.8, 134.6, 134.0, 129.8, 129.7, 128.7, 128.6, 128.41, 128.35, 128.2, 128.0, 73.2, 67.3, 66.4, 42.1, 30.6, 26.8. HRMS (ESI-MS) calculated for **65** $C_{29}H_{29}O_6^+$ $[M + H]^+$: 473.1959, not found.

General Procedure B:

To an aromatic ester (1 equiv.) was added Rh/C (10-20% w/w), followed by a 4:1 mixture of EtOAc and acetic acid. The vessel was transferred to a sealed hydrogenation chamber, pressurized to 10 atmospheres of H_2 and vented to atmospheric pressure twice, before being pressurized to 10 atmospheres of H_2 and heated to 100 °C for 16 h. At completion, the mixture was filtered through celite using MeOH, before the solvent was removed in vacuo. Typically, the hydrogenated mixtures were carried forward immediately to the hydrolysis step (General Procedure C).

Diester **66**

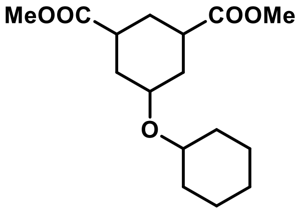

Following General Procedure B: diester **39** (0.047 g, 0.16 mmol) and Rh/C (0.010 g) were subjected to reaction. Workup provided a white solid that was immediately subjected to subsequent reaction.

^{1}H NMR (400 MHz, $CDCl_3$) δ 3.82-3.56 (s, 6H), 3.43-3.33 (m, 2H), 2.36 (tt, J = 12.6, 3.4 Hz, 2H), 2.29-2.15 (m, 2H), 1.96-1.66 (m, 4H), 1.66-1.43 (m, 3H), 1.43-0.82 (m, 7H). ^{13}C NMR (101 MHz, $CDCl_3$) δ 174.6, 75.3, 73.3, 51.9, 41.0, 35.2, 33.1, 30.4, 25.7, 24.3. HRMS (ESI-MS) calculated for **66** $C_{16}H_{27}O_5^+$ $[M + H]^+$: 299.1853, found; 299.1856.

Triester **67**

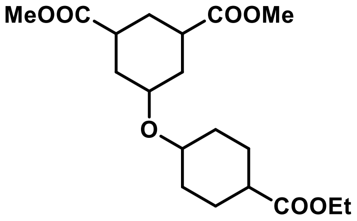

Following General Procedure B: triester **41** (0.032 g, 0.088 mmol) and Rh/C (0.021 g) were subjected to reaction. Workup provided the title compound as a mixture of diastereomers (0.031 g, 95%) as a pale yellow semi-solid that was immediately subjected to subsequent reaction.

NMR data reported for the highest intensity diastereomer: ^{1}H NMR (400 MHz, $CDCl_3$) δ 4.18-4.08 (m, 2H), 3.69-3.67 (m, 8H), 2.42-2.28 (m, 2H), 2.28-2.15 (m, 3H), 2.11-1.94 (m, 4H), 1.93-1.58 (m, 2H), 1.58-1.32 (m, 4H), 1.31-1.19 (m, 5H). ^{13}C NMR (101 MHz, $CDCl_3$) δ 175.6, 174.5, 74.9, 73.7, 60.3, 51.9, 42.4, 40.9, 35.2, 32.1, 30.3, 27.2, 14.2. HRMS (ESI-MS) calculated for **67** $C_{19}H_{31}O_7^+$ $[M + H]^+$: 371.2064, found; 371.2066.

Triester **68**

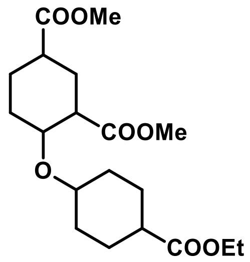

Following General Procedure B: triester **42** (0.052 g, 0.14 mmol) and Rh/C (0.010 g) were subjected to reaction. Workup provided a clear semi-solid that was immediately subjected to subsequent reaction.

^{1}H NMR (400 MHz, $CDCl_3$) δ 4.24-3.85 (m, 2H), 3.84-3.14 (m, 6H), 2.87-2.14 (m, 4H), 2.12-1.17 (m, 18H). ^{13}C NMR (101 MHz, $CDCl_3$) δ 175.8, 175.7, 175.7, 173.5, 173.4, 75.4, 71.4, 71.0, 60.4, 60.2, 51.81, 51.80, 51.76, 51.7, 47.0, 46.9, 42.6, 42.3, 42.24, 42.18, 39.1, 34.7, 32.6, 32.2, 31.1, 31.1, 30.9, 29.4, 29.3, 29.1, 28.7, 28.6, 28.4, 28.0, 27.3, 27.1, 25.5, 24.9, 24.70, 24.67, 24.0, 23.92, 23.85, 23.5, 23.4, 22.4, 22.2, 14.37, 14.35. HRMS (ESI-MS) calculated for **68** $C_{19}H_{31}O_7^+$ [M + H]$^+$: 371.2064, found; 371.2065.

Triester **69**

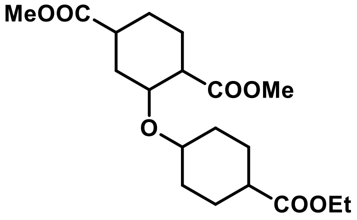

Following General Procedure B: triester **43** (0.172 g, 0.47 mmol) and Rh/C (0.037 g) were subjected to reaction. Workup provided the title compound as a mixture of diastereomers (0.061 g, 35%) as a clear semi-solid that was immediately subjected to subsequent reaction.

^{1}H NMR (400 MHz, $CDCl_3$) δ 4.67 (tt, 0.4 H, *J* = 10.7, 3.9 Hz), 4.13-4.05 (m, 2H), 3.87-3.79 (m, 0.6H), 3.73-3.45 (m, 6.6H), 3.26 (tt, 0.4H, *J* = 10.3, 3.6 Hz), 2.72-2.08 (m, 5H), 1.97-1.31 (m, 11H), 1.26-1.02 (m, 4H). ^{13}C NMR (101 MHz, $CDCl_3$) δ 175.84, 175.79, 175.68, 175.67, 175.65, 175.4, 173.5, 173.4, 75.4, 71.4, 71.0, 60.37, 60.35, 60.2, 51.81, 51.80, 51.76, 51.7, 47.0, 46.9, 42.6, 42.4, 42.3, 42.24, 42.18, 39.1, 34.7, 32.6, 32.2, 31.14, 31.11, 30.9, 29.4, 29.3, 29.1, 28.7, 28.6, 28.4, 28.0, 27.3, 27.2, 27.1, 25.5, 24.9, 24.70, 24.67, 24.0, 23.92, 23.85, 23.5, 23.4, 22.4, 22.2, 14.37, 14.35. HRMS (ESI-MS) calculated for **69** $C_{19}H_{31}O_7^+$ [M + H]$^+$: 371.2064, found; 371.2065.

Triester **70**

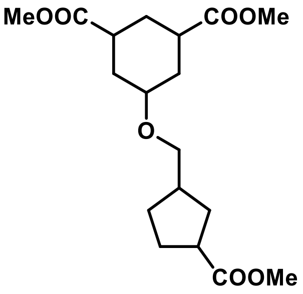

Following General Procedure B: triester **46** (0.014 g, 0.14 mmol) and Rh/C (0.004 g) were subjected to reaction. Workup provided a clear semi-solid that was immediately subjected to subsequent reaction.

Triester **71**

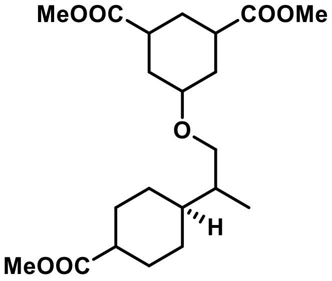

Following General Procedure B: triester **49** (0.064 g, 0.14 mmol) and Rh/C (0.032 g) were subjected to reaction. Workup provided the title compound as a mixture of diastereomers (0.034 g, 60%) as a clear semi-solid that was immediately subjected to subsequent reaction.

^{1}H NMR (400 MHz, $CDCl_3$) δ 4.07-3.66 (m, 10H), 3.43-3.18 (m, 2H), 2.74-2.54 (m, 1H), 2.51-2.13 (m, 5H), 2.09-1.88 (m, 4H), 1.79-1.63 (m, 2H), 1.62-1.42 (m, 5H), 1.41-1.29 (m, 4H), 1.28-1.21 (m, 2H), 1.17-0.94 (m, 1H), 0.92-0.80 (m, 3H). ^{13}C NMR (101 MHz, $CDCl_3$) δ 176.7, 176.0, 174.70, 174.68, 76.60, 76.57, 72.00, 71.99, 52.03, 52.02, 51.64, 51.59, 43.6, 41.0, 30.0, 39.08, 39.05, 38.7, 38.5, 37.5, 34.46, 34.45, 34.40, 34.39, 30.60, 30.60, 29.9, 29.7, 29.4, 29.3, 29.2, 27.9, 27.20, 27.17, 27.1, 25.7, 22.1, 21.2, 14.4, 14.0. HRMS (ESI-MS) calculated for **71** $C_{21}H_{34}O_7^+$ $[M + H]^+$: 399.2377, found; 399.2385.

Tetraester **72**

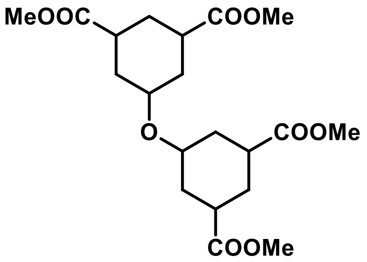

Following General Procedure B: tetraester **50** (0.082 g, 31% purity (contaminated with reduced DIAD) 0.061 mmol) and Rh/C (0.014 g) were subjected to reaction. Workup provided the title compound as a single diastereomer (0.076 g, contaminated with reduced DIAD (27% purity) 81% yield) as a pale yellow semi-solid that was immediately subjected to subsequent reaction.

^{1}H NMR (400 MHz, $CDCl_3$) δ 3.70-3.67 (m, 12H), 3.43 (tt, 2H, *J* = 11.1, 4.2 Hz), 2.35 (tt, 4H, *J* = 12.5, 3.5 Hz), 2.29-2.16 (m, 6H), 1.56-1.47 (m, 2H), 1.40-1.37 (m, 4H). ^{13}C NMR (101 MHz, $CDCl_3$) δ 174.5, 74.1, 52.1, 41.0, 35.1, 30.4. HRMS (ESI-MS) calculated for **72** $C_{20}H_{31}O_9^+$ $[M + H]^+$: 415.1963, found; 415.1961.

Triester **73**

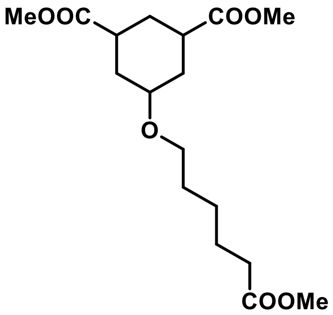

Following General Procedure B: triester **51** (0.063 g, 0.19 mmol) and Rh/C (0.012 g) were subjected to reaction. Workup provided the title compound as a 1:2 mixture of diastereomers (0.054 g, 82%) as an opaque semi-solid that was immediately subjected to subsequent reaction.

Major ^{1}H NMR (400 MHz, $CDCl_3$) δ 3.69-3.66 (m, 9H), 3.46 (t, 2H, *J* = 6.5 Hz), 3.25 (tt, 1H, *J* = 11.1, 3.9 Hz), 2.41-2.27 (m, 5H), 2.26-2.15 (m, 1H), 1.80-1.45 (m, 6H), 1.45-1.22 (m, 4H). ^{13}C NMR (101 MHz, $CDCl_3$) δ 174.6, 174.3, 76.4, 68.3, 52.0, 51.6, 40.9, 34.4, 34.1, 30.6, 29.8, 25.9, 24.9. *Minor* ^{1}H NMR (400 MHz, $CDCl_3$) δ 4.05 (t, 2H, *J* = 6.6 Hz), 3.69-3.66 (m, 10H), 2.41-2.27 (m, 5H), 2.26-2.15 (m, 1H), 1.80-1.45 (m, 6H), 1.45-1.22 (m, 4H). ^{13}C NMR data not reported due to insufficient S:N for minor diastereomer. HRMS (ESI-MS) calculated for **73** $C_{17}H_{29}O_7^+$ $[M + H]^+$: 345.1908, found; 345.1901.

Triester **74**

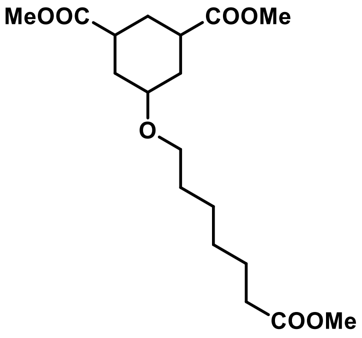

Following General Procedure B: triester **52** (0.097 g, 0.28 mmol) and Rh/C (0.018 g) were subjected to reaction. Workup provided the title compound as a 1:6 mixture of diastereomers (0.080 g, 79%) as an opaque semi-solid that was immediately subjected to subsequent reaction.

Major ^{1}H NMR (400 MHz, $CDCl_3$) δ 3.69-3.66 (m, 9H), 3.45 (t, 2H, *J* = 6.4 Hz), 3.25 (tt, 1H, *J* = 11.2, 3.9 Hz), 2.39-2.28 (m, 5H), 2.24-2.15 (m, 1H), 1.75-1.38 (m, 6H), 1.38-1.24 (m, 6H). ^{13}C NMR (101 MHz, $CDCl_3$) δ 174.7, 174.4, 76.4, 68.5, 52.0, 51.6, 41.0, 34.4, 34.2, 30.6, 30.0, 29.1, 26.0, 25.0. *Minor* ^{1}H NMR (400 MHz, $CDCl_3$) δ 4.04 (t, 2H, *J* = 6.7 Hz), 3.69-3.66 (m, 10H), 2.39-2.28 (m, 5H), 2.24-2.15 (m, 1H), 1.75-1.38 (m, 6H), 1.38-1.24 (m, 6H). ^{13}C NMR data not reported due to insufficient S:N for minor diastereomer. HRMS (ESI-MS) calculated for **74** $C_{18}H_{31}O_7^+$ $[M + H]^+$: 359.2064, found; 359.2066.

Triester **75**

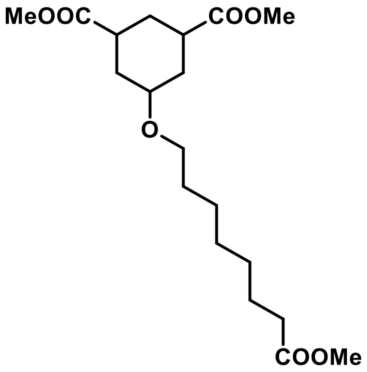

Following General Procedure B: triester **53** (0.120 g, 0.33 mmol) and Rh/C (0.021 g) were subjected to reaction. Workup provided the title compound as a 1:6 mixture of diastereomers (0.115 g, 93%) as a pale yellow semi-solid that was immediately subjected to subsequent reaction.

Major ^{1}H NMR (400 MHz, $CDCl_3$) δ 3.72-3.67 (m, 9H), 3.48 (t, 2H, *J* = 6.6 Hz), 3.28 (tt, 1H, *J* = 11.1, 4.0 Hz), 2.42-2.19 (m, 7H), 1.70-1.50 (m, 5H), 1.41-1.27 (m, 8H). ^{13}C NMR (101 MHz, $CDCl_3$) δ 176.7, 174.4, 76.3, 68.6, 52.0, 51.6, 41.0, 34.5, 32.2, 30.6, 20.1, 29.20, 29.20, 26.1, 20.0. *Minor* ^{1}H NMR (400 MHz, $CDCl_3$) δ 4.07 (t, 2H, *J* = 6.8 Hz), 3.72-3.67 (m, 10H), 2.42-2.19 (m, 7H), 1.70-1.50 (m, 5H), 1.41-1.27 (m, 8H). ^{13}C NMR data not reported due to insufficient S:N for minor diastereomer. HRMS (ESI-MS) calculated for **75** $C_{19}H_{33}O_7^+$ $[M + H]^+$: 373.2221, found; 373.2223.

Triester **76**

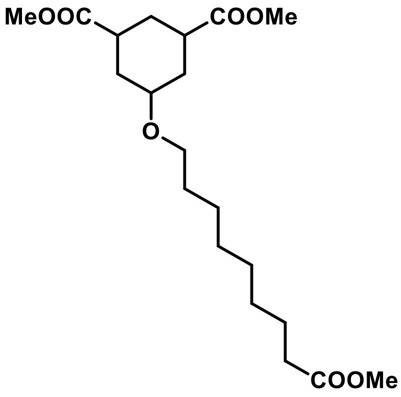

Following General Procedure B: triester **54** (0.095 g, 0.33 mmol) and Rh/C (0.021 g) were subjected to reaction. Workup provided the title compound as a 1:6 mixture of diastereomers (0.115 g, 93%) as a pale yellow semi-solid that was immediately subjected to subsequent reaction.

Major ^{1}H NMR (400 MHz, $CDCl_3$) δ 3.70-3.65 (m, 9H), 3.45 (t, 2H, *J* = 6.6 Hz), 3.25 (tt, 1H, *J* = 11.1, 3.9 Hz), 2.40-2.28 (m, 5H), 2.23-2.16 (m, 1H), 1.75-1.44 (m, 6H), 1.37-1.24 (m, 10H). ^{13}C NMR (101 MHz, $CDCl_3$) δ 174.7, 174.5, 76.3, 68.6, 52.0, 51.6, 41.0, 34.5, 34.2, 30.6, 30.2, 29.4, 29.3, 29.2, 26.2, 25.1. *Minor* ^{1}H NMR (400 MHz, $CDCl_3$) δ 4.04 (t, 2H, *J* = 6.8 Hz), 3.70-3.65 (m, 10H), 2.40-2.28 (m, 5H), 2.23-2.16 (m, 1H), 1.75-1.44 (m, 6H), 1.37-1.24 (m, 10H). ^{13}C NMR data not reported due to insufficient S:N for minor diastereomer. HRMS (ESI-MS) calculated for **76** $C_{20}H_{35}O_7^+$ [M + H]$^+$: 387.2377, found; 387.2378.

Alcohol **77**

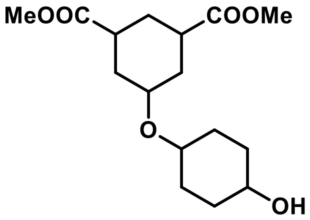

Following General Procedure B: compound **56** (0.067 g, 0.47 mmol) and Rh/C (0.015 g) were subjected to reaction. Workup provided an opaque semi solid that was immediately subjected to subsequent reaction.

^{1}H NMR (400 MHz, $CDCl_3$) δ 4.93-4.69 (m, 1H), 3.71-3.56 (m, 6H), 3.56-3.32 (m, 1H), 2.88-2.56 (m, 1H), 2.45-2.13 (m, 2H), 2.07-1.57 (m, 10H), 1.57-1.18 (m, 5H). HRMS (ESI-MS) calculated for **77** $C_{16}H_{27}O_6^+$ [M + H]$^+$: 315.1802, found; 315.1805.

Diacid **21**

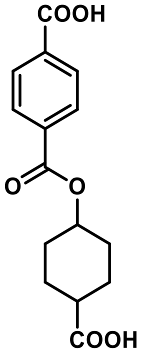

To triester **64** (0.006 g, 0.01 mmol) was added Pd/C (0.003 g , 50% w/w), followed by a 4:1 mixture of EtOAc and acetic acid. The vessel was transferred to a sealed hydrogenation chamber, pressurized to 10 atmospheres of H_2 and vented to atmospheric pressure twice, before being pressurized to 10 atmospheres of H_2 and heated to 80 °C for 16 h. At completion, the mixture was filtered through celite using MeOH, before the solvent was removed in vacuo which provided an opaque semi solid (0.001 g, 26%).

^{1}H NMR (400 MHz, MeOD) δ 8.15-8.06 (m, 4H), 5.22-5.19 (m, 1H), 2.52-2.27 (m, 1H), 2.21-2.07 (m, 1H), 2.07-2.01 (m, 1H), 1.97-1.69 (m, 4H), 1.69-1.56 (m, 1H), 1.39-1.22 (m, 1H). HRMS (ESI-MS) calculated for **21** $C_{15}H_{15}O_6^-$ [M - H]$^-$: 291.0874, found; 291.0879.

Carboxylic acid **37**

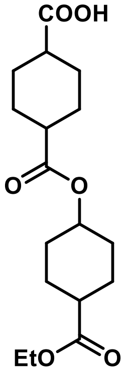

Following General Procedure B: triester **62** (0.062 g, 0.15 mmol) and Rh/C (0.010 g) were subjected to reaction. Workup provided a 7:3 ratio of diastereomers (0.001 g, 1%) as an opaque semi solid. As this reaction initially was meant as a test reaction, a carbon NMR was not procured.

^{1}H NMR (400 MHz, MeOD) δ 4.97-4.70 (m, 1H), 4.17-4.10 (m, 2H), 3.89-3.86, (m, 1H), 2.51-2.38 (m, 1H), 2.38-2.22 (m, 1H), 2.17-2.00 (m, 4H), 1.91-1.80 (m, 4H), 1.77-1.53 (m, 6H), 1.45-1.33 (m, 2H), 1.28-1.19 (m, 3H). HRMS (ESI-MS) calculated for **21** $C_{17}H_{25}O_6^-$ [M - H]$^-$: 325.1657, found; 325.1664.

Carboxylic acid **38**

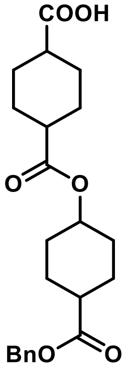

Following General Procedure B: Triester **65** (0.006 g, 0.01 mmol) and Rh/C (0.003 g) were subjected to reaction. Workup provided an opaque semi solid that contained the title compound **38** which was analysed immediately by LCMS2.

^{1}H NMR (400 MHz, MeOD) 8.15-8.09 (m, 5H), 5.34-4.94 (m, 1H), 4.19-3.87 (m, 2H), 2.61-0.90 (m, 19H). HRMS (ESI-MS) calculated for **38** $C_{15}H_{21}O_6^-$ $[M-H]^-$: 387.1813, found: 387.1820.

Dicarboxylic acid **36**

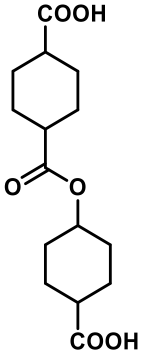

To triester **38** (0.001 g, 0.002 mmol) was added Rh/C (0.001 g), followed by a 4:1 mixture of MeOH and acetic acid. The vessel was transferred to a sealed hydrogenation chamber, pressurized to 10 atmospheres of H_2 and vented to atmospheric pressure twice, before being pressurized to 10 atmospheres of H_2 and heated to 60 °C for 16 h. At completion, the mixture was filtered through celite using MeOH, before the solvent was removed in vacuo which provided an opaque semi solid (0.001 g).

^{1}H NMR (400 MHz, MeOD) δ 3.97-3.65 (m, 1H), 2.79-2.13 (m, 1H), 2.13-1.86 (m, 6H), 1.86-1.45 (m, 6H), 1.45-1.13 (m, 4H), 1.13-0.73 (m, 2H). HRMS (ESI-MS) calculated for **36** $C_{15}H_{21}O_6^-$ $[M-H]^-$: 297.1344, found: 291.0881.

General Procedure C:

A poly-ester compound was dissolved in either MeOH (aliphatic compounds) or 1:1 MeOH:THF (aromatic compounds) before 25% aqueous NaOH was added and the solution stirred vigorously overnight at 50 °C. At completion as monitored by LCMS, the solution was diluted with water and washed with diethyl ether (×3) The aqueous phase was acidified to pH 1 with a 1 N hydrochloric acid solution, before being extracted with ethyl acetate (×3), before the organic portions were combined and reduced *in vacuo*.

Dicarboxylic acid **1**

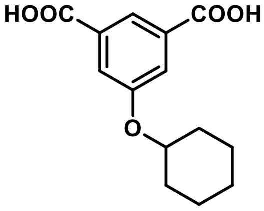

Following General Procedure C: diester **39** (0.058 g, 0.198 mmol) was subjected to reaction. The crude mixture was subjected to silica gel column chromatography (MeOH:DCM:AcOH, 0:1000:1 to 40:960:1) to afford the title compound (0.011 g, 20%) as a white amorphous solid.

^{1}H NMR (400 MHz, MeOD) δ 8.20 (t, J = 1.5 Hz, 2H), 7.71 (d, J = 1.4 Hz, 2H), 4.50 (tt, J = 3.8, 8.8 Hz, 1H), 2.08-1.79 (m, 4H), 1.64-1.28 (m, 6H). ^{13}C NMR (101 MHz, MeOD) δ 169.0, 159.3, 134.0, 124.0, 122.0, 76.9, 32.6, 26.7, 24.6. HRMS (ESI-MS) calculated for **1** $C_{14}H_{15}O_5^-$ [M-H]$^-$: 263.0925, found: 263.0926.

Dicarboxylic acid **2**

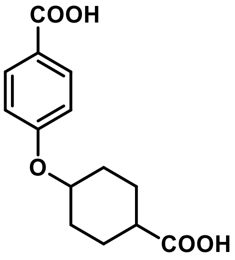

Following General Procedure C: diester **40** (0.026 g, 0.081 mmol) was subjected to reaction to afford the title compound without further purification as a 4:1 mixture of diastereomers (0.018 g, quantitative) as an amorphous white solid.

^{1}H NMR (400 MHz, MeOD) δ 7.97-7.92 (m, 2H), 7.00-6.95 (m, 2H), 4.66-4.36 (m, 1H), 2.36 (tt, J = 11.3, 3.8 Hz, 1H), 2.23-2.04 (m, 3H), 2.00-1.87 (m, 1H), 1.80-1.23 (m, 4H). ^{13}C NMR (101 MHz, MeOD) δ 179.2, 169.9, 163.1, 132.9, 123.9, 116.3, 116.1, 76.2, 43.2, 35.3, 31.8, 29.7, 27.8, 24.8. HRMS (ESI-MS) calculated for **2** $C_{14}H_{15}O_5^-$ [M-H]$^-$: 263.0925, found: 263.0926.

Tricarboxylic acid **3**

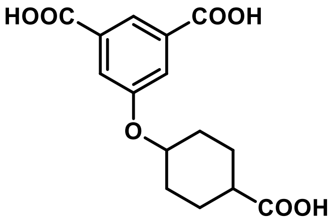

Following General Procedure C: triester **41** (0.021 g, 0.058 mmol) was reacted to afford the title compound as a 1:6 mixture of diastereomers (0.021 g, quantitative) as a white amorphous solid.

Major ^{1}H NMR (400 MHz, MeOD) δ 8.21 (t, 1H, *J* = 1.4 Hz), 7.73 (d, 2H, *J* = 1.4 Hz), 4.40 (tt, 1H, *J* = 9.9, 3.9 Hz), 2.37 (tt, 1H, *J* = 11.1, 3.7 Hz), 2.24-2.15 (m, 2H), 2.15-2.05 (m, 2H), 1.71-1.70 (m, 2H), 1.57-1.48 (m, 2H). ^{13}C NMR (101 MHz, MeOD) δ 179.1, 168.7, 159.3, 133.8, 124.2, 122.0, 76.8, 43.2, 31.7, 27.8. *Minor* ^{1}H NMR (400 MHz, MeOD) δ 8.21 (t, 1H, *J* = 1.4 Hz), 7.76 (d, 2H, *J* = 1.4 Hz), 4.68-4.62 (m, 1H), 2.50-2.42 (m, 1H), 2.02-1.89 (m, 3H), 1.84-1.71 (m, 3H). ^{13}C NMR data not reported due to insufficient S:N for minor diastereomer. HRMS (ESI-MS) calculated for **3** $C_{15}H_{15}O_7^-$ $[M-H]^-$: 307.0823, found: 307.0829.

Tricarboxylic acid **4**

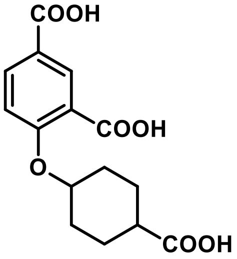

Following General Procedure C: triester **42** (0.041 g, 0.11 mmol) was reacted and title compound was received (0.026 g, 76%) as a white amorphous solid.

Major ^{1}H NMR (400 MHz, MeOD) δ 8.39 (d, J = 2.3 Hz, 1H), 8.12 (dd, J = 8.8, 2.3 Hz, 1H), 7.24 (d, J = 9.1 Hz, 1H), 4.57 (tt, J = 9.4, 4.0 Hz, 1H), 2.46-2.36 (m, 1H), 2.26-1.93 (m, 4H), 1.93-1.81 (m, 1H), 1.72-1.54 (m, 3H). ^{13}C NMR (101 MHz, MeOD) δ 179.0, 169.3, 168.8, 161.8, 135.9, 134.5, 123.8, 123.0, 115.4, 77.7, 42.8, 31.3, 29.8, 27.4, 24.6. *Minor* ^{1}H NMR (400 MHz, MeOD) δ 8.40 (d, J = 1.8 Hz, 1H), 8.12 (dd, J = 8.8, 1.7 Hz, 1H), 7.24 (d, J = 9.1 Hz, 1H), 4.83-4.79 (m, 1H), 2.46-2.36 (m, 1H), 2.26-1.93 (m, 4H), 1.93-1.81 (m, 1H), 1.72-1.54 (m, 3H). ^{13}C NMR (101 MHz, MeOD) δ 179.2, 169.3, 168.8, 161.8, 135.9, 134.6, 123.8, 123.0, 115.1, 74.3, 42.8, 31.3, 29.8, 27.4, 24.6. HRMS (ESI-MS) calculated for **4** $C_{15}H_{15}O_7^-$ $[M-H]^-$: 307.0823, found: 307.0830.

Tricarboxylic acid **5**

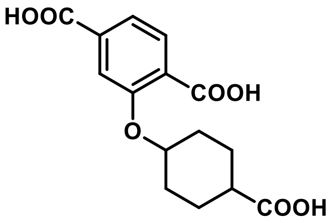

Following General Procedure C: triester **43** (0.134 g, 0.37 mmol) was reacted and title compound was received as a 9:1 mixture of diastereomers (0.102 g, 89%) as a white amorphous solid.

Major ^{1}H NMR (400 MHz, MeOD) δ 7.77 (d, J = 7.9 Hz, 1H), 7.71 (d, J = 1.6 Hz, 1H), 7.64 (dd, J = 8.0, 1.5 Hz, 1H), 4.47 (tt, J = 8.3, 3.9 Hz, 1H), 2.47-2.33 (m, 1H), 2.30-1.87 (m, 4H), 1.86-1.53 (m, 4H). ^{13}C NMR (101 MHz, MeOD) δ 179.2, 169.5, 168.8, 157.7, 157.5, 136.2, 132.2, 122.7, 117.1, 78.1, 42.9, 31.4, 29.8, 27.5, 24.6. *Minor* ^{1}H NMR (400 MHz, MeOD) δ 7.77 (d, J = 8.0 Hz, 1H), 7.68 (d, J = 1.6 Hz, 1H), 7.62 (dd, J = 8.0, 1.4 Hz, 1H), 4.77-4.72 (m, 1H), 2.47-2.33 (m, 1H), 2.30-1.87 (m, 4H), 1.86-1.53 (m, 4H). ^{13}C NMR (101 MHz, MeOD) δ 179.3, 179.2, 169.8, 168.8, 157.5, 136.1, 127.8, 122.4, 116.6, 74.4, 42.9, 31.4, 29.8, 27.5, 24.6. HRMS (ESI-MS) calculated for **5** $C_{15}H_{15}O_7^-$ $[M-H]^-$: 307.0823, found: 307.0831

Tricarboxylic acid **6**

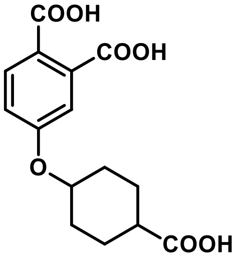

Following General Procedure C: triester **44** (0.165 g, 0.45 mmol) was reacted and title compound was received as a 3:2 mixture of diastereomers (0.092 g, 66%) as a white amorphous solid.

Major ^{1}H NMR (400 MHz, MeOD) δ 7.96 (d, J = 8.8 Hz, 1H), 7.32 (d, J = 2.7 Hz, 1H), 7.08 (dd, J = 8.8, 2.6 Hz, 1H), 4.43 (tt, J = 10.0, 4.0 Hz, 1H), 2.36 (tt, J = 11.3, 3.8 Hz, 1H), 2.25-2.14 (m, 1H), 2.13-2.04 (m, 1H), 2.02-1.85 (m, 2H), 1.83-1.71 (m, 2H), 1.71-1.58 (m, 1H), 1.58-1.45 (m, 1H). ^{13}C NMR (101 MHz, MeOD) δ 177.7, 171.0, 160.3, 132.8, 116.5, 116.3, 75.1, 54.4, 42.4, 41.8, 30.3, 28.3, 26.4, 23.4, 11.8. *Minor* ^{1}H NMR (400 MHz, MeOD) δ 7.97 (d, J = 8.7 Hz, 1H), 7.35 (d, J = 2.6 Hz, 1H), 7.09 (dd, J = 8.7, 2.7 Hz, 1H), 4.69-4.65 (m, 1H), 2.45 (tt, J = 9.8, 3.9 Hz, 1H), 2.25-2.14 (m, 1H), 2.13-2.04 (m, 1H), 2.02-1.85 (m, 2H), 1.83-1.71 (m, 1H), 1.71-1.58 (m, 2H), 1.58-1.45 (m, 1H). ^{13}C NMR (101 MHz, MeOD) δ 177.8, 169.2, 160.1, 136.9, 123.1, 116.8, 72.1, 54.4, 42.4, 41.2, 30.3, 28.3, 26.4, 23.4, 11.8. HRMS (ESI-MS) calculated for **6** $C_{15}H_{15}O_7^-$ $[M-H]^-$: 307.0823, found: 307.0830.

Tricarboxylic acid **7**

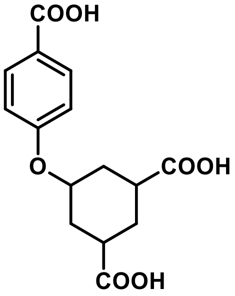

Following General Procedure C: triester **45** (0.043 g, 0.12 mmol) was subjected to reaction. The crude mixture was subjected to silica gel column chromatography (MeOH:DCM:AcOH, 0:1000:1 to 40:960:1) to afford the title compound (0.008 g, 21%) as a white solid.

^{1}H NMR (400 MHz, MeOD) δ 7.99-7.94 (m, 2H), 7.04-6.99 (m, 2H), 4.52 (tt, J = 11.0, 4.1 Hz, 1H), 2.65 (tt, J = 12.7, 3.5 Hz, 1H), 2.58 (tt, J = 12.7, 3.5 Hz, 1H), 2.43-2.22 (m, 3H), 1.53-1.41 (m, 3H). ^{13}C NMR (101 MHz, MeOD) δ = 174.7, 161.4, 131.5, 114.8, 74.0, 40.3, 40.1, 33.8, 33.6, 30.6. HRMS (ESI-MS) calculated for **7** $C_{15}H_{15}O_7^-$ [M-H]$^-$: 307.0823, found: 307.0827.

Tricarboxylic acid **8**

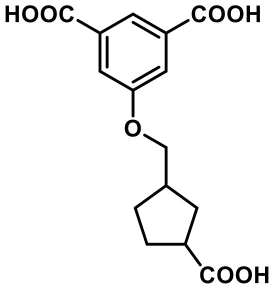

Following General Procedure C: triester **46** (0.026 g, 0.07 mmol) was reacted and title compound was received (0.022 g, 95%) as a white amorphous solid.

^{1}H NMR (400 MHz, MeOD) δ 8.25-8.20 (m, 1H), 7.72 (d, *J* = 1.5 Hz, 2H), 4.01 (d, *J* = 6.7 Hz, 1H), 3.97 (dd, *J* = 6.7, 1.3 Hz, 1H), 2.96-2.81 (m, 1H), 2.62-2.43 (m, 1H), 2.27-1.76 (m, 4H), 1.72-1.46 (m, 2H). ^{13}C NMR (101 MHz, MeOD) δ 180.1, 180.0, 169.0, 160.7, 134.0, 124.1, 120.6, 73.2, 73.1, 45.0, 44.5, 43.5, 40.7, 40.0, 34.4, 33.8, 30.9, 30.3, 30.2, 29.9, 11.6. HRMS (ESI-MS) calculated for **8** $C_{15}H_{15}O_7^-$ [M-H]$^-$: 307.0823, found: 307.0830.

Tricarboxylic acid **9**

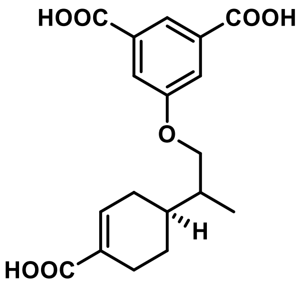

Following General Procedure C: triester **49** (0.053 g, 0.136 mmol) was reacted and title compound was received (0.031 g, 65%) as a white amorphous solid.

^{1}H NMR (400 MHz, MeOD) δ 8.23 (t, J = 1.4 Hz, 1H), 7.74 (d, J = 1.5 Hz, 2H), 7.02-6.98 (m, 1H), 4.12-4.05 (m, 1H), 4.05-3.94 (m, 1H), 2.49-2.44 (m, 1H), 2.37-2.26 (m, 1H), 2.26-2.03 (m, 2H), 2.03-1.86 (m, 2H), 1.86-1.65 (m, 1H), 1.46-1.27 (m, 1H), 1.08-1.02 (m, 3H). ^{13}C NMR (101 MHz, MeOD) δ 170.9, 168.9, 160.7, 140.8, 133.9, 131.6, 124.1, 120.6, 72.8, 38.4, 36.4, 31.2, 29.6, 27.5, 26.1, 25.8, 14.4, 14.0. HRMS (ESI-MS) calculated for **9** $C_{18}H_{19}O_7^-$ $[M-H]^-$: 347.1136, found: 347.1142.

Tetracarboxylic acid **10**

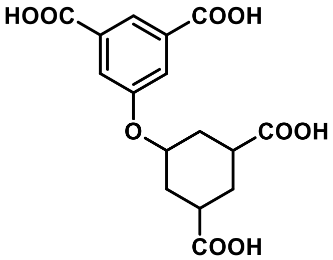

Following General Procedure C: tetraester **50** (0.049 g (31% purity, contaminated with reduced DIAD), 0.037 mmol) was reacted and title compound was received (0.021 g, quantitative) as a white amorphous solid.

^{1}H NMR (400 MHz, MeOD) δ 8.24 (t, 1H, *J* = 1.4 Hz), 7.77 (m, 2H, *J* = 1.4 Hz), 4.54 (tt, 1H, *J* = 10.9, 3.9 Hz), 2.72-2.58 (m, 2H), 2.45-2.36 (m, 2H), 2.34-2.21 (m, 1H), 1.53-1.44 (m, 3H). ^{13}C NMR (101 MHz, MeOD) δ 177.7, 168.7, 159.0, 133.9, 124.5, 122.1, 76.1, 41.5, 35.1, 32.0. HRMS (ESI-MS) calculated for **10** $C_{16}H_{15}O_9^-$ $[M-H]^-$: 351.0722, found: 351.0726.

Tricarboxylic acid **11**

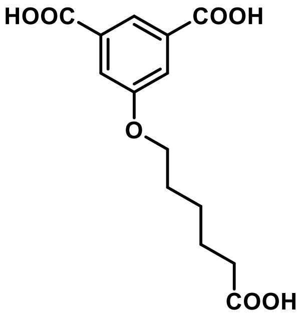

Following General Procedure C: triester **51** (0.037 g, 0.11 mmol) was reacted to afford the title compound (0.029 g, 88%) as a white amorphous solid.

^{1}H NMR (400 MHz, MeOD) δ 8.22 (t, 1H, *J* = 1.4 Hz), 7.74 (d, 2H, *J* = 1.4 Hz), 4.08 (t, 2H, *J* = 6.3 Hz), 2.34 (t, 2H, *J* = 7.4 Hz), 1.88-1.81 (m, 2H), 1.74-1.67 (m, 2H), 1.59-1.52 (m, 2H). ^{13}C NMR (101 MHz, MeOD) δ 177.5, 168.7, 160.7, 133.7, 124.0, 120.7, 69.4, 34.8, 29.9, 26.7, 25.8. HRMS (ESI-MS) calculated for **11** $C_{14}H_{15}O_7^-$ [M-H]$^-$: 295.0823, found: 295.0828.

Tricarboxylic acid **12**

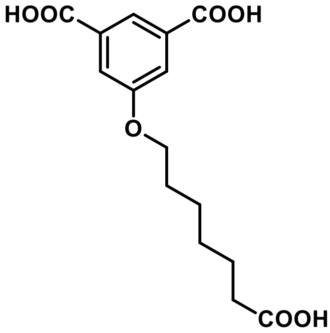

Following General Procedure C: triester **52** (0.054 g, 0.15 mmol) was reacted to afford the title compound (0.037 g, 79%) as a white amorphous solid.

^{1}H NMR (400 MHz, MeOD) δ 8.22 (t, 1H, *J* = 1.4 Hz), 7.74 (d, 2H, *J* = 1.4 Hz), 4.08 (t, 2H, *J* = 6.4 Hz), 2.32 (t, 2H, *J* = 7.4 Hz), 1.87-1.80 (m, 2H), 1.70-1.62 (m, 2H), 1.58-1.50 (m, 2H), 1.48-1.40 (m, 2H). ^{13}C NMR (101 MHz, MeOD) δ 177.7, 168.5, 160.7, 133.7, 124.0, 120.7, 69.5, 34.9, 30.1, 29.9, 26.8, 26.0. HRMS (ESI-MS) calculated for **12** $C_{15}H_{17}O_7^-$ [M-H]$^-$: 309.0980, found: 309.0987.

Tricarboxylic acid **13**

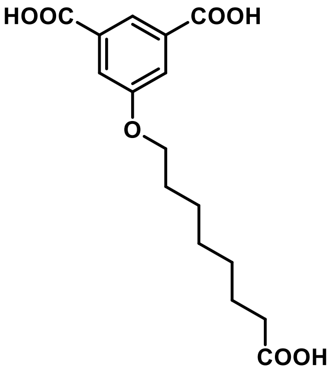

Following General Procedure C: triester **53** (0.072 g, 0.20 mmol) was reacted to afford the title compound (0.060 g, 92%) as a white amorphous solid.

^{1}H NMR (400 MHz, MeOD) δ 8.22 (t, 1H, *J* = 1.4 Hz), 7.73 (d, 2H, *J* = 1.4 Hz), 4.07 (t, 2H, *J* = 6.5 Hz), 2.30 (t, 2H, *J* = 7.5 Hz), 1.86-1.79 (m, 2H), 1.67-1.60 (m, 2H), 1.56-1.49 (m, 2H), 1.47-1.35 (m, 4H). ^{13}C NMR (101 MHz, MeOD) δ 177.7, 168.8, 160.7, 133.7, 124.0, 120.7, 69.6, 34.9, 30.15, 30.14, 26.9, 26.0. HRMS (ESI-MS) calculated for **13** $C_{16}H_{19}O_7^-$ [M-H]$^-$: 323.1136, found: 323.1141.

Tricarboxylic acid **14**

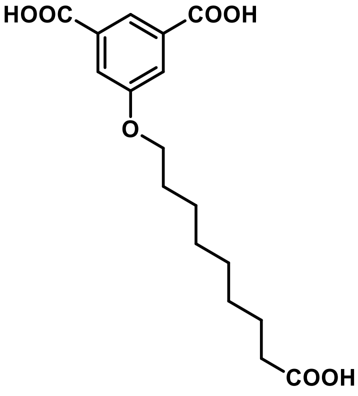

Following General Procedure C: triester **54** (0.059 g, 0.16 mmol) was reacted to afford the title compound (0.054 g, 99%) as a white amorphous solid.

^{1}H NMR (400 MHz, MeOD) δ 8.22 (t, 1H, *J* = 1.5 Hz), 7.72 (d, 2H, *J* = 1.4 Hz), 4.07 (t, 2H, *J* = 6.4 Hz), 2.29 (t, 2H, *J* = 7.43 Hz), 1.86-1.77 (m, 2H), 1.64-1.57 (m, 2H), 1.54-1.44 (m, 2H), 1.44-1.33 (m, 6H). ^{13}C NMR (101 MHz, MeOD) δ 177.7, 168.7, 160.7, 133.7, 124.0, 120.7, 69.6, 34.9, 30.33, 30.32, 30.19, 30.16, 27.0, 26.1. HRMS (ESI-MS) calculated for **14** $C_{17}H_{21}O_7^-$ [M-H]$^-$: 337.1293, found: 337.1300.

Ketone **15**

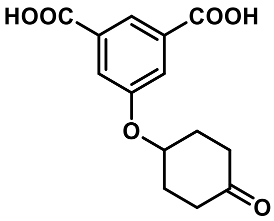

Following General Procedure C: diester **55** (0.129 g, 0.42 mmol) was reacted and title compound was received without further purification as a 7:3 mixture of diastereomers (0.084 g, 71%) as a brown amorphous solid that contained dimerization and polymerization products from likely condensation and Michael reactions, that was immediately analysed by LCMS2 analysis.

HRMS (ESI-MS) calculated for **15** $C_{14}H_{13}O_6^-$ [M-H]$^-$: 277.0718, found: 277.0722.

Alcohol **16**

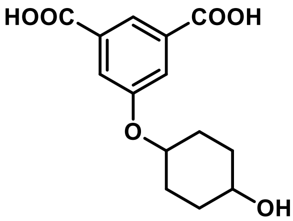

Following General Procedure C: diester **56** (0.097 g, 0.31 mmol) was reacted and title compound was received without further purification as a 3:2 mixture of diastereomers (0.054 g, 61%) as a white amorphous solid.

Major ^{1}H NMR (400 MHz, MeOD) δ 8.22 (t, *J* = 1.4 Hz, 1H), 7.75 (d, *J* = 1.5 Hz, 2H), 4.55 (m, 1H), 3.77-3.68 (m, 1H), 2.19-1.98 (m, 3H), 1.78-1.70 (m, 4H), 1.62-1.43 (m, 1H). ^{13}C NMR (101 MHz, MeOD) δ 168.8, 159.1, 133.9, 124.1, 122.1, 76.4, 74.1, 69.6, 68.9, 30.9, 28.3. *Minor* ^{1}H NMR (400 MHz, MeOD) δ 8.21 (t, J = 1.3 Hz, 1H), 7.72 (d, J = 1.5 Hz, 2H), 4.42 (td, J = 9.2, 4.7 Hz, 1H), 3.77-3.68 (m, 1H), 2.19-1.98 (m, 3H), 1.78-1.70 (m, 4H), 1.62-1.43 (m, 1H). ^{13}C NMR (101 MHz, MeOD) δ 168.8, 159.4, 133.9, 124.1, 122.0, 76.4, 74.1, 69.6, 68.9, 32.7, 29.7. HRMS (ESI-MS) calculated for **16** $C_{14}H_{15}O_6^-$ [M-H]$^-$: 279.0874, found: 279.0877.

Diol **17**

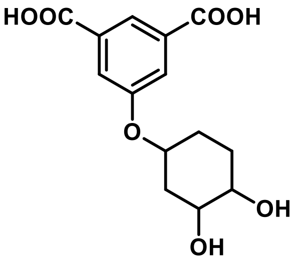

Following General Procedure C: diester **58** (0.011 g, 0.03 mmol) was reacted and title compound was received without further purification as a 7:3 mixture of diastereomers (0.003 g, 33%) as a white amorphous solid.

Major ^{1}H NMR (400 MHz, MeOD) δ 8.22 (t, J = 1.4 Hz, 1H), 7.75 (t, J = 1.6 Hz, 2H), 4.79 (sept, J = 3.5 Hz, 1H), 3.99 (dt, J = 8.0, 3.3 Hz, 1H), 3.83 (td, J = 5.6, 2.8 Hz, 1H), 3.35 (s, 2H), 2.24-1.98 (m, 2H), 1.98-1.52 (m, 4H). ^{13}C NMR (101 MHz, MeOD) δ 168.7, 159.3, 133.8, 124.2, 122.0, 74.2, 71.1, 69.6, 35.1, 27.1, 26.0. *Minor* ^{1}H NMR (400 MHz, MeOD) δ 8.22 (t, J = 1.4 Hz, 1H), 7.75 (t, J = 1.6 Hz, 2H), 4.43 (tt, J = 10.5, 4.1 Hz, 1H), 3.92-3.87 (m, 1H), 3.71 (ddd, J = 11.5, 4.3, 2.8 Hz, 1H), 3.35 (s, 2H), 2.24-1.98 (m, 2H), 1.98-1.52 (m, 4H). ^{13}C NMR (101 MHz, MeOD) δ 168.7, 159.2, 133.8, 124.2, 122.0, 75.8, 70.9, 69.3, 35.3, 28.1, 26.0. HRMS (ESI-MS) calculated for **17** $C_{14}H_{15}O_7^-$ [M-H]$^-$: 295.0823, found: 295.0831.

Dicarboxylic acid **18**

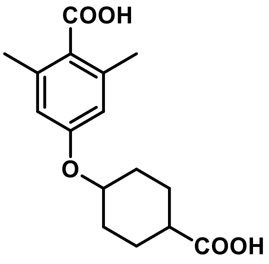

Following General Procedure C: diester **59** (0.037 g, 0.11 mmol) was reacted and title compound was received as a 4:1 mixture of diastereomers (0.088 g, quantitative) as a white amorphous solid.

Major ^{1}H NMR (400 MHz, MeOD) δ 6.61 (s, 1H), 4.29 (tt, J = 10.1, 4.0 Hz, 1H), 2.47-2.36 (m, 1H), 2.31 (s, 6H), 2.18-1.77 (m, 4H), 1.77-1.52 (m, 3H), 1.52-1.39 (m, 2H). ^{13}C NMR (101 MHz, MeOD) δ 159.2, 159.1, 137.9, 116.1, 115.9, 75.9, 31.9, 29.8, 27.9, 24.8, 20.3. *Minor* ^{1}H NMR (400 MHz, MeOD) δ 6.63 (s, 1H), 4.58-4.51 (m, 1H), 2.47-2.36 (m, 1H), 2.32 (s, 6H), 2.18-1.77 (m, 4H), 1.77-1.52 (m, 3H), 1.52-1.39 (m, 2H). ^{13}C NMR data not reported due to insufficient S:N for minor diastereomer. HRMS (ESI-MS) calculated for **18** $C_{16}H_{19}O_5^-$ [M-H]$^-$: 291.1238, found: 291.1242.

Methoxy ether **19**

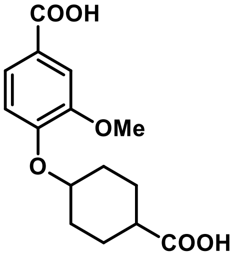

Following General Procedure C: diester **60** (0.066 g, 0.18 mmol) was reacted. The crude mixture was subjected to silica gel column chromatography (MeOH:DCM:AcOH, 0:1000:1 to 40:950:1) to afford the title compound (0.052 g, 88%) as a white amorphous solid.

^{1}H NMR (400 MHz, MeOD) δ 7.67-7.54 (m, 2H), 7.05-7.01 (m, 1H), 4.61-4.34 (m, 1H), 3.90-3.86 (m, 3H), 2.45-2.31 (m, 1H), 2.23-1.93 (m, 4H), 1.76-1.26 (m, 4H). ^{13}C NMR (101 MHz, MeOD) δ 152.6, 151.1, 124.8, 115.4, 114.3, 77.5, 56.6, 56.4, 43.4, 31.9, 29.9, 28.0, 24.9. HRMS (ESI-MS) calculated for **19** $C_{15}H_{17}O_6^-$ [M-H]$^-$: 293.1031, found: 293.1034.

Methoxy ether **20**

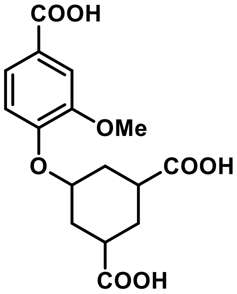

Following the General Procedure C: triester **61** (0.060 g, 0.162 mmol) was subjected to reaction. The crude mixture was subjected to silica gel column chromatography (MeOH:DCM:AcOH, 0:1000:1 to 150:850:1) to afford the title compound (0.013 g, 25%) as a white amorphous solid.

^{1}H NMR (400 MHz, MeOD) δ 7.65-7.60 (m, 2H), 7.07 (d, 1H), 4.90 (m, 1H), 3.89 (s, 3H), 2.87-2.84 (m, 2H), 2.32-2.26 (m, 3H), 1.66-1.50 (m, 3H). ^{13}C NMR (126 MHz, MeOD) δ 179.2, 170.2, 152.0, 151.7, 125.7, 124.7, 116.5, 114.4, 74.1, 56.4, 38.5, 32.9, 32.2. HRMS (ESI-MS) calculated for **20** $C_{16}H_{17}O_8^-$ [M-H]$^-$: 337.0929, found: 337.0936.

Dicarboxylic acid **22**

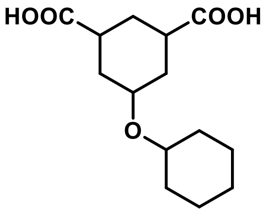

Following General Procedure C: diester **66** (0.006 g, 0.18 mmol) was reacted to afford the title compound (0.010 g, quantitative) as a white amorphous solid.

^{1}H NMR (400 MHz, MeOD) δ 3.69-3.42 (m, 2H), 2.58-2.36 (m, 2H), 2.30-2.01 (m, 3H), 1.93-1.48 (m, 5H), 1.48-0.88 (m, 8H). ^{13}C NMR (101 MHz, MeOD) δ 178.2, 76.9, 75.1, 41.9, 36.6, 34.2, 32.0, 26.8, 25.3. HRMS (ESI-MS) calculated for **22** $C_{14}H_{21}O_5^-$ [M-H]$^-$: 269.1394, found: 269.1395.

Tricarboxylic acid **23**

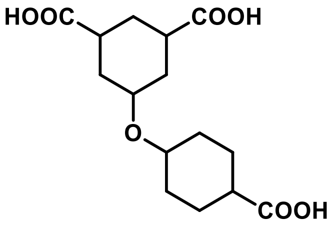

Following General Procedure C: triester **67** (0.015 g, 0.05 mmol) was reacted to afford the title compound (0.030 g, quantitative) as a white amorphous solid.

^{1}H NMR (400 MHz, MeOD) δ 4.25-3.37 (m, 2H), 2.56-2.35 (m, 2H), 2.34-2.12 (m, 3H), 2.12-1.81 (m, 4H), 1.81-1.16 (m, 7H), 0.98-0.88 (m, 1H). ^{13}C NMR (101 MHz, MeOD) δ 178.0, 176.7, 131.0, 128.5, 75.1, 75.0, 73.9, 73.8, 67.7, 42.1, 40.5, 40.4, 38.9, 38.8, 35.2, 35.0, 31.9, 31.8, 30.6, 30.4, 30.2, 28.7, 27.0, 26.9, 23.6, 22.6, 19.1, 13.0, 10.0. HRMS (ESI-MS) calculated for **23** $C_{15}H_{21}O_7^-$ [M-H]$^-$: 313.1293, found: 313.1301.

Tricarboxylic acid **24**

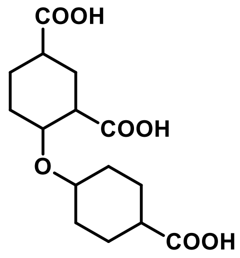

Following General Procedure C: triester **68** (0.041 g, 0.11 mmol) was reacted to afford the title compound (0.021 g, 60%) as a white amorphous solid.

^{1}H NMR (400 MHz, MeOD) δ 4.15-3.47 (m, 2H), 2.55-2.12 (m, 3H), 2.12-1.88 (m, 4H), 1.88-1.50 (m, 7H), 1.50-1.14 (m, 3H). ^{13}C NMR (101 MHz, MeOD) δ 179.7, 179.5, 177.0, 72.8, 67.8, 47.6, 43.2, 40.4, 32.8, 32.1, 29.8, 29.7, 26.1, 25.0, 23.8. HRMS (ESI-MS) calculated for **24** $C_{15}H_{21}O_7^-$ [M-H]$^-$: 313.1293, found: 313.1302.

Tricarboxylic acid **25**

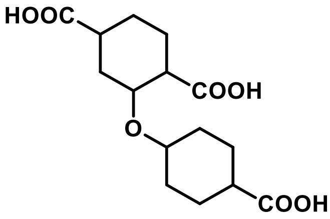

Following General Procedure C: triester **69** (0.025 g, 0.07 mmol) was reacted to afford the title compound (0.016 g, 72%) as a white amorphous solid.

^{1}H NMR (400 MHz, MeOD) δ3.99-3.35 (m, 2H), 2.71-2.54 (m, 1H), 2.53-2.34 (m, 2H), 2.34-1.74 (m, 7H), 1.74-1.37 (m, 5H), 1.37-1.10 (m, 2H). ^{13}C NMR (101 MHz, MeOD) δ 179.5, 178.9, 178.7, 177.1, 133.8, 133.1, 133.0, 130.0, 129.9, 76.2, 73.6, 73.4, 70.5, 52.9, 46.6, 43.6, 41.8, 35.3, 32.8, 32.5, 32.1, 32.0, 31.7, 30.9, 29.4, 28.5, 28.3, 28.2, 27.2, 25.4. HRMS (ESI-MS) calculated for **25** $C_{15}H_{21}O_7^-$ [M-H]$^-$:313.1293, found: 313.1300.

Tricarboxylic acid **26**

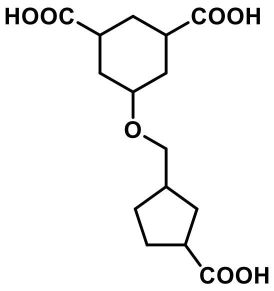

Following General Procedure C: triester **70** (0.013 g, 0.11 mmol) was reacted to afford the title compound (0.021 g, 79%) as a white amorphous solid.

^{1}H NMR (400 MHz, MeOD) δ 3.49-3.33 (m, 3H), 2.86-2.73 (m, 1H), 2.40 (tt, J = 12.6, 3.5 Hz, 1H), 2.32-2.24 (m, 2H) 2.24-2.13 (m, 1H), 2.13-1.92 (m, 2H), 1.91-1.83 (m, 1H), 1.83-1.69 (m, 1H), 1.69-1.59 (m, 1H), 1.59-1.41 (m, 2H), 1.41-1.28 (m, 2H), 1.26-1.17 (m, 2H). ^{13}C NMR (101 MHz, MeOD) δ 180.2, 178.2, 120.5, 78.0, 73.6, 41.9, 41.4, 40.6, 35.8, 34.8, 34.1, 32.1, 30.9, 30.1, 30.0. HRMS (ESI-MS) calculated for **26** $C_{15}H_{21}O_7^-$ [M-H]$^-$: 313.1293, found: 313.1301.

Tricarboxylic acid **27**

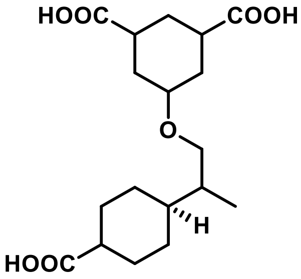

Following General Procedure C: triester **71** (0.034 g, 0.09 mmol) was reacted to afford the title compound (0.014 g, 46%) as a white amorphous solid.

^{1}H NMR (400 MHz, MeOD) δ 3.57-3.44 (m, 1H), 3.41-3.26 (m, 2H), 2.80-2.55 (m, 1H), 2.40 (tt, *J* = 11.7, 3.0 Hz, 2H), 2.26-2.23 (m, 1H), 2.23-2.12 (m, 1H), 2.12-2.05 (m, 1H), 2.03-1.87 (m, 2H), 1.79-1.68 (m, 1H), 1.63-1.47 (m, 3H), 1.47-1.27 (m, 4H), 1.27-1.02 (m, 3H), 0.93-0.85 (m, 3H). ^{13}C NMR (101 MHz, MeOD) δ = 180.1, 179.1, 178.2, 78.0, 73.0, 66.2, 44.7, 43.7, 41.9, 41.7, 40.9, 40.4, 40.3, 40.0, 39.9, 39.7, 39.5, 38.8, 35.8, 35.7, 32.5, 32.1, 31.1, 30.9, 30.6, 30.5, 29.8, 29.0, 28.7, 28.4, 28.1, 26.6, 26.3, 26.0, 14.6, 14.3, 14.0, 13.7. HRMS (ESI-MS) calculated for **27** $C_{18}H_{27}O_7^-$ [M-H]$^-$: 355.1762, found: 355.1767.

Tetracarboxylic acid **28**

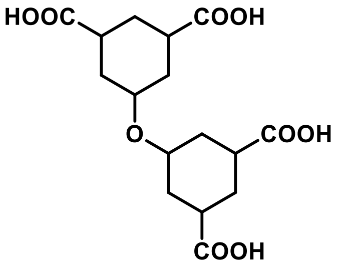

Following General Procedure C: triester **72** (0.076 g, 0.18 mmol) was reacted to afford the title compound (0.029 g, 44%) as a white amorphous solid.

^{1}H NMR (400 MHz, MeOD) δ 3.65-3.49 (m, 2H), 2.57-2.36 (m, 4H), 2.36-2.29 (m, 3H), 2.29-2.09 (m, 2H), 1.47-1.32 (m, 2H), 1.32-1.14 (m, 5H). ^{13}C NMR (101 MHz, MeOD) δ 177.9, 176.4, 75.5, 75.4, 41.7, 36.3, 31.7, 22.3. HRMS (ESI-MS) calculated for **28** $C_{16}H_{21}O_9^-$ [M-H]$^-$: 357.1191, found: 357.1199.

Dicarboxylic acid **29**

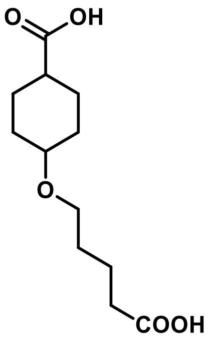

NaH (60% in mineral oil, 0.070 g, 1.60 mmol, 1.1 equiv.) was added to a flask along with dry THF (4 mL), before ethyl 4-hydroxycyclohexanecarboxylate (0.1 g, 0.1 mL, 0.6 mmol, 1 equiv.) was added slowly and the mixture stirred for 15 min. Next, methyl 5-bromovalerate (0.08 mL, 0.13 g, 0.56 mmol, 1 equiv.) was added slowly, before the mixture was left to stir at room temperature overnight. The reaction was monitored by TLC, and at completion, the mixture was diluted into 2 N aqueous HCl (10 mL) before being extracted with EtOAc (×3). The combined organic portions were washed with saturated $NaHCO_3$ (×1) and brine (×1). The organic portion was dried over Na_2SO_4 before solvent was evaporated in vacuo. The material was immediately used in the next step following General Procedure C. The mixture was subsequently subjected to silica gel column chromatography (MeOH:DCM:AcOH, 0:1000:1 to 40:960:1), to afford the title compound (0.011 g, 13%) as a white amorphous solid.

^{1}H NMR (400 MHz, MeOD) δ 3.81-3.37 (m, 3H), 2.41-2.28 (m, 1H), 2.28-2.14 (m, 1H), 2.04-1.91 (m, 4H), 1.70-1.53 (m, 5H), 1.53-1.39 (m, 2H), 1.32-1.20 (m, 2H).^{13}C NMR (101 MHz, MeOD) δ 179.5, 179.2, 177.5, 70.5, 67.8, 62.5, 35.3, 33.0, 32.8, 28.5, 25.0, 22.5. HRMS (ESI-MS) calculated for **29** $C_{12}H_{19}O_5^-$ [M-H]$^-$: 243.1238, found: 243.1236.

Tricarboxylic acid **30**

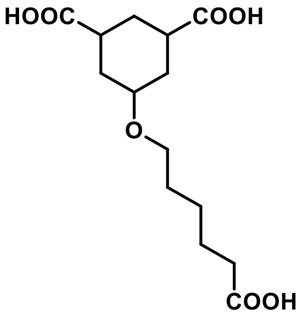

Following General Procedure C: triester **73** (0.054 g, 0.16 mmol) was reacted to afford the title compound (0.045 g, 94%) as a white amorphous solid.

^{1}H NMR (400 MHz, MeOD) δ 3.57-3.32 (m, 3H), 2.47-2.26 (m, 5H), 2.22-2.12 (m, 1H), 1.76-1.50 (m, 5H), 1.50-1.27 (m, 4H), 1.27-1.13 (m, 1H). ^{13}C NMR (101 MHz, MeOD) δ 176.7, 176.2, 76.4, 67.9, 42.2, 40.4, 34.3, 33.5, 30.7, 29.4, 28.4, 25.4, 24.5. HRMS (ESI-MS) calculated for **30** $C_{14}H_{21}O_7^-$ [M-H]$^-$: 301.1293, found; 301.1300.

Tricarboxylic acid **31**

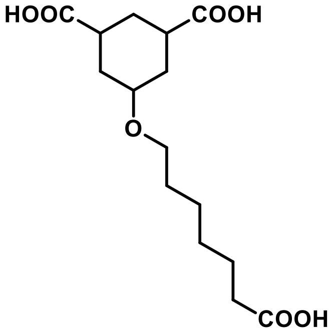

Following General Procedure C: triester **74** (0.080 g, 0.22 mmol) was reacted to afford the title compound (0.072 g, quantitative) as a white amorphous solid.

^{1}H NMR (400 MHz, MeOD) δ 3.57-3.48 (m, 2H), 3.48-3.33 (m, 1H), 2.47-2-35 (m, 2H), 2.35-2.26 (m, 4H), 2.22-2.12 (m, 1H), 1.65- 1.50 (m, 4H), 1.46-1.31 (m, 6H), 1.27-1.14 (m, 1H). ^{13}C NMR (101 MHz, MeOD) δ 176.7, 176.3, 76.3, 68.1, 61.6, 40.4, 34.3, 33.5, 30.7, 29.7, 28.82, 28.79, 25.7, 24.6. HRMS (ESI-MS) calculated for **31** $C_{15}H_{23}O_7^-$ [M-H]$^-$: 315.1449, found: 315.1458.

Tricarboxylic acid **32**

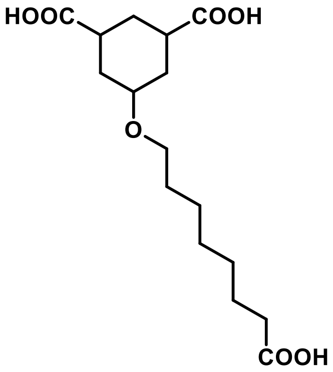

Following General Procedure C: triester **75** (0.115 g, 0.31 mmol) was reacted to afford the title compound (0.099 g, 98%) as a white amorphous solid.

^{1}H NMR (400 MHz, MeOD) δ 3.58-3.48 (m, 2H), 3.48-3.33 (m, 1H), 2.47-2.36 (m, 2H), 2.35-2.26 (m, 4H), 2.22-2.13 (m, 1H), 2.10-1.70 (m, 1H), 1.68-1.50 (m, 5H), 1.47-1.31 (m, 5H), 1.31-1.13 (m, 2H). ^{13}C NMR (101 MHz, MeOD) δ 178.1, 177.7, 77.7, 69.4, 62.9, 41.8, 35.7, 34.9, 32.0, 30.9, 30.0, 26.9, 26.0. HRMS (ESI-MS) calculated for **32** $C_{16}H_{25}O_7^-$ $[M-H]^-$:329.1606, found: 329.1609.

Tricarboxylic acid **33**

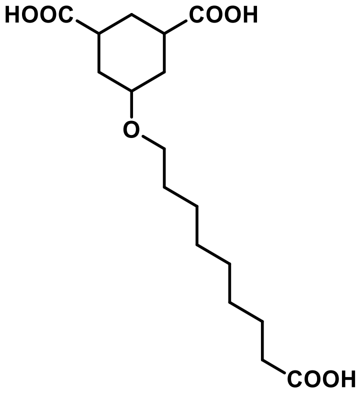

Following General Procedure C: triester **76** (0.086 g, 0.23 mmol) was reacted to afford the title compound (0.079 g, quantitative) as a white amorphous solid.

^{1}H NMR (400 MHz, MeOD) δ 3.56-3-48 (m, 2H), 3.46-3.33 (m, 1H), 2.47-2.35 (m, 2H), 2.35-2.22 (m, 4H), 2.22-2.14 (m, 1H), 1.67-1.49 (m, 4H), 1.49-1.27 (m, 9H), 1.27-1.14 (m, 2H). ^{13}C NMR (101 MHz, MeOD) δ 178.1, 177.7, 77.7, 69.5, 41.8, 35.7, 34.9, 32.0, 31.1, 30.38, 30.35, 30.1, 27.2, 26.1. HRMS (ESI-MS) calculated for **33** $C_{17}H_{27}O_7^-$ $[M-H]^-$: 343.1762, found: 343.1765.

Alcohol **34**

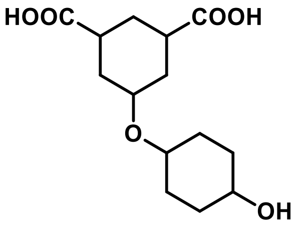

Following General Procedure C: triester **77** (0.045 g, 0.14 mmol) was reacted to afford the title compound (0.016 g, 39%) as a white amorphous solid.

^{1}H NMR (400 MHz, MeOD) δ 3.69-3.41 (m, 2H), 2.65 (p, 1H, *J* = 6.0 Hz), 2.48-2.29 (m, 1H), 2.41 (tt, 1H, *J* = 12.7, 3.5 Hz), 2.33 (tt, 1H, *J* = 11.4, 3.3 Hz), 2.30-2.15 (m, 2H), 2.03-1.84 (m, 4H), 1.84-1.53 (m, 4H), 1.50-1.14 (m, 5H). ^{13}C NMR (101 MHz, MeOD) δ = 179.1, 179.0, 178.1, 76.2, 75.4, 74.9, 70.3, 43.7, 41.9, 40.3, 36.6, 33.8, 33.6, 32.5, 32.0, 31.5, 31.2, 30.6, 29.8, 29.3, 29.0, 23.2. HRMS (ESI-MS) calculated for **34** $C_{14}H_{21}O_6^-$ [M-H]$^-$: 285.1344, found: 285.1350.

Acetyl ester **35**

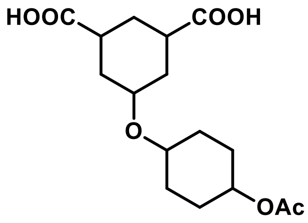

Alcohol **16** (0.002 g, 0.01 mmol) was dissolved in acetonitrile (200 µL) and acetic anhydride (200 µL) and it was left stirring at room temperature for four hours, after which the solvent was removed in vacuo, yielding a white amorphous solid which contained the title compound and was immediately analysed by LCMS2.

HRMS (ESI-MS) calculated for **35** $C_{16}H_{23}O_7^-$ [M-H]$^-$: 327.1449, found: 327.1462.

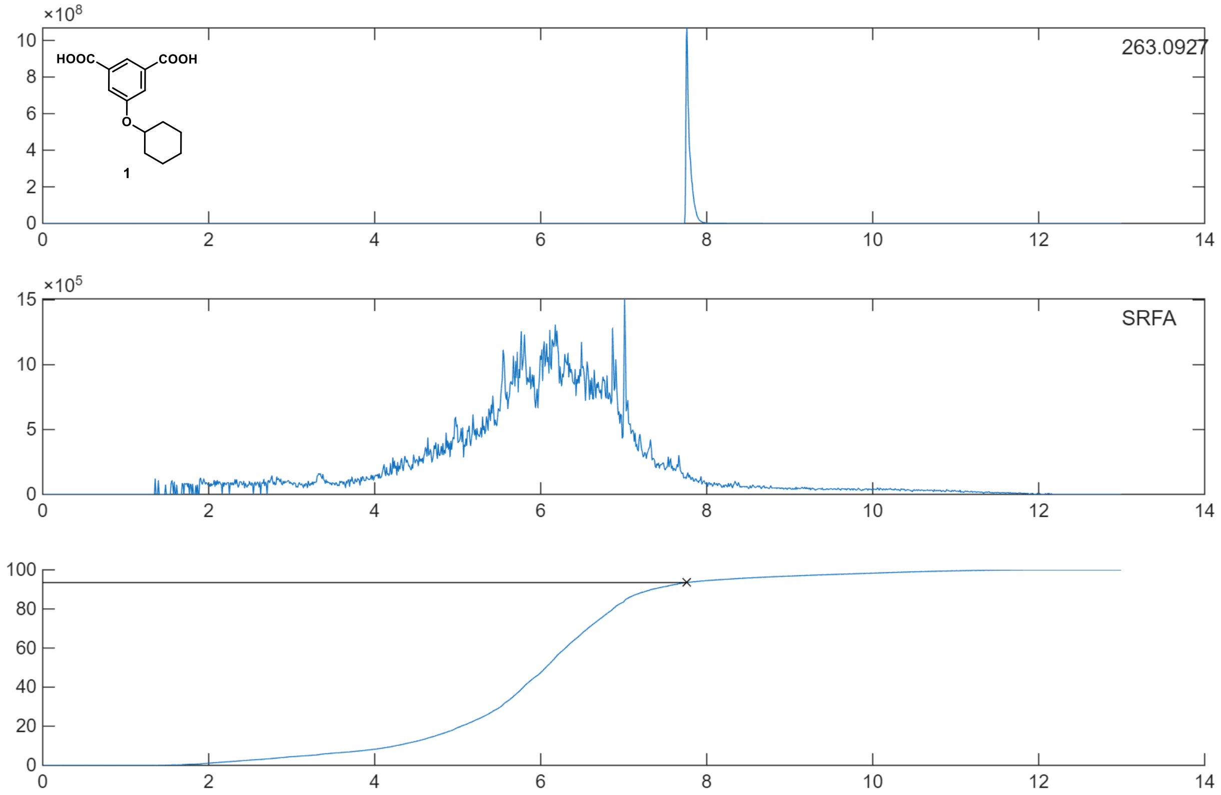

Figure S1: *Top: XIC for 263.0927 m/z for dicarboxylic acid* ***1****, Middle: XIC for 263.0927 for SRFA, Bottom: CI graph for* ***1****.*

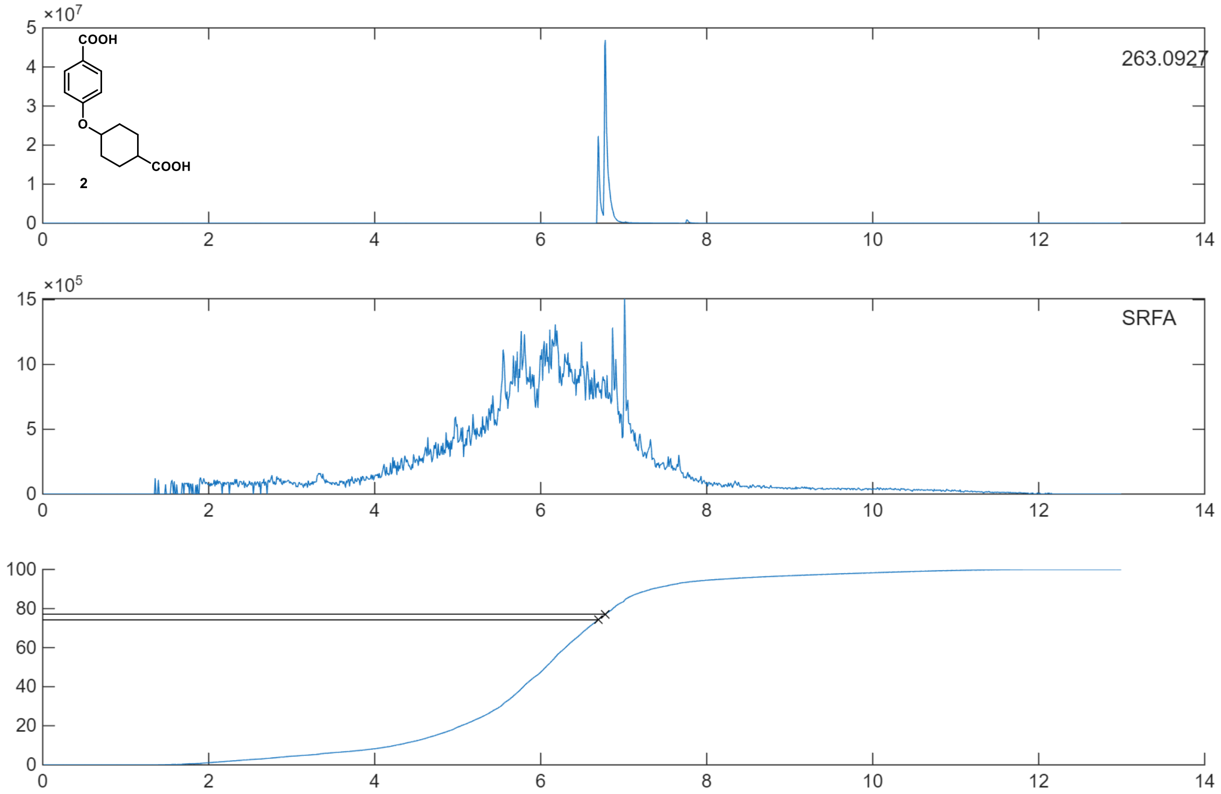

Figure S2: *Top: XIC for 263.0927 m/z for dicarboxylic acid* ***2****, Middle: XIC for 263.0927 for SRFA, Bottom: CI graph for* ***2****.*

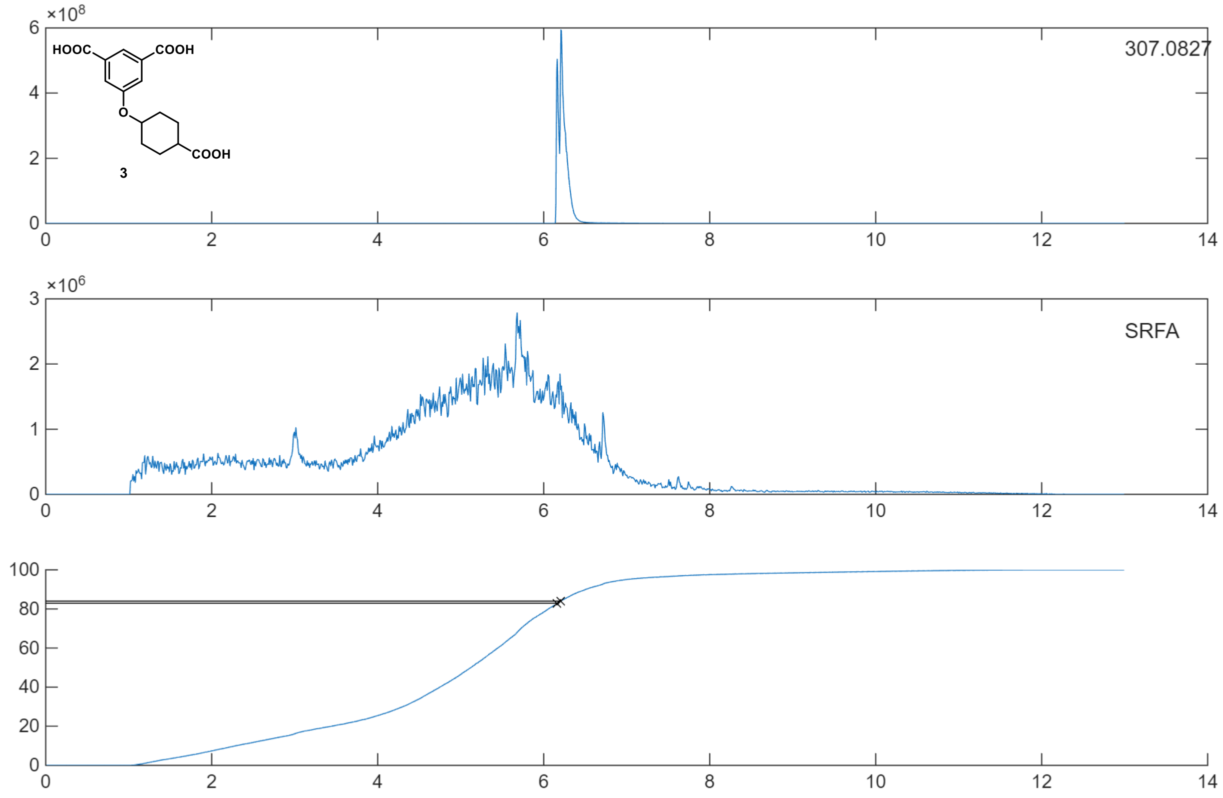

Figure S3: *Top: XIC for 307.0827 m/z for tricarboxylic acid **3**, Middle: XIC for 307.0827 for SRFA, Bottom: CI graph for **3**.*

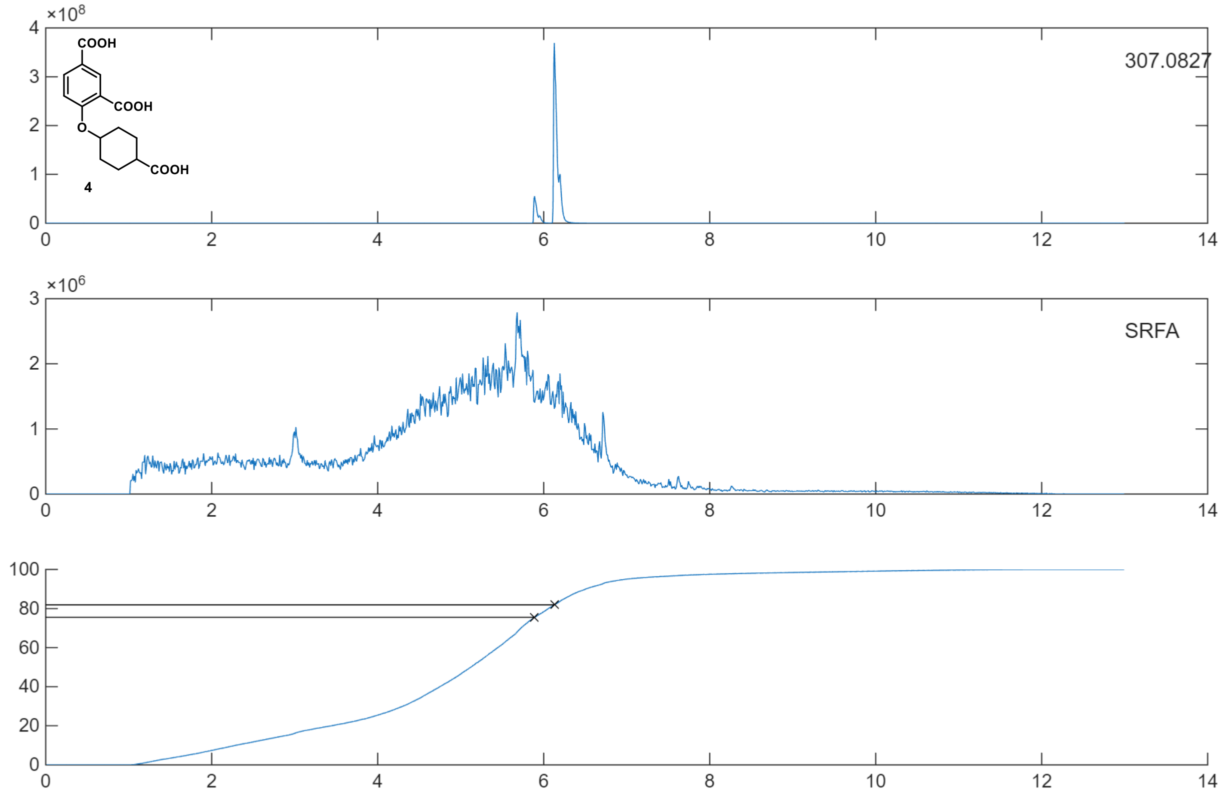

Figure S4: *Top: XIC for 307.0827 m/z for tricarboxylic acid **4**, Middle: XIC for 307.0827 for SRFA, Bottom: CI graph for **4**.*

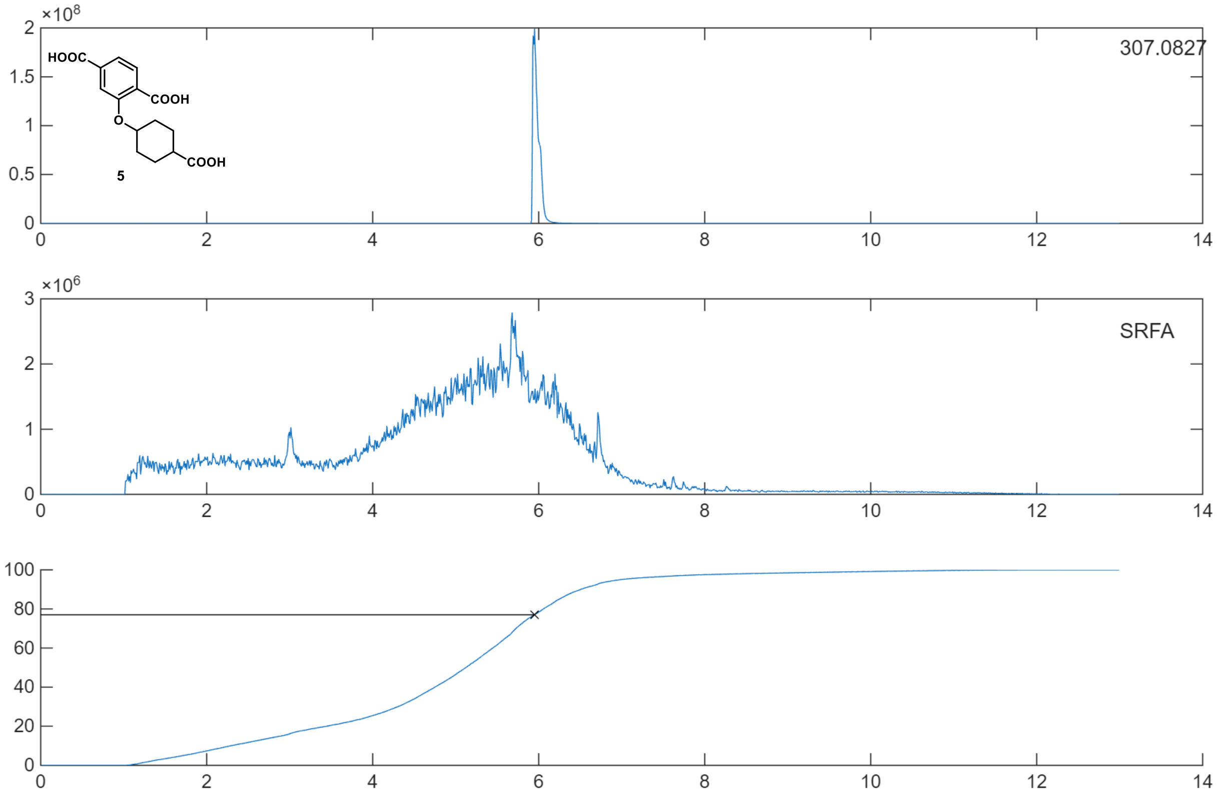

Figure S5: *Top: XIC for 307.0827 m/z for tricarboxylic acid* ***5****, Middle: XIC for 307.0827 for SRFA, Bottom: CI graph for* ***5****.*

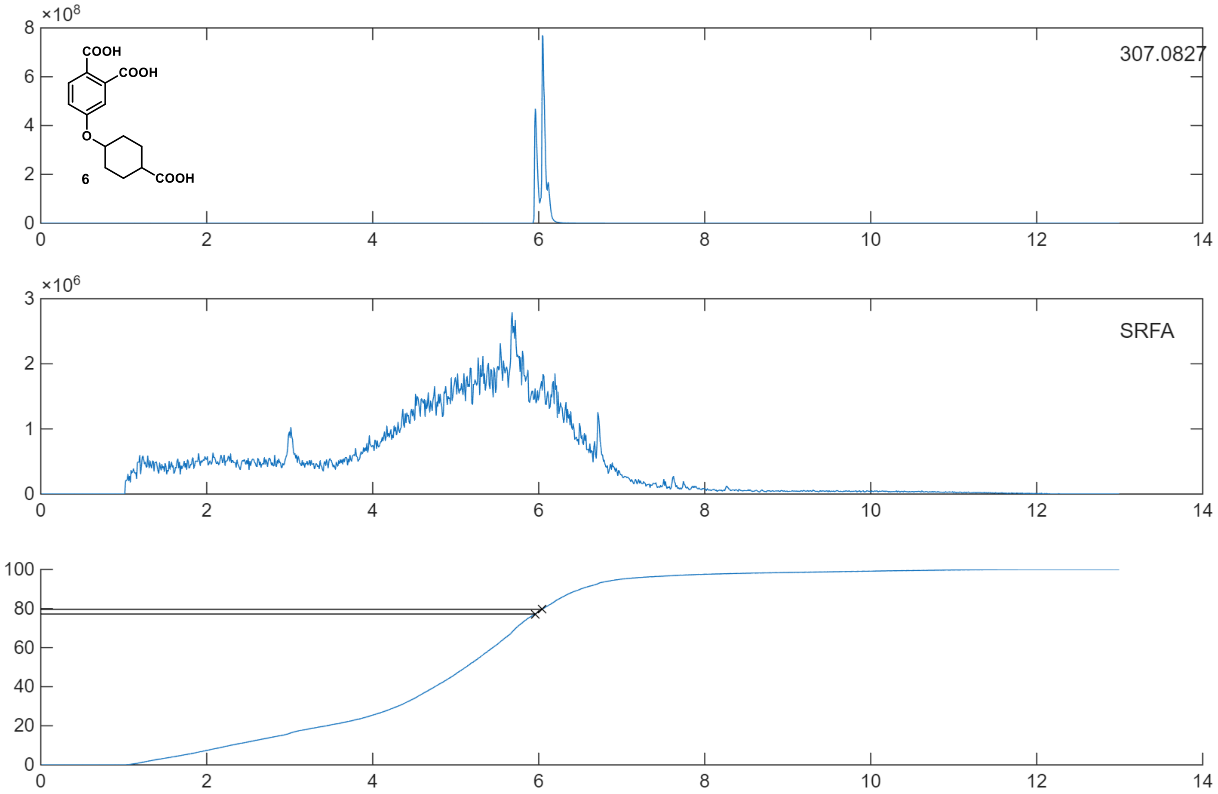

Figure S6: *Top: XIC for 307.0827 m/z for tricarboxylic acid* ***6****, Middle: XIC for 307.0827 for SRFA, Bottom: CI graph for* ***6****.*

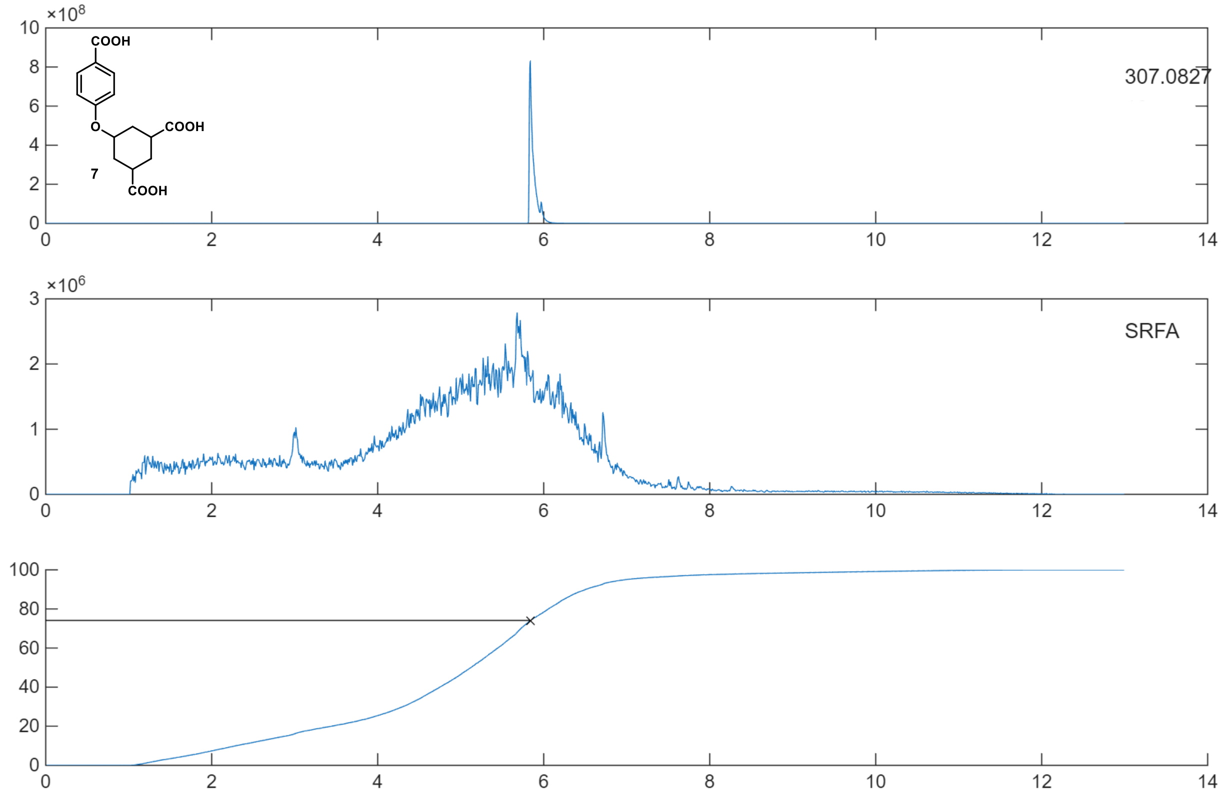

Figure S7: *Top: XIC for 307.0827 m/z for tricarboxylic acid **7**, Middle: XIC for 307.0827 for SRFA, Bottom: CI graph for **7**.*

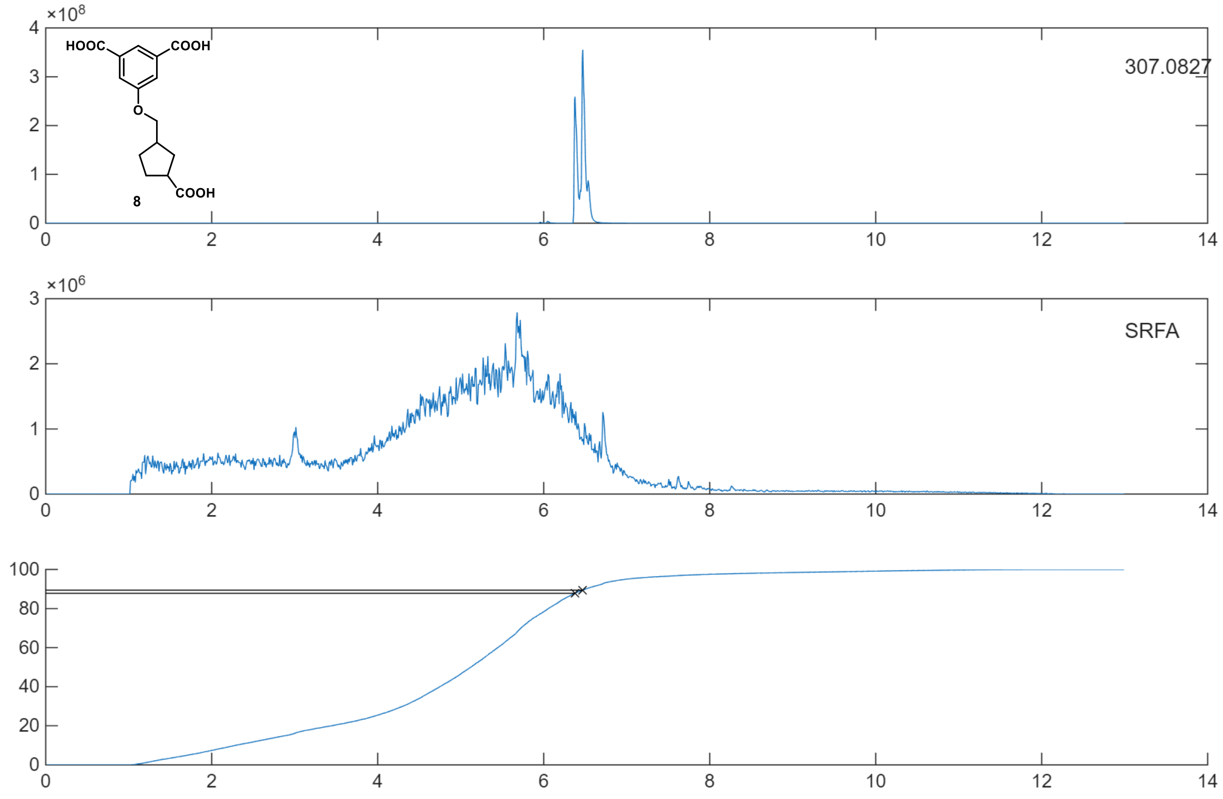

Figure S8: *Top: XIC for 307.0827 m/z for tricarboxylic acid **8**, Middle: XIC for 307.0827 for SRFA, Bottom: CI graph for **8**.*

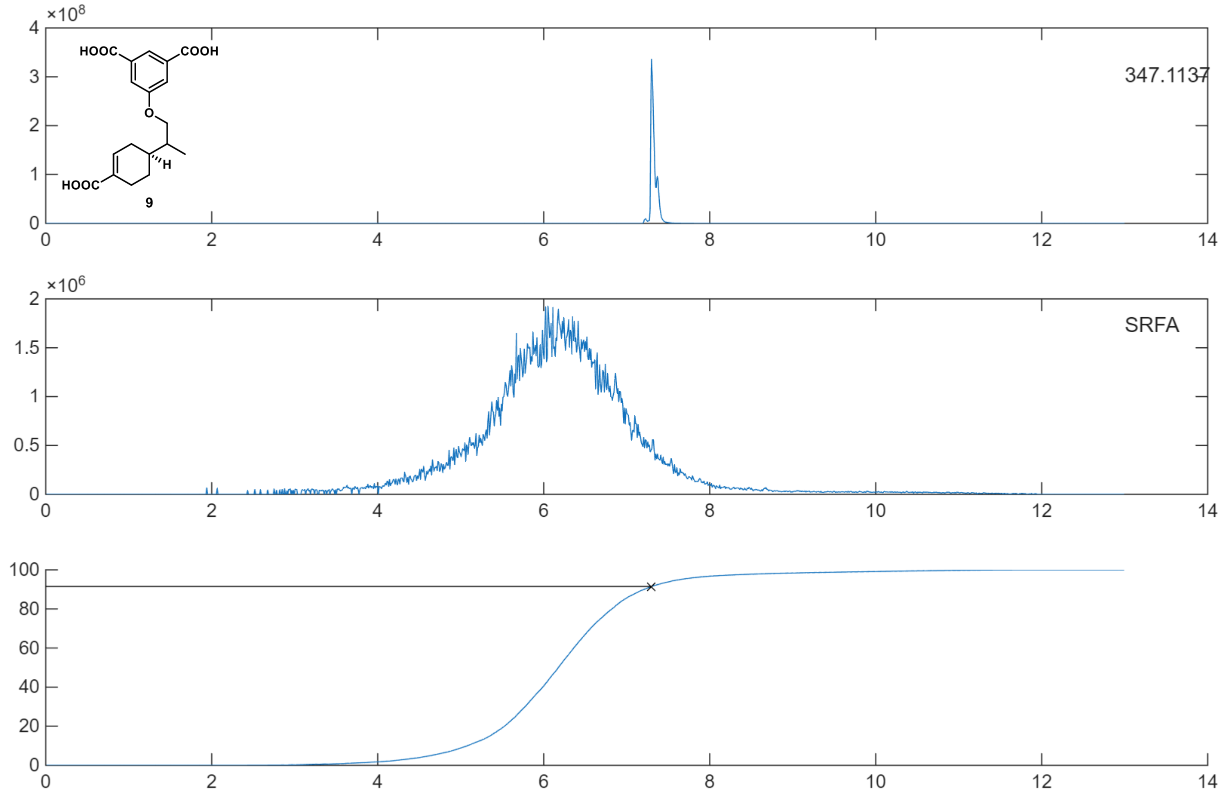

Figure S9: *Top: XIC for 347.1137 m/z for tricarboxylic acid **9**, Middle: XIC for 347.1137 for SRFA, Bottom: CI graph for **9**.*

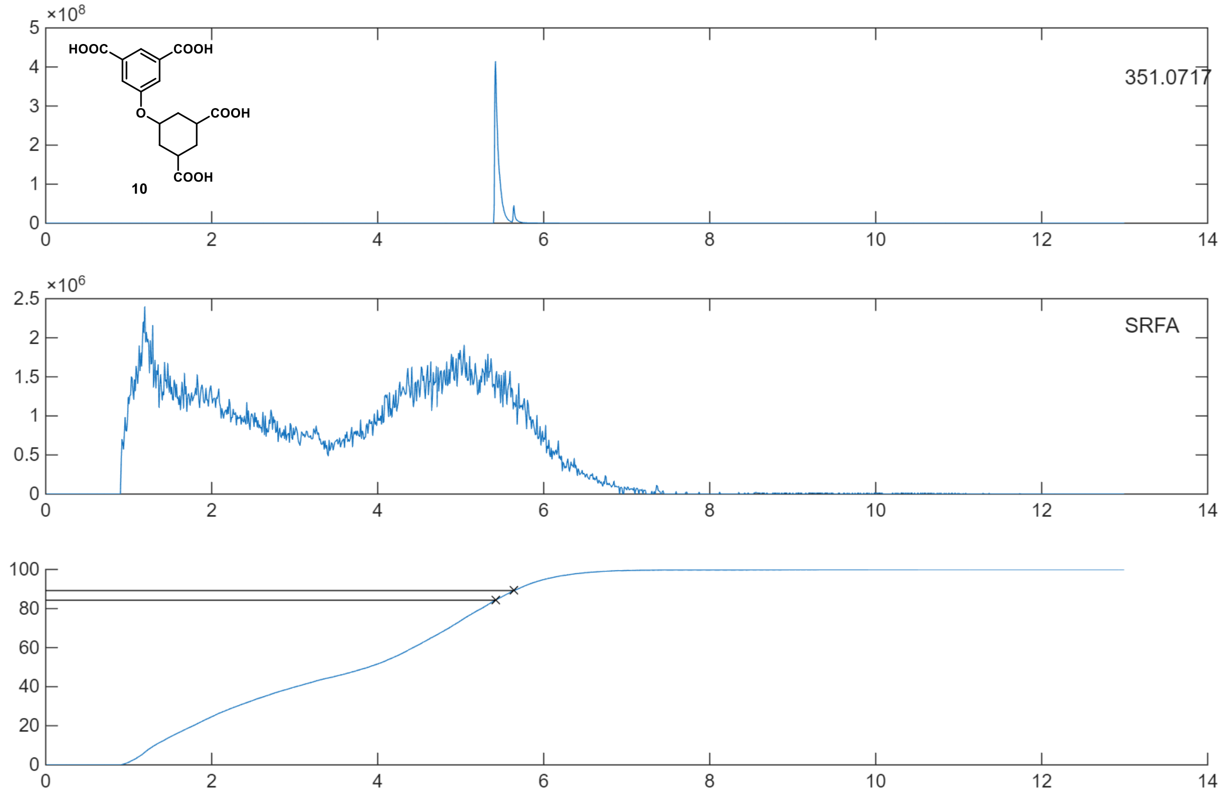

Figure S10: *Top: XIC for 351.0717 m/z for tetracarboxylic acid **10**, Middle: XIC for 351.0717 for SRFA, Bottom: CI graph for **10**.*

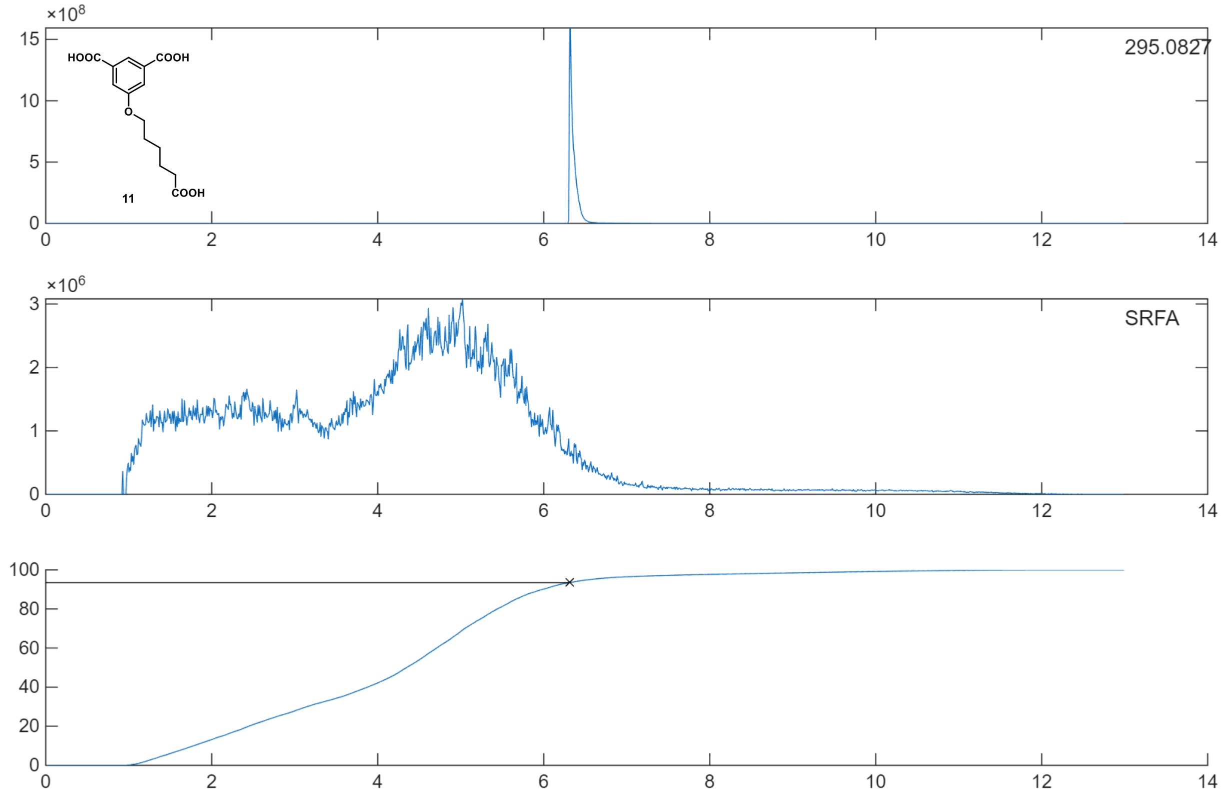

Figure S11: *Top: XIC for 295.0827 m/z for tricarboxylic acid* ***11****, Middle: XIC for 295.0827 for SRFA, Bottom: CI graph for* ***11****.*

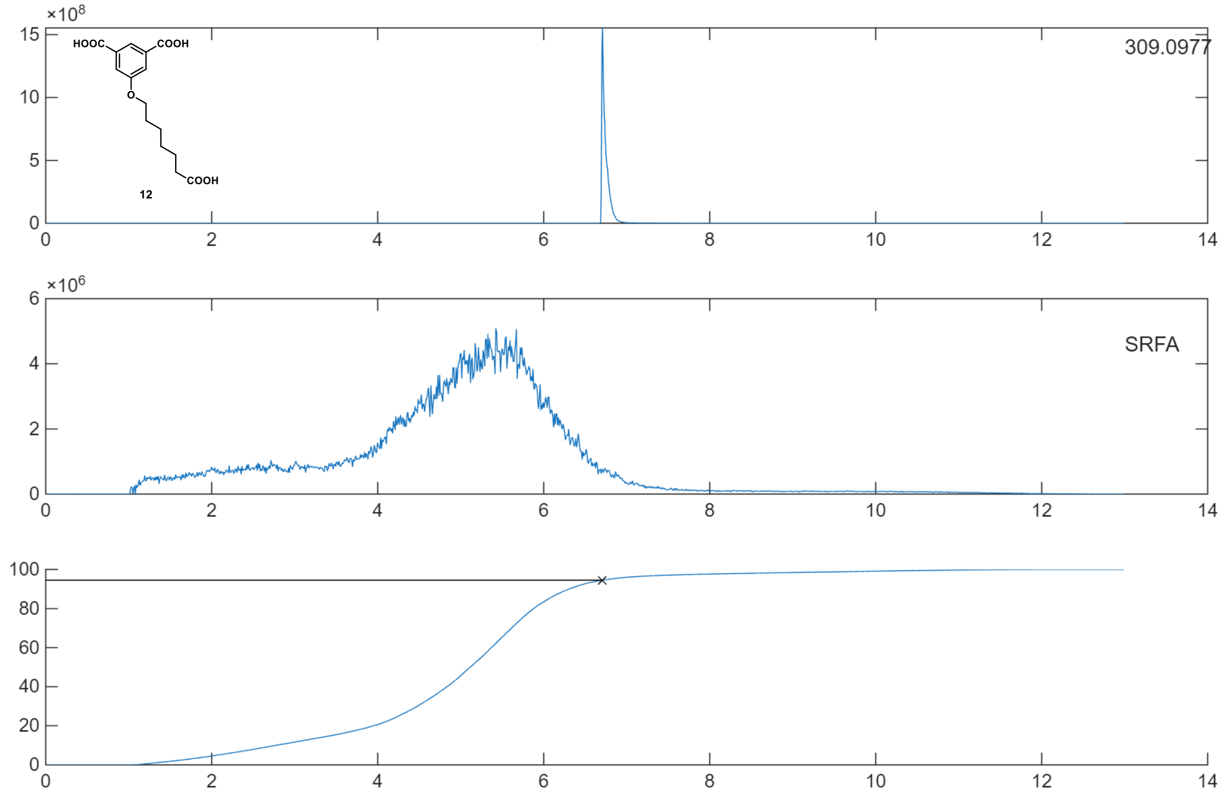

Figure S12: *Top: XIC for 309.0977 m/z for tricarboxylic acid* ***13****, Middle: XIC for 309.0977 for SRFA, Bottom: CI graph for* ***12****.*

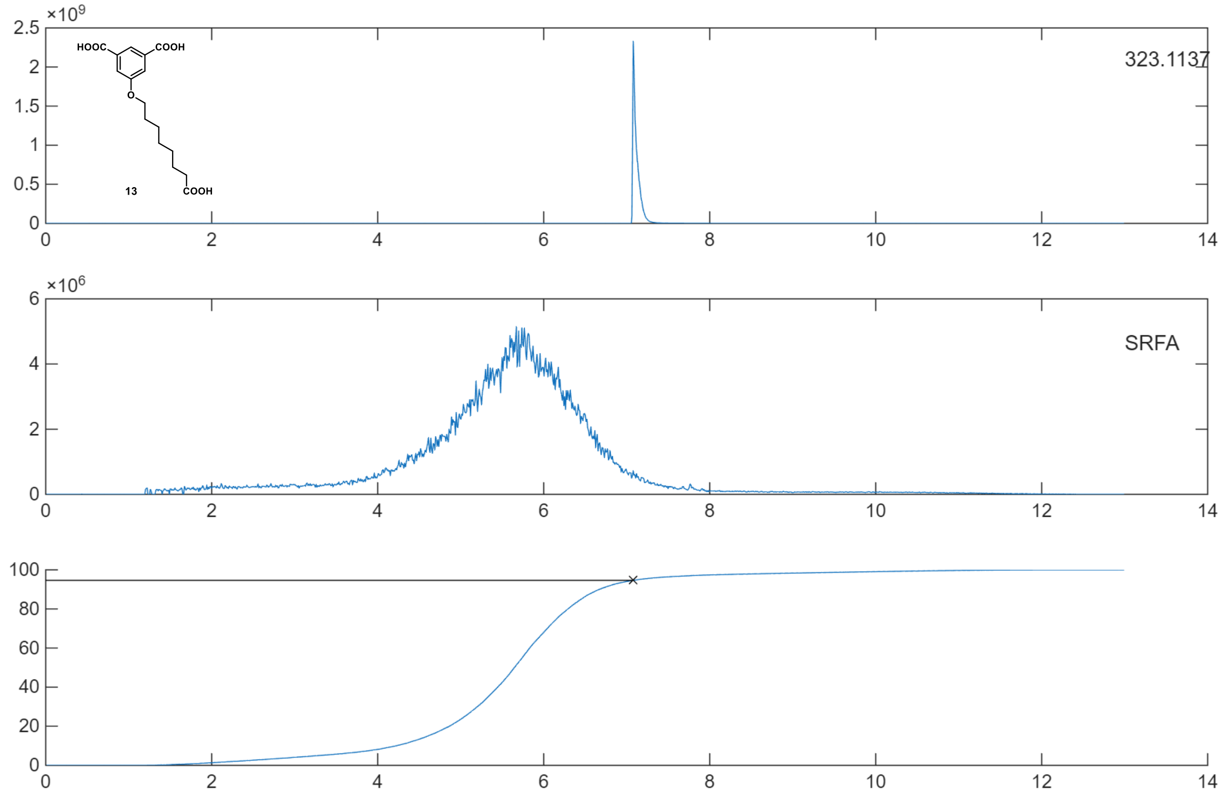

Figure S13: *Top: XIC for 323.1137 m/z for tricarboxylic acid* ***13****, Middle: XIC for 323.1137 for SRFA, Bottom: CI graph for* ***13****.*

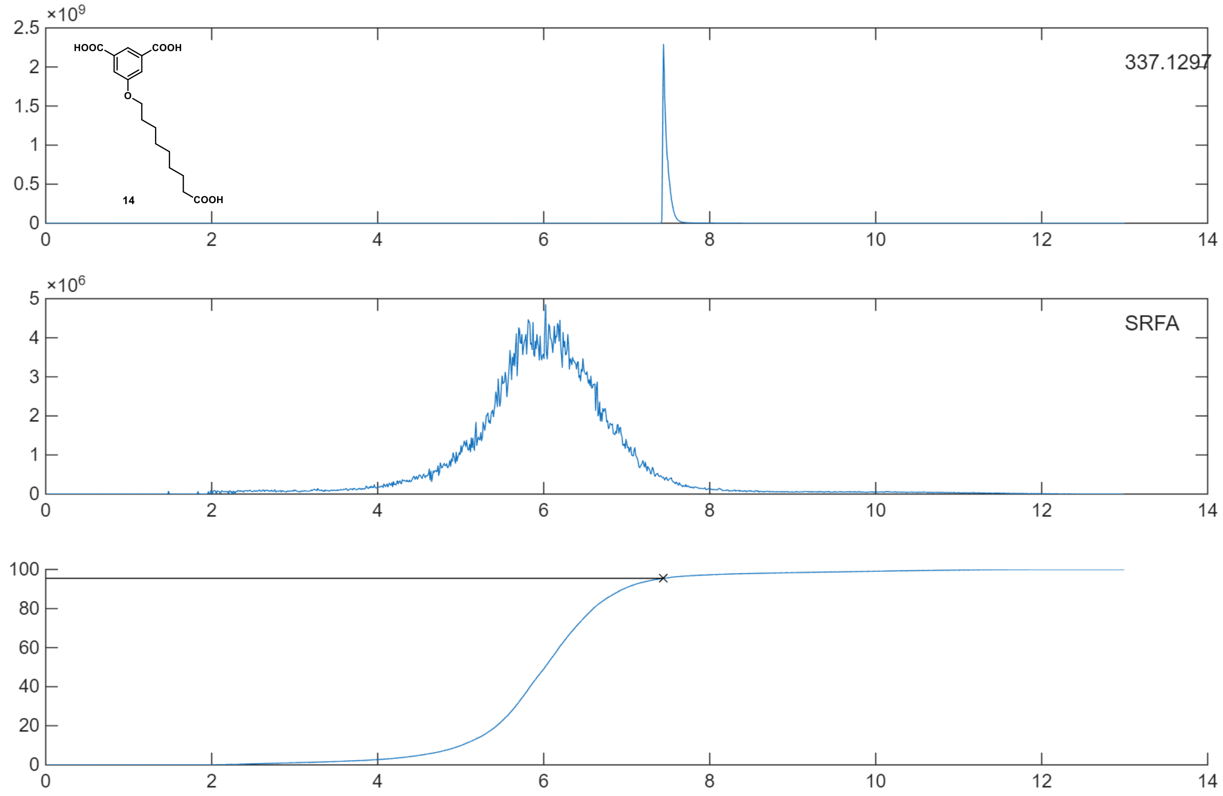

Figure S14: *Top: XIC for 337.1297 m/z for tricarboxylic acid* ***14****, Middle: XIC for 337.1297 for SRFA, Bottom: CI graph for* ***14.***

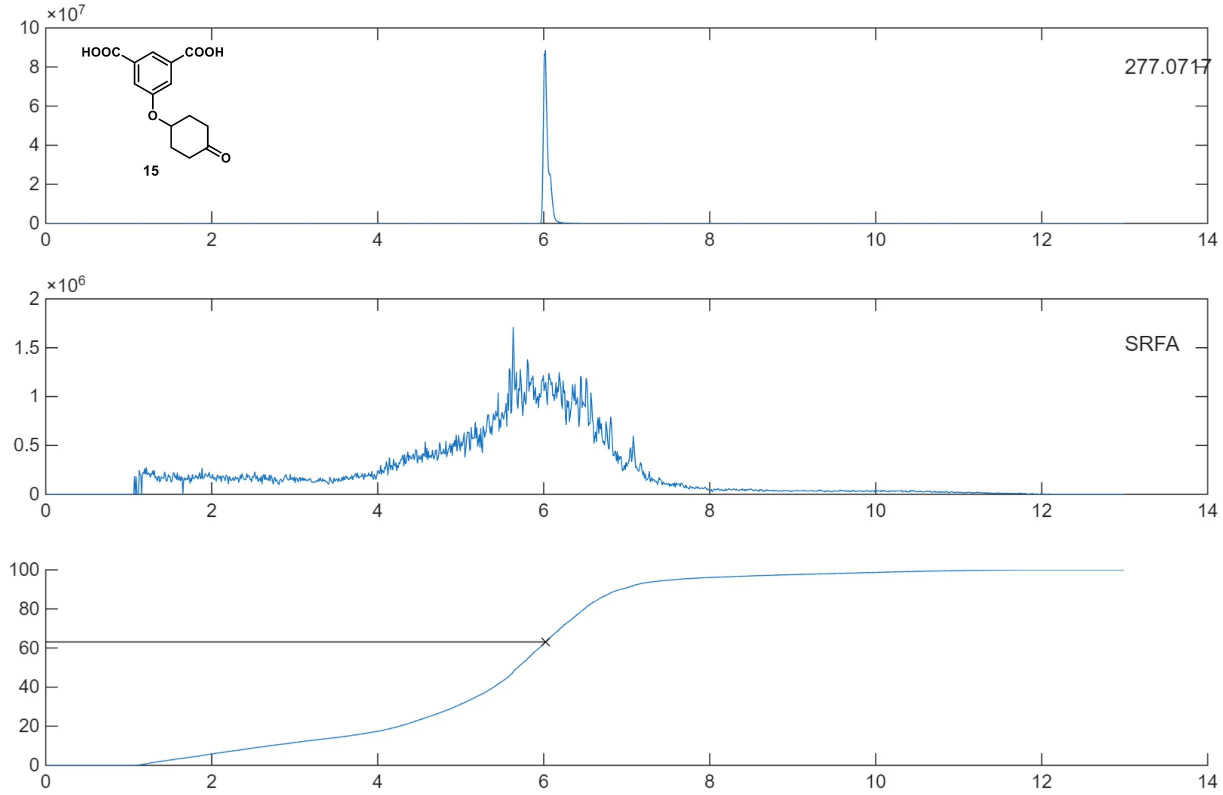

Figure S15: *Top: XIC for 277.0717 m/z for ketone* ***15****, Middle: XIC for 277.0717 for SRFA, Bottom: CI graph for* ***15****.*

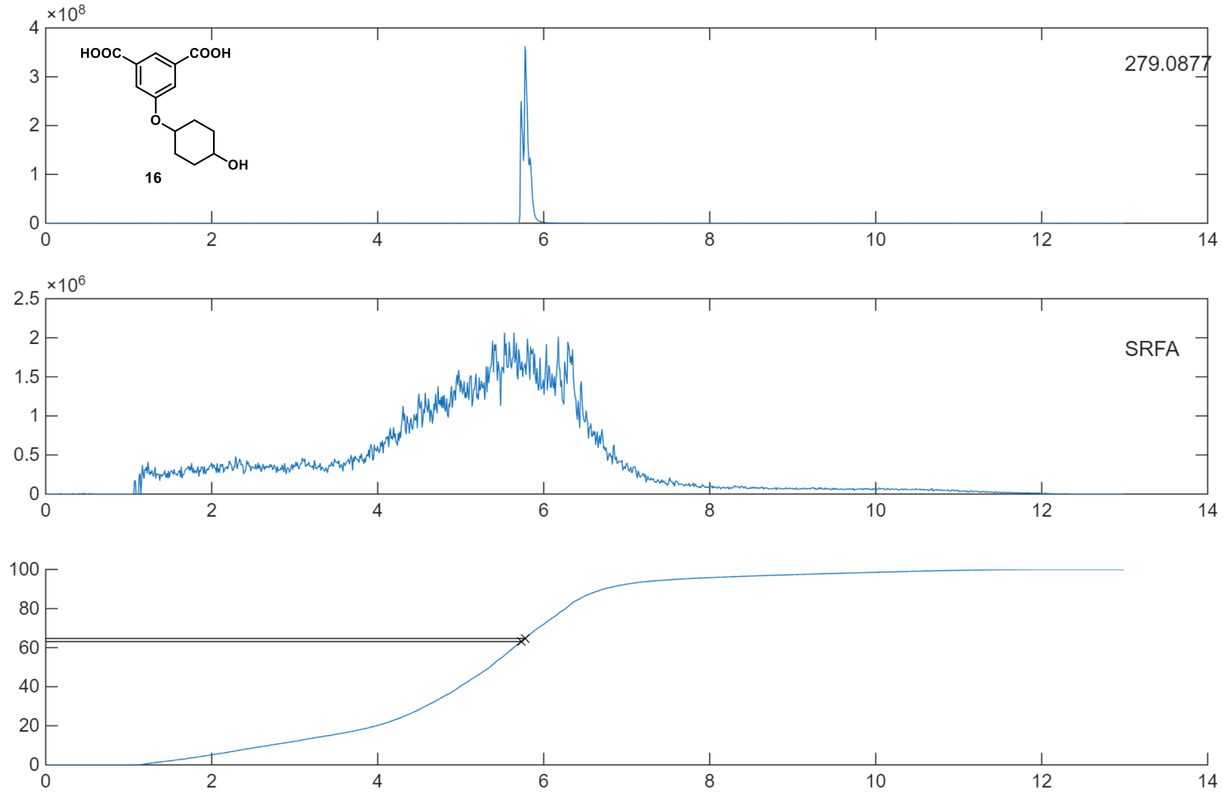

Figure S16: *Top: XIC for 279.0877 m/z for alcohol* ***16****, Middle: XIC for 279.0877 for SRFA, Bottom: CI graph for* ***16****.*

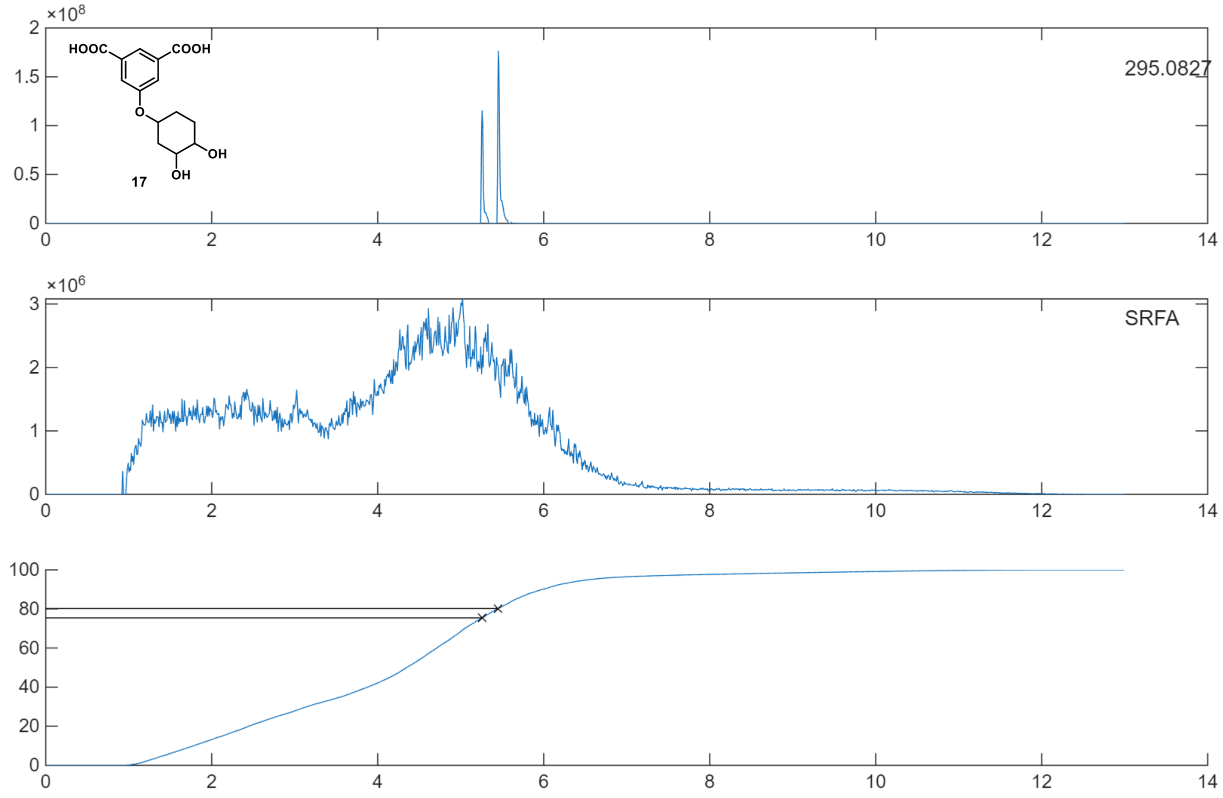

Figure S17: *Top: XIC for 295.0827 m/z for diol **17**, Middle: XIC for 295.0827 for SRFA, Bottom: CI graph for **17**.*

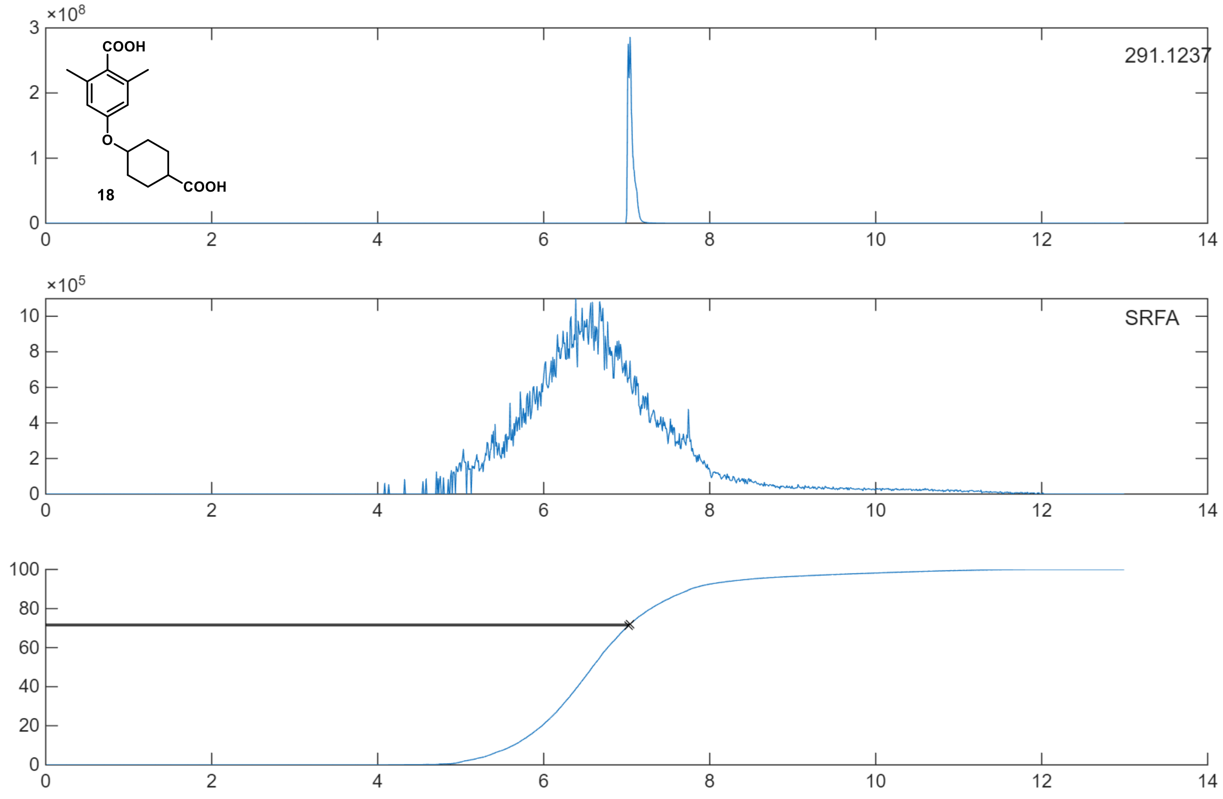

Figure S18: *Top: XIC for 291.1237 m/z for dicarboxylic acid **18**, Middle: XIC for 291.1237 for SRFA, Bottom: CI graph for **18**.*

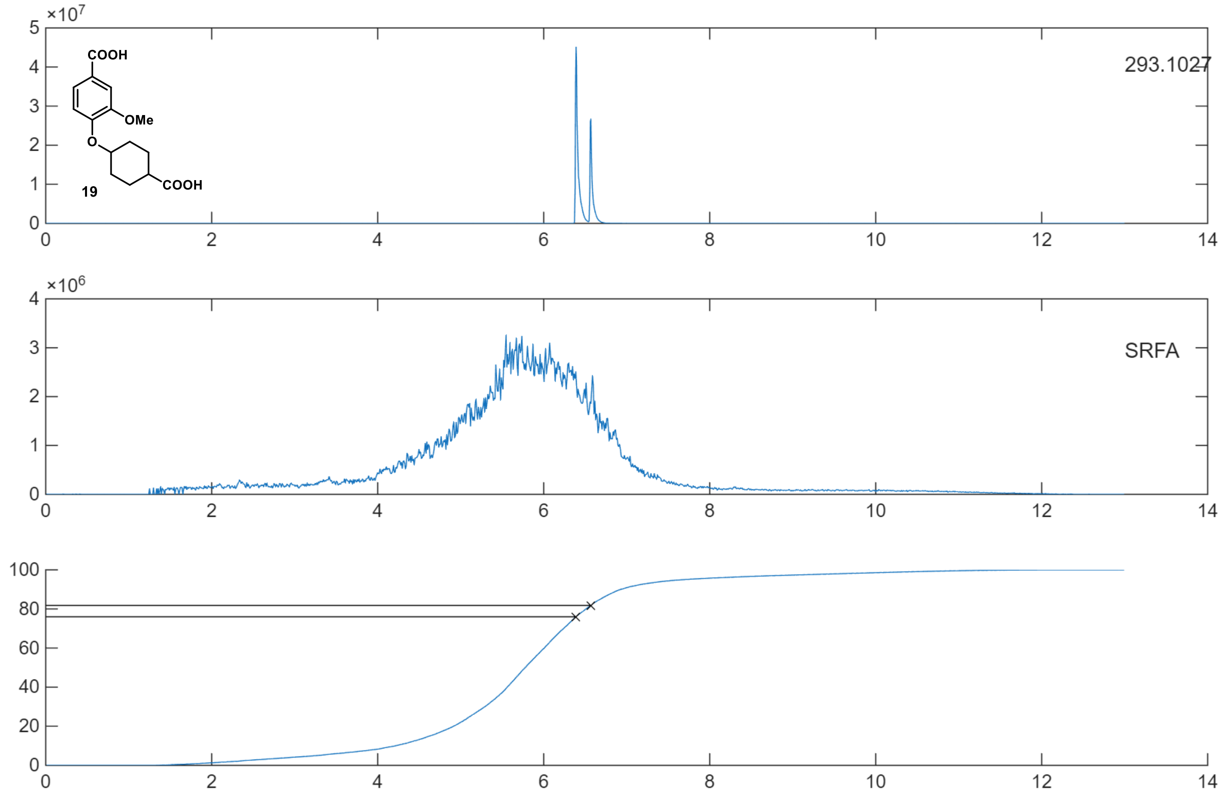

Figure S19: *Top: XIC for 293.1027 m/z for methoxy ether* ***19****, Middle: XIC for 293.1027 for SRFA, Bottom: CI graph for* ***19****.*

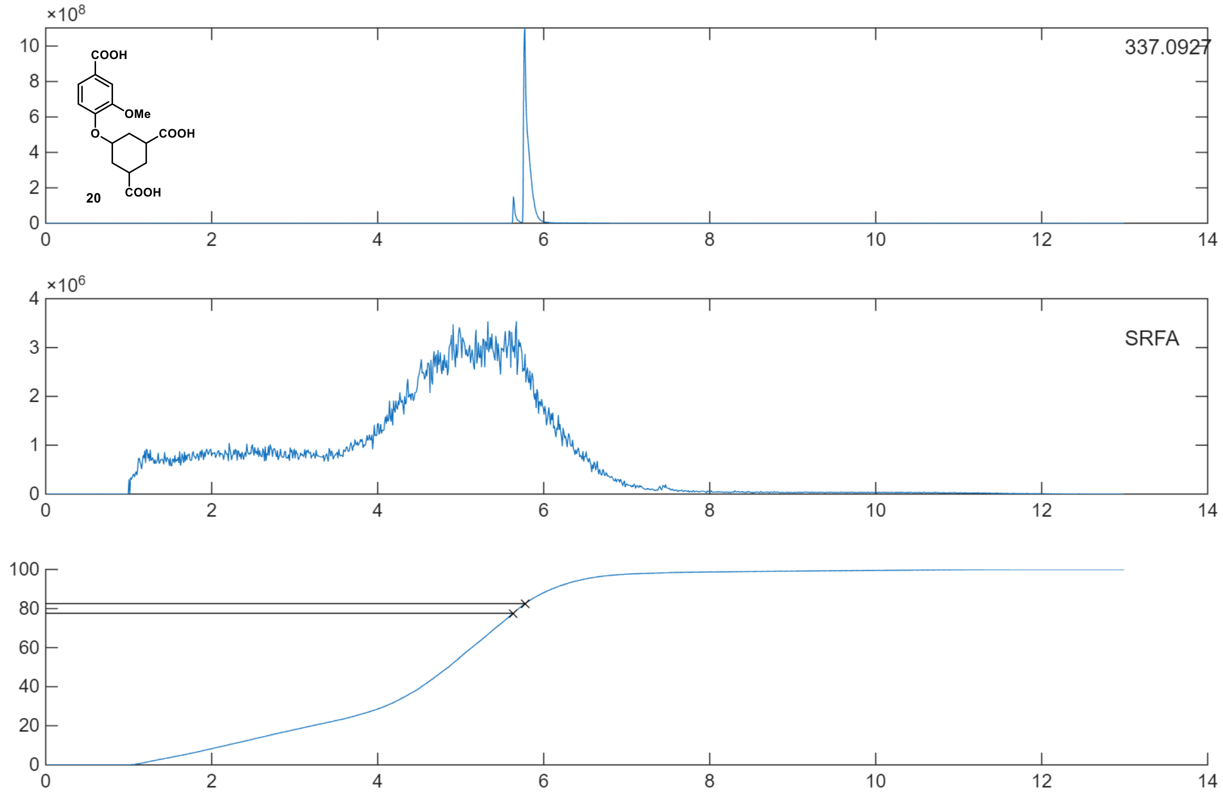

Figure S20: *Top: XIC for 337.0927 m/z for methoxy ether* ***20****, Middle: XIC for 337.0927 for SRFA, Bottom: CI graph for* ***20****.*

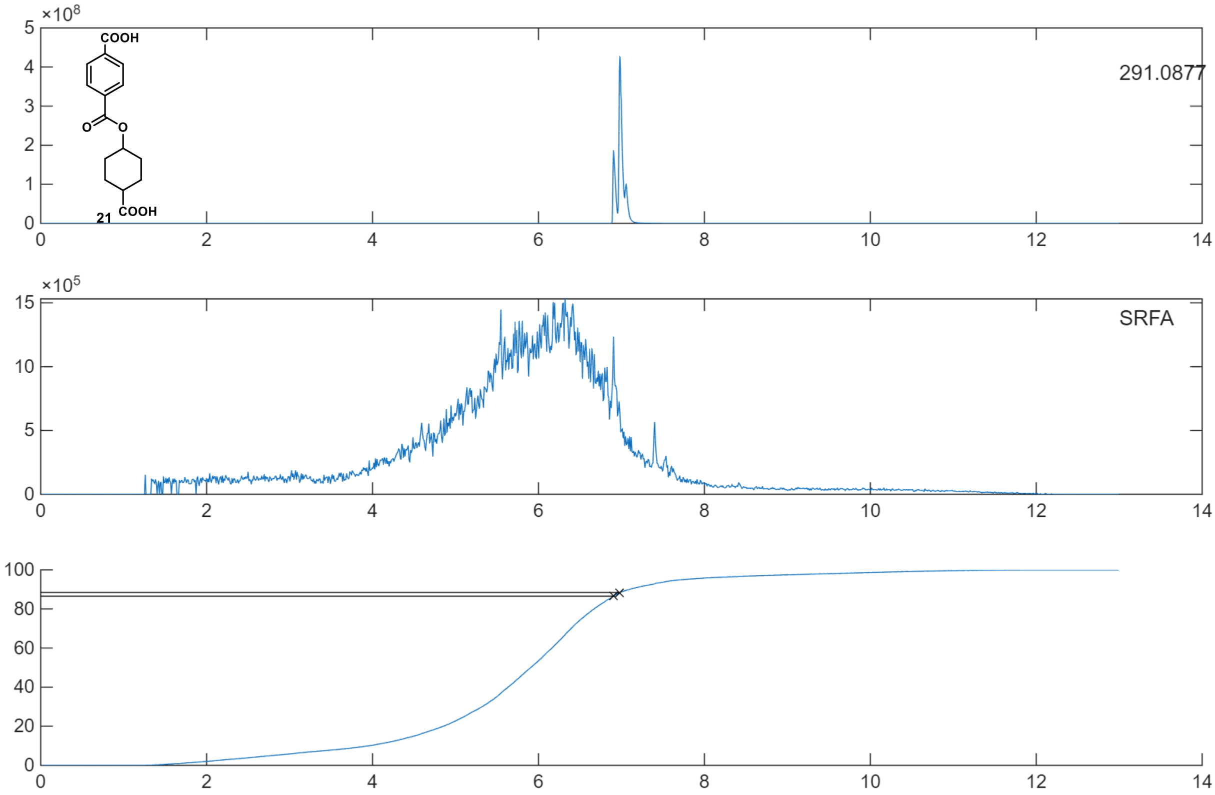

Figure S21: *Top: XIC for 291.0877 m/z for dicarboxylic acid* ***21****, Middle: XIC for 291.0877 for SRFA, Bottom: CI graph for* ***21****.*

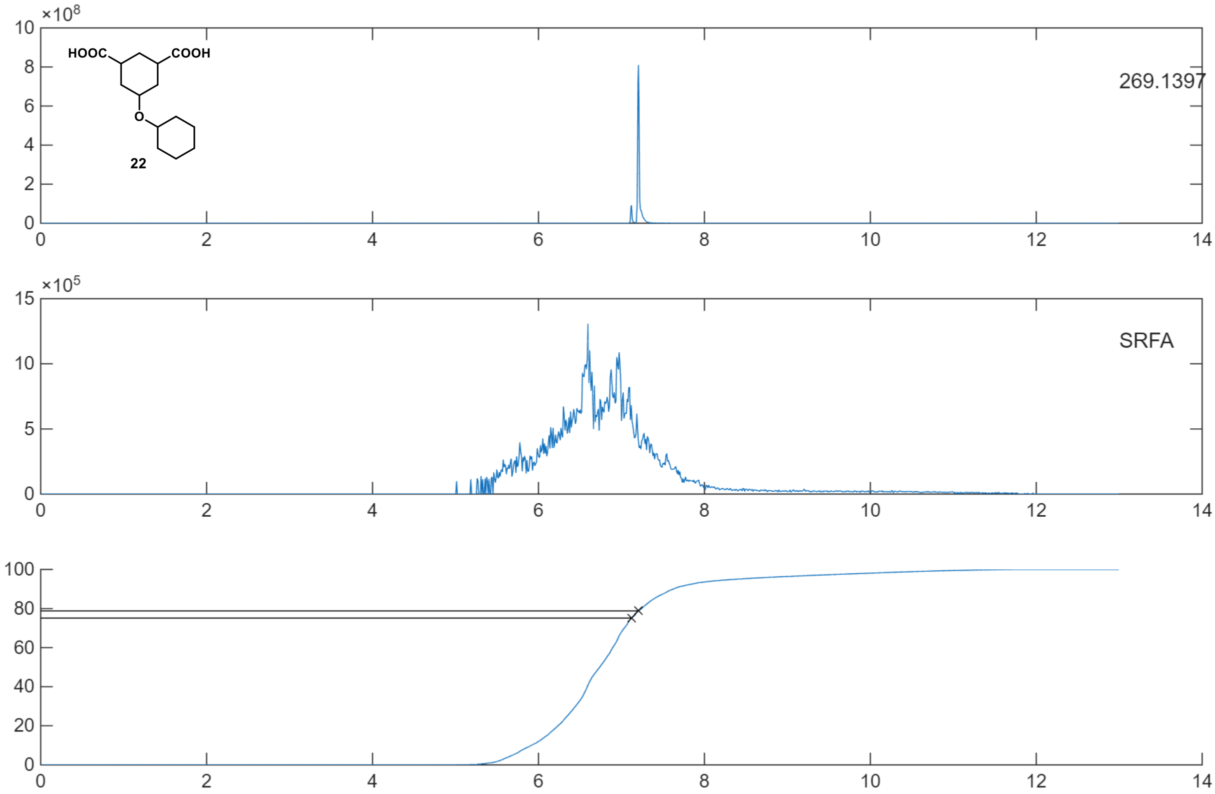

Figure S22: *Top: XIC for 269.1397 m/z for dicarboxylic acid* ***22****, Middle: XIC for 269.1397 for SRFA, Bottom: CI graph for* ***22***

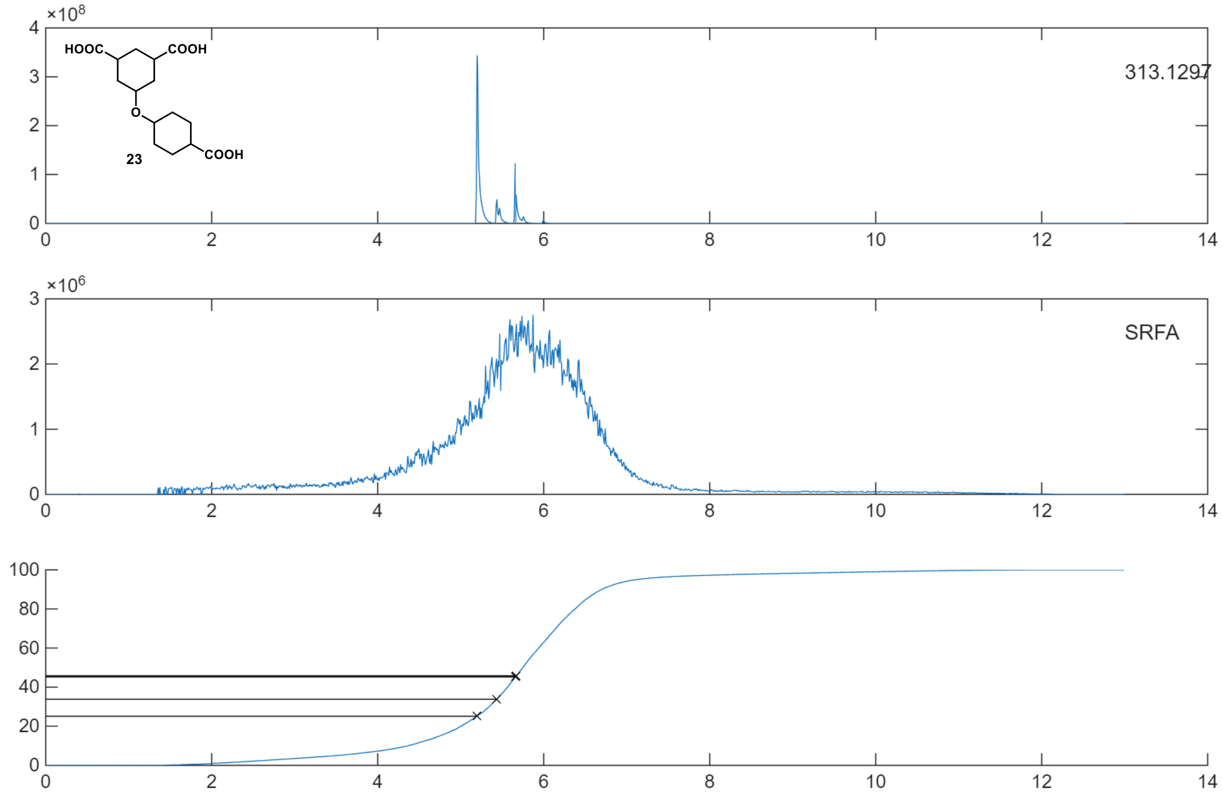

Figure S23: *Top: XIC for 313.1297 m/z for tricarboxylic acid* ***23****, Middle: XIC for 313.1297 for SRFA, Bottom: CI graph for* ***23.***

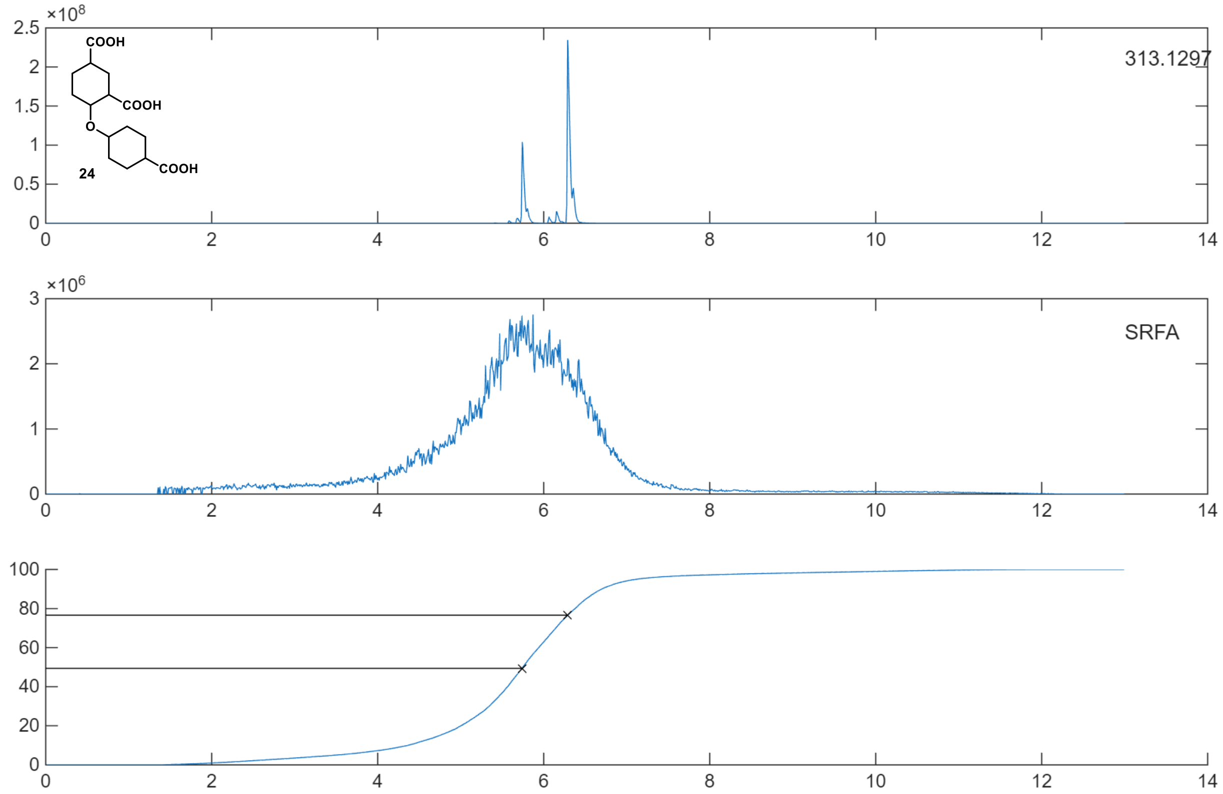

Figure S24: *Top: XIC for 313.1297 m/z for tricarboxylic acid* ***24****, Middle: XIC for 313.1297 for SRFA, Bottom: CI graph for* ***24.***

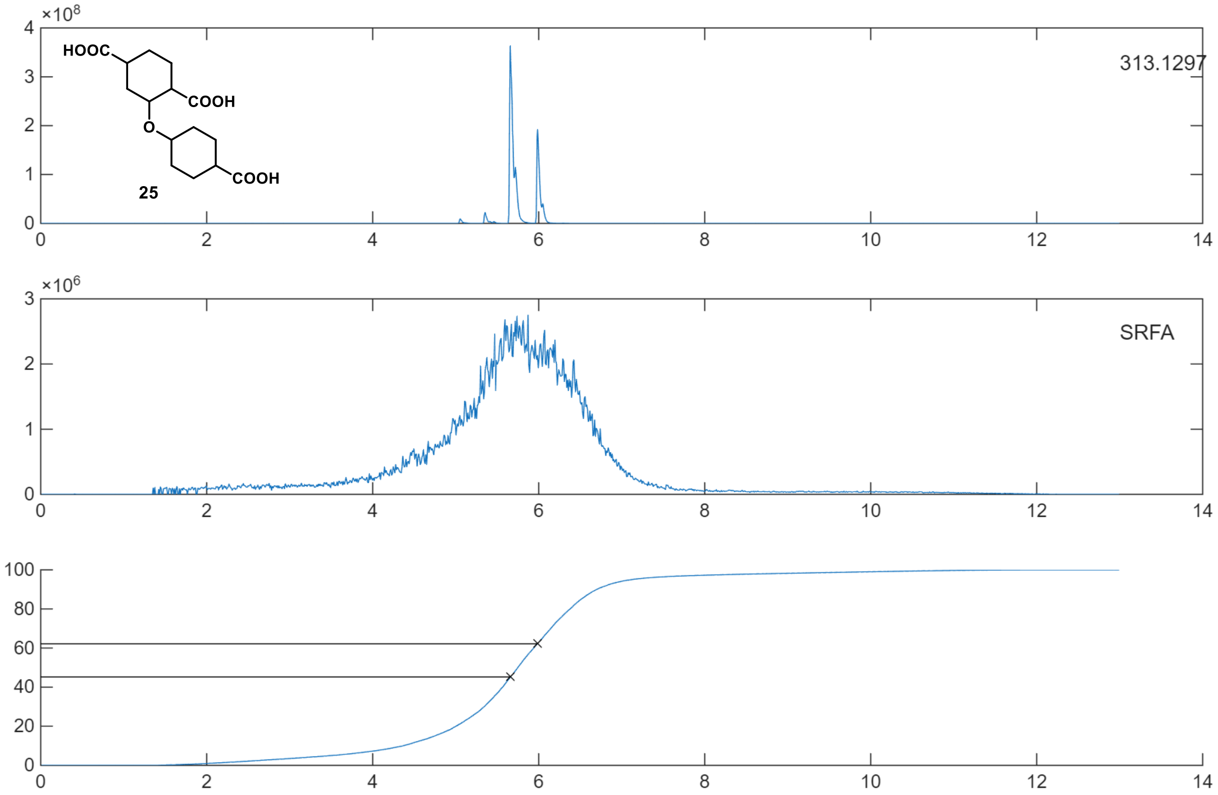

Figure S25: *Top: XIC for 313.1297 m/z for tricarboxylic acid* ***25****, Middle: XIC for 313.1297 for SRFA, Bottom: CI graph for* ***25.***

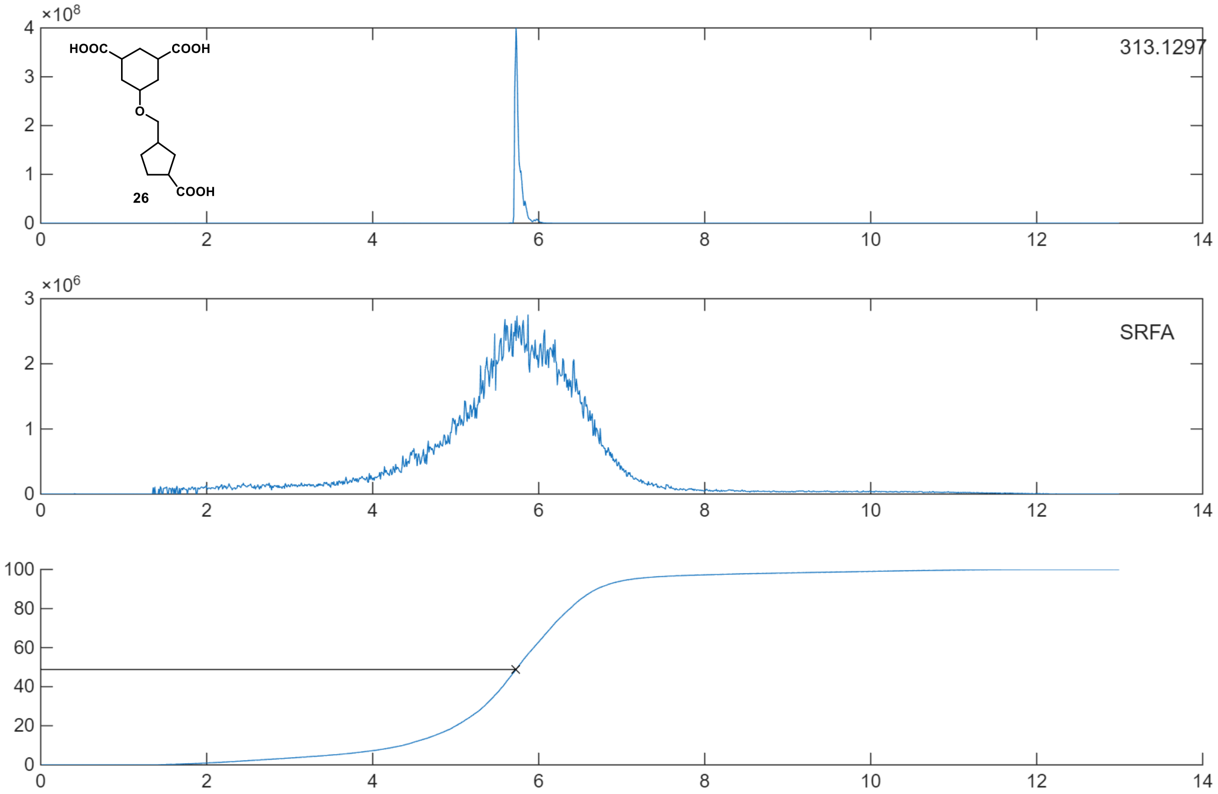

Figure S26: *Top: XIC for 313.1297 m/z for tricarboxylic acid* ***26****, Middle: XIC for 313.1297 for SRFA, Bottom: CI graph for* ***26.***

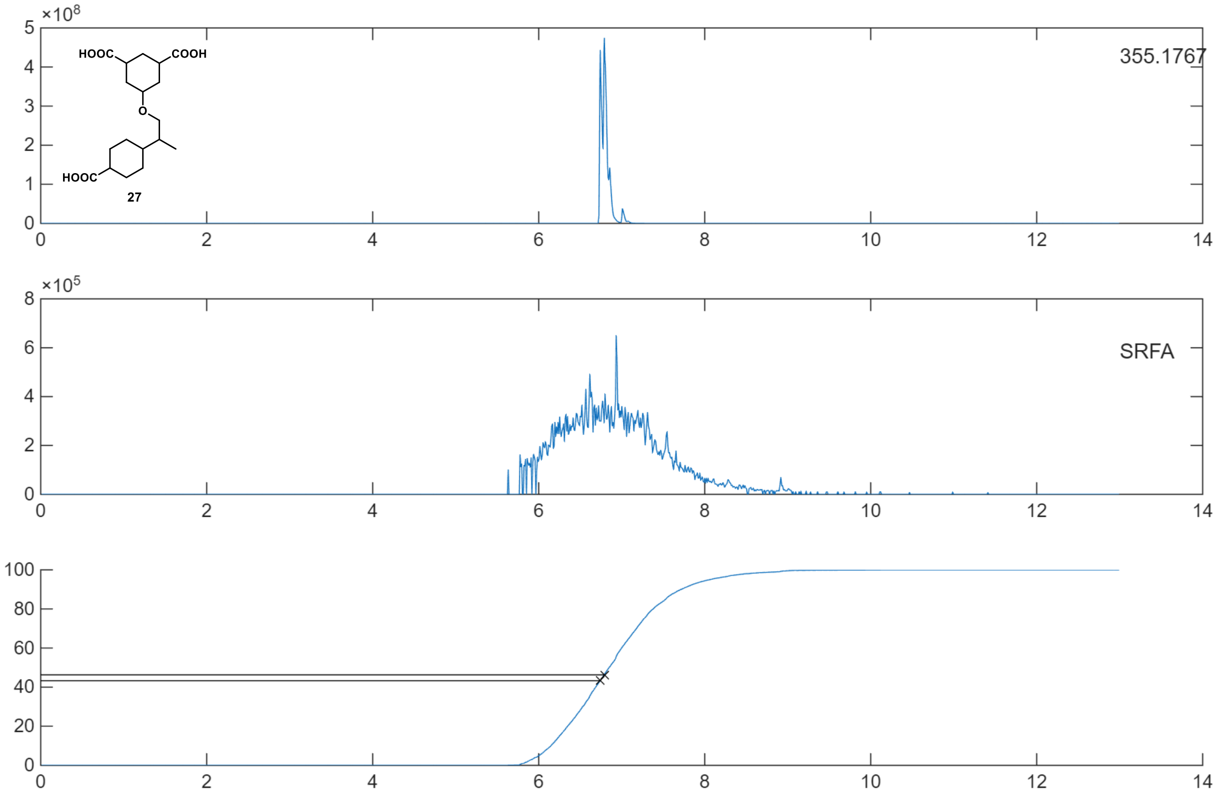

Figure S27: *Top: XIC for 355.1767 m/z for tricarboxylic acid **27**, Middle: XIC for 355.1767 for SRFA, Bottom: CI graph for **27.***

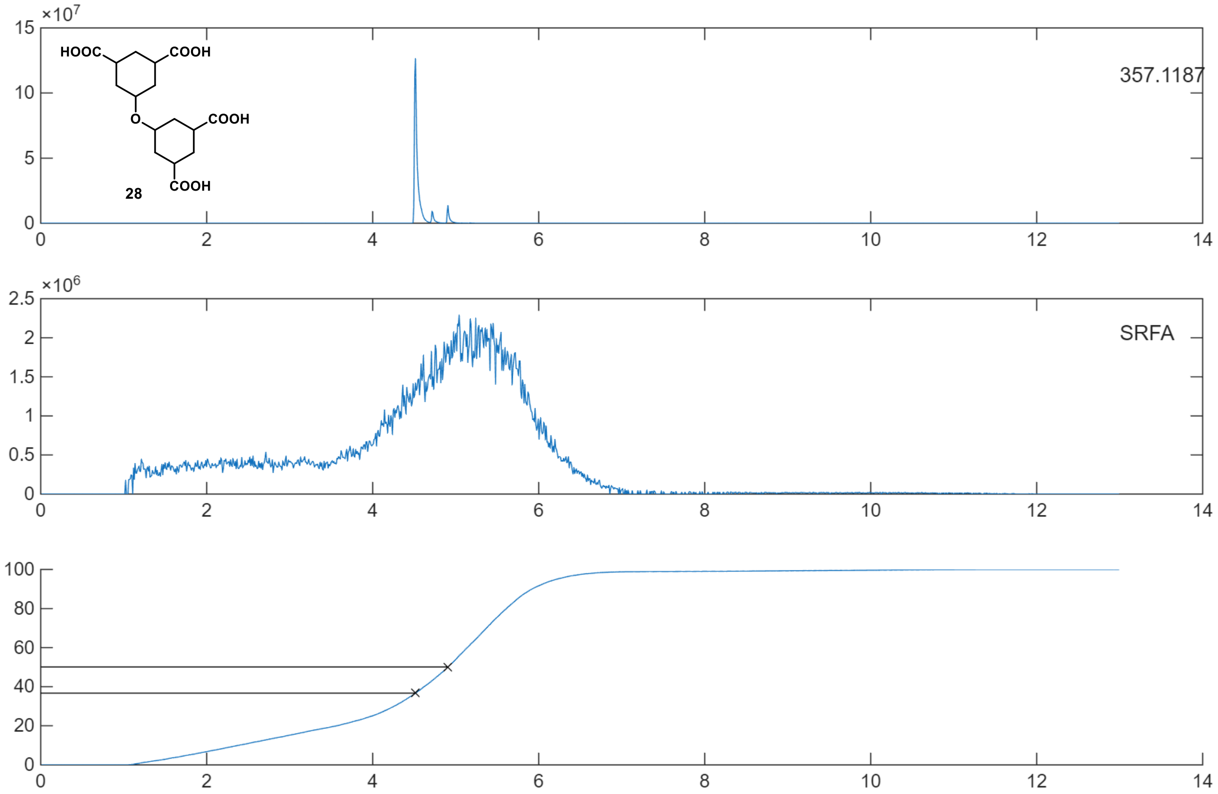

Figure S28: *Top: XIC for 357.1187 m/z for tetracarboxylic acid **28**, Middle: XIC for 357.1187 for SRFA, Bottom: CI graph for **28.***

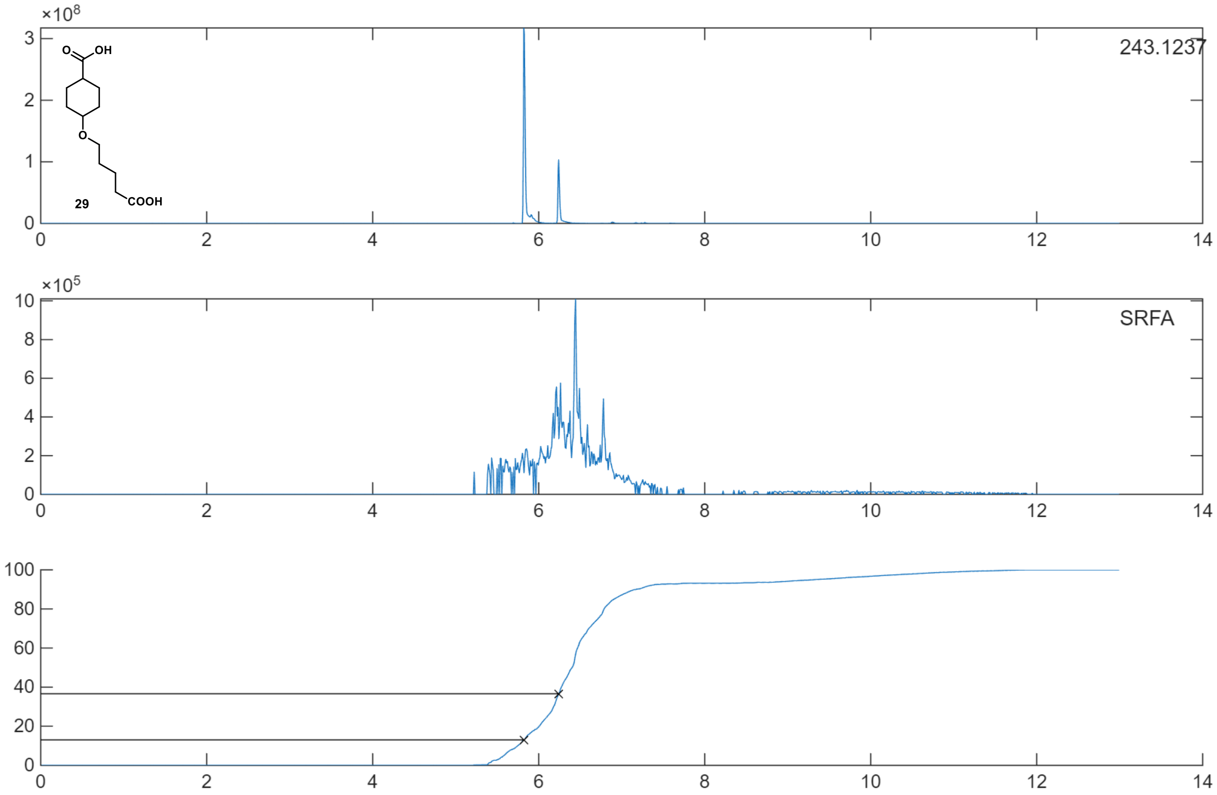

Figure S29: *Top: XIC for 243.1237 m/z for dicarboxylic acid* ***29****, Middle: XIC for 243.1237 for SRFA, Bottom: CI graph for* ***29.***

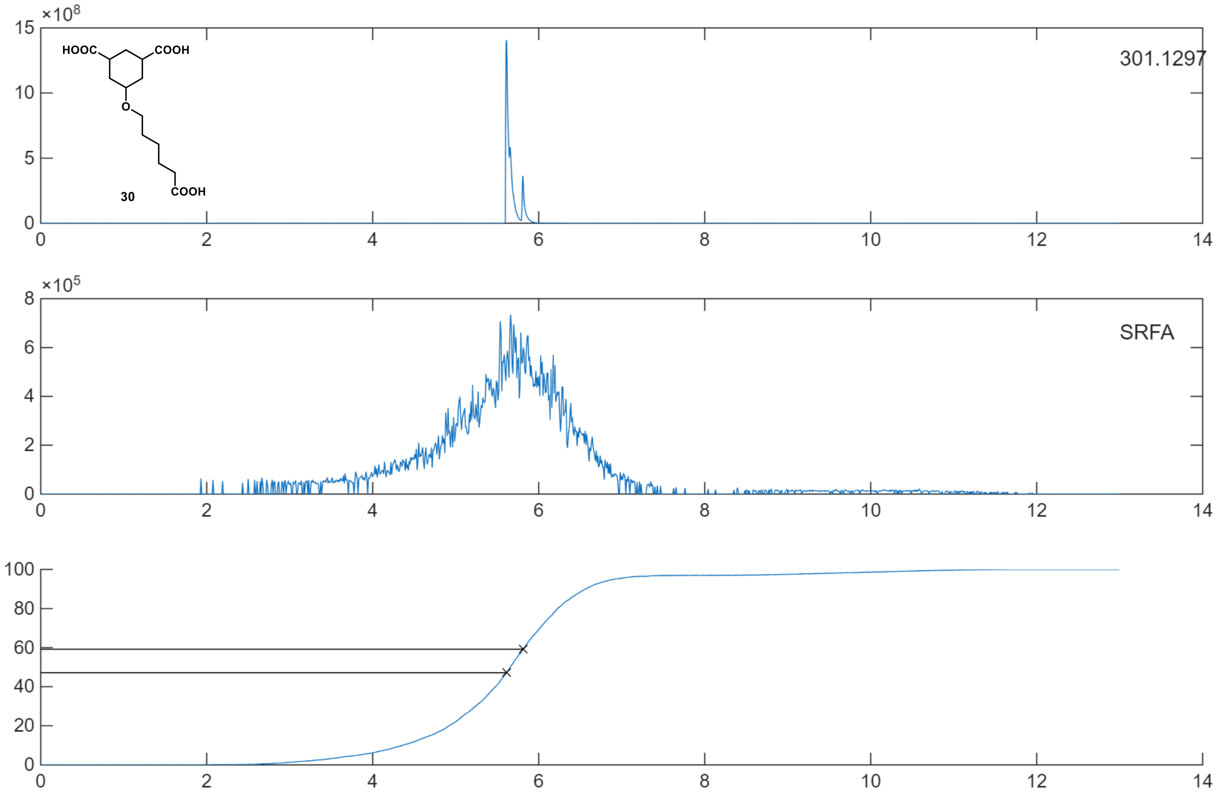

Figure S30: *Top: XIC for 301.1297 m/z for tricarboxylic acid* ***30****, Middle: XIC for 301.1297 for SRFA, Bottom: CI graph for* ***30.***

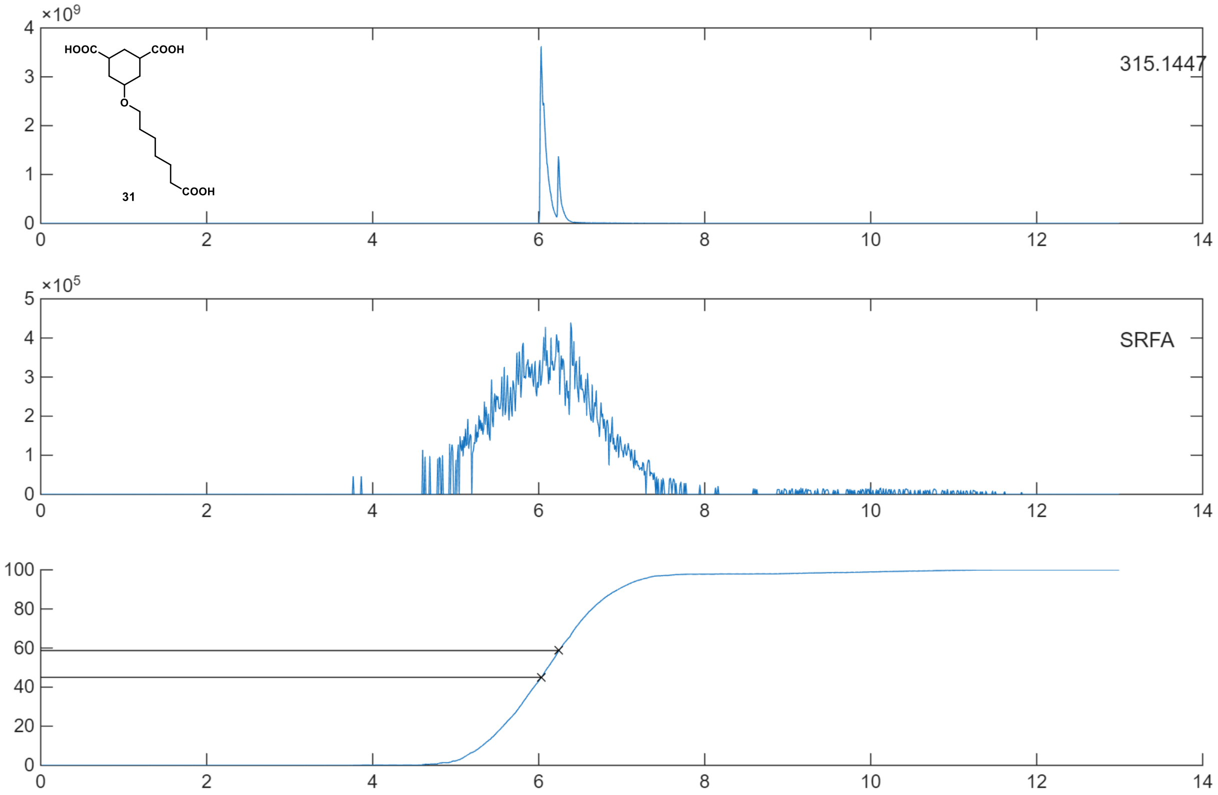

Figure S31: *Top: XIC for 315.1447 m/z for tricarboxylic acid* ***31****, Middle: XIC for 315.1447 for SRFA, Bottom: CI graph for* ***31.***

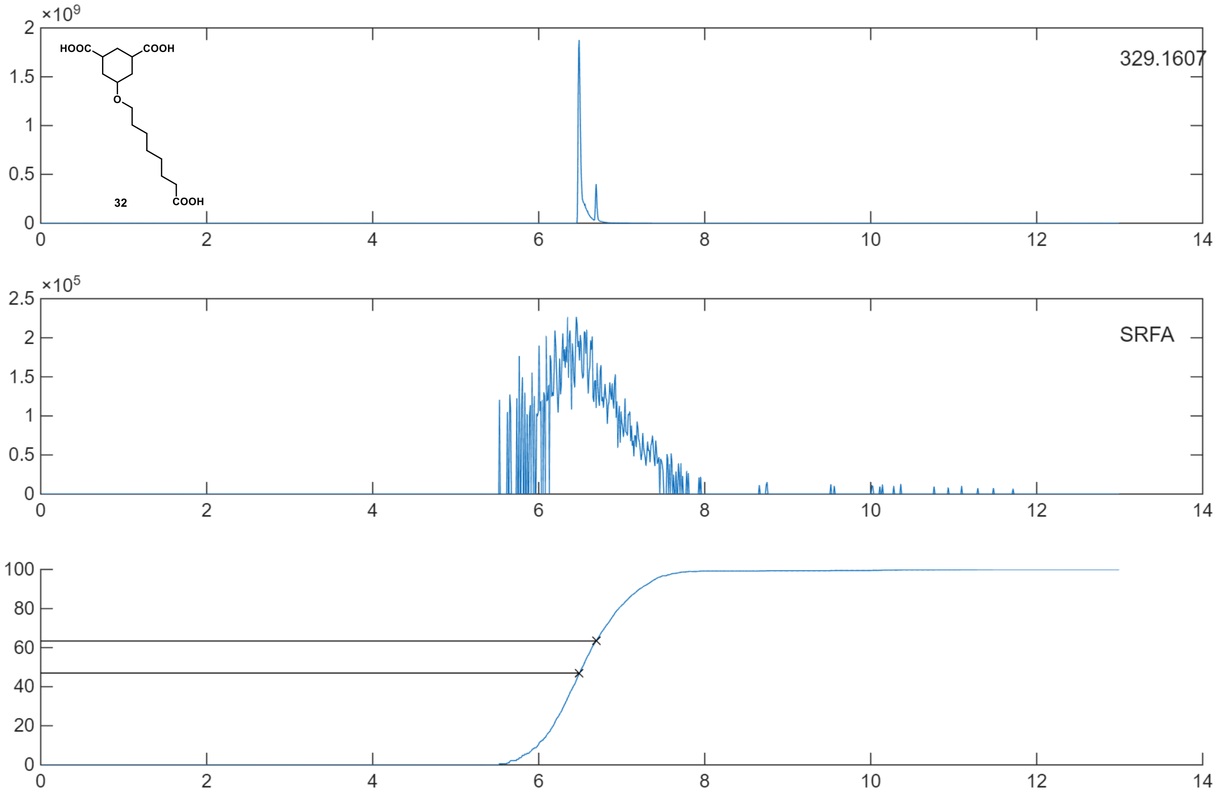

Figure S32: *Top: XIC for 329.1607 m/z for tricarboxylic acid* ***32****, Middle: XIC for 329.1607 for SRFA, Bottom: CI graph for* ***32.***

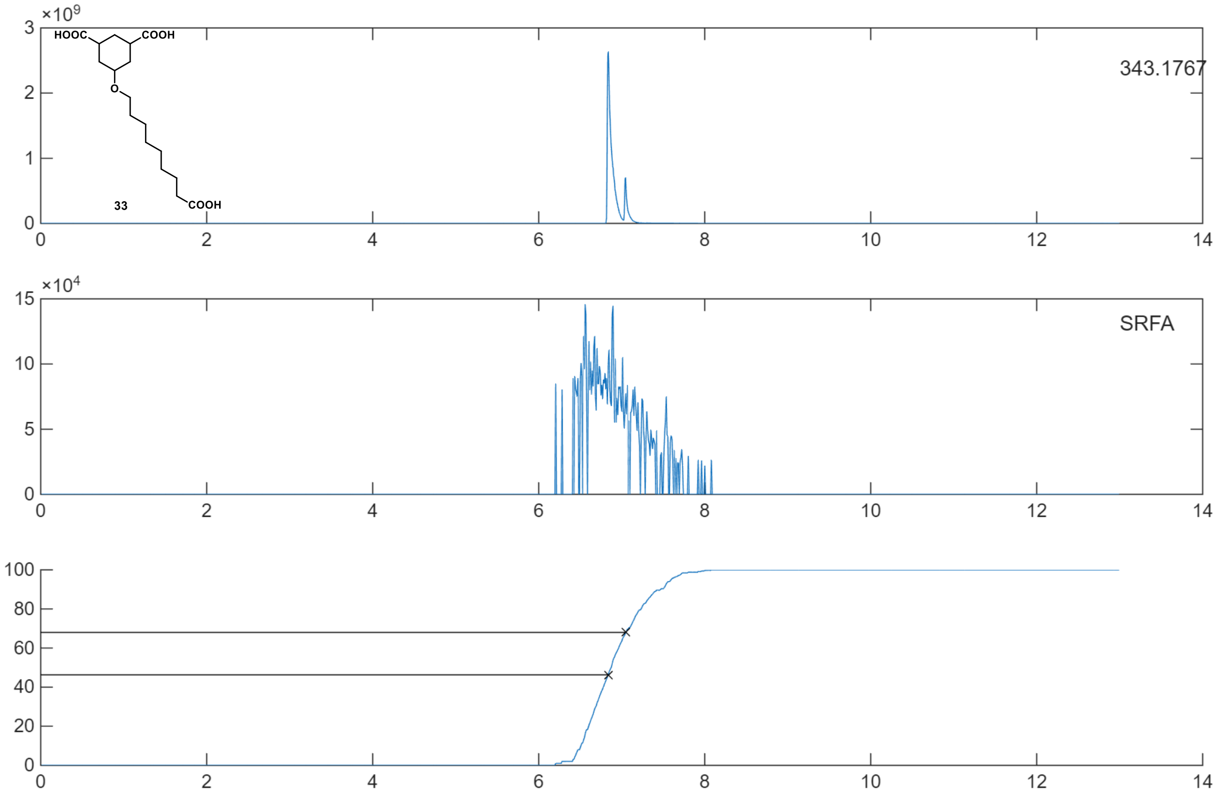

Figure S33: *Top: XIC for 343.1767 m/z for tricarboxylic acid* ***33****, Middle: XIC for 343.1767 for SRFA, Bottom: CI graph for* ***33.***

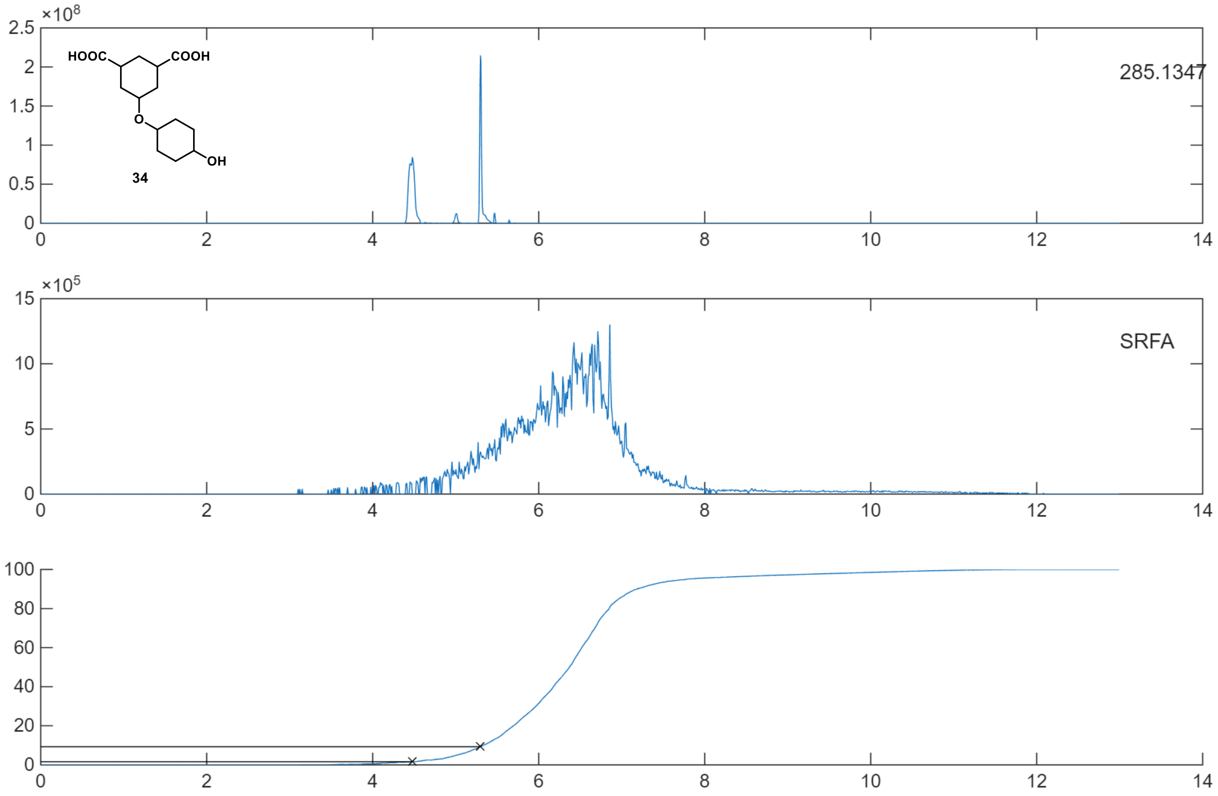

Figure S34: *Top: XIC for 337.1297 m/z for alcohol* ***34****, Middle: XIC for 285.1357 for SRFA, Bottom: CI graph for* ***34.***

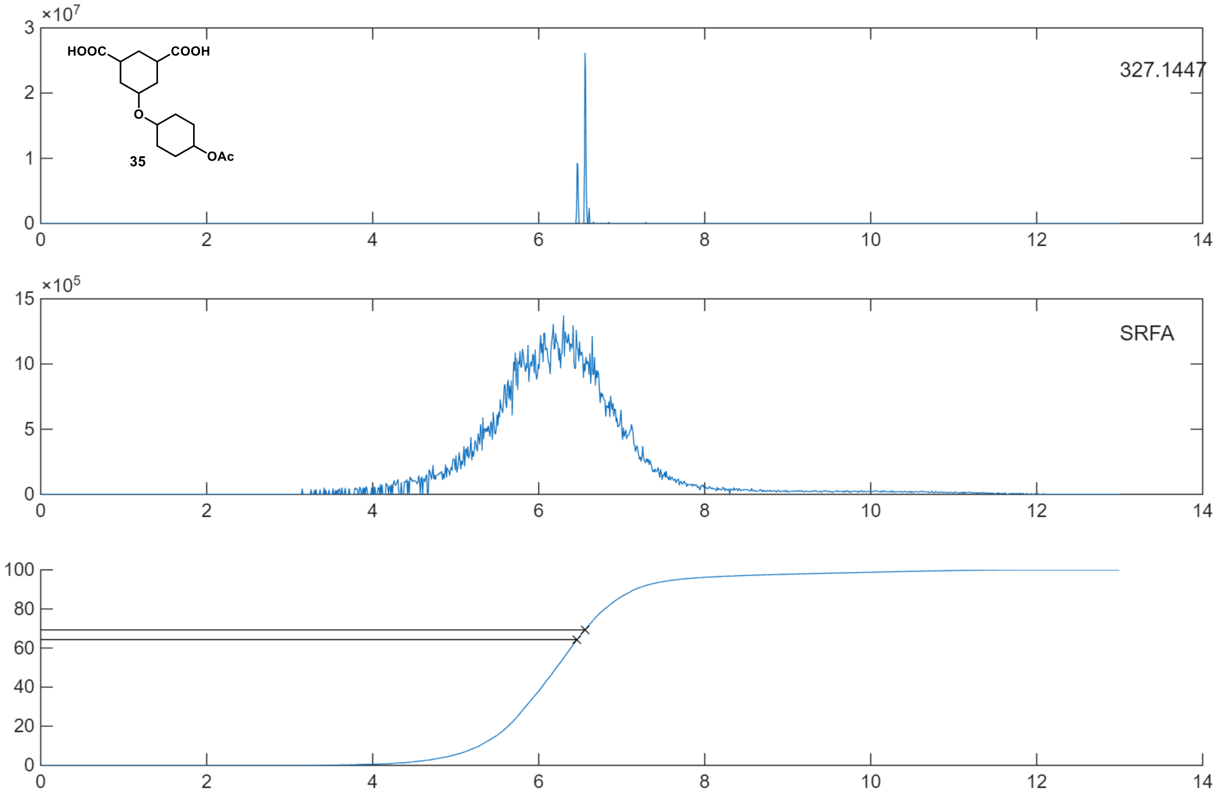

Figure S35: *Top: XIC for 327.1447 m/z for acetyl ester* ***35****, Middle: XIC for 327.1447 for SRFA, Bottom: CI graph for* ***35.***

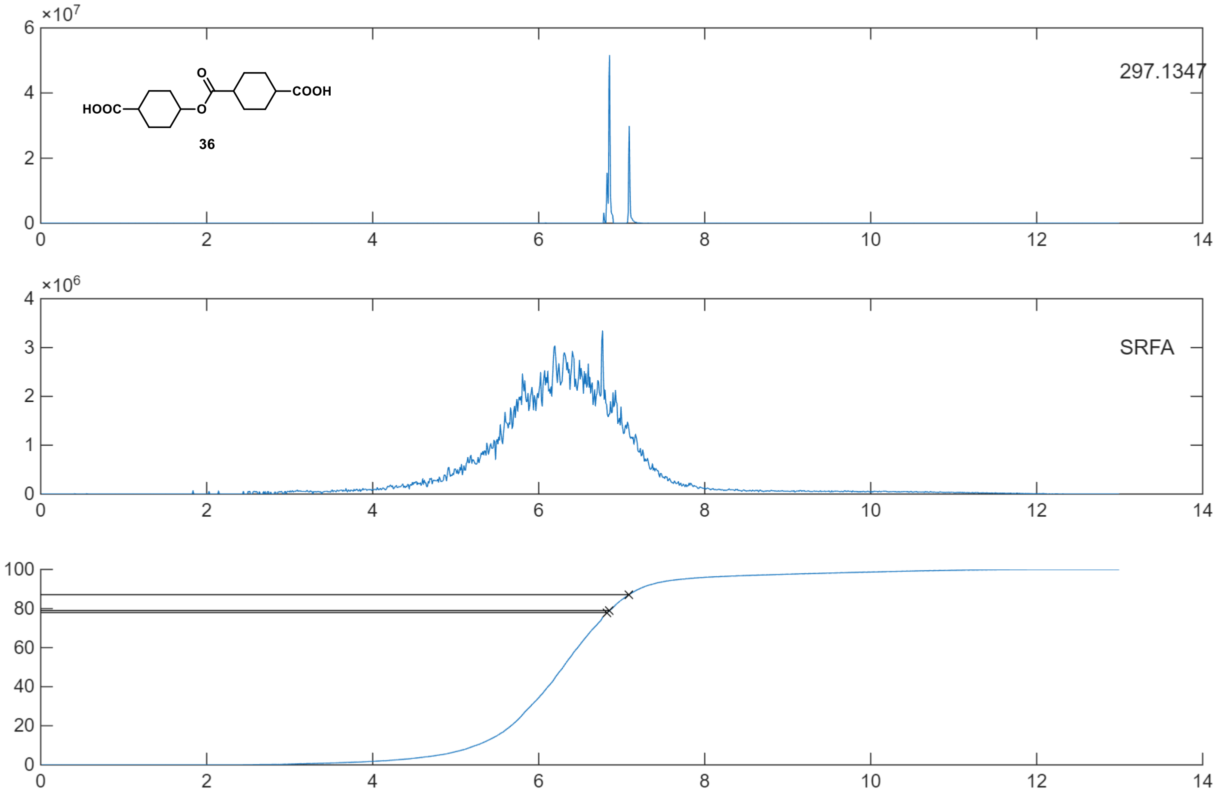

Figure S36: *Top: XIC for 297.1347 m/z for dicarboxylic acid* ***36****, Middle: XIC for 297.1347 for SRFA, Bottom: CI graph for* ***36.***

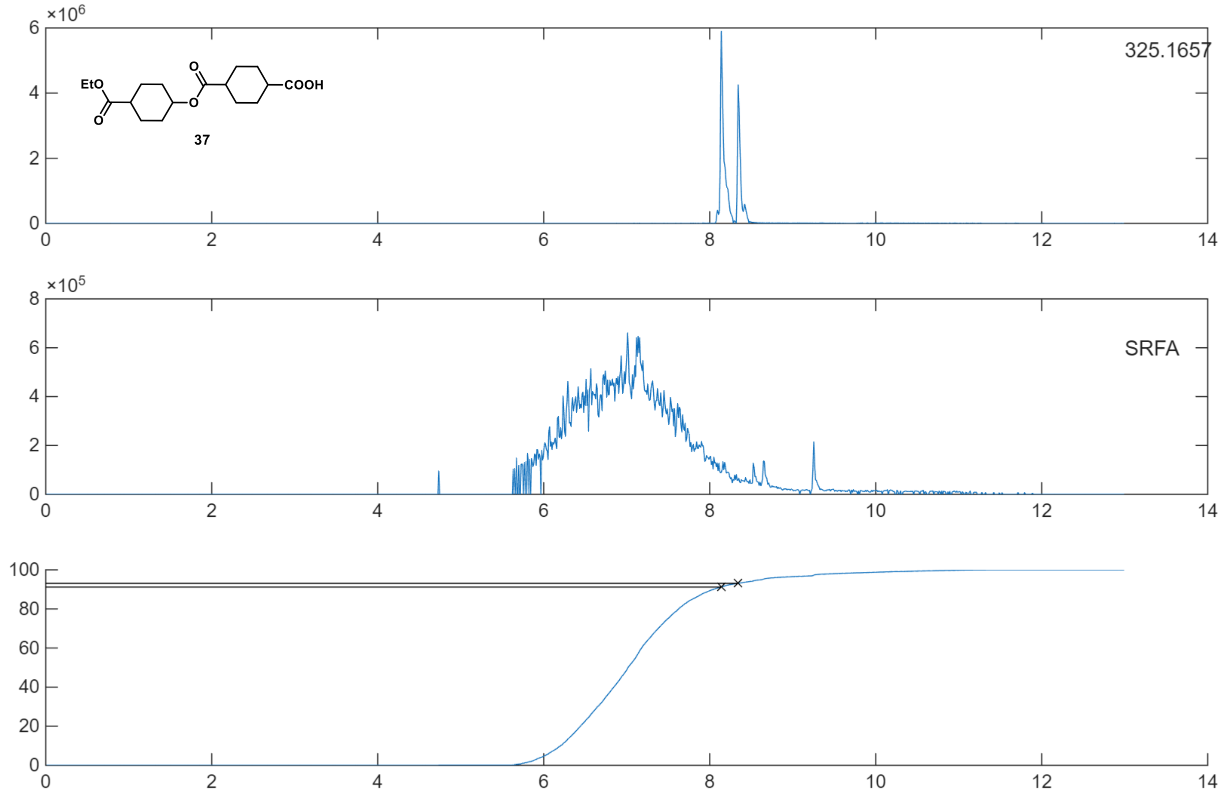

Figure S37: *Top: XIC for 325.1657 m/z for carboxylic acid **37**, Middle: XIC for 325.1657 for SRFA, Bottom: CI graph for **37**.*

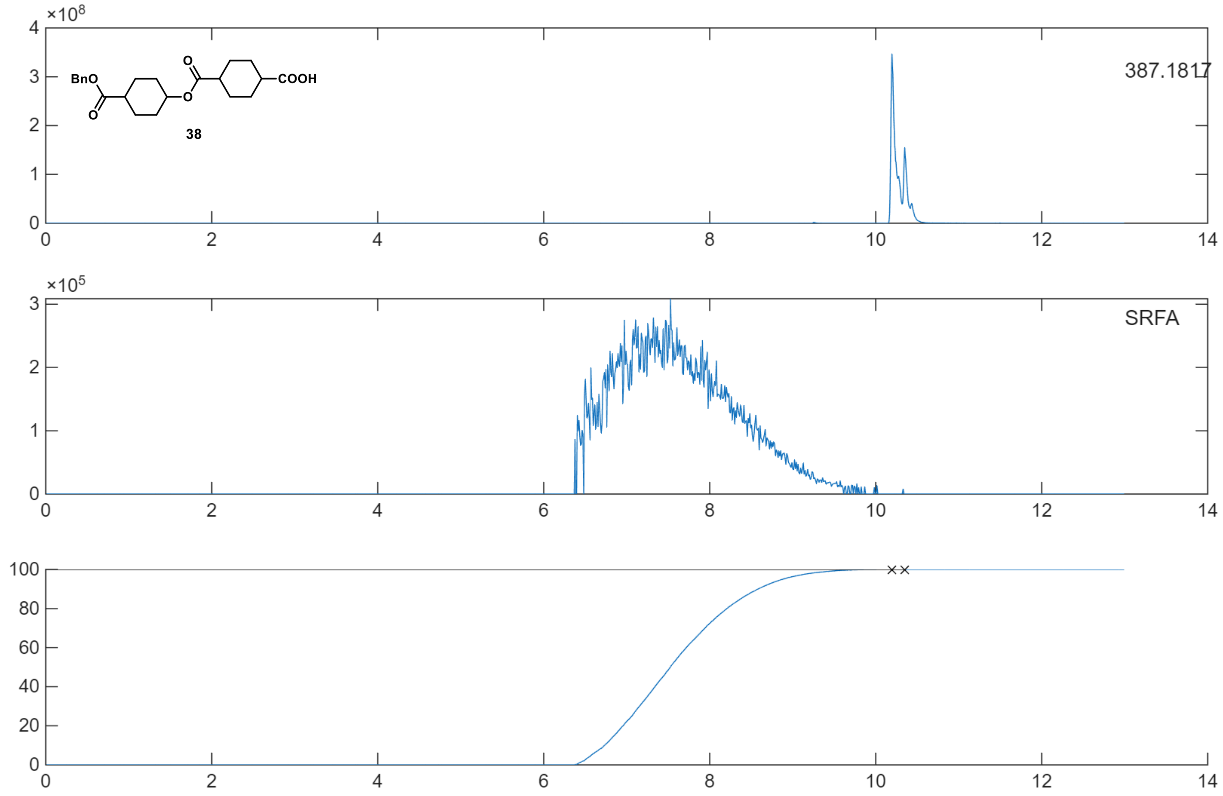

Figure S38: *Top: XIC for 387.1817 m/z for carboxylic acid **38**, Middle: XIC for 387.1817 for SRFA, Bottom: CI graph for **38**.*

Table S1: *35V %Parent, 35V Avg Frag, and Ratio of 35V/Avg Frag (35V Rel avg frag) for* ***1***-***38*** *and Corresponding SRFA Masses.*

Compound	Isomer	35V %Parent	SRFA 35V %Parent	35V Avg Frag	35V Rel avg frag	SRFA 35V Avg Frag	SRFA 35V Rel Avg Frag
1	1	7.5	10.8	197.96	0.75	190.71	0.73
2	1	6.1	10.8	134.37	0.51	190.71	0.73
2	2	0.0	10.8	122.26	0.46	190.71	0.73
3	1	10.3	7.1	150.91	0.49	196.56	0.64
3	2	0.0	7.1	154.50	0.50	196.56	0.64
4	2	0.0	7.1	134.05	0.44	196.56	0.64
4	1	0.0	7.1	151.21	0.49	196.56	0.64
5	1	0.0	7.1	149.54	0.49	196.56	0.64
6	1	0.0	7.1	144.07	0.47	196.56	0.64
6	2	0.0	7.1	127.22	0.41	196.56	0.64
7	1	4.5	7.1	138.12	0.45	196.56	0.64
8	2	0.0	7.1	161.10	0.52	196.56	0.64
8	1	2.6	7.1	139.75	0.46	196.56	0.64
9	1	6.1	5.5	168.83	0.49	228.06	0.66
10	1	9.7	6.3	171.62	0.49	202.96	0.58
10	2	0.1	6.3	160.95	0.46	202.96	0.58
11	1	3.1	9.9	148.82	0.50	184.74	0.62
12	1	6.2	7.5	143.47	0.46	188.78	0.61
13	1	7.3	7.6	151.74	0.47	193.93	0.60
14	1	7.5	6.9	158.18	0.47	197.58	0.59
15	1	3.4	9.3	181.93	0.66	193.28	0.70
16	1	2.4	9.5	184.62	0.66	186.56	0.67
17	1	4.6	9.9	125.87	0.43	184.74	0.62
17	2	4.3	9.9	128.29	0.43	184.74	0.62
18	1	5.3	7.3	139.71	0.48	200.10	0.69
19	2	2.6	7.2	155.31	0.53	191.05	0.65
19	1	0.0	7.2	152.43	0.52	191.05	0.65
20	1	0.0	6.9	157.05	0.47	197.58	0.59
21	1	1.9	7.3	136.91	0.47	200.10	0.69
22	1	11.2	15.9	195.84	0.73	174.04	0.65
22	2	52.7	15.9	234.40	0.87	174.04	0.65
23	2	63.9	10.5	234.40	0.88	187.11	0.60
23	1	41.7	10.5	220.96	0.70	187.11	0.60
24	2	29.4	10.5	244.02	0.78	187.11	0.60
24	1	9.4	10.5	178.30	0.57	187.11	0.60
25	2	29.8	10.5	195.48	0.62	187.11	0.60
25	1	2.6	10.5	179.03	0.57	187.11	0.60
26	1	44.4	10.5	238.95	0.76	187.11	0.60
27	1	48.4	6.2	307.19	0.86	191.66	0.54
28	1	40.1	6.2	255.40	0.71	198.68	0.56
29	2	36.7	18.2	185.66	0.76	161.48	0.66
29	1	14.4	18.2	140.08	0.58	161.48	0.66
30	2	25.5	8.2	230.58	0.77	191.23	0.64
30	1	37.2	8.2	240.35	0.80	191.23	0.64
31	2	30.4	8.4	258.78	0.82	197.08	0.63
31	1	34.1	8.4	262.56	0.83	197.08	0.63
32	2	28.1	11.0	271.94	0.82	198.94	0.60
32	1	32.6	11.0	274.80	0.83	198.94	0.60
33	2	28.2	7.0	284.32	0.83	198.00	0.58
33	1	31.0	7.0	284.84	0.83	198.00	0.58
34	1	53.1	12.4	247.35	0.87	179.91	0.63
34	2	61.5	12.4	253.61	0.89	179.91	0.63
35	1	37.2	8.1	256.24	0.78	189.82	0.58
35	2	28.1	8.1	209.19	0.64	189.82	0.58
36	1	22.0	12.2	184.80	0.62	185.77	0.63
36	2	0.9	12.2	165.19	0.56	185.77	0.63
37	1	16.2	8.5	197.99	0.61	189.72	0.58
37	2	11.0	8.5	221.46	0.68	189.72	0.58
38	2	4.8	3.9	151.57	0.39	219.35	0.57
38	1	7.4	3.9	173.05	0.45	219.35	0.57

Table S2: *75V Avg Frag, and Ratio of 75V/Avg Frag (75V Rel avg frag) for* ***1****-****38*** *and Corresponding SRFA Masses.*

Compound	Isomer	75V Avg Frag	75V Rel avg frag	SRFA 75V Avg Frag	SRFA 75V Rel Avg Frag
1	1	95.96	0.36	129.93	0.49
2	1	96.30	0.36	129.93	0.49
2	2	94.41	0.36	129.93	0.49
3	1	94.15	0.31	125.02	0.41
3	2	91.25	0.30	125.02	0.41
4	2	95.69	0.31	125.02	0.41
4	1	93.14	0.30	125.02	0.41
5	1	93.75	0.31	125.02	0.41
6	1	95.69	0.31	125.02	0.41
6	2	94.61	0.31	125.02	0.41
7	1	96.84	0.32	125.02	0.41
8	2	92.40	0.30	125.02	0.41
8	1	93.48	0.30	125.02	0.41
9	1	93.72	0.27	134.82	0.39
10	1	96.62	0.28	118.64	0.34
10	2	94.39	0.27	118.64	0.34
11	1	93.64	0.32	112.24	0.38
12	1	93.60	0.30	112.07	0.36
13	1	93.10	0.29	114.55	0.35
14	1	92.71	0.28	116.23	0.34
15	1	91.82	0.33	132.79	0.48
16	1	93.22	0.33	121.91	0.44
17	1	94.37	0.32	112.24	0.38
17	2	93.93	0.32	112.24	0.38
18	1	121.30	0.42	135.24	0.46
19	2	123.49	0.42	122.55	0.42
19	1	122.73	0.42	122.55	0.42
20	1	129.43	0.38	116.23	0.34
21	1	116.50	0.40	135.24	0.46
22	1	98.06	0.36	99.66	0.37
22	2	101.33	0.38	99.66	0.37
23	2	122.50	0.39	97.35	0.31
23	1	113.21	0.36	97.35	0.31
24	2	119.59	0.38	97.35	0.31
24	1	119.52	0.38	97.35	0.31
25	2	117.84	0.38	97.35	0.31
25	1	122.67	0.39	97.35	0.31
26	1	115.02	0.37	97.35	0.31
27	1	158.24	0.44	98.50	0.28
28	1	110.93	0.31	102.41	0.29
29	2	113.89	0.47	86.94	0.36
29	1	89.68	0.37	86.94	0.36
30	2	94.35	0.31	115.72	0.38
30	1	102.30	0.34	115.72	0.38
31	2	112.25	0.36	108.42	0.34
31	1	114.01	0.36	108.42	0.34
32	2	126.52	0.38	102.48	0.31
32	1	126.27	0.38	102.48	0.31
33	2	139.33	0.40	104.26	0.30
33	1	134.82	0.39	104.26	0.30
34	1	113.97	0.40	99.75	0.35
34	2	116.24	0.41	99.75	0.35
35	1	109.37	0.33	105.23	0.32
35	2	67.73	0.21	105.23	0.32
36	1	134.32	0.45	102.24	0.34
36	2	131.80	0.44	102.24	0.34
37	1	135.64	0.42	104.11	0.32
37	2	123.12	0.38	104.11	0.32
38	2	105.87	0.27	133.87	0.34
38	1	110.27	0.28	133.87	0.34

Table S3: *35V %FG and 75V %FG for* ***1-38*** *and Corresponding SRFA Masses.*

Compound	Isomer	35V %FG	SRFA 35V %FG	75V %FG	SRFA 75V %FG
1	1	68.9	68.9	0.6	22.7
2	1	0.0	68.9	0.0	22.7
2	2	0.0	68.9	0.0	22.7
3	1	8.0	58.7	0.0	11.8
3	2	0.0	58.7	0.0	11.8
4	2	5.0	58.7	0.0	11.8
4	1	0.0	58.7	0.0	11.8
5	1	3.9	58.7	0.0	11.8
6	1	13.4	58.7	0.0	11.8
6	2	0.0	58.7	0.0	11.8
7	1	0.0	58.7	0.0	11.8
8	2	0.0	58.7	0.0	11.8
8	1	0.0	58.7	0.0	11.8
9	1	25.4	74.2	0.0	28.1
10	1	1.3	45.1	0.0	7.4
10	2	0.0	45.1	0.0	7.4
11	1	2.0	51.3	0.0	17.0
12	1	8.5	51.6	0.0	12.9
13	1	18.7	46.9	0.0	7.7
14	1	23.1	48.2	0.0	8.4
15	1	53.5	68.6	0.0	30.6
16	1	12.9	61.3	0.0	15.4
17	1	4.0	51.3	0.0	17.0
17	2	3.2	51.3	0.0	17.0
18	1	2.0	68.7	0.0	20.4
19	2	0.0	49.6	0.0	14.2
19	1	0.0	49.6	0.0	14.2
20	1	0.0	48.2	0.0	8.4
21	1	0.0	68.7	0.0	20.4
22	1	45.3	48.3	0.0	16.1
22	2	33.6	48.3	2.0	16.1
23	2	20.5	45.8	0.8	9.6
23	1	0.1	45.8	0.0	9.6
24	2	41.2	45.8	0.8	9.6
24	1	4.4	45.8	0.0	9.6
25	2	4.6	45.8	0.0	9.6
25	1	0.0	45.8	0.0	9.6
26	1	15.3	45.8	0.0	9.6
27	1	36.2	38.1	4.4	5.4
28	1	14.6	47.6	0.0	6.5
29	2	10.9	44.8	1.5	12.3
29	1	4.3	44.8	0.0	12.3
30	2	47.3	55.0	0.0	27.2
30	1	35.0	55.0	0.7	27.2
31	2	52.8	53.9	1.7	16.3
31	1	48.9	53.9	2.3	16.3
32	2	56.9	50.1	2.4	15.5
32	1	51.6	50.1	3.0	15.5
33	2	57.4	52.0	3.0	14.4
33	1	53.3	52.0	2.7	14.4
34	1	31.9	47.0	3.3	15.5
34	2	25.7	47.0	4.4	15.5
35	1	28.0	43.9	0.0	12.1
35	2	6.1	43.9	0.0	12.1
36	1	0.0	44.6	0.0	10.0
36	2	0.0	44.6	0.0	10.0
37	1	0.0	43.4	0.0	7.0
37	2	0.0	43.4	0.0	7.0
38	2	5.7	59.4	0.0	24.4
38	1	0.7	59.4	0.0	24.4

Table S4: *35V %Radical and 75V %Radical for* ***1-38*** *and Corresponding SRFA Masses.*

Compound	Isomer	35V %Radical	35V SRFA %Radical	75V %Radical	75V SRFA %Radical
1	1	6.7	1.7	48.4	11.8
2	1	0.0	1.7	0.0	11.8
2	2	0.0	1.7	0.0	11.8
3	1	0.0	1.1	0.0	9.9
3	2	0.0	1.1	0.0	9.9
4	2	0.0	1.1	0.0	9.9
4	1	0.0	1.1	0.0	9.9
5	1	0.0	1.1	0.0	9.9
6	1	0.0	1.1	0.0	9.9
6	2	0.0	1.1	0.0	9.9
7	1	0.0	1.1	0.0	9.9
8	2	0.0	1.1	0.0	9.9
8	1	0.0	1.1	0.0	9.9
9	1	0.0	1.5	4.3	12.2
10	1	0.0	0.5	0.0	8.6
10	2	0.0	0.5	0.0	8.6
11	1	0.0	0.6	0.0	6.7
12	1	0.0	0.5	0.0	6.4
13	1	0.0	0.5	0.0	9.8
14	1	0.0	0.5	0.0	7.7
15	1	5.1	3.1	27.4	12.4
16	1	2.1	2.0	15.2	12.7
17	1	0.0	0.6	0.0	6.7
17	2	0.0	0.6	1.5	6.7
18	1	0.9	2.2	1.1	13.2
19	2	23.8	5.8	90.3	9.0
19	1	36.0	5.8	98.3	9.0
20	1	21.1	0.5	95.1	7.7
21	1	0.0	2.2	0.0	13.2
22	1	0.0	0.2	0.0	5.7
22	2	0.0	0.2	0.0	5.7
23	2	0.0	0.3	0.7	5.3
23	1	0.0	0.3	0.0	5.3
24	2	0.0	0.3	0.0	5.3
24	1	0.0	0.3	0.0	5.3
25	2	0.0	0.3	0.0	5.3
25	1	0.0	0.3	0.0	5.3
26	1	0.0	0.3	1.1	5.3
27	1	0.0	0.2	0.5	5.4
28	1	0.0	0.8	0.8	8.4
29	2	0.0	1.8	0.0	9.8
29	1	0.0	1.8	0.0	9.8
30	2	0.0	0.9	0.0	5.3
30	1	0.0	0.9	1.3	5.3
31	2	0.0	1.0	0.0	6.9
31	1	0.0	1.0	1.2	6.9
32	2	0.0	0.9	0.0	7.9
32	1	0.0	0.9	0.6	7.9
33	2	0.0	0.7	0.0	7.9
33	1	0.0	0.7	0.3	7.9
34	1	0.0	0.5	4.0	3.7
34	2	0.0	0.5	1.7	3.7
35	1	0.0	0.6	0.0	6.4
35	2	0.0	0.6	0.0	6.4
36	1	0.0	0.4	0.0	5.5
36	2	0.0	0.4	0.0	5.5
37	1	0.0	0.1	0.0	6.8
37	2	0.0	0.1	5.4	6.8
38	2	0.0	1.7	19.4	9.9
38	1	0.0	1.7	16.9	9.9

Table S5: *Avg Frag, %Radical, %FG, %Parent, and Rel Avg Frag for DOM samples SRFA, CJ11, DS11, DS1, NR, and P at 35V for m/z 263, 277, 293, 301, 307, 313, 325, 337, 343, and 357 with no mass defect filter.*

Sample, *m/z*	Avg Frag	%Radical	%FG	%Parent	Rel Avg Frag
SRFA, 263	190.71	1.7	68.9	10.8	0.73
CJ11, 263	196.45	2.0	60.8	18.0	0.75
DS11, 263	188.79	3.8	53.1	17.4	0.72
DS1, 263	194.34	3.0	57.0	18.8	0.74
NR, 263	203.39	2.8	62.0	21.3	0.77
P, 263	200.50	2.1	61.1	20.6	0.76
SRFA, 277	193.28	3.1	68.6	9.3	0.70
CJ11, 277	199.68	3.6	59.7	15.4	0.72
DS11, 277	190.55	5.5	52.0	14.7	0.69
DS1, 277	197.61	4.1	55.8	15.9	0.71
NR, 277	203.68	4.7	63.7	16.4	0.74
P, 277	198.42	5.1	62.7	15.3	0.72
SRFA, 293	191.05	5.8	49.6	7.2	0.65
CJ11, 293	197.79	0.9	47.5	13.0	0.68
DS11, 293	190.44	3.0	35.0	15.6	0.65
DS1, 293	196.43	1.3	45.6	13.1	0.67
NR, 293	202.98	1.2	51.6	14.6	0.69
P, 293	201.53	0.9	51.4	13.9	0.69
SRFA, 301	191.23	0.9	55.0	8.2	0.64
CJ11, 301	207.44	1.8	55.4	17.0	0.69
DS11, 301	193.05	3.0	39.5	21.4	0.64
DS1, 301	192.23	2.9	44.6	10.3	0.64
NR, 301	219.65	1.5	54.4	23.7	0.73
P, 301	199.54	1.2	36.8	28.1	0.66
SRFA, 307	196.56	1.1	58.7	7.1	0.64
CJ11, 307	202.62	0.8	49.0	12.2	0.66
DS11, 307	205.18	4.7	42.1	14.9	0.67
DS1, 307	201.08	1.5	48.7	12.2	0.66
NR, 307	208.84	0.8	55.3	13.2	0.68
P, 307	207.44	1.0	54.9	13.5	0.68
SRFA, 313	187.11	0.3	45.8	10.5	0.60
CJ11, 313	192.04	0.1	43.3	12.7	0.61
DS11, 313	188.44	1.5	35.6	16.5	0.60
DS1, 313	186.22	1.0	38.4	14.0	0.60
NR, 313	201.34	0.4	45.5	17.2	0.64
P, 313	182.35	0.7	41.3	12.1	0.58
SRFA, 325	189.72	0.1	43.4	8.5	0.58
CJ1, 325	196.01	0.0	39.2	11.5	0.60
DS11, 325	194.70	1.1	36.0	12.8	0.60
DS1, 325	191.80	0.5	39.0	10.5	0.59
NR, 325	200.34	0.3	41.4	13.8	0.62
P, 325	193.77	0.4	43.2	10.3	0.60
SRFA, 337	197.58	0.5	48.2	6.9	0.59
CJ11, 337	205.64	0.3	42.2	11.2	0.61
DS11, 337	209.03	0.8	37.7	13.5	0.62
DS1, 337	202.04	0.8	42.1	9.6	0.60
NR, 337	210.72	0.1	44.8	13.5	0.63
P, 337	206.22	0.4	45.8	11.1	0.61
SRFA, 343	198.00	0.7	52.0	7.0	0.58
CJ11, 343	197.30	4.4	46.6	7.9	0.58
DS11, 343	183.18	4.4	35.2	9.3	0.53
DS1, 343	184.93	2.6	38.5	8.5	0.54
NR, 343	190.07	17.6	41.7	8.5	0.55
P, 343	168.32	8.4	32.4	7.2	0.49
SRFA, 357	198.68	0.8	47.6	6.2	0.56
CJ11, 357	201.97	1.0	45.3	7.1	0.57
DS11, 357	184.21	3.1	31.8	8.3	0.52
DS1, 357	184.05	1.7	35.1	6.6	0.52
NR, 357	218.93	1.2	52.6	11.3	0.61
P, 357	175.69	1.2	34.1	6.2	0.49

Table S6: *Avg Frag, %Radical, %FG, %Parent, and Rel Avg Frag for DOM samples SRFA, CJ11, DS11, DS1, NR, and P at 75V for m/z 263, 277, 293, 301, 307, 313, 325, 337, 343, and 357 with no mass defect filter.*

Sample, *m/z*	Avg Frag	%Radical	%FG	%Parent	Rel Avg Frag
SRFA, 263	129.93	11.8	22.7	0.0	0.49
CJ11, 263	126.48	9.5	25.8	0.0	0.48
DS11, 263	118.55	16.9	17.8	0.0	0.45
DS1, 263	123.18	13.5	24.0	0.0	0.47
NR, 263	125.15	13.0	23.8	0.0	0.48
P, 263	123.16	13.7	27.5	0.0	0.47
SRFA, 277	132.79	12.4	30.6	0.0	0.48
CJ11, 277	129.42	10.5	30.4	0.0	0.47
DS11, 277	115.77	17.3	18.5	0.0	0.42
DS1, 277	124.59	14.4	26.7	0.0	0.45
NR, 277	127.62	12.2	31.0	0.0	0.46
P, 277	124.04	14.5	32.2	0.0	0.45
SRFA, 293	122.55	9.0	14.2	0.0	0.42
CJ11, 293	119.13	8.7	9.6	0.0	0.41
DS11, 293	105.38	17.1	6.3	0.0	0.36
DS1,293	118.39	11.3	9.3	0.0	0.40
NR, 293	116.05	9.7	9.6	0.0	0.40
P, 293	116.48	10.8	11.6	0.0	0.40
SRFA, 301	115.72	5.3	27.2	0.0	0.38
CJ11, 301	116.96	6.0	24.4	0.0	0.39
DS11, 301	110.94	19.4	14.6	0.6	0.37
DS1, 301	102.93	17.6	15.5	0.0	0.34
NR, 301	105.28	18.2	16.8	0.0	0.35
P, 301	99.03	19.5	11.7	0.0	0.33
SRFA, 307	125.02	9.9	11.8	0.0	0.41
CJ11, 307	120.08	7.7	10.5	0.0	0.39
DS11, 307	112.44	16.3	7.4	0.0	0.37
DS1, 307	119.99	12.1	11.0	0.0	0.39
NR, 307	114.80	6.5	8.6	0.0	0.37
P, 307	117.46	10.0	11.4	0.0	0.38
SRFA, 313	97.35	5.3	9.6	0.0	0.31
CJ11, 313	96.52	4.5	7.6	0.0	0.31
DS11, 313	104.61	13.7	6.3	0.0	0.33
DS1, 313	101.27	10.6	7.6	0.0	0.32
NR, 313	89.43	11.0	3.8	0.0	0.29
P, 313	91.94	10.0	4.9	0.0	0.29
SRFA, 325	104.11	6.8	7.0	0.0	0.32
CJ11, 325	101.94	4.7	6.6	0.0	0.31
DS11, 325	101.16	10.6	6.7	0.0	0.31
DS1, 325	107.02	8.3	7.7	0.0	0.33
NR, 325	93.93	6.2	4.3	0.0	0.29
P, 325	101.64	6.9	6.8	0.0	0.31
SRFA, 337	116.23	7.7	8.4	0.0	0.34
CJ11, 337	112.21	6.9	6.6	0.0	0.33
DS11, 337	106.91	11.7	6.2	0.0	0.32
DS1, 337	115.28	10.1	7.8	0.0	0.34
NR, 337	103.07	6.0	4.9	0.0	0.31
P, 337	111.59	9.0	8.1	0.0	0.33
SRFA, 343	104.26	7.9	14.4	0.0	0.30
CJ11, 343	114.71	8.8	18.2	0.0	0.33
DS11, 343	101.02	16.7	9.2	0.0	0.29
DS1, 343	104.95	13.9	13.0	0.0	0.31
NR, 343	105.75	20.3	13.1	0.0	0.31
P, 343	92.71	15.7	7.7	0.0	0.27
SRFA, 357	102.41	8.4	6.5	0.0	0.29
CJ11, 357	112.49	7.8	10.0	0.0	0.32
DS11, 357	98.57	16.3	4.3	0.0	0.28
DS1, 357	104.87	12.1	7.7	0.0	0.29
NR, 357	104.95	15.3	6.9	0.0	0.29
P, 357	95.43	11.8	3.7	0.0	0.27

Table S7: *Avg Frag, %Radical, %FG, and %Parent for DOM samples SRFA, CJ11, DS11, DS1, NR, and P at 35V for m/z 263, 277, 293, 301, 307, 313, 325, 337, 343, and 357 excluding mass defects from x.5 to x.0.*

Sample, *m/z*	Avg Frag	%Radical	%FG	%Parent	Rel Avg Frag
SRFA, 263	190.71	1.7	68.9	10.8	0.73
CJ11, 263	196.73	2.1	61.1	18.0	0.75
DS11, 263	195.18	2.8	56.4	18.5	0.74
DS1, 263	196.55	2.2	58.1	19.2	0.75
NR, 263	205.75	1.5	64.8	22.3	0.78
P, 263	202.78	0.6	64.0	21.5	0.77
SRFA, 277	193.28	3.1	68.6	9.3	0.70
CJ11, 277	200.56	3.6	60.1	15.5	0.72
DS11, 277	198.85	4.6	56.1	15.8	0.72
DS1, 277	200.60	3.5	57.2	16.3	0.72
NR, 277	203.77	4.5	63.8	16.5	0.74
P, 277	201.10	4.5	64.2	15.7	0.73
SRFA, 293	191.05	5.8	49.6	7.2	0.65
CJ11, 293	198.44	0.9	47.7	13.0	0.68
DS11, 293	201.75	1.7	39.3	17.5	0.69
DS1, 293	198.96	0.9	46.6	13.4	0.68
NR, 293	203.79	0.7	52.6	14.9	0.70
P, 293	202.73	0.3	53.0	14.3	0.69
SRFA, 301	191.23	0.9	55.0	8.2	0.64
CJ11, 301	208.19	1.9	57.0	16.1	0.69
DS11, 301	198.83	1.8	44.5	20.8	0.66
DS1, 301	199.27	1.6	48.4	10.2	0.66
NR, 301	223.46	1.2	58.3	23.1	0.74
P, 301	188.98	0.0	46.4	16.5	0.63
SRFA, 307	196.56	1.1	58.7	7.1	0.64
CJ11, 307	203.54	0.8	49.3	12.4	0.66
DS11, 307	211.06	1.1	46.5	16.6	0.69
DS1, 307	203.97	1.1	49.9	12.5	0.66
NR, 307	209.46	0.8	55.6	13.4	0.68
P, 307	209.73	0.6	55.8	13.8	0.68
SRFA, 313	187.11	0.3	45.8	10.5	0.60
CJ11, 313	194.51	0.1	44.2	13.0	0.62
DS11, 313	198.08	0.0	38.8	18.0	0.63
DS1, 313	192.04	0.2	40.5	14.8	0.61
NR, 313	207.67	0.0	48.0	18.1	0.66
P, 313	186.95	0.0	43.1	12.6	0.60
SRFA, 325	189.72	0.1	43.4	8.5	0.58
CJ1, 325	198.17	0.0	39.9	11.7	0.61
DS11, 325	201.91	0.1	38.4	13.6	0.62
DS1, 325	195.01	0.1	40.1	10.8	0.60
NR, 325	204.23	0.0	42.8	14.3	0.63
P, 325	196.37	0.0	44.2	10.6	0.60
SRFA, 337	197.58	0.5	48.2	6.9	0.59
CJ11, 337	207.44	0.3	42.8	11.3	0.62
DS11, 337	217.14	0.0	40.2	14.4	0.64
DS1, 337	205.09	0.5	43.1	9.8	0.61
NR, 337	213.11	0.0	45.7	13.8	0.63
P, 337	209.45	0.0	47.0	11.4	0.62
SRFA, 343	198.00	0.7	52.0	7.0	0.58
CJ11, 343	205.98	0.9	51.0	8.6	0.60
DS11, 343	198.13	0.8	40.9	10.8	0.58
DS1, 343	194.29	0.7	42.1	9.3	0.57
NR, 343	228.67	0.7	62.8	12.9	0.67
P, 343	182.83	0.1	39.9	8.9	0.53
SRFA, 357	198.68	0.8	47.6	6.2	0.56
CJ11, 357	206.27	0.7	47.0	7.4	0.58
DS11, 357	198.67	0.4	36.5	9.5	0.56
DS1, 357	191.82	0.7	37.8	7.1	0.54
NR, 357	228.33	0.4	56.7	12.2	0.64

Table S8: *Avg Frag, %Radical, %FG, %Parent, and Rel Avg Frag for DOM samples SRFA, CJ11, DS11, DS1, NR, and P at 75V for m/z 263, 277, 293, 301, 307, 313, 325, 337, 343, and 357 excluding mass defects from x.5 to x.0.*

Sample, *m/z*	Avg Frag	%Radical	%FG	%Parent	Rel Avg Frag
SRFA, 263	129.93	11.8	22.7	0.0	0.49
CJ11, 263	127.22	7.9	27.3	0.0	0.48
DS11, 263	128.73	10.9	23.7	0.0	0.49
DS1, 263	127.32	8.5	26.8	0.0	0.48
NR, 263	128.31	5.4	29.8	0.0	0.49
P, 263	128.62	4.2	35.5	0.0	0.49
SRFA, 277	132.79	12.4	30.6	0.0	0.48
CJ11, 277	131.06	10.3	31.6	0.0	0.47
DS11, 277	128.35	11.2	26.0	0.0	0.46
DS1, 277	130.60	10.0	30.4	0.0	0.47
NR, 277	130.02	9.2	33.1	0.0	0.47
P, 277	131.34	7.5	38.0	0.0	0.47
SRFA, 293	122.55	9.0	14.2	0.0	0.42
CJ11, 293	120.05	8.4	9.9	0.0	0.41
DS11, 293	117.29	7.6	10.2	0.0	0.40
DS1,293	122.45	9.0	10.3	0.0	0.42
NR, 293	117.33	6.5	10.3	0.0	0.40
P, 293	119.69	6.5	12.9	0.0	0.41
SRFA, 301	115.72	5.3	27.2	0.0	0.38
CJ11, 301	120.18	3.4	26.3	0.0	0.40
DS11, 301	124.84	9.6	21.8	0.9	0.41
DS1, 301	114.29	2.2	23.4	0.0	0.38
NR, 301	113.49	1.6	25.4	0.0	0.38
P, 301	103.68	2.2	17.2	0.0	0.34
SRFA, 307	125.02	9.9	11.8	0.0	0.41
CJ11, 307	121.23	7.6	10.8	0.0	0.39
DS11, 307	122.63	9.0	10.2	0.0	0.40
DS1, 307	124.28	9.5	12.2	0.0	0.40
NR, 307	116.71	4.8	9.3	0.0	0.38
P, 307	121.28	6.1	12.7	0.0	0.40
SRFA, 313	97.35	5.3	9.6	0.0	0.31
CJ11, 313	98.04	2.5	8.2	0.0	0.31
DS11, 313	114.16	3.3	9.1	0.0	0.36
DS1, 313	106.86	3.0	9.6	0.0	0.34
NR, 313	91.06	1.9	5.1	0.0	0.29
P, 313	94.34	2.3	6.1	0.0	0.30
SRFA, 325	104.11	6.8	7.0	0.0	0.32
CJ11, 325	103.13	3.9	6.9	0.0	0.32
DS11, 325	105.85	4.7	8.7	0.0	0.33
DS1, 325	109.93	5.5	8.5	0.0	0.34
NR, 325	95.07	2.2	5.0	0.0	0.29
P, 325	103.30	3.9	7.3	0.0	0.32
SRFA, 337	116.23	7.7	8.4	0.0	0.34
CJ11, 337	113.34	6.5	6.9	0.0	0.34
DS11, 337	112.97	7.3	8.1	0.0	0.34
DS1, 337	118.28	8.4	8.5	0.0	0.35
NR, 337	104.17	3.7	5.3	0.0	0.31
P, 337	114.58	6.2	9.0	0.0	0.34
SRFA, 343	104.26	7.9	14.4	0.0	0.30
CJ11, 343	119.91	3.7	21.4	0.0	0.35
DS11, 343	110.61	2.9	15.0	0.0	0.32
DS1, 343	113.69	3.8	17.6	0.0	0.33
NR, 343	118.93	3.5	23.9	0.0	0.35
P, 343	96.86	1.8	11.6	0.0	0.28
SRFA, 357	102.41	8.4	6.5	0.0	0.29
CJ11, 357	115.79	4.3	11.2	0.0	0.32
DS11, 357	106.86	3.6	6.9	0.0	0.30
DS1, 357	112.19	3.9	10.0	0.0	0.31
NR, 357	109.76	4.5	9.3	0.0	0.31
P, 357	98.61	2.3	4.7	0.0	0.28

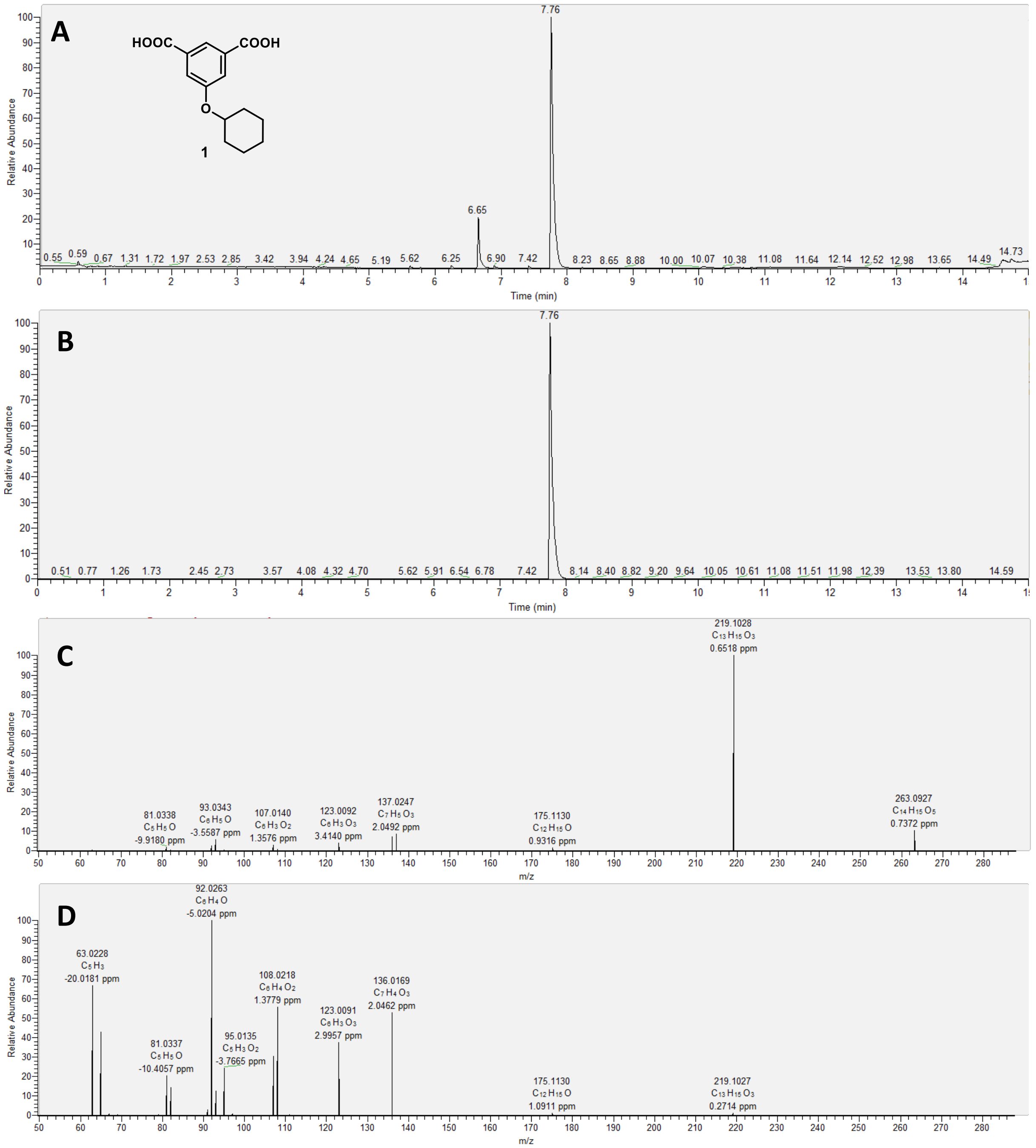

Figure S39: *TIC (A), XIC at 263.0927 m/z (B), HCD35V at 263 nominal mass (C), and HCD75V at 263 nominal mass (D) for dicarboxylic acid **1**.*

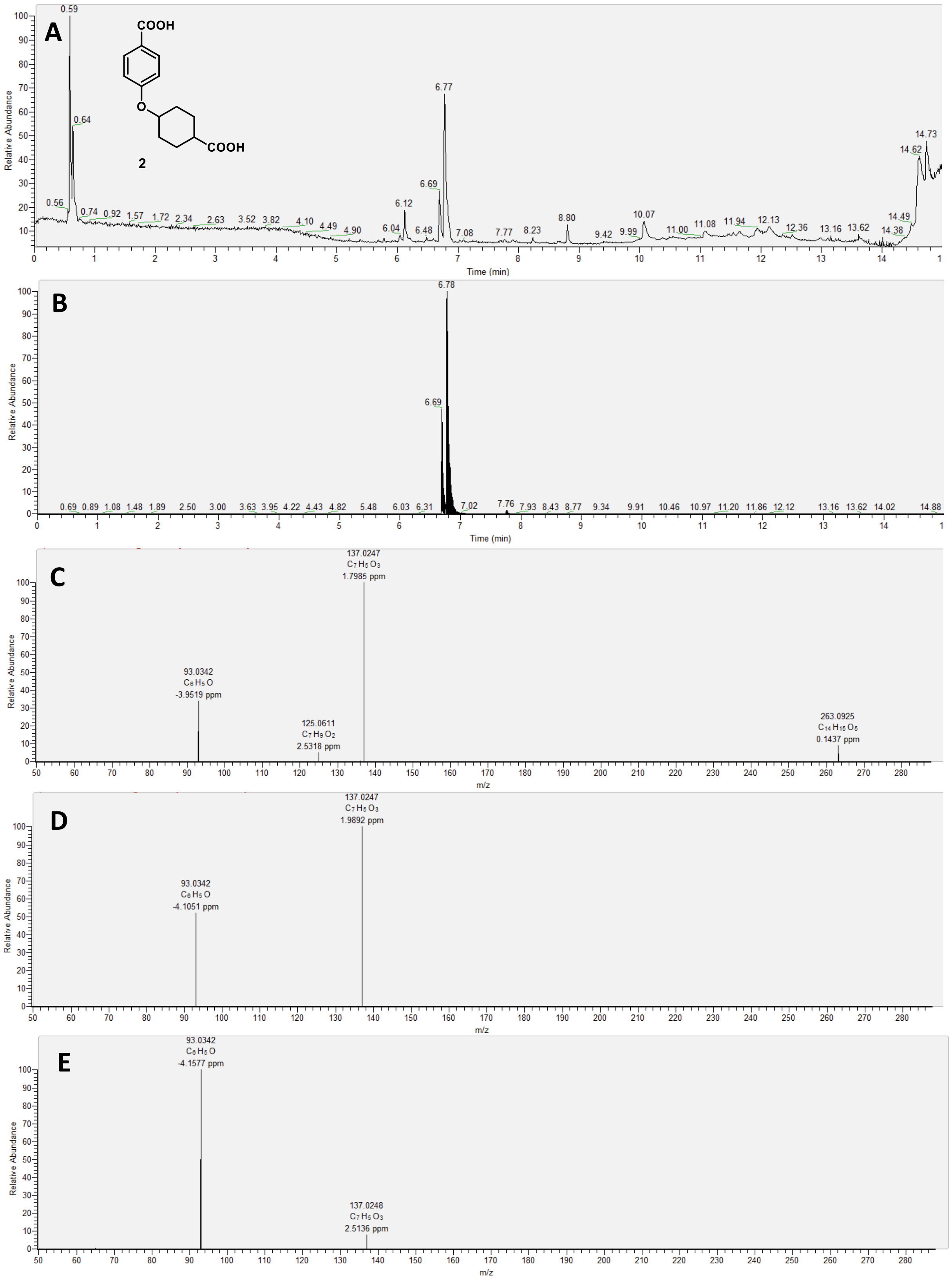
A
COOH
O
COOH
2
Relative Abundance
Time (min)
0.59
0.64
0.56
6.77
6.69
6.12
14.73
14.62
14.49
10.07
8.80
12.13
B
6.78
6.69
7.02
7.76
Relative Abundance
Time (min)
C
137.0247
C7 H5 O3
1.7985 ppm
93.0342
C6 H5 O
-3.9519 ppm
125.0611
C7 H9 O2
2.5318 ppm
263.0925
C14 H15 O5
0.1437 ppm
Relative Abundance
m/z
D
137.0247
C7 H5 O3
1.9892 ppm
93.0342
C6 H5 O
-4.1051 ppm
Relative Abundance
m/z
E
93.0342
C6 H5 O
-4.1577 ppm
137.0248
C7 H5 O3
2.5136 ppm
Relative Abundance
m/z

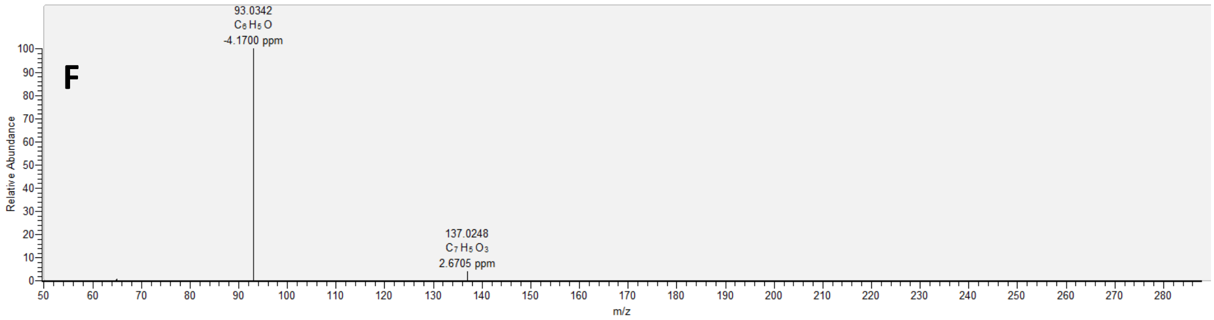

Figure S40: *TIC (A), XIC at 263.0927 m/z (B), HCD35V at 263 nominal mass for isomer 1 (6.69 min, C), HCD35V at 263 nominal mass for isomer 2 (6.78 min, D)), HCD75V at 263 nominal mass for isomer 1 (6.69 min, E), and HCD75V at 263 nominal mass for isomer 2 (6.78 min, F), dicarboxylic acid* ***2.***

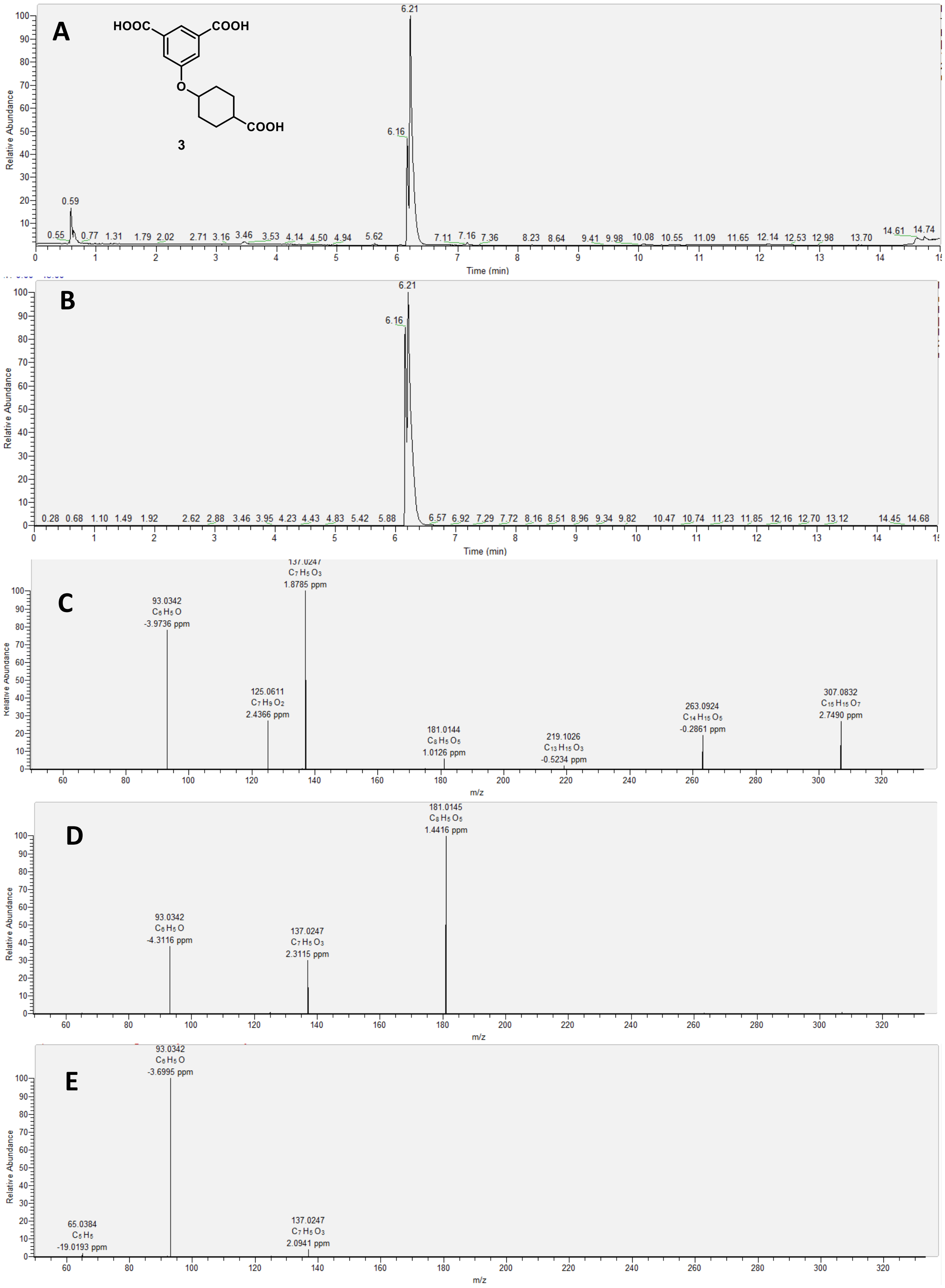

A
HOOC
COOH
O
COOH
3
6.21
6.16
0.59
Relative Abundance
Time (min)
B
6.21
6.16
Relative Abundance
Time (min)
C
93.0342
C6 H5 O
-3.9736 ppm
137.0247
C7 H5 O3
1.8785 ppm
125.0611
C7 H9 O2
2.4366 ppm
181.0144
C8 H5 O5
1.0126 ppm
219.1026
C13 H15 O3
-0.5234 ppm
263.0924
C14 H15 O5
-0.2861 ppm
307.0832
C15 H15 O7
2.7490 ppm
Relative Abundance
m/z
D
181.0145
C8 H5 O5
1.4416 ppm
93.0342
C6 H5 O
-4.3116 ppm
137.0247
C7 H5 O3
2.3115 ppm
Relative Abundance
m/z
E
93.0342
C6 H5 O
-3.6995 ppm
65.0384
C5 H5
-19.0193 ppm
137.0247
C7 H5 O3
2.0941 ppm
Relative Abundance
m/z

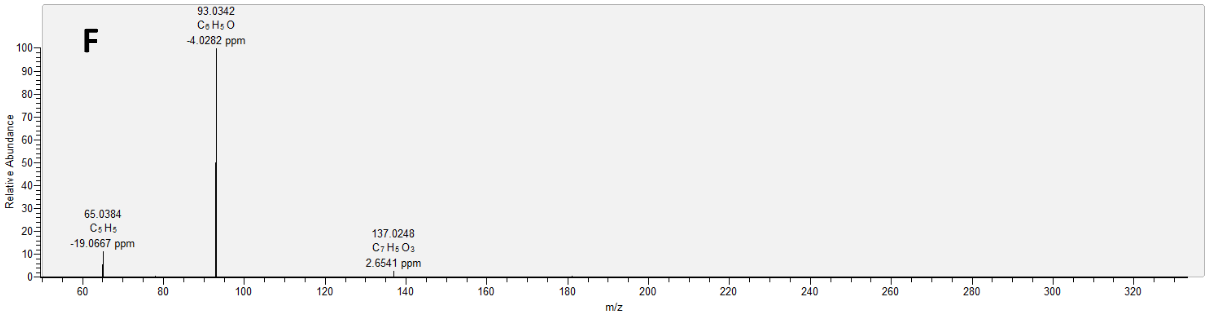

Figure S41: *TIC (A), XIC at 307.0832 m/z (B), HCD35V at 307 nominal mass for isomer 1 (6.16 min, C), HCD35V at 307 nominal mass for isomer 2 (6.21 min, D)), HCD75V at 307 nominal mass for isomer 1 (6.16 min, E), and HCD75V at 263 nominal mass for isomer 2 (6.21 min, F), for tricarboxylic acid* ***3****.*

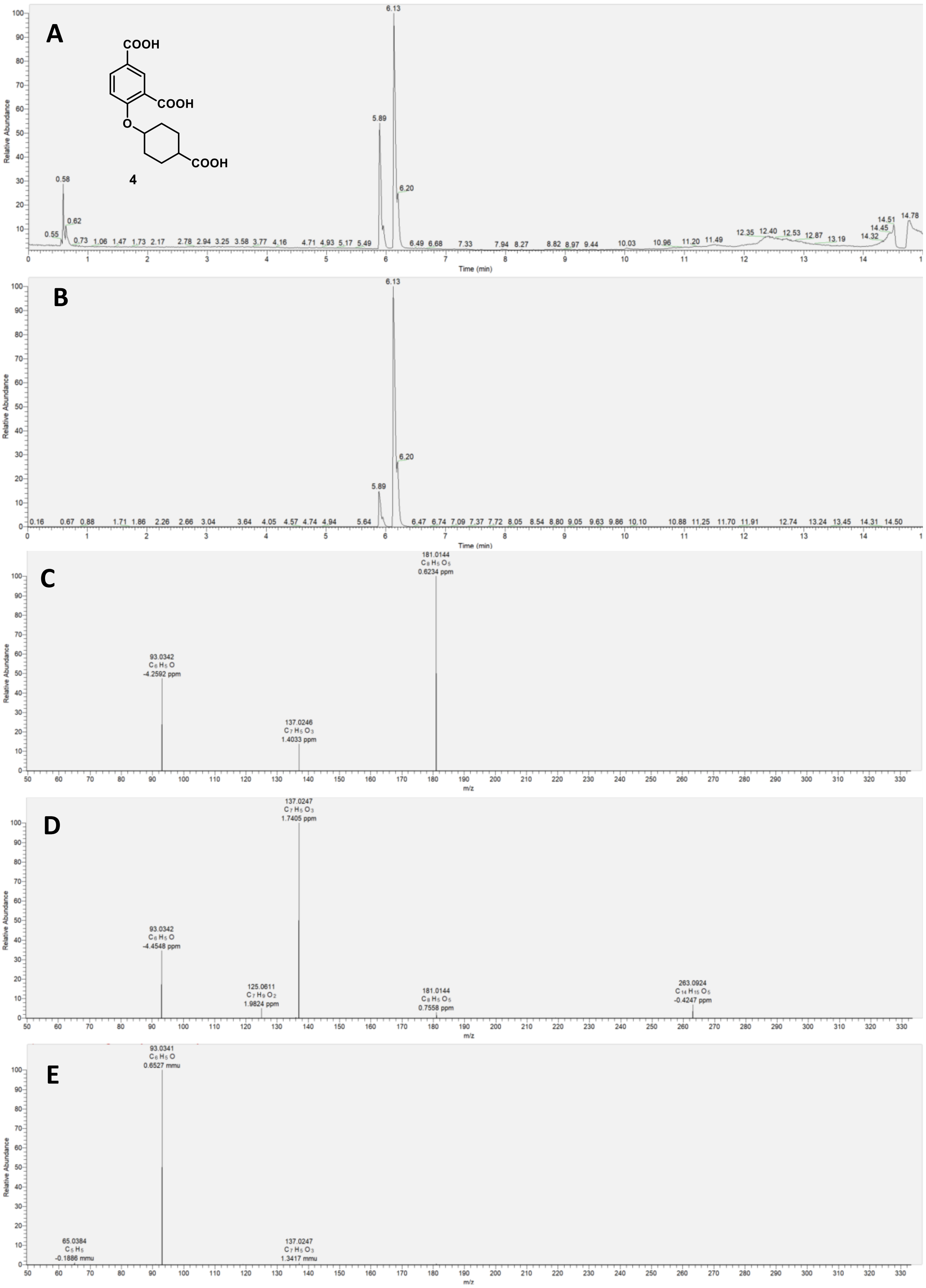
A
B
C
D
E
COOH
COOH
O
COOH
4
Relative Abundance
Time (min)
m/z
6.13
5.89
6.20
0.58
0.62
14.78
14.51
181.0144
C8 H5 O5
0.6234 ppm
93.0342
C6 H5 O
-4.2592 ppm
137.0246
C7 H5 O3
1.4033 ppm
137.0247
C7 H5 O3
1.7405 ppm
93.0342
C6 H5 O
-4.4548 ppm
125.0611
C7 H9 O2
1.9824 ppm
181.0144
C8 H5 O5
0.7558 ppm
263.0924
C14 H15 O5
-0.4247 ppm
93.0341
C6 H5 O
0.6527 mmu
65.0384
C5 H5
-0.1886 mmu
137.0247
C7 H5 O3
1.3417 mmu

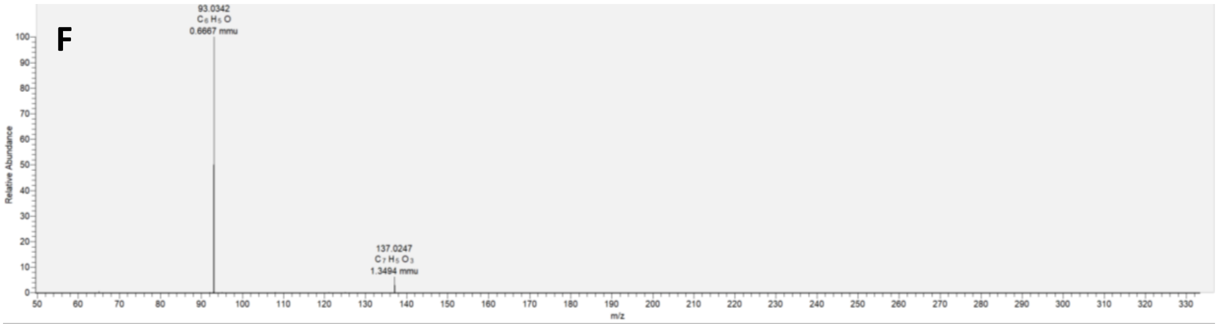

Figure S42: *TIC (A), XIC at 307.0832 m/z (B), HCD35V at 307 nominal mass for isomer 1 (5.89 min, C)), HCD35V at 307 nominal mass for isomer 2 (6.13 min D)), HCD75V at 307 nominal mass for isomer 1 (5.89 min E), and HCD75V at 307 nominal mass for isomer 2 (6.13 min, F), for tricarboxylic acid* ***4****.*

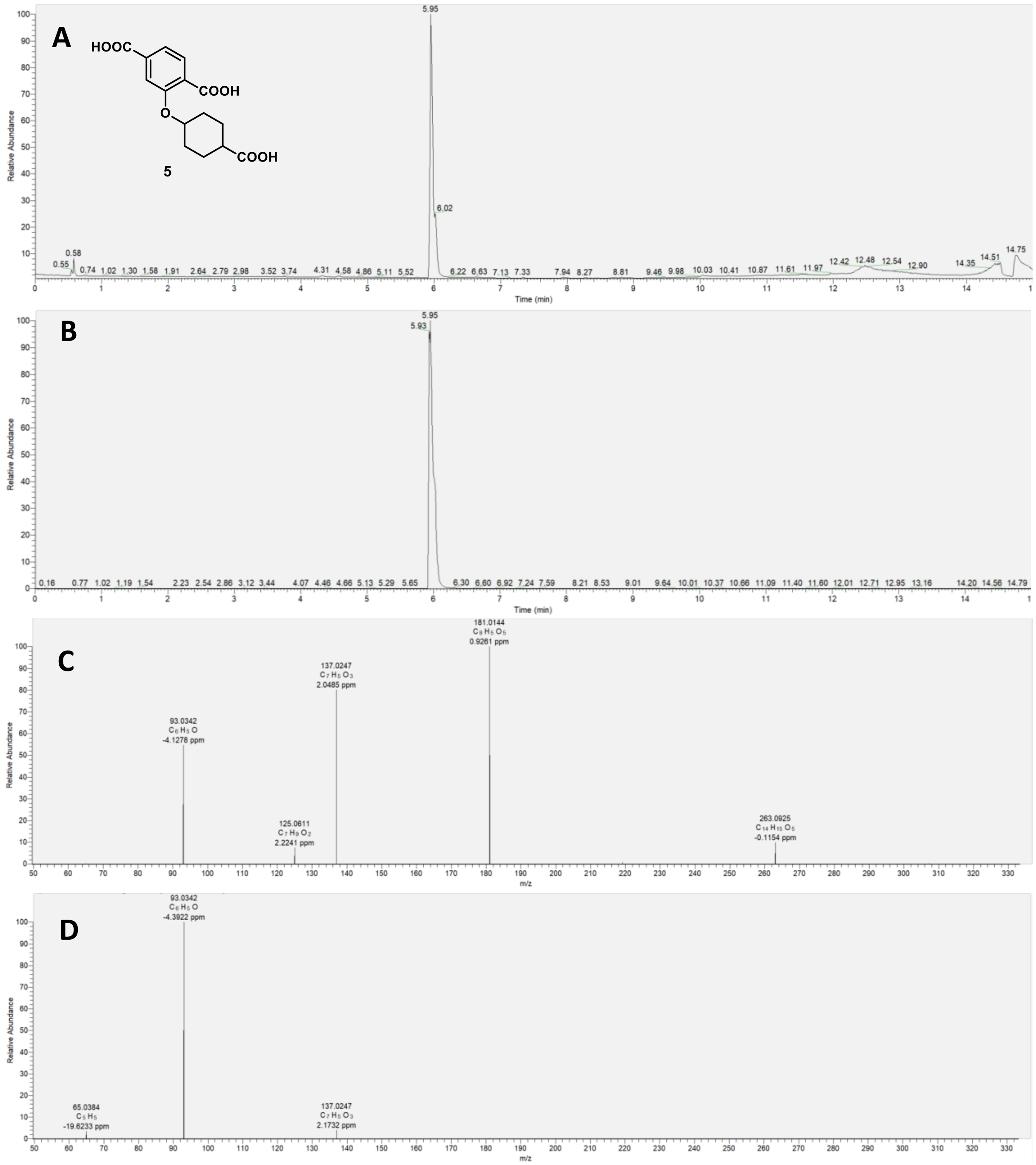

Figure S43: *TIC (A), XIC at 307.0832 m/z (B), HCD35V at 307 nominal mass (C), and HCD75V at 307 nominal mass (D) for tricarboxylic acid **5**.*

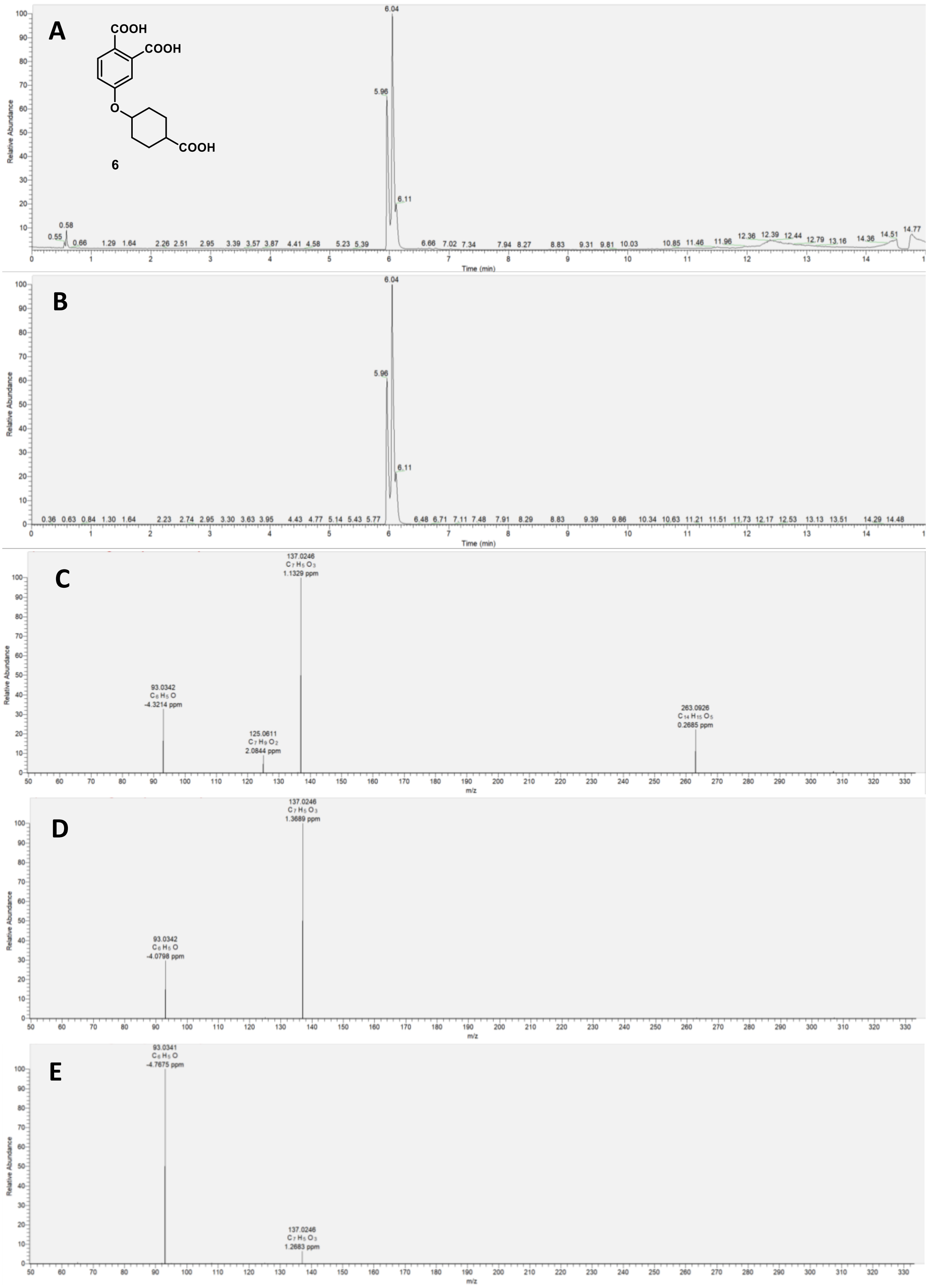
A
COOH
COOH
O
COOH
6
6.04
5.96
6.11
Relative Abundance
Time (min)
B
6.04
5.96
6.11
Relative Abundance
Time (min)
C
137.0246
C7 H5 O3
1.1329 ppm
93.0342
C6 H5 O
-4.3214 ppm
125.0611
C7 H9 O2
2.0844 ppm
263.0926
C14 H15 O5
0.2685 ppm
Relative Abundance
m/z
D
137.0246
C7 H5 O3
1.3689 ppm
93.0342
C6 H5 O
-4.0798 ppm
Relative Abundance
m/z
E
93.0341
C6 H5 O
-4.7675 ppm
137.0246
C7 H5 O3
1.2683 ppm
Relative Abundance
m/z

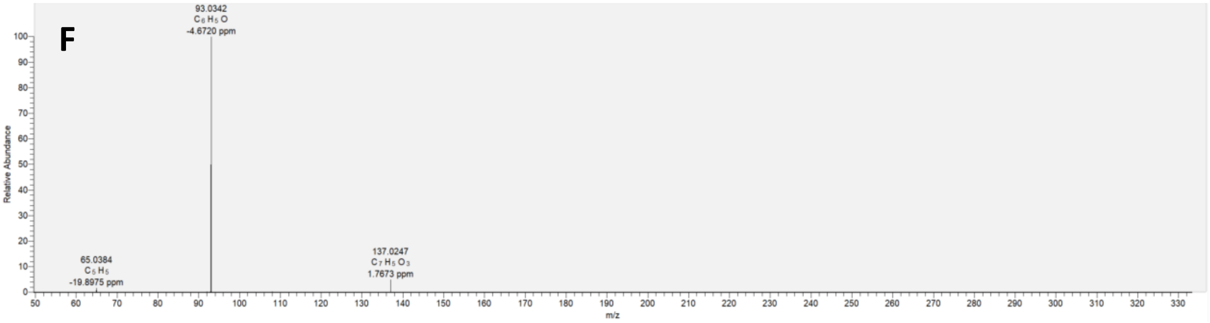

Figure S44: *TIC (A), XIC at 307.0832 m/z (B), HCD35V at 307 nominal mass for isomer 1 (5.96 min, C), HCD35V at 307 nominal mass for isomer 2 (6.04 min D)), HCD75V at 307 nominal mass for isomer 1 (5.96 min E), and HCD75V at 307 nominal mass for isomer 2 (6.04 min, F), for tricarboxylic acid* ***6****.*

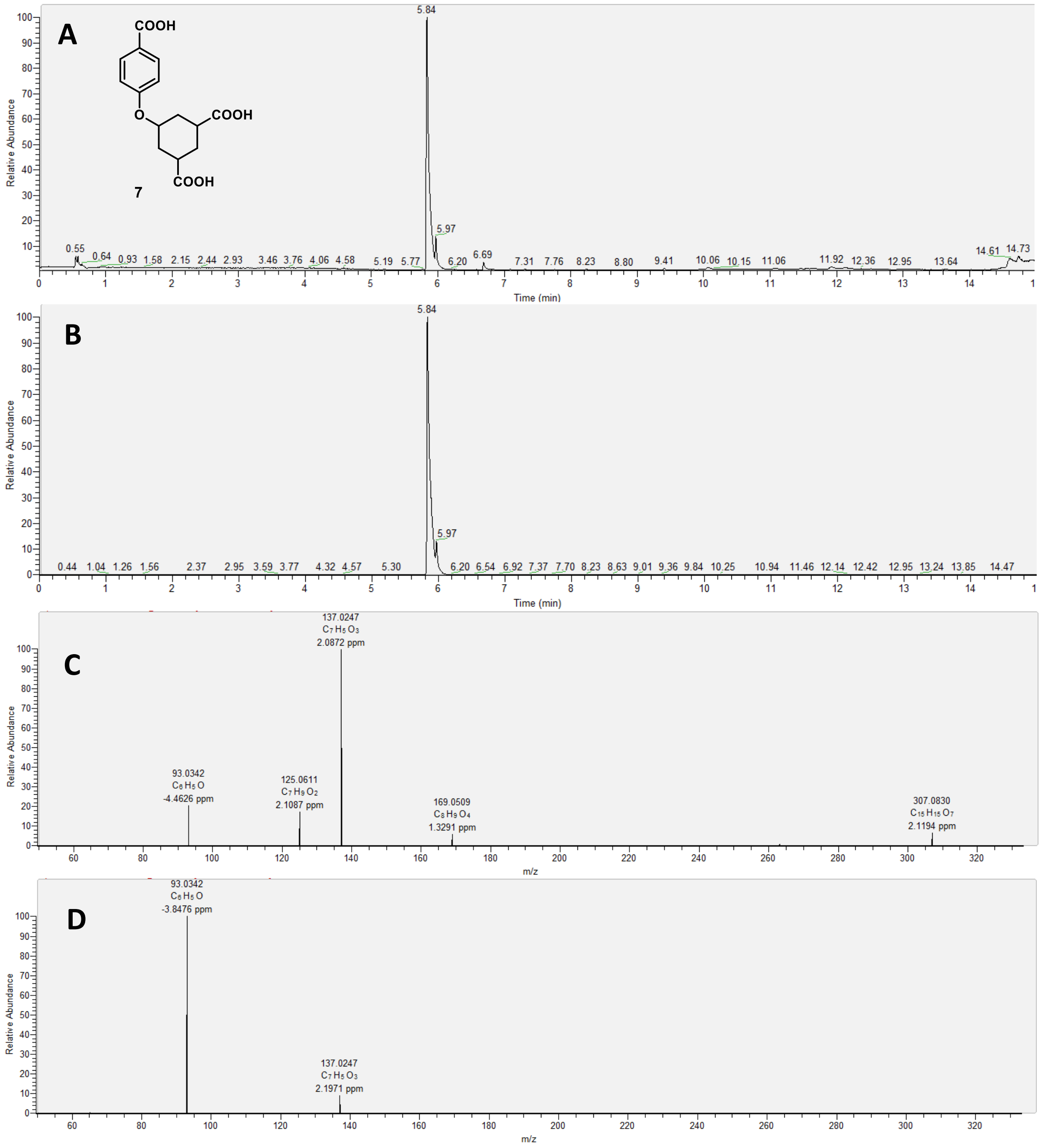

Figure S45: *TIC (A), XIC at 307.0832 m/z (B), HCD35V at 307 nominal mass (C), and HCD75V at 307 nominal mass (D) for tricarboxylic acid **7**.*

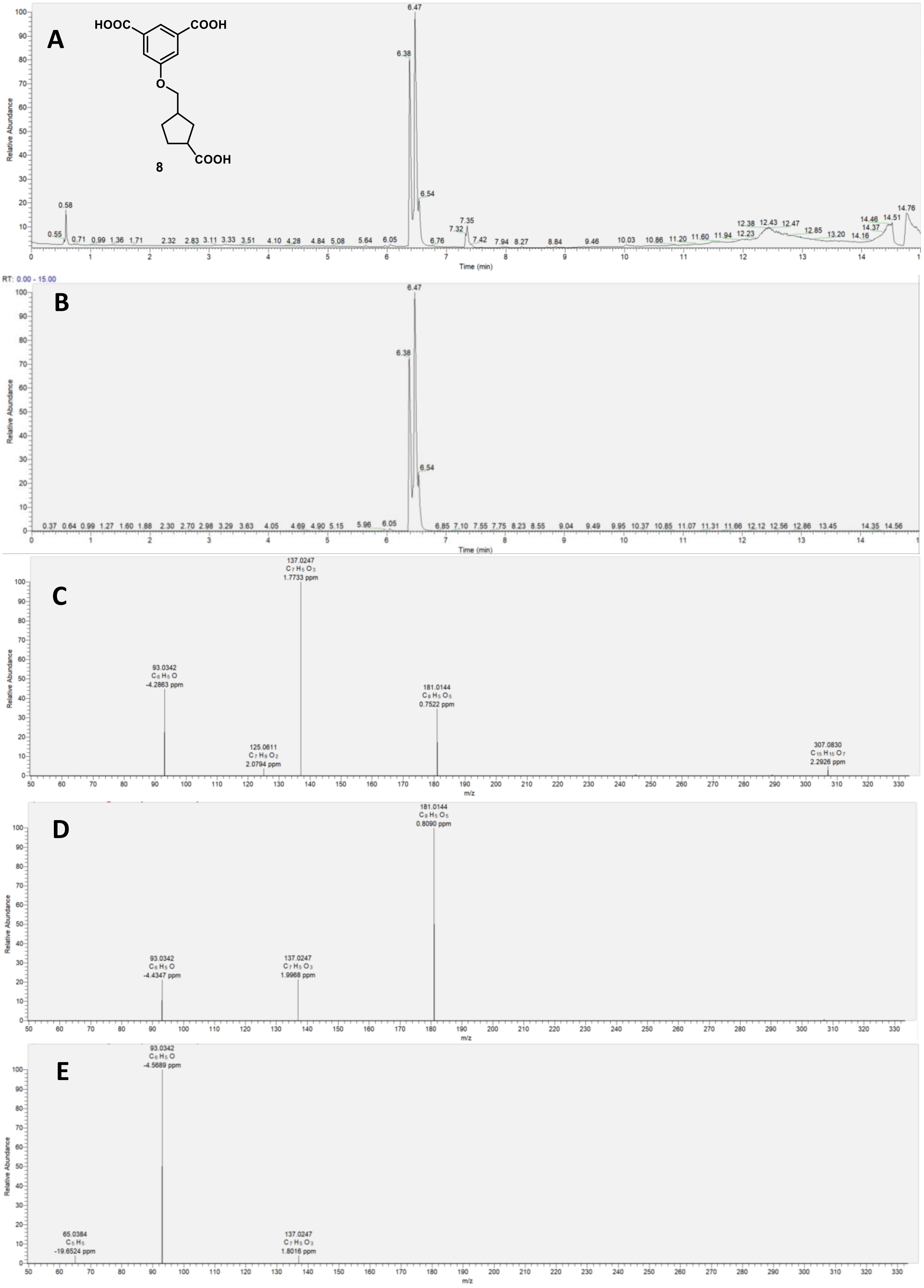
A
HOOC
COOH
O
COOH
8
Relative Abundance
Time (min)
B
RT: 0.00 - 15.00
C
137.0247
C7 H5 O3
1.7733 ppm
93.0342
C6 H5 O
-4.2863 ppm
181.0144
C8 H5 O5
0.7522 ppm
125.0611
C7 H9 O2
2.0794 ppm
307.0830
C15 H15 O7
2.2926 ppm
m/z
D
181.0144
C8 H5 O5
0.8090 ppm
93.0342
C6 H5 O
-4.4347 ppm
137.0247
C7 H5 O3
1.9968 ppm
E
93.0342
C6 H5 O
-4.5689 ppm
65.0384
C5 H5
-19.6524 ppm
137.0247
C7 H5 O3
1.8016 ppm

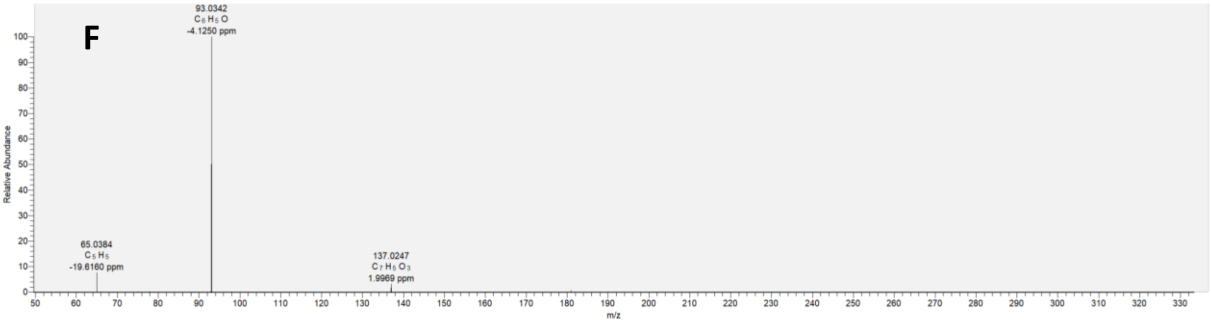

Figure S46: *TIC (A), XIC at 307.0830 m/z (B), HCD35V at 307 nominal mass for isomer 1 (6.38 min, C), HCD35V at 307 nominal mass for isomer 2 (6.47 min D)), HCD75V at 307 nominal mass for isomer 1 (6.38 min E), and HCD75V at 307 nominal mass for isomer 2 (6.47 min, F), for tricarboxylic acid* ***8****.*

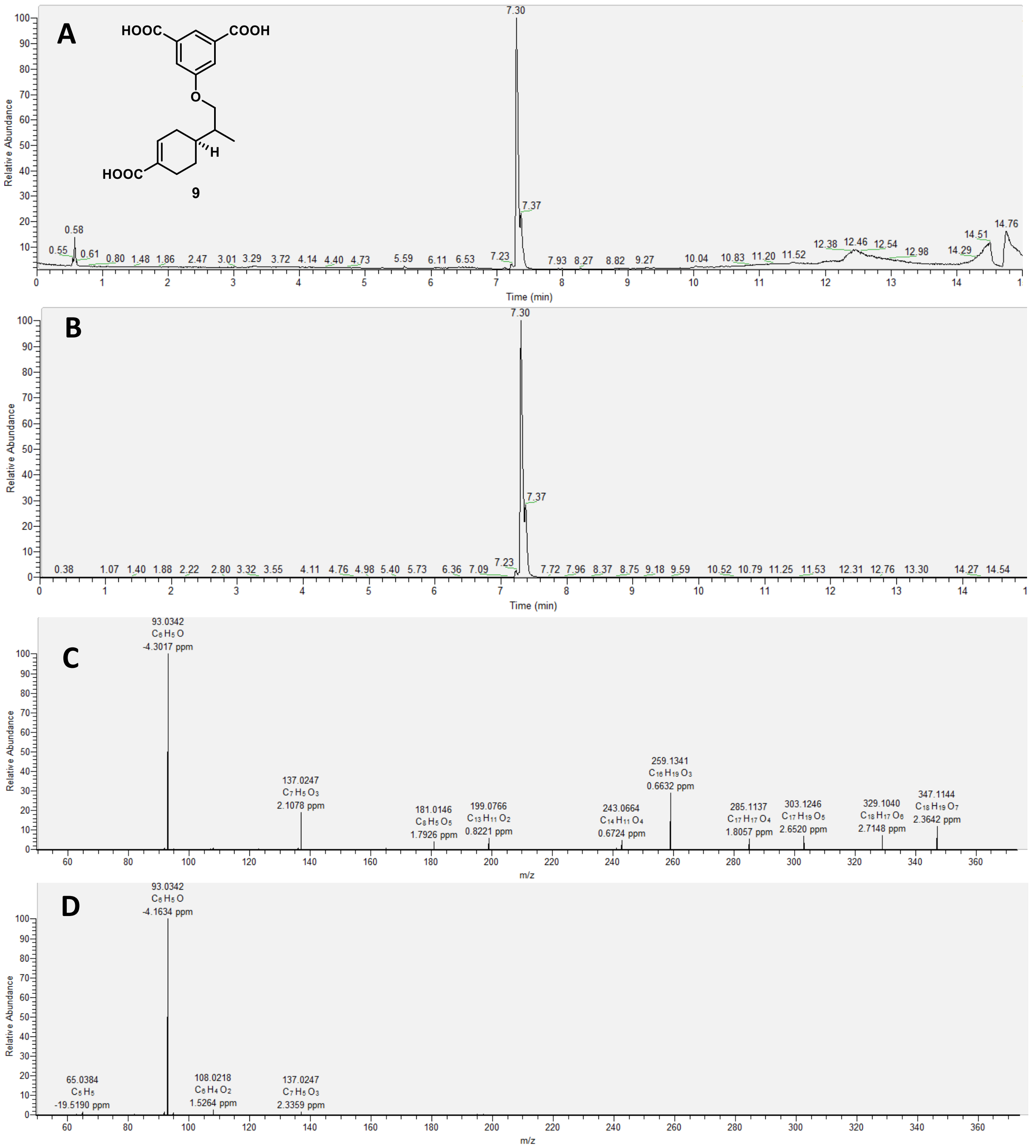

Figure S47: *TIC (A), XIC at 347.1144 m/z (B), HCD35V at 347 nominal mass (C), and HCD75V at 347 nominal mass (D) for tricarboxylic acid* ***9****.*

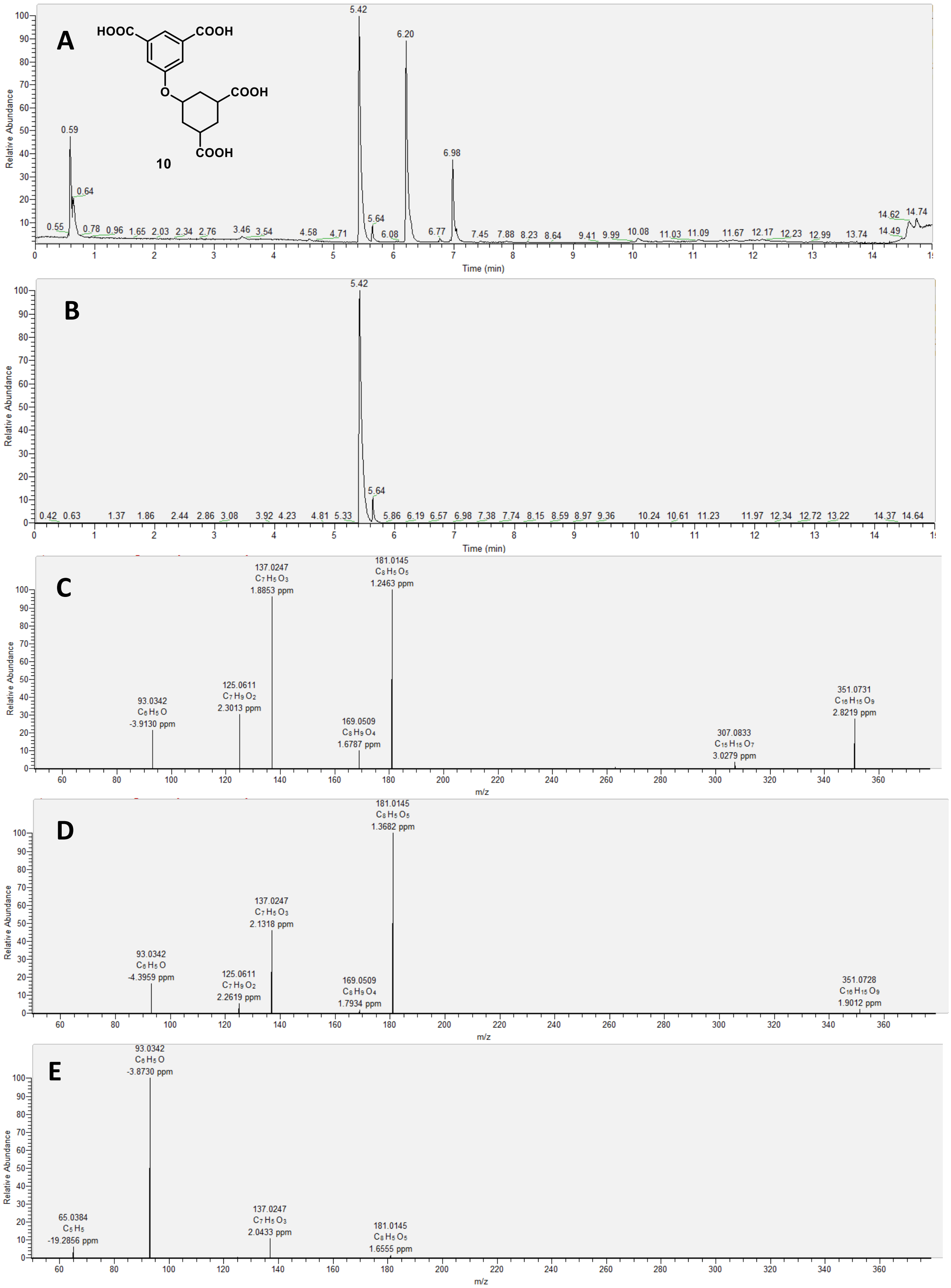
A
HOOC
COOH
O
COOH
COOH
10
5.42
6.20
6.98
0.59
0.64
5.64
Time (min)
Relative Abundance
B
5.42
5.64
Time (min)
C
137.0247
C7 H5 O3
1.8853 ppm
181.0145
C8 H5 O5
1.2463 ppm
93.0342
C6 H5 O
-3.9130 ppm
125.0611
C7 H9 O2
2.3013 ppm
169.0509
C8 H9 O4
1.6787 ppm
307.0833
C15 H15 O7
3.0279 ppm
351.0731
C16 H15 O9
2.8219 ppm
m/z
D
181.0145
C8 H5 O5
1.3682 ppm
137.0247
C7 H5 O3
2.1318 ppm
93.0342
C6 H5 O
-4.3959 ppm
125.0611
C7 H9 O2
2.2619 ppm
169.0509
C8 H9 O4
1.7934 ppm
351.0728
C16 H15 O9
1.9012 ppm
m/z
E
93.0342
C6 H5 O
-3.8730 ppm
65.0384
C5 H5
-19.2856 ppm
137.0247
C7 H5 O3
2.0433 ppm
181.0145
C8 H5 O5
1.6555 ppm
m/z

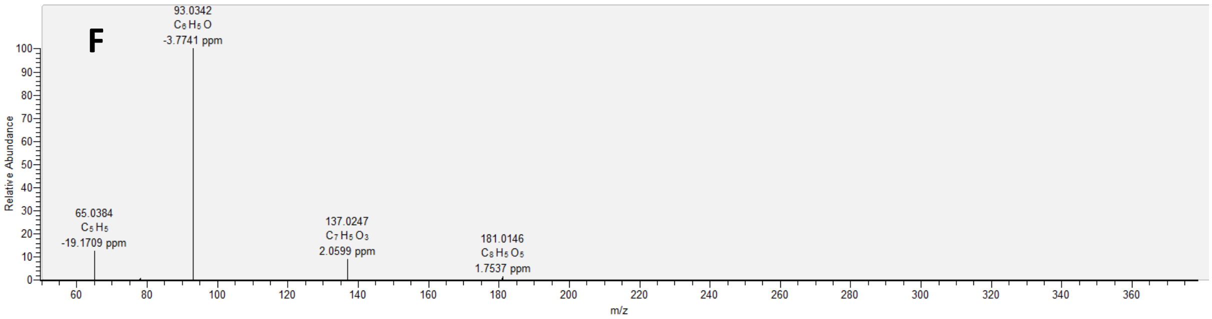

Figure S48: *TIC (A), XIC at 351.0731 m/z (B), HCD35V at 351 nominal mass for isomer 1 (5.42 min, (C)), HCD35V at 351 nominal mass for isomer 2 (5.64 min D)), HCD75V at 351 nominal mass for isomer 1 (5.42 min E), and HCD75V at 351 nominal mass for isomer 2 (5.64 min, F), for tetracarboxylic acid* ***10****.*

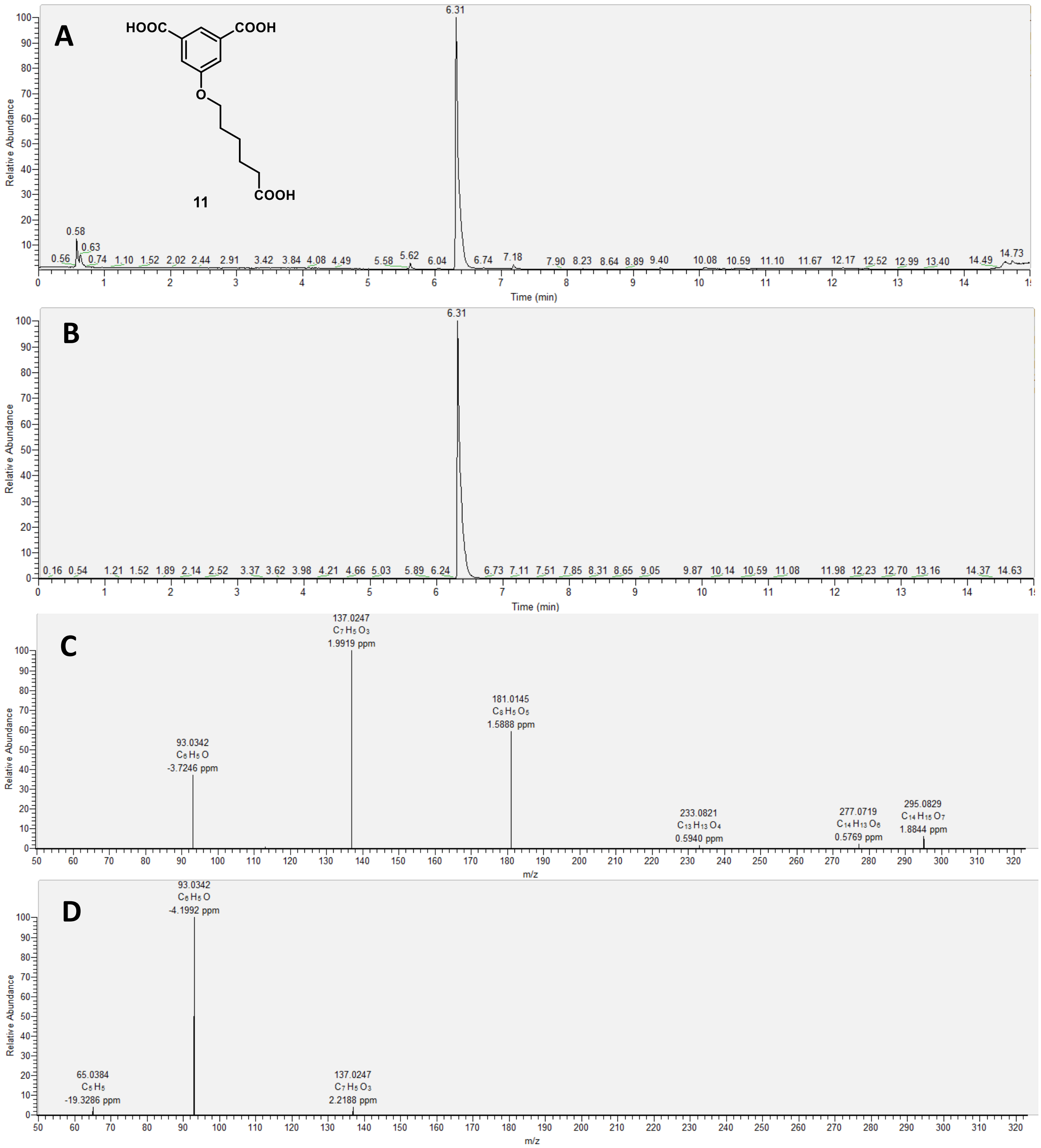

Figure S49: *TIC (A), XIC at 295.0829 m/z (B), HCD35V at 295 nominal mass (C), and HCD75V at 295 nominal mass (D) for tricarboxylic acid* ***11****.*

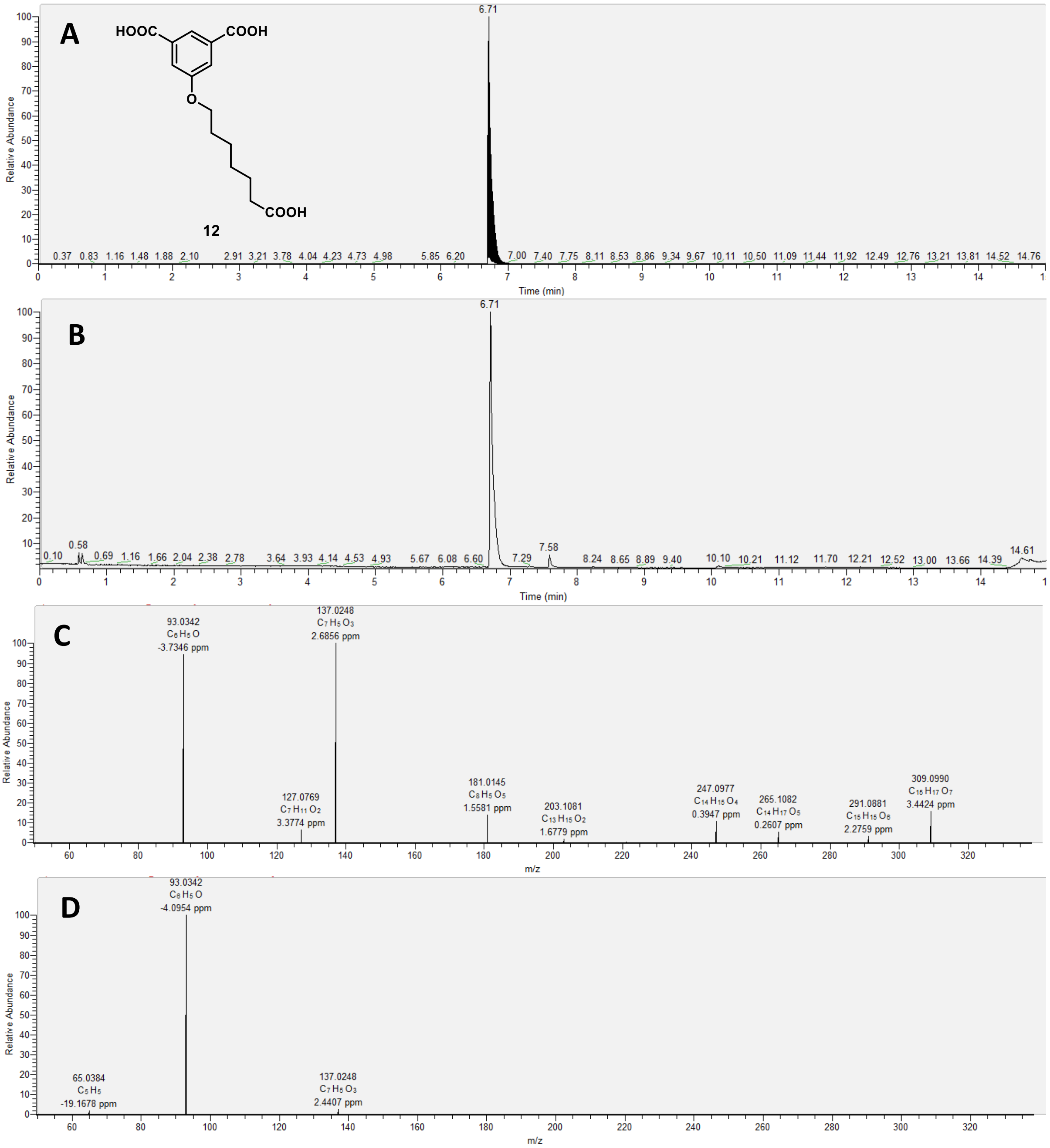

Figure S50: *TIC (A), XIC at 309.0990 m/z (B), HCD35V at 309 nominal mass (C), and HCD75V at 309 nominal mass (D) for tricarboxylic acid **12**.*

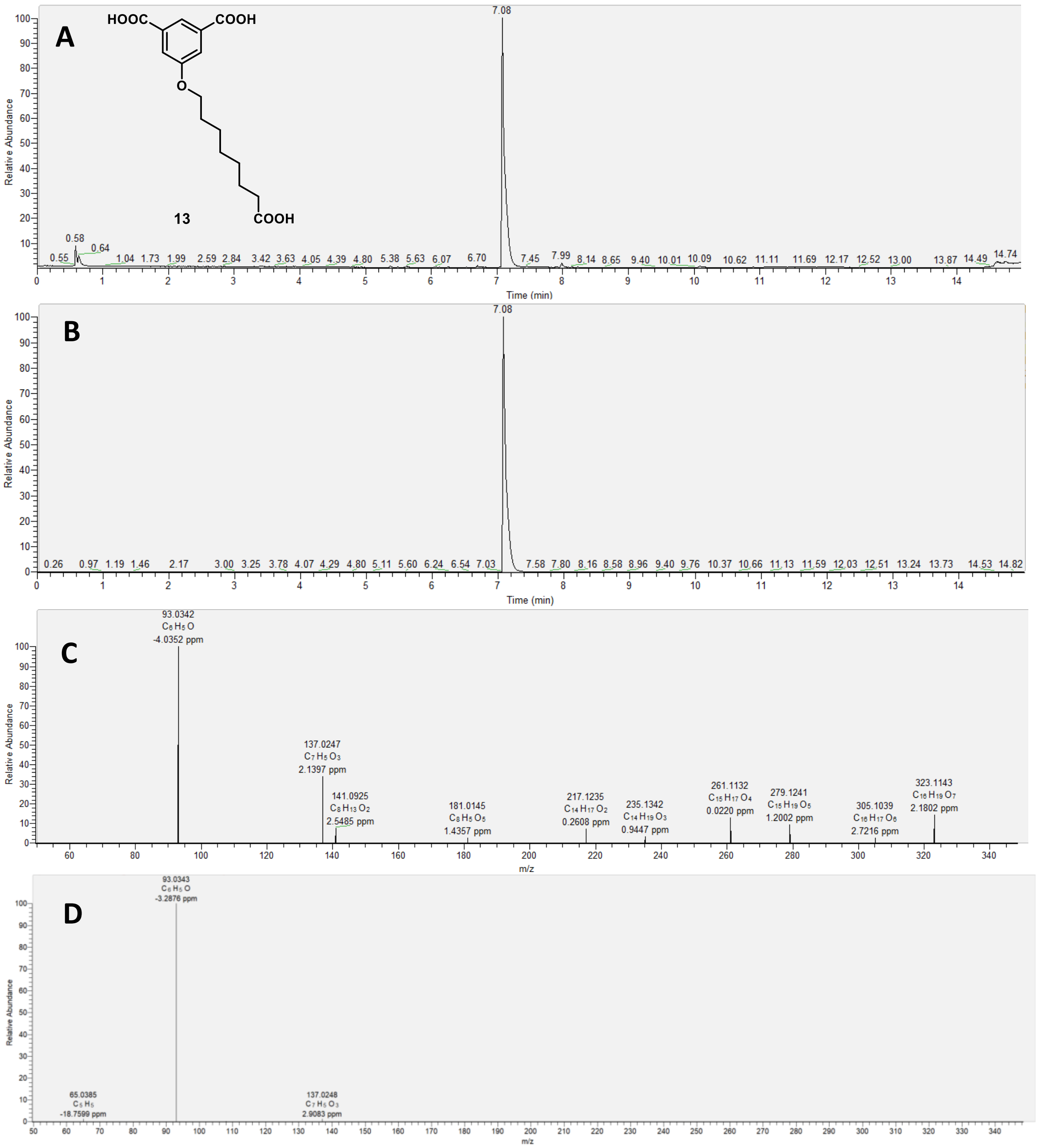

Figure S51: *TIC (A), XIC at 323.1143 m/z (B), HCD35V at 323 nominal mass (C), and HCD75V at 323 nominal mass (D) for tricarboxylic acid* ***13****.*

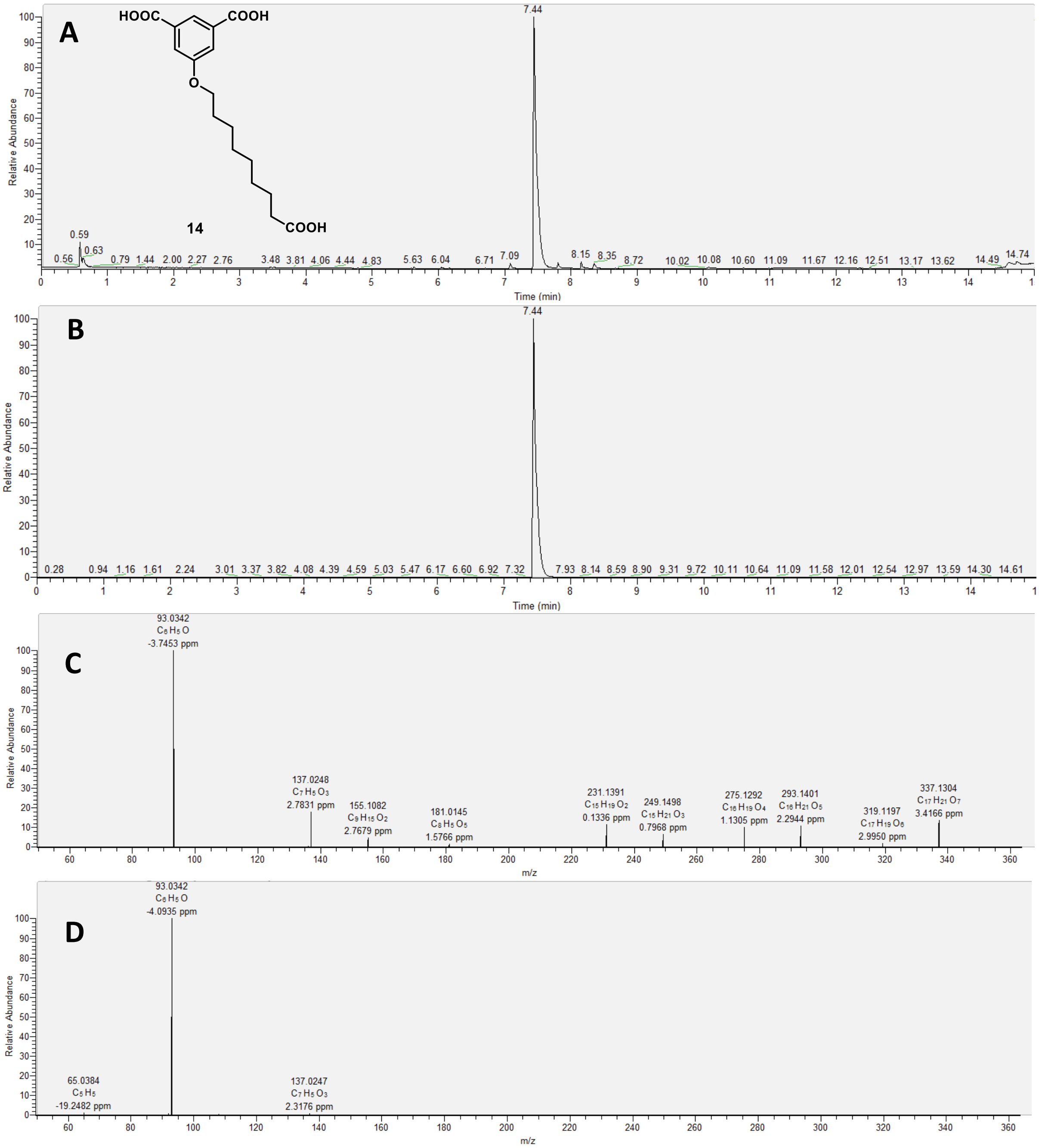

Figure S52: *TIC (A), XIC at 337.1304 m/z (B), HCD35V at 337 nominal mass (C), and HCD75V at 337 nominal mass (D) for tricarboxylic acid **14**.*

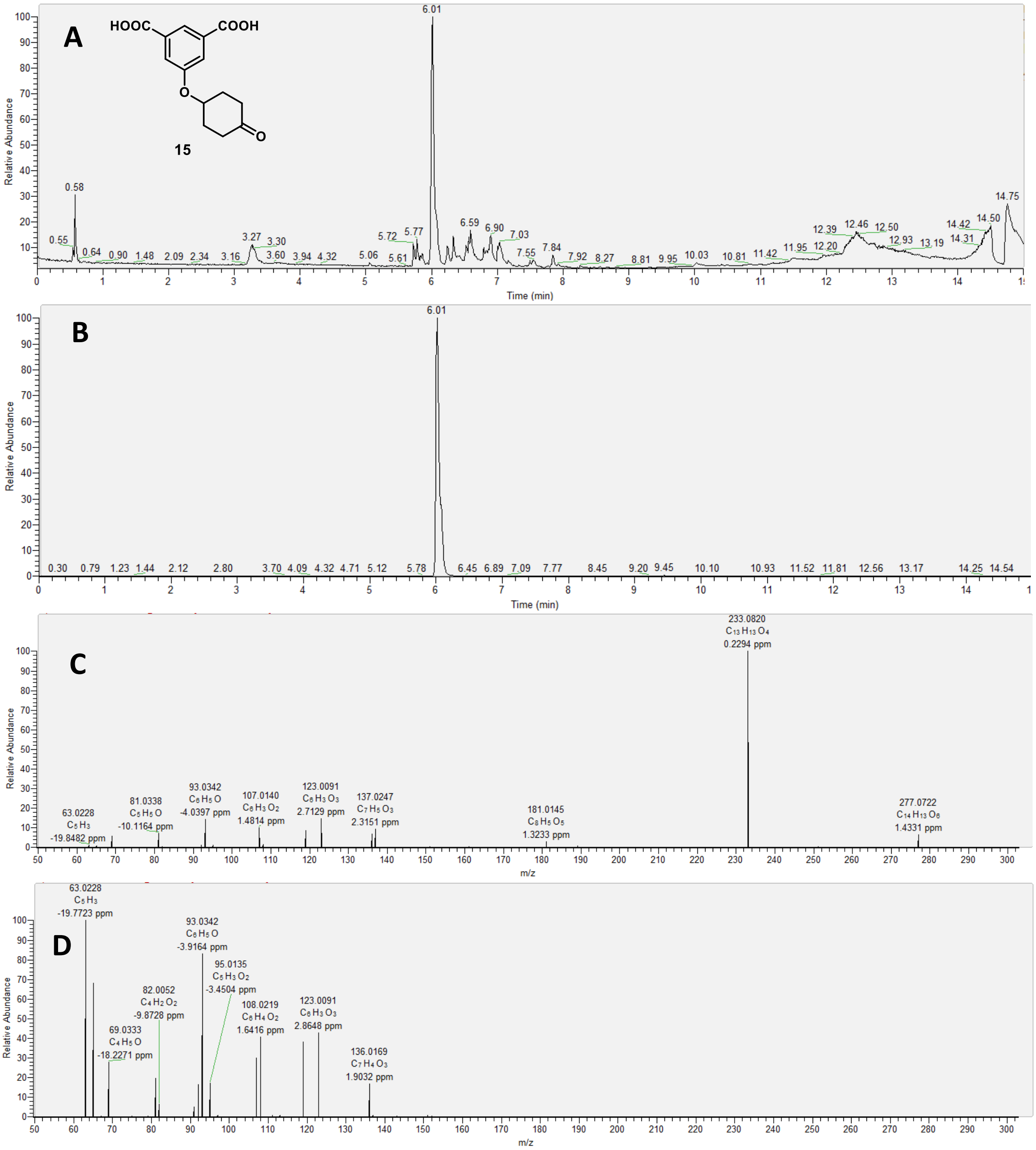

Figure S53: *TIC (A), XIC at 277.0722 m/z (B), HCD35V at 277 nominal mass (C), and HCD75V at 277 nominal mass (D) for ketone **15**.*

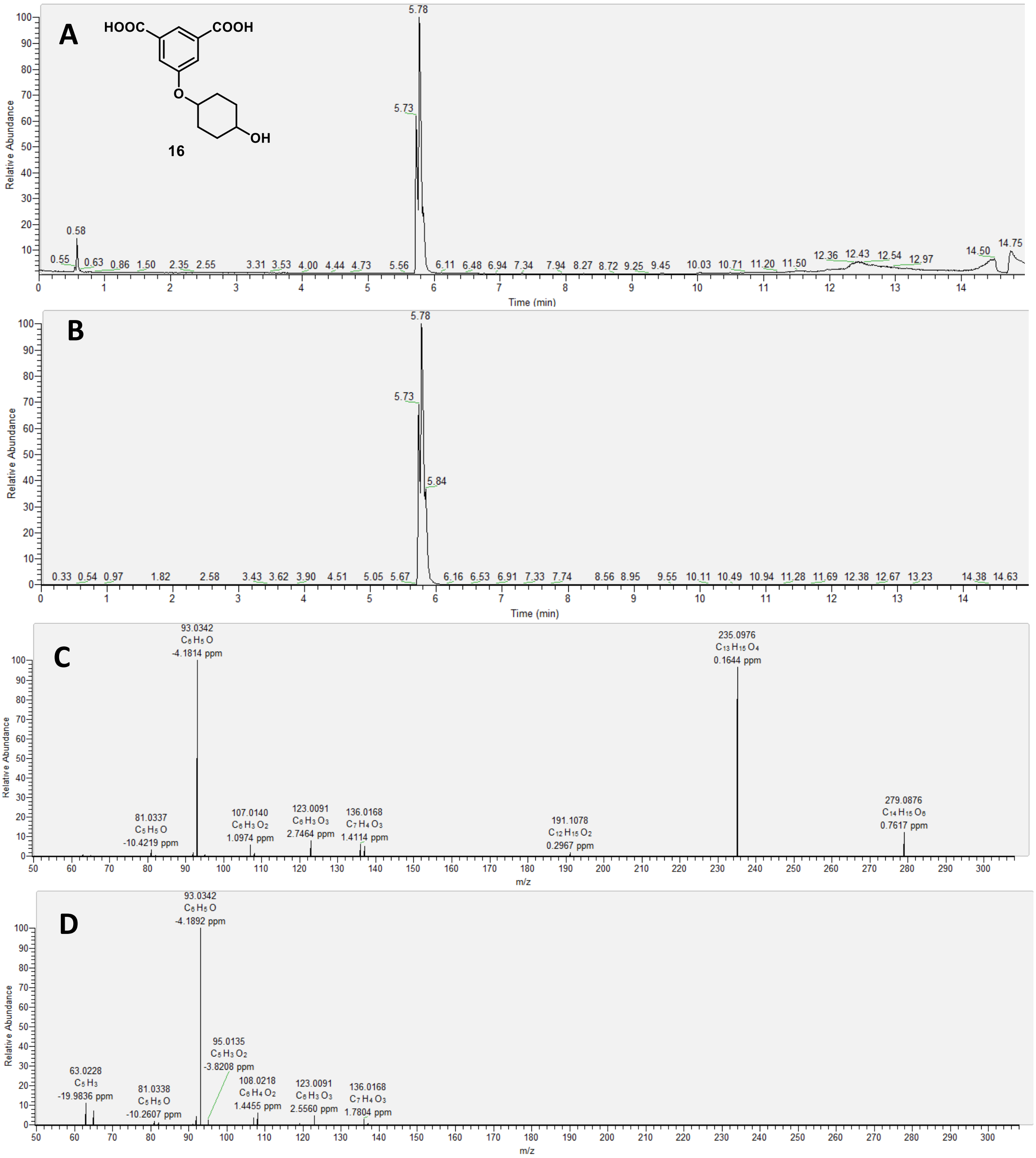

Figure S54: *TIC (A), XIC at 279.0876 m/z (B), HCD35V at 279 nominal mass (C), and HCD75V at 279 nominal mass (D) for alcohol* ***16****.*

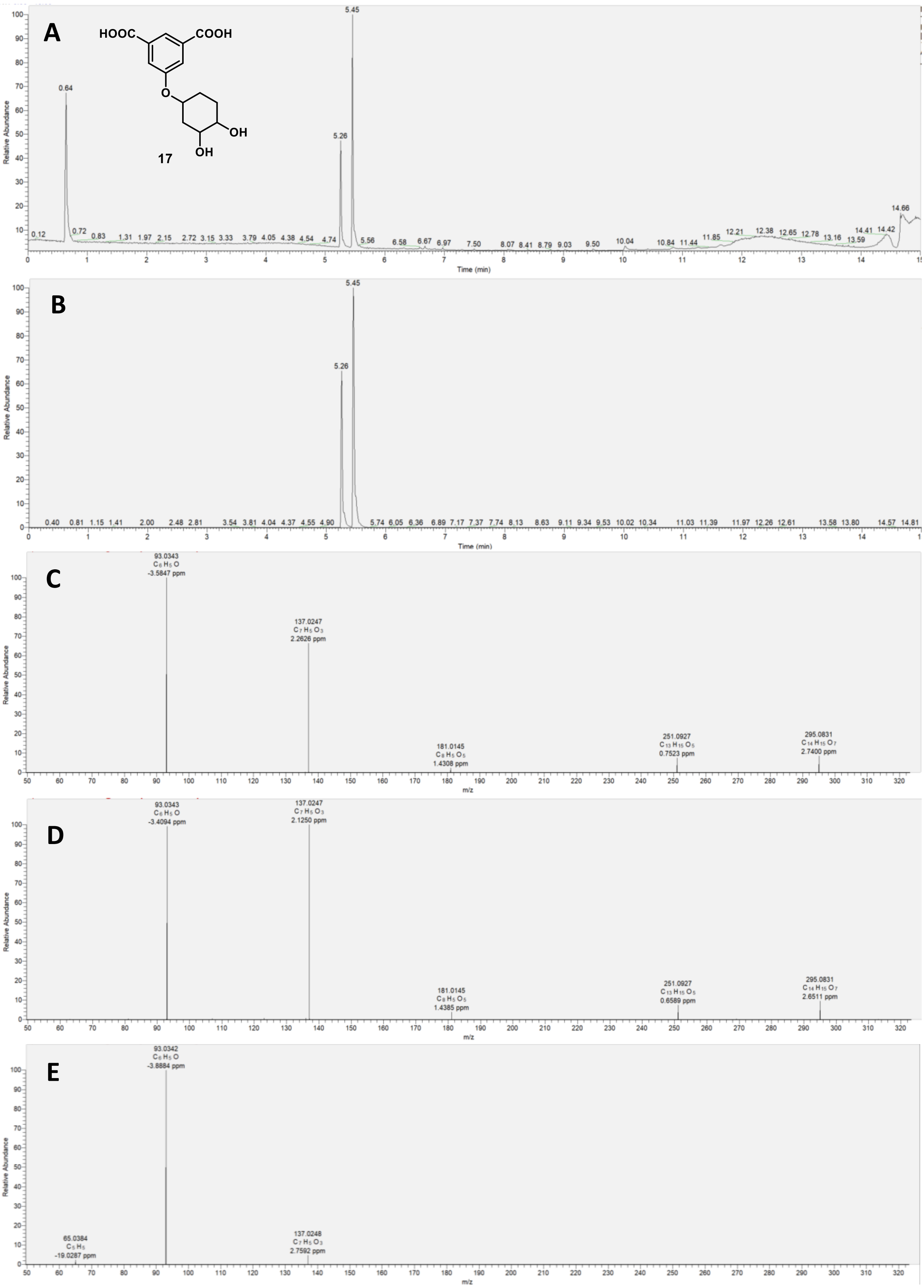
A
HOOC
COOH
O
OH
OH
17
0.64
5.26
5.45
14.66
Relative Abundance
Time (min)
B
5.26
5.45
Relative Abundance
Time (min)
C
93.0343
C6 H5 O
-3.5847 ppm
137.0247
C7 H5 O3
2.2626 ppm
181.0145
C8 H5 O5
1.4308 ppm
251.0927
C13 H15 O5
0.7523 ppm
295.0831
C14 H15 O7
2.7400 ppm
Relative Abundance
m/z
D
93.0343
C6 H5 O
-3.4094 ppm
137.0247
C7 H5 O3
2.1250 ppm
181.0145
C8 H5 O5
1.4385 ppm
251.0927
C13 H15 O5
0.6589 ppm
295.0831
C14 H15 O7
2.6511 ppm
Relative Abundance
m/z
E
93.0342
C6 H5 O
-3.8884 ppm
65.0384
C5 H5
-19.0287 ppm
137.0248
C7 H5 O3
2.7592 ppm
Relative Abundance
m/z

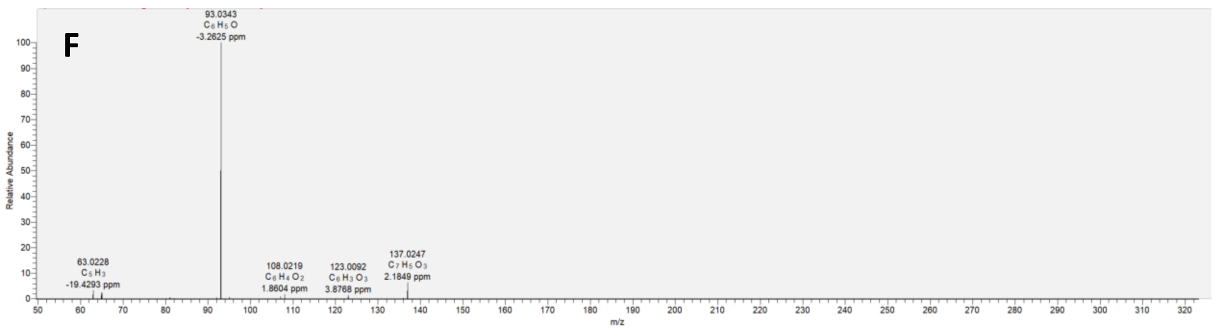

Figure S55: *TIC (A), XIC at 295.0831 m/z (B), HCD35V at 295 nominal mass for isomer 1 (5.26 min, C), HCD35V at 295 nominal mass for isomer 2 (5.45 min D)), HCD75V at 295 nominal mass for isomer 1 (5.26 min E), and HCD75V at 295 nominal mass for isomer 2 (5.45 min, F), for diol* ***17****.*

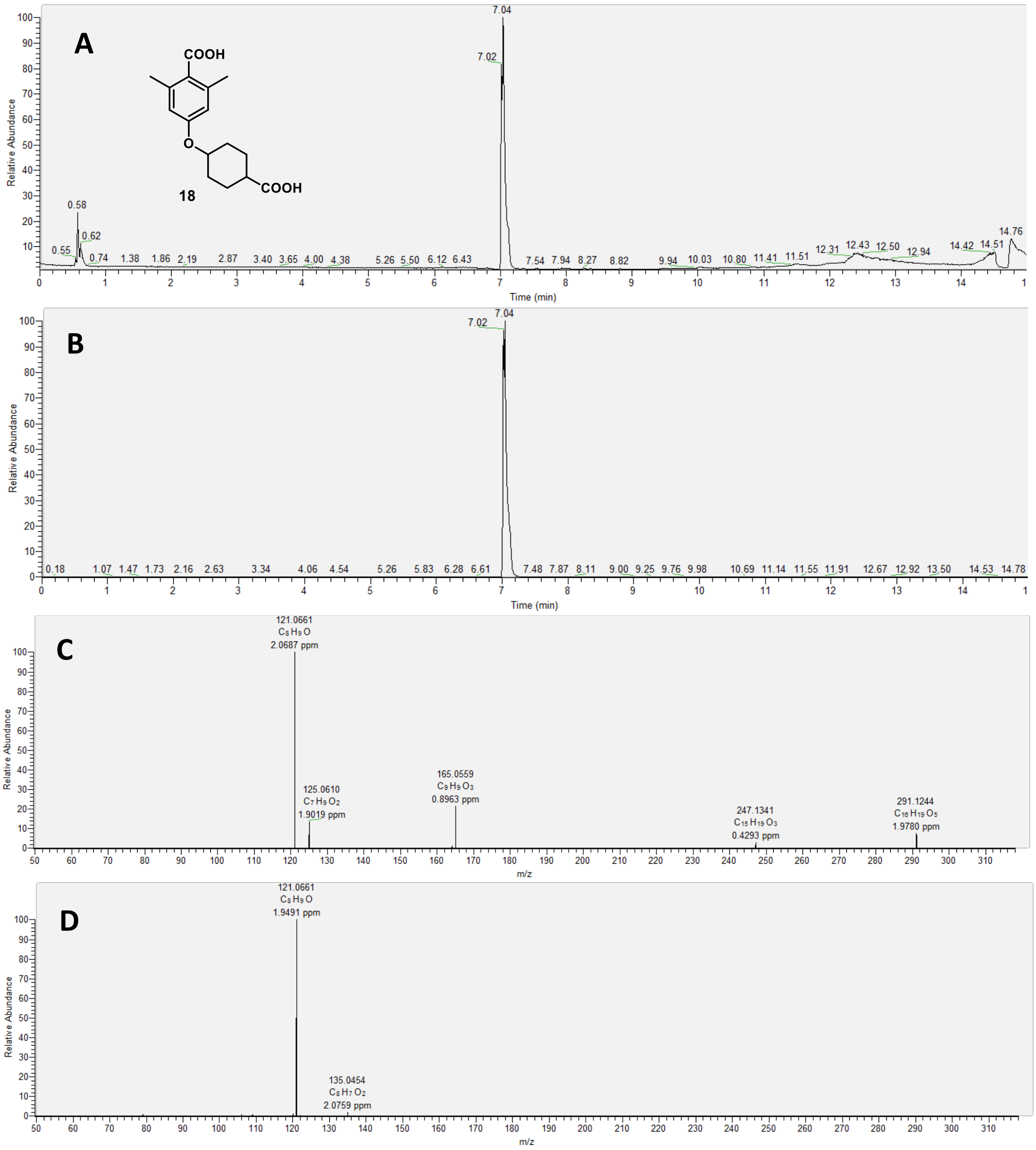

Figure S56: *TIC (A), XIC at 291.1244 m/z (B), HCD35V at 291 nominal mass (C), and HCD75V at 291 nominal mass (D) for dicarboxylic acid* ***18****.*

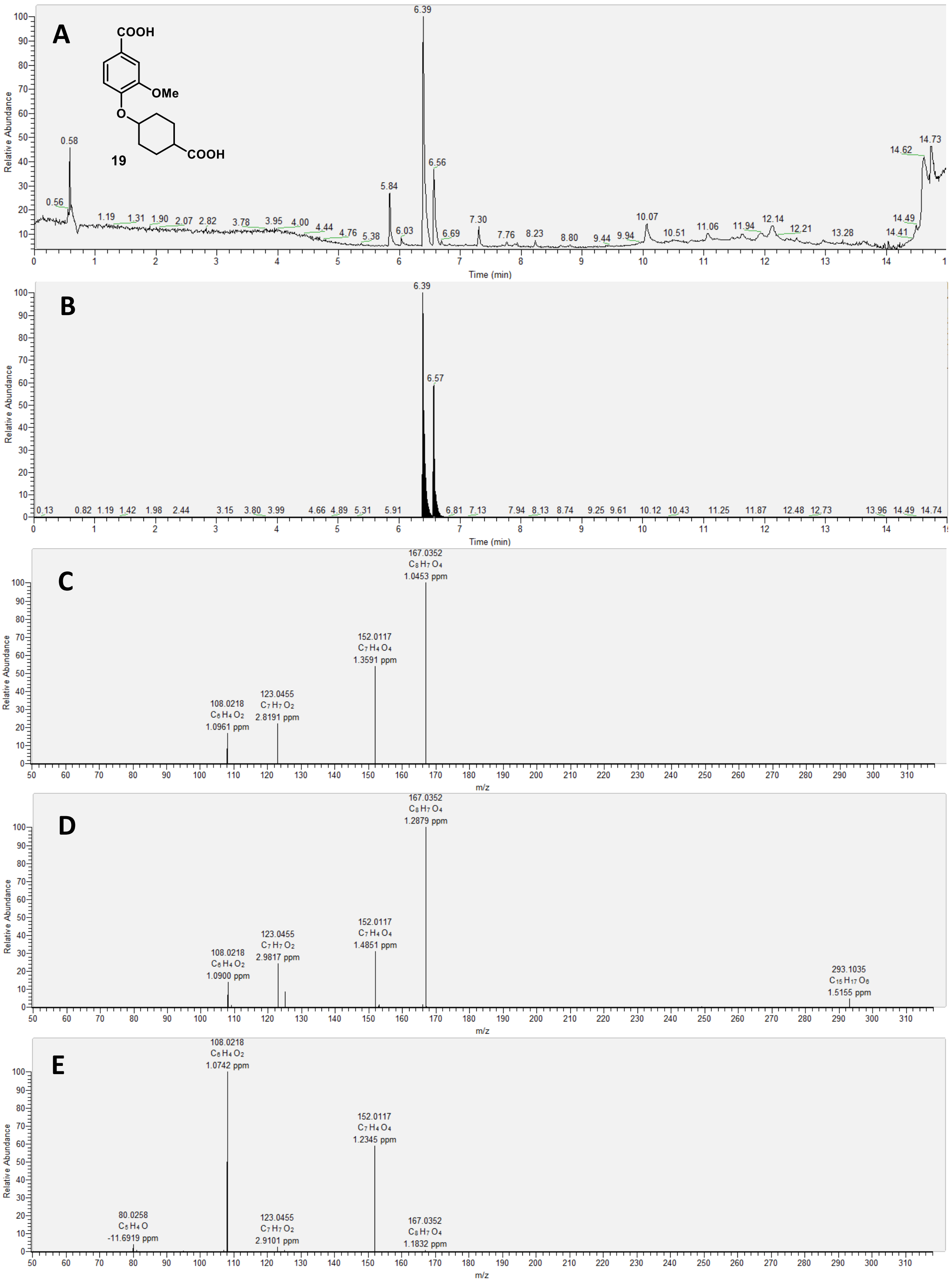
A
COOH
OMe
O
COOH
19
Relative Abundance
Time (min)
0.56
0.58
1.19
1.31
1.90
2.07
2.82
3.78
3.95
4.00
4.44
4.76
5.38
5.84
6.03
6.39
6.56
6.69
7.30
7.76
8.23
8.80
9.44
9.94
10.07
10.51
11.06
11.94
12.14
12.21
13.28
14.41
14.49
14.62
14.73
B
Relative Abundance
Time (min)
0.13
0.82
1.19
1.42
1.98
2.44
3.15
3.80
3.99
4.66
4.89
5.31
5.91
6.39
6.57
6.81
7.13
7.94
8.13
8.74
9.25
9.61
10.12
10.43
11.25
11.87
12.48
12.73
13.96
14.49
14.74
C
Relative Abundance
m/z
108.0218
C6 H4 O2
1.0961 ppm
123.0455
C7 H7 O2
2.8191 ppm
152.0117
C7 H4 O4
1.3591 ppm
167.0352
C8 H7 O4
1.0453 ppm
D
Relative Abundance
m/z
108.0218
C6 H4 O2
1.0900 ppm
123.0455
C7 H7 O2
2.9817 ppm
152.0117
C7 H4 O4
1.4851 ppm
167.0352
C8 H7 O4
1.2879 ppm
293.1035
C15 H17 O6
1.5155 ppm
E
Relative Abundance
m/z
80.0258
C5 H4 O
-11.6919 ppm
108.0218
C6 H4 O2
1.0742 ppm
123.0455
C7 H7 O2
2.9101 ppm
152.0117
C7 H4 O4
1.2345 ppm
167.0352
C8 H7 O4
1.1832 ppm

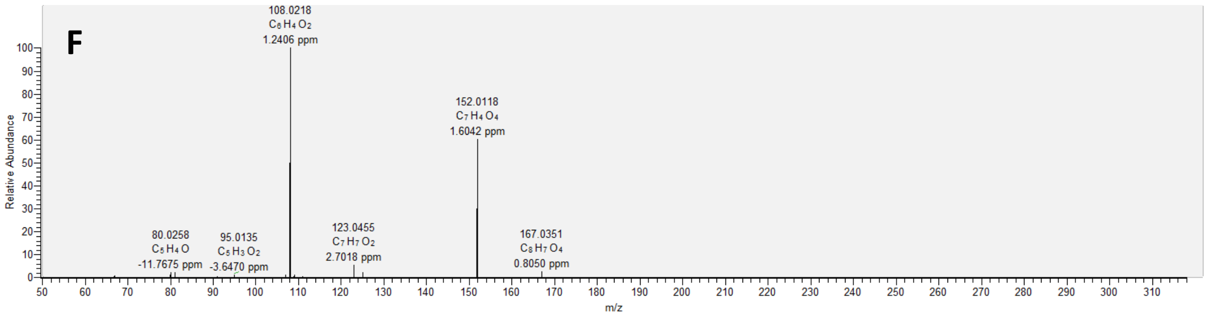

Figure S57: *TIC (A), XIC at 293.1035 m/z (B), HCD35V at 293 nominal mass for isomer 1 (6.39 min, C), HCD35V at 293 nominal mass for isomer 2 (6.56 min D)), HCD75V at 293 nominal mass for isomer 1 (6.39 min E), and HCD75V at 293 nominal mass for isomer 2 (6.56 min, F), for methoxy ether* ***19****.*

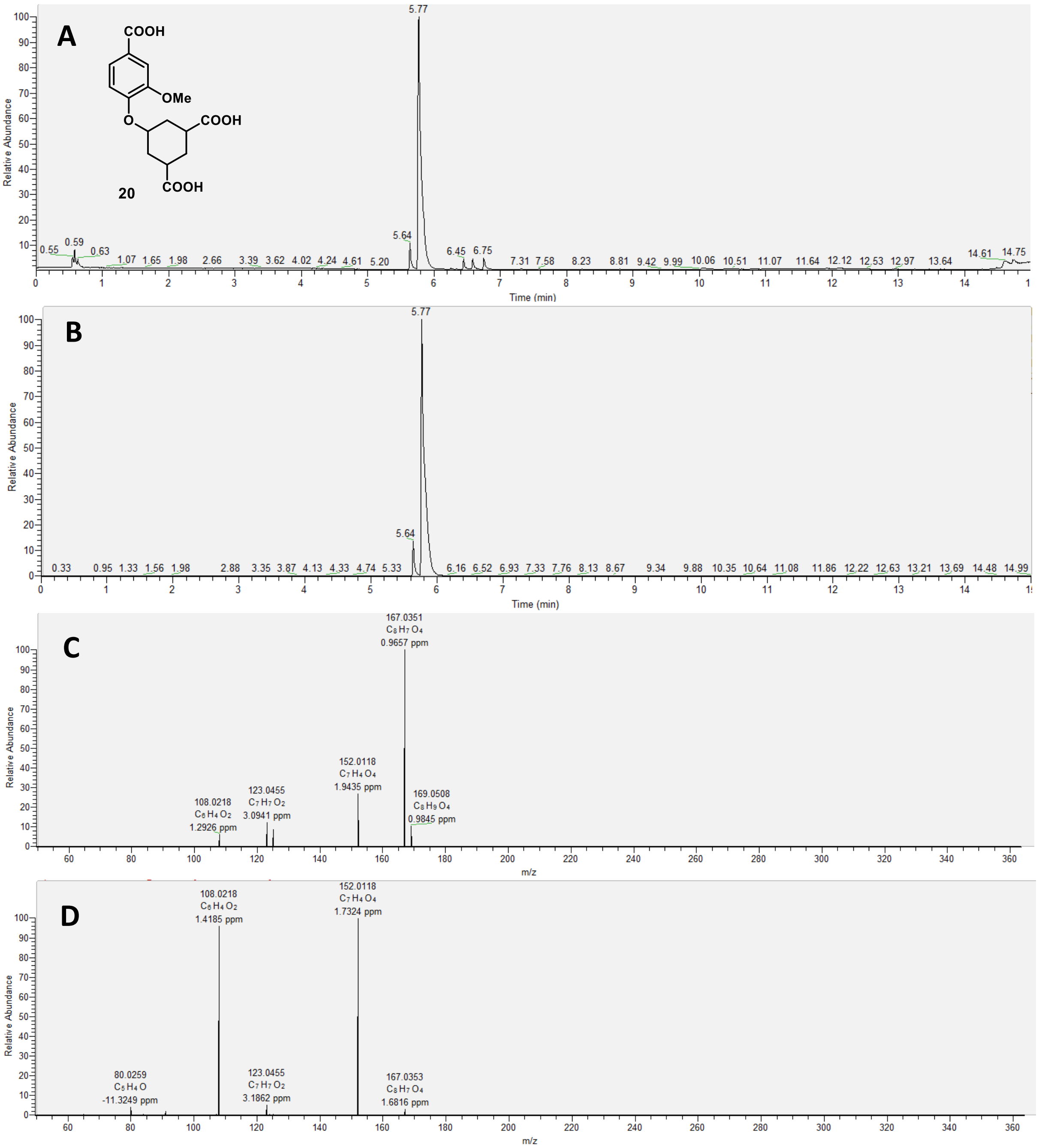

Figure S58: *TIC (A), XIC at 337.0929 m/z (B), HCD35V at 337 nominal mass (C), and HCD75V at 337 nominal mass (D) for methoxy ether **20**.*

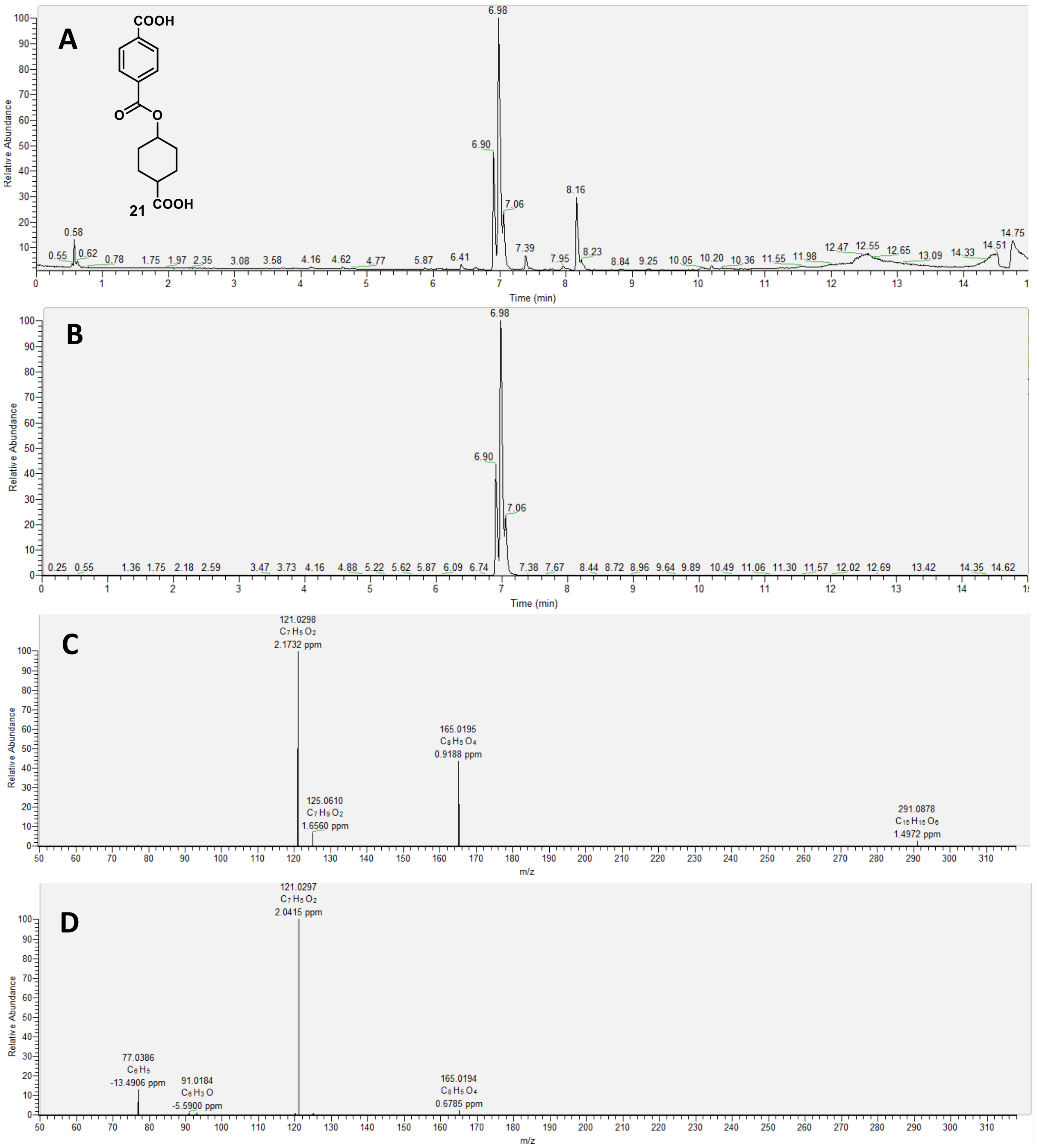

Figure S59: *TIC (A), XIC at 291.0878 m/z (B), HCD35V at 291 nominal mass (C), and HCD75V at 291 nominal mass (D) for dicarboxylic acid **21**.*

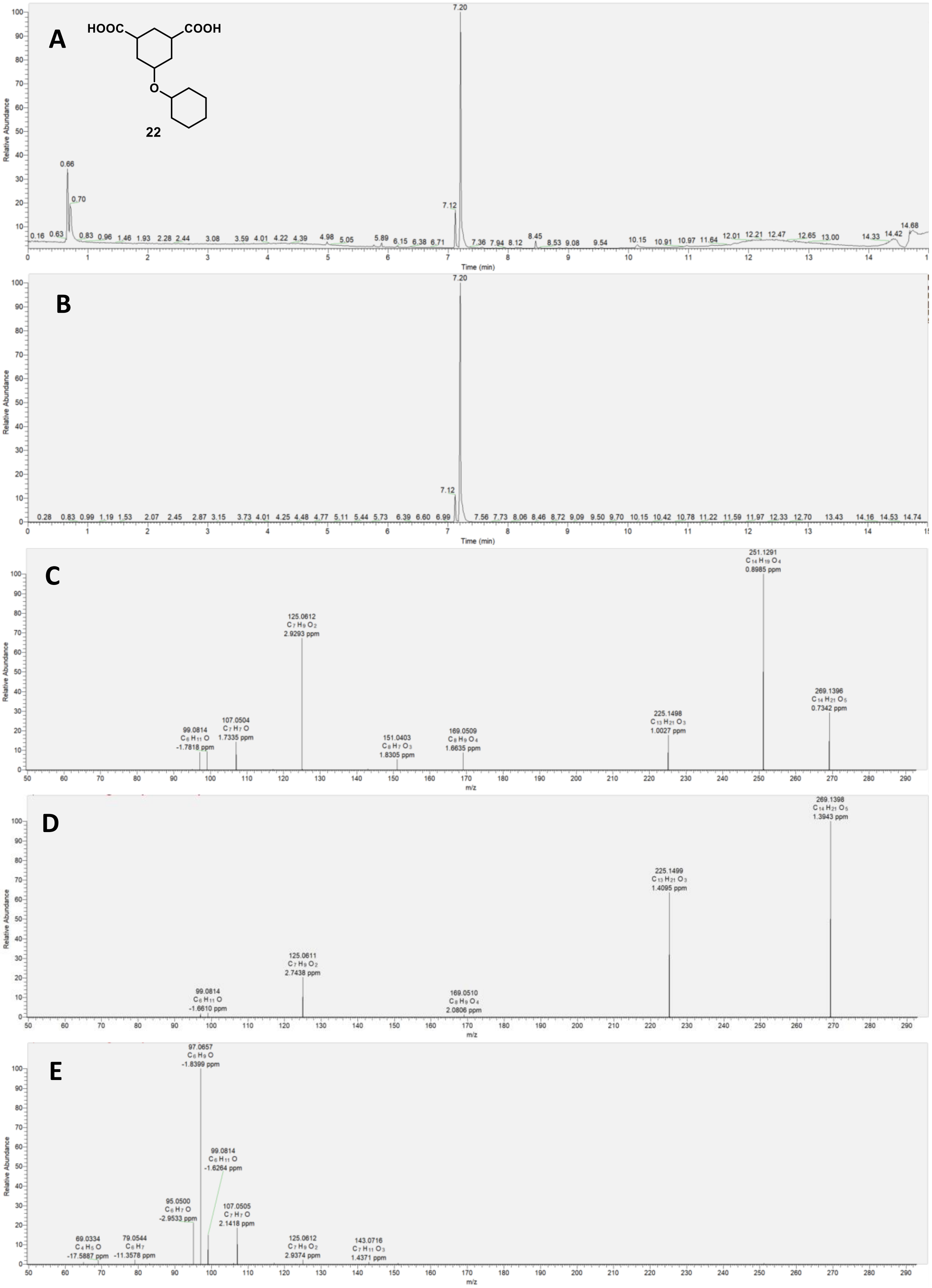

A
HOOC
COOH
O
22
7.20
7.12
0.66
0.70
14.68
Time (min)
Relative Abundance
B
7.20
7.12
Time (min)
C
251.1291
C14 H19 O4
0.8985 ppm
125.0612
C7 H9 O2
2.9293 ppm
269.1396
C14 H21 O5
0.7342 ppm
225.1498
C13 H21 O3
1.0027 ppm
107.0504
C7 H7 O
1.7335 ppm
99.0814
C6 H11 O
-1.7818 ppm
151.0403
C8 H7 O3
1.8305 ppm
169.0509
C8 H9 O4
1.6635 ppm
m/z
D
269.1398
C14 H21 O5
1.3943 ppm
225.1499
C13 H21 O3
1.4095 ppm
125.0611
C7 H9 O2
2.7438 ppm
99.0814
C6 H11 O
-1.6610 ppm
169.0510
C8 H9 O4
2.0806 ppm
m/z
E
97.0657
C6 H9 O
-1.8399 ppm
99.0814
C6 H11 O
-1.6264 ppm
95.0500
C6 H7 O
-2.9533 ppm
107.0505
C7 H7 O
2.1418 ppm
69.0334
C4 H5 O
-17.5887 ppm
79.0544
C6 H7
-11.3578 ppm
125.0612
C7 H9 O2
2.9374 ppm
143.0716
C7 H11 O3
1.4371 ppm
m/z

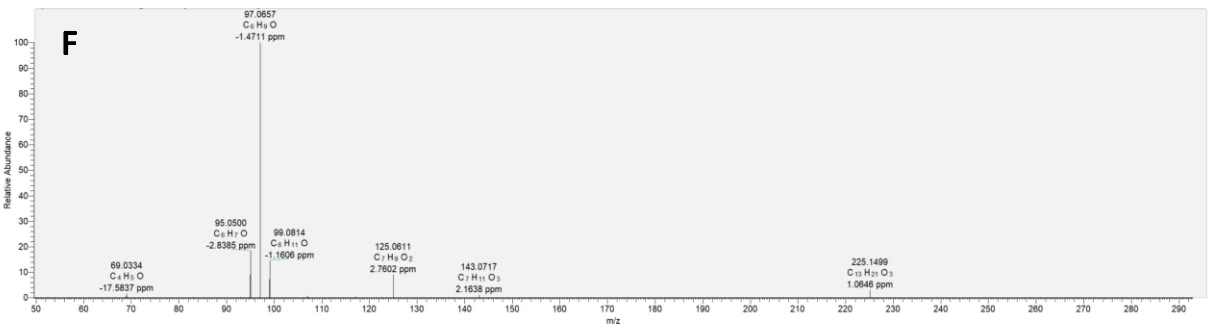

Figure S60: *TIC (A), XIC at 269.1396 m/z (B), HCD35V at 269 nominal mass for isomer 1 (7.12 min, C)), HCD35V at 269 nominal mass for isomer 2 (7.20 min D)), HCD75V at 269 nominal mass for isomer 1 (7.12 min E), and HCD75V at 269 nominal mass for isomer 2 (7.20 min, F), for dicarboxylic acid* ***22****.*

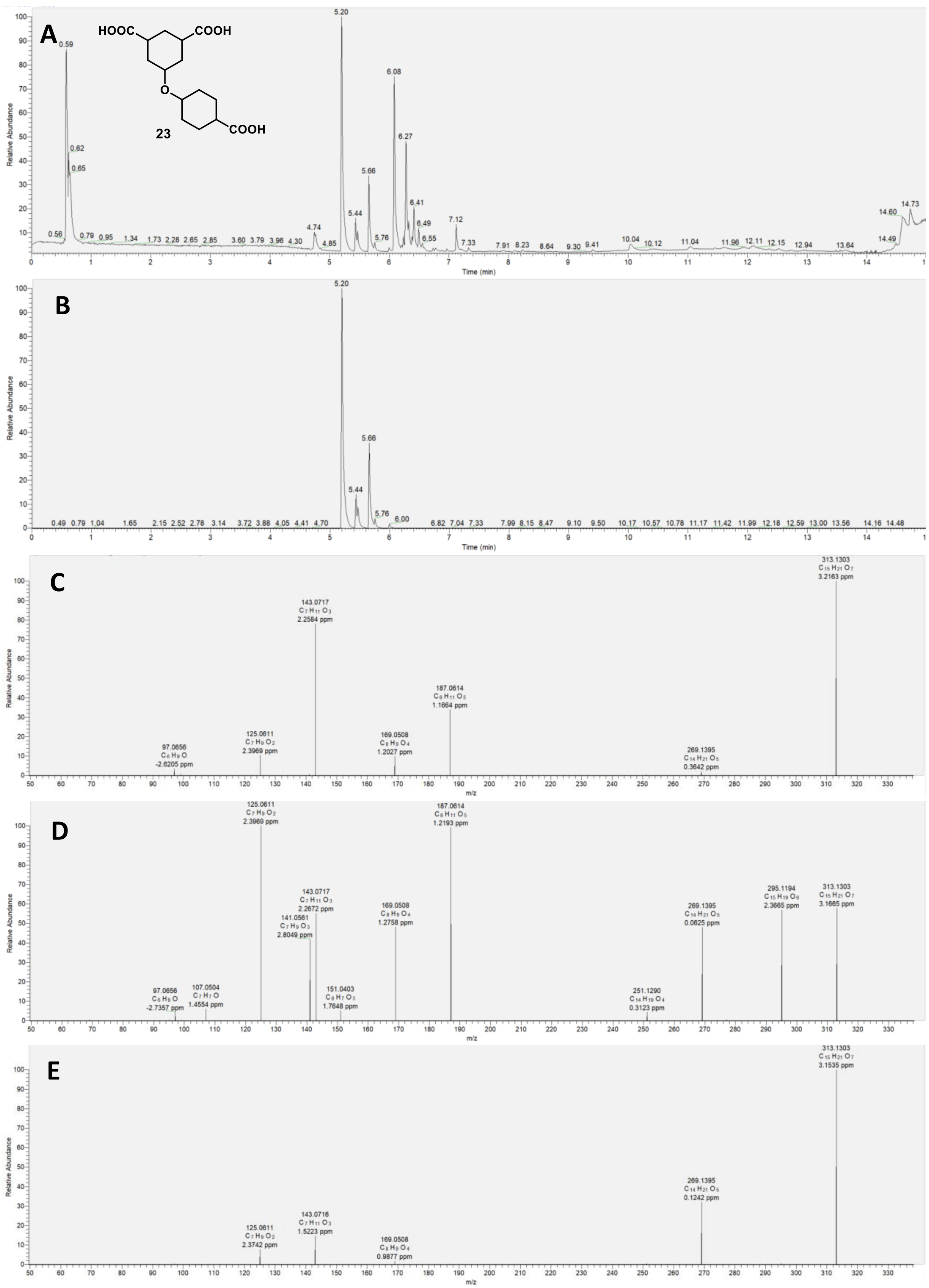
A
HOOC
COOH
O
COOH
23
B
C
313.1303 C15 H21 O7 3.2163 ppm
143.0717 C7 H11 O3 2.2584 ppm
187.0614 C8 H11 O5 1.1664 ppm
125.0611 C7 H9 O2 2.3969 ppm
169.0508 C8 H9 O4 1.2027 ppm
97.0656 C6 H9 O -2.6205 ppm
269.1395 C14 H21 O5 0.3642 ppm
D
125.0611 C7 H9 O2 2.3969 ppm
187.0614 C8 H11 O5 1.2193 ppm
143.0717 C7 H11 O3 2.2672 ppm
141.0561 C7 H9 O3 2.8049 ppm
169.0508 C8 H9 O4 1.2758 ppm
269.1395 C14 H21 O5 0.0625 ppm
295.1194 C15 H19 O6 2.3665 ppm
313.1303 C15 H21 O7 3.1665 ppm
97.0656 C6 H9 O -2.7357 ppm
107.0504 C7 H7 O 1.4554 ppm
151.0403 C8 H7 O3 1.7648 ppm
251.1290 C14 H19 O4 0.3123 ppm
E
313.1303 C15 H21 O7 3.1535 ppm
269.1395 C14 H21 O5 0.1242 ppm
125.0611 C7 H9 O2 2.3742 ppm
143.0716 C7 H11 O3 1.5223 ppm
169.0508 C8 H9 O4 0.9877 ppm
Relative Abundance
Time (min)
m/z

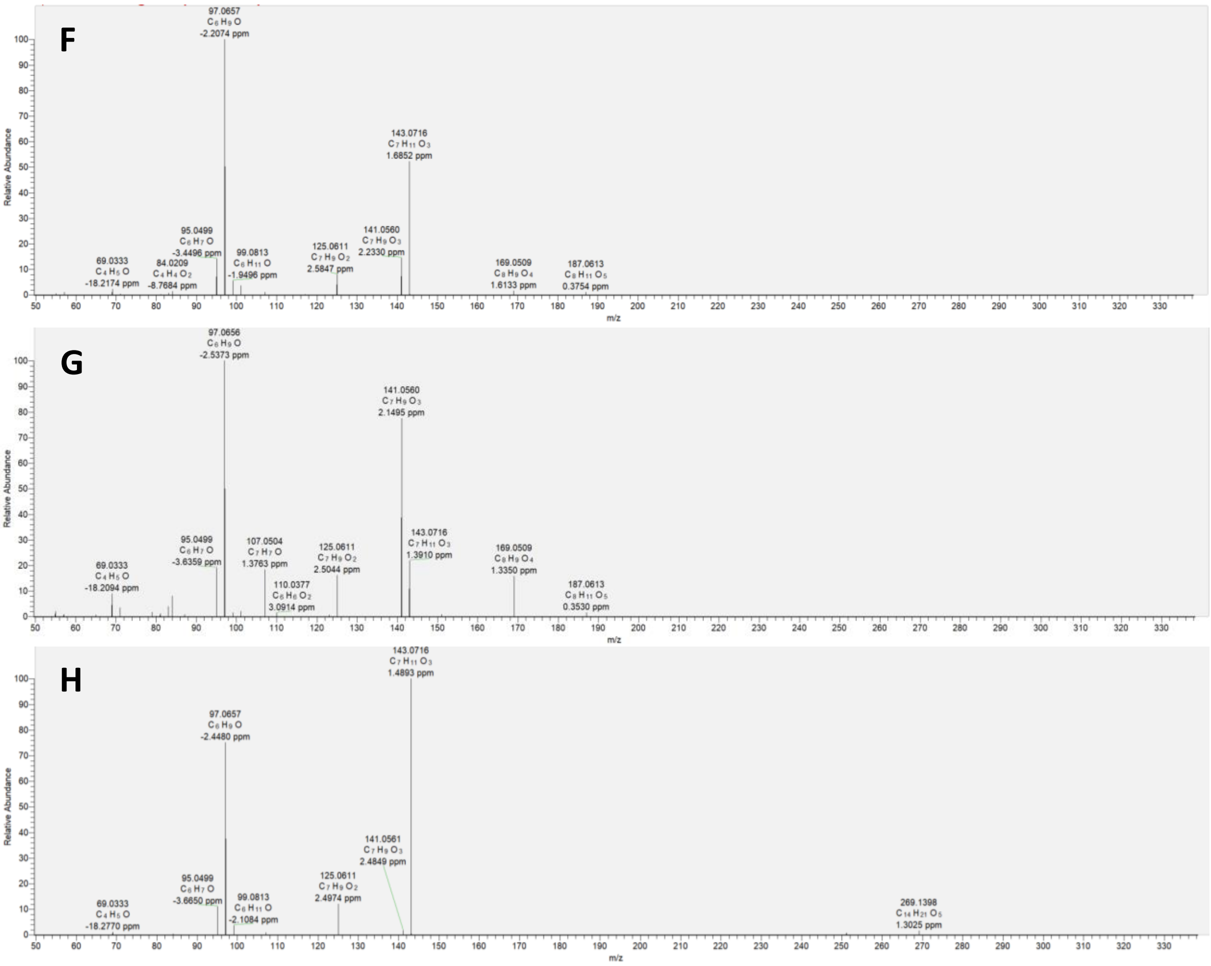

Figure S61: *TIC (A), XIC at 313.1303 m/z (B), HCD35V at 313 nominal mass for isomer 1 (5.20 min, C), HCD35V at 313 nominal mass for isomer 2 (5.45 min, D), HCD35V at 313 nominal mass for isomer 3 (5.66 min, E), HCD75V at 313 nominal mass for isomer 1 (5.20 min, F), HCD75V at 313 nominal mass for isomer 2 (5.45 min, G), and HCD75V at 313 nominal mass for isomer 3 (5.66 min, H), for aliphatic tricarboxylic acid* ***23****.*

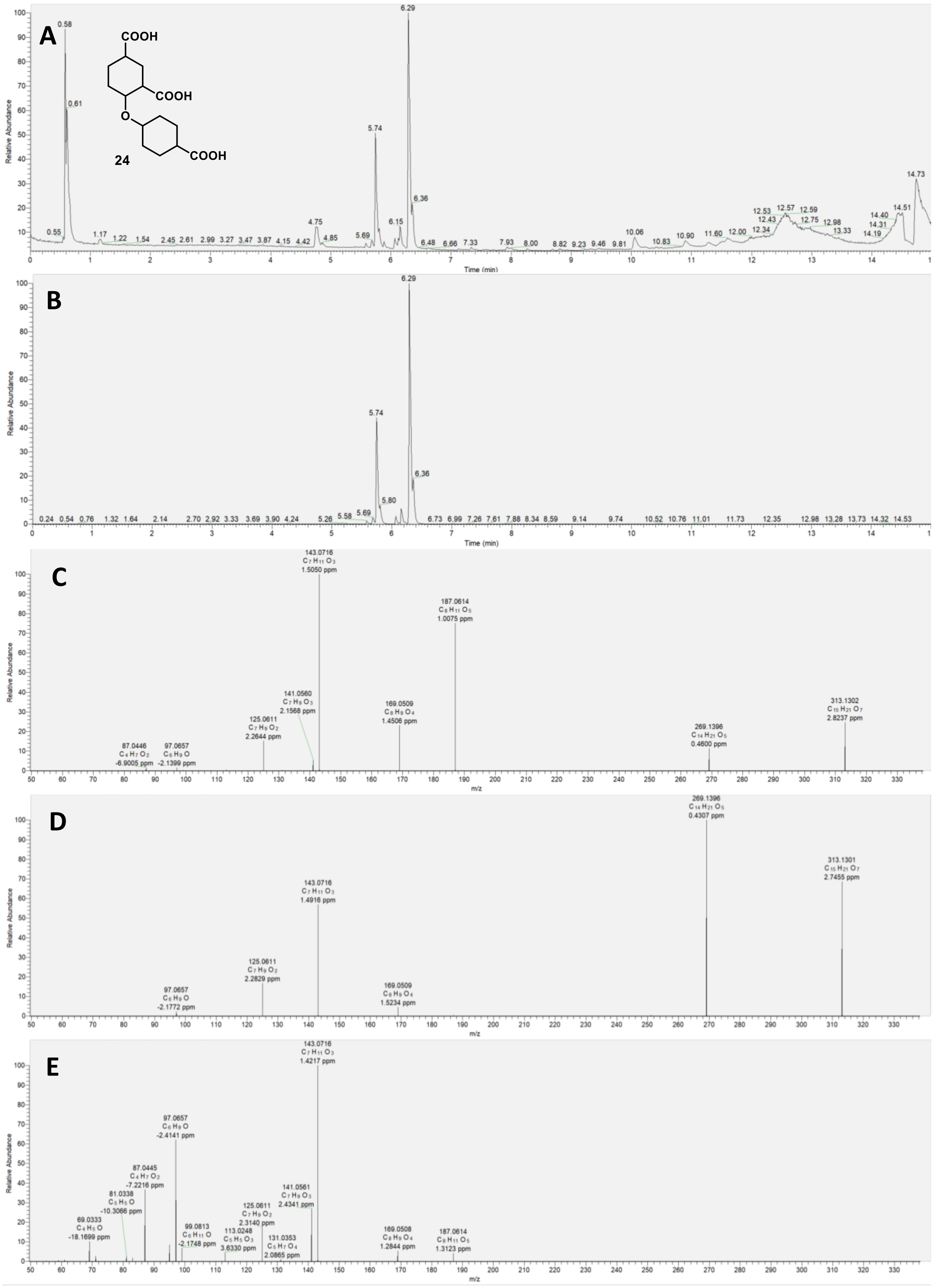
A
COOH
COOH
O
COOH
24
0.58
0.61
5.74
6.29
6.36
14.73
B
5.74
6.29
6.36
C
143.0716
C7 H11 O3
1.5050 ppm
187.0614
C8 H11 O5
1.0075 ppm
141.0560
C7 H9 O3
2.1568 ppm
125.0611
C7 H9 O2
2.2644 ppm
169.0509
C8 H9 O4
1.4506 ppm
87.0446
C4 H7 O2
-6.9005 ppm
97.0657
C6 H9 O
-2.1399 ppm
269.1396
C14 H21 O5
0.4600 ppm
313.1302
C15 H21 O7
2.8237 ppm
D
269.1396
C14 H21 O5
0.4307 ppm
313.1301
C15 H21 O7
2.7455 ppm
143.0716
C7 H11 O3
1.4916 ppm
125.0611
C7 H9 O2
2.2829 ppm
97.0657
C6 H9 O
-2.1772 ppm
169.0509
C8 H9 O4
1.5234 ppm
E
143.0716
C7 H11 O3
1.4217 ppm
97.0657
C6 H9 O
-2.4141 ppm
87.0445
C4 H7 O2
-7.2216 ppm
81.0338
C5 H5 O
-10.3066 ppm
69.0333
C4 H5 O
-18.1699 ppm
99.0813
C6 H11 O
-2.1748 ppm
113.0248
C5 H5 O3
3.6330 ppm
125.0611
C7 H9 O2
2.3140 ppm
131.0353
C5 H7 O4
2.0865 ppm
141.0561
C7 H9 O3
2.4341 ppm
169.0508
C8 H9 O4
1.2844 ppm
187.0614
C8 H11 O5
1.3123 ppm
Relative Abundance
Time (min)
m/z

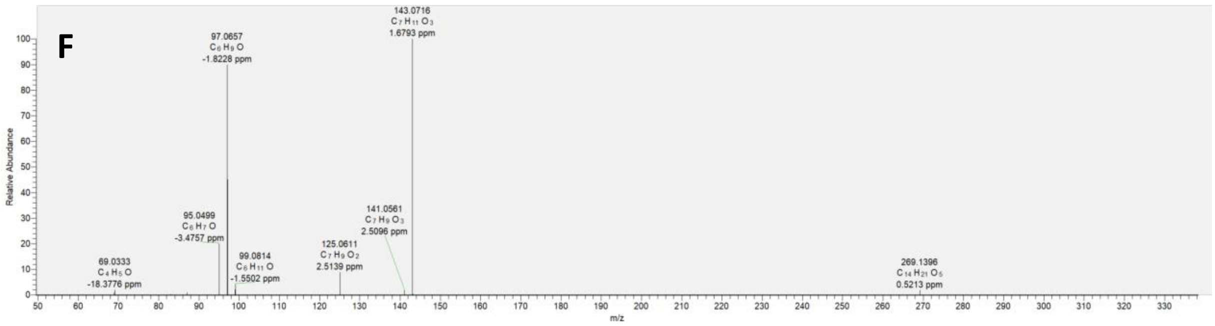

Figure S62: *TIC (A), XIC at 313.1301 m/z (B), HCD35V at 313 nominal mass for isomer 1 (5.74 min, C), HCD35V at 313 nominal mass for isomer 2 (6.29 min, D), HCD75V at 313 nominal mass for isomer 1 (5.74 min, E), and HCD75V at 313 nominal mass for isomer 2 (6.29 min, F), for tricarboxylic acid* ***24****.*

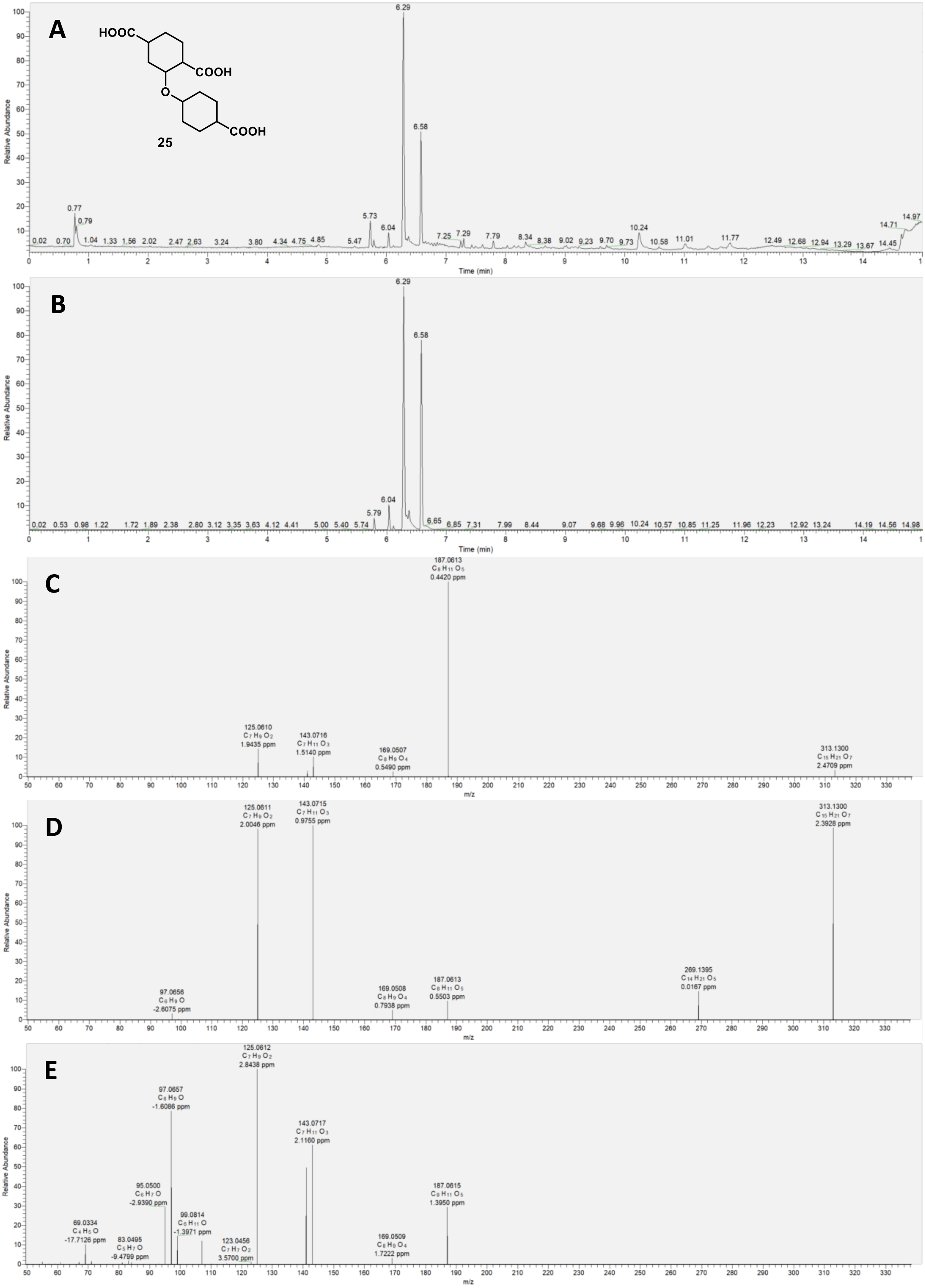
A
HOOC
COOH
O
COOH
25
Relative Abundance
Time (min)
0.77
0.79
5.73
6.04
6.29
6.58
7.29
7.79
8.34
10.24
11.01
11.77
14.71
14.97
B
6.29
6.58
5.79
6.04
6.65
C
187.0613
C8 H11 O5
0.4420 ppm
125.0610
C7 H9 O2
1.9435 ppm
143.0716
C7 H11 O3
1.5140 ppm
169.0507
C8 H9 O4
0.5490 ppm
313.1300
C15 H21 O7
2.4709 ppm
m/z
D
125.0611
C7 H9 O2
2.0046 ppm
143.0715
C7 H11 O3
0.9755 ppm
313.1300
C15 H21 O7
2.3928 ppm
97.0656
C6 H9 O
-2.6075 ppm
169.0508
C8 H9 O4
0.7938 ppm
187.0613
C8 H11 O5
0.5503 ppm
269.1395
C14 H21 O5
0.0167 ppm
E
125.0612
C7 H9 O2
2.8438 ppm
97.0657
C6 H9 O
-1.6086 ppm
143.0717
C7 H11 O3
2.1160 ppm
95.0500
C6 H7 O
-2.9390 ppm
187.0615
C8 H11 O5
1.3950 ppm
69.0334
C4 H5 O
-17.7126 ppm
99.0814
C6 H11 O
-1.3971 ppm
83.0495
C5 H7 O
-9.4799 ppm
123.0456
C7 H7 O2
3.5700 ppm
169.0509
C8 H9 O4
1.7222 ppm

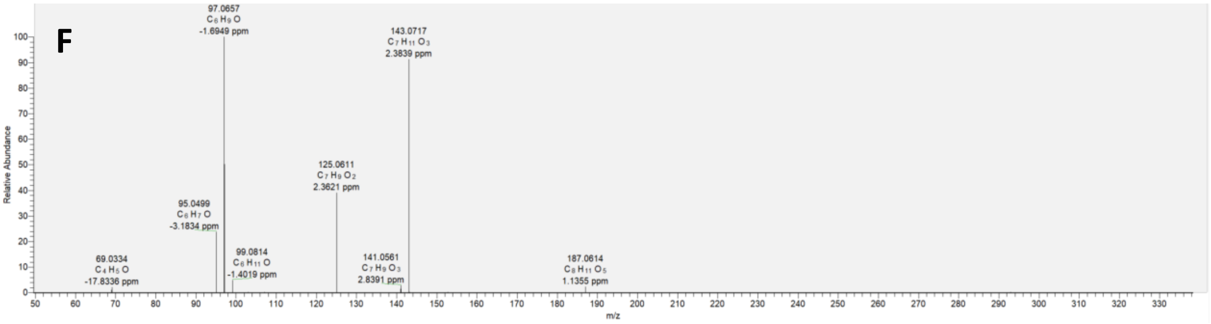

Figure S63: *TIC (A), XIC at 313.1300 m/z (B), HCD35V at 313 nominal mass for isomer 1 (5.66 min, C), HCD35V at 313 nominal mass for isomer 2 (5.99 min, D), HCD75V at 313 nominal mass for isomer 1 (5.66 min, E), and HCD75V at 313 nominal mass for isomer 2 (5.99 min, F), for tricarboxylic acid* ***25****.*

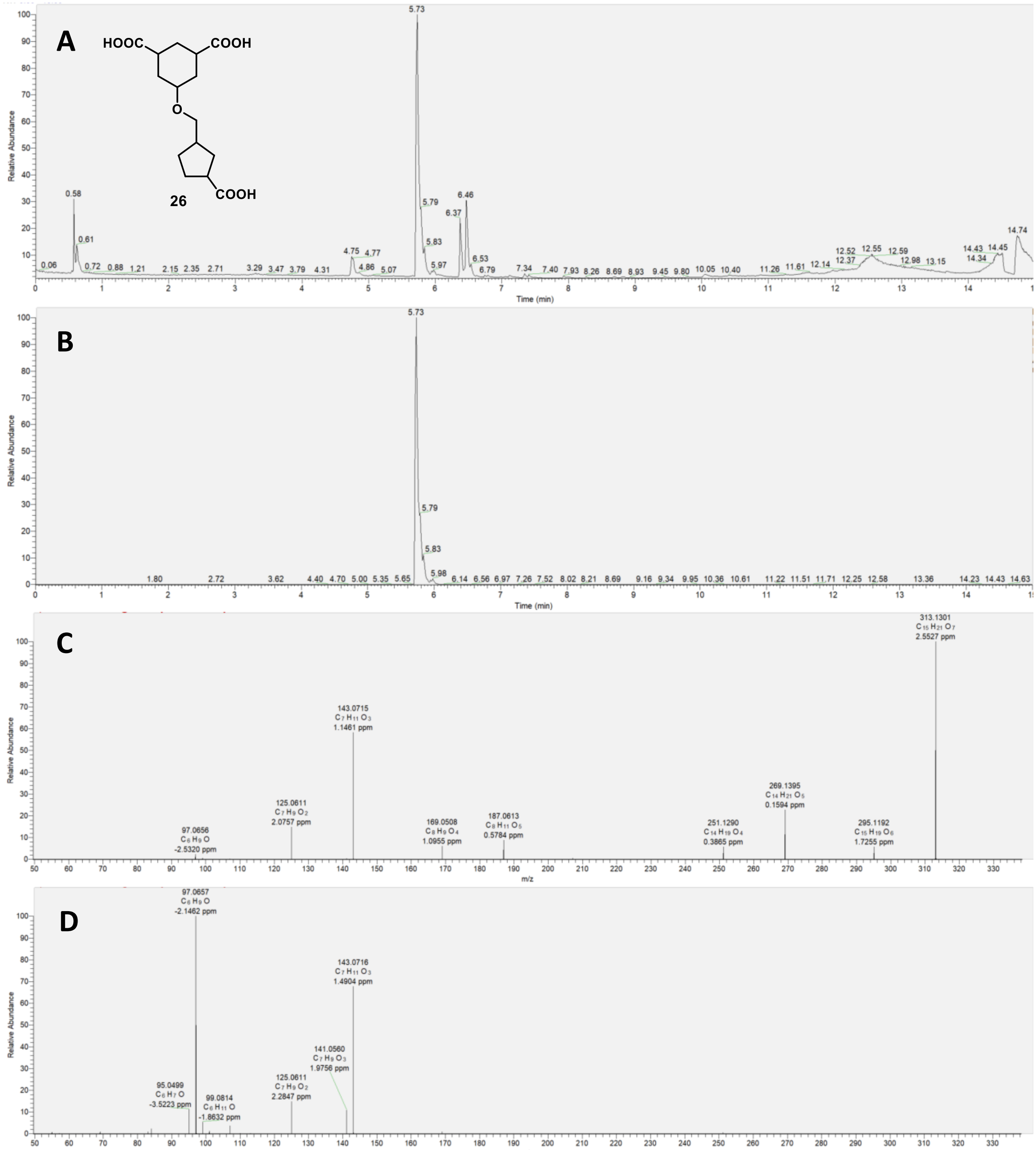

Figure S64: *TIC (A), XIC at 313.1301 m/z (B), HCD35V at 313 nominal mass (C), and HCD75V at 313 nominal mass (D) for tricarboxylic acid* ***26****.*

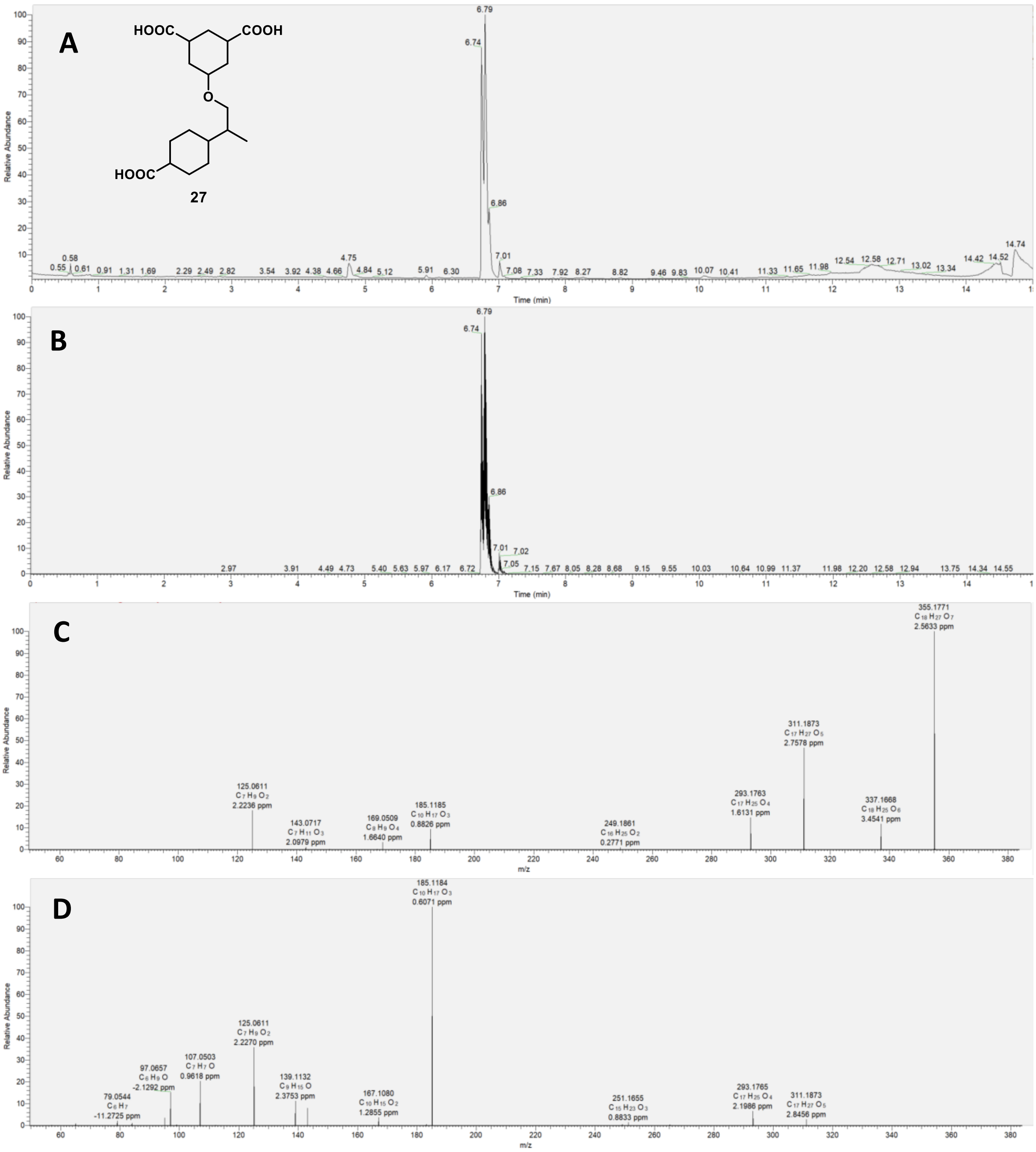

Figure S65: *TIC (A), XIC at 355.1771 m/z (B), HCD35V at 355 nominal mass (C), and HCD75V at 355 nominal mass (D) for tricarboxylic acid **27**.*

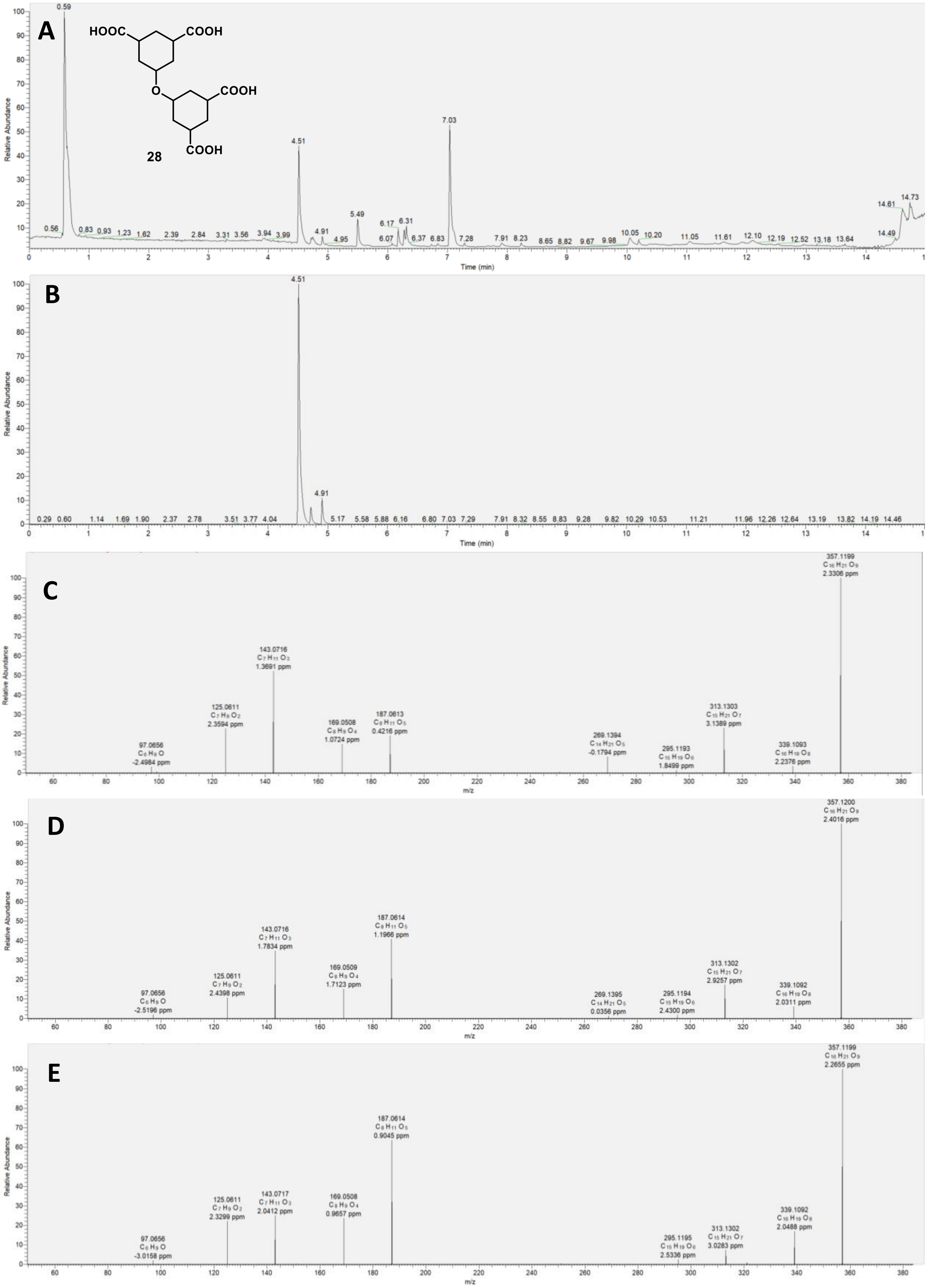
A
HOOC
COOH
COOH
COOH
O
28
B
C
D
E
Relative Abundance
Time (min)
m/z
357.1199
C16 H21 O9
2.3306 ppm
357.1200
C16 H21 O9
2.4016 ppm
357.1199
C16 H21 O9
2.2655 ppm

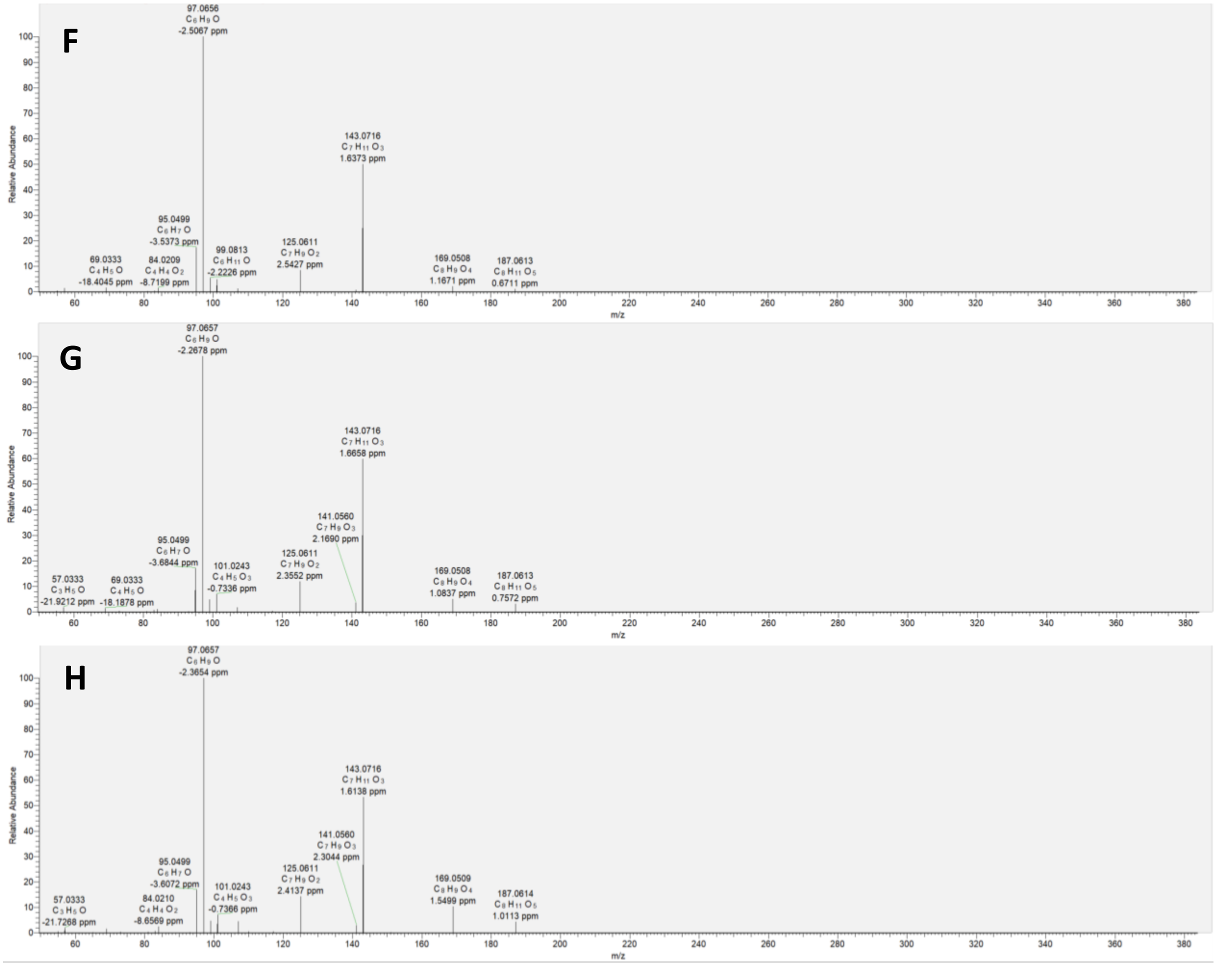

Figure S66: *TIC (A), XIC at 357.1200 m/z (B), HCD35V at 357 nominal mass for isomer 1 (4.51 min, C), HCD35V at 357 nominal mass for isomer 2 (4.72 min, D), HCD75V at 357 nominal mass for isomer 1 (4.51 min, E), and HCD75V at 357 nominal mass for isomer 2 (4.72 min, F), for tetracarboxylic acid* ***28****.*

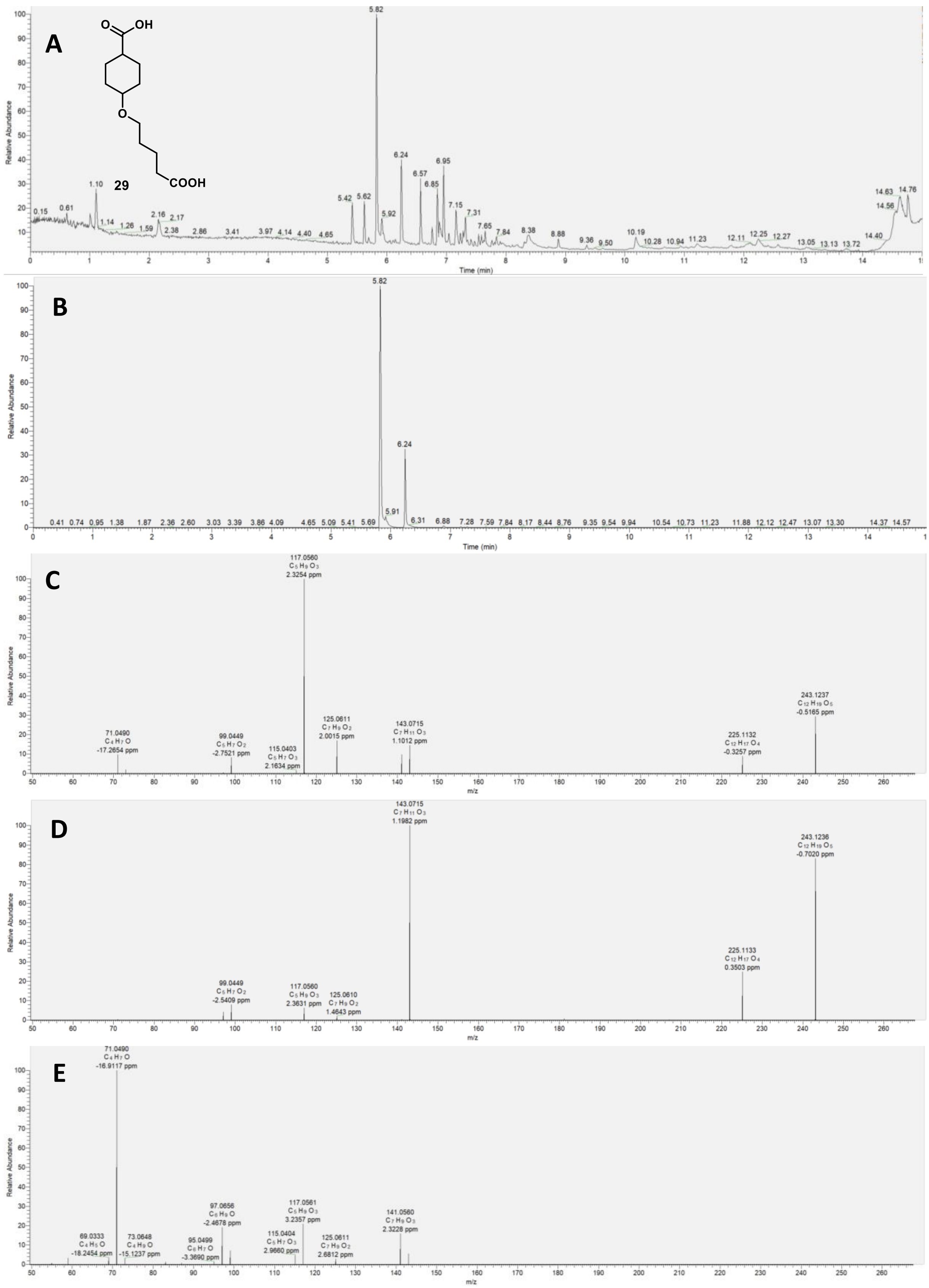
A
O OH
O
29
COOH
Relative Abundance
Time (min)
5.82
6.24
6.57
6.85
6.95
7.15
7.31
5.42
5.62
5.92
1.10
14.63
14.56
14.76
B
5.82
6.24
5.91
6.31
C
117.0560
C5 H9 O3
2.3254 ppm
71.0490
C4 H7 O
-17.2654 ppm
99.0449
C5 H7 O2
-2.7521 ppm
115.0403
C5 H7 O3
2.1634 ppm
125.0611
C7 H9 O2
2.0015 ppm
143.0715
C7 H11 O3
1.1012 ppm
225.1132
C12 H17 O4
-0.3257 ppm
243.1237
C12 H19 O5
-0.5165 ppm
m/z
D
143.0715
C7 H11 O3
1.1982 ppm
243.1236
C12 H19 O5
-0.7020 ppm
225.1133
C12 H17 O4
0.3503 ppm
99.0449
C5 H7 O2
-2.5409 ppm
117.0560
C5 H9 O3
2.3631 ppm
125.0610
C7 H9 O2
1.4643 ppm
E
71.0490
C4 H7 O
-16.9117 ppm
69.0333
C4 H5 O
-18.2454 ppm
73.0648
C4 H9 O
-15.1237 ppm
95.0499
C6 H7 O
-3.3690 ppm
97.0656
C6 H9 O
-2.4678 ppm
115.0404
C5 H7 O3
2.9660 ppm
117.0561
C5 H9 O3
3.2357 ppm
125.0611
C7 H9 O2
2.6812 ppm
141.0560
C7 H9 O3
2.3228 ppm

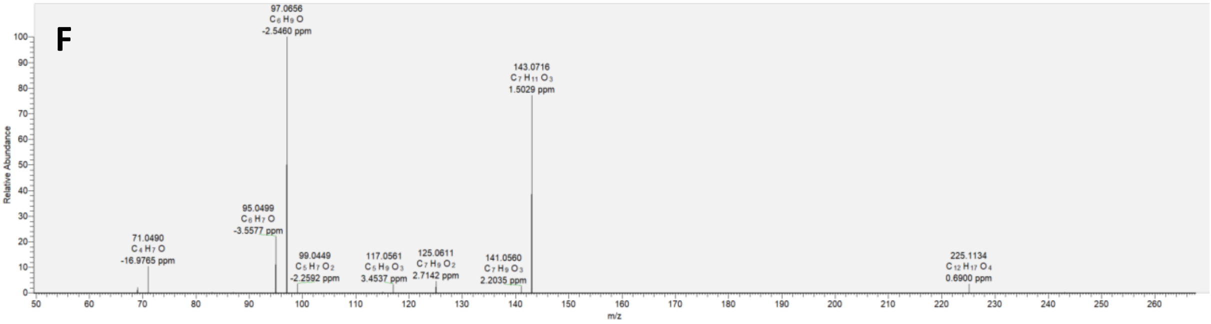

Figure S67: *TIC (A), XIC at 243.1237 m/z (B), HCD35V at 243 nominal mass for isomer 1 (5.82 min, C), HCD35V at 243 nominal mass for isomer 2 (6.24 min, D), HCD75V at 243 nominal mass for isomer 1 (5.82 min, E), and HCD75V at 243 nominal mass for isomer 2 (6.24 min, F), for dicarboxylic acid* ***29.***

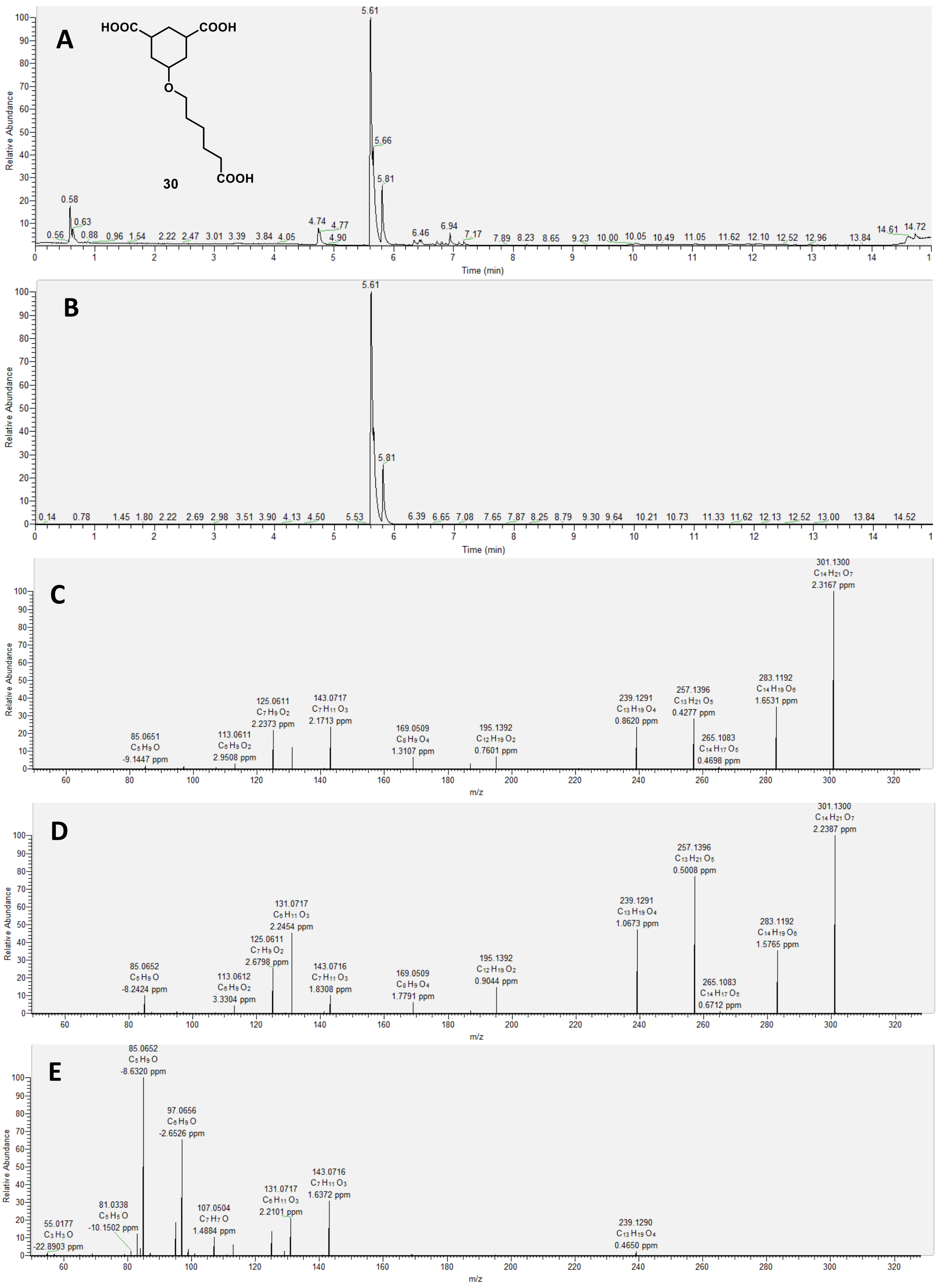
A
HOOC
COOH
O
COOH
30
B
C
D
E
Relative Abundance
Time (min)
m/z
301.1300
C14 H21 O7
283.1192
C14 H19 O6
257.1396
C13 H21 O5
239.1291
C13 H19 O4
265.1083
C14 H17 O5
195.1392
C12 H19 O2
169.0509
C8 H9 O4
143.0717
C7 H11 O3
131.0717
C6 H11 O3
125.0611
C7 H9 O2
113.0611
C6 H9 O2
97.0656
C6 H9 O
85.0652
C5 H9 O
81.0338
C5 H5 O
107.0504
C7 H7 O
55.0177
C3 H3 O

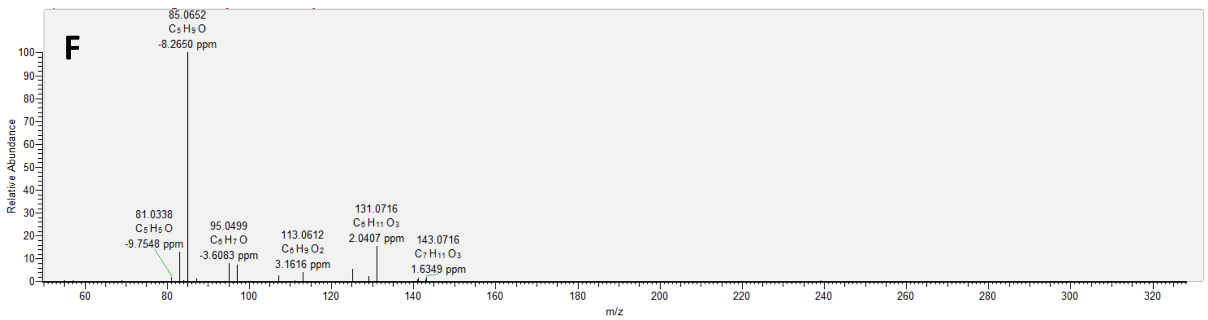

Figure S68: *TIC (A), XIC at 301.1300 m/z (B), HCD35V at 301 nominal mass for isomer 1 (5.61 min, C), HCD35V at 301 nominal mass for isomer 2 (5.81 min, D), HCD75V at 301 nominal mass for isomer 1 (5.61 min, E), and HCD75V at 301 nominal mass for isomer 2 (5.81 min, F), for tricarboxylic acid* ***30****.*

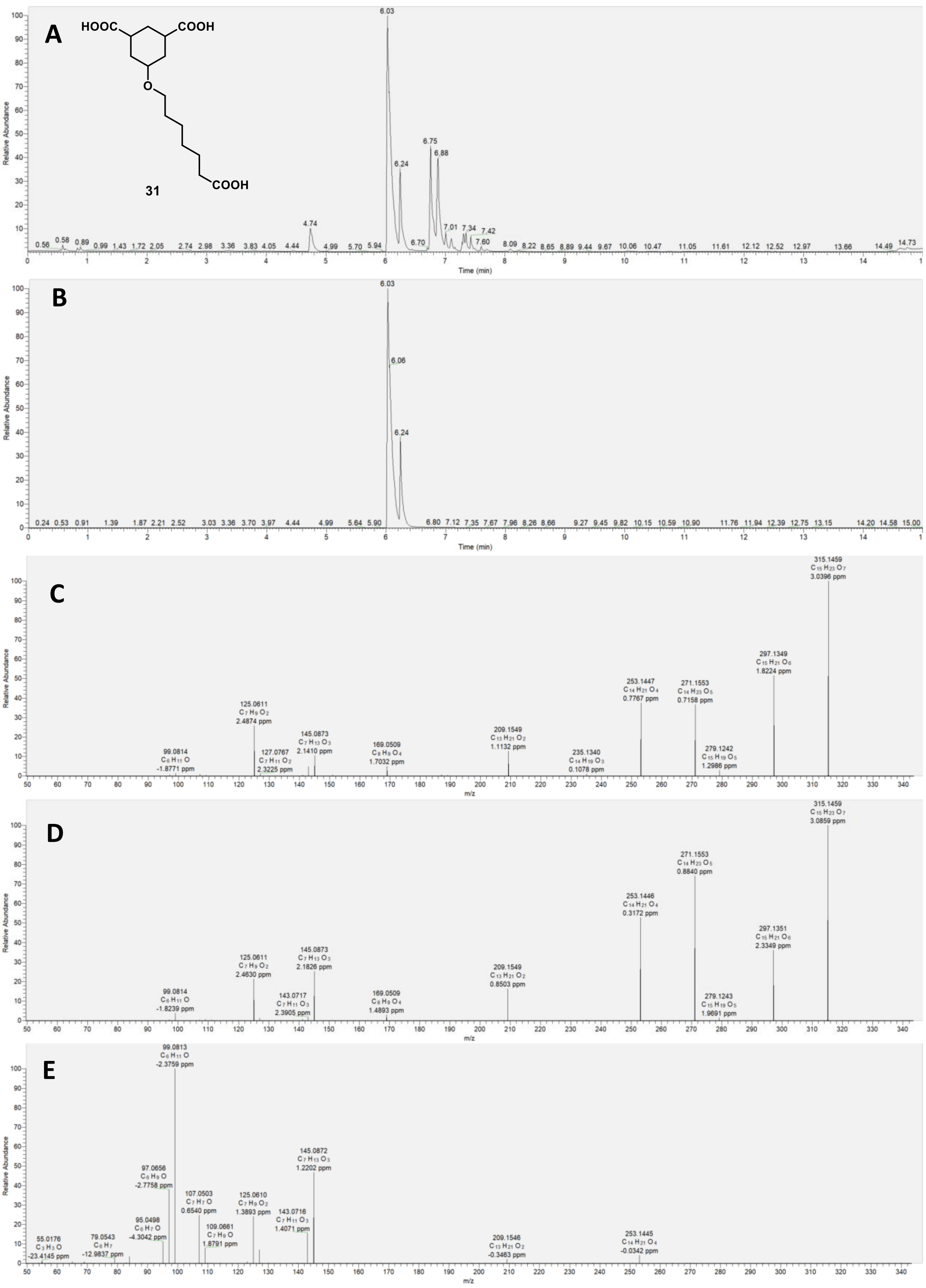
A
HOOC
COOH
O
COOH
31
Relative Abundance
Time (min)
6.03
6.24
6.75
6.88
7.01
7.34
7.42
7.60
4.74
B
Relative Abundance
Time (min)
6.03
6.06
6.24
C
Relative Abundance
m/z
315.1459
C15 H23 O7
3.0396 ppm
297.1349
C15 H21 O6
1.8224 ppm
271.1553
C14 H23 O5
0.7158 ppm
253.1447
C14 H21 O4
0.7767 ppm
279.1242
C15 H19 O5
1.2986 ppm
235.1340
C14 H19 O3
0.1078 ppm
209.1549
C13 H21 O2
1.1132 ppm
169.0509
C8 H9 O4
1.7032 ppm
145.0873
C7 H13 O3
2.1410 ppm
127.0767
C7 H11 O2
2.3225 ppm
125.0611
C7 H9 O2
2.4874 ppm
99.0814
C6 H11 O
-1.8771 ppm
D
Relative Abundance
m/z
315.1459
C15 H23 O7
3.0859 ppm
271.1553
C14 H23 O5
0.8840 ppm
253.1446
C14 H21 O4
0.3172 ppm
297.1351
C15 H21 O6
2.3349 ppm
279.1243
C15 H19 O5
1.9691 ppm
209.1549
C13 H21 O2
0.8503 ppm
169.0509
C8 H9 O4
1.4893 ppm
145.0873
C7 H13 O3
2.1826 ppm
143.0717
C7 H11 O3
2.3905 ppm
125.0611
C7 H9 O2
2.4630 ppm
99.0814
C6 H11 O
-1.8239 ppm
E
Relative Abundance
m/z
99.0813
C6 H11 O
-2.3759 ppm
145.0872
C7 H13 O3
1.2202 ppm
97.0656
C6 H9 O
-2.7758 ppm
107.0503
C7 H7 O
0.6540 ppm
125.0610
C7 H9 O2
1.3893 ppm
95.0498
C6 H7 O
-4.3042 ppm
109.0661
C7 H9 O
1.8791 ppm
143.0716
C7 H11 O3
1.4071 ppm
79.0543
C6 H7
-12.9837 ppm
55.0176
C3 H3 O
-23.4145 ppm
209.1546
C13 H21 O2
-0.3463 ppm
253.1445
C14 H21 O4
-0.0342 ppm

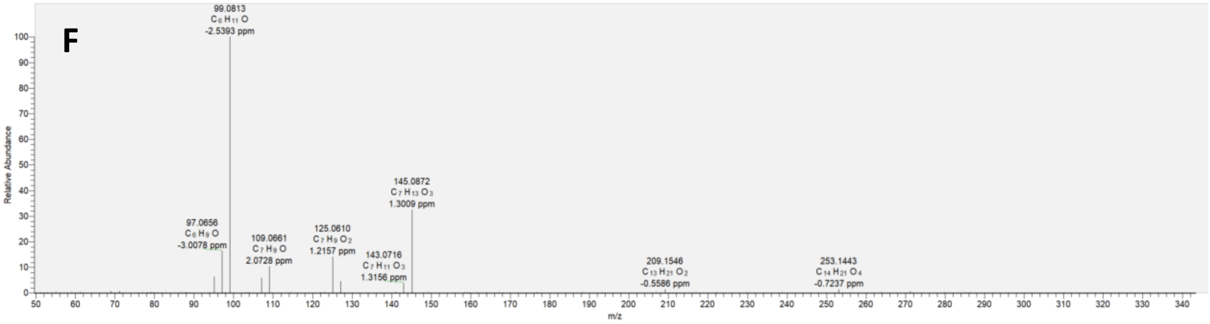

Figure S69: *TIC (A), XIC at 315.1459 m/z (B), HCD35V at 315 nominal mass for isomer 1 (6.03 min, C), HCD35V at 315 nominal mass for isomer 2 (6.24 min, D), HCD75V at 315 nominal mass for isomer 1 (6.03 min, E), and HCD75V at 315 nominal mass for isomer 2 (6.24 min, F), for tricarboxylic acid* ***31****.*

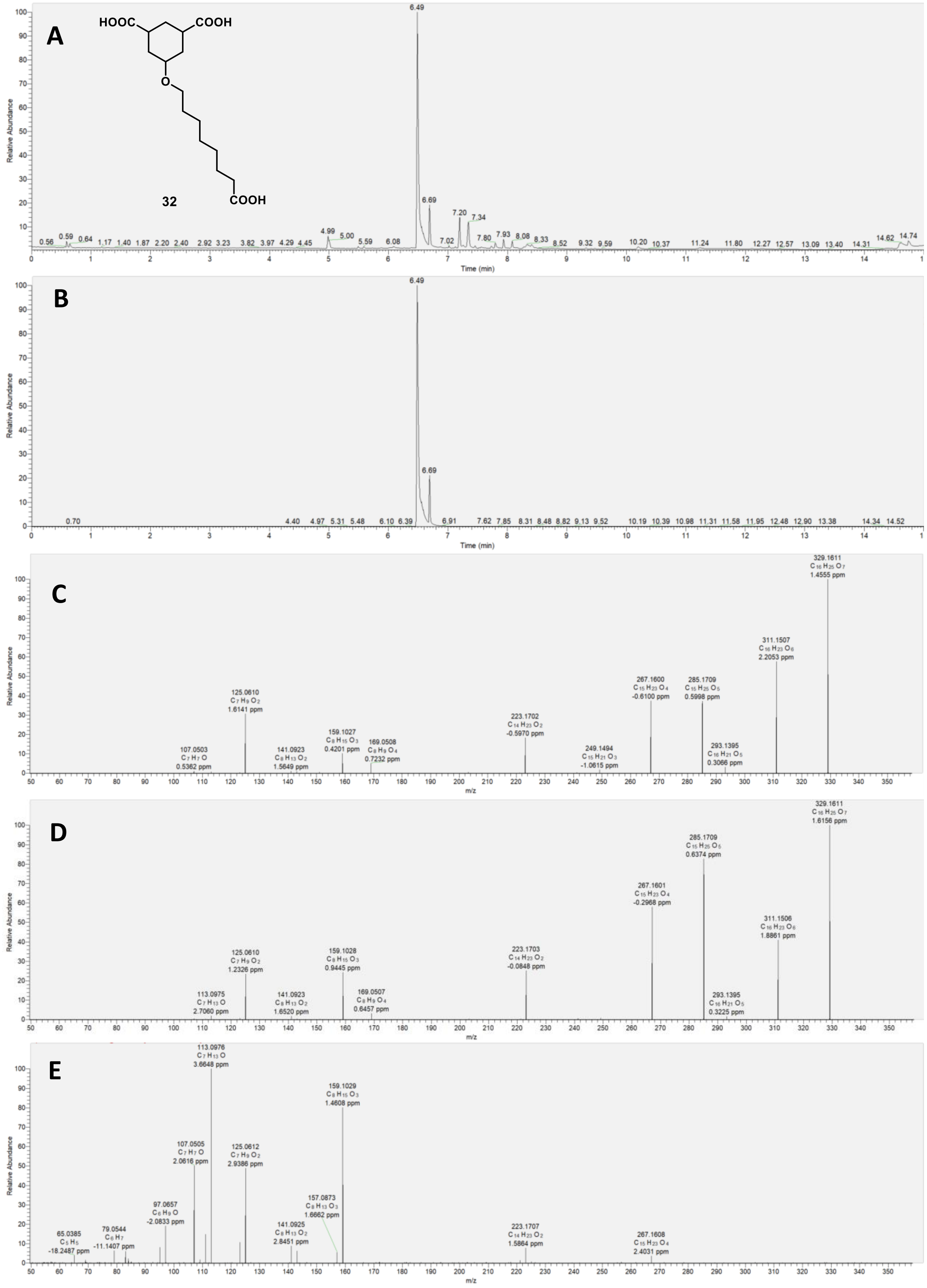
A
HOOC
COOH
O
32
COOH
B
C
329.1611 C16 H25 O7 1.4555 ppm
311.1507 C16 H23 O6 2.2053 ppm
285.1709 C15 H25 O5 0.5998 ppm
267.1600 C15 H23 O4 -0.6100 ppm
223.1702 C14 H23 O2 -0.5970 ppm
125.0610 C7 H9 O2 1.6141 ppm
D
329.1611 C16 H25 O7 1.6156 ppm
285.1709 C15 H25 O5 0.6374 ppm
267.1601 C15 H23 O4 -0.2968 ppm
311.1506 C16 H23 O6 1.8861 ppm
223.1703 C14 H23 O2 -0.0848 ppm
159.1028 C8 H15 O3 0.9445 ppm
125.0610 C7 H9 O2 1.2326 ppm
E
113.0976 C7 H13 O 3.6648 ppm
159.1029 C8 H15 O3 1.4608 ppm
107.0505 C7 H7 O 2.0616 ppm
125.0612 C7 H9 O2 2.9386 ppm
223.1707 C14 H23 O2 1.5864 ppm
267.1608 C15 H23 O4 2.4031 ppm
Time (min)
m/z
Relative Abundance

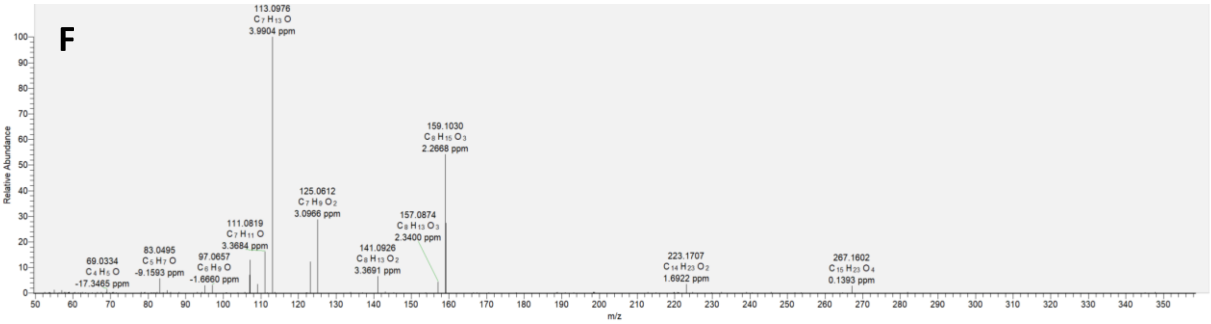

Figure S70: *TIC (A), XIC at 329.1611 m/z (B), HCD35V at 329 nominal mass for isomer 1 (6.49 min, C), HCD35V at 329 nominal mass for isomer 2 (6.69 min, D), HCD75V at 329 nominal mass for isomer 1 (6.49 min, E), and HCD75V at 329 nominal mass for isomer 2 (6.69 min, F), for tricarboxylic acid* ***32****.*

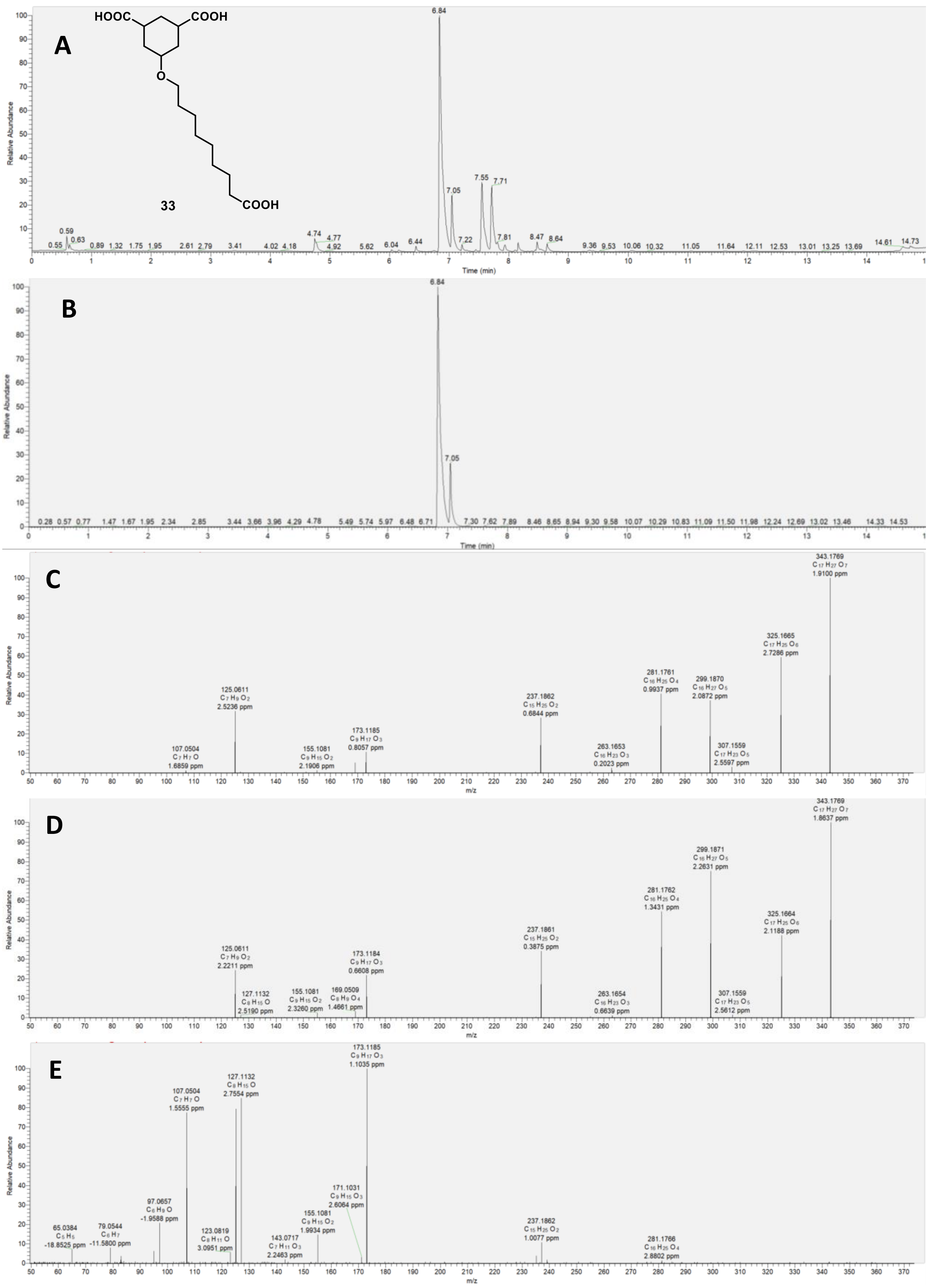
A
HOOC
COOH
O
COOH
33
B
C
D
E
Relative Abundance
Time (min)
m/z
343.1769
C17 H27 O7
325.1665
C17 H25 O6
299.1870
C16 H27 O5
281.1761
C16 H25 O4
237.1862
C15 H25 O2
173.1185
C9 H17 O3
125.0611
C7 H9 O2
107.0504
C7 H7 O

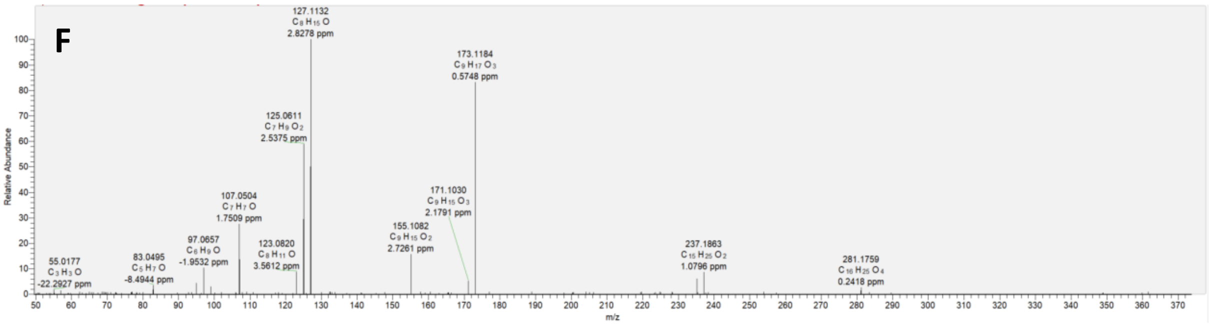

Figure S71: *TIC (A), XIC at 343.1769 m/z (B), HCD35V at 343 nominal mass for isomer 1 (6.84 min, C), HCD35V at 343 nominal mass for isomer 2 (7.05 min, D), HCD75V at 343 nominal mass for isomer 1 (6.84 min, E), and HCD75V at 343 nominal mass for isomer 2 (7.05 min, F), for tricarboxylic acid* ***33****.*

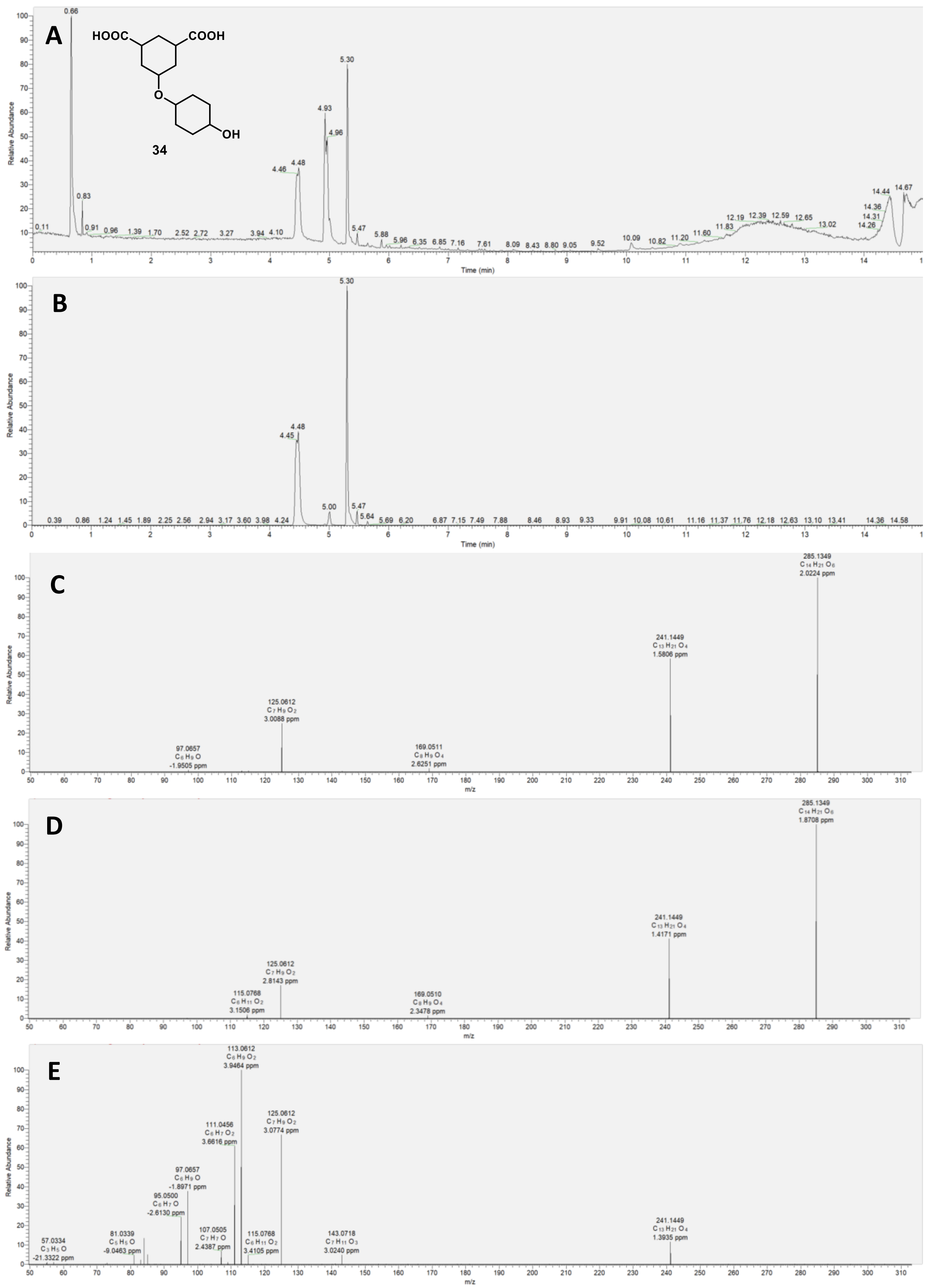
A
HOOC
COOH
O
OH
34
B
C
285.1349
C14 H21 O6
2.0224 ppm
241.1449
C13 H21 O4
1.5806 ppm
125.0612
C7 H9 O2
3.0088 ppm
97.0657
C6 H9 O
-1.9505 ppm
169.0511
C8 H9 O4
2.6251 ppm
D
285.1349
C14 H21 O6
1.8708 ppm
241.1449
C13 H21 O4
1.4171 ppm
125.0612
C7 H9 O2
2.8143 ppm
115.0768
C6 H11 O2
3.1506 ppm
169.0510
C8 H9 O4
2.3478 ppm
E
113.0612
C6 H9 O2
3.9464 ppm
111.0456
C6 H7 O2
3.6616 ppm
125.0612
C7 H9 O2
3.0774 ppm
97.0657
C6 H9 O
-1.8971 ppm
95.0500
C6 H7 O
-2.6130 ppm
57.0334
C3 H5 O
-21.3322 ppm
81.0339
C5 H5 O
-9.0463 ppm
107.0505
C7 H7 O
2.4387 ppm
115.0768
C6 H11 O2
3.4105 ppm
143.0718
C7 H11 O3
3.0240 ppm
241.1449
C13 H21 O4
1.3935 ppm
Relative Abundance
Time (min)
m/z

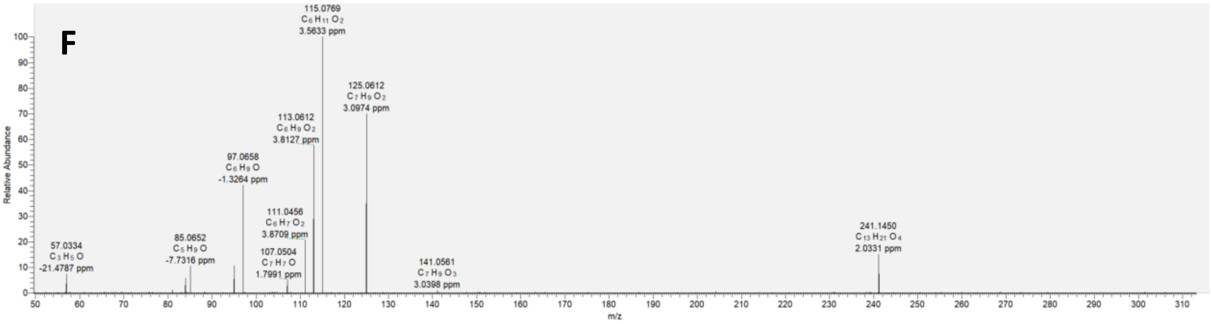

Figure S72: *TIC (A), XIC at 285.1349 m/z (B), HCD35V at 285 nominal mass for isomer 1 (4.45 min, C), HCD35V at 285 nominal mass for isomer 2 (5.30 min, D), HCD75V at 285 nominal mass for isomer 1 (4.45 min, E), and HCD75V at 285 nominal mass for isomer 2 (5.30 min, F), for alcohol* ***34****.*

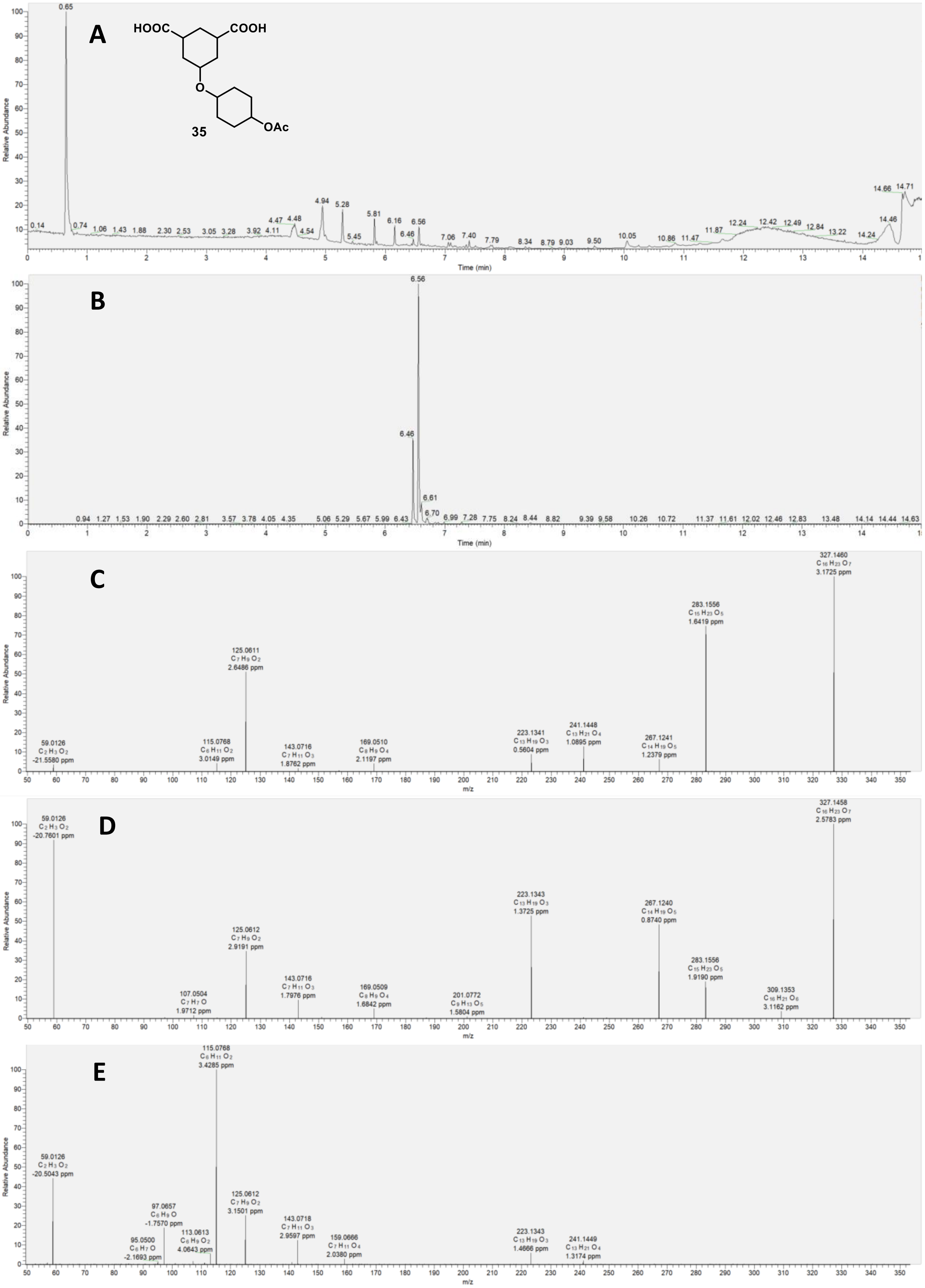
A
HOOC
COOH
O
OAc
35
Relative Abundance
Time (min)
B
C
327.1460
C16 H23 O7
3.1725 ppm
283.1556
C15 H23 O5
1.6419 ppm
125.0611
C7 H9 O2
2.6486 ppm
m/z
D
327.1458
C16 H23 O7
2.5783 ppm
59.0126
C2 H3 O2
-20.7601 ppm
223.1343
C13 H19 O3
1.3725 ppm
267.1240
C14 H19 O5
0.8740 ppm
E
115.0768
C6 H11 O2
3.4285 ppm
59.0126
C2 H3 O2
-20.5043 ppm
97.0657
C6 H9 O
-1.7570 ppm
125.0612
C7 H9 O2
3.1501 ppm
143.0718
C7 H11 O3
2.9597 ppm

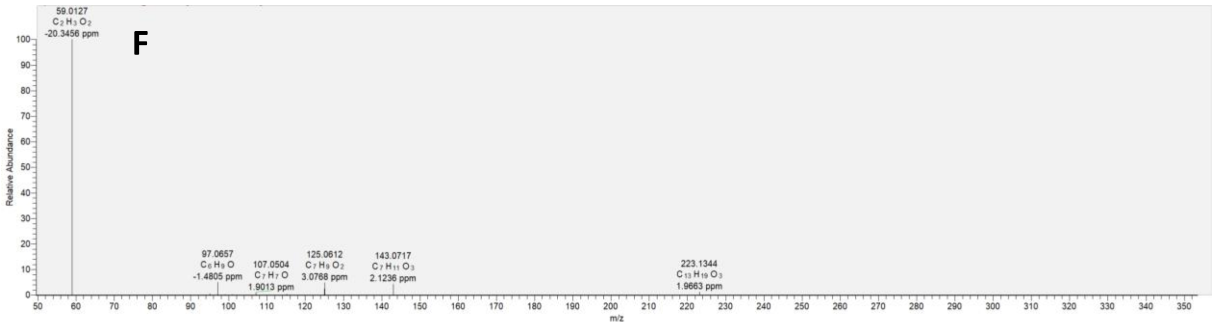

Figure S73: *TIC (A), XIC at 327.1458 m/z (B), HCD35V at 327 nominal mass for isomer 1 (6.46 min, C), HCD35V at 327 nominal mass for isomer 2 (6.56 min, D), HCD75V at 327 nominal mass for isomer 1 (6.46 min, E), and HCD75V at 327 nominal mass for isomer 2 (6.56 min, F), for acetyl ester* ***35****.*

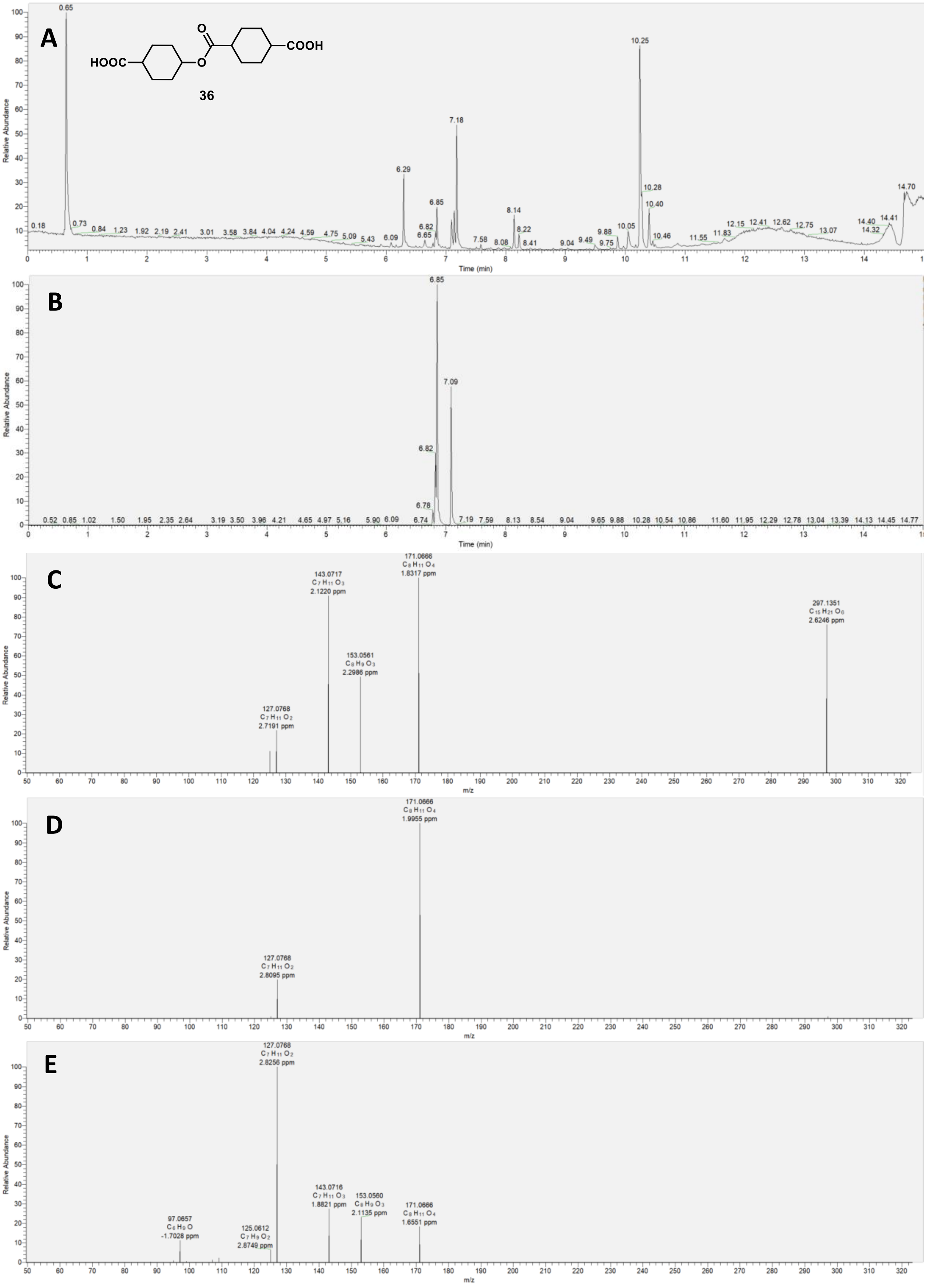
A
HOOC
COOH
O
36
Relative Abundance
Time (min)
B
C
143.0717
$C_7H_{11}O_3$
2.1220 ppm
171.0666
$C_8H_{11}O_4$
1.8317 ppm
297.1351
$C_{15}H_{21}O_6$
2.6246 ppm
153.0561
$C_8H_9O_3$
2.2986 ppm
127.0768
$C_7H_{11}O_2$
2.7191 ppm
m/z
D
171.0666
$C_8H_{11}O_4$
1.9955 ppm
127.0768
$C_7H_{11}O_2$
2.8095 ppm
E
127.0768
$C_7H_{11}O_2$
2.8256 ppm
143.0716
$C_7H_{11}O_3$
1.8821 ppm
153.0560
$C_8H_9O_3$
2.1135 ppm
171.0666
$C_8H_{11}O_4$
1.6551 ppm
97.0657
C_6H_9O
-1.7028 ppm
125.0612
$C_7H_9O_2$
2.8749 ppm

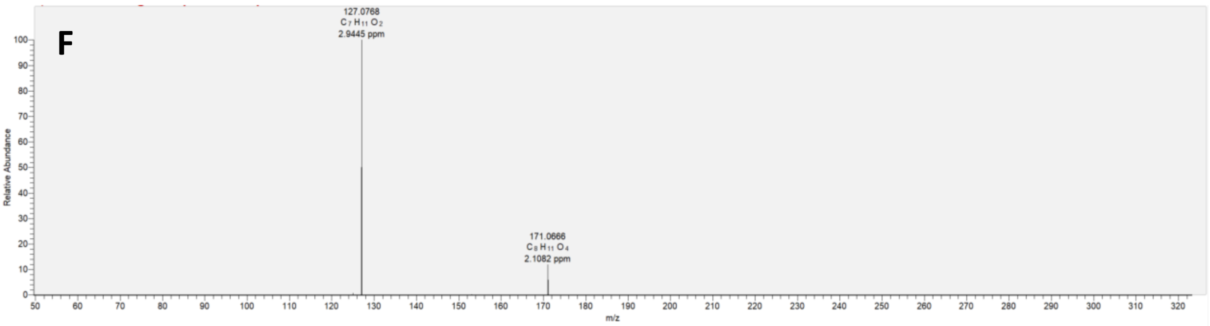

Figure S74: *TIC (A), XIC at 297.1351 m/z (B), HCD35V at 297 nominal mass for isomer 1 (6.85 min, C), HCD35V at 297 nominal mass for isomer 2 (7.09 min, D), HCD75V at 297 nominal mass for isomer 1 (6.85 min, E), and HCD75V at 297 nominal mass for isomer 2 (7.09 min, F), for dicarboxylic acid* ***36****.*

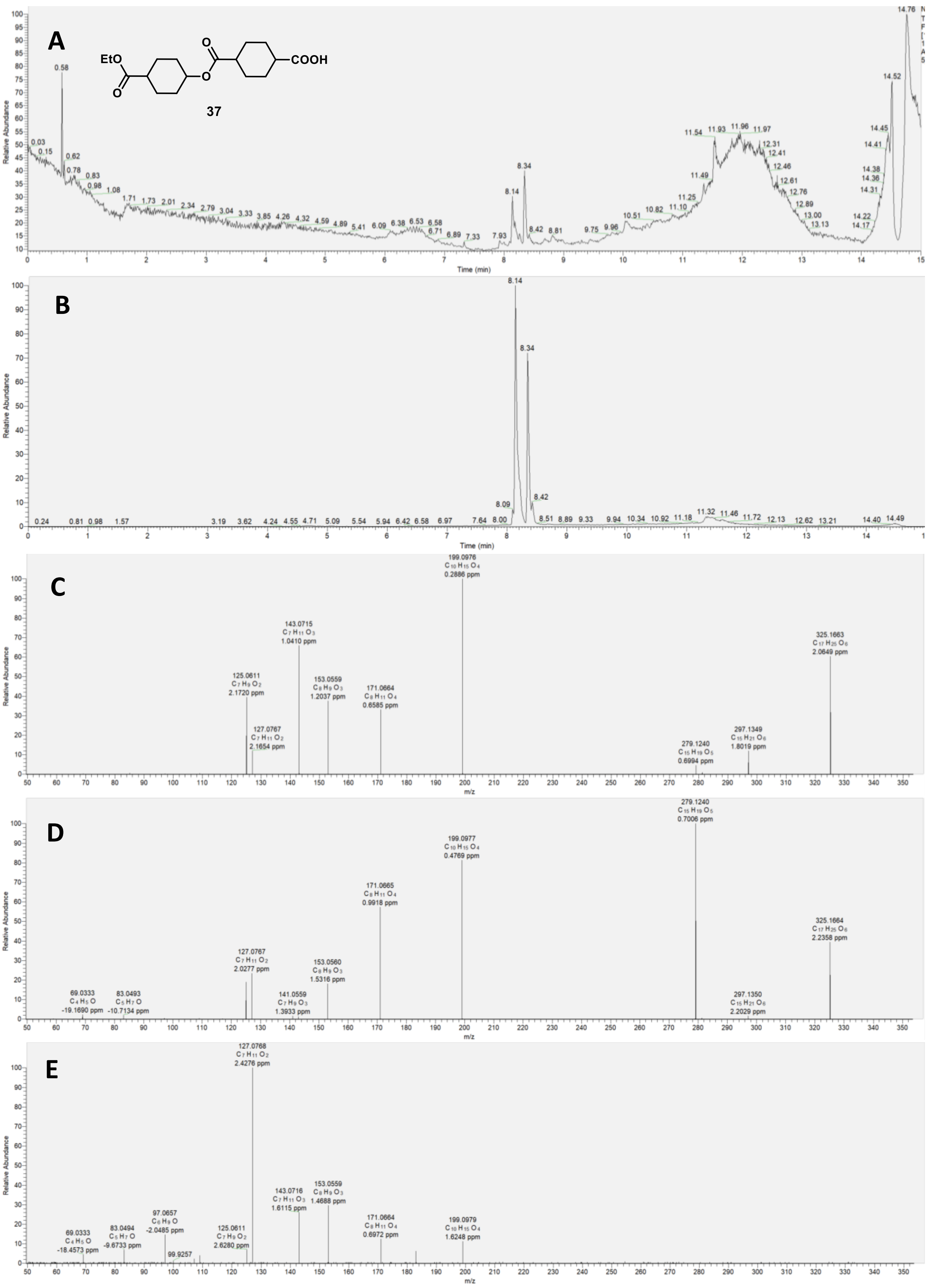
A
EtO
O
O
O
COOH
37
Relative Abundance
Time (min)
B
C
199.0976
C10 H15 O4
0.2886 ppm
143.0715
C7 H11 O3
1.0410 ppm
125.0611
C7 H9 O2
2.1720 ppm
127.0767
C7 H11 O2
2.1654 ppm
153.0559
C8 H9 O3
1.2037 ppm
171.0664
C8 H11 O4
0.6585 ppm
279.1240
C15 H19 O5
0.6994 ppm
297.1349
C15 H21 O6
1.8019 ppm
325.1663
C17 H25 O6
2.0649 ppm
m/z
D
279.1240
C15 H19 O5
0.7006 ppm
199.0977
C10 H15 O4
0.4769 ppm
171.0665
C8 H11 O4
0.9918 ppm
127.0767
C7 H11 O2
2.0277 ppm
153.0560
C8 H9 O3
1.5316 ppm
141.0559
C7 H9 O3
1.3933 ppm
69.0333
C4 H5 O
-19.1690 ppm
83.0493
C5 H7 O
-10.7134 ppm
297.1350
C15 H21 O6
2.2029 ppm
325.1664
C17 H25 O6
2.2358 ppm
E
127.0768
C7 H11 O2
2.4276 ppm
143.0716
C7 H11 O3
1.6115 ppm
153.0559
C8 H9 O3
1.4688 ppm
97.0657
C6 H9 O
-2.0485 ppm
69.0333
C4 H5 O
-18.4573 ppm
83.0494
C5 H7 O
-9.6733 ppm
125.0611
C7 H9 O2
2.6280 ppm
99.9257
171.0664
C8 H11 O4
0.6972 ppm
199.0979
C10 H15 O4
1.6248 ppm

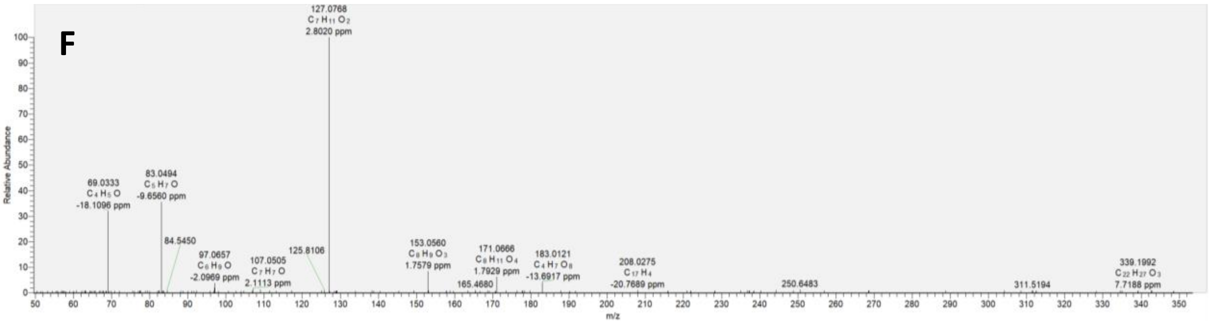

Figure S75: *TIC (A), XIC at 325.1663 m/z (B), HCD35V at 325 nominal mass for isomer 1 (8.14 min, C), HCD35V at 325 nominal mass for isomer 2 (8.34 min, D), HCD75V at 325 nominal mass for isomer 1 (8.14 min, E), and HCD75V at 325 nominal mass for isomer 2 (8.34 min, F), for carboxylic acid* ***37****.*

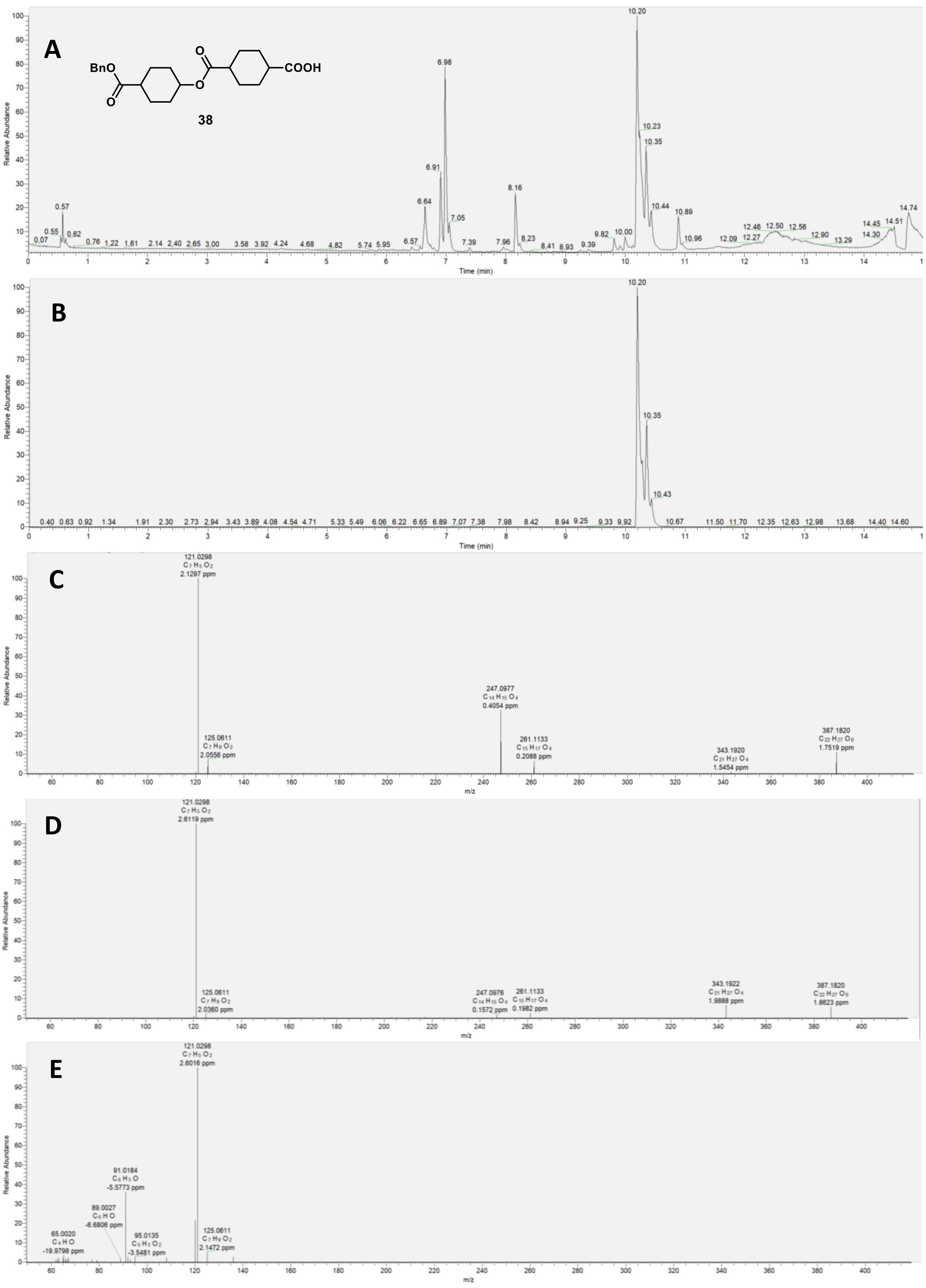
A
BnO
O
O
O
COOH
38
Relative Abundance
Time (min)
10.20
6.98
10.23
10.35
6.91
8.16
6.64
7.05
10.44
10.89
0.57
14.74
B
10.20
10.35
10.43
C
121.0298
C7 H5 O2
2.1297 ppm
247.0977
C14 H15 O4
0.4054 ppm
125.0611
C7 H9 O2
2.0556 ppm
261.1133
C15 H17 O4
0.2088 ppm
343.1920
C21 H27 O4
1.5454 ppm
387.1820
C22 H27 O6
1.7519 ppm
m/z
D
121.0298
C7 H5 O2
2.6119 ppm
125.0611
C7 H9 O2
2.0360 ppm
247.0976
C14 H15 O4
0.1572 ppm
261.1133
C15 H17 O4
0.1982 ppm
343.1922
C21 H27 O4
1.9888 ppm
387.1820
C22 H27 O6
1.8623 ppm
E
121.0298
C7 H5 O2
2.6016 ppm
91.0184
C6 H3 O
-5.5773 ppm
89.0027
C6 H O
-6.6806 ppm
65.0020
C4 H O
-19.9798 ppm
95.0135
C5 H3 O2
-3.5481 ppm
125.0611
C7 H9 O2
2.1472 ppm

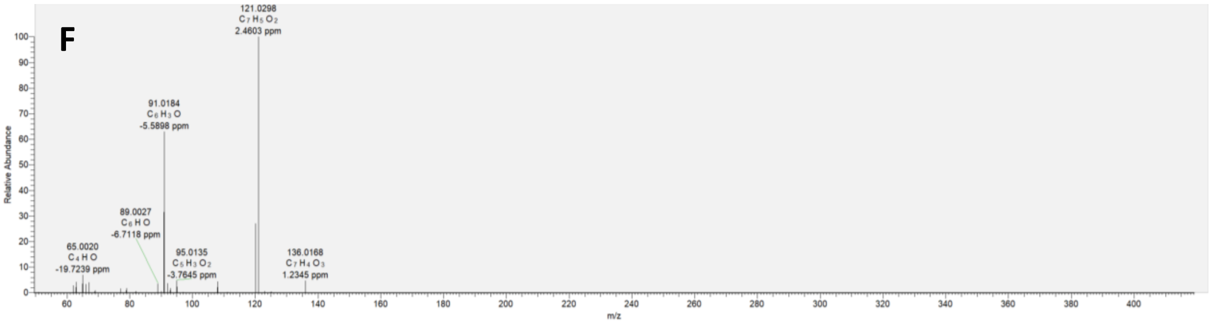

Figure S76: *TIC (A), XIC at 387.1820 m/z (B), HCD35V at 387 nominal mass for isomer 1 (10.19 min, C), HCD35V at 387 nominal mass for isomer 2 (10.35 min, D), HCD75V at 387 nominal mass for isomer 1 (10.19 min, E), and HCD75V at 387 nominal mass for isomer 2 (10.35 min, F), for carboxylic acid* ***38****.*

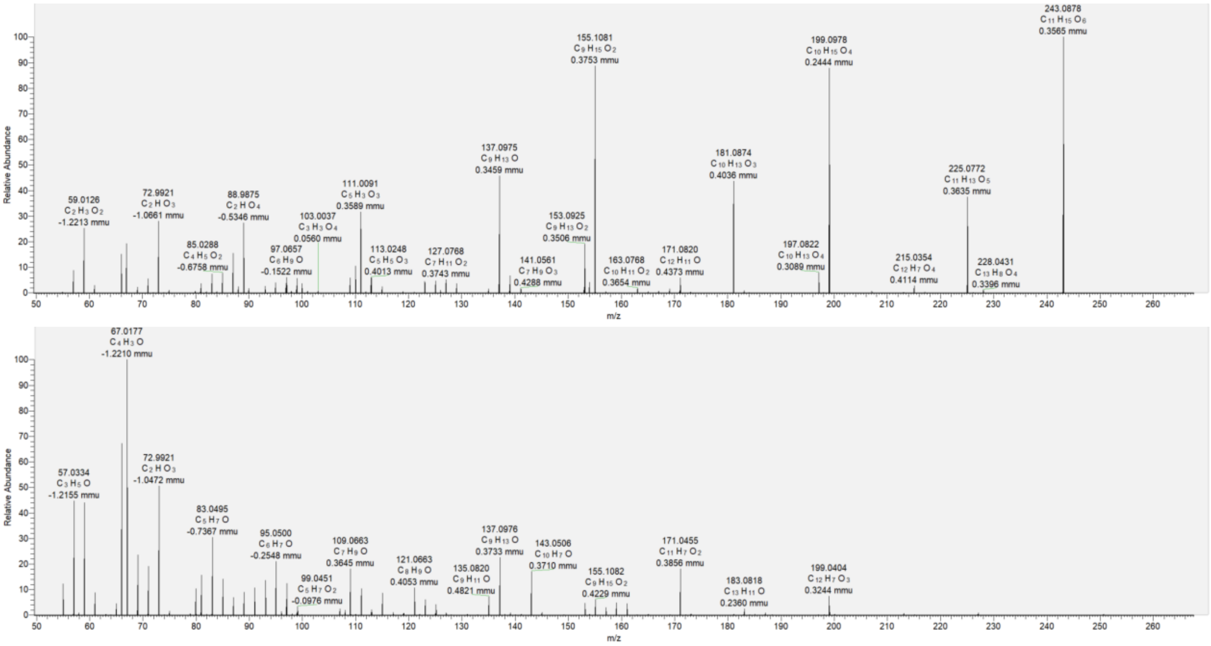

Figure S77: *Top: 35V HCD Fragmentation data from 3-9 minutes in LC for SRFA Nominal Mass 243. Bottom: 75V HCD Fragmentation data from 3-9 minutes in LC for SRFA Nominal Mass 243.*

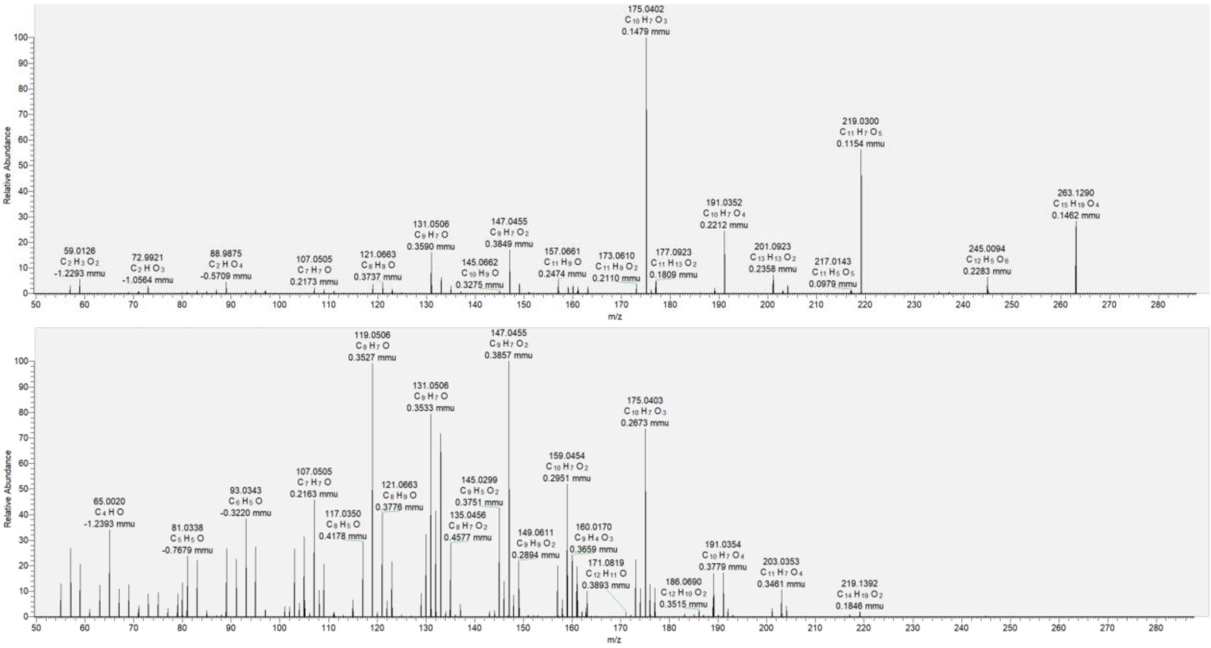

Figure S78: *Top: 35V HCD Fragmentation data from 3-9 minutes in LC for SRFA Nominal Mass 263. Bottom: 75V HCD Fragmentation data from 3-9 minutes in LC for SRFA Nominal Mass 263.*

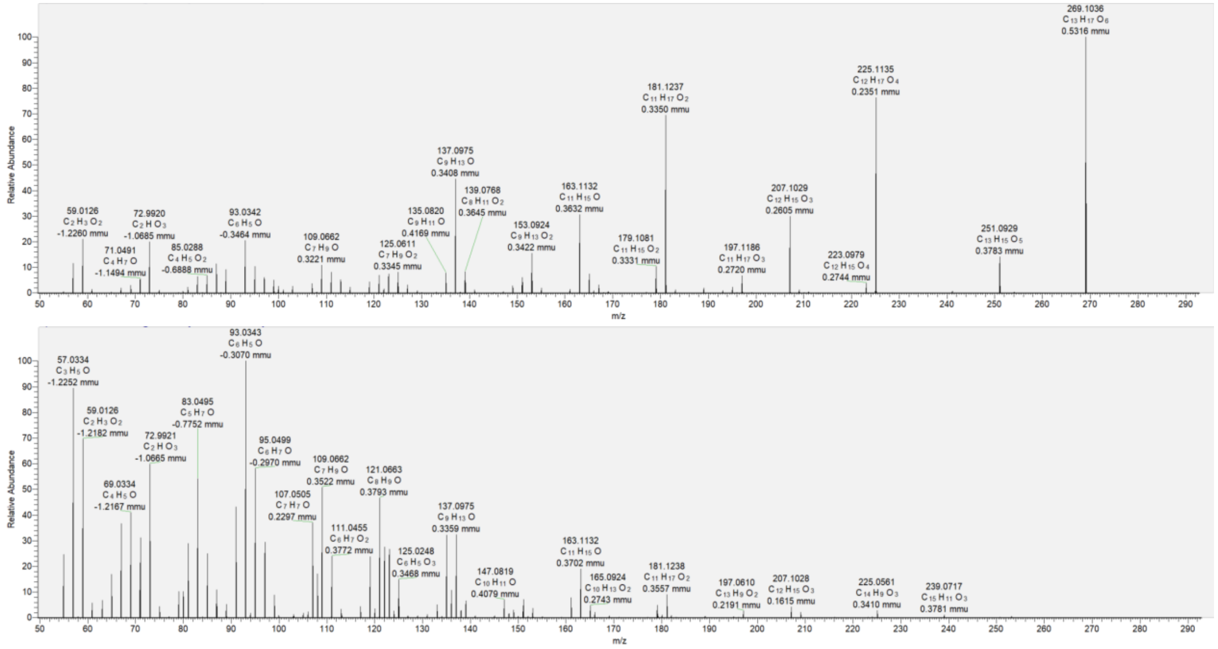

Figure S79: *Top: 35V HCD Fragmentation data from 3-9 minutes in LC for SRFA Nominal Mass 269. Bottom: 75V HCD Fragmentation data from 3-9 minutes in LC for SRFA Nominal Mass 269.*

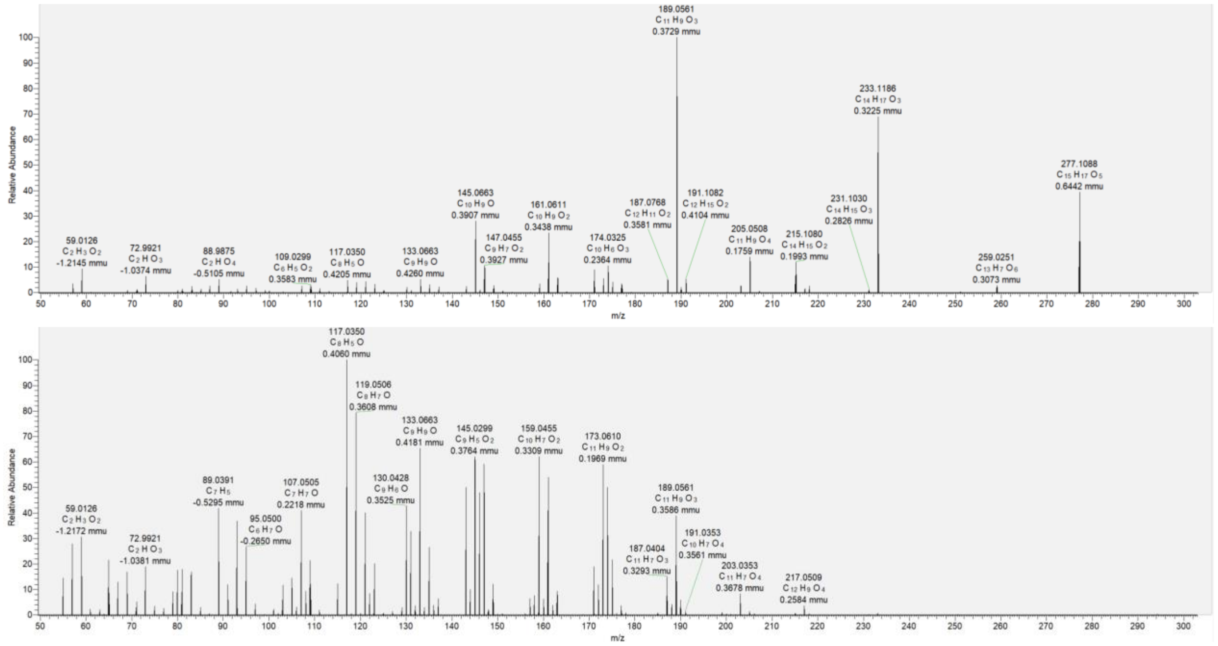

Figure S80: *Top: 35V HCD Fragmentation data from 3-9 minutes in LC for SRFA Nominal Mass 277. Bottom: 75V HCD Fragmentation data from 3-9 minutes in LC for SRFA Nominal Mass 277.*

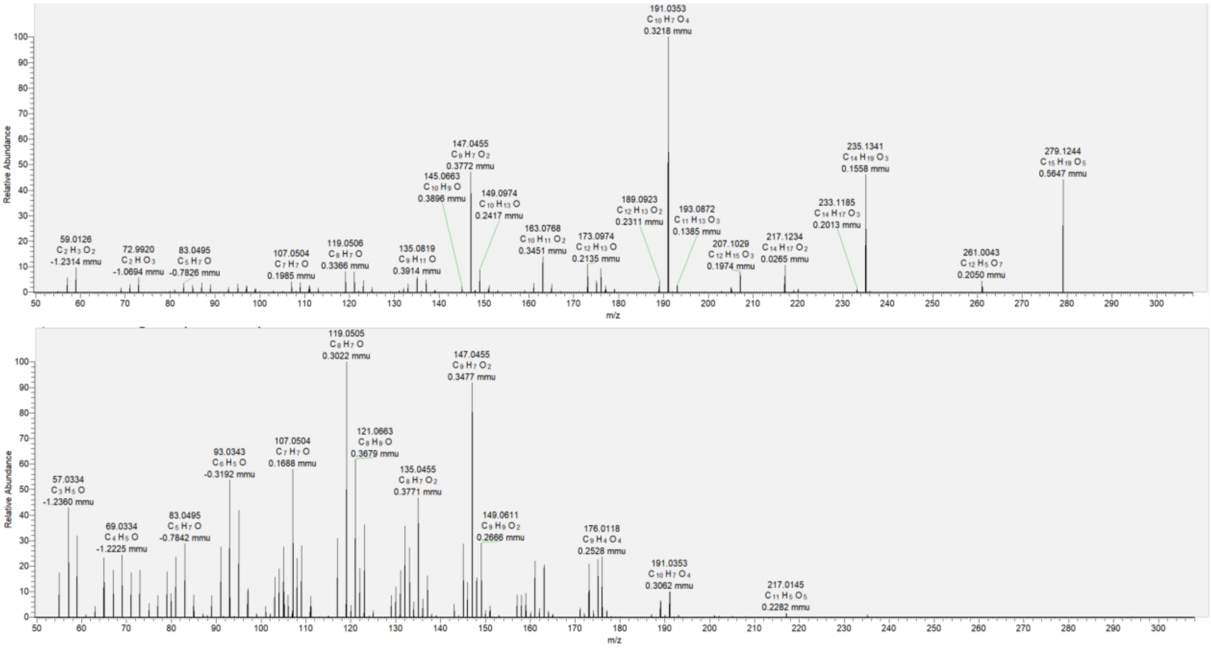

Figure S81: *Top: 35V HCD Fragmentation data from 3-9 minutes in LC for SRFA Nominal Mass 279. Bottom: 75V HCD Fragmentation data from 3-9 minutes in LC for SRFA Nominal Mass 279.*

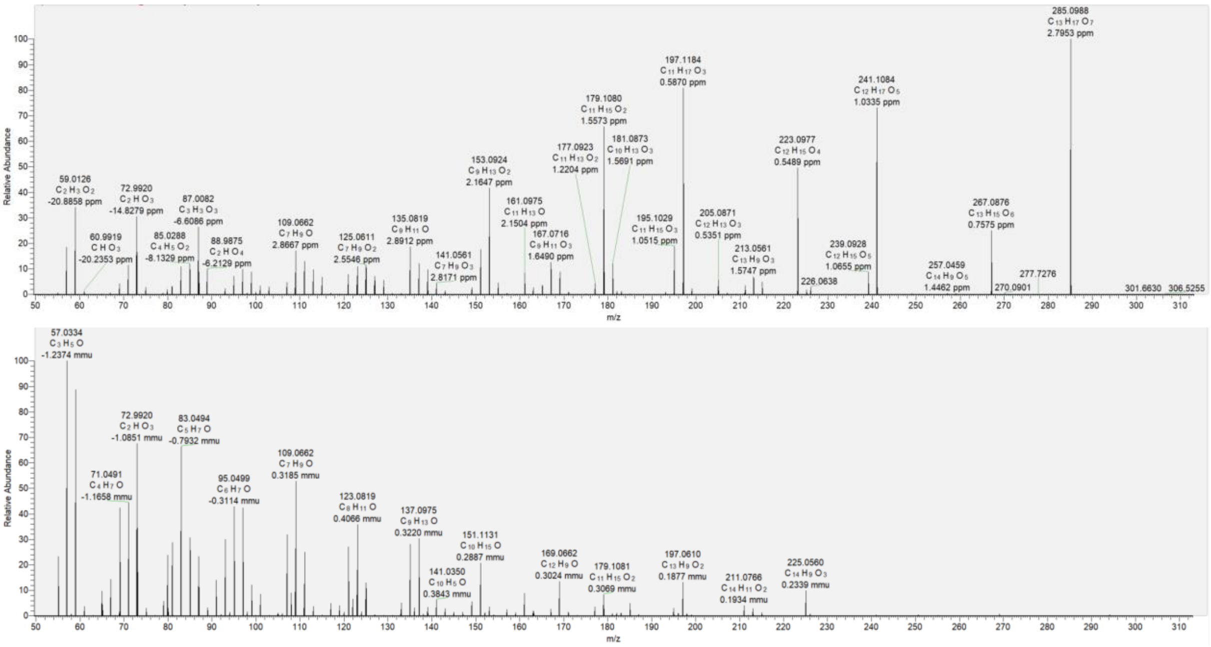

Figure S82: *Top: 35V HCD Fragmentation data from 3-9 minutes in LC for SRFA Nominal Mass 285. Bottom: 75V HCD Fragmentation data from 3-9 minutes in LC for SRFA Nominal Mass 285.*

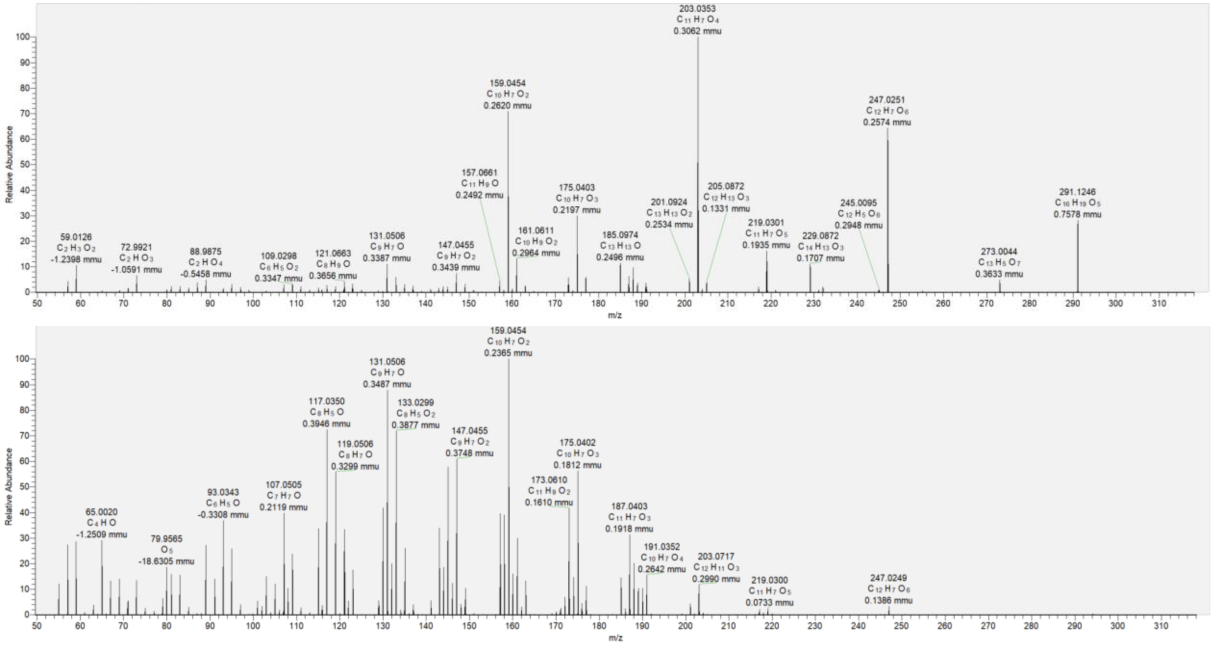

Figure S83: *Top: 35V HCD Fragmentation data from 3-9 minutes in LC for SRFA Nominal Mass 291. Bottom: 75V HCD Fragmentation data from 3-9 minutes in LC for SRFA Nominal Mass 291.*

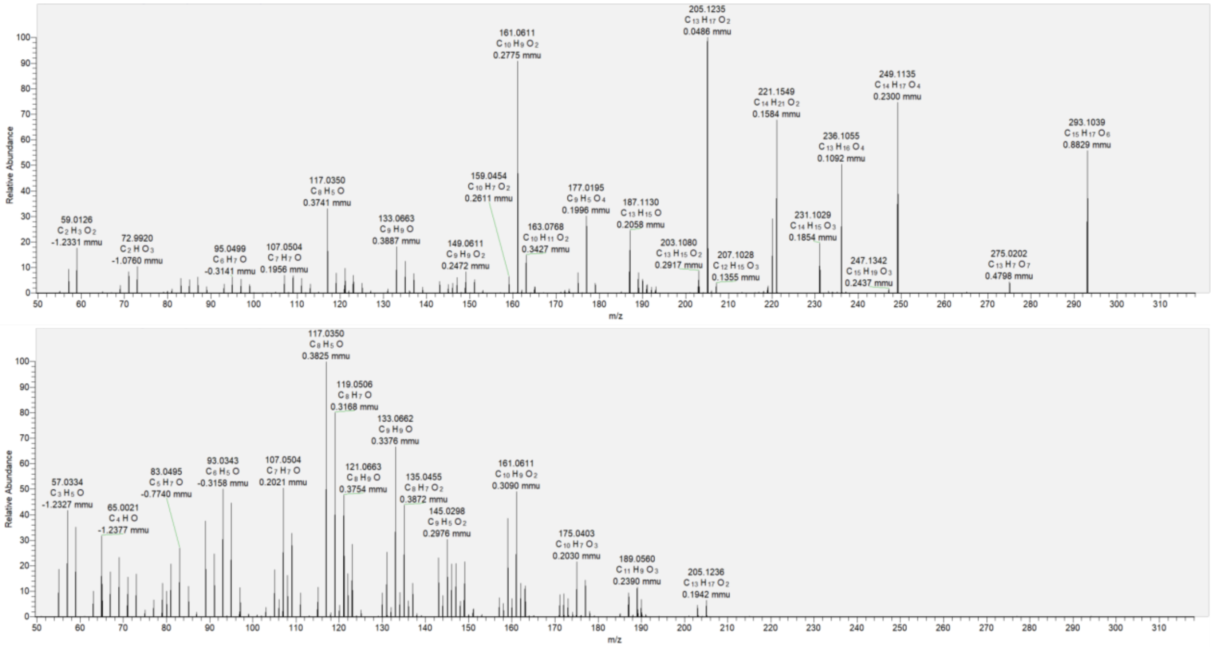

Figure S84: *Top: 35V HCD Fragmentation data from 3-9 minutes in LC for SRFA Nominal Mass 293. Bottom: 75V HCD Fragmentation data from 3-9 minutes in LC for SRFA Nominal Mass 293.*

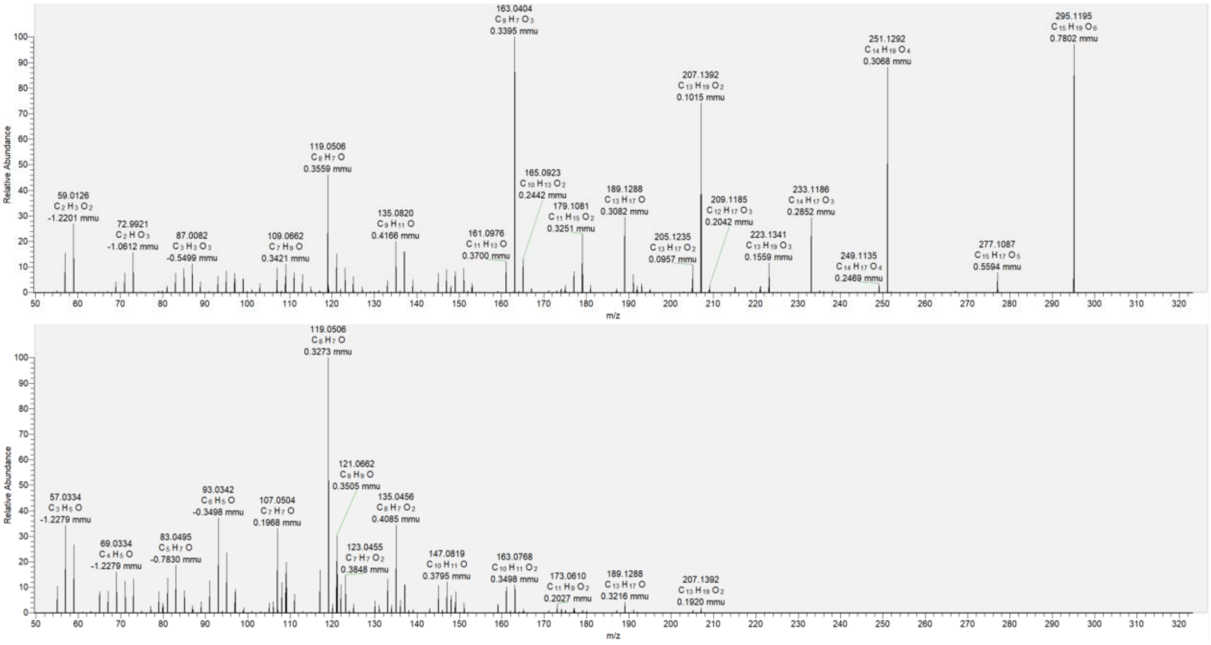

Figure S85: *Top: 35V HCD Fragmentation data from 3-9 minutes in LC for SRFA Nominal Mass 295. Bottom: 75V HCD Fragmentation data from 3-9 minutes in LC for SRFA Nominal Mass 295.*

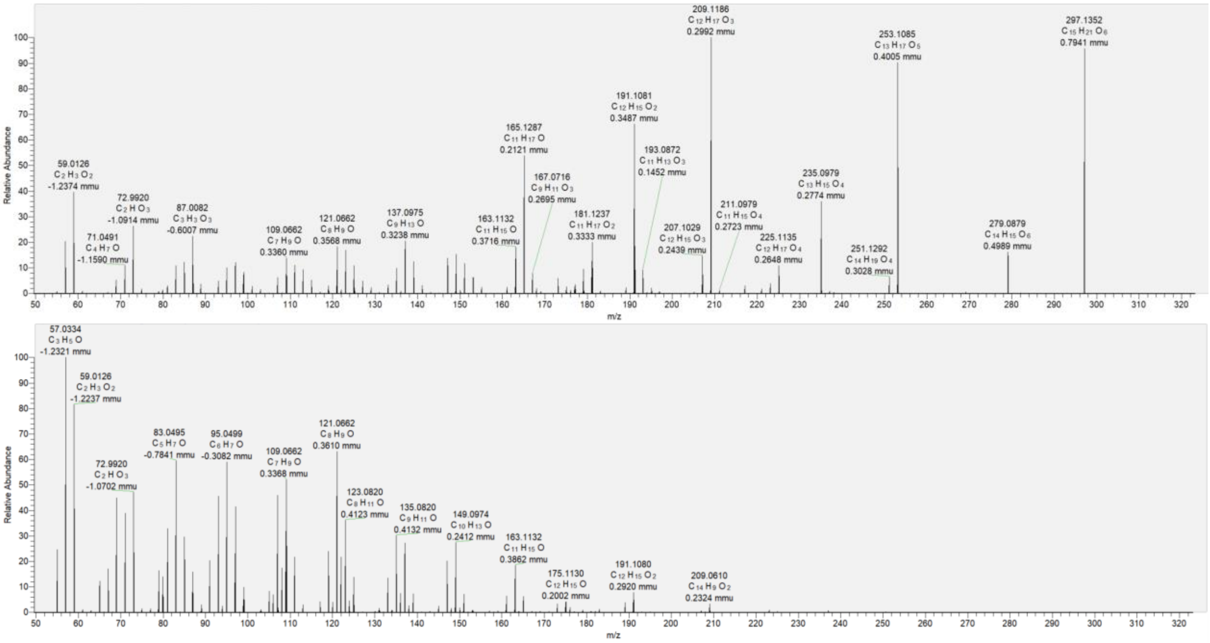

Figure S86: *Top: 35V HCD Fragmentation data from 3-9 minutes in LC for SRFA Nominal Mass 297. Bottom: 75V HCD Fragmentation data from 3-9 minutes in LC for SRFA Nominal Mass 297.*

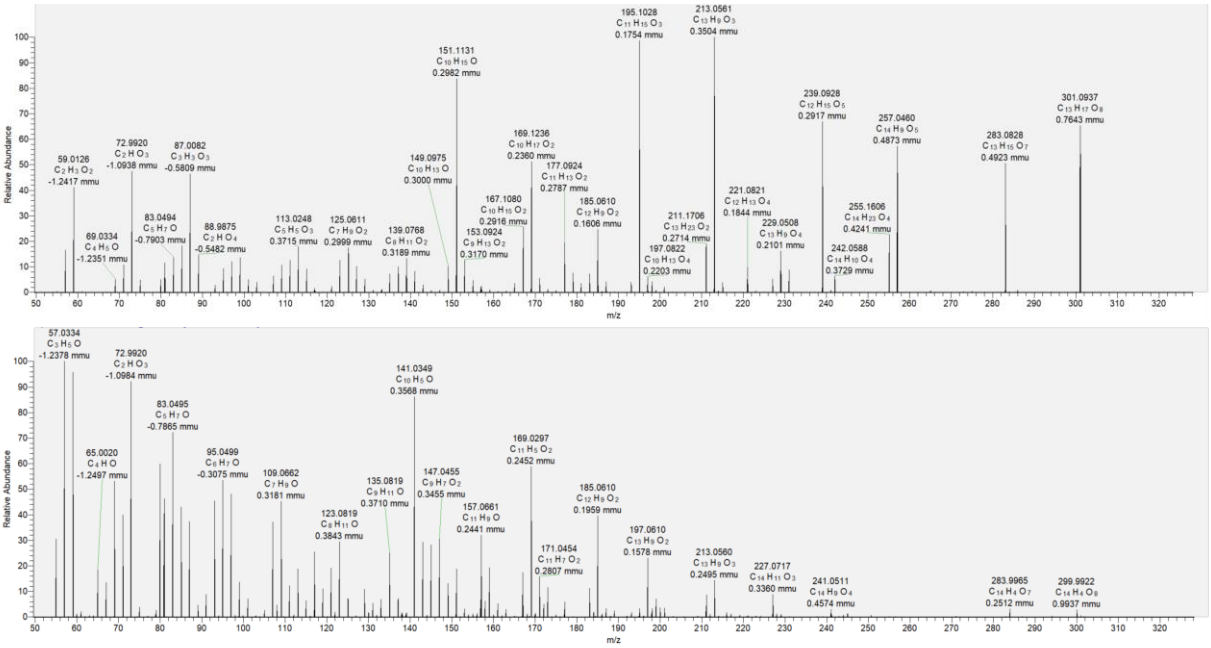

Figure S87: *Top: 35V HCD Fragmentation data from 3-9 minutes in LC for SRFA Nominal Mass 301. Bottom: 75V HCD Fragmentation data from 3-9 minutes in LC for SRFA Nominal Mass 301.*

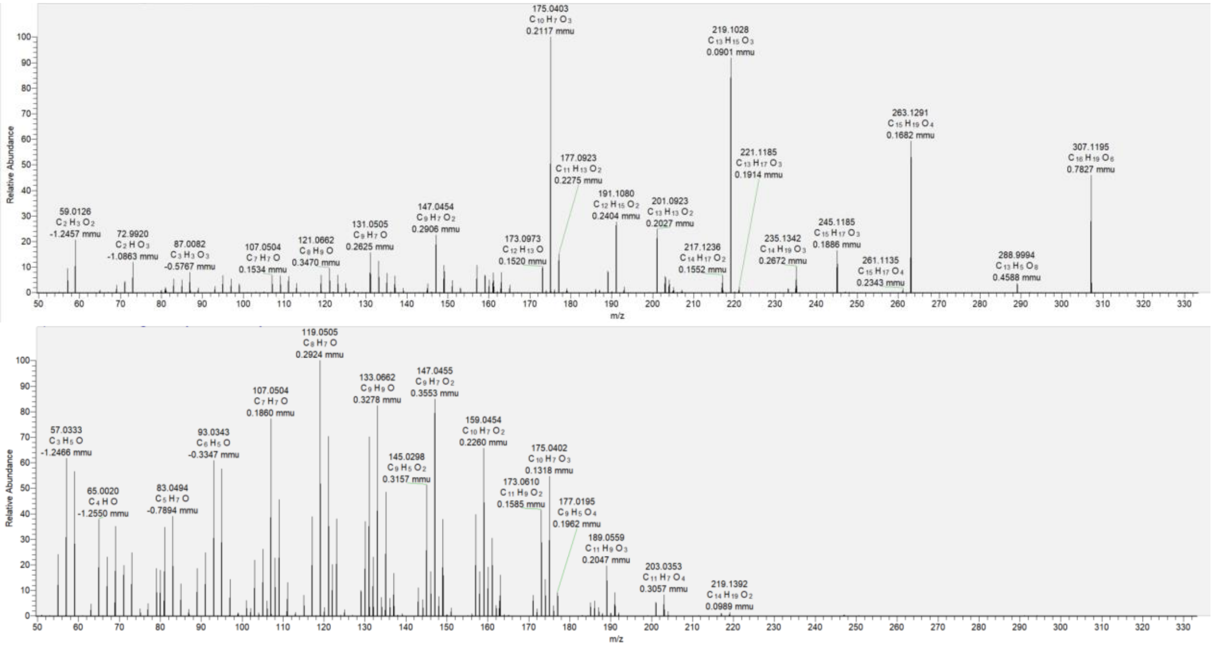

Figure S88: *Top: 35V HCD Fragmentation data from 3-9 minutes in LC for SRFA Nominal Mass 307. Bottom: 75V HCD Fragmentation data from 3-9 minutes in LC for SRFA Nominal Mass 307.*

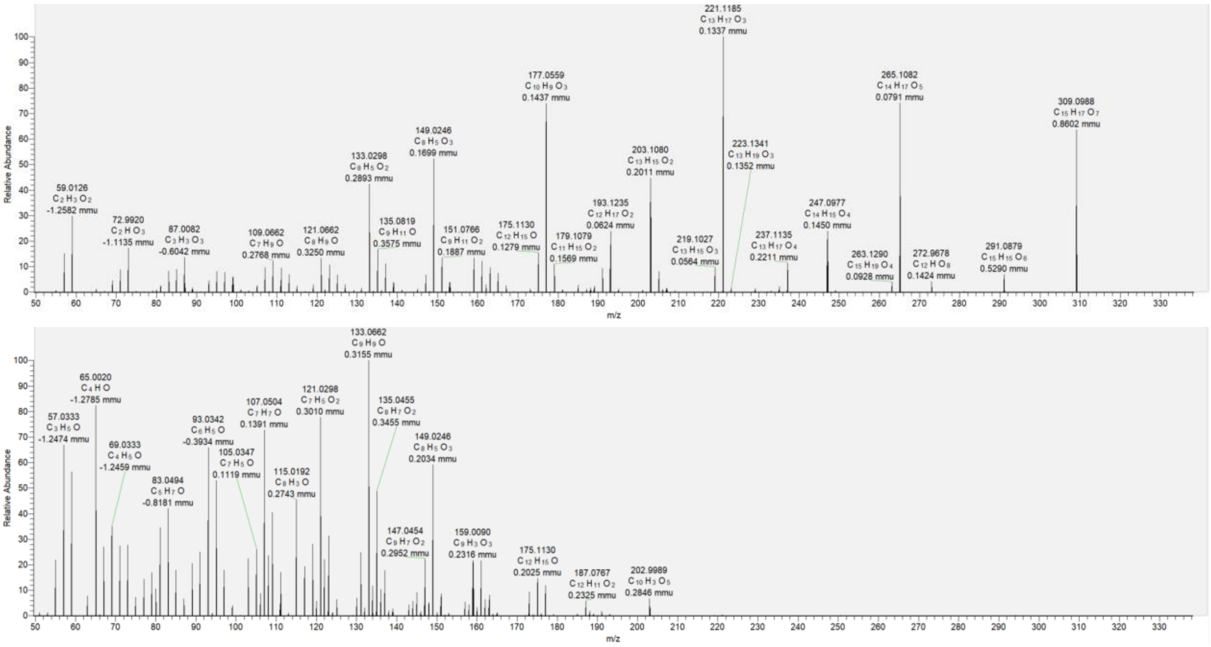

Figure S89: *Top: 35V HCD Fragmentation data from 3-9 minutes in LC for SRFA Nominal Mass 309. Bottom: 75V HCD Fragmentation data from 3-9 minutes in LC for SRFA Nominal Mass 309.*

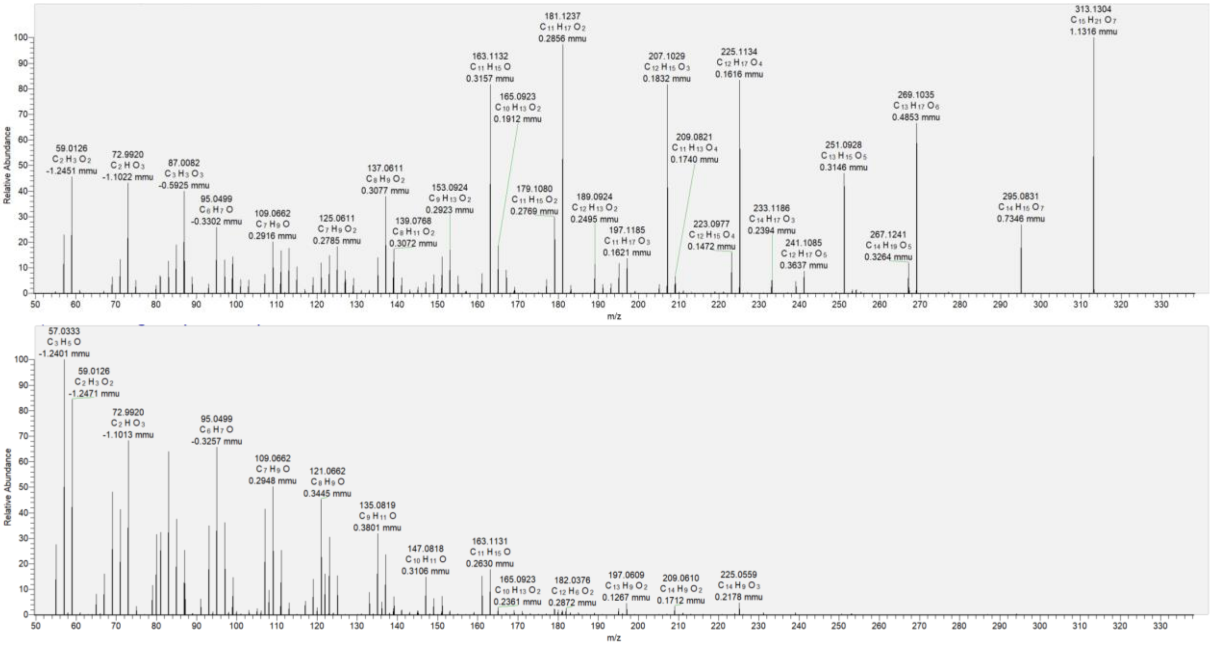

Figure S90: *Top: 35V HCD Fragmentation data from 3-9 minutes in LC for SRFA Nominal Mass 313. Bottom: 75V HCD Fragmentation data from 3-9 minutes in LC for SRFA Nominal Mass 313.*

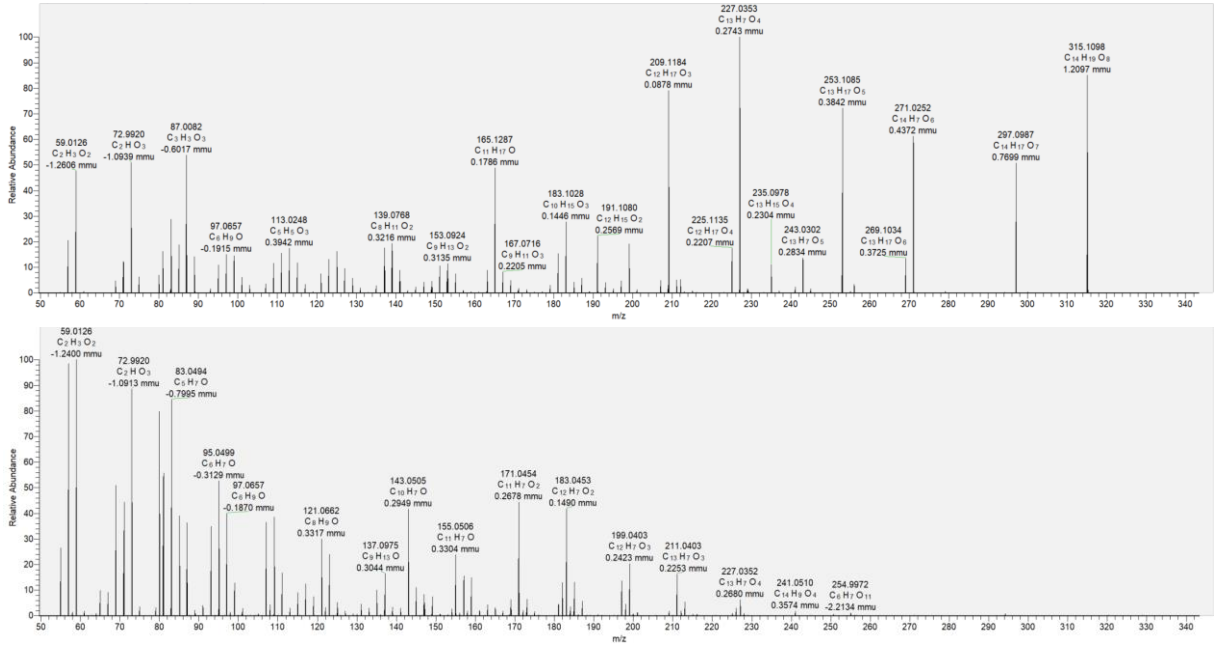

Figure S91: *Top: 35V HCD Fragmentation data from 3-9 minutes in LC for SRFA Nominal Mass 315. Bottom: 75V HCD Fragmentation data from 3-9 minutes in LC for SRFA Nominal Mass 315.*

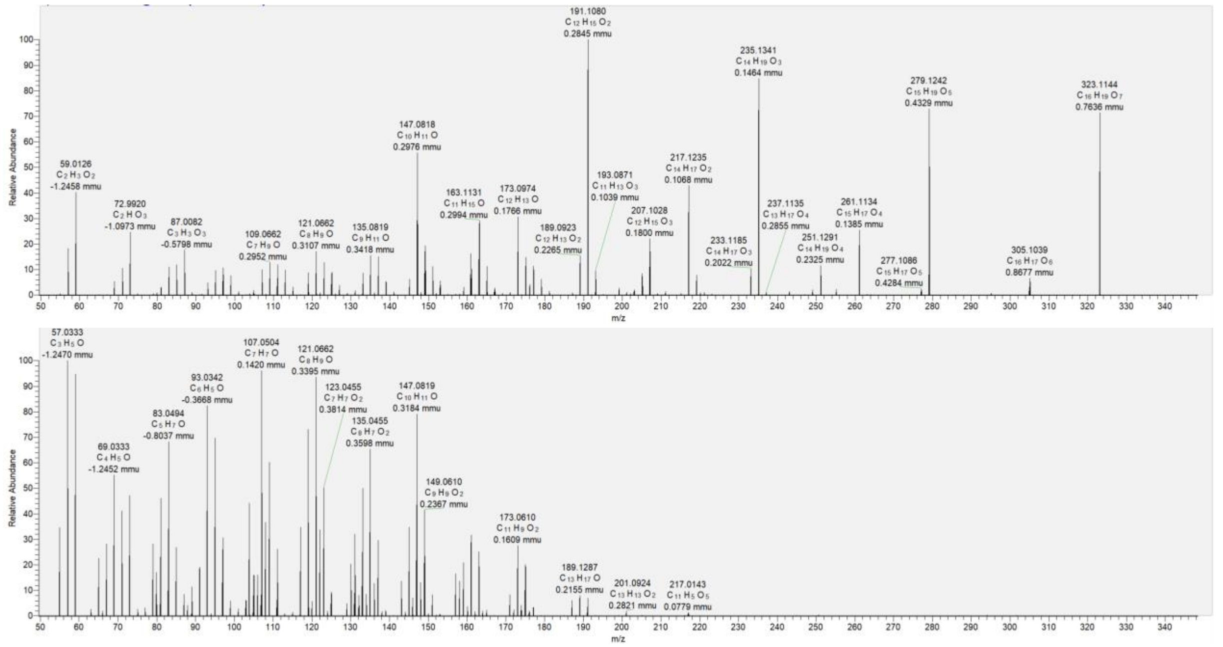

Figure S92: *Top: 35V HCD Fragmentation data from 3-9 minutes in LC for SRFA Nominal Mass 323. Bottom: 75V HCD Fragmentation data from 3-9 minutes in LC for SRFA Nominal Mass 323.*

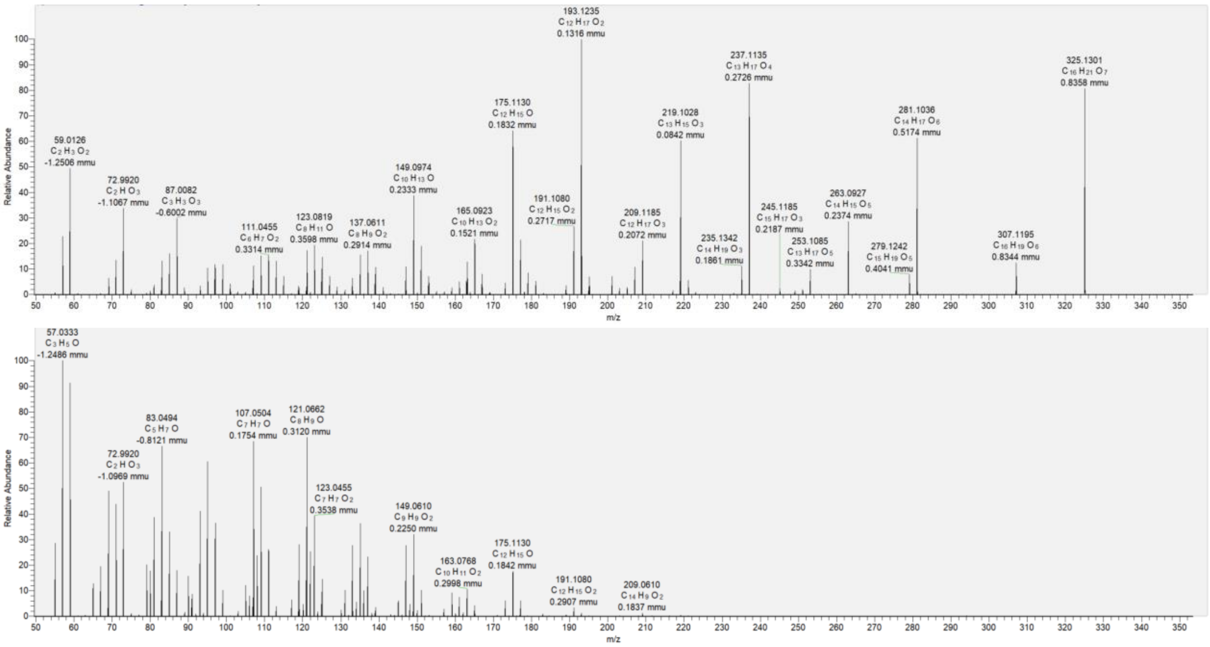

Figure S93: *Top: 35V HCD Fragmentation data from 3-9 minutes in LC for SRFA Nominal Mass 325. Bottom: 75V HCD Fragmentation data from 3-9 minutes in LC for SRFA Nominal Mass 325.*

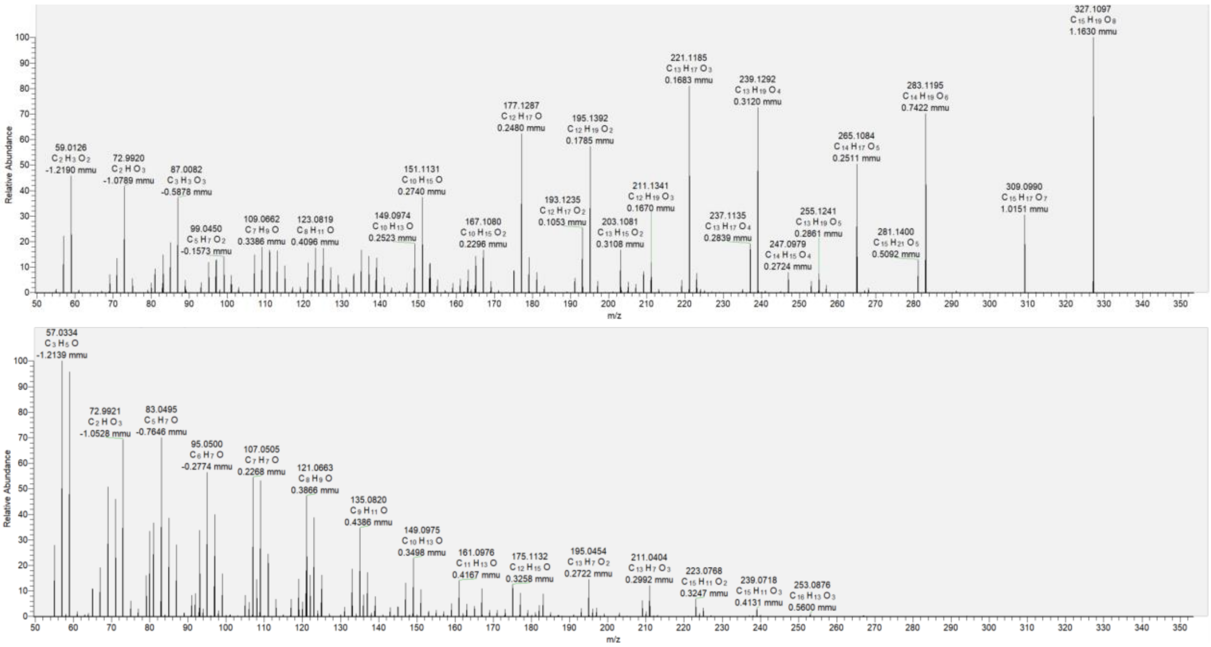

Figure S94: *Top: 35V HCD Fragmentation data from 3-9 minutes in LC for SRFA Nominal Mass 327. Bottom: 75V HCD Fragmentation data from 3-9 minutes in LC for SRFA Nominal Mass 327.*

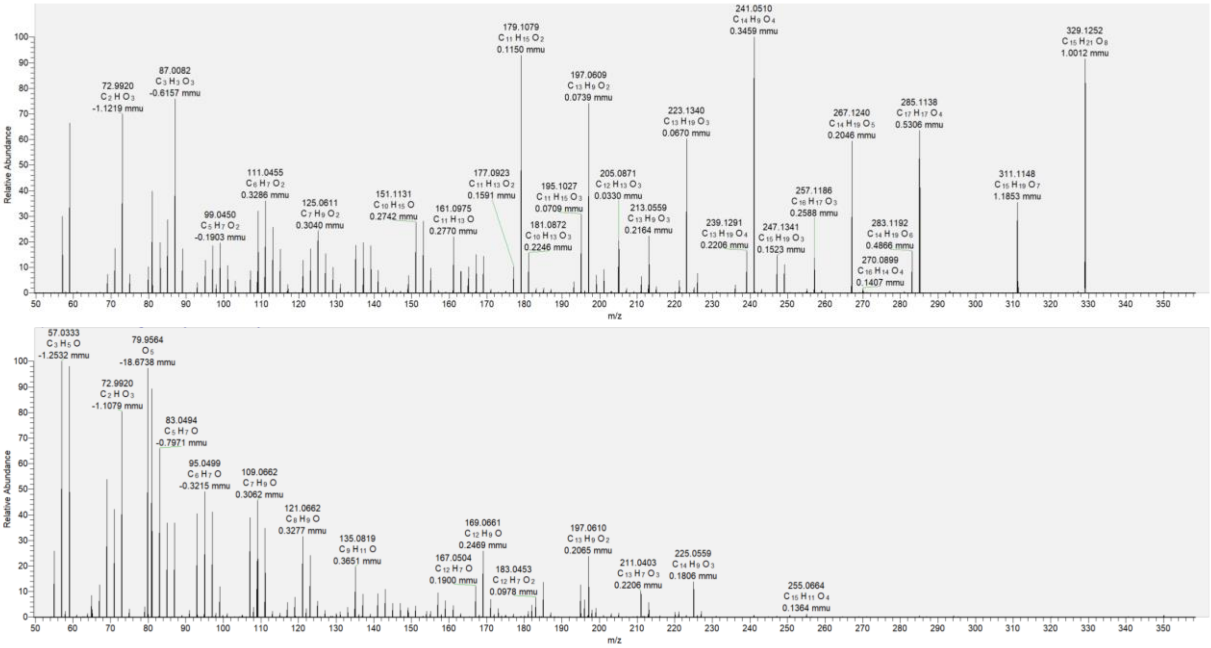

Figure S95: *Top: 35V HCD Fragmentation data from 3-9 minutes in LC for SRFA Nominal Mass 329. Bottom: 75V HCD Fragmentation data from 3-9 minutes in LC for SRFA Nominal Mass 329.*

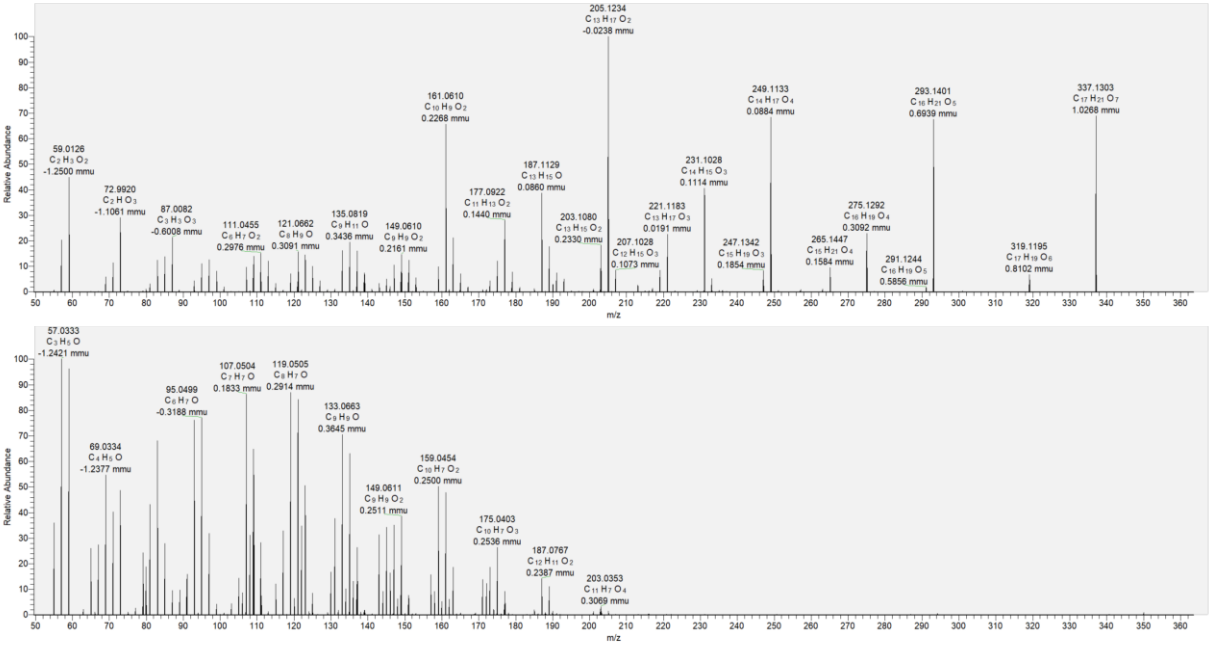

Figure S96: *Top: 35V HCD Fragmentation data from 3-9 minutes in LC for SRFA Nominal Mass 337. Bottom: 75V HCD Fragmentation data from 3-9 minutes in LC for SRFA Nominal Mass 337.*

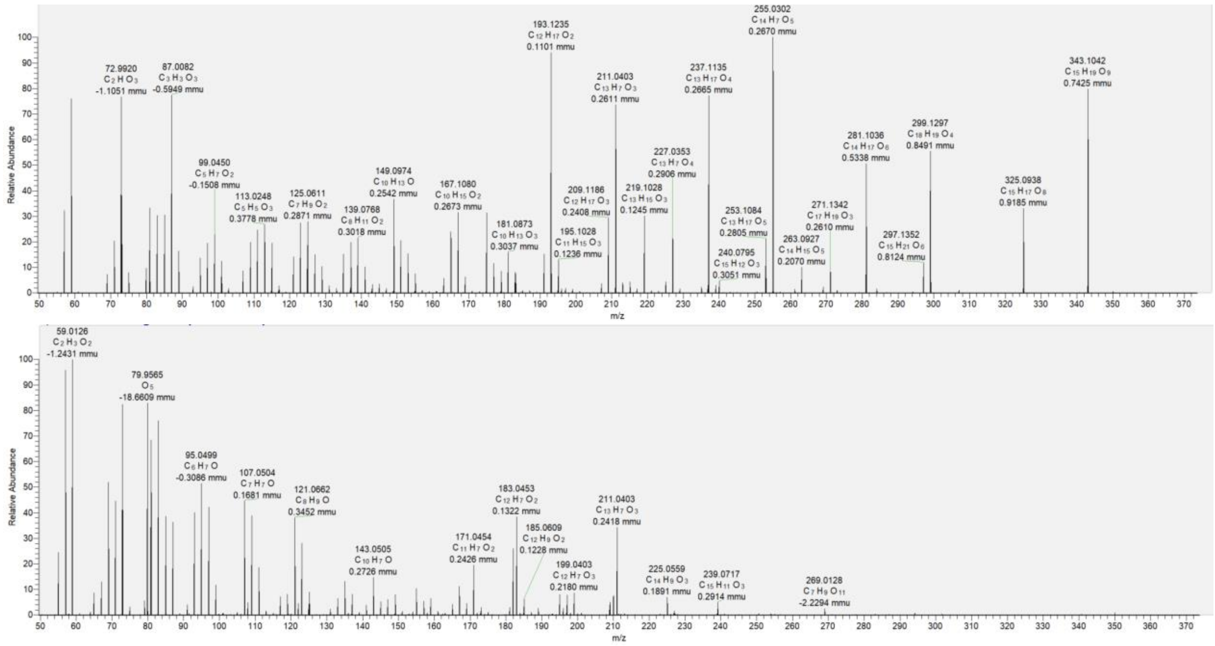

Figure S97: *Top: 35V HCD Fragmentation data from 3-9 minutes in LC for SRFA Nominal Mass 343. Bottom: 75V HCD Fragmentation data from 3-9 minutes in LC for SRFA Nominal Mass 343.*

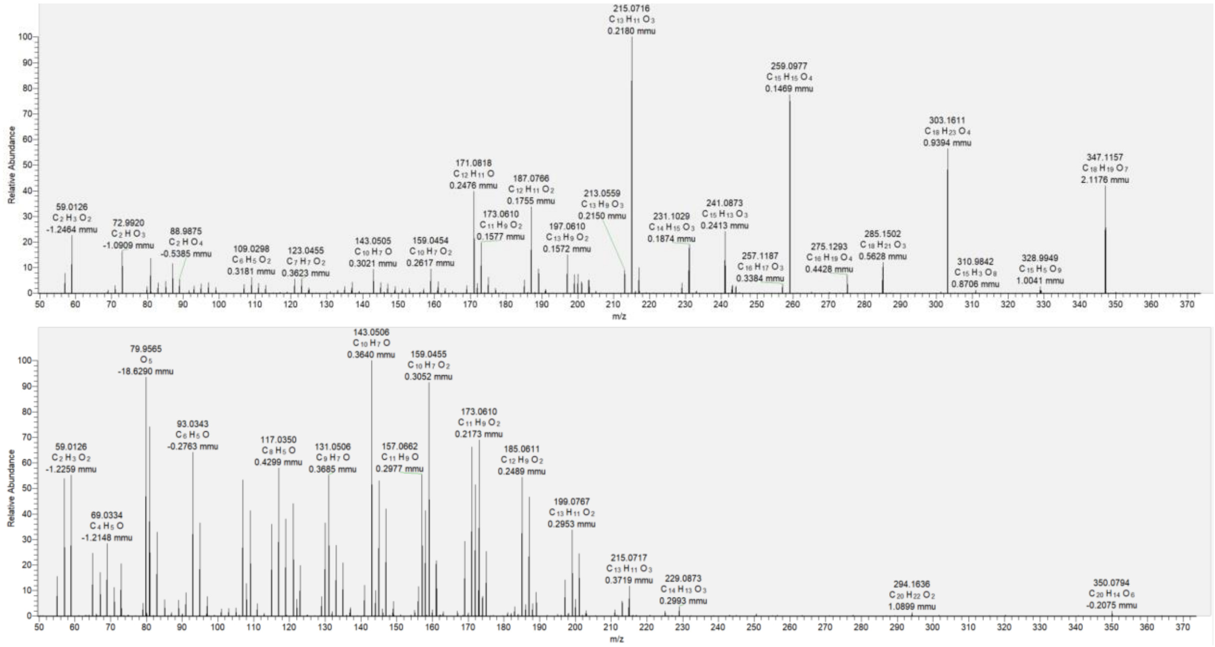

Figure S98: *Top: 35V HCD Fragmentation data from 3-9 minutes in LC for SRFA Nominal Mass 347. Bottom: 75V HCD Fragmentation data from 3-9 minutes in LC for SRFA Nominal Mass 347.*

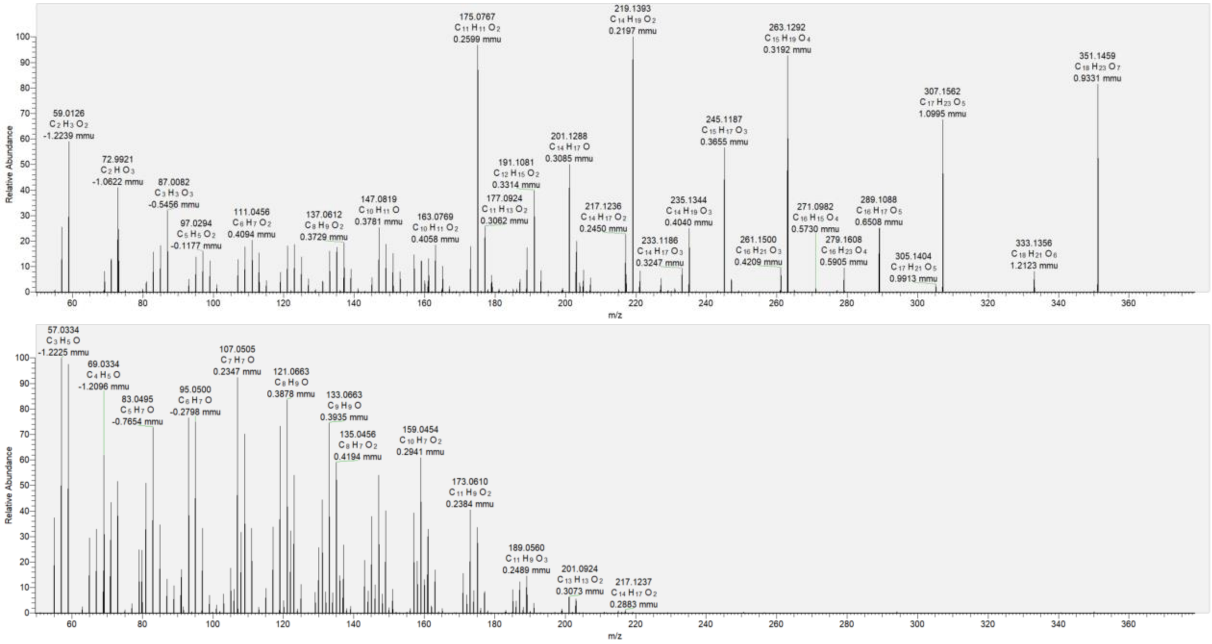

Figure S99: *Top: 35V HCD Fragmentation data from 3-9 minutes in LC for SRFA Nominal Mass 351. Bottom: 75V HCD Fragmentation data from 3-9 minutes in LC for SRFA Nominal Mass 351.*

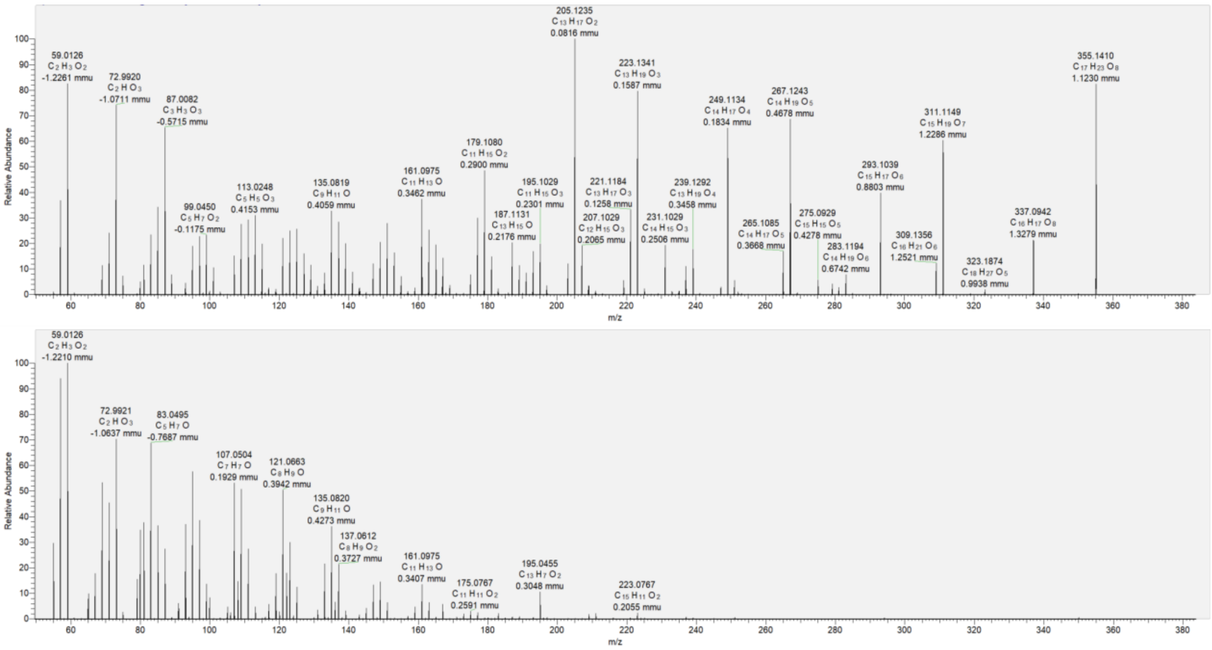

Figure S100: *Top: 35V HCD Fragmentation data from 3-9 minutes in LC for SRFA Nominal Mass 355. Bottom: 75V HCD Fragmentation data from 3-9 minutes in LC for SRFA Nominal Mass 355.*

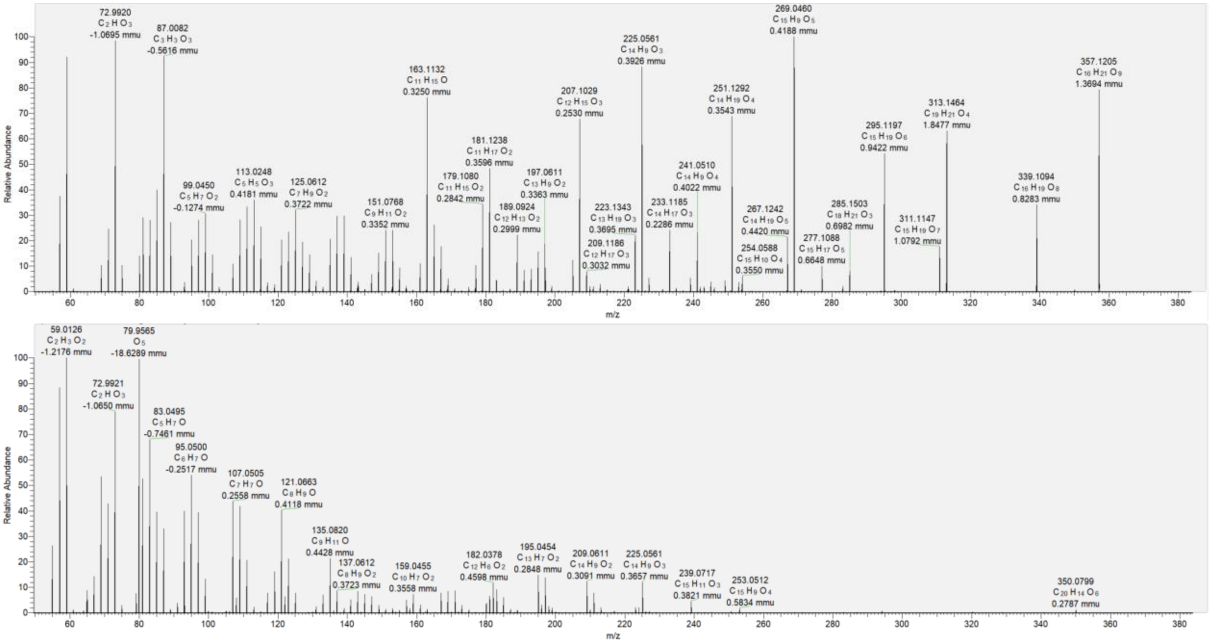

Figure S101: *Top: 35V HCD Fragmentation data from 3-9 minutes in LC for SRFA Nominal Mass 357. Bottom: 75V HCD Fragmentation data from 3-9 minutes in LC for SRFA Nominal Mass 357.*

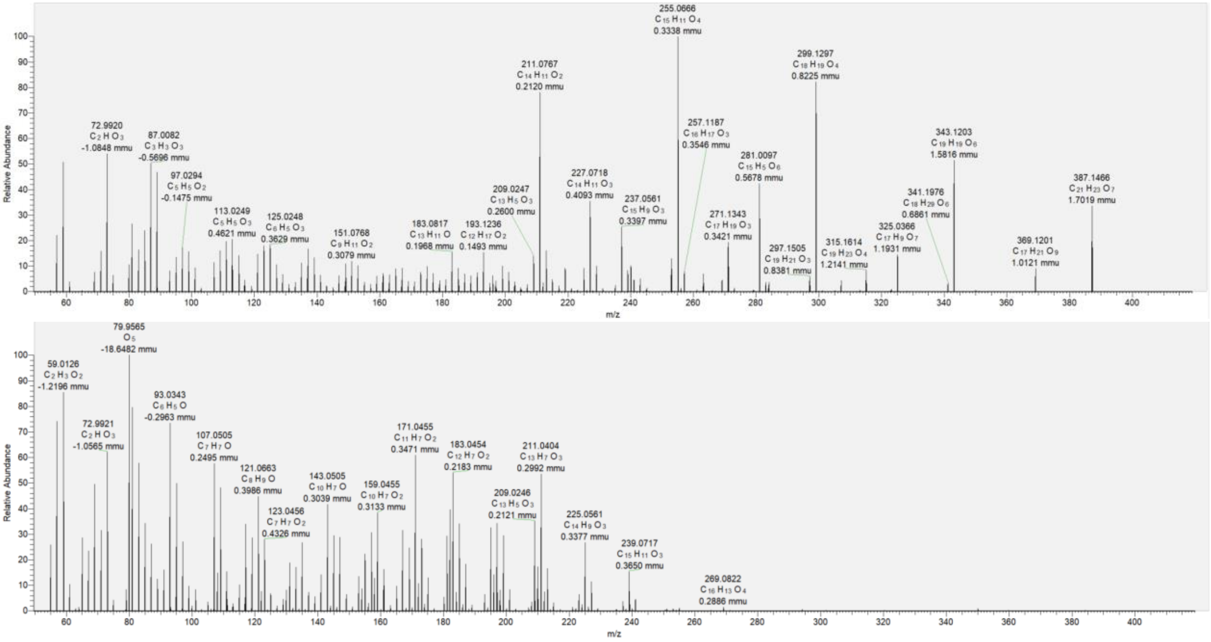

Figure S102: *Top: 35V HCD Fragmentation data from 3-9 minutes in LC for SRFA Nominal Mass 387. Bottom: 75V HCD Fragmentation data from 3-9 minutes in LC for SRFA Nominal Mass 387.*

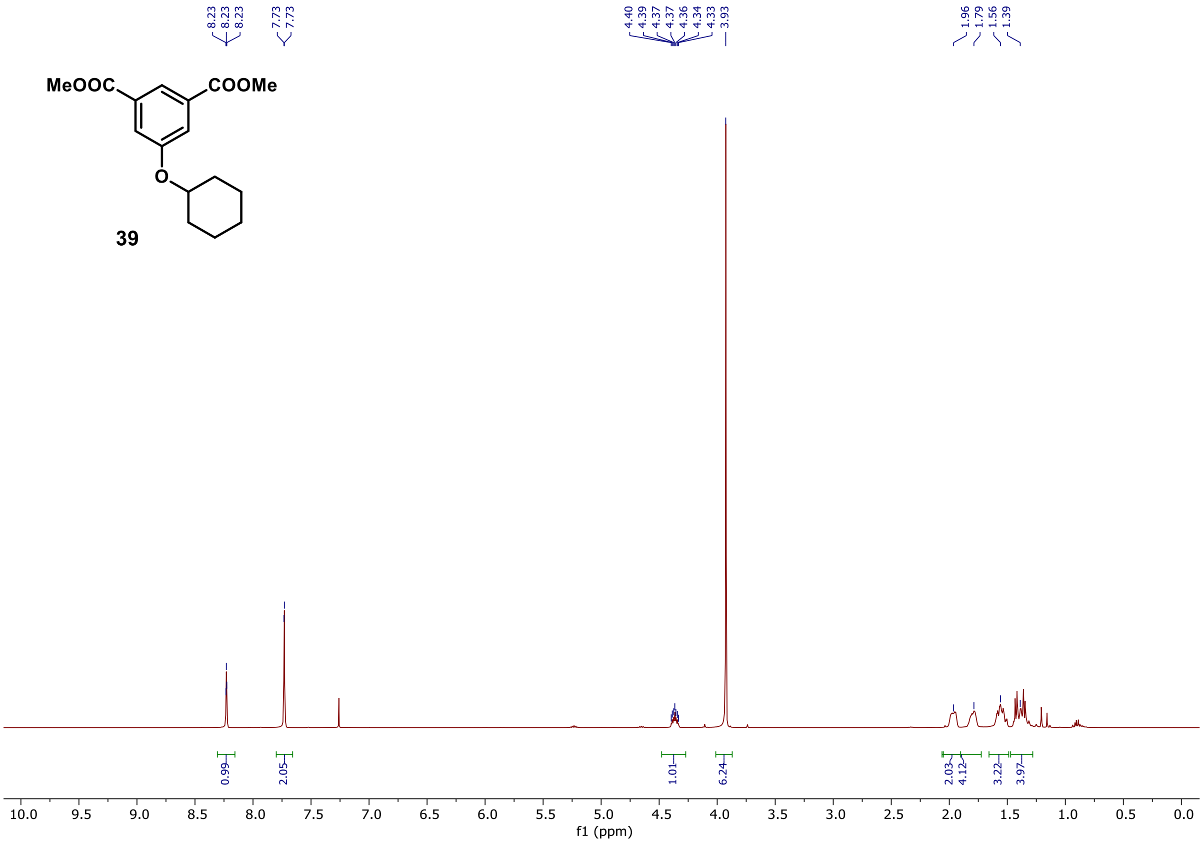

Figure S103: *^{1}H NMR spectra of diester **39** (400 MHz, $CDCl_3$).*

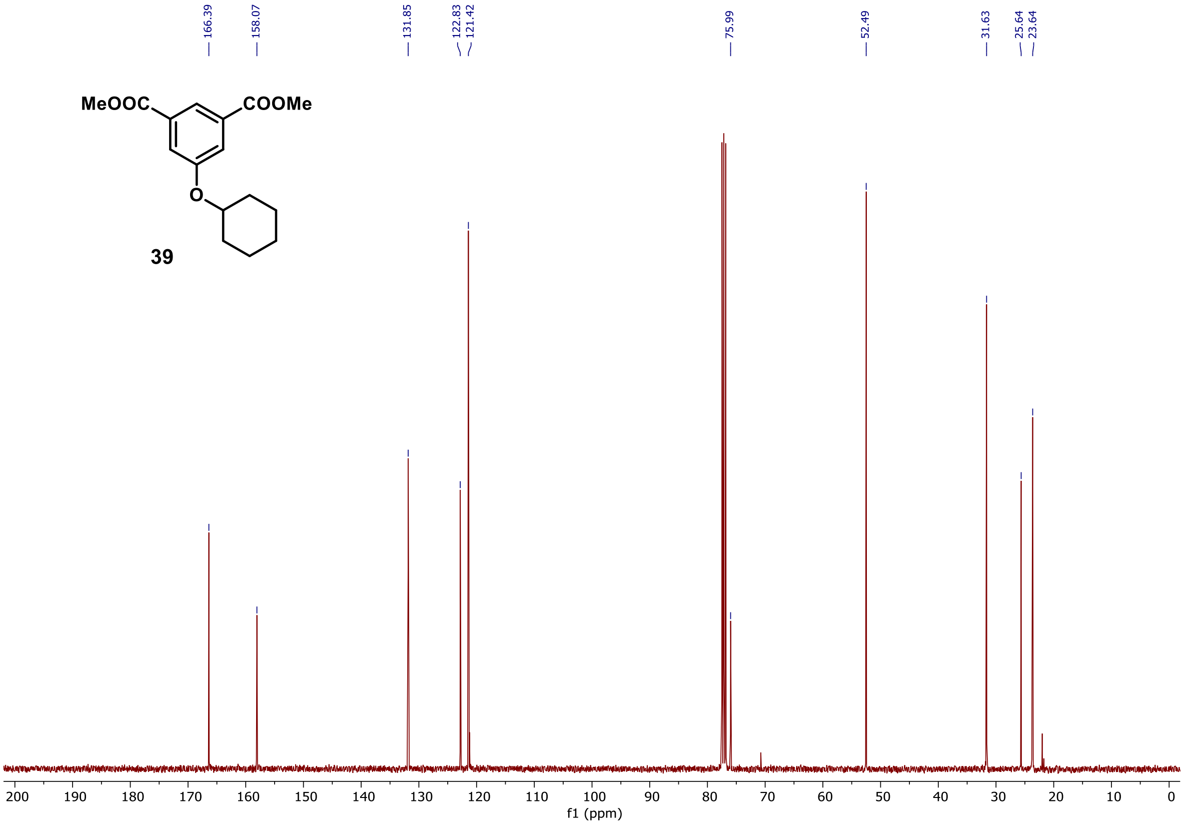

Figure S104: *^{13}C NMR spectrum of diester **39** ($CDCl_3$, 101 MHz).*

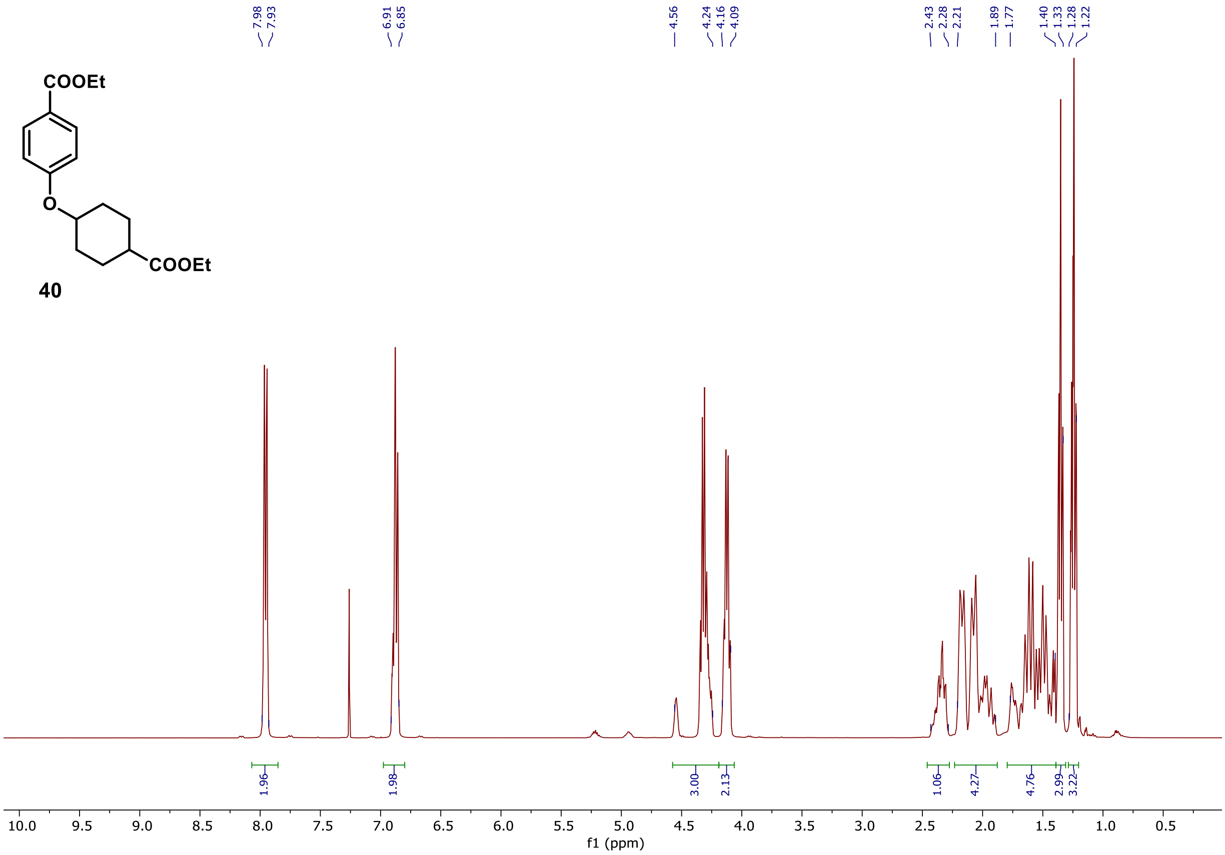

Figure S105: *^{1}H NMR spectra of diester* ***40*** *(400 MHz, $CDCl_3$).*

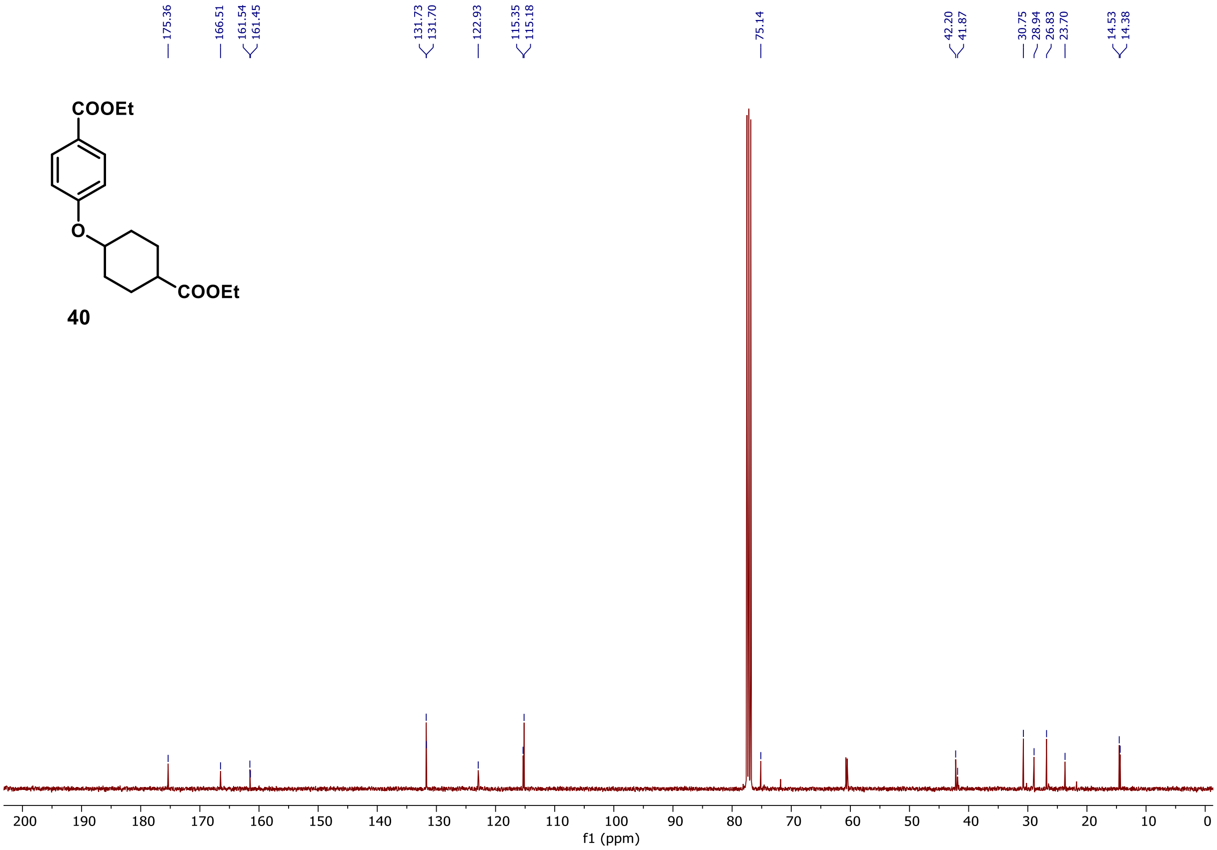

Figure S106: *^{13}C NMR spectrum of diester* ***40*** *($CDCl_3$, 101 MHz).*

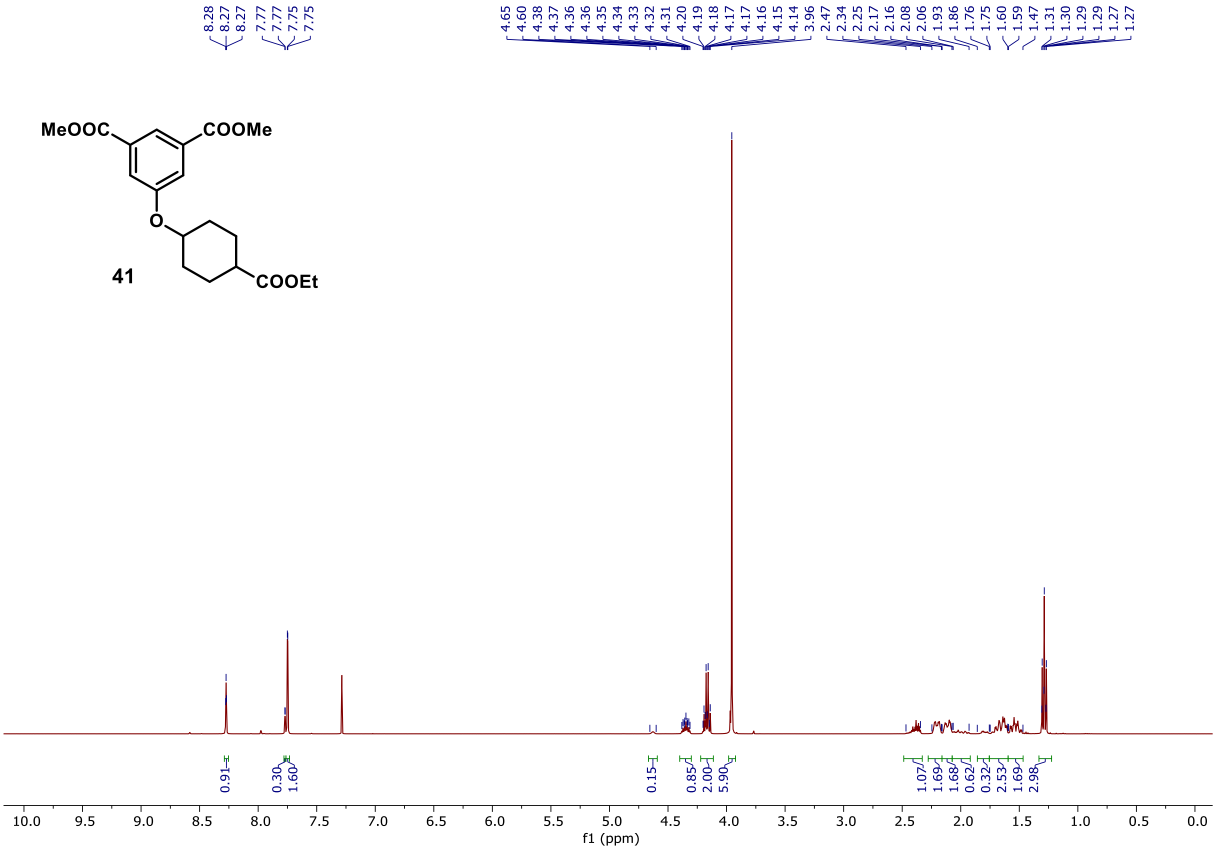

Figure S107: *^{1}H NMR spectra of triester* ***41*** *(400 MHz, $CDCl_3$).*

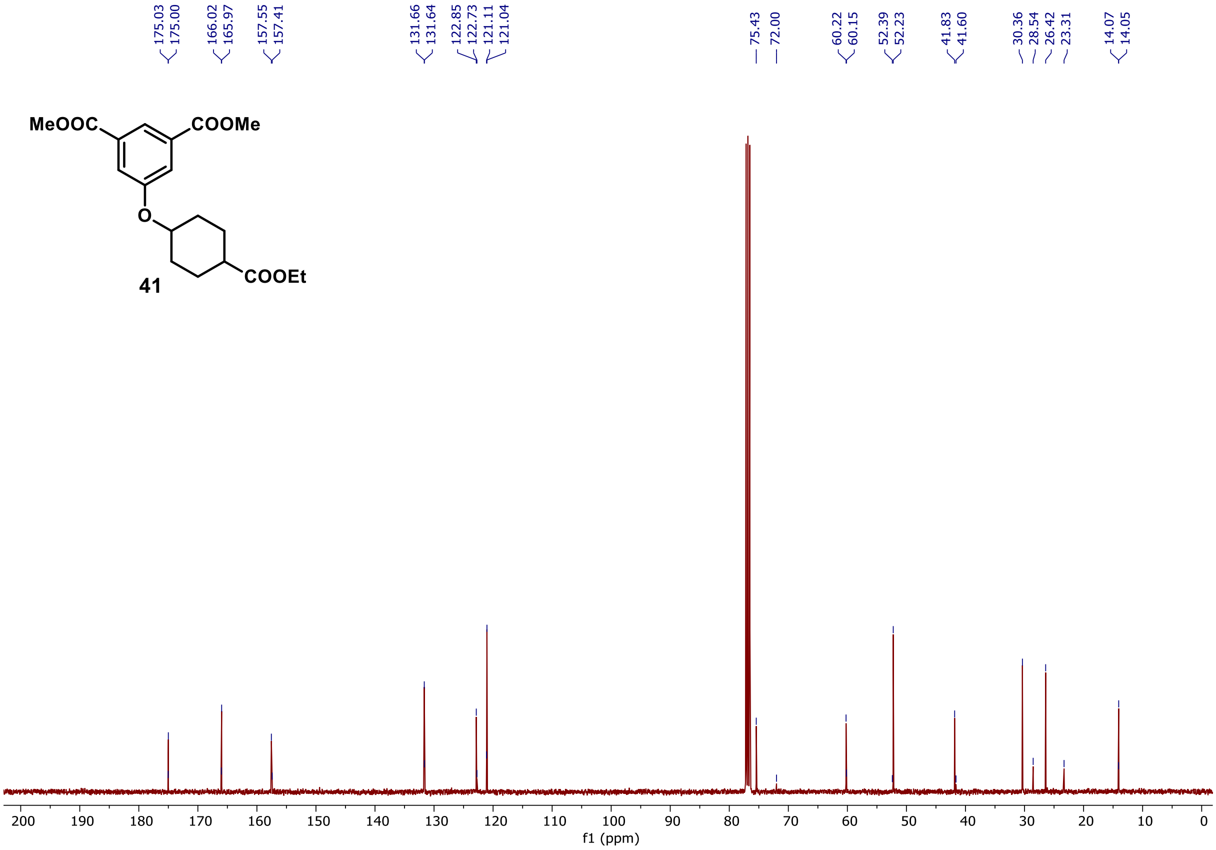

Figure S108: *^{13}C NMR spectrum of triester* ***41*** *($CDCl_3$, 101 MHz).*

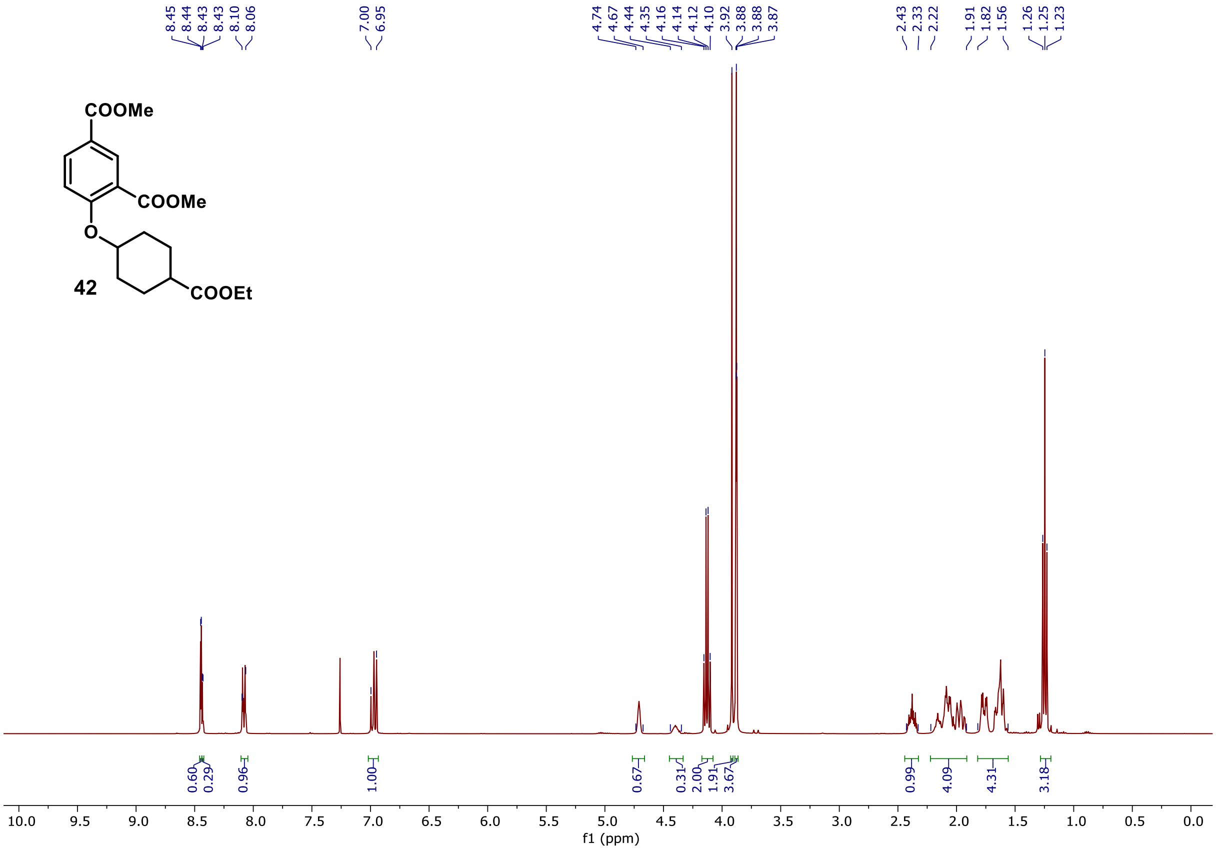

Figure S109: *^{1}H NMR spectra of triester **42** (400 MHz, $CDCl_3$).*

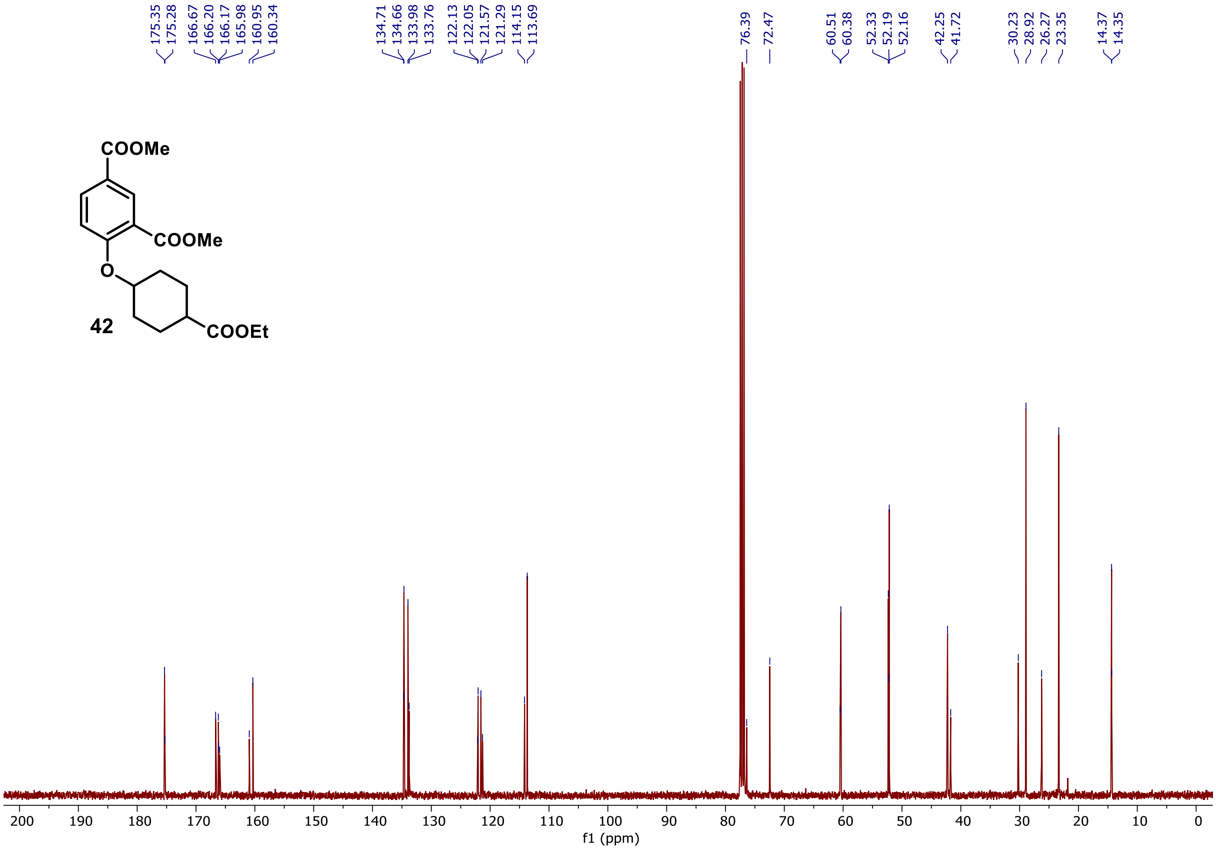

Figure S110: *^{1}H NMR spectra of triester **42** (400 MHz, $CDCl_3$).*

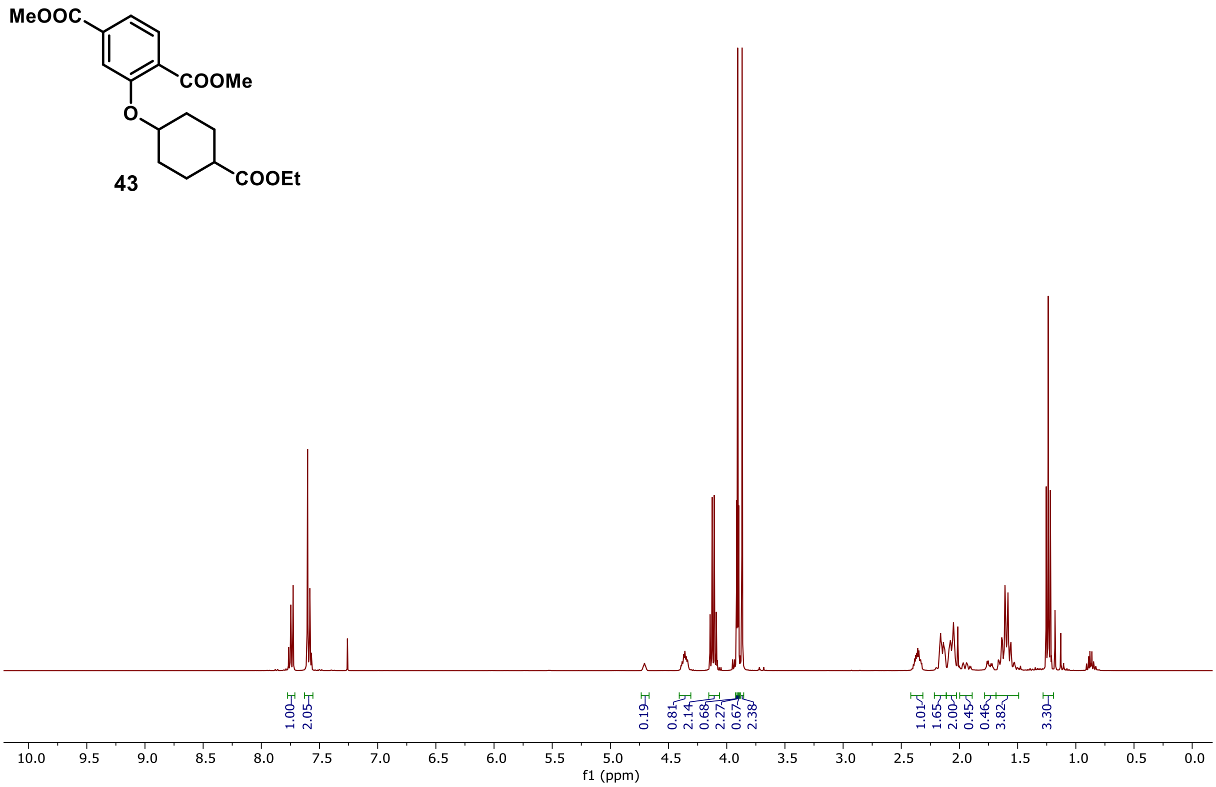

Figure S111: *^{1}H NMR spectra of triester **43** (400 MHz, $CDCl_3$).*

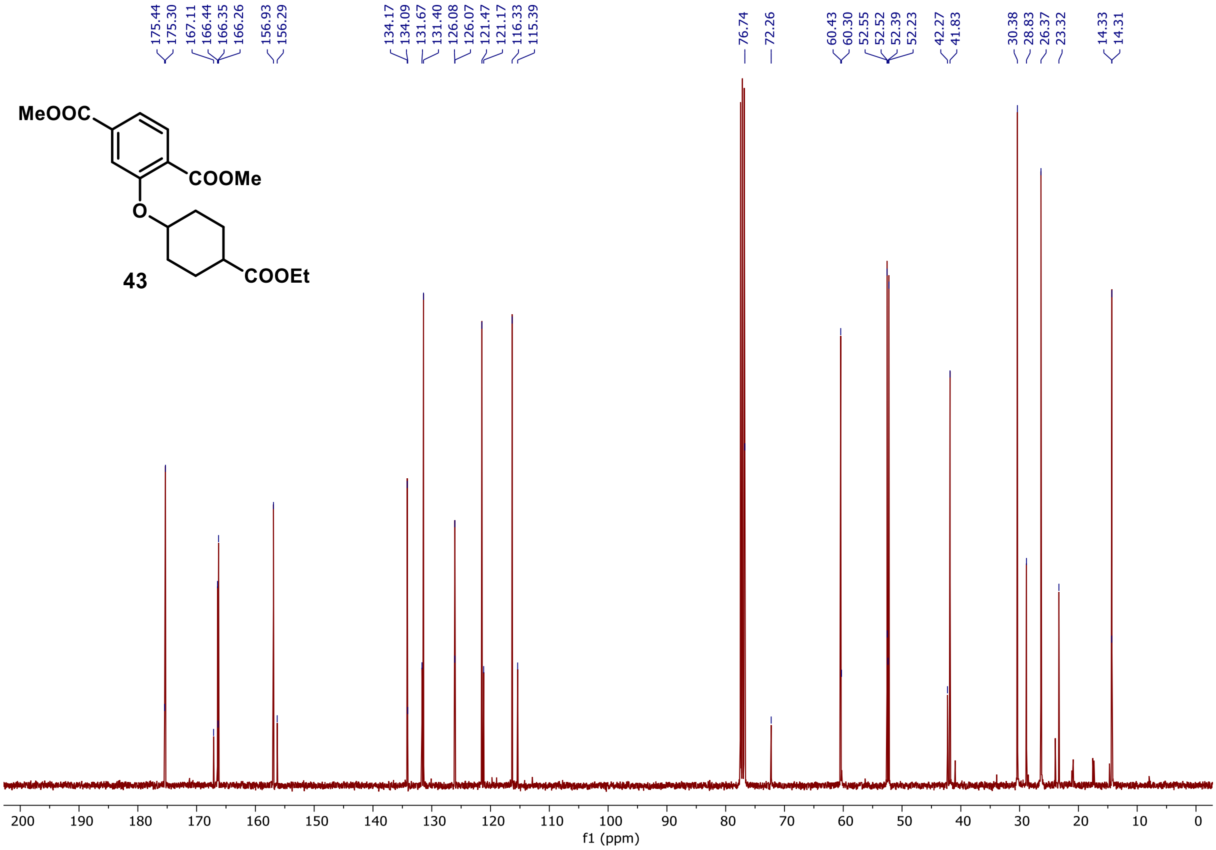

Figure S112: *^{13}C NMR spectrum of triester **43** ($CDCl_3$, 101 MHz)***.***

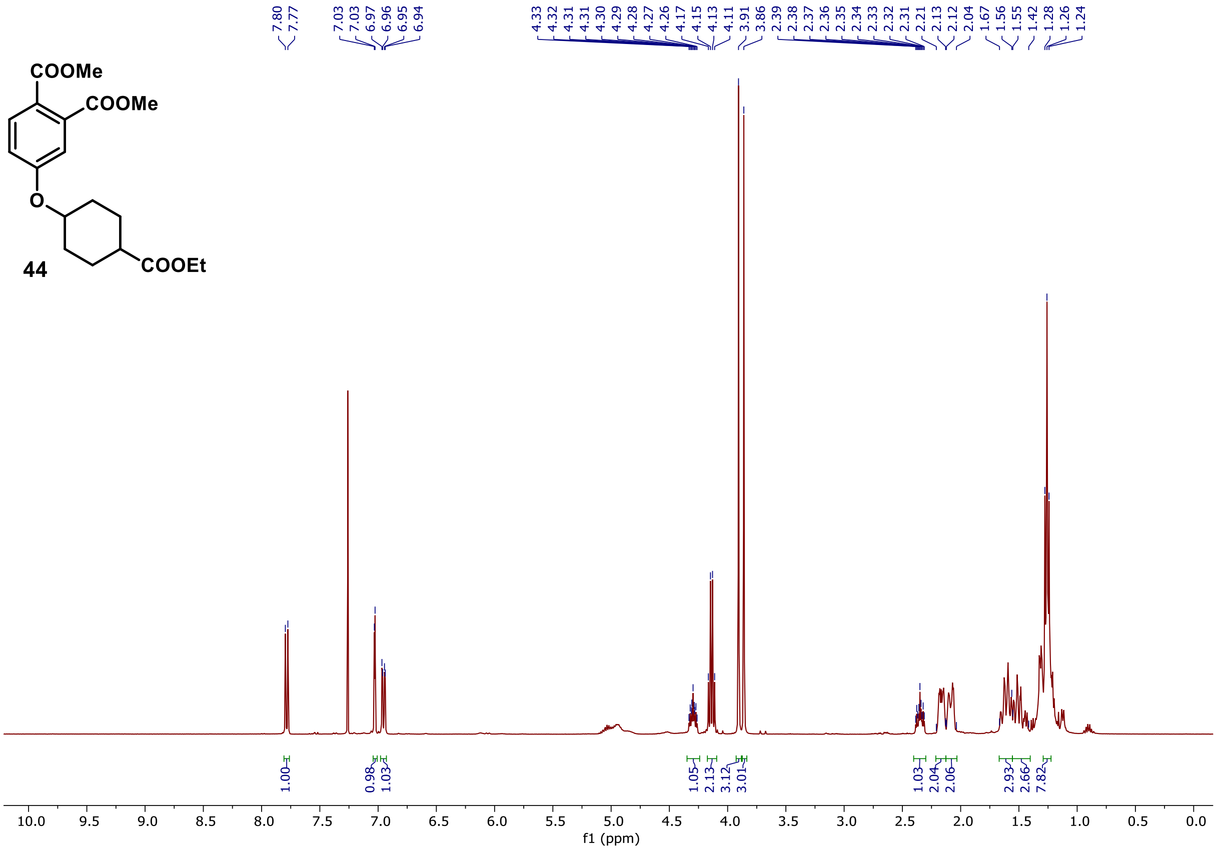

Figure S113: *^{1}H NMR spectra of triester* ***44*** *(400 MHz, $CDCl_3$).*

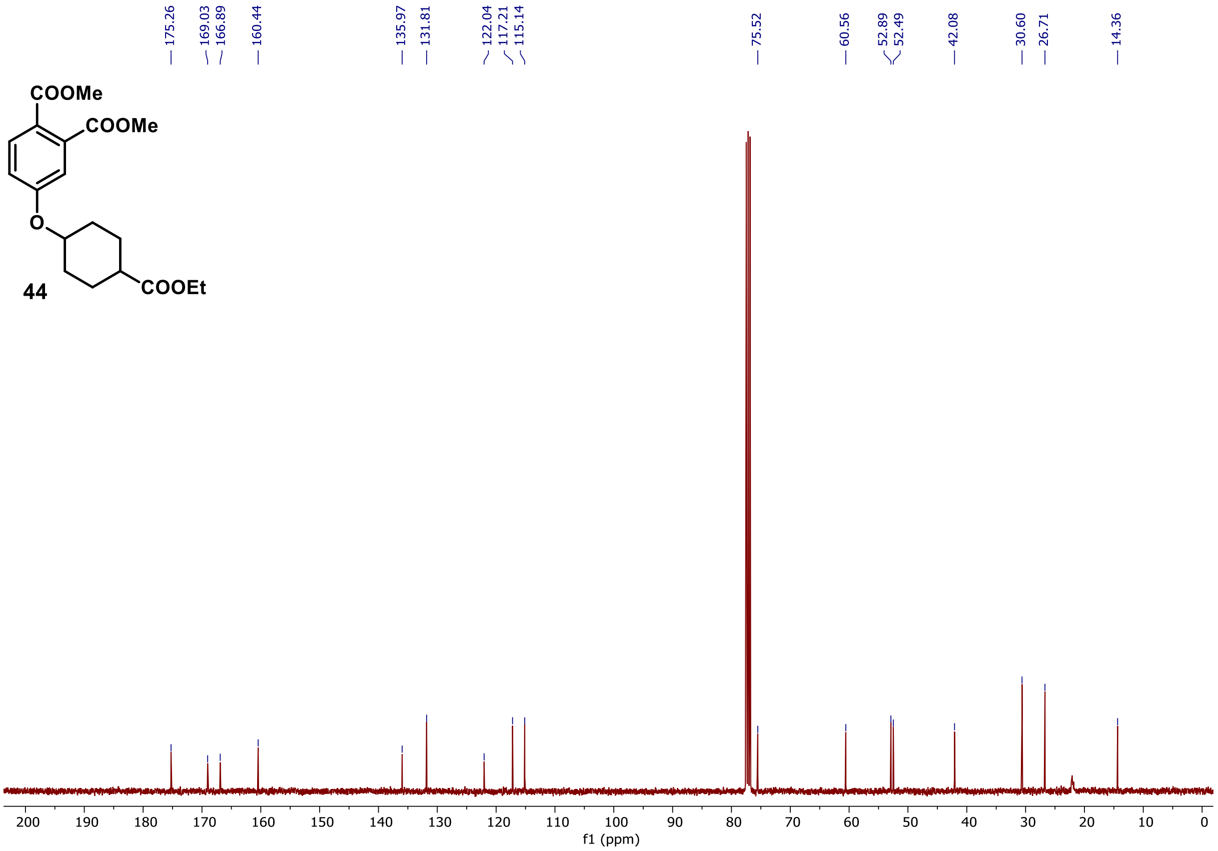

Figure S114: *^{13}C NMR spectrum of triester* ***44*** *($CDCl_3$, 101 MHz).*

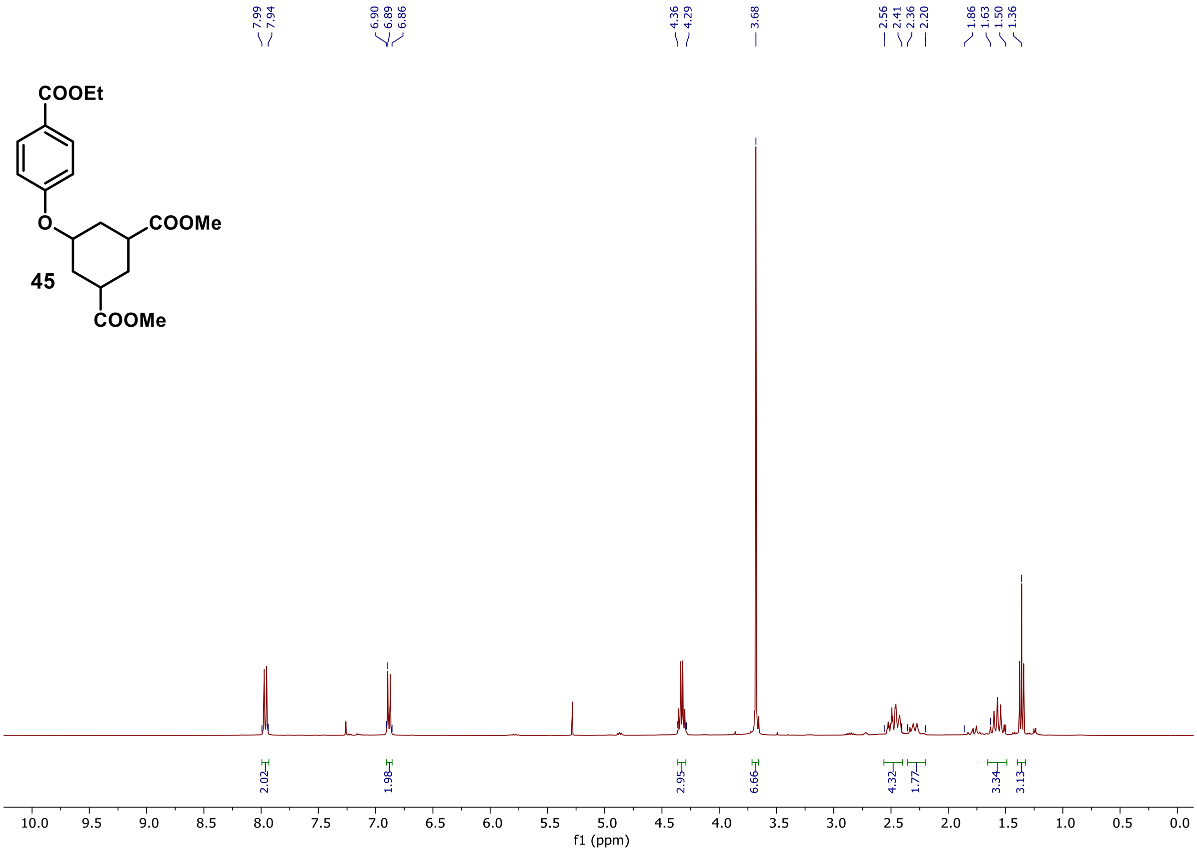

Figure S115: *^{1}H NMR spectra of triester* ***45*** *(400 MHz, CDCl₃).*

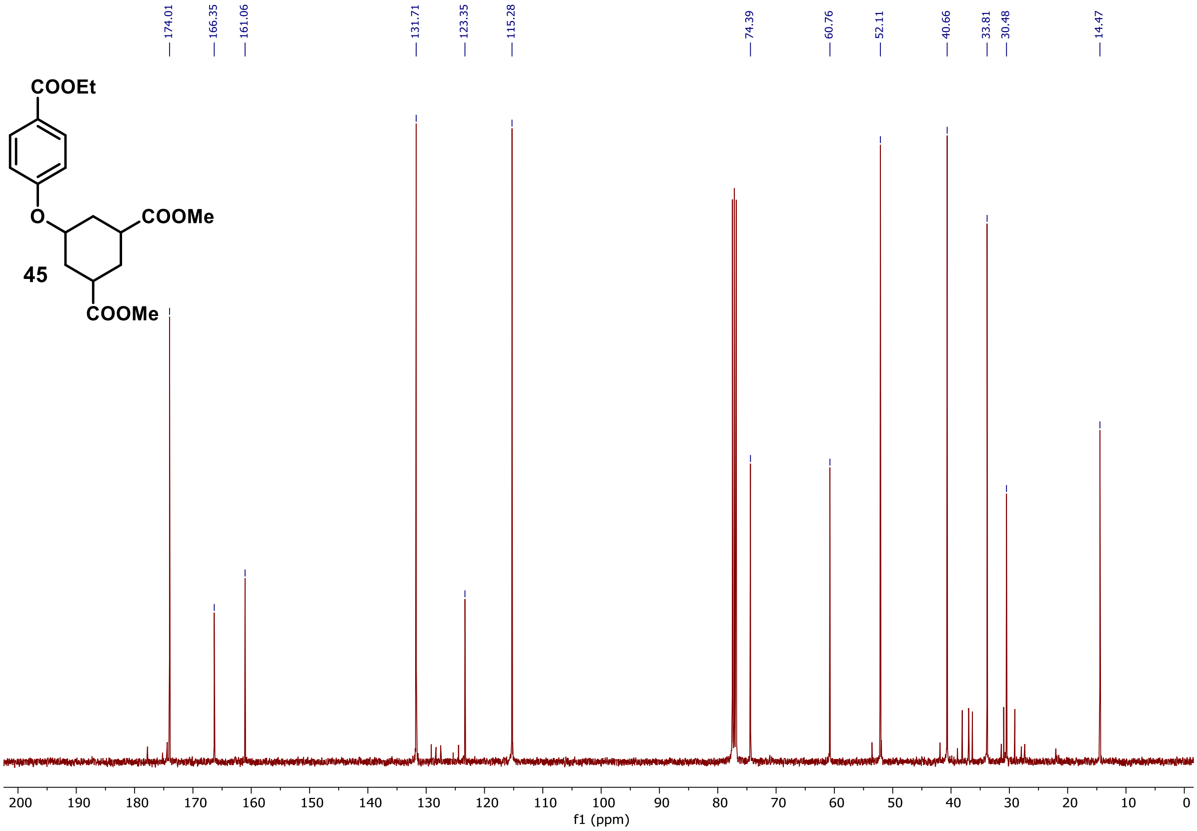

Figure S116: *^{13}C NMR spectrum of triesteracid* ***45*** ***(**CDCl₃, 101 MHz**)**.*

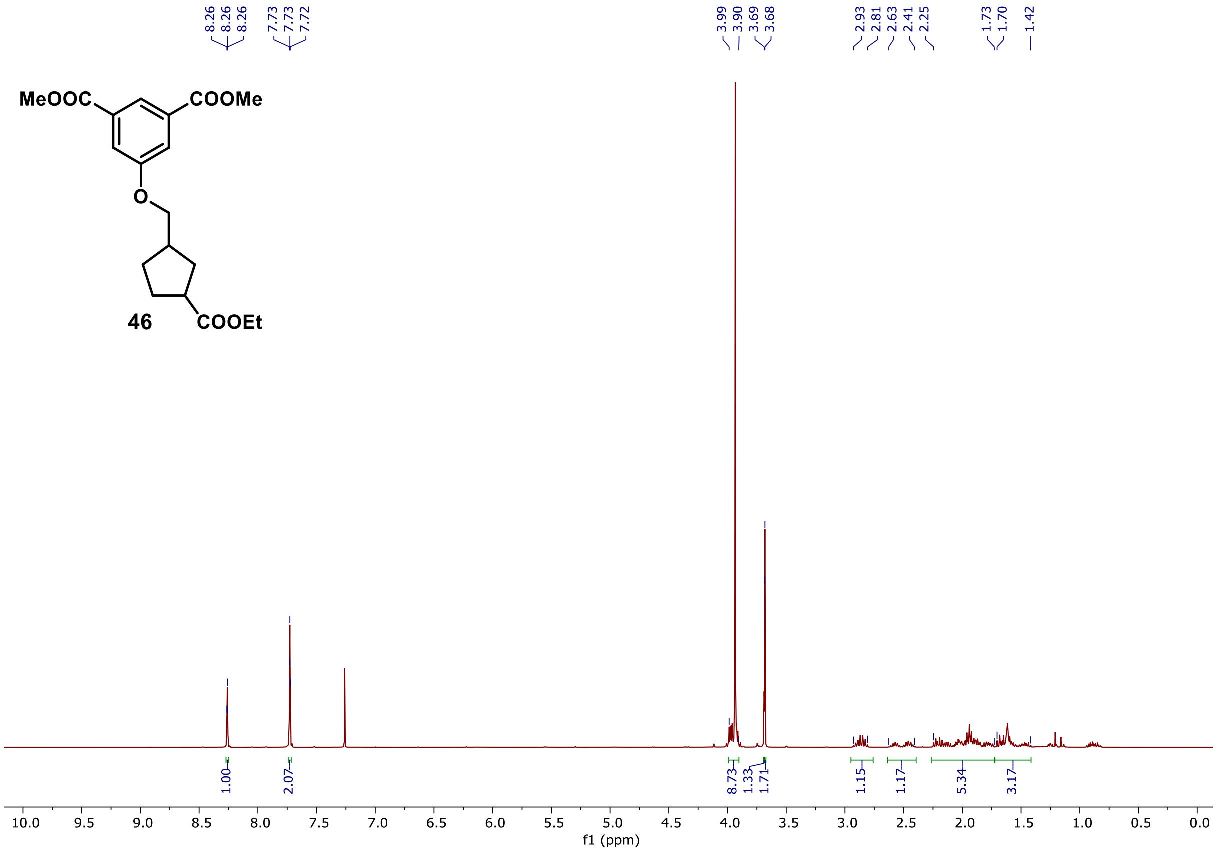

Figure S117: [1]*H NMR spectra of triester **46** (400 MHz, CDCl$_3$).*

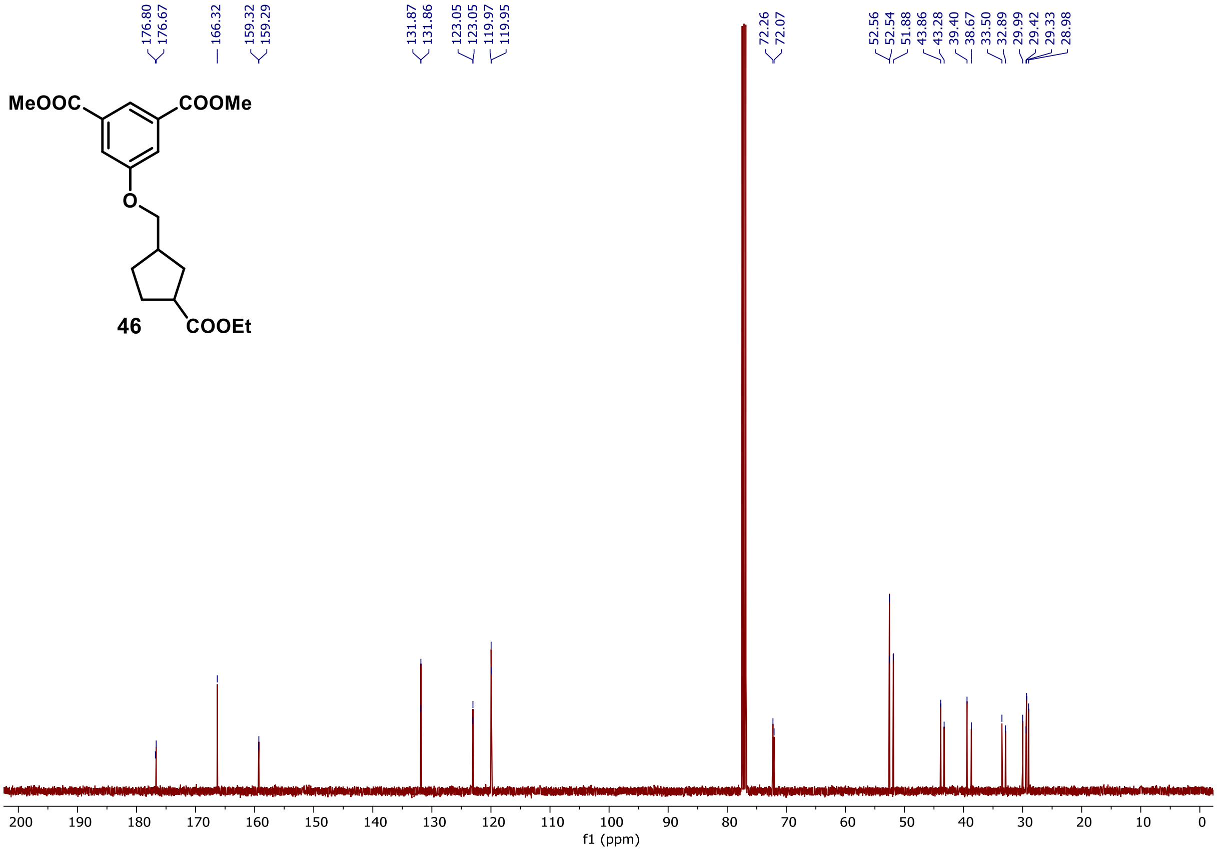

Figure S118: [13]*C NMR spectrum of triester **46** (CDCl$_3$, 101 MHz).*

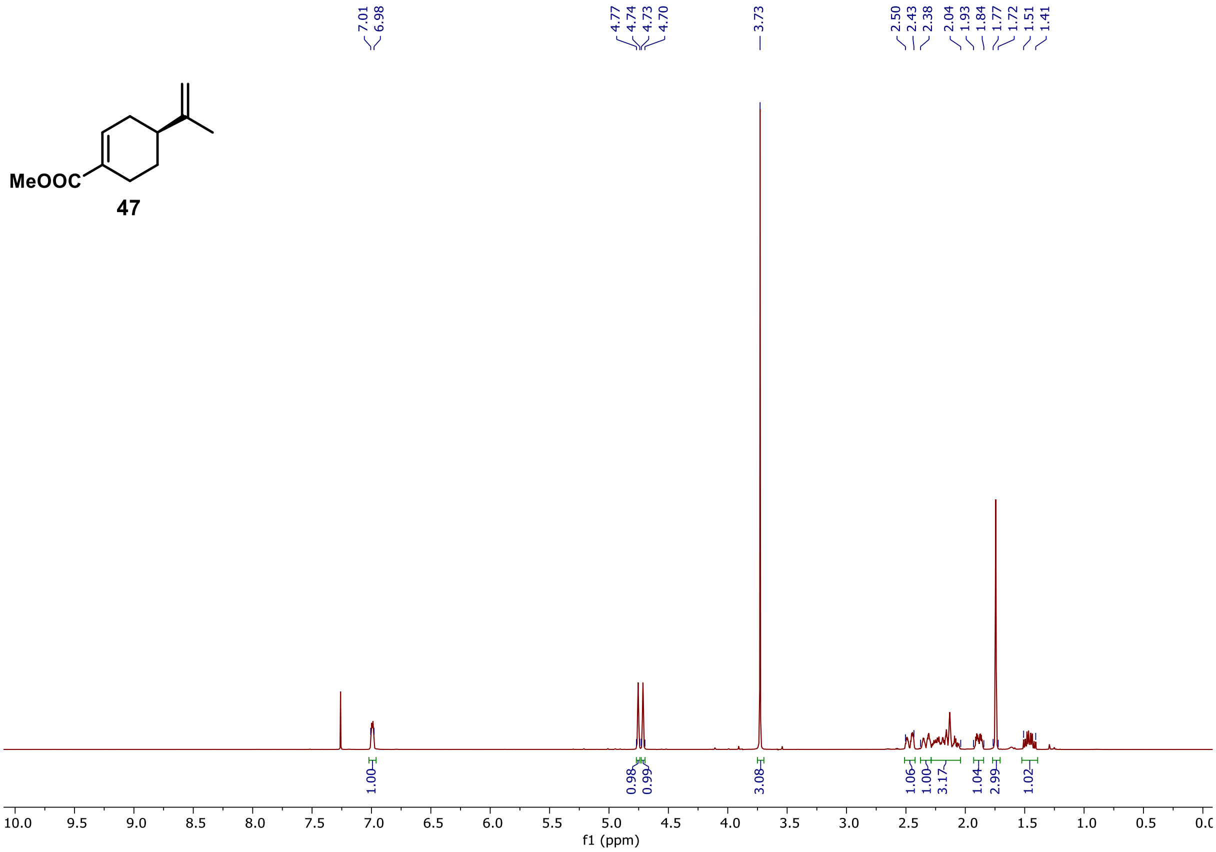

Figure S119: *^{1}H NMR spectra of (-)-perillic acid methyl ester* ***47*** *(400 MHz, $CDCl_3$).*

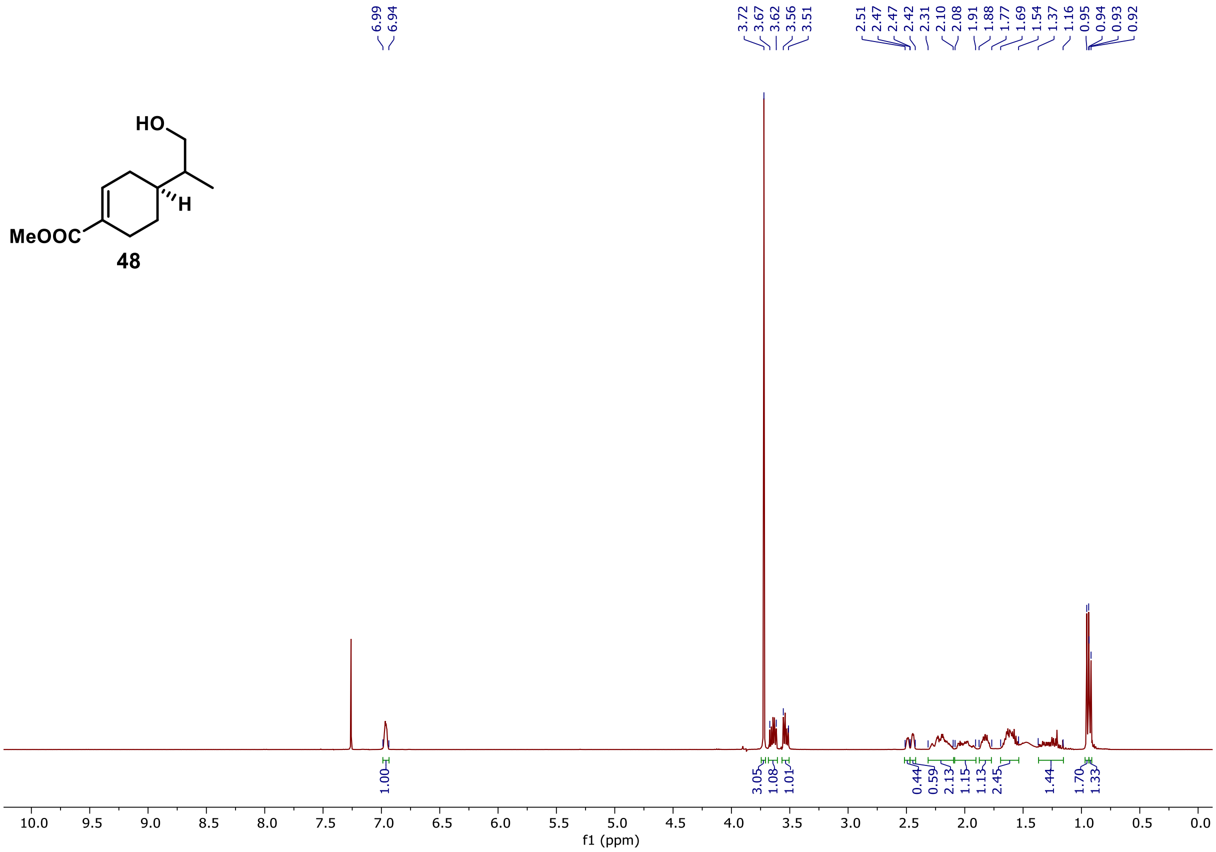

Figure S120: *^{1}H NMR spectra of alcohol* ***48*** *(400 MHz, $CDCl_3$).*

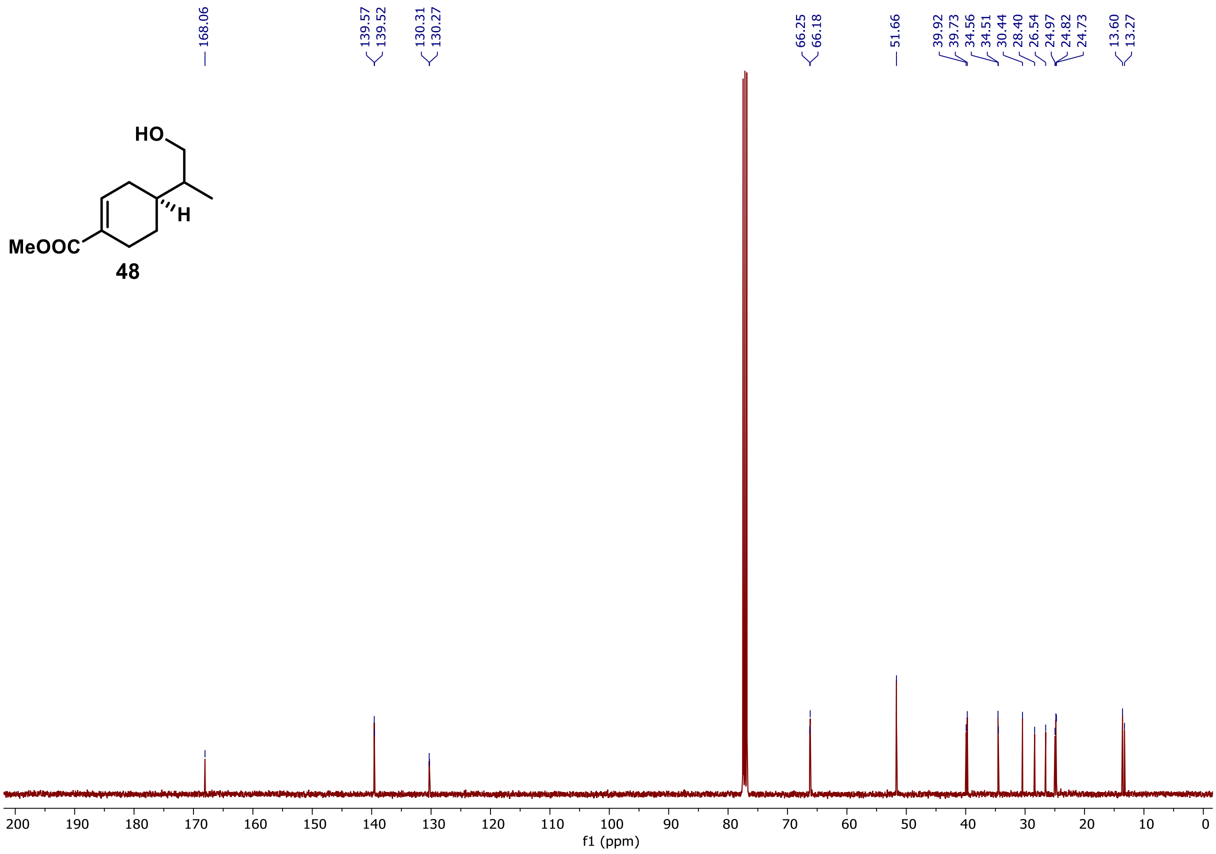

Figure S121: *^{13}C NMR spectrum of alcohol* ***48*** *($CDCl_3$, 101 MHz)****.***

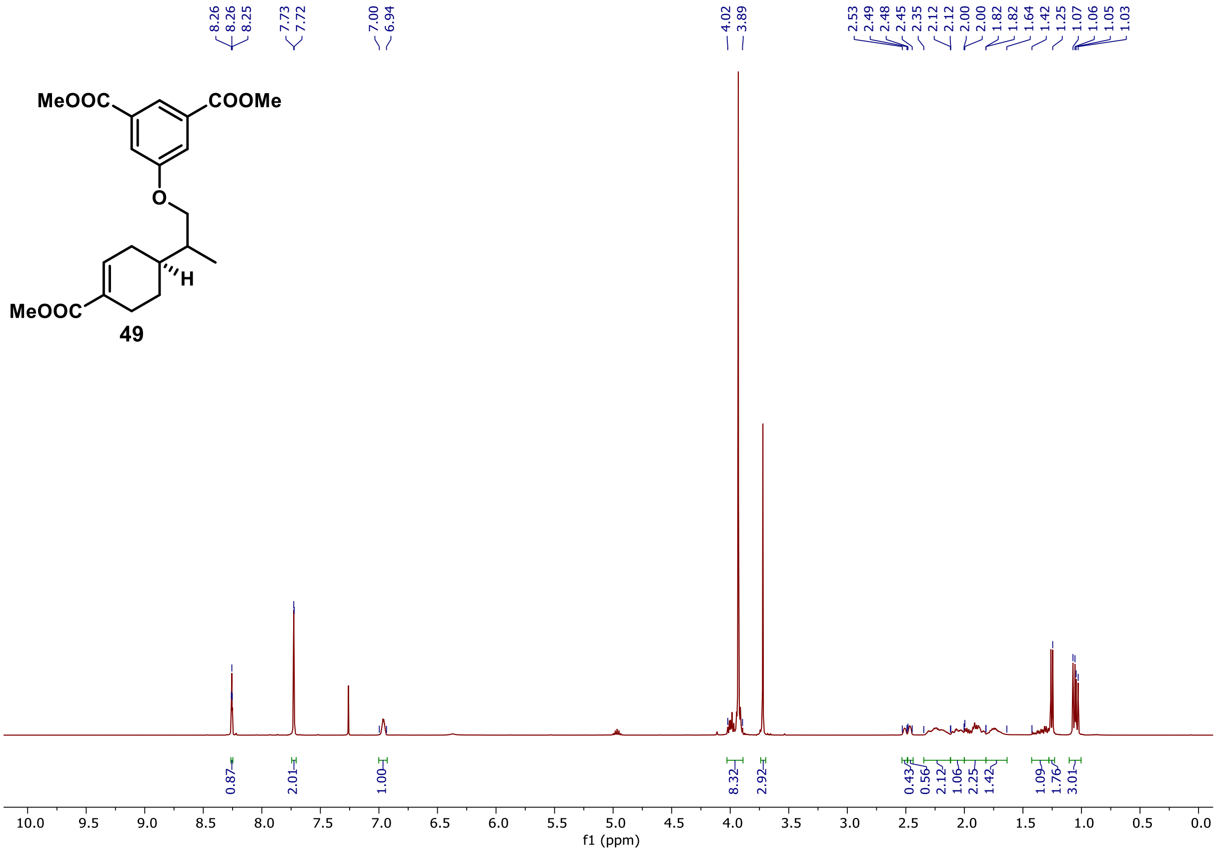

Figure S122: *^{1}H NMR spectra of triester* ***49*** *(400 MHz, $CDCl_3$).*

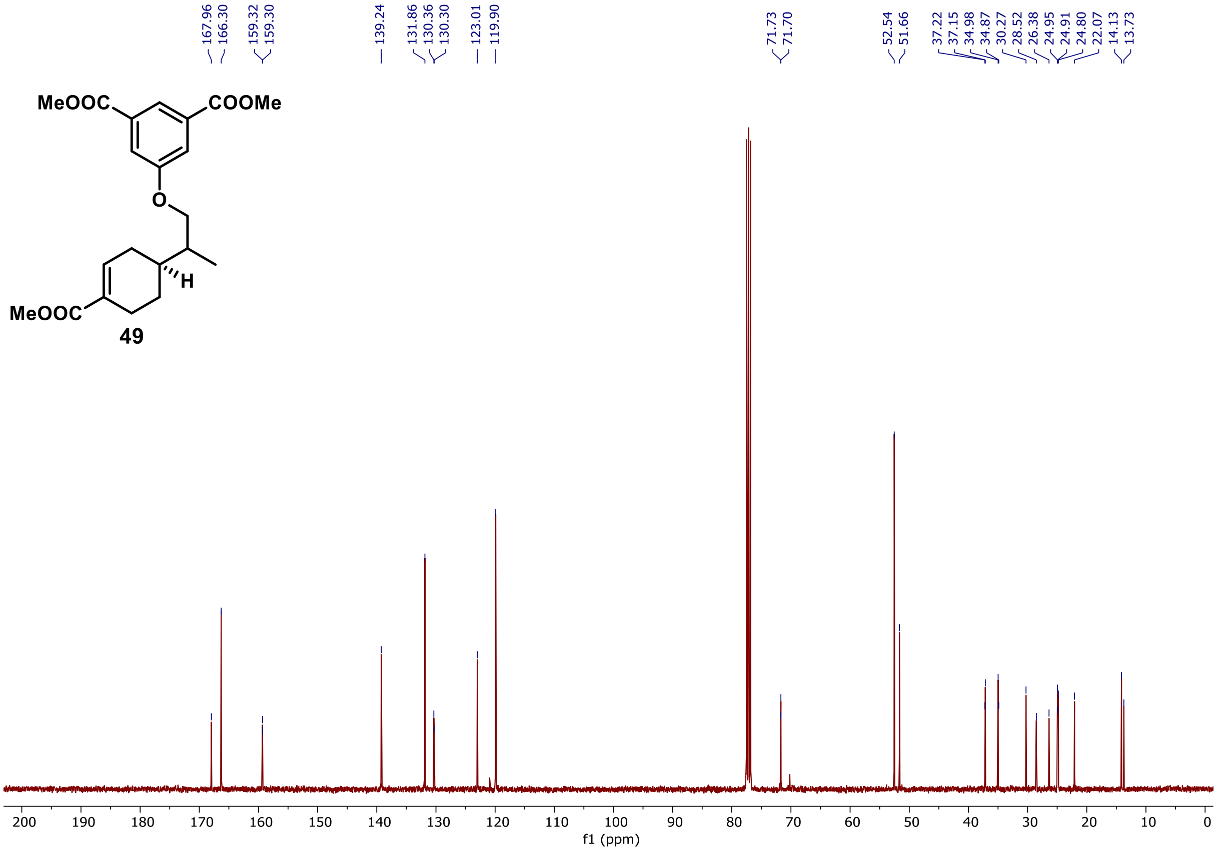

Figure S123: *^{13}C NMR spectrum of triester* ***49*** *($CDCl_3$, 101 MHz)****.***

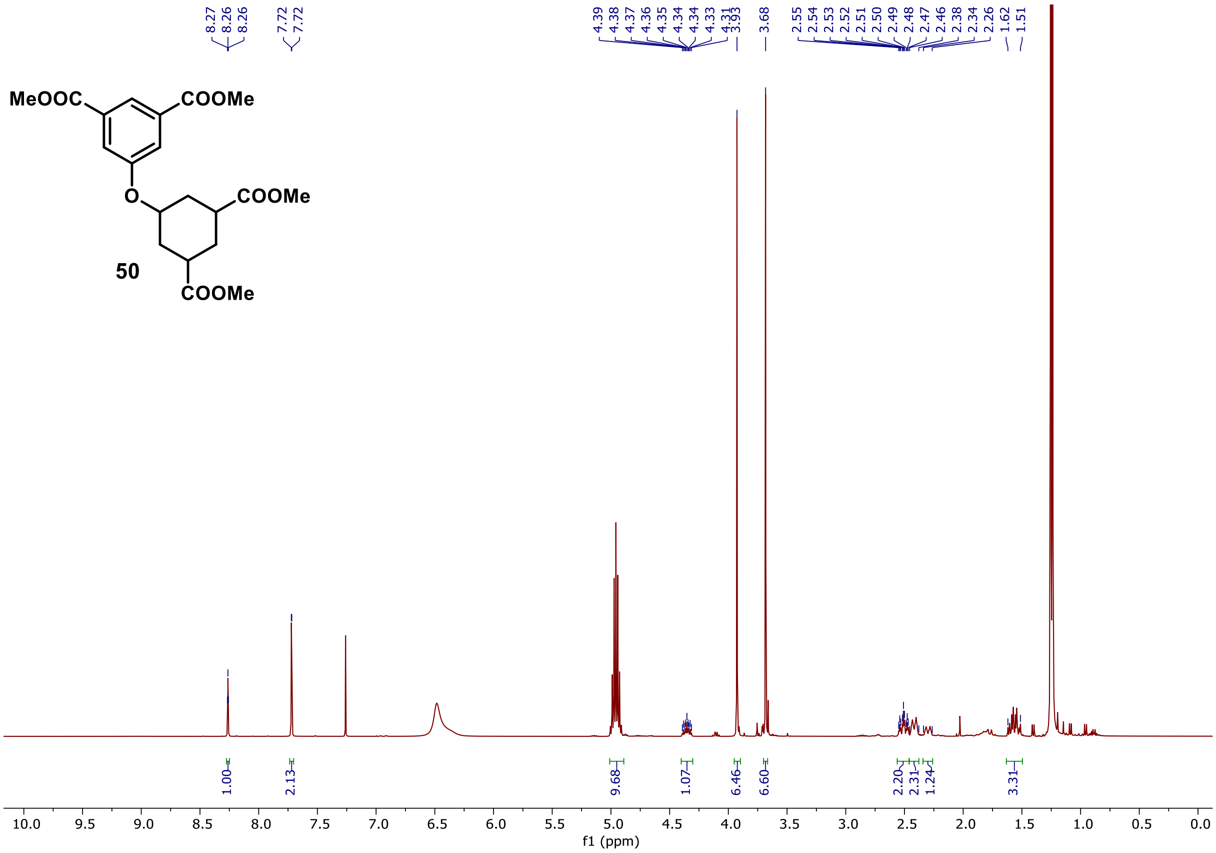

Figure S124: *^{1}H NMR spectra of tetraester **50** (400 MHz, CDCl₃).*

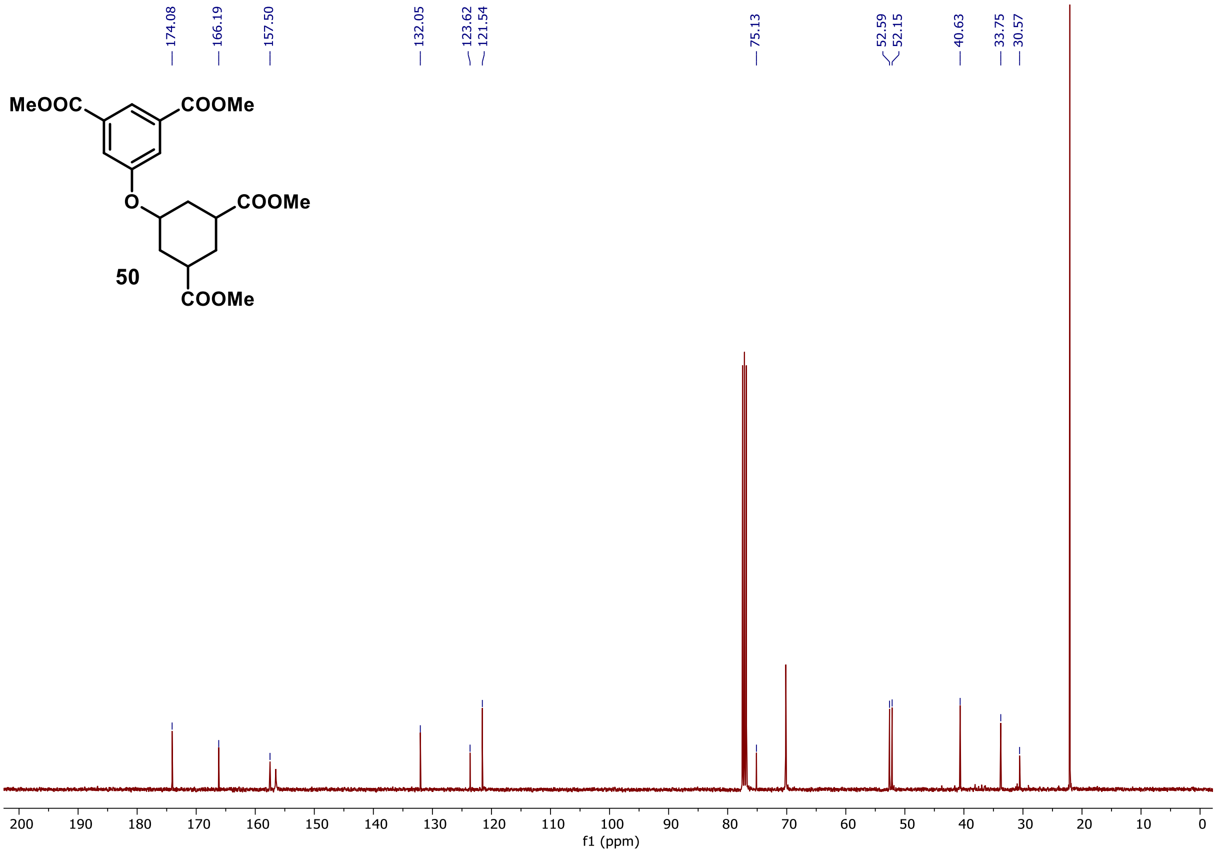

Figure S125: *^{13}C NMR spectrum of tetraester **50** (CDCl₃, 101 MHz).*

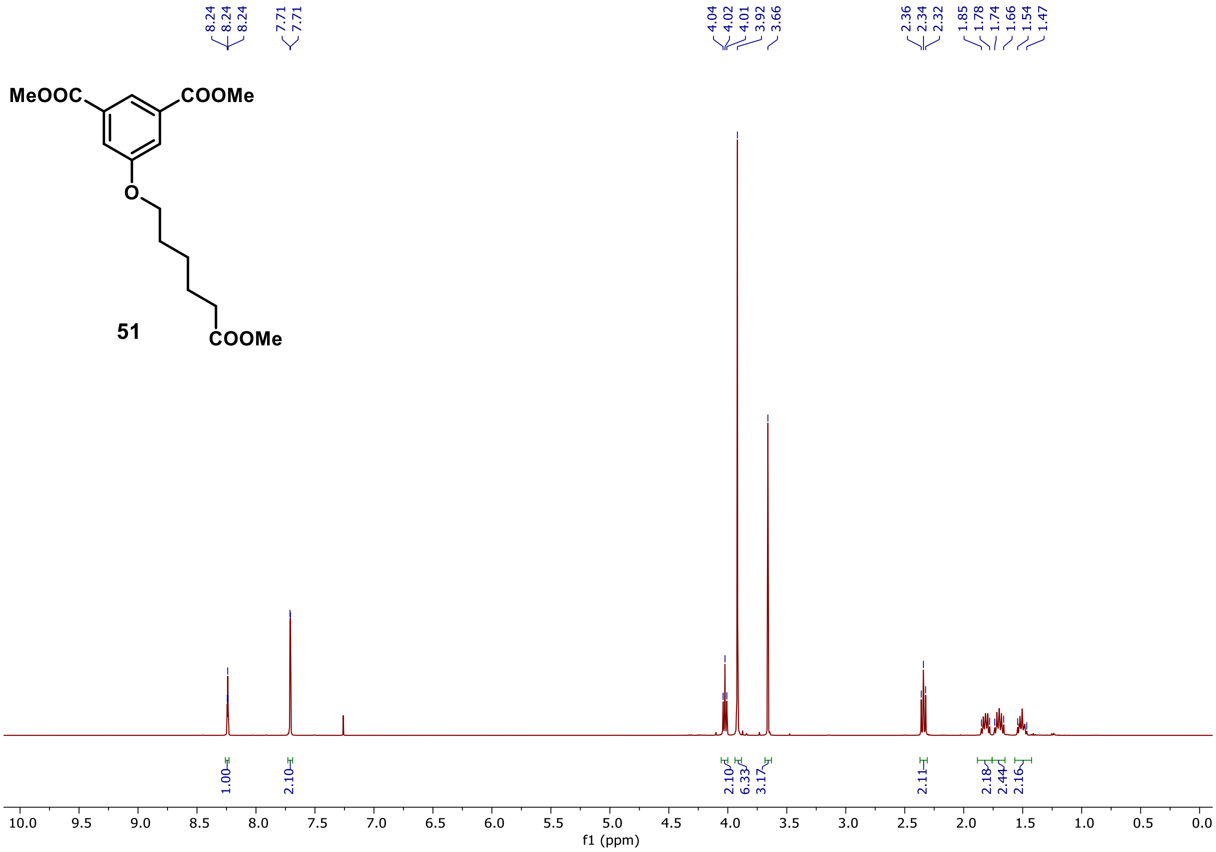

Figure S126: *^{1}H NMR spectra of triester* ***51*** *(400 MHz, $CDCl_3$).*

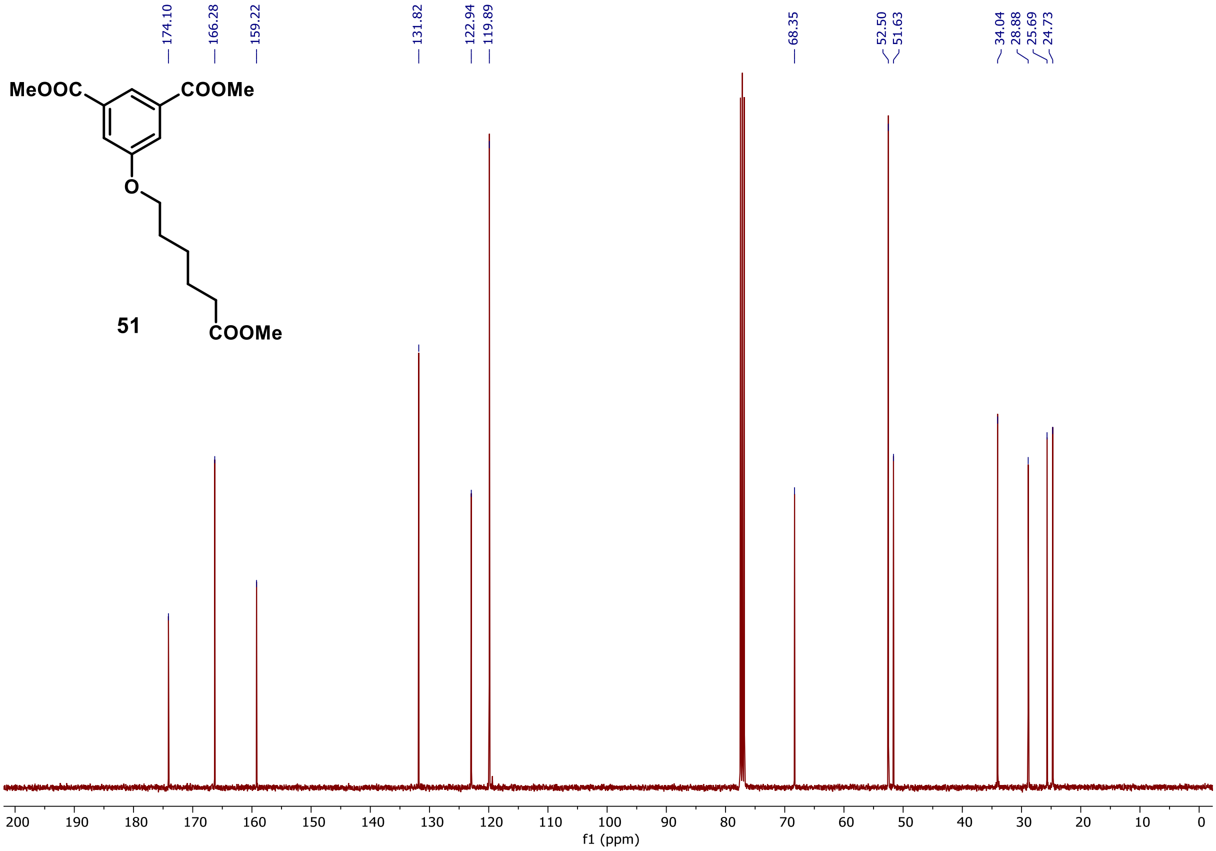

Figure S127: *^{13}C NMR spectrum of triester* ***51*** *($CDCl_3$, 101 MHz)****.***

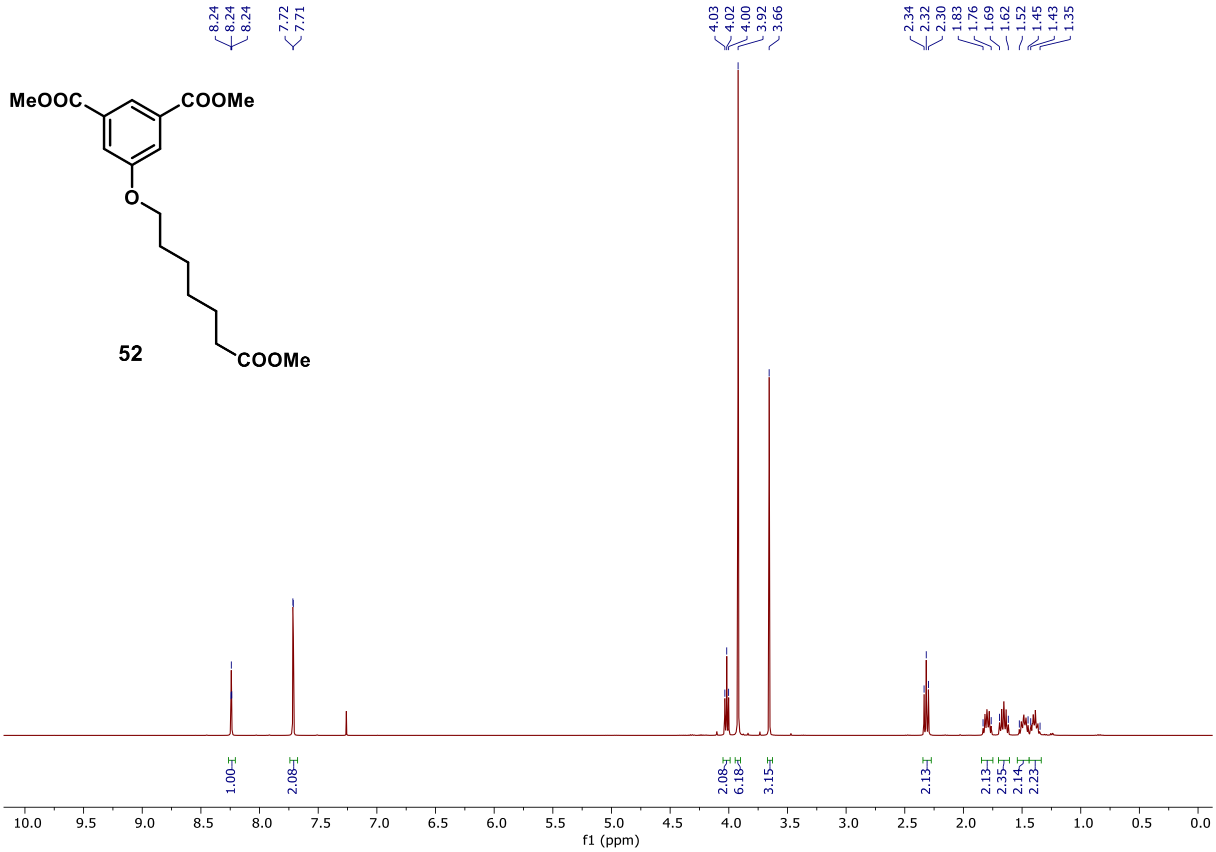

Figure S128: *¹H NMR spectra of triester* ***52*** *(400 MHz, $CDCl_3$).*

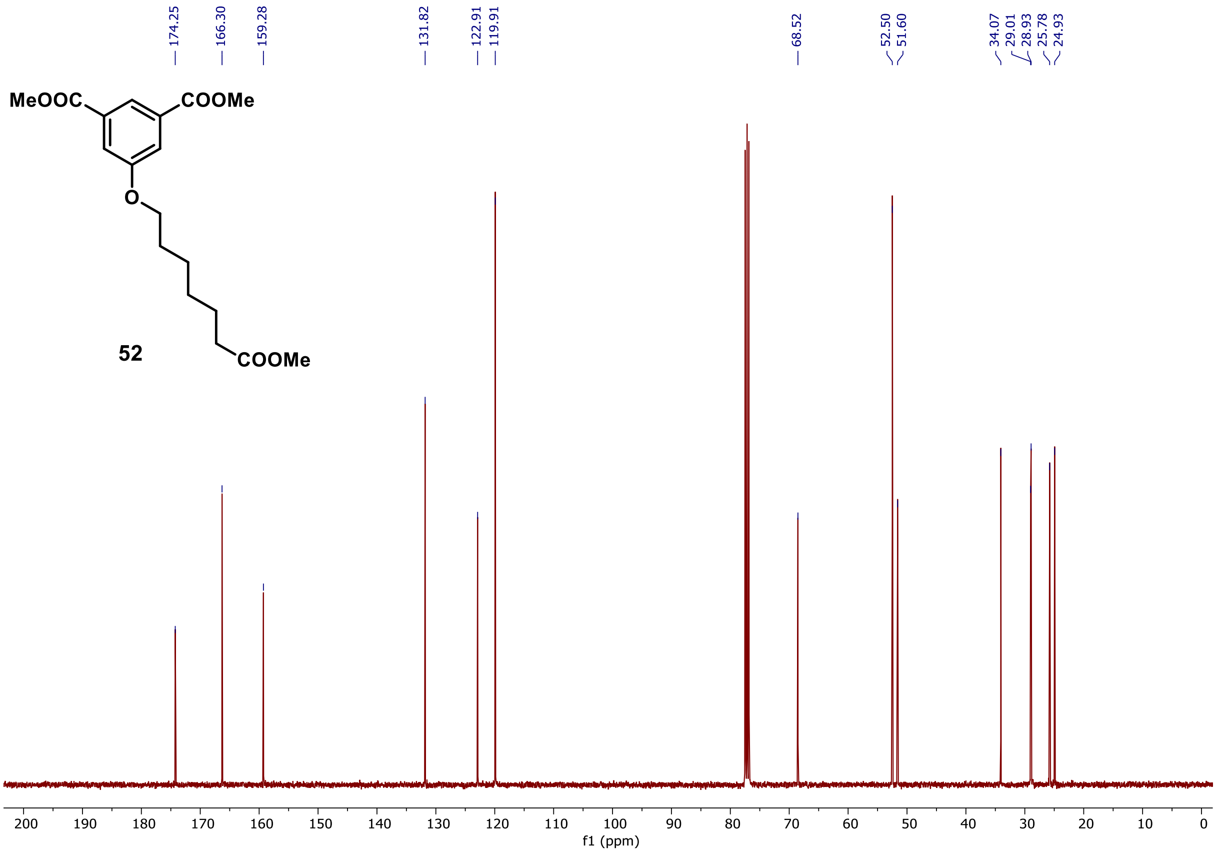

Figure S129: *¹³C NMR spectrum of triester* ***52*** *($CDCl_3$, 101 MHz)****.***

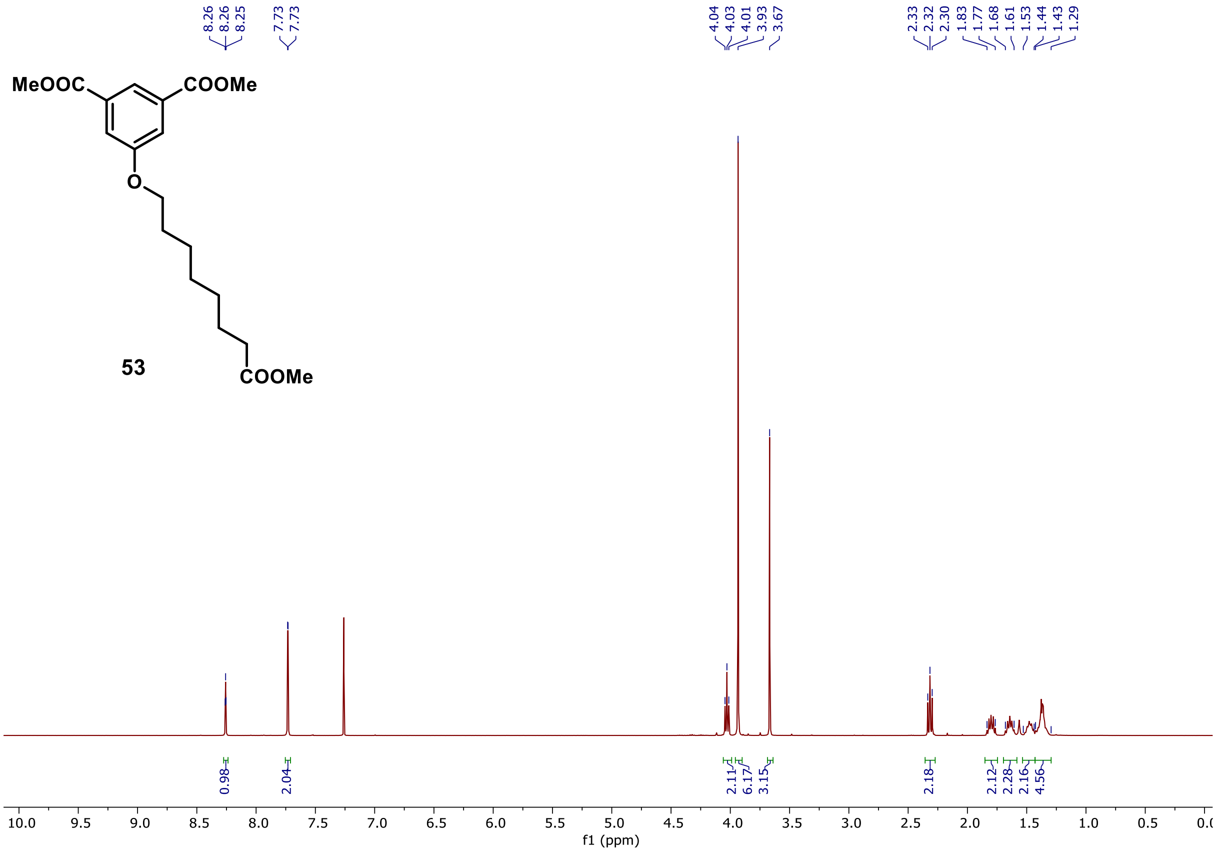

Figure S130: ^{1}H NMR spectra of triester **53** (400 MHz, $CDCl_3$).

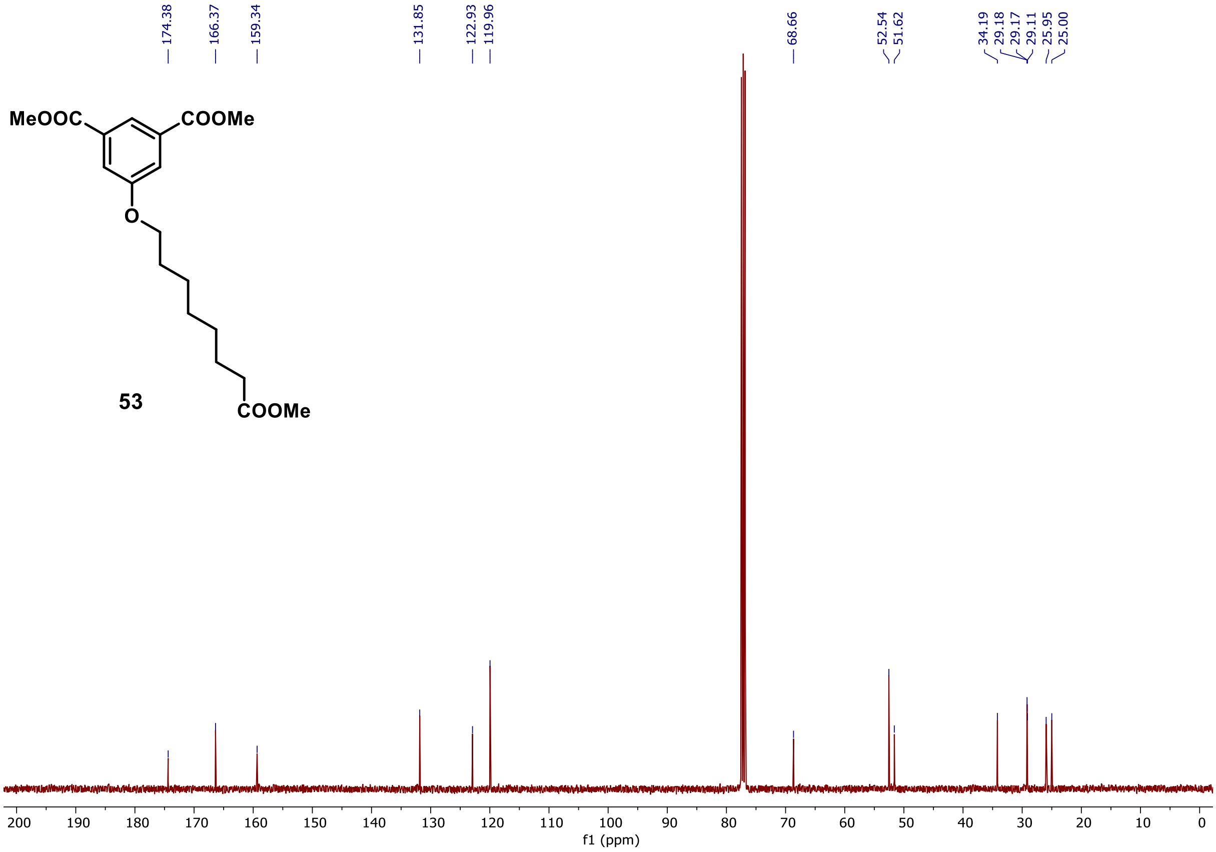

Figure S131: ^{13}C NMR spectrum of triester **53** ($CDCl_3$, 101 MHz).

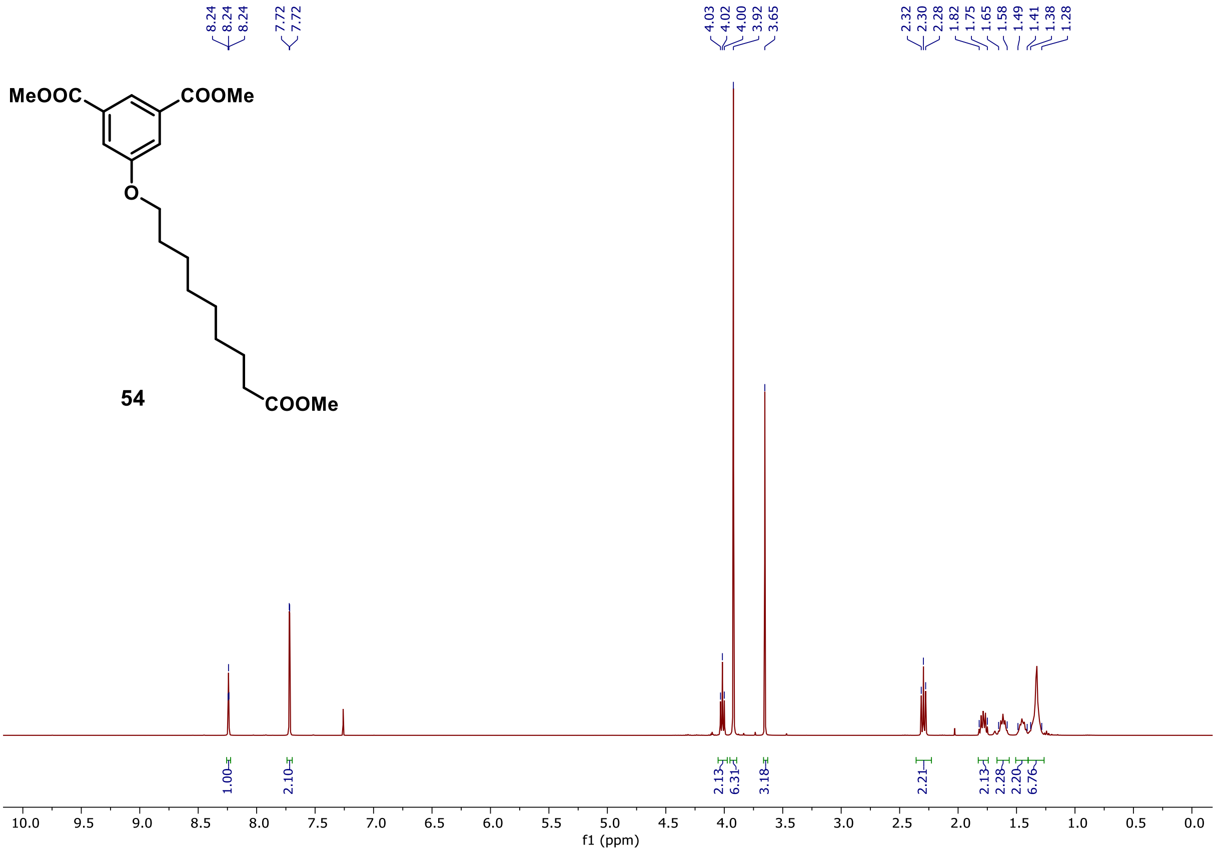

Figure S132: *^{1}H NMR spectra of triester **54** (400 MHz, $CDCl_3$).*

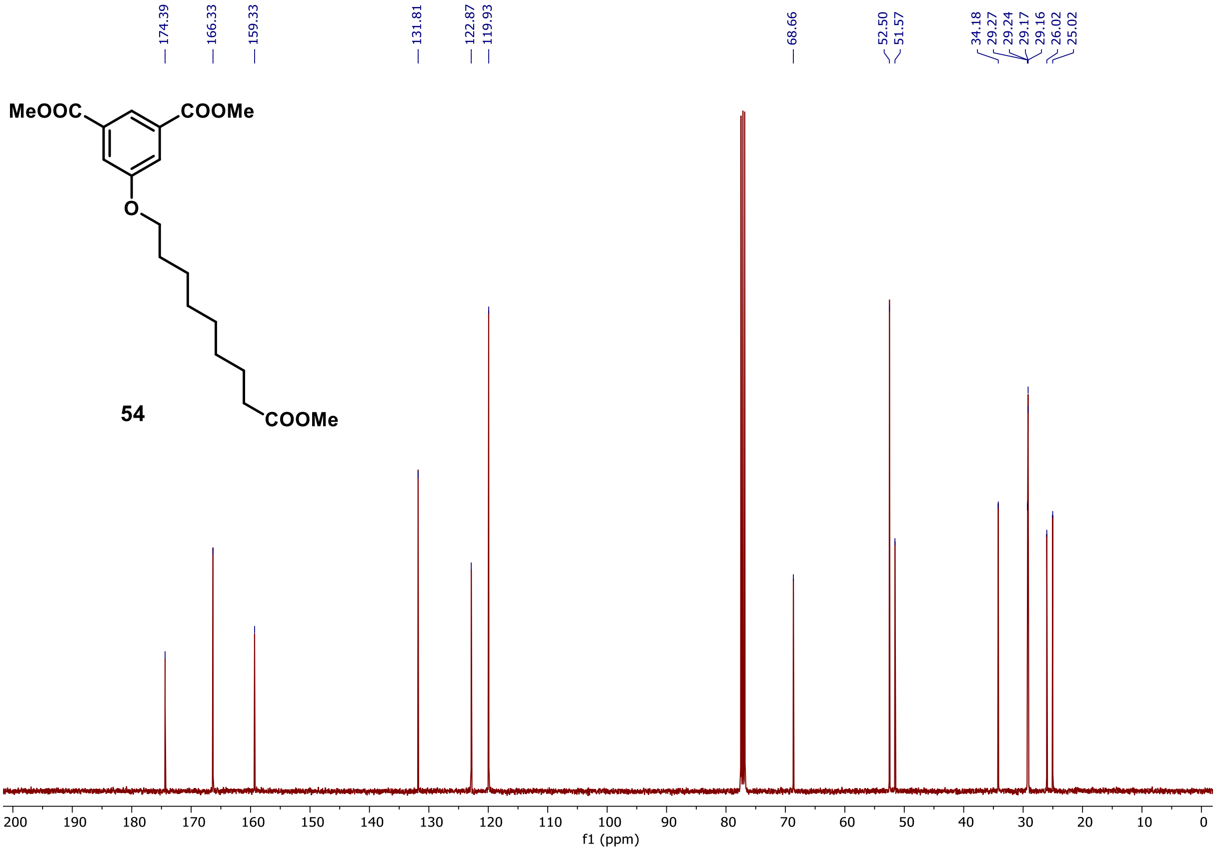

Figure S133: *^{13}C NMR spectrum of triester **54** ($CDCl_3$, 101 MHz).*

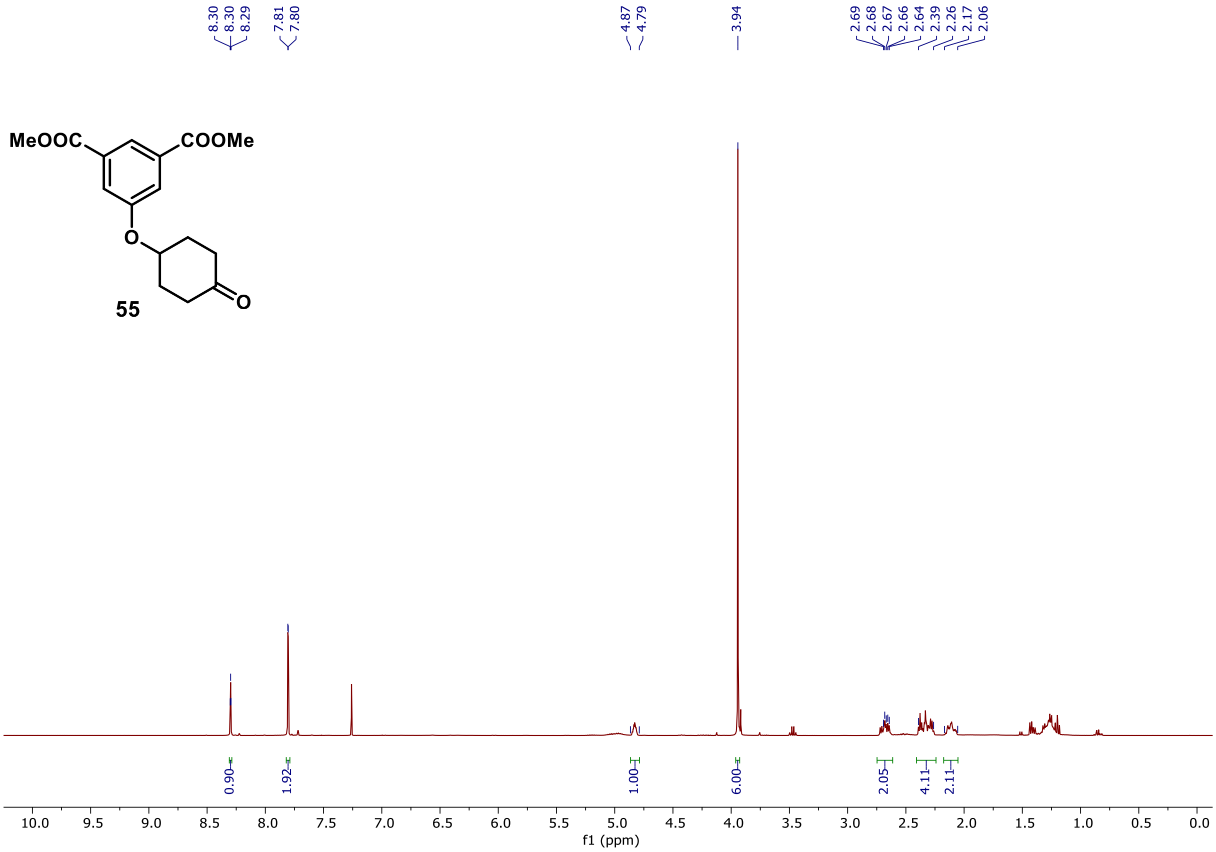

Figure S134: *^{1}H NMR spectra of ketone **55** (400 MHz, $CDCl_3$).*

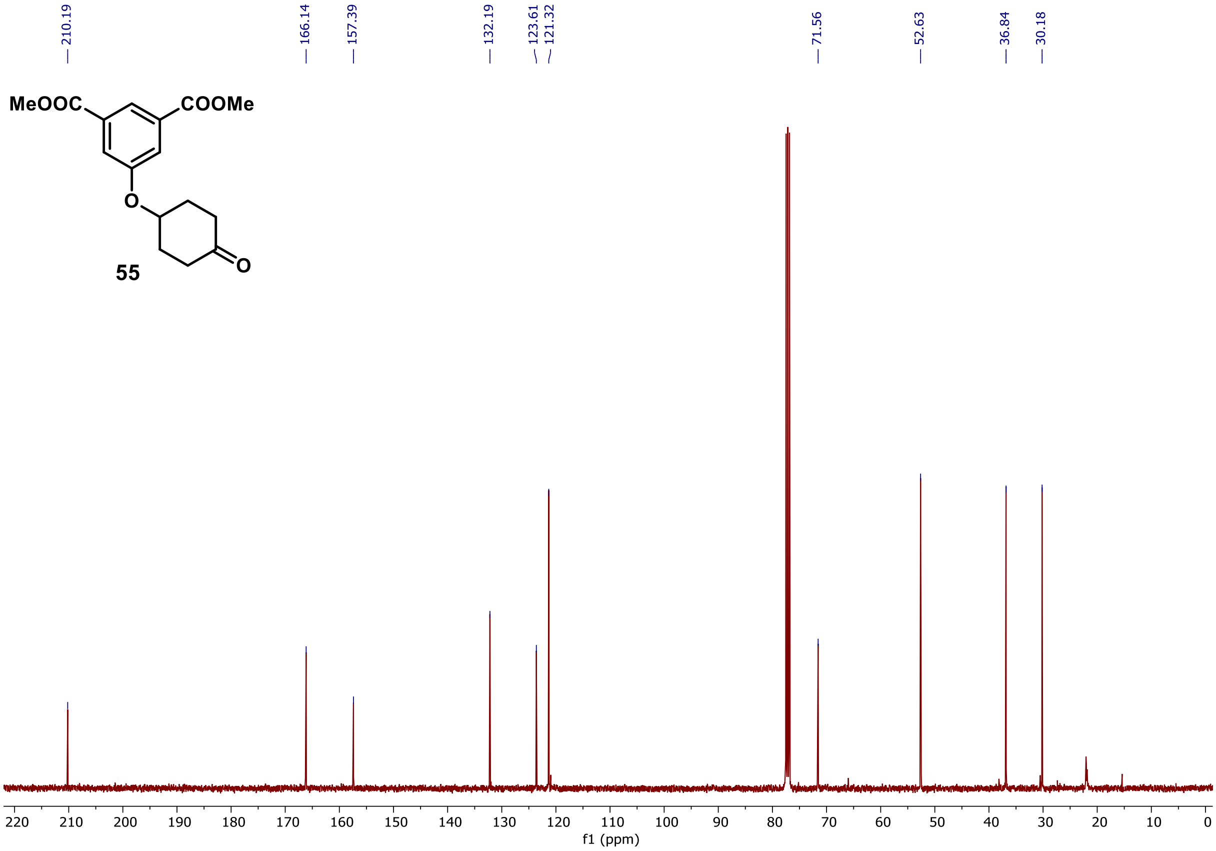

Figure S135: *^{13}C NMR spectrum of ketone **55** ($CDCl_3$, 101 MHz).*

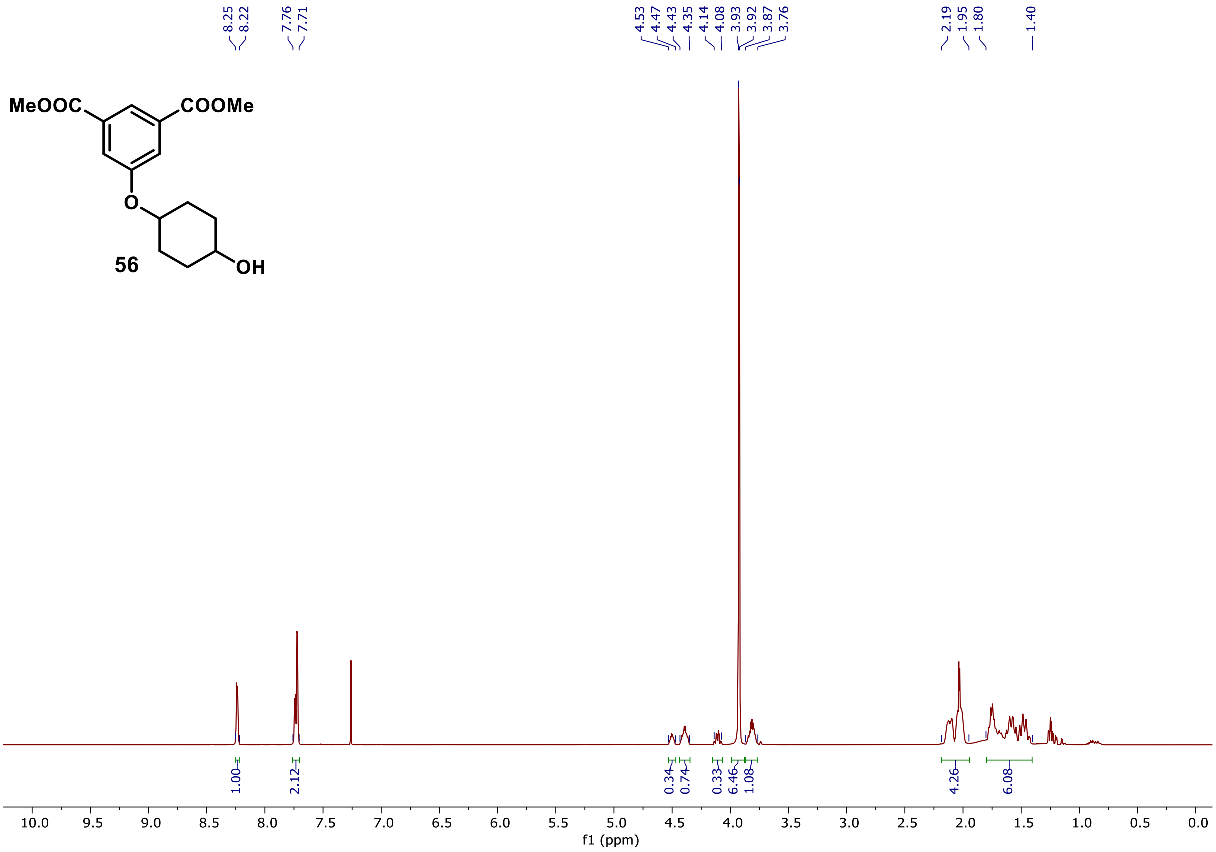

Figure S136: *^{1}H NMR spectra of alcohol* ***56*** *(400 MHz, $CDCl_3$).*

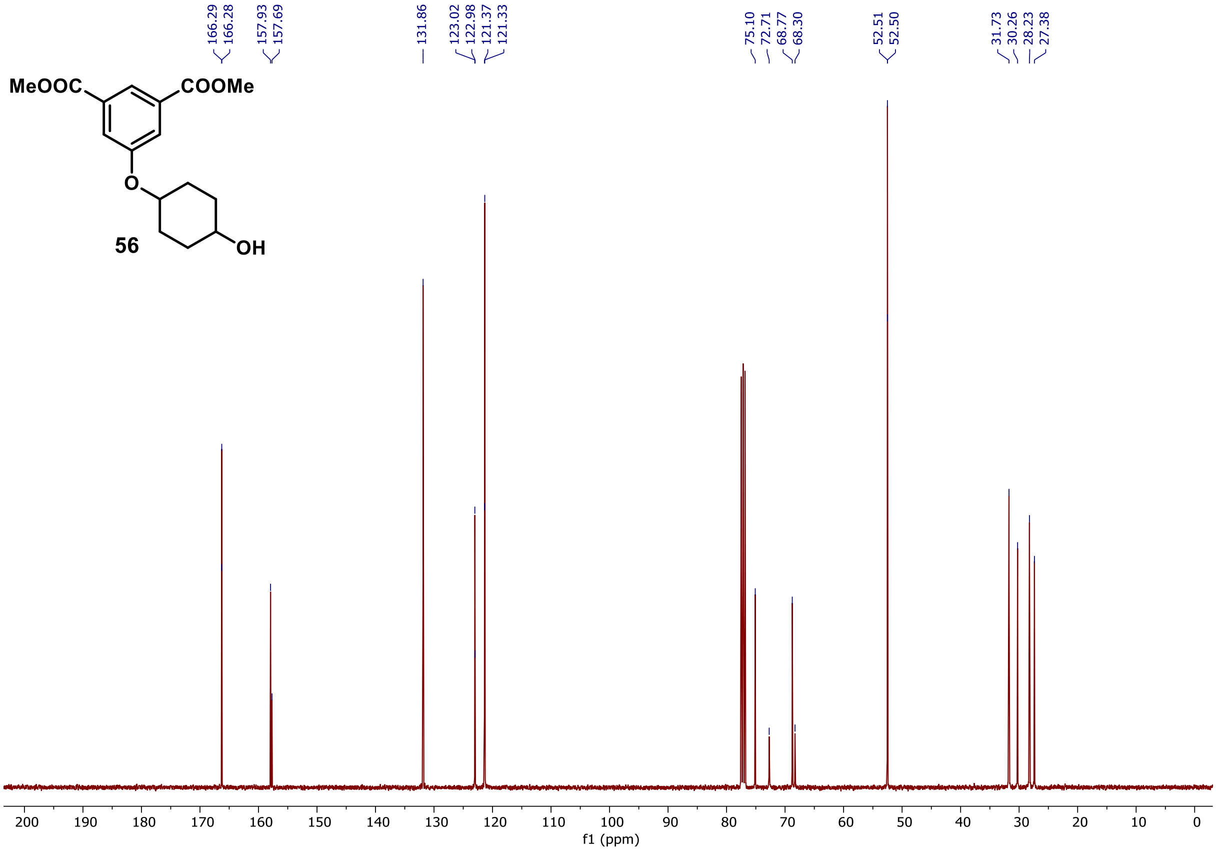

Figure S137: *^{13}C NMR spectrum of alcohol* ***56*** *($CDCl_3$, 101 MHz)****.***

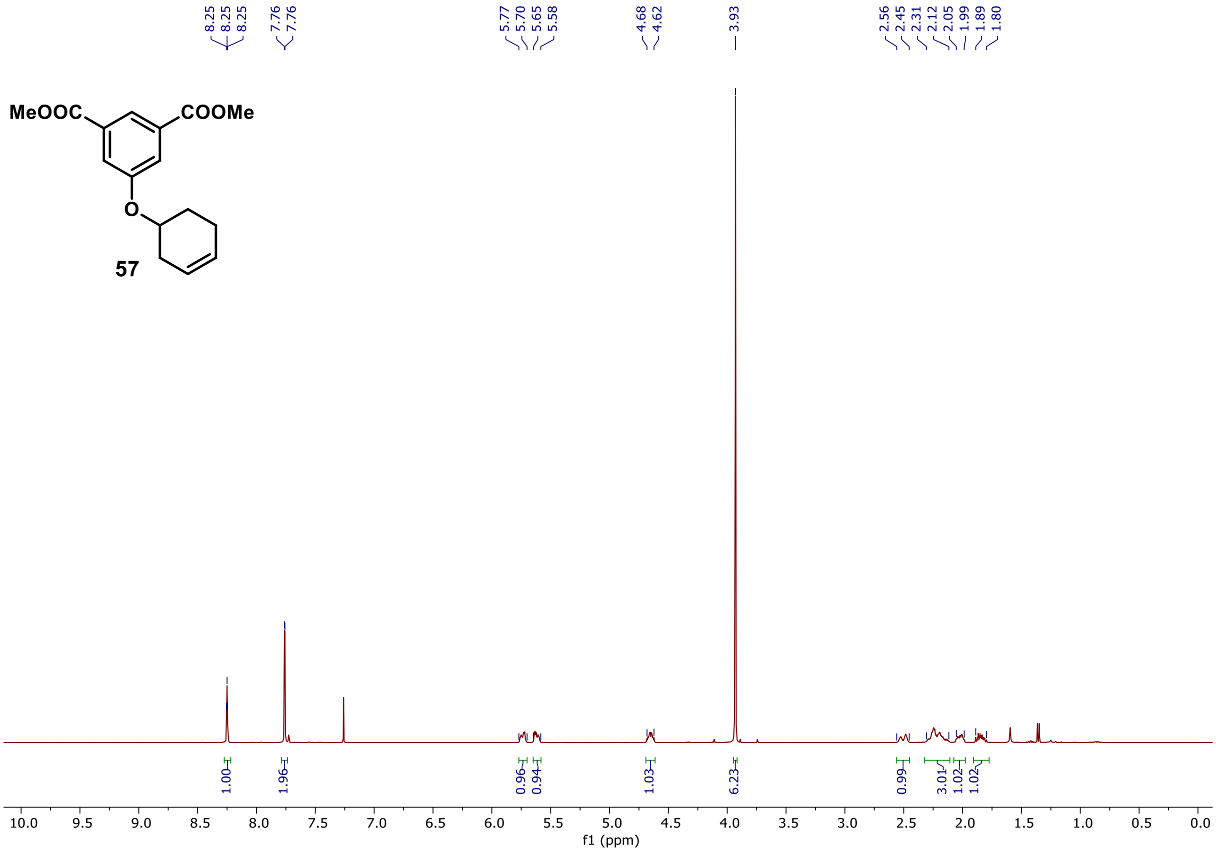

Figure S138: *^{1}H NMR spectra of alkene **57** (400 MHz, $CDCl_3$).*

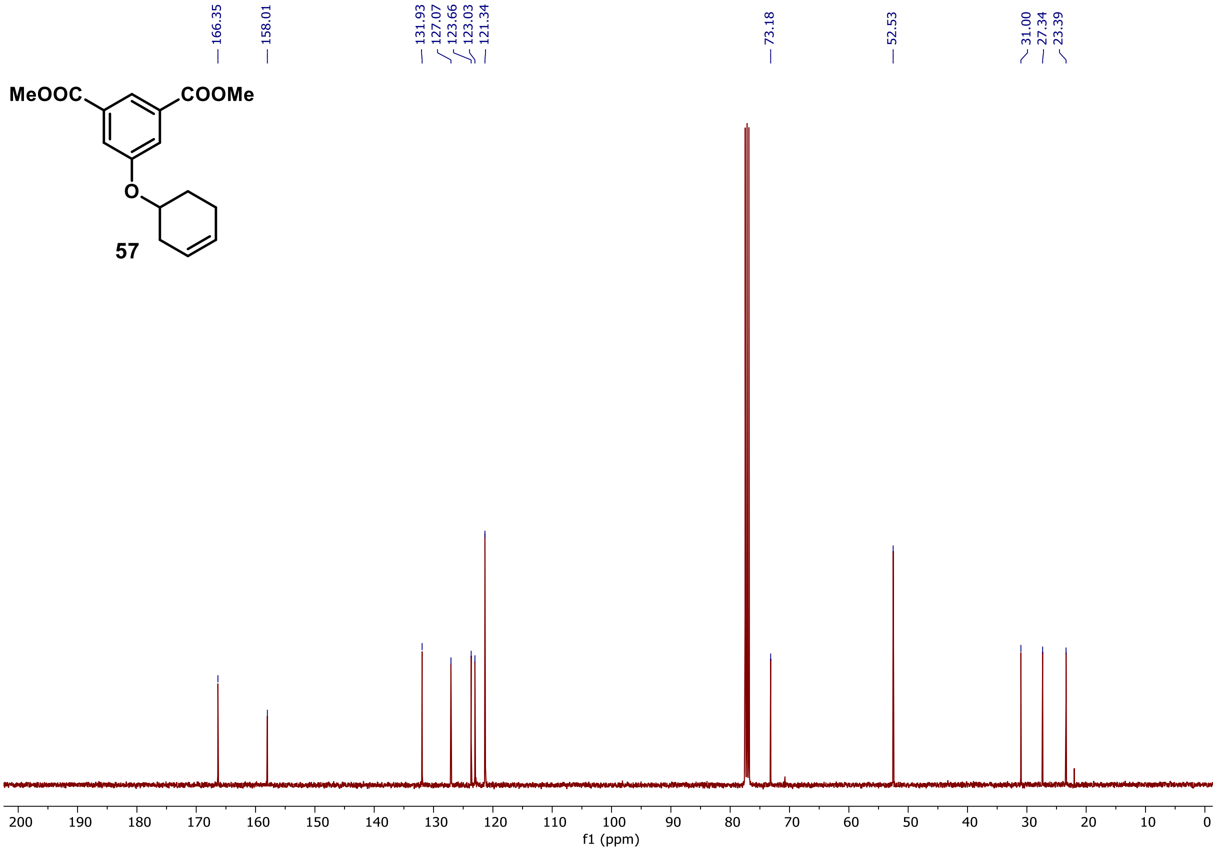

Figure S139: *^{13}C NMR spectrum of alkene **57** ($CDCl_3$, 101 MHz).*

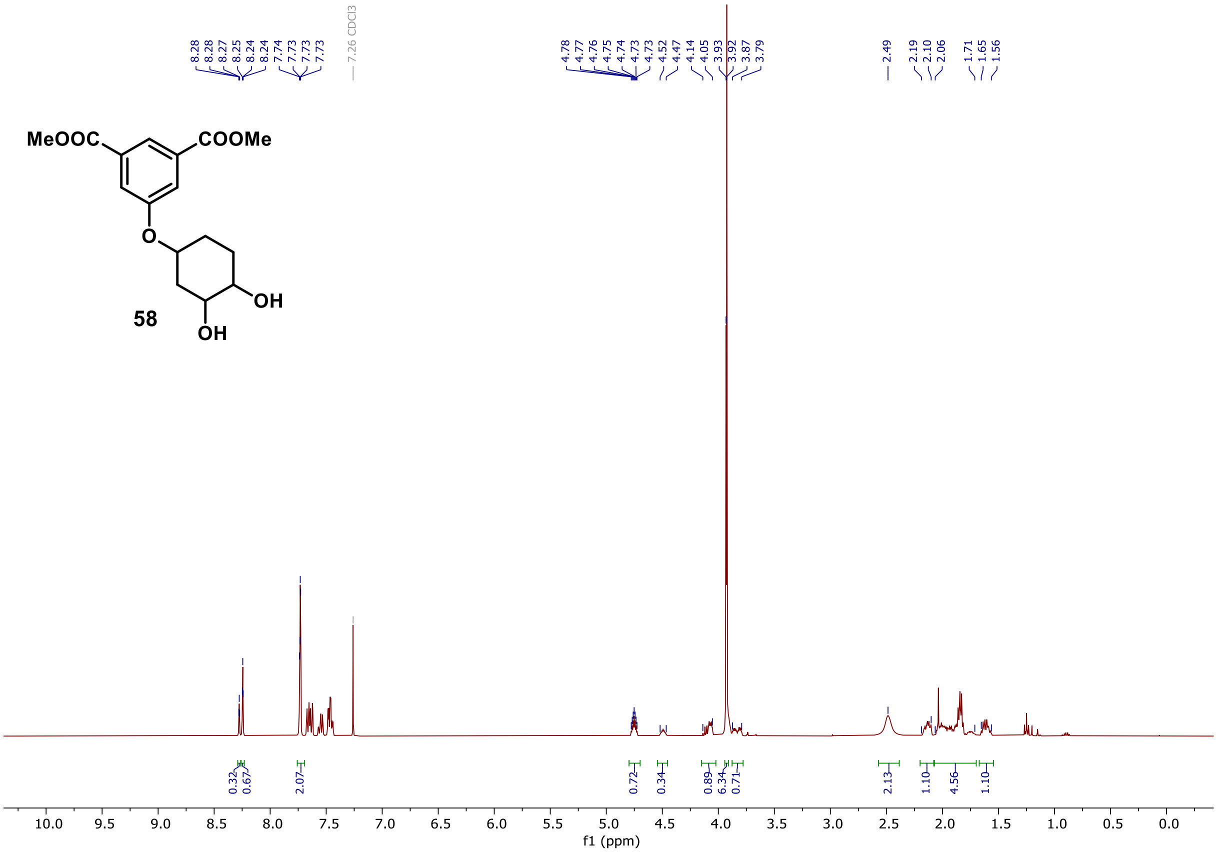

Figure S140: *^{1}H NMR spectra of diol* ***58*** *(400 MHz, $CDCl_3$).*

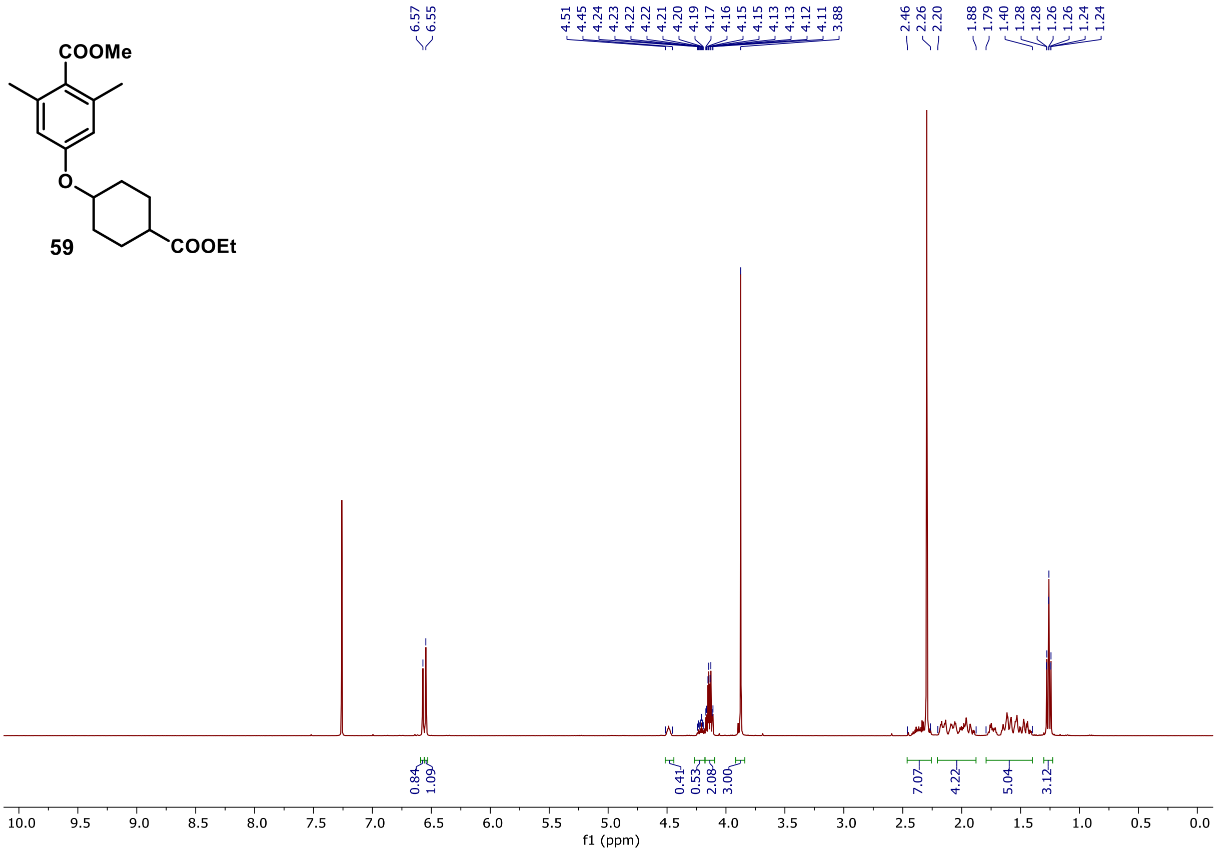

Figure S141: *^{1}H NMR spectra of diester* ***59*** *(400 MHz, CDCl₃).*

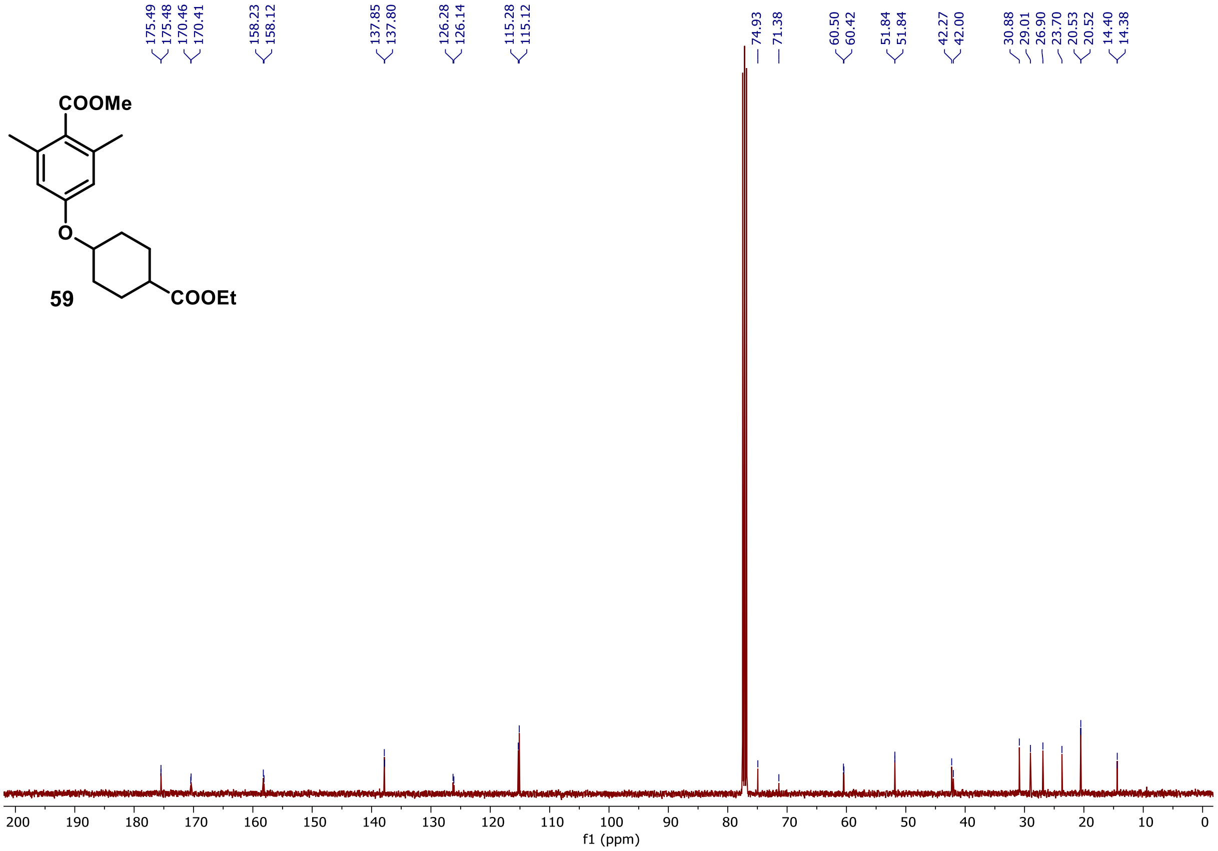

Figure S142: *^{13}C NMR spectrum of diester* ***59*** *($CDCl_3$, 101 MHz)****.***

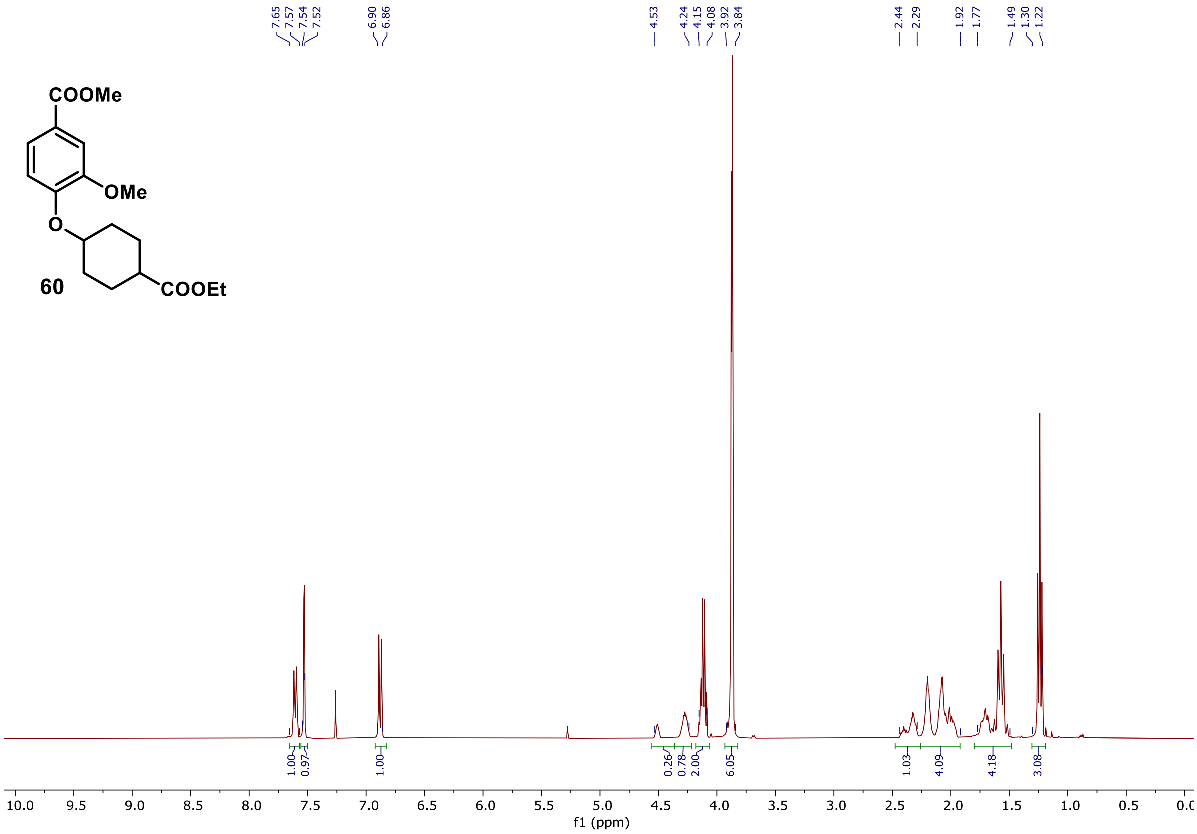

Figure S143: *^{1}H NMR spectra of methoxy ether **60** (400 MHz, $CDCl_3$).*

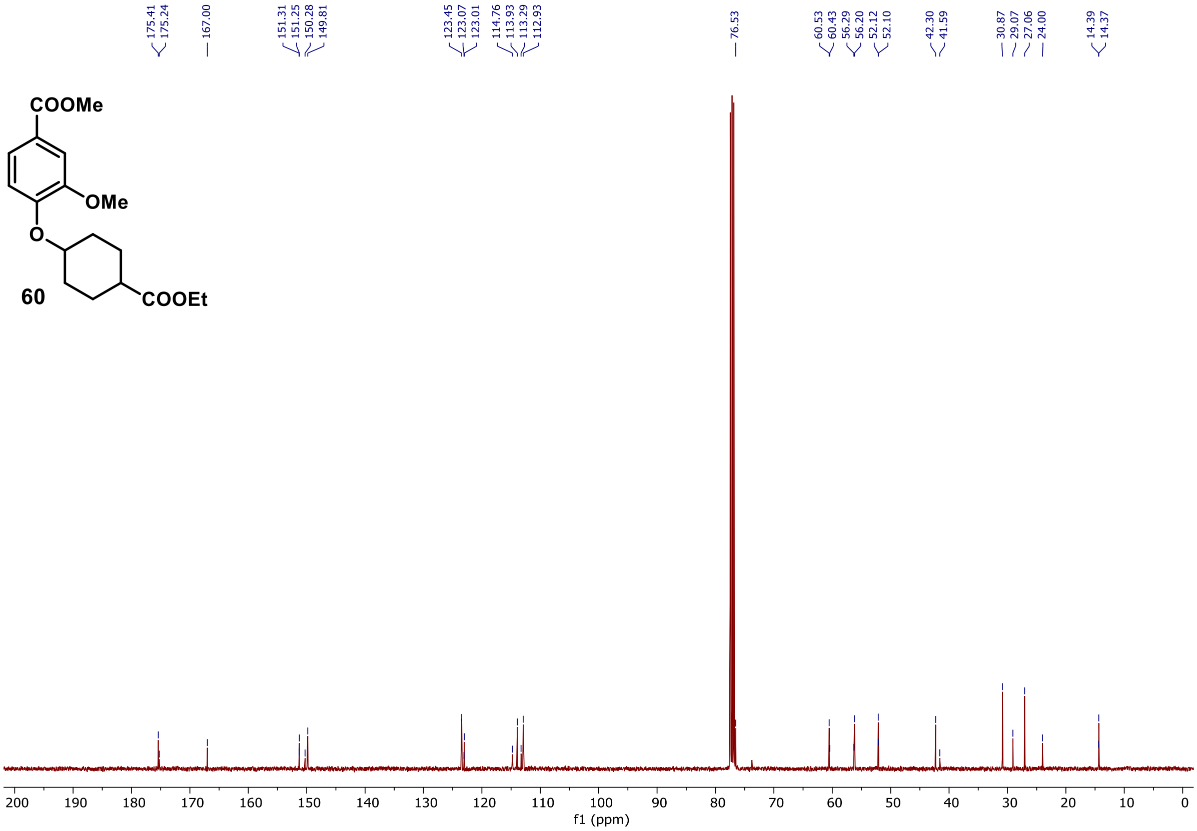

Figure S144: *^{13}C NMR spectrum of methoxy ether **60** ($CDCl_3$, 101 MHz).*

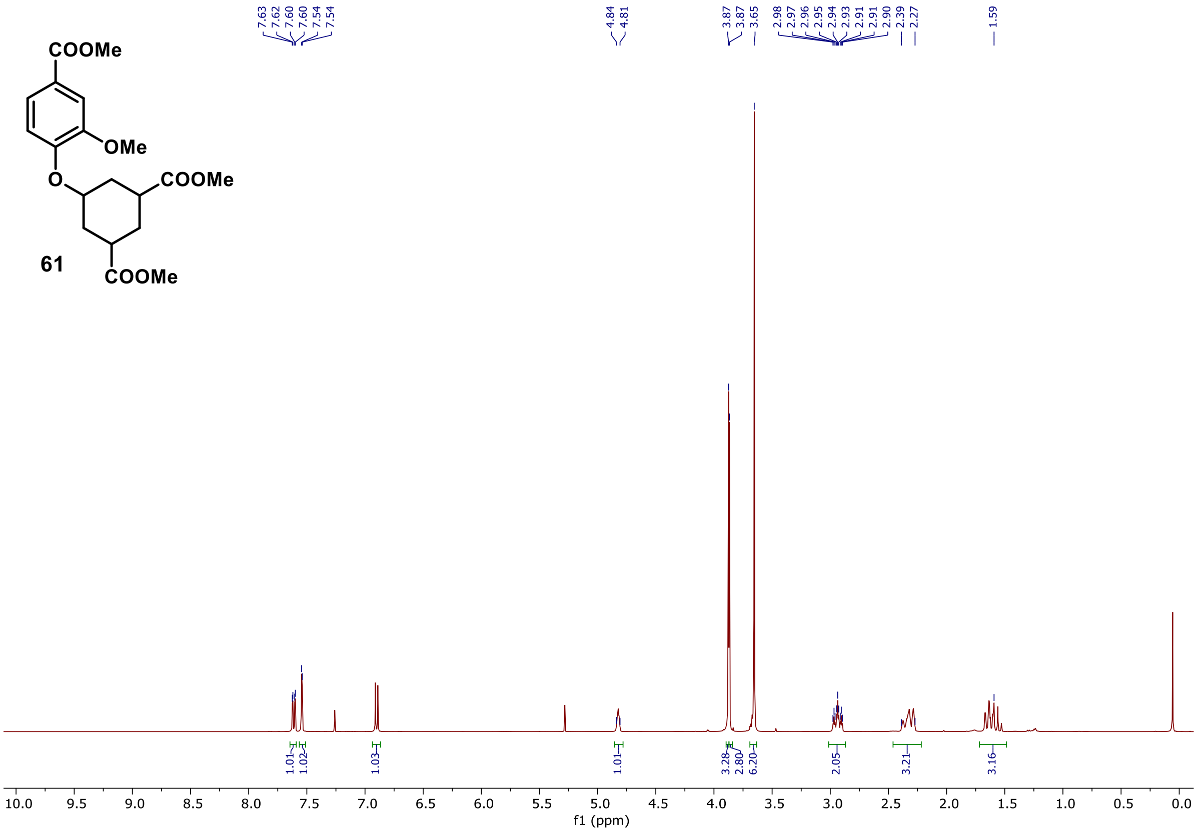

Figure S145: *^{1}H NMR spectra of methoxy ether **61** (400 MHz, $CDCl_3$).*

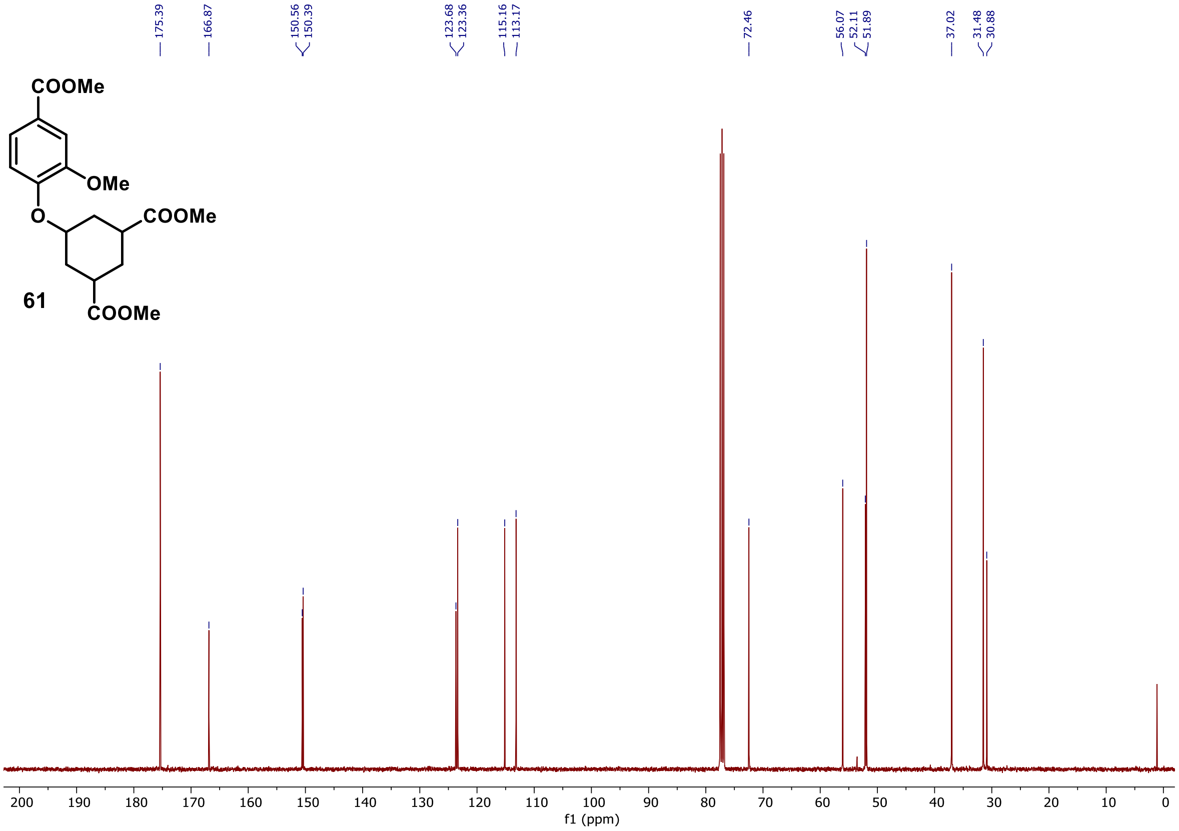

Figure S146: *^{13}C NMR spectrum of methoxy ether **61** ($CDCl_3$, 101 MHz)*

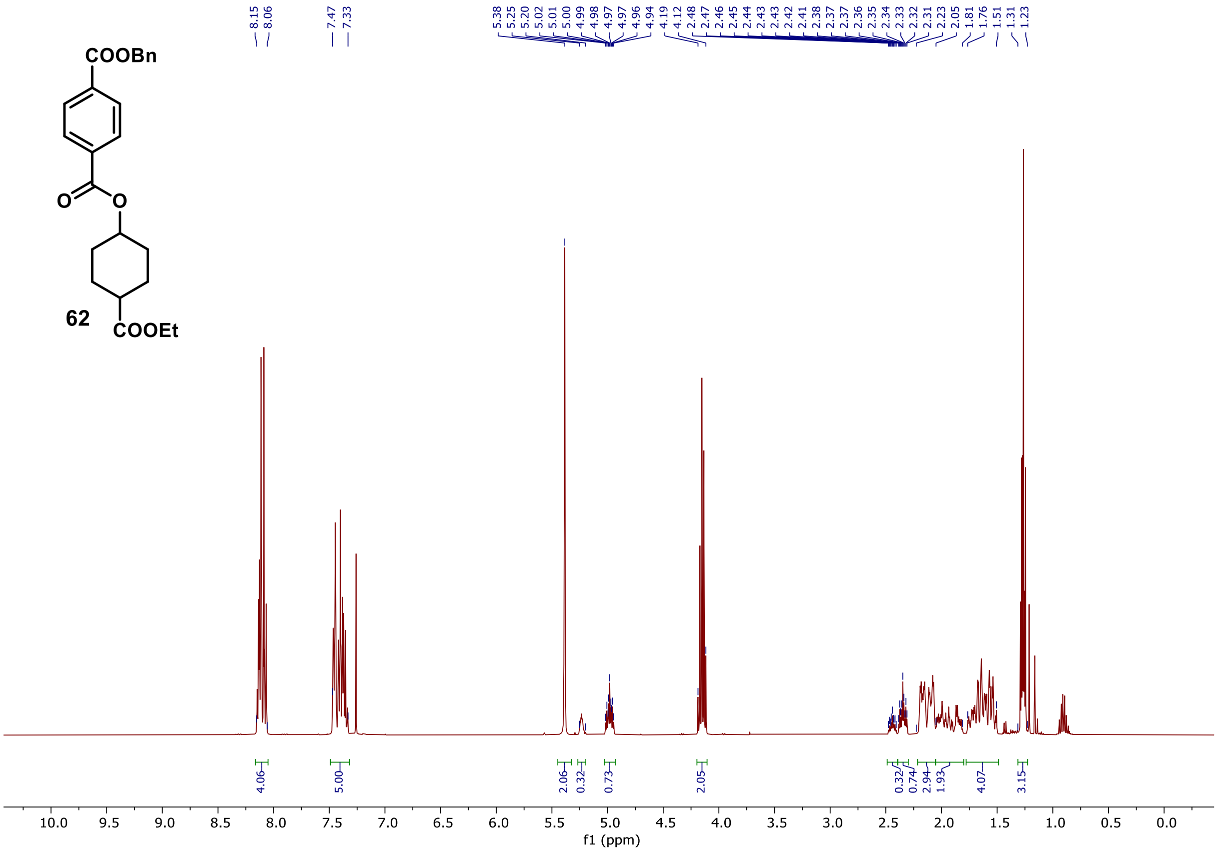

Figure S147: *^{1}H NMR spectra of triester* ***62*** *(400 MHz, $CDCl_3$).*

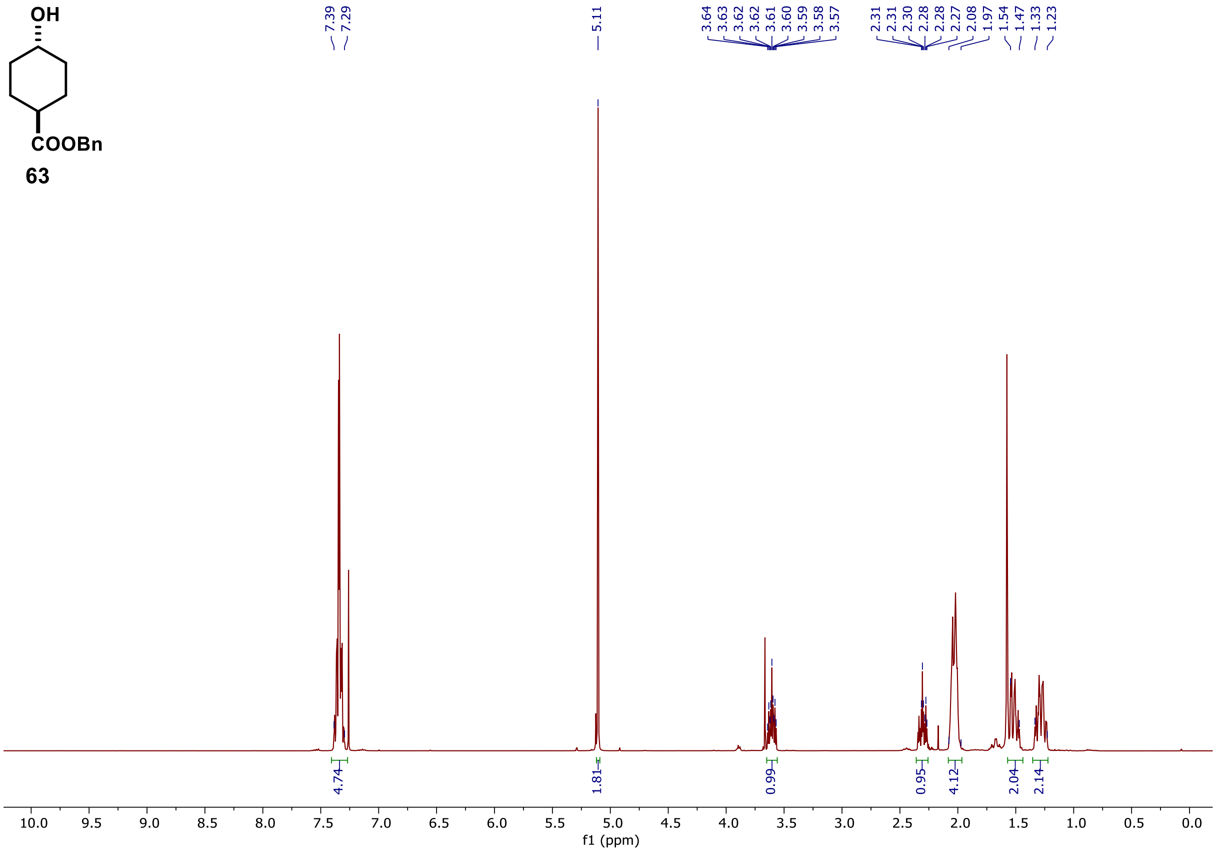

Figure S148: *^{1}H NMR spectra of alcohol **63** (400 MHz, CDCl$_3$).*

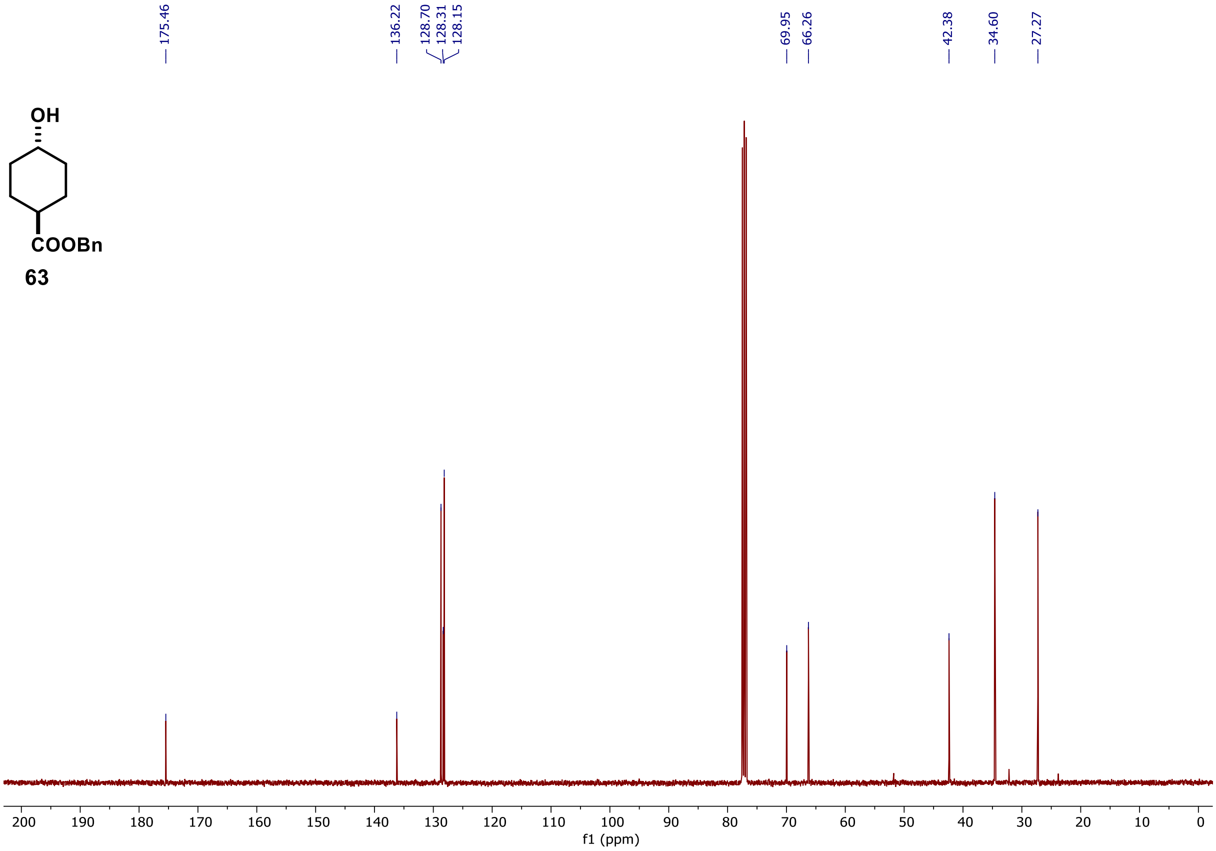

Figure S149: *^{13}C NMR spectrum of alcohol **63** (CDCl$_3$, 101 MHz)**.***

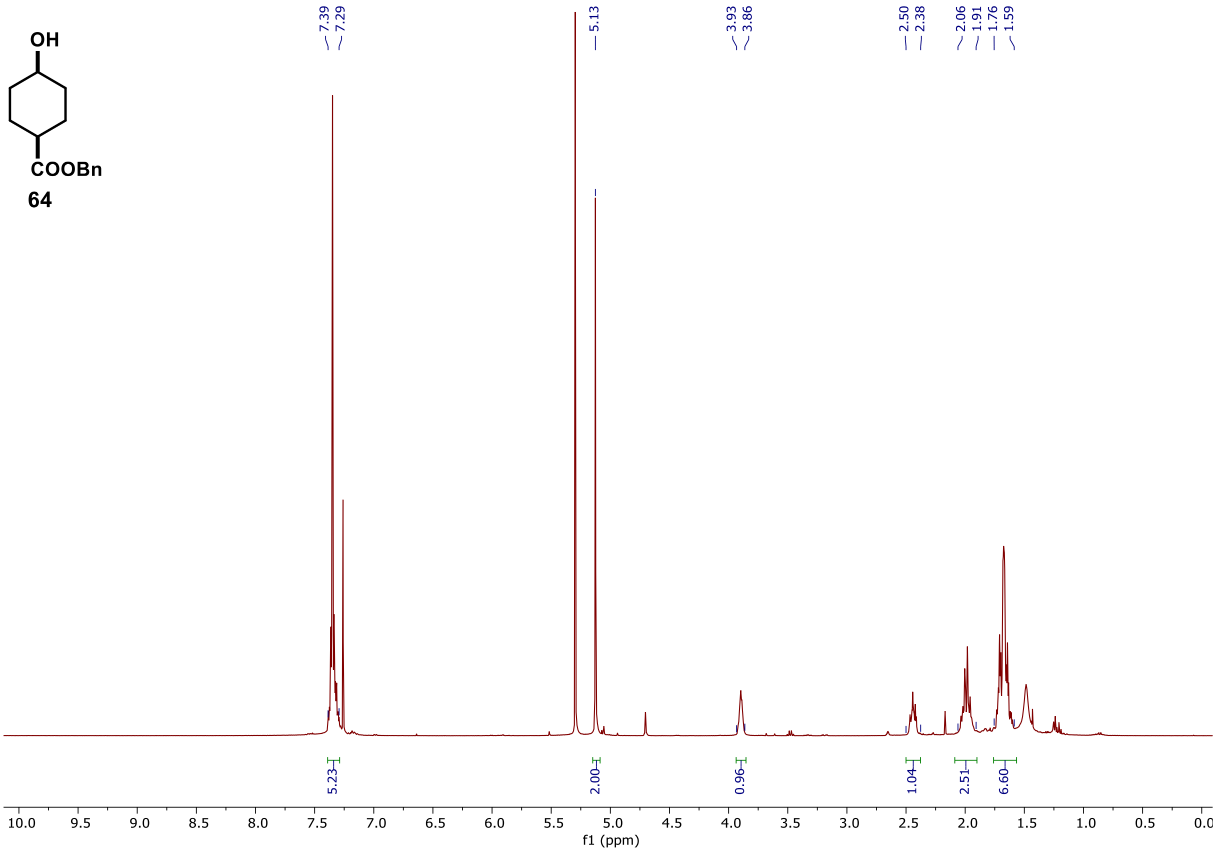

Figure S150: *^{1}H NMR spectra of alcohol **64** (400 MHz, $CDCl_3$).*

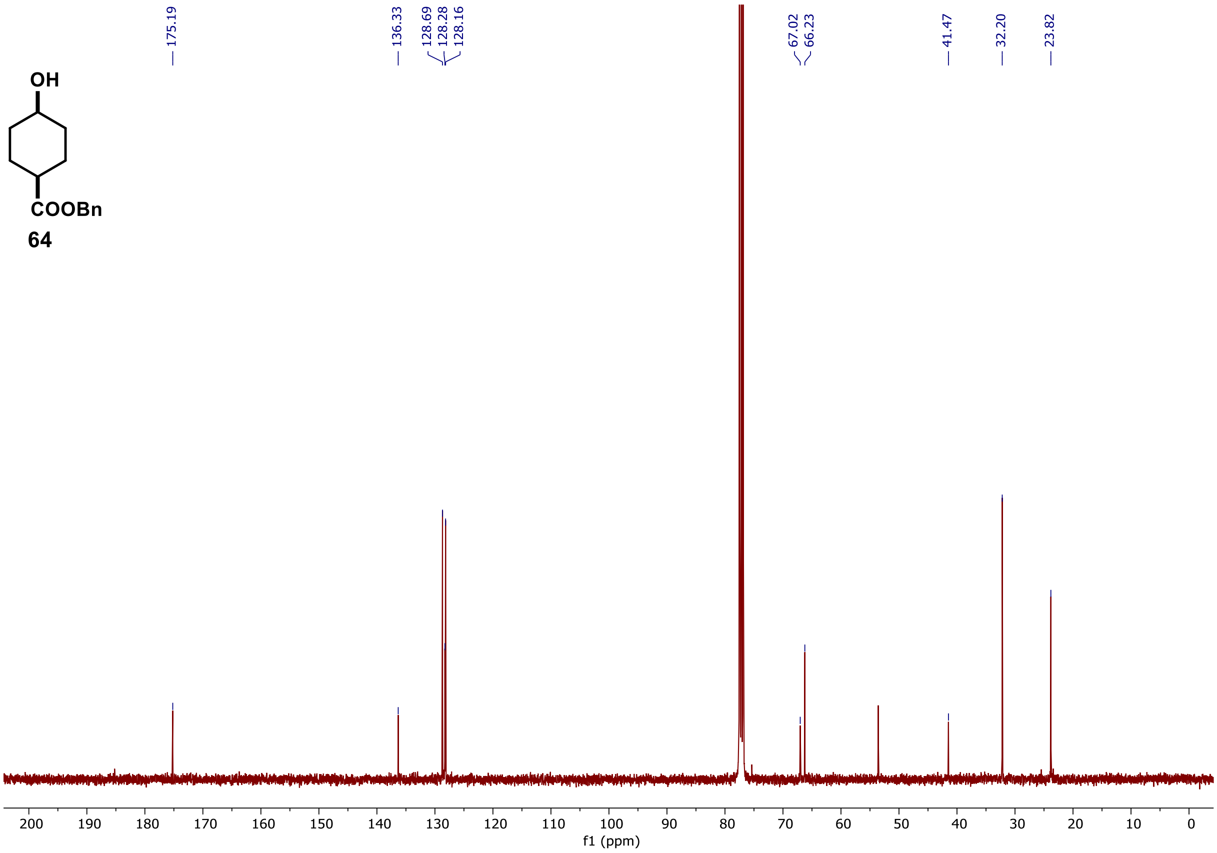

Figure S151: *^{13}C NMR spectrum of alcohol **64** ($CDCl_3$, 101 MHz)**.***

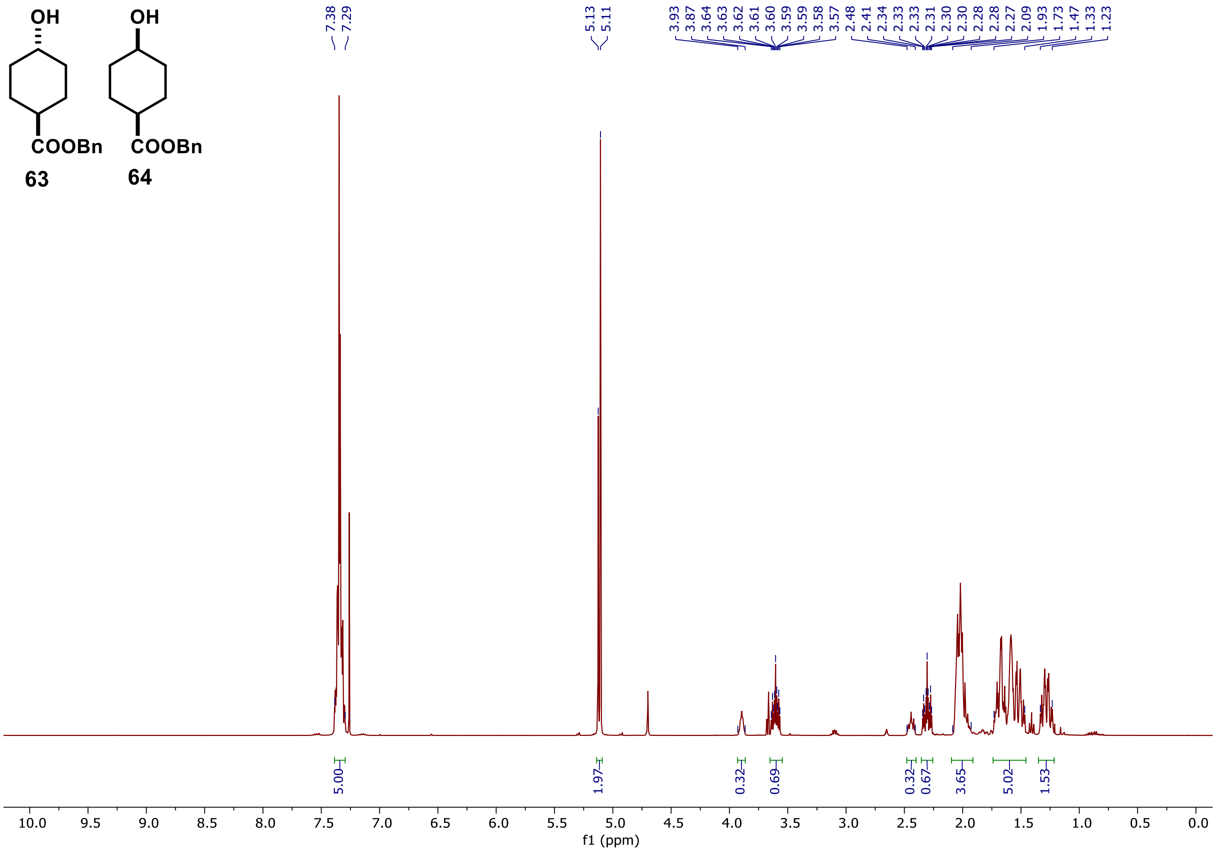

Figure S152: *^{1}H NMR spectra of alcohols **63** and **64** (400 MHz, $CDCl_3$).*

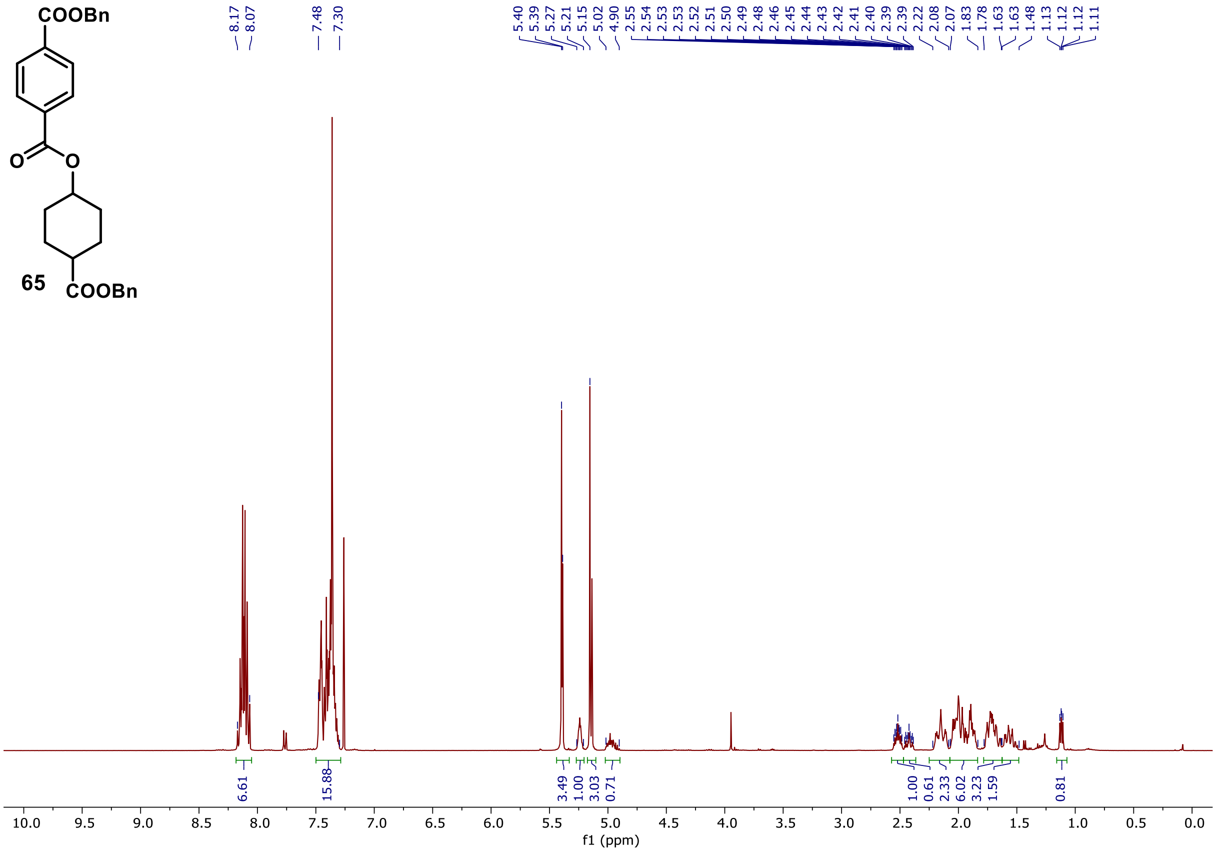

Figure S153: *^{1}H NMR spectra of triester **65** (400 MHz, $CDCl_3$).*

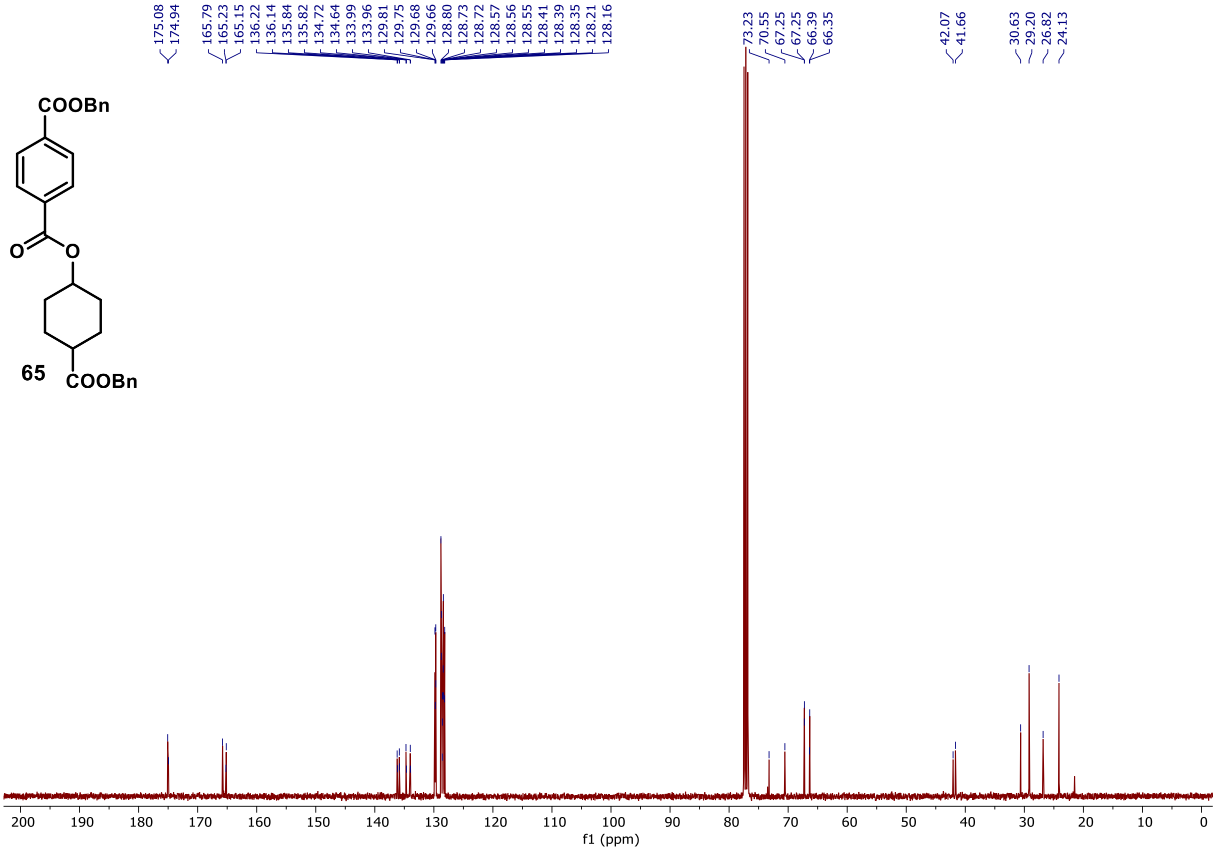

Figure S154: *^{13}C NMR spectrum of triester **65** ($CDCl_3$, 101 MHz)**.***

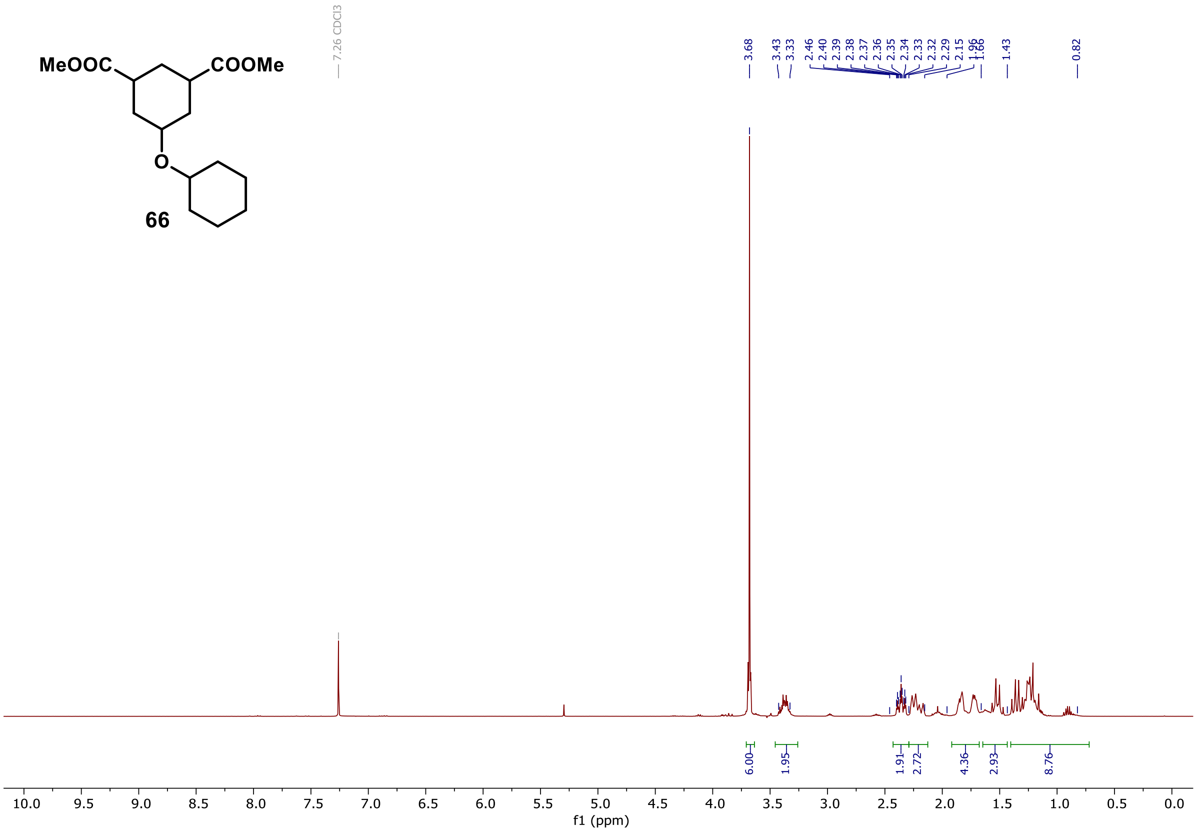

Figure S155: *^{1}H NMR spectra of diester **66** (400 MHz, $CDCl_3$).*

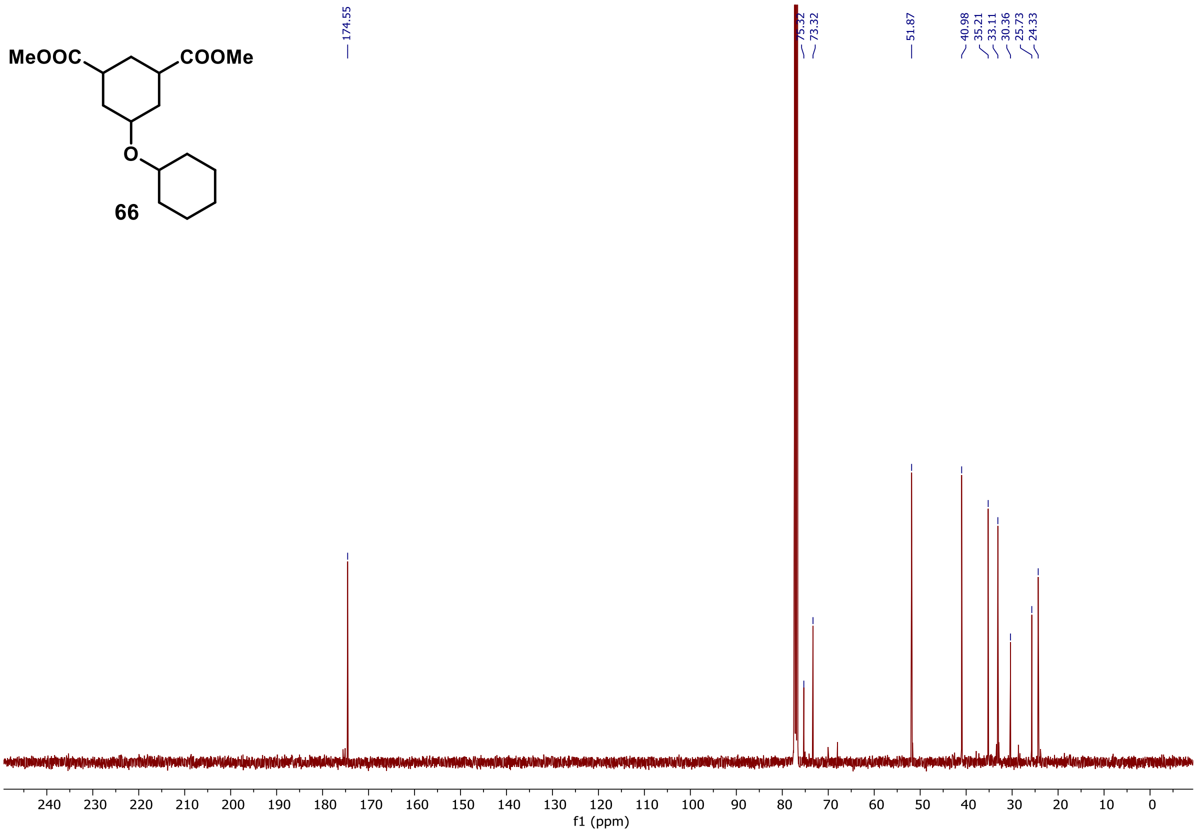

Figure S156: *^{13}C NMR spectrum of diester **66** ($CDCl_3$, 101 MHz)**.***

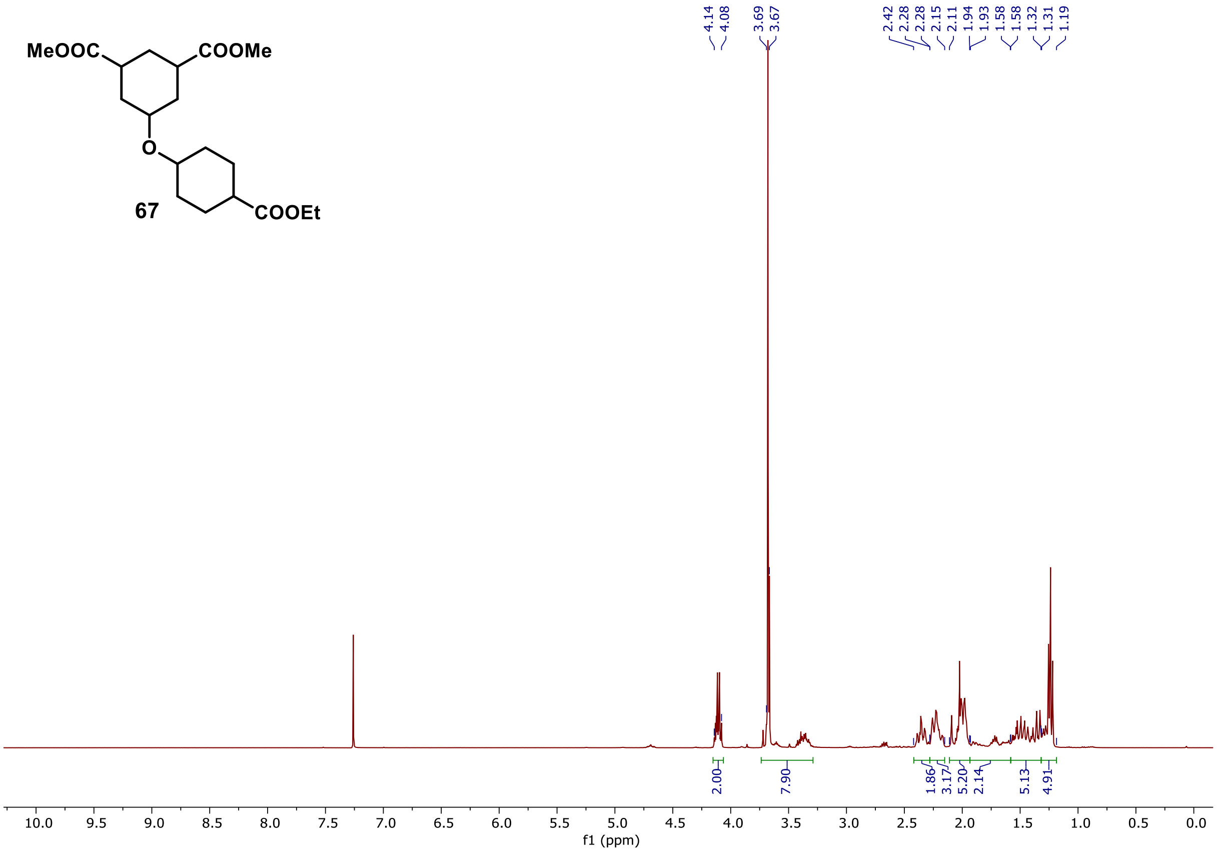

Figure S157: *^{1}H NMR spectra of triester* ***67*** *(400 MHz, $CDCl_3$).*

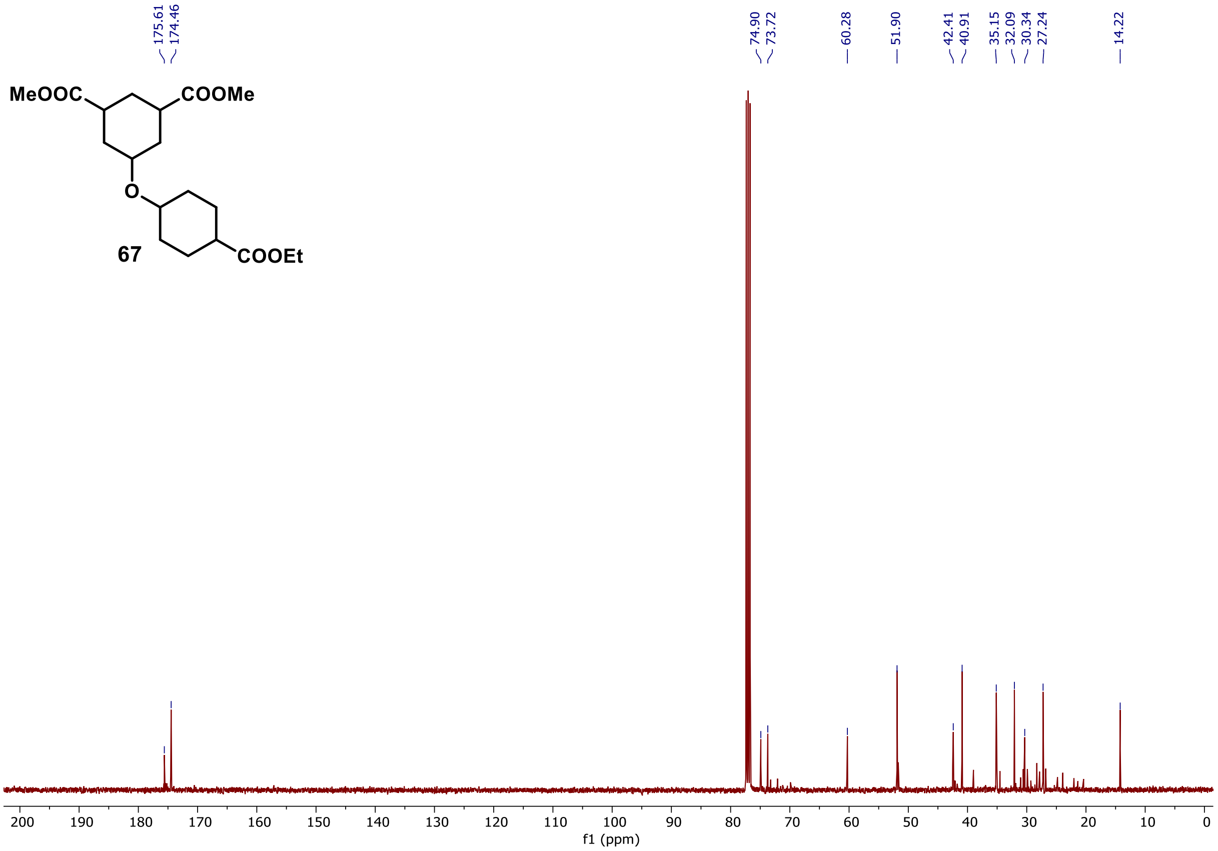

Figure S158: *^{13}C NMR spectrum of triester* ***67*** *($CDCl_3$, 101 MHz)****.***

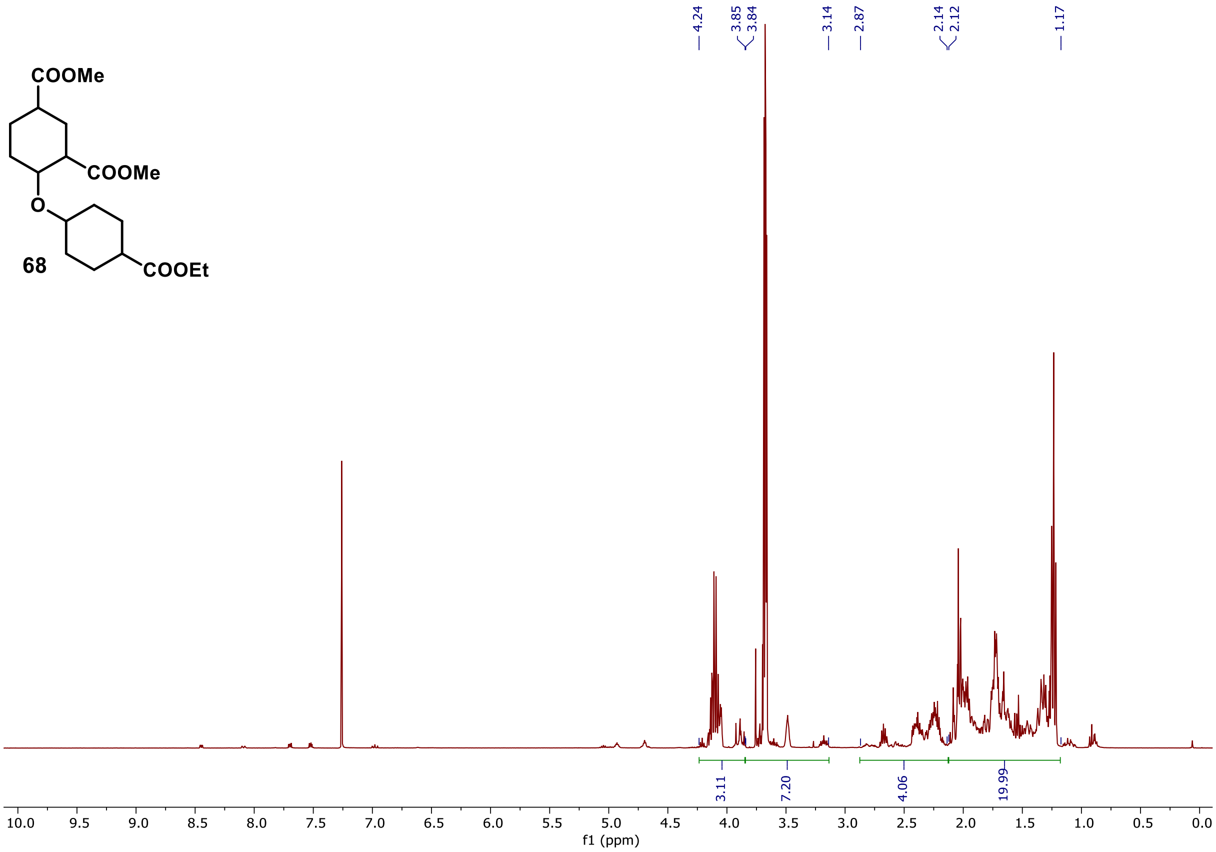

Figure S59: *^{1}H NMR spectra of triester* ***68*** *(400 MHz, $CDCl_3$).*

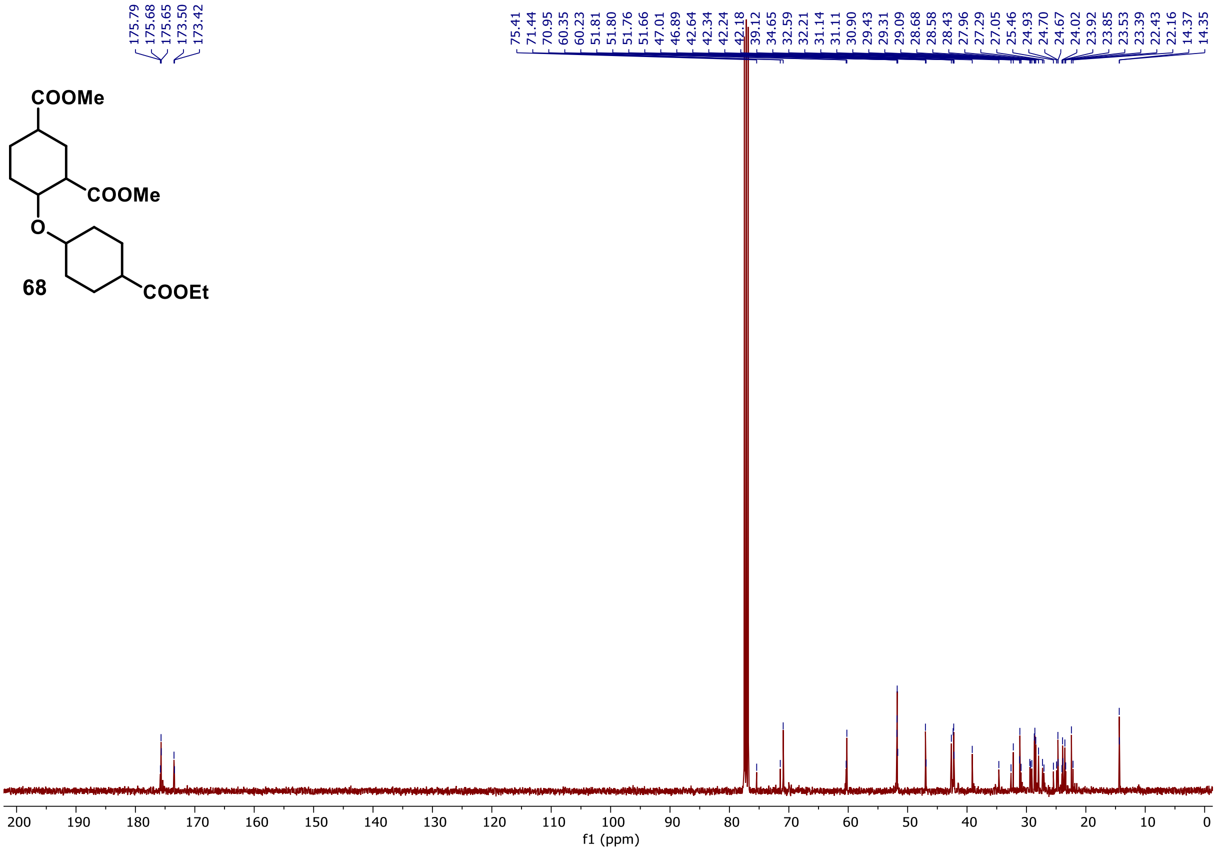

Figure S160: *^{13}C NMR spectrum of triester* ***68*** *($CDCl_3$, 101 MHz)****.***

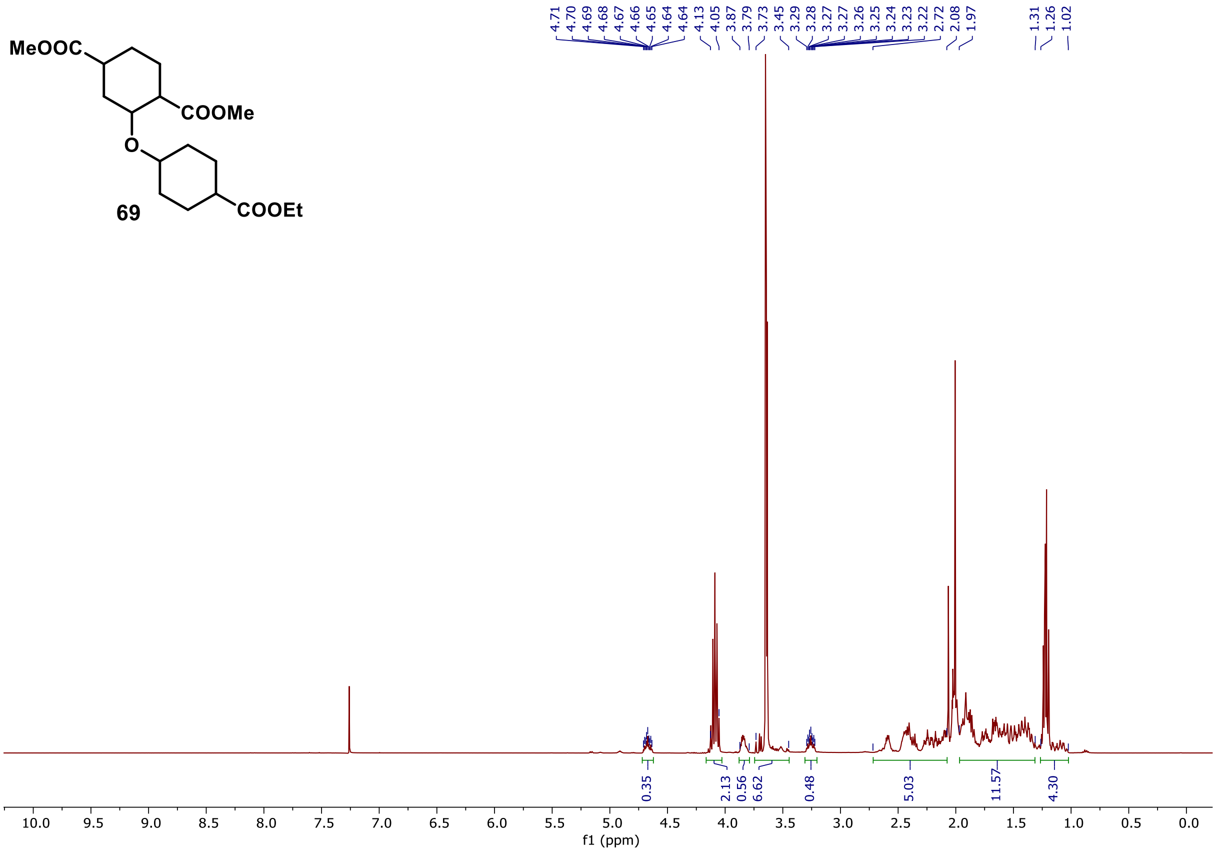

Figure S161: *^{1}H NMR spectra of triester **69** (400 MHz, $CDCl_3$).*

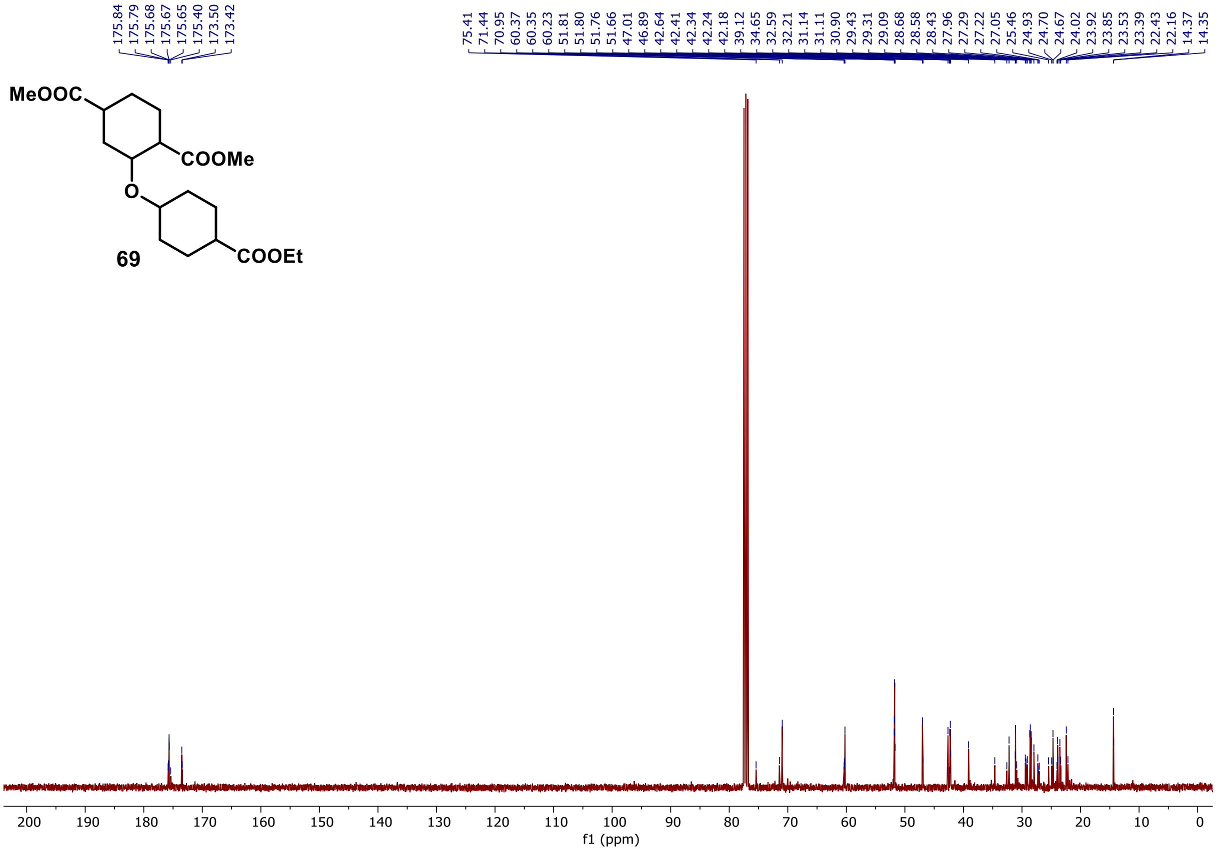

Figure S162: *^{13}C NMR spectrum of triester **69** ($CDCl_3$, 101 MHz)**.***

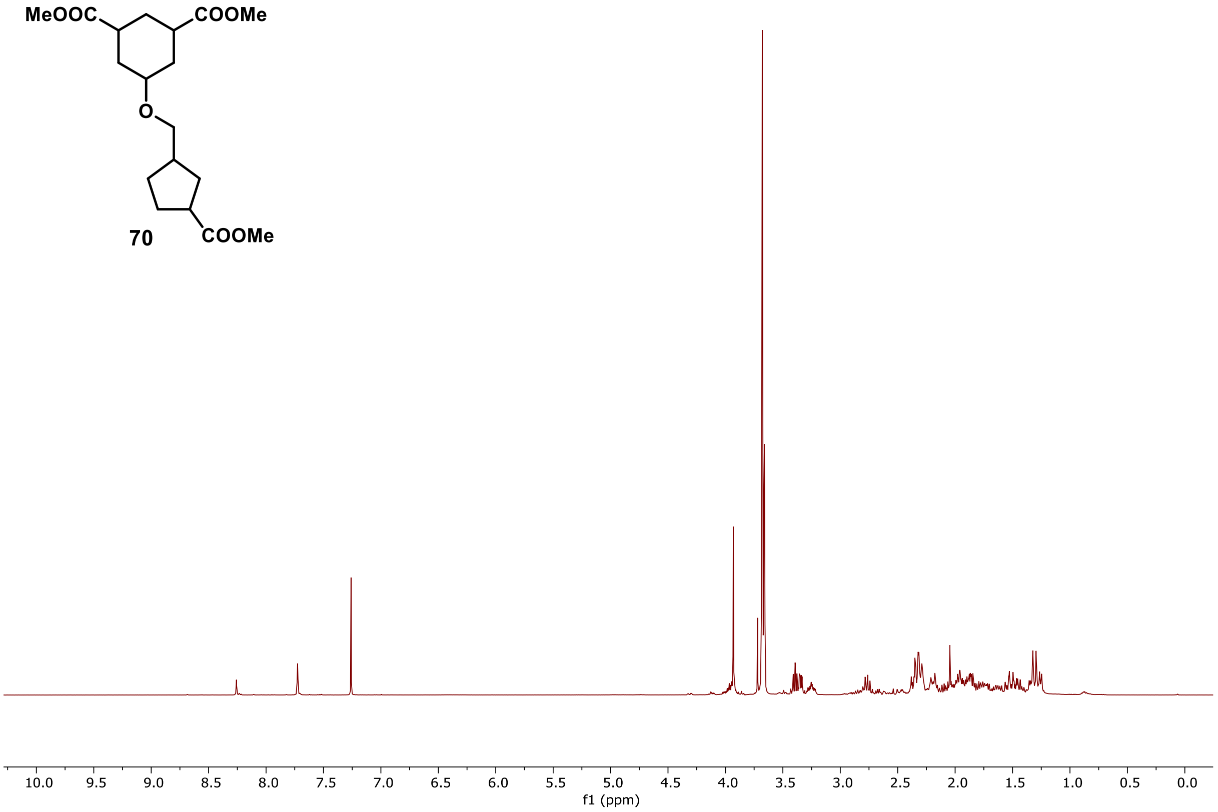

Figure S163: *Crude ^{1}H NMR spectra of triester* ***70*** *(400 MHz, $CDCl_3$).*

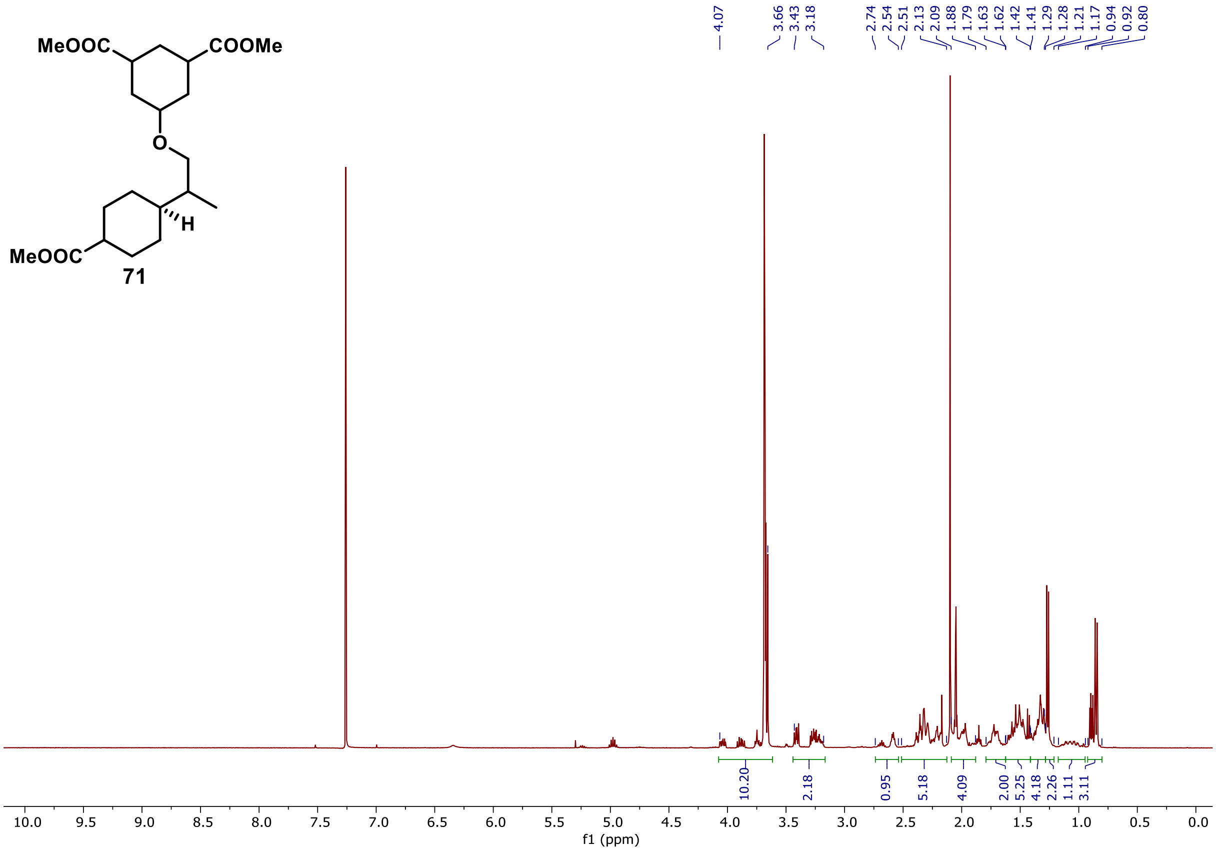

Figure S164: *¹H NMR spectra of triester* ***71*** *(400 MHz, CDCl₃).*

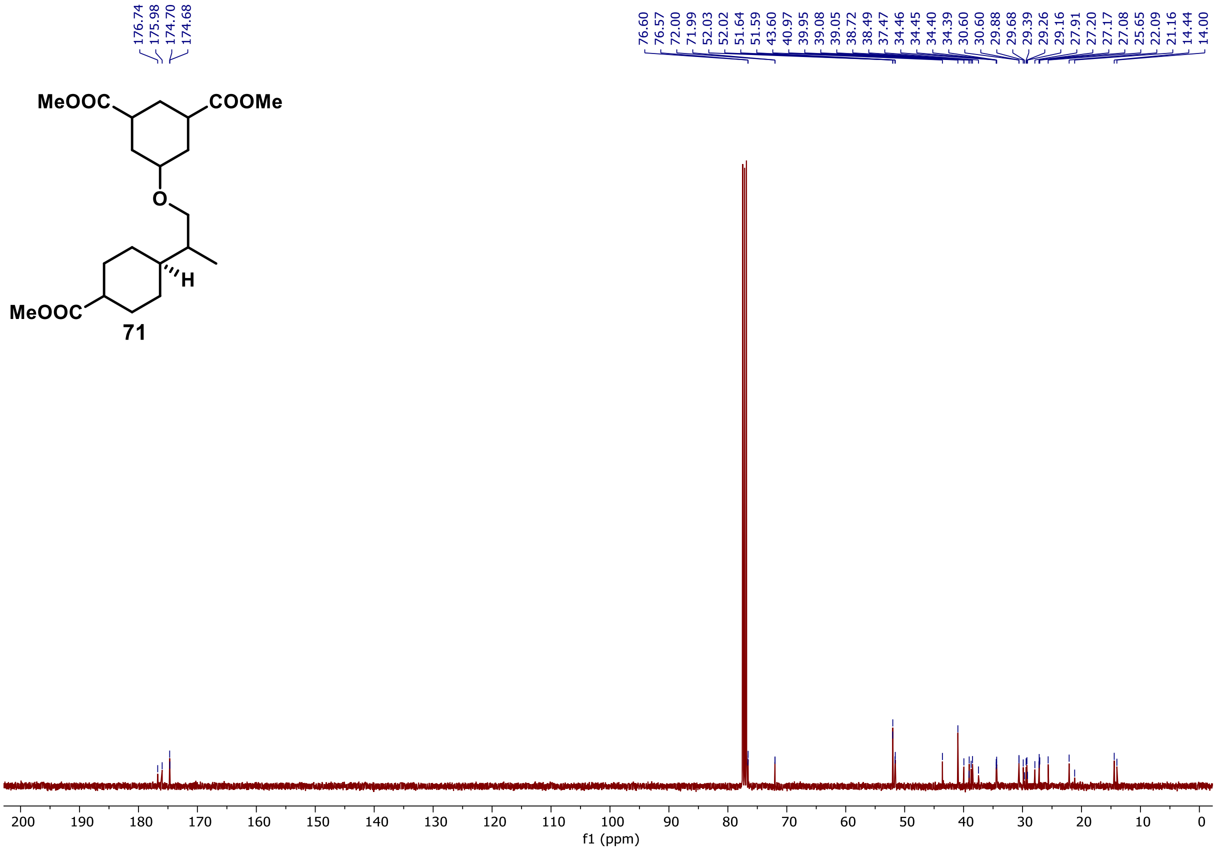

Figure S165: *^{13}C NMR spectrum of triester* ***71*** *($CDCl_3$, 101 MHz)****.***

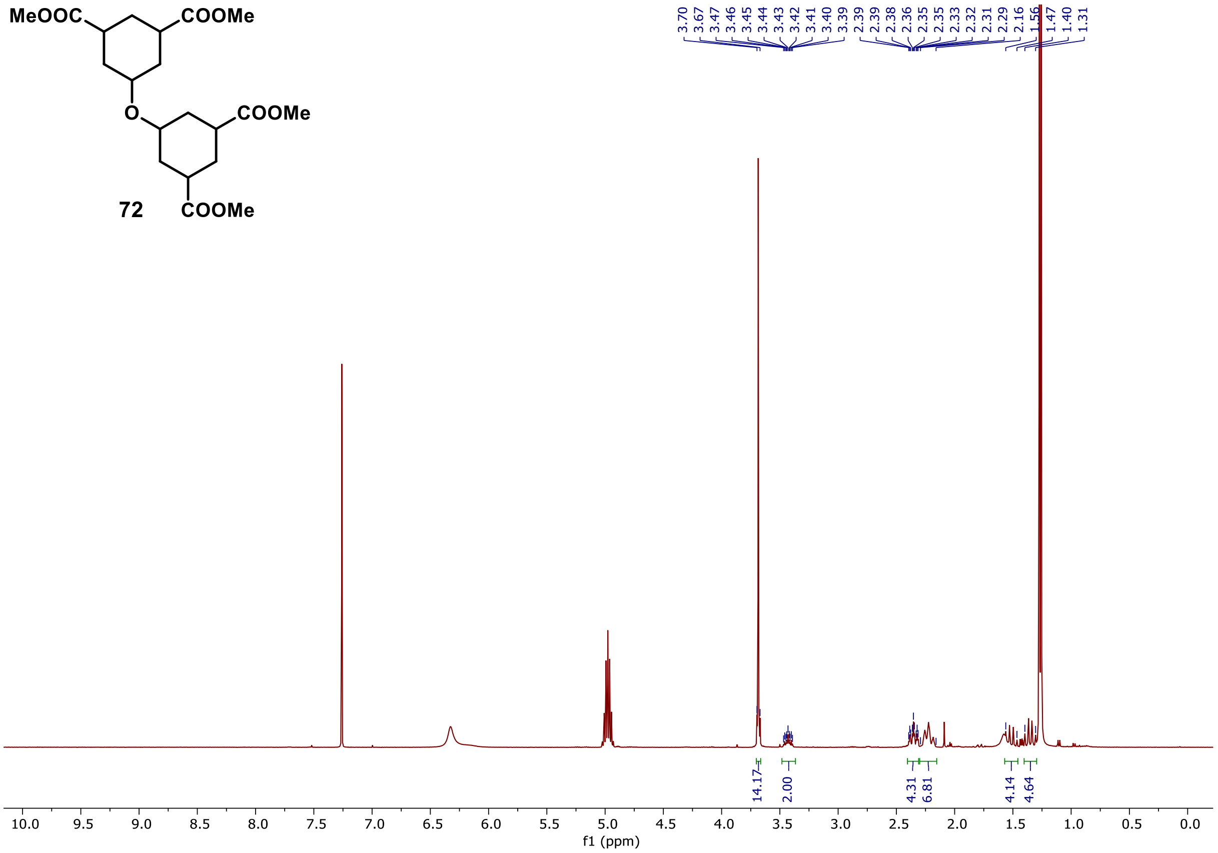

Figure S166: *^{1}H NMR spectra of tetraester* ***72*** *(400 MHz, $CDCl_3$).*

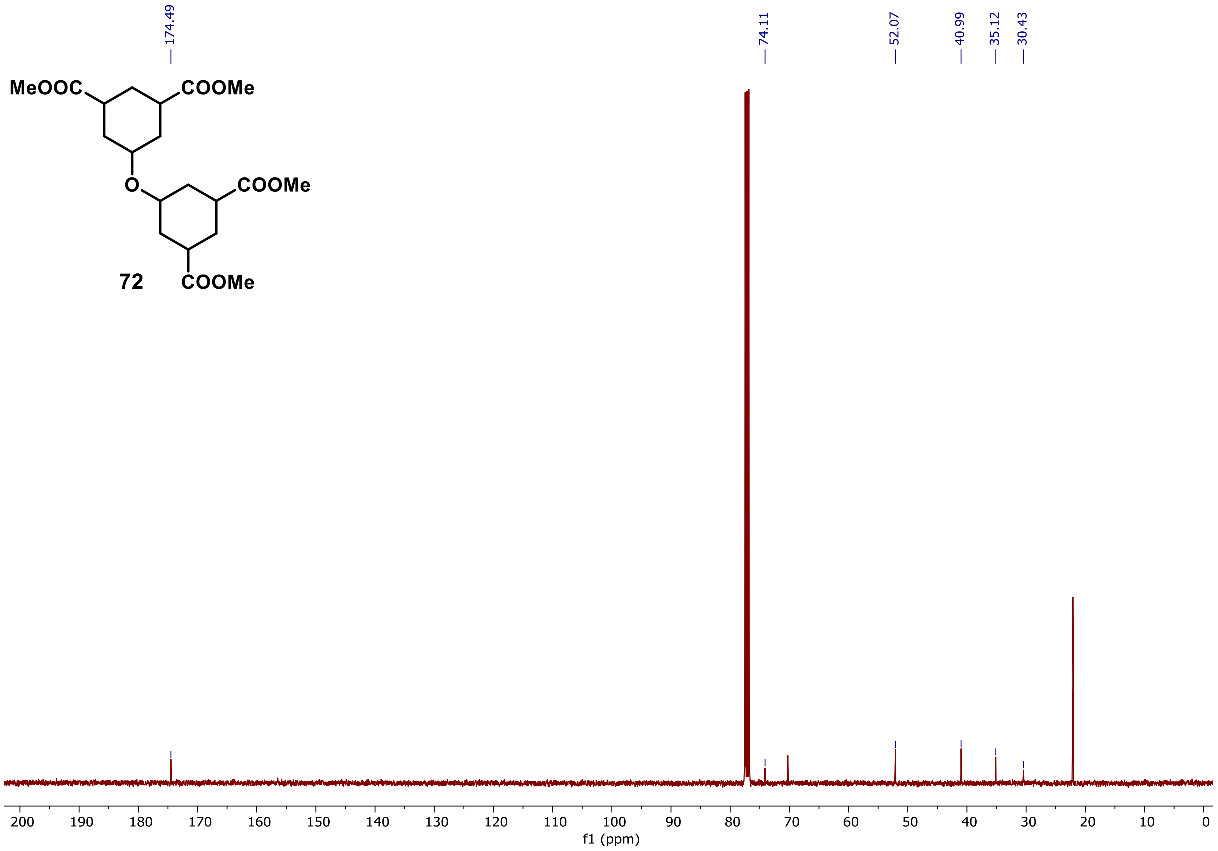

Figure S167: *^{13}C NMR spectrum of tetraester* ***72*** *($CDCl_3$, 101 MHz).*

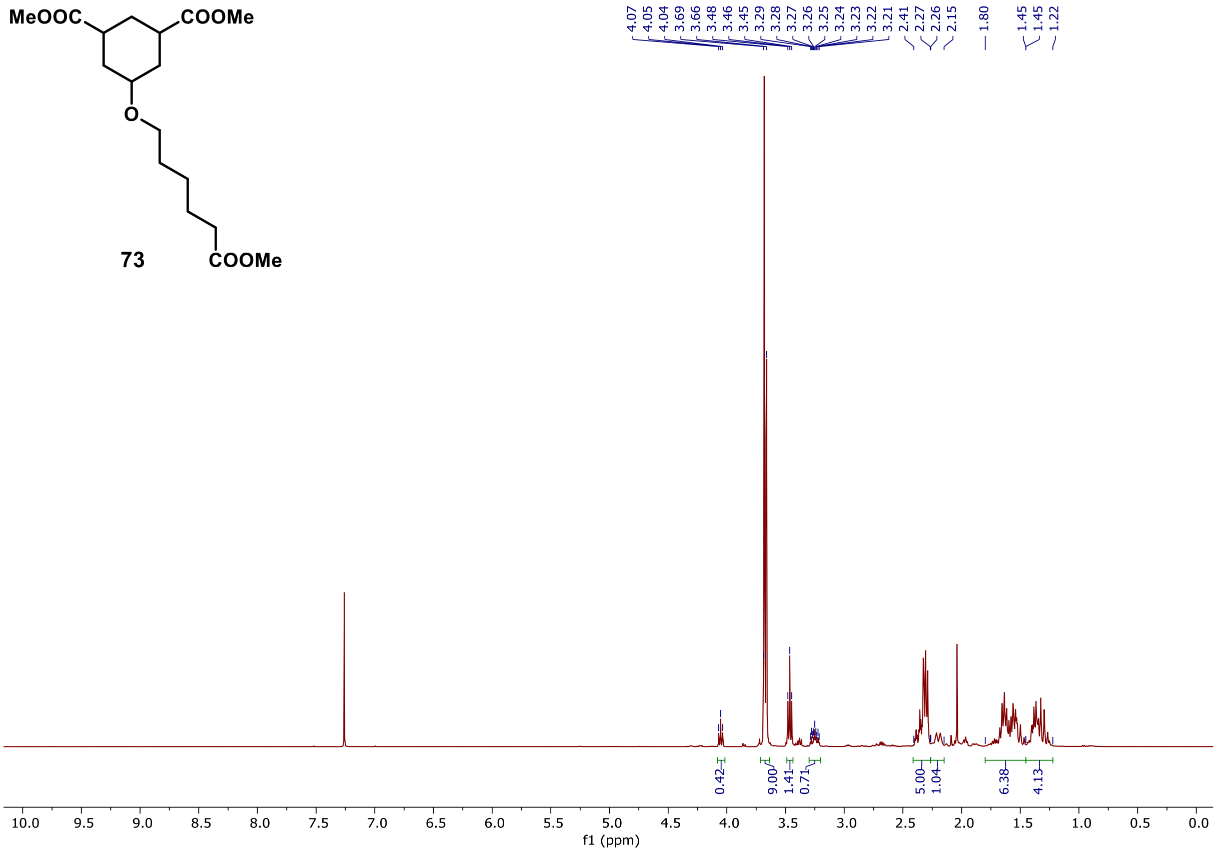

Figure S168: *^{1}H NMR spectra of triester **73** (400 MHz, $CDCl_3$).*

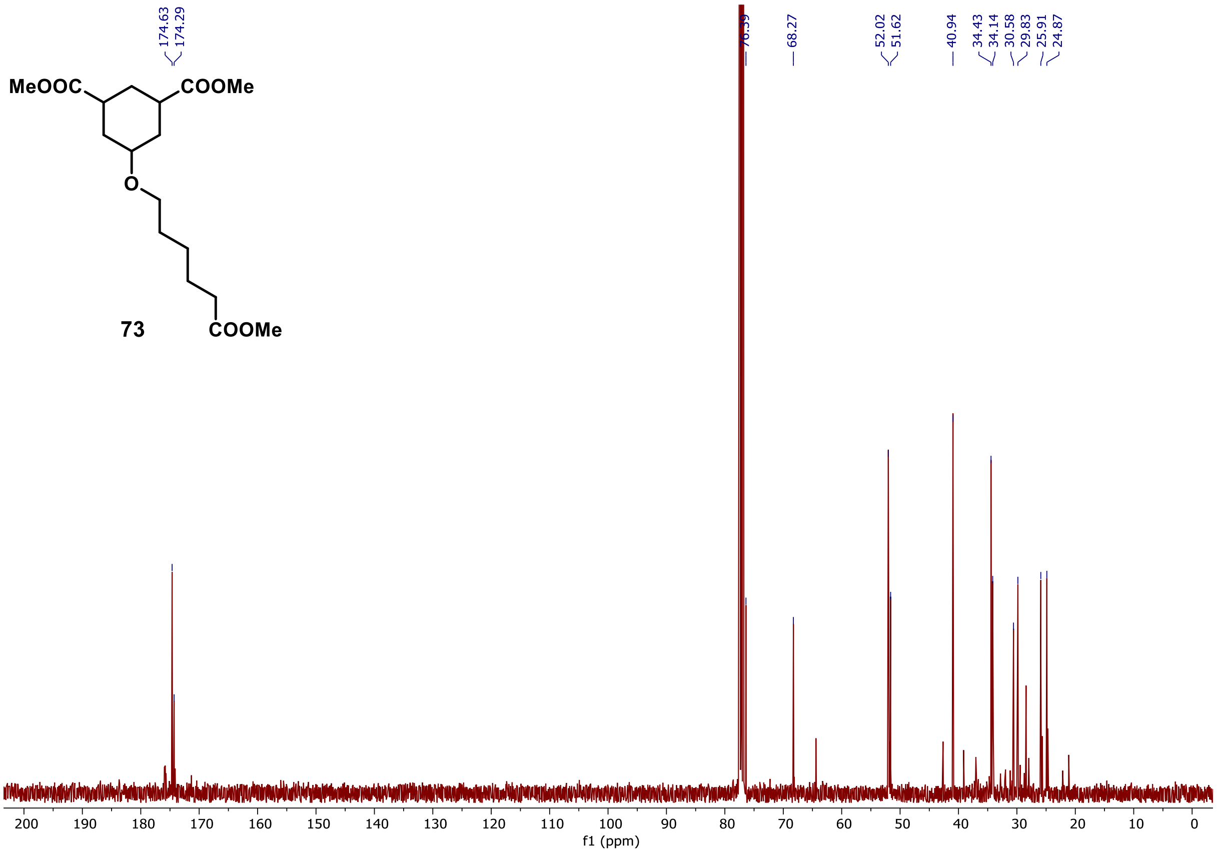

Figure S169: *^{13}C NMR spectrum of triester **73** ($CDCl_3$, 101 MHz).*

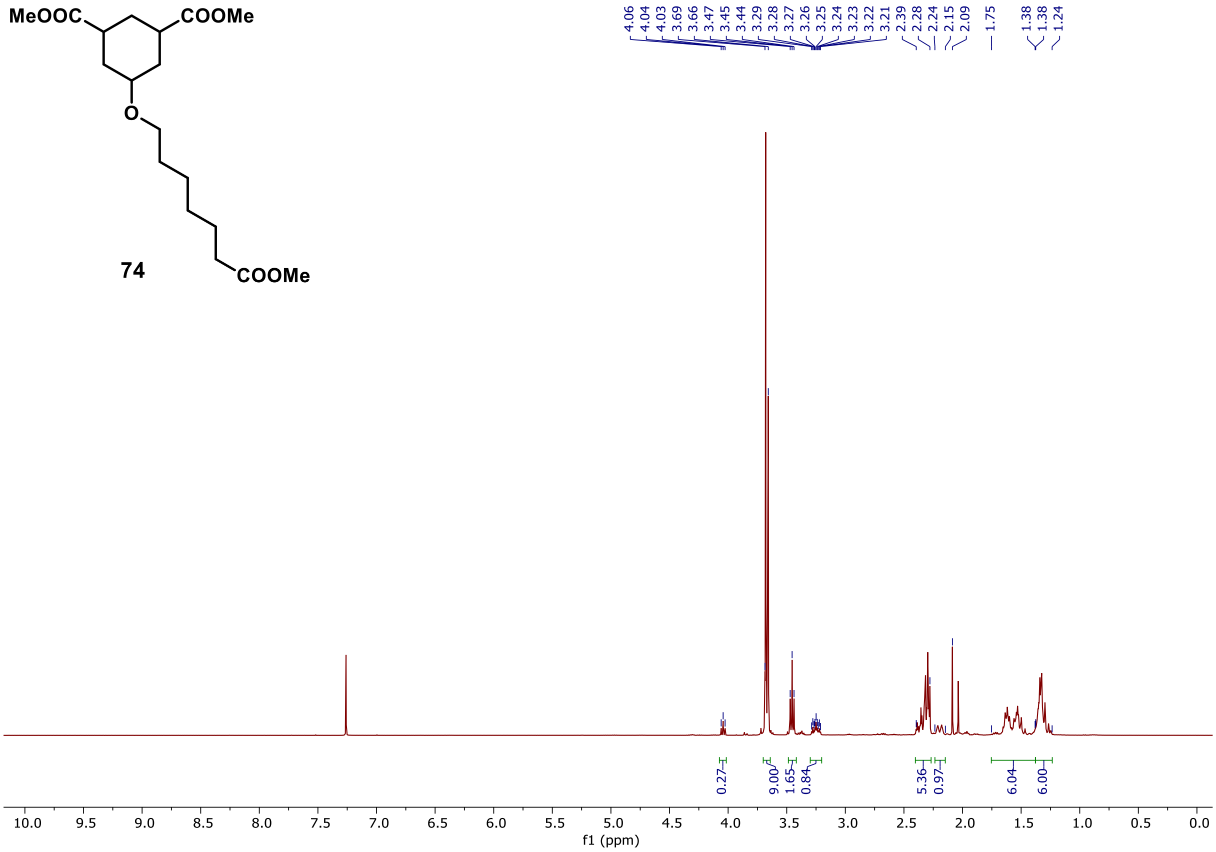

Figure S170: *^{1}H NMR spectra of triester **74** (400 MHz, $CDCl_3$).*

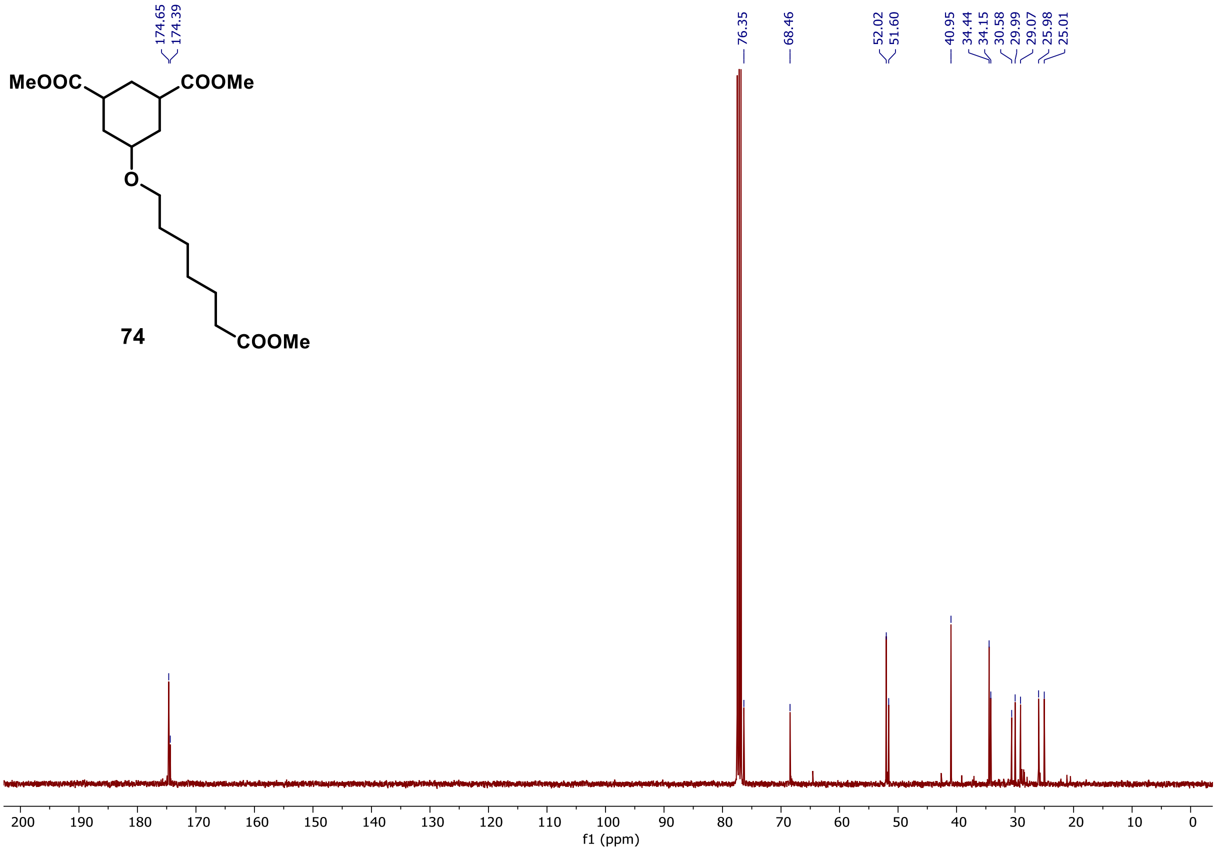

Figure S171: *^{13}C NMR spectrum of triester **74** ($CDCl_3$, 101 MHz)**.***

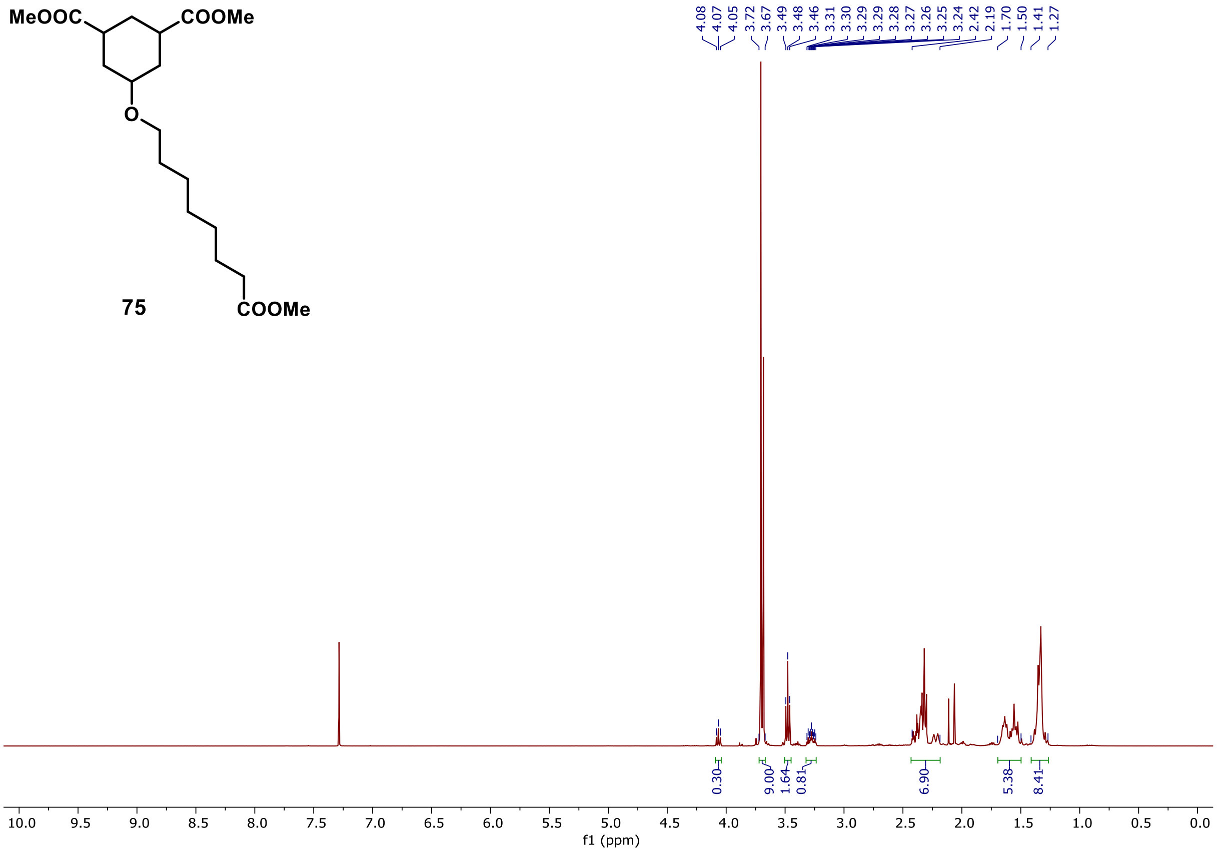

Figure S172: *^{1}H NMR spectra of triester **75** (400 MHz, $CDCl_3$).*

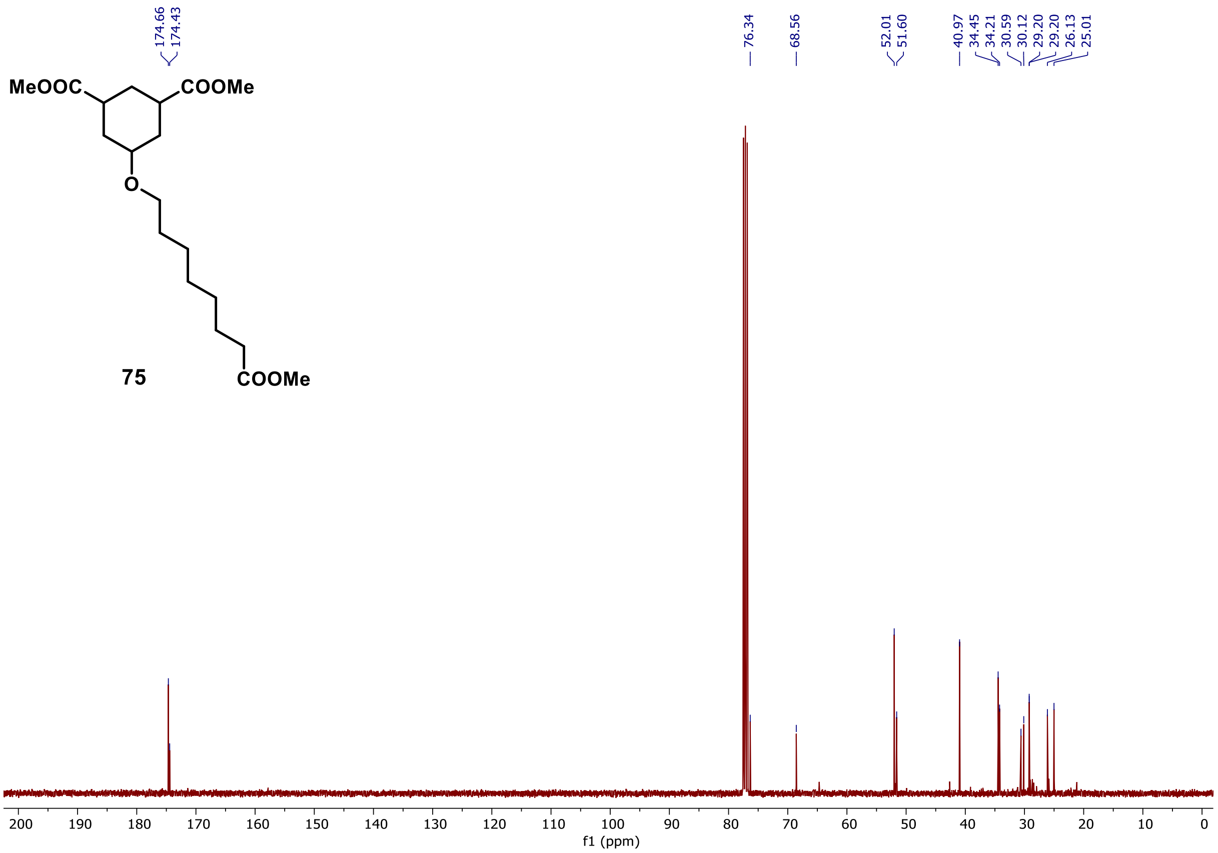

Figure S173: *^{13}C NMR spectrum of triester **75** ($CDCl_3$, 101 MHz)**.***

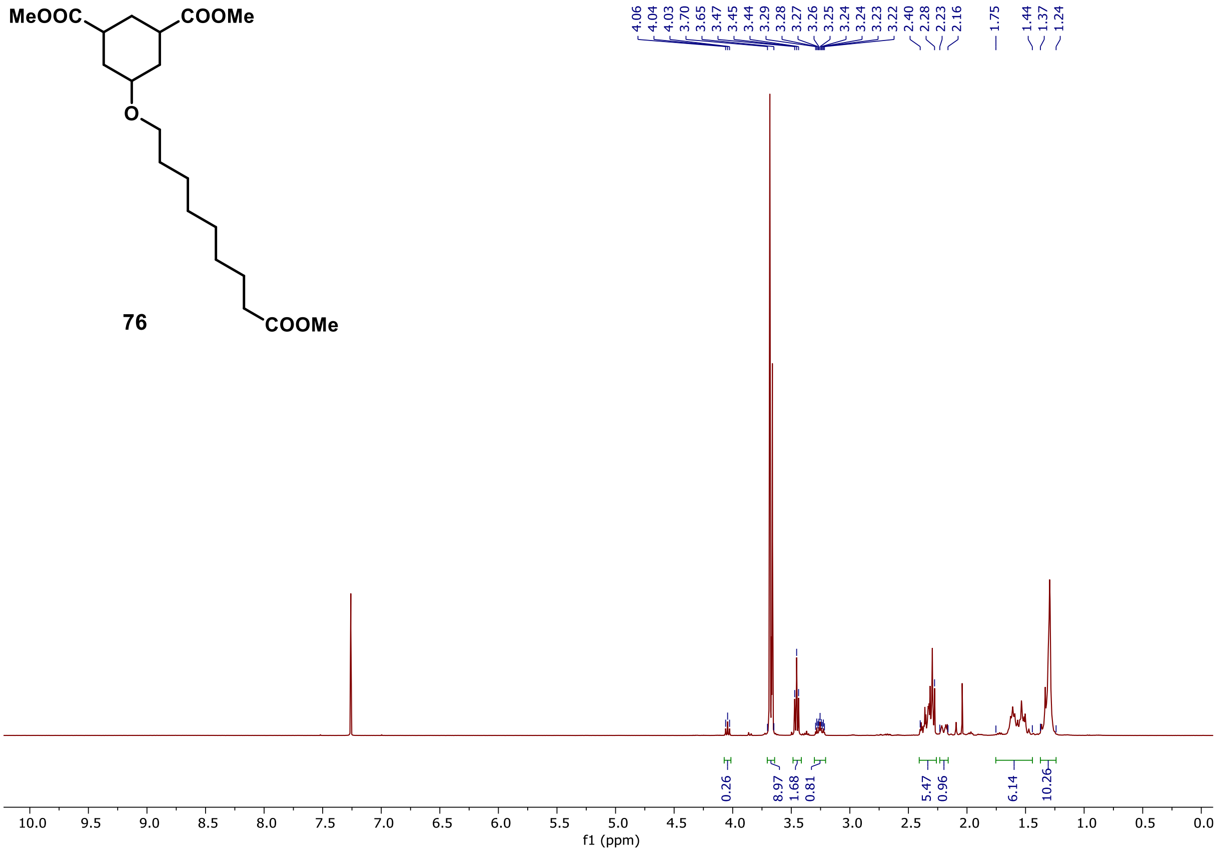

Figure S174: *^{1}H NMR spectra of triester **76** (400 MHz, $CDCl_3$).*

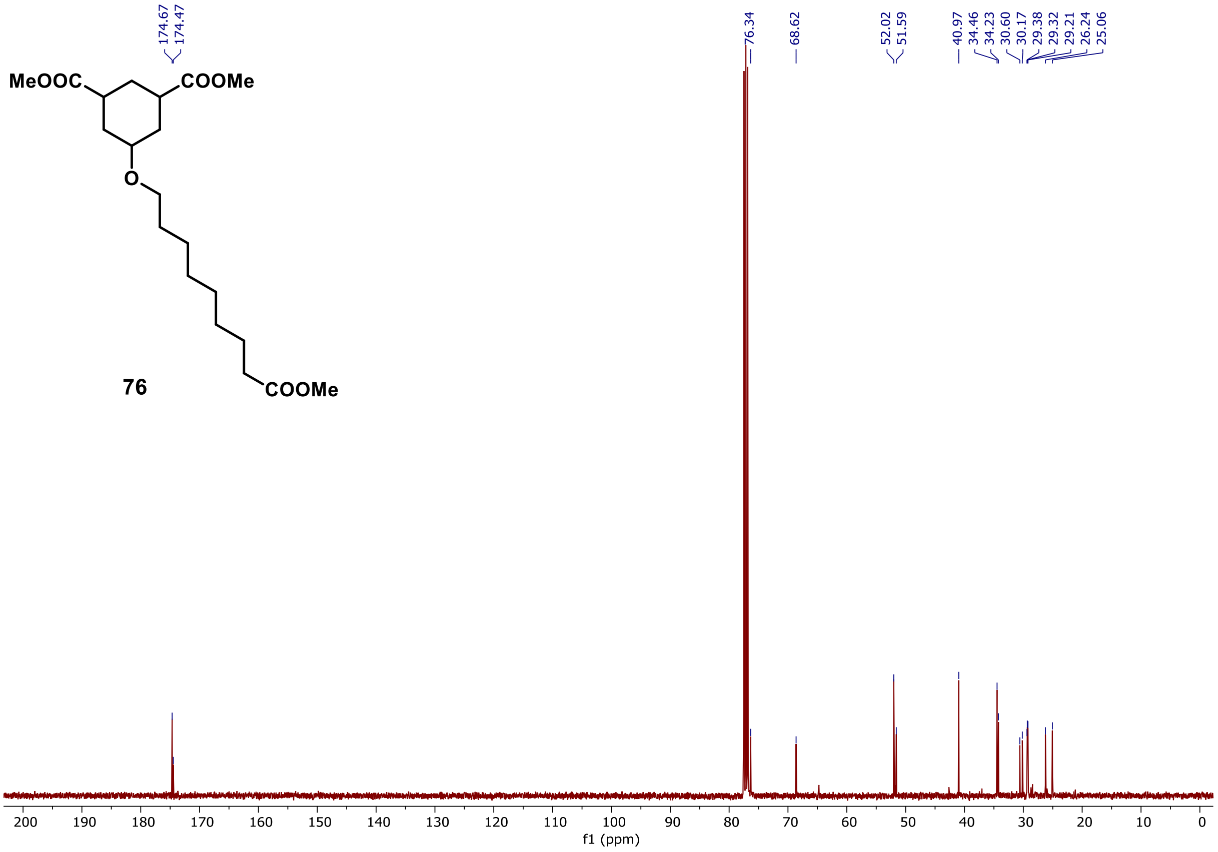

Figure S175: *^{13}C NMR spectrum of triester **76** ($CDCl_3$, 101 MHz).*

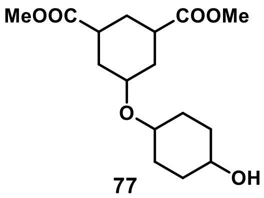

77

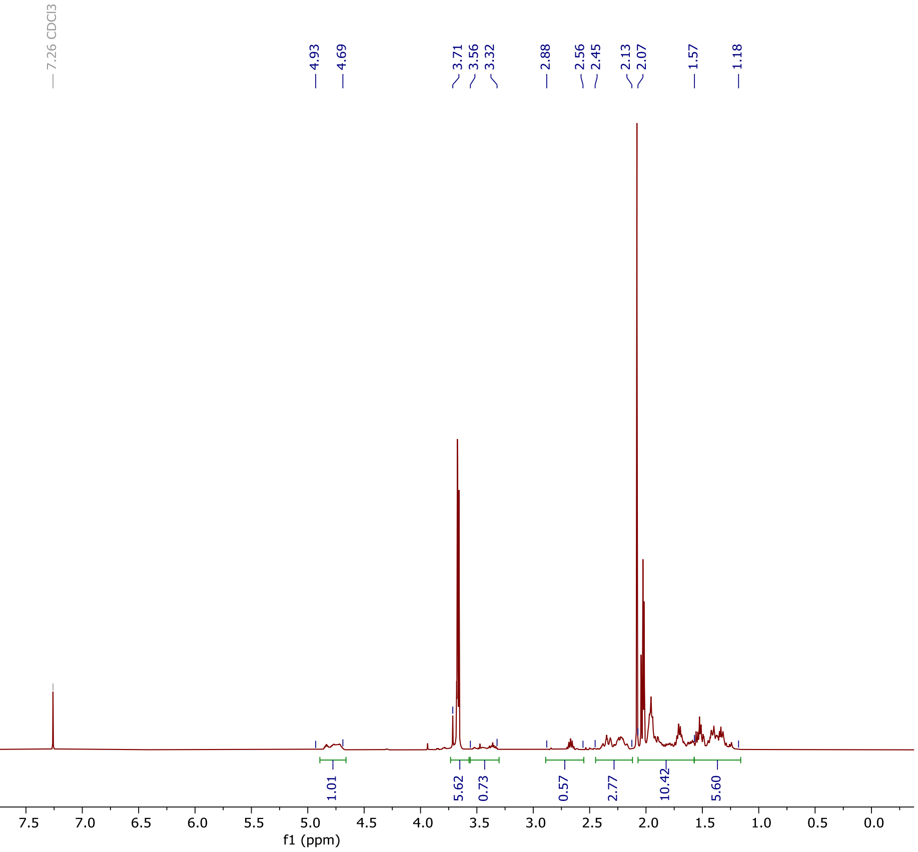

Figure S176: *^{1}H NMR spectra of alcohol* ***77*** *(400 MHz, $CDCl_3$).*

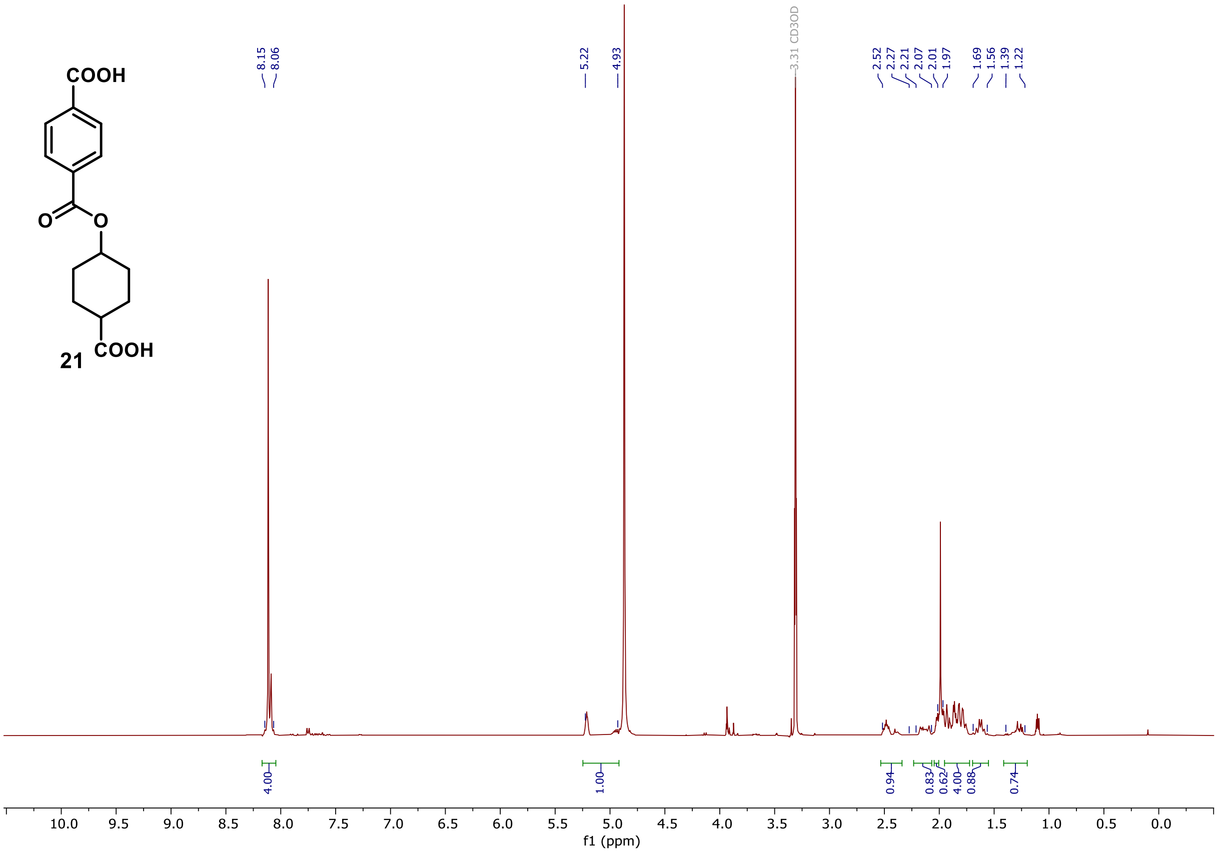

Figure S177: *^{1}H NMR spectra of diacid* ***21*** *(400 MHz, MeOD).*

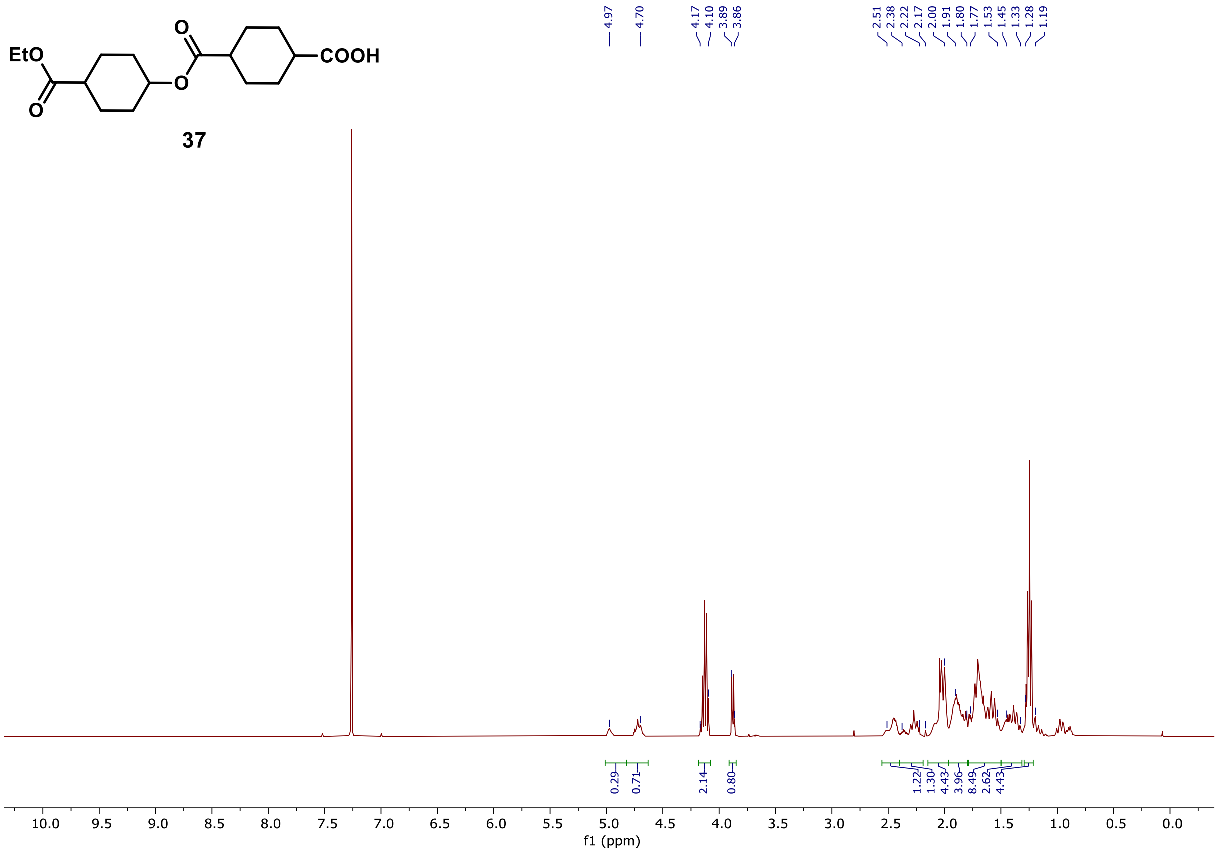

Figure S178: *^{1}H NMR spectra of carboxylic acid* ***37*** *(400 MHz, $CDCl_3$).*

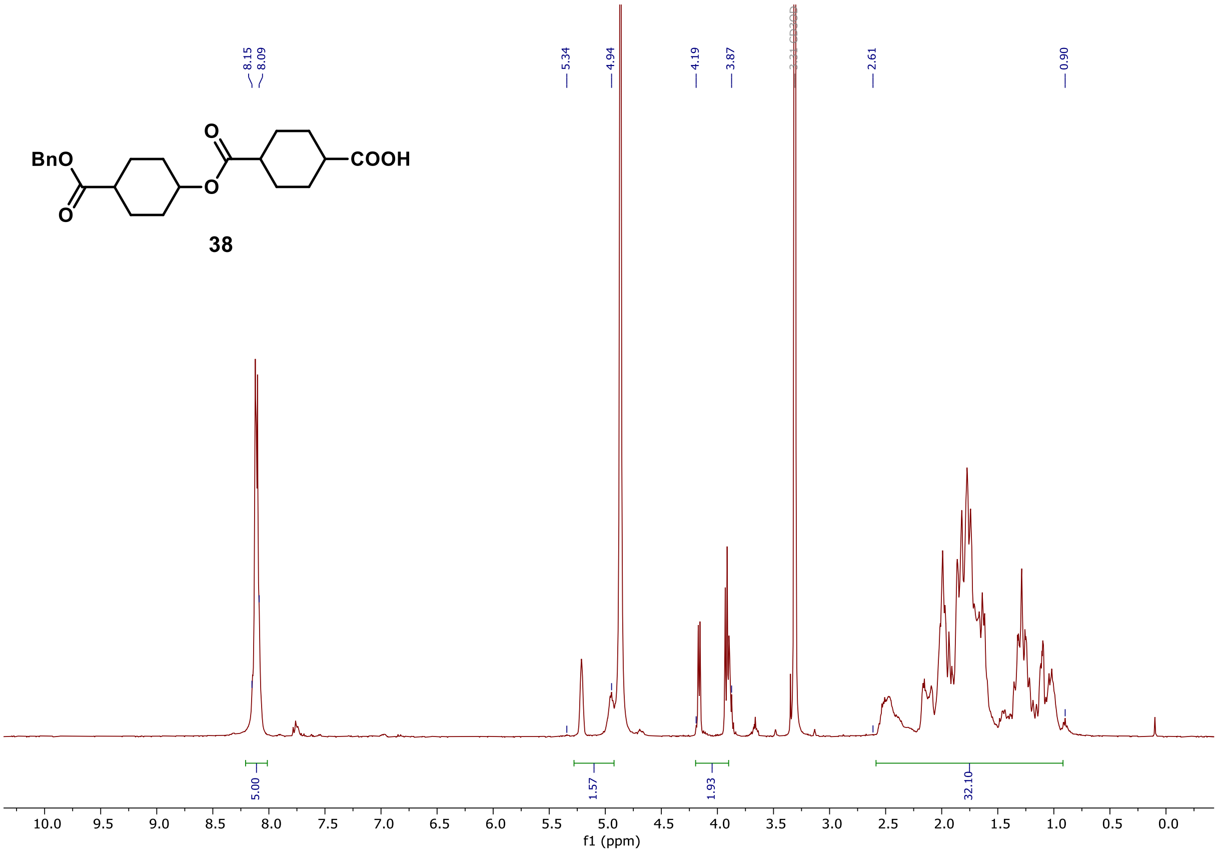

Figure S179: *Crude ^{1}H NMR spectra of carboxylic acid* ***38*** *(400 MHz, MeOD).*

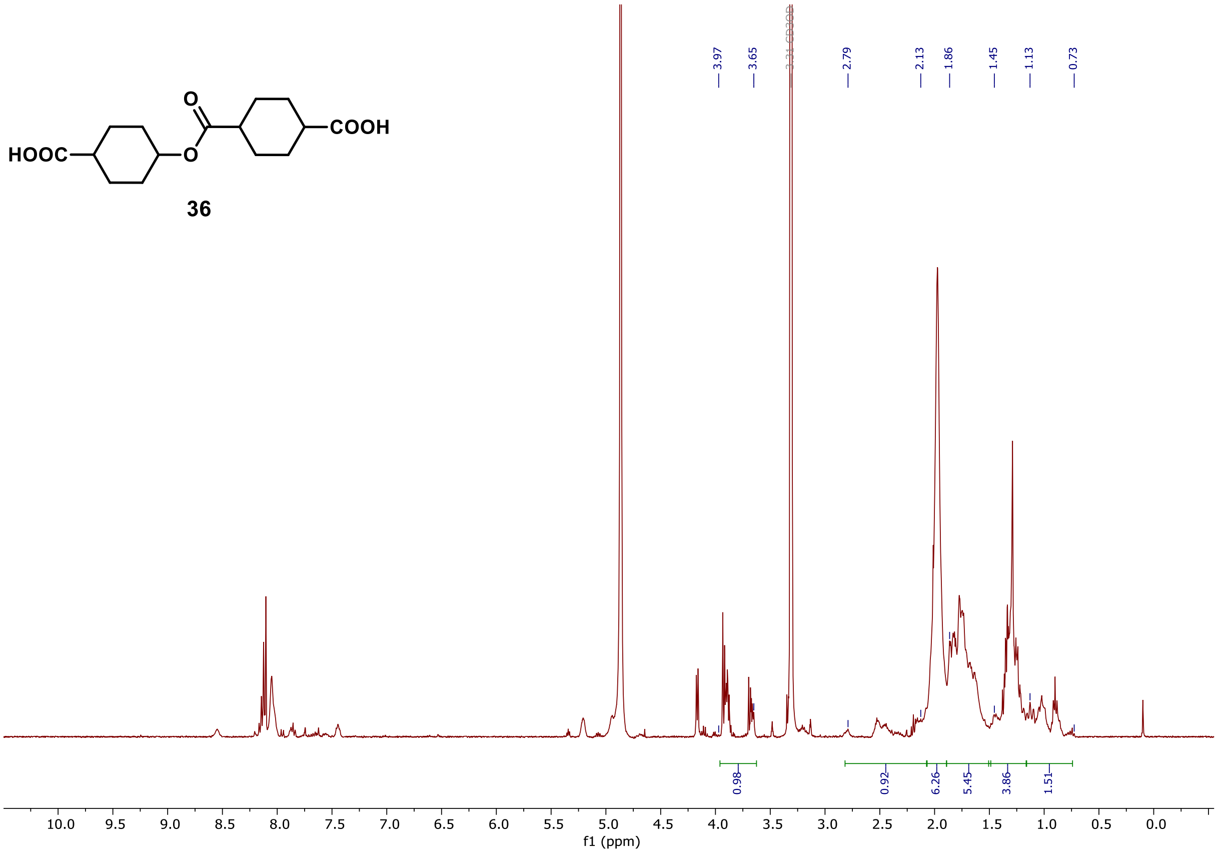

Figure S180: *Crude ^{1}H NMR spectra of dicarboxylic acid* ***36*** *(400 MHz, MeOD).*

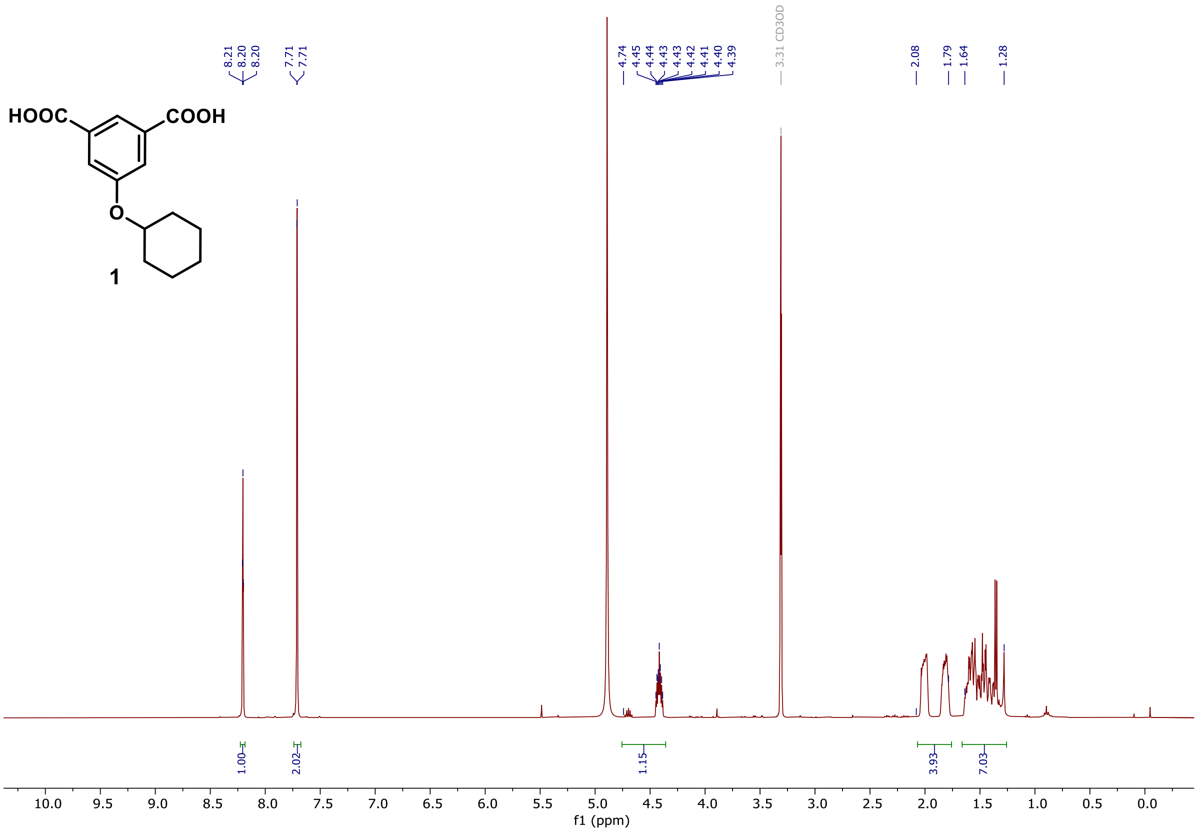

Figure S181: ^{1}H NMR spectra of dicarboxylic acid **1** (400 MHz, MeOD).

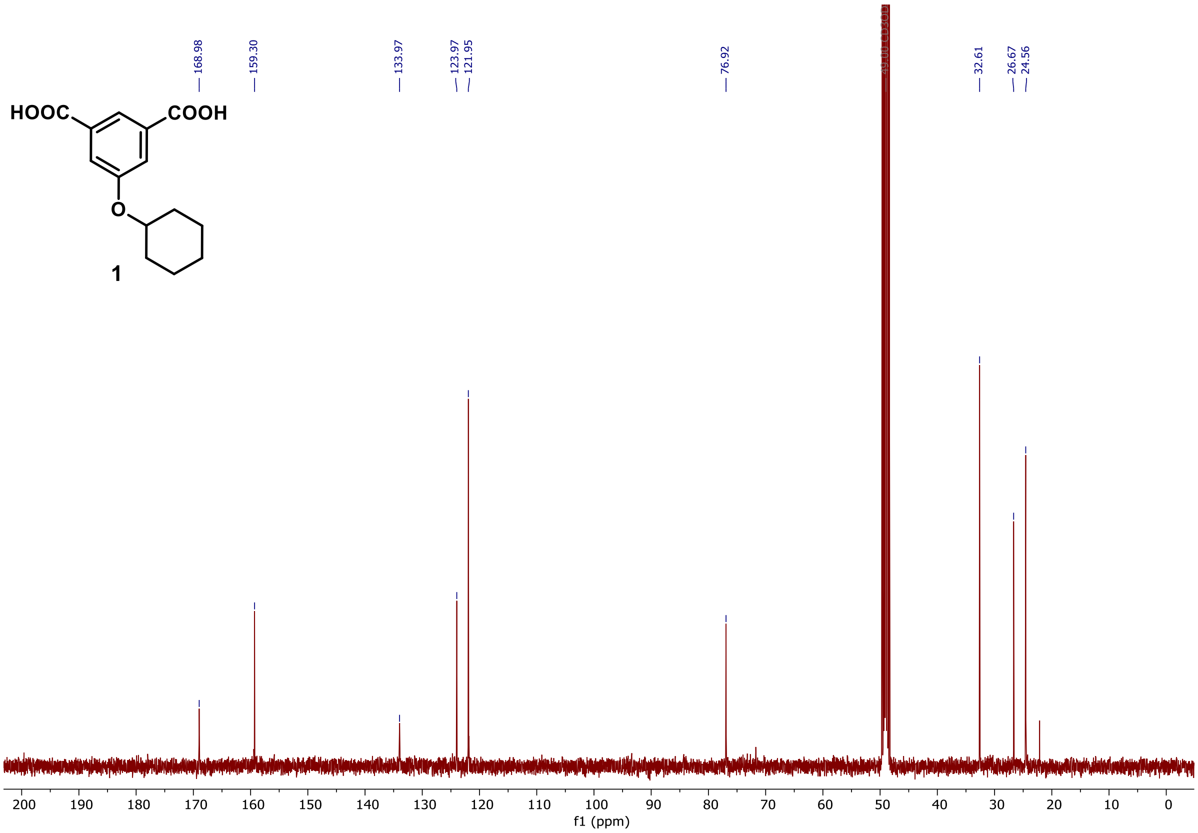

Figure S182: ^{13}C NMR spectrum of dicarboxylic acid **1** (MeOD, 101 MHz).

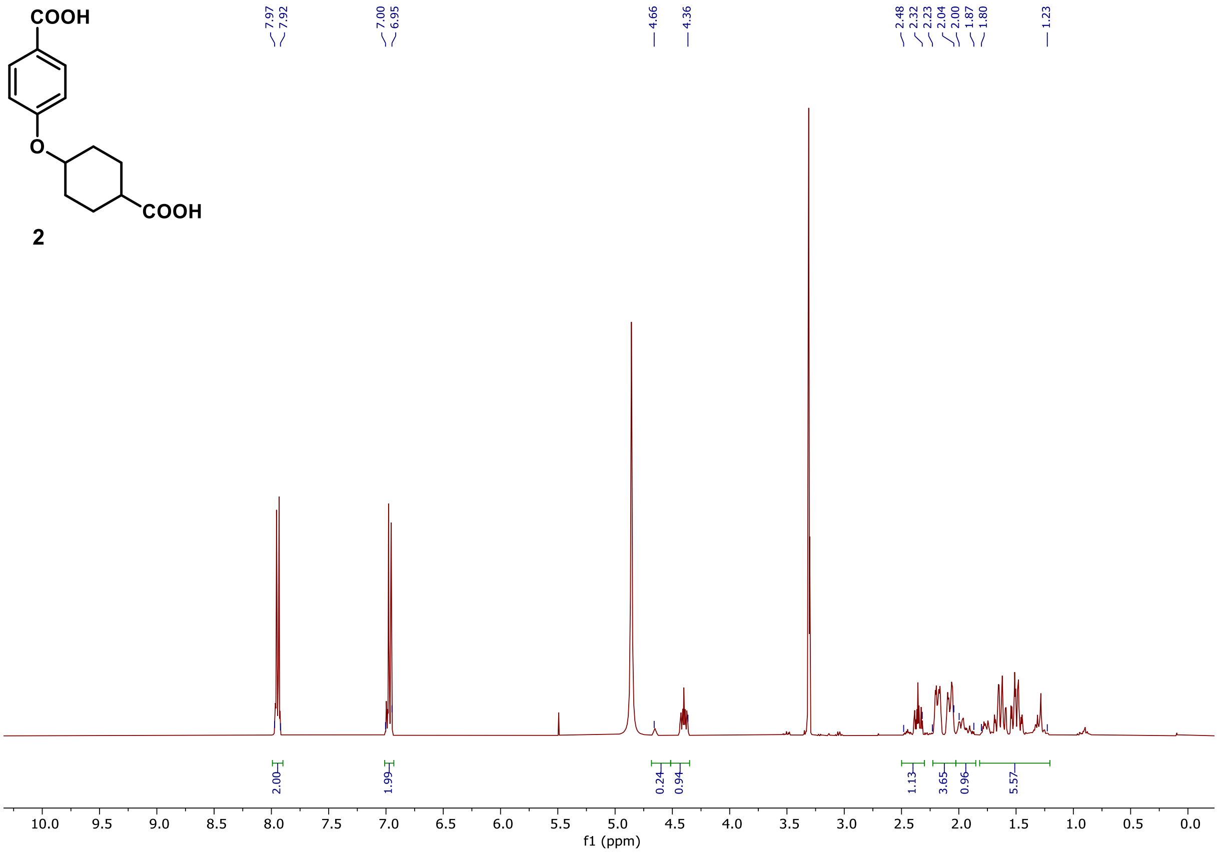

Figure S183: ^{1}*H NMR spectra of dicarboxylic acid* ***2*** *(400 MHz, MeOD).*

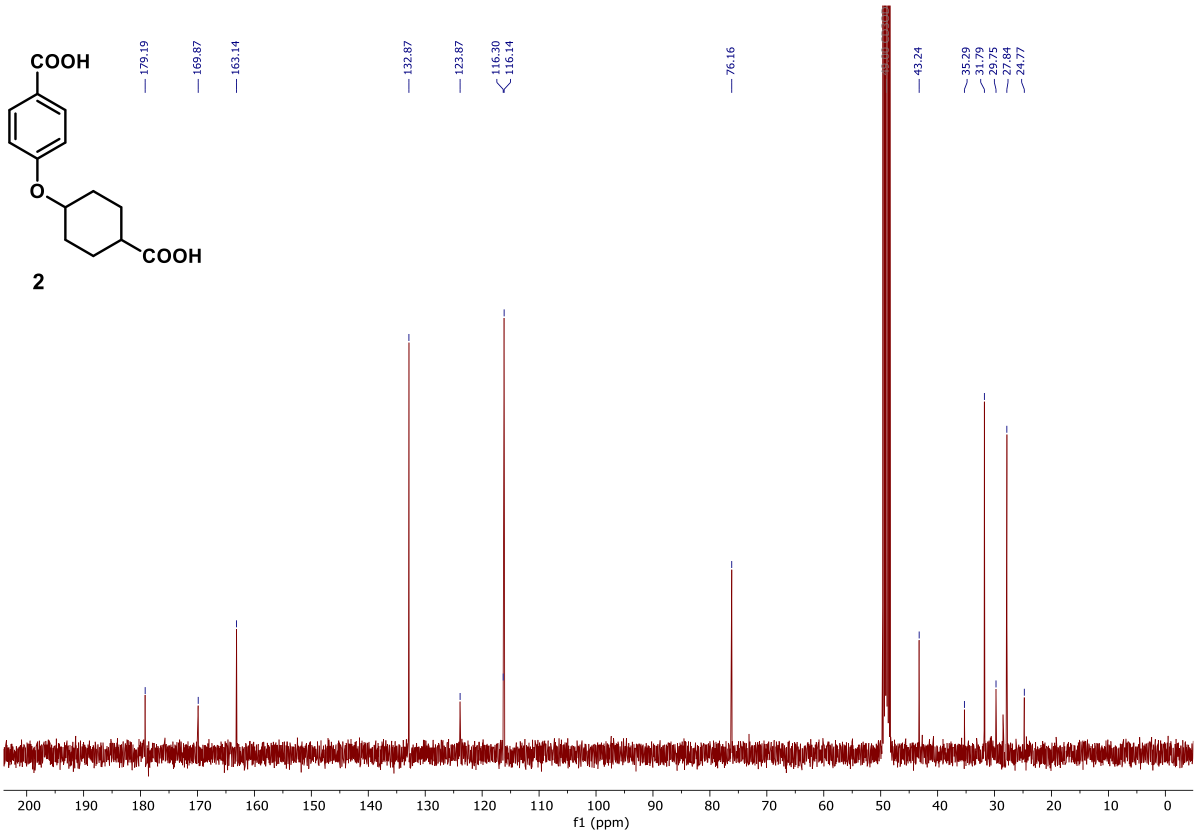

Figure S184: ^{13}*C NMR spectrum of dicarboxylic acid* ***2*** *(MeOD, 101 MHz)****.***

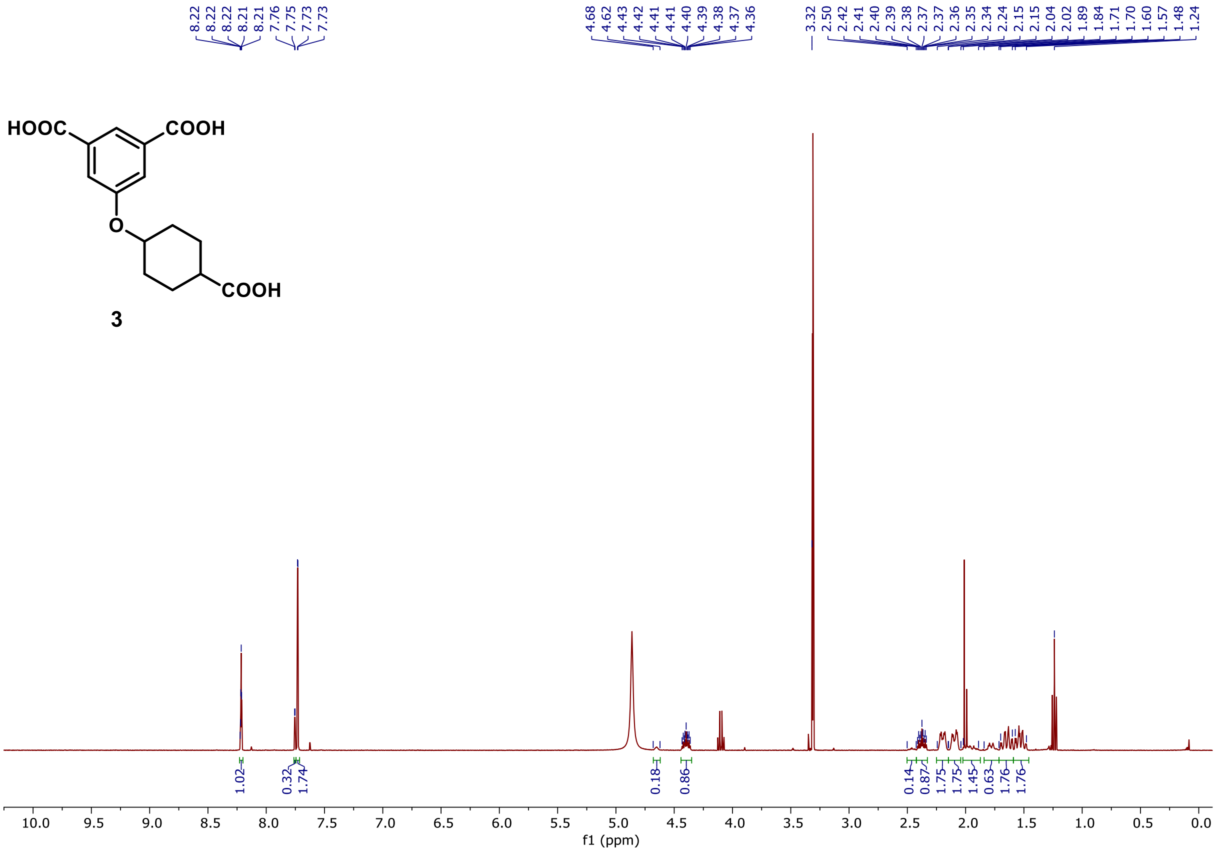

Figure S185: *^{1}H NMR spectra of tricarboxylic acid **3** (400 MHz, MeOD).*

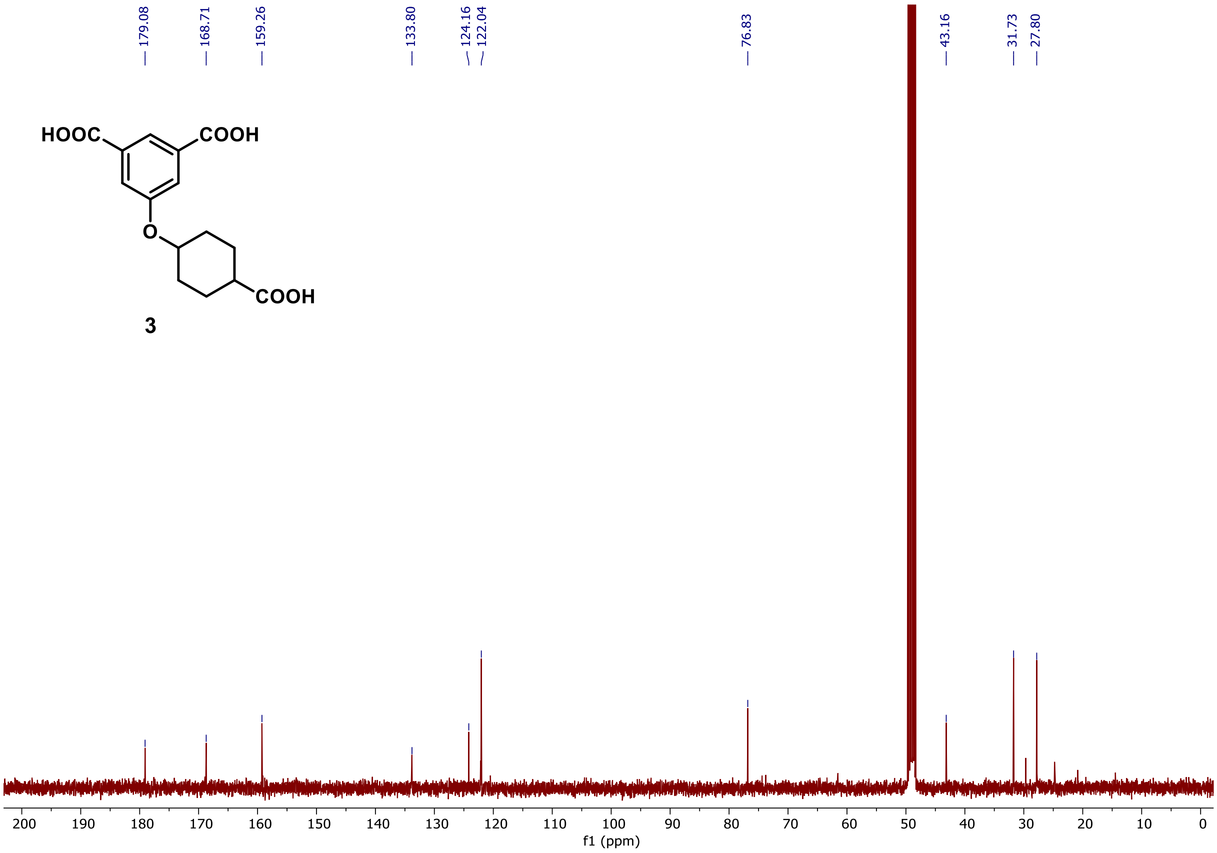

Figure S186: *^{13}C NMR spectrum of tricarboxylic acid **3** (MeOD, 101 MHz).*

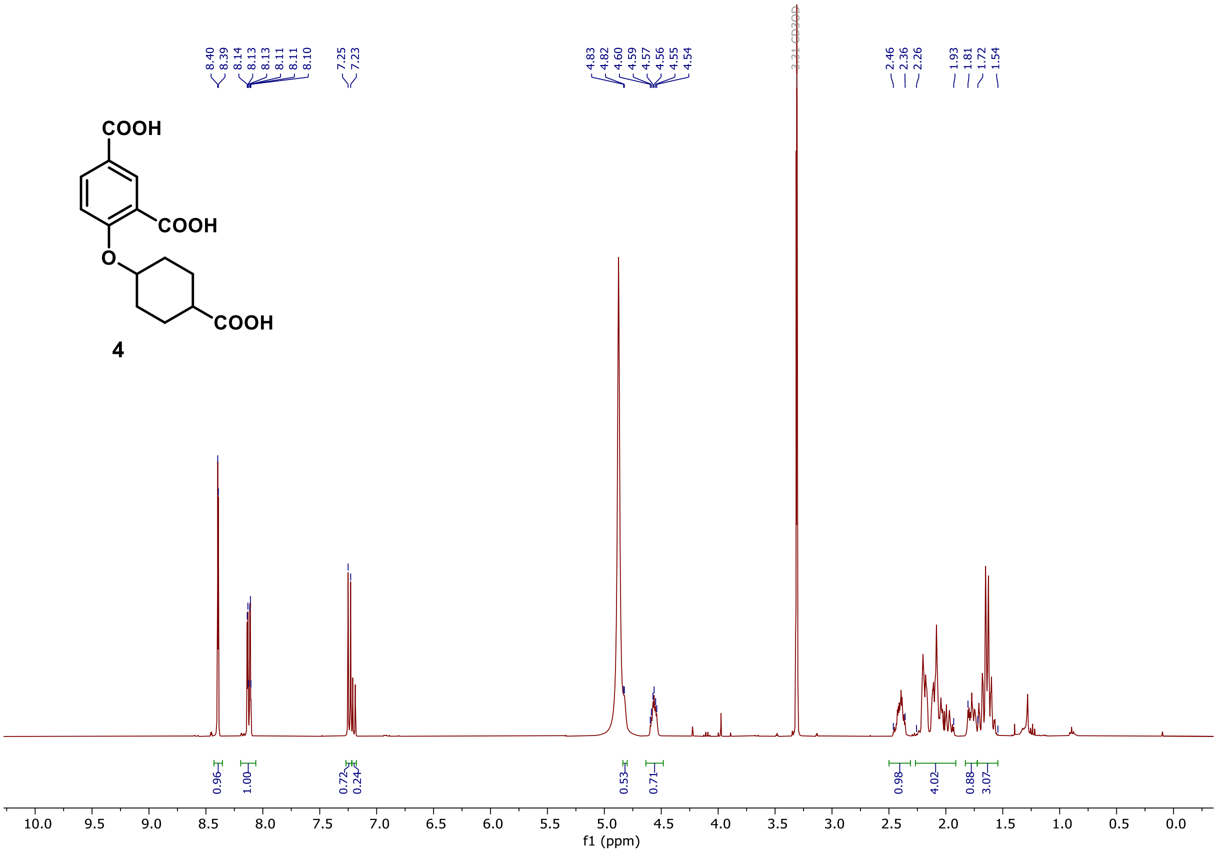

Figure S187: *^{1}H NMR spectra of tricarboxylic acid* ***4*** *(400 MHz, MeOD).*

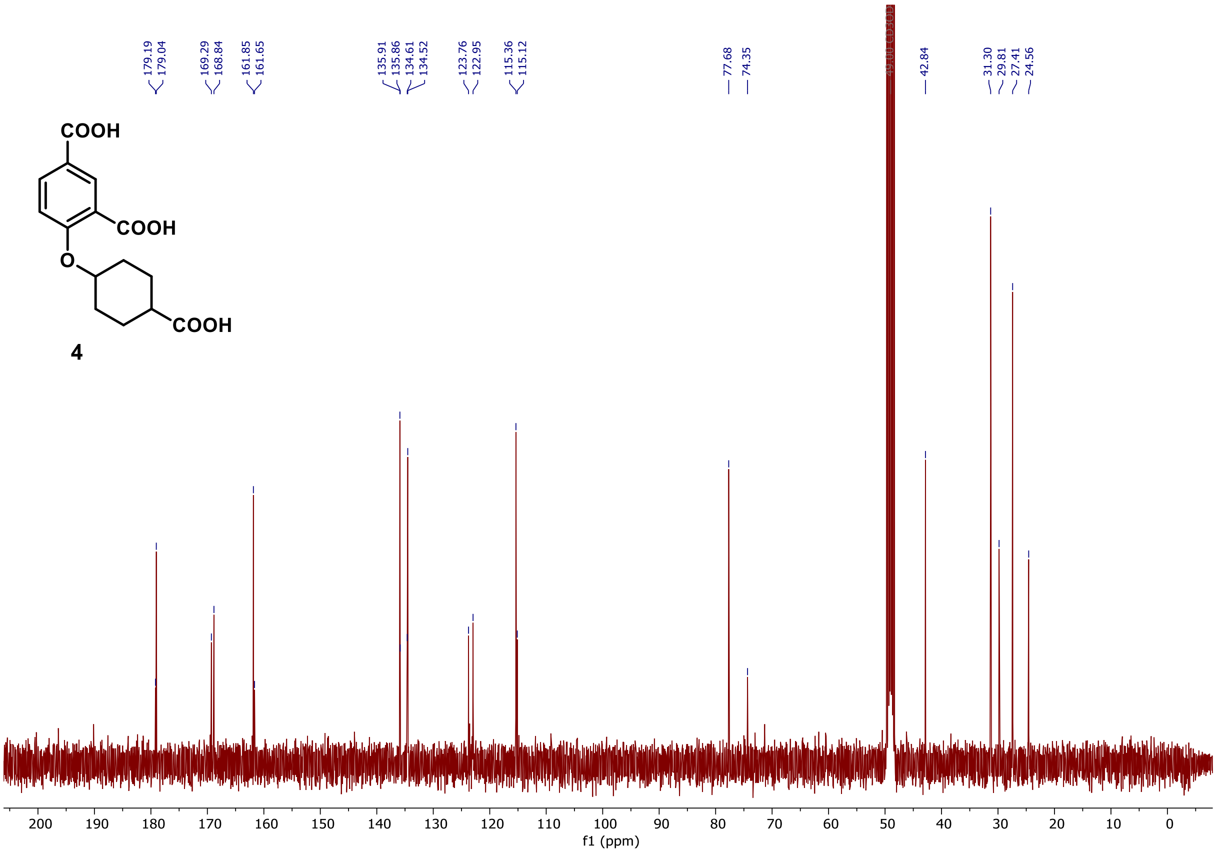

Figure S188: *^{13}C NMR spectrum of tricarboxylic acid* ***4*** ***(MeOD, 101 MHz).***

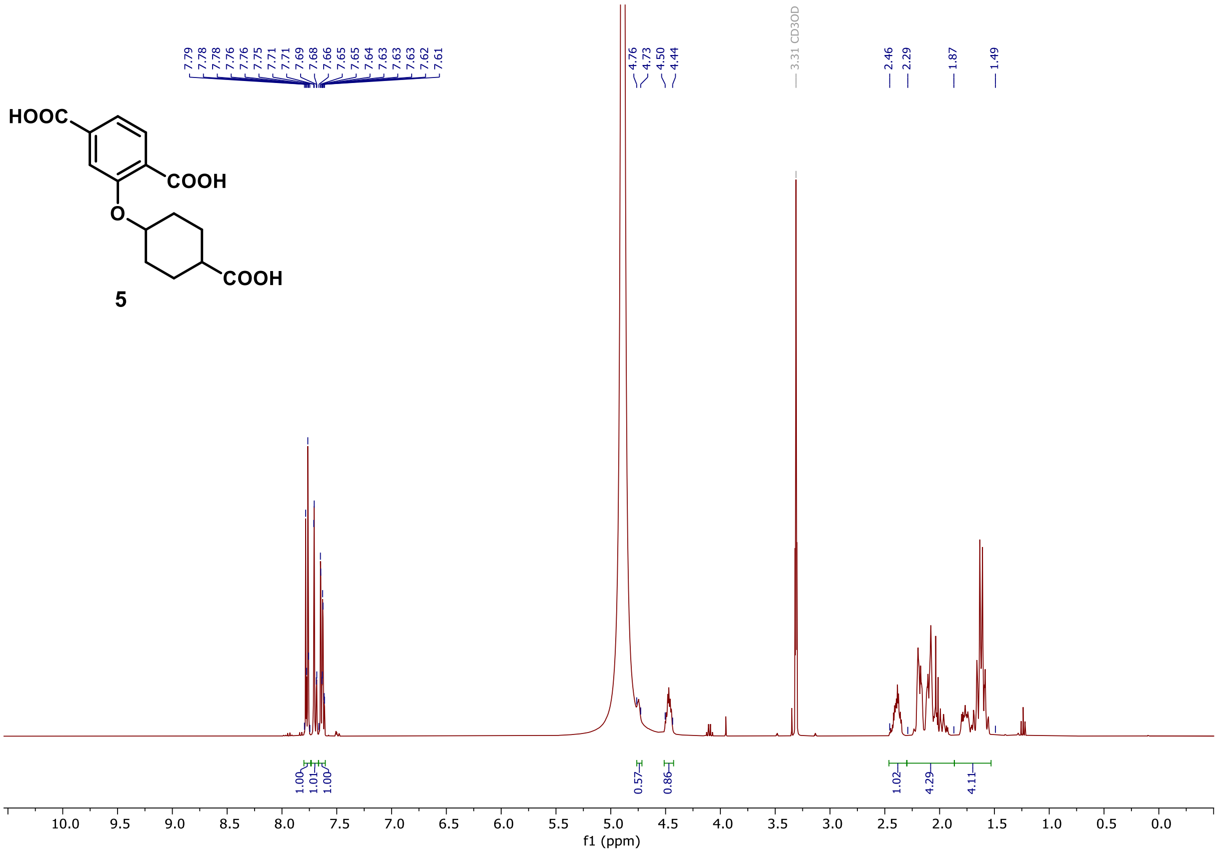

Figure S189: *^{1}H NMR spectra of tricarboxylic acid **5** (400 MHz, MeOD).*

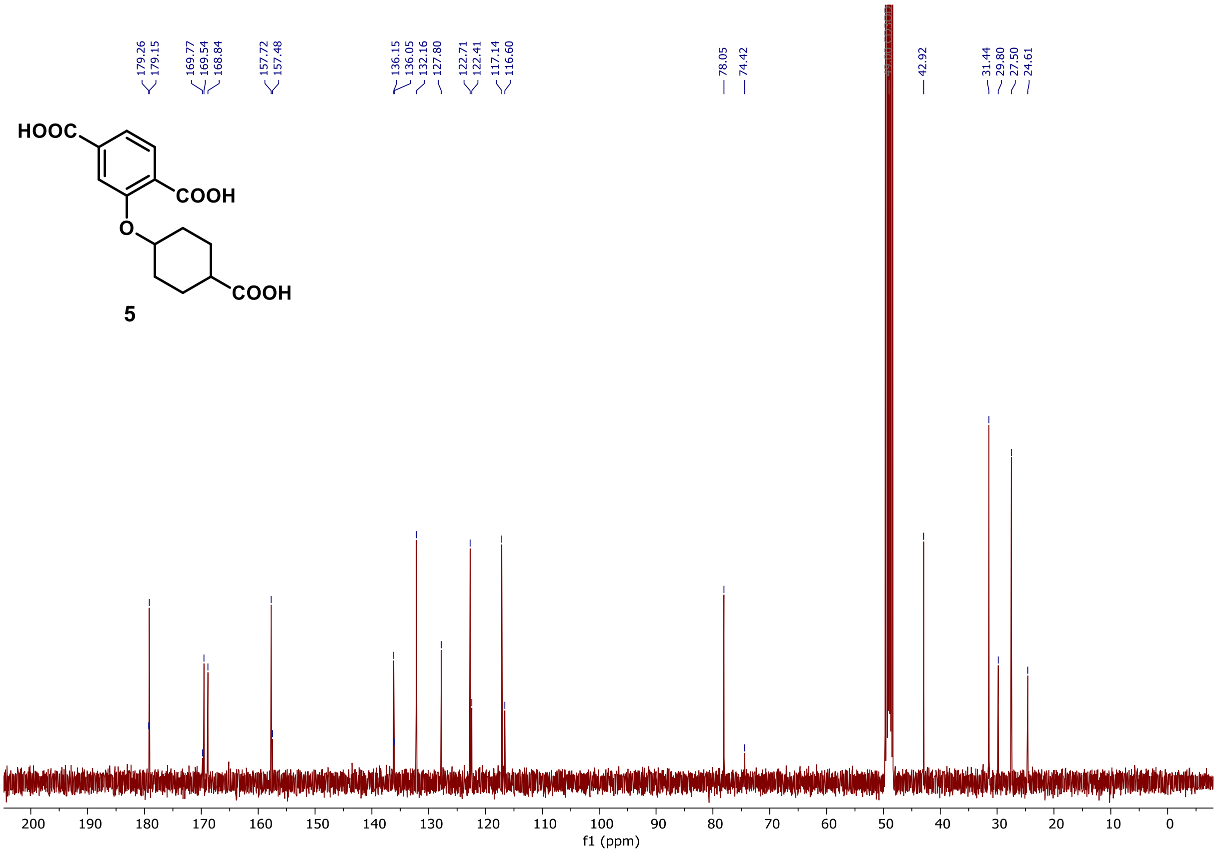

Figure S190: *^{13}C NMR spectrum of tricarboxylic acid **5** (MeOD, 101 MHz).*

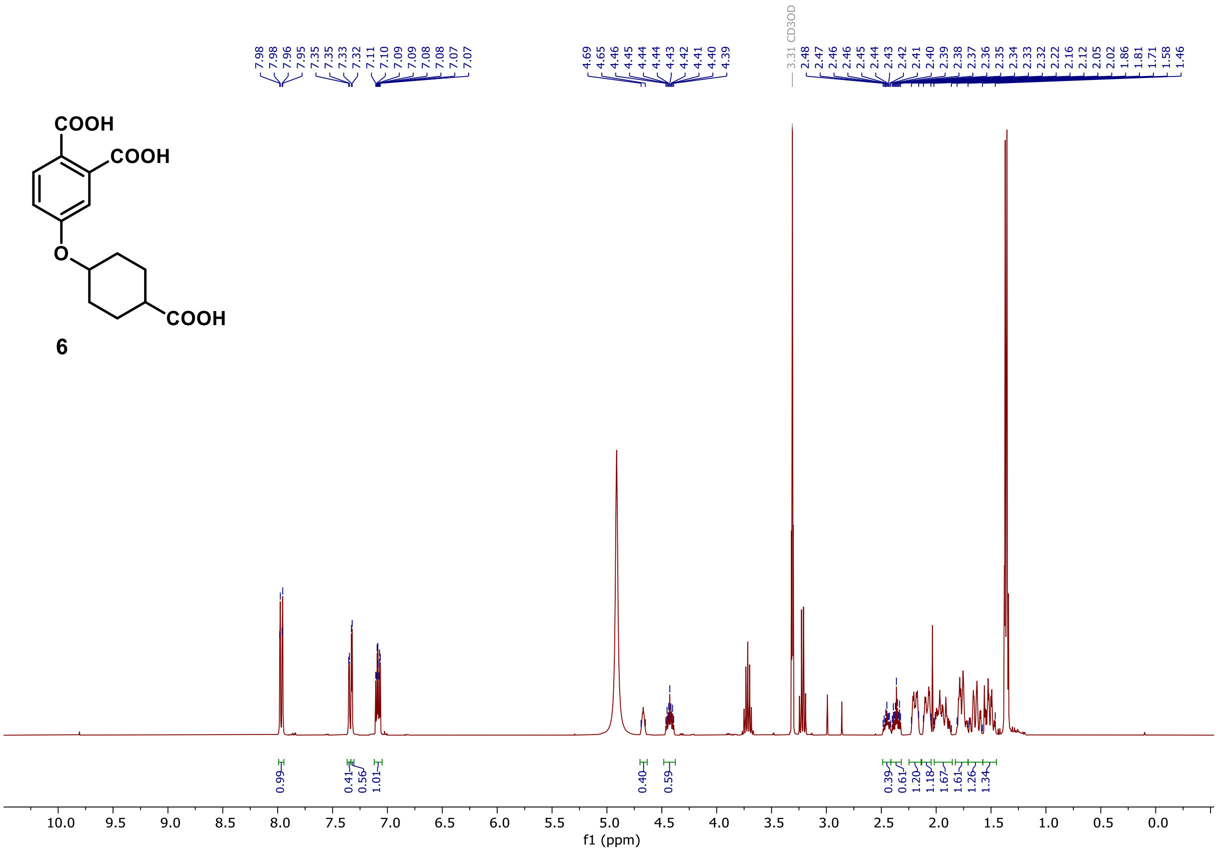

Figure S191: *^{1}H NMR spectra of tricarboxylic acid* ***6*** *(400 MHz, MeOD).*

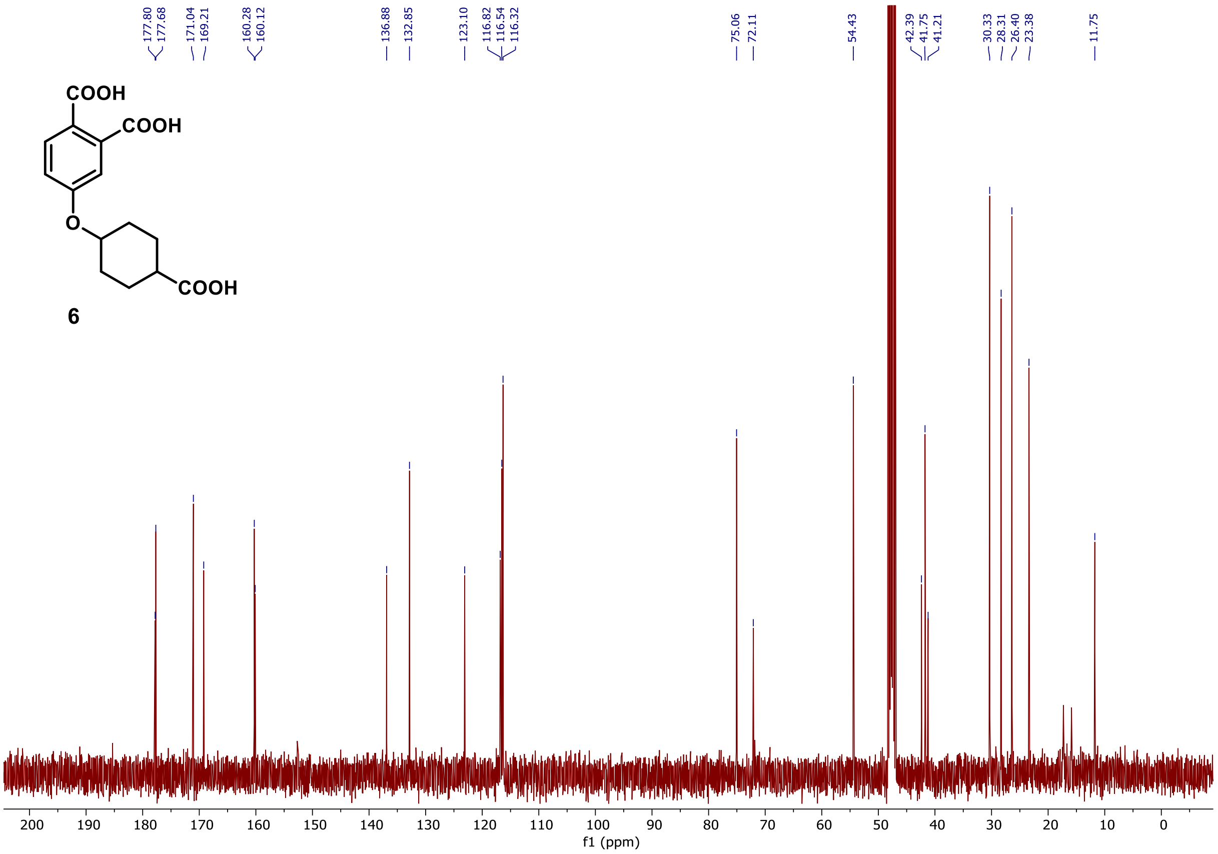

Figure S192: *^{13}C NMR spectrum of tricarboxylic acid* ***6*** *(MeOD, 101 MHz).*

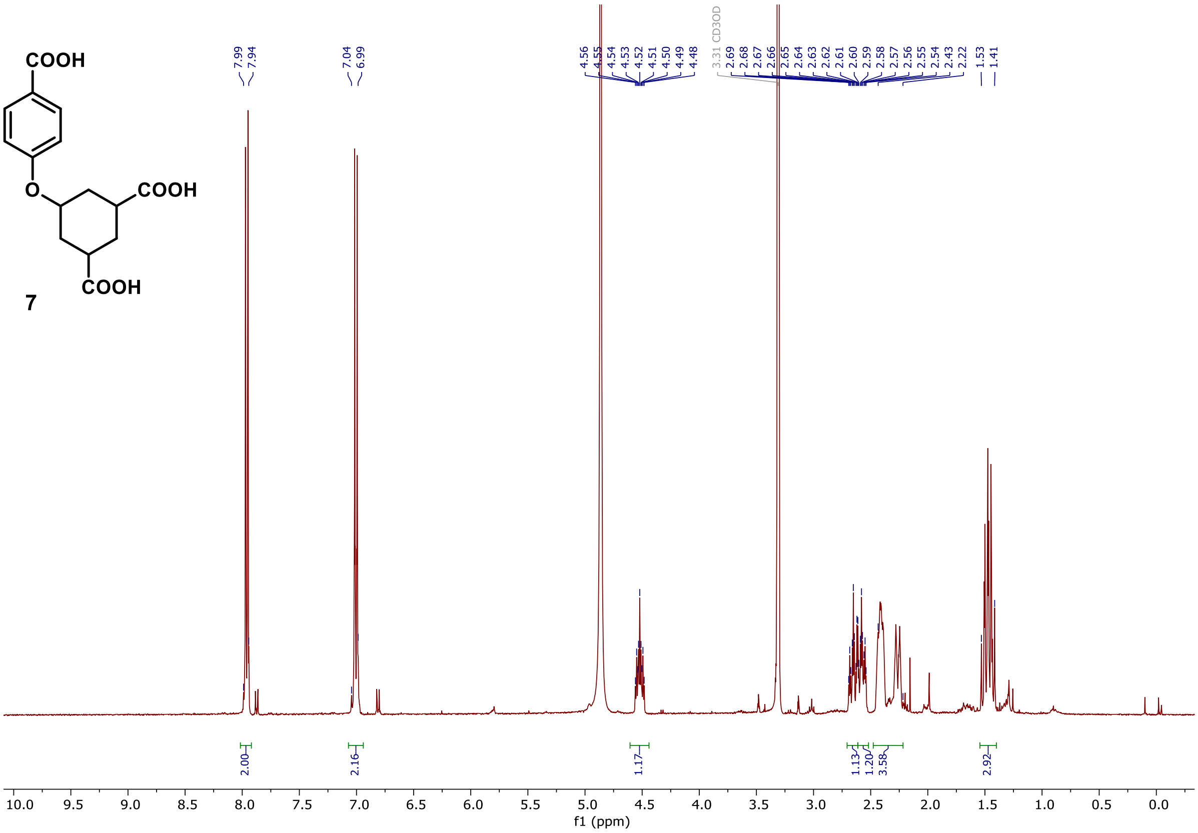

Figure S193: *^{1}H NMR spectra of tricarboxylic acid **7** (400 MHz, MeOD).*

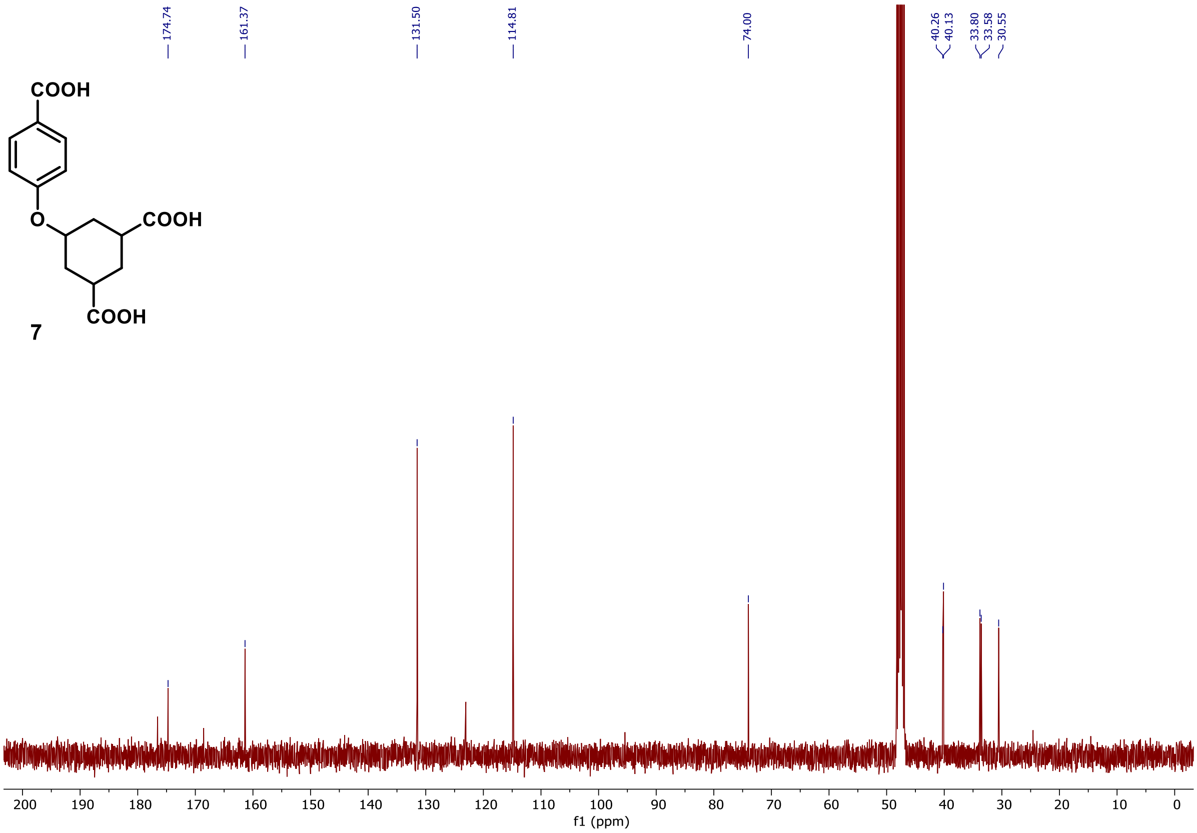

Figure S194: *^{13}C NMR spectrum of tricarboxylic acid **7** (101 MHz, MeOD).*

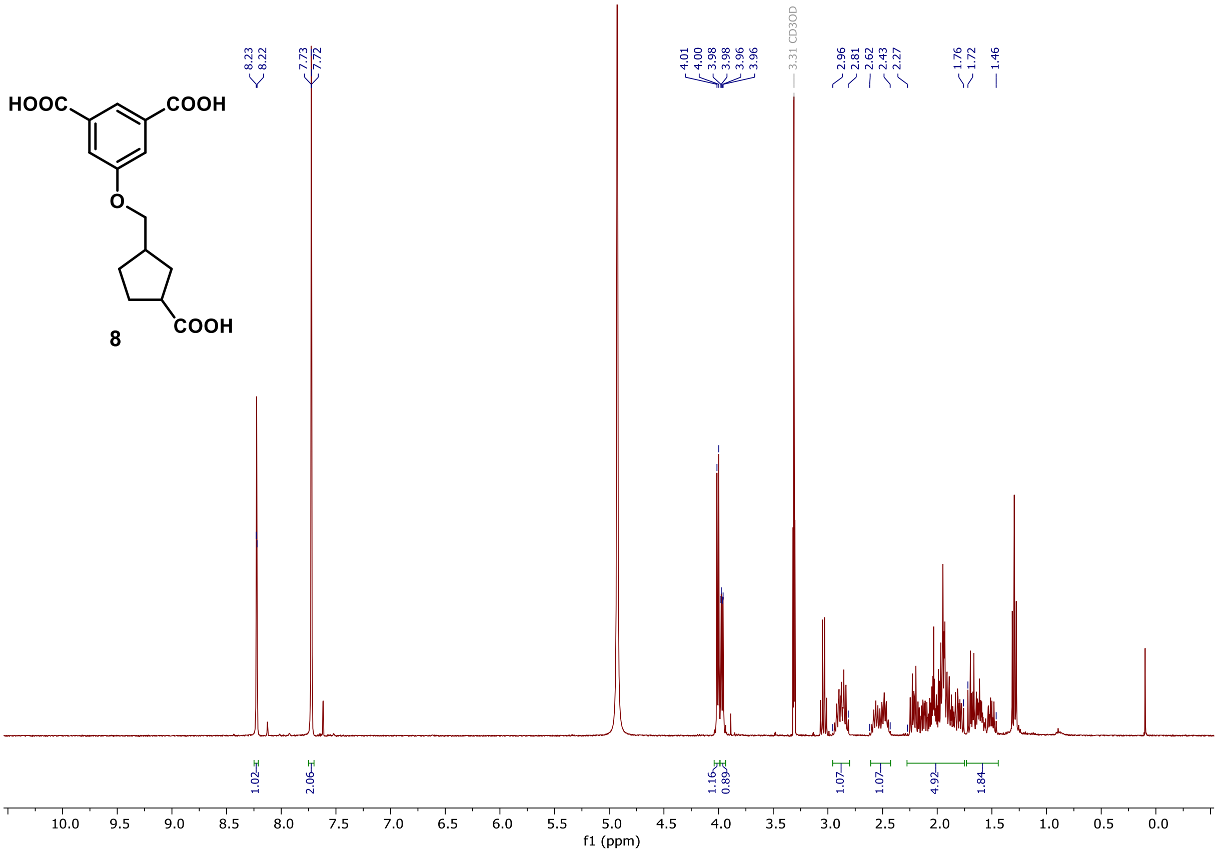

Figure S197: *¹H NMR spectra of tricarboxylic acid* ***8*** *(400 MHz, MeOD).*

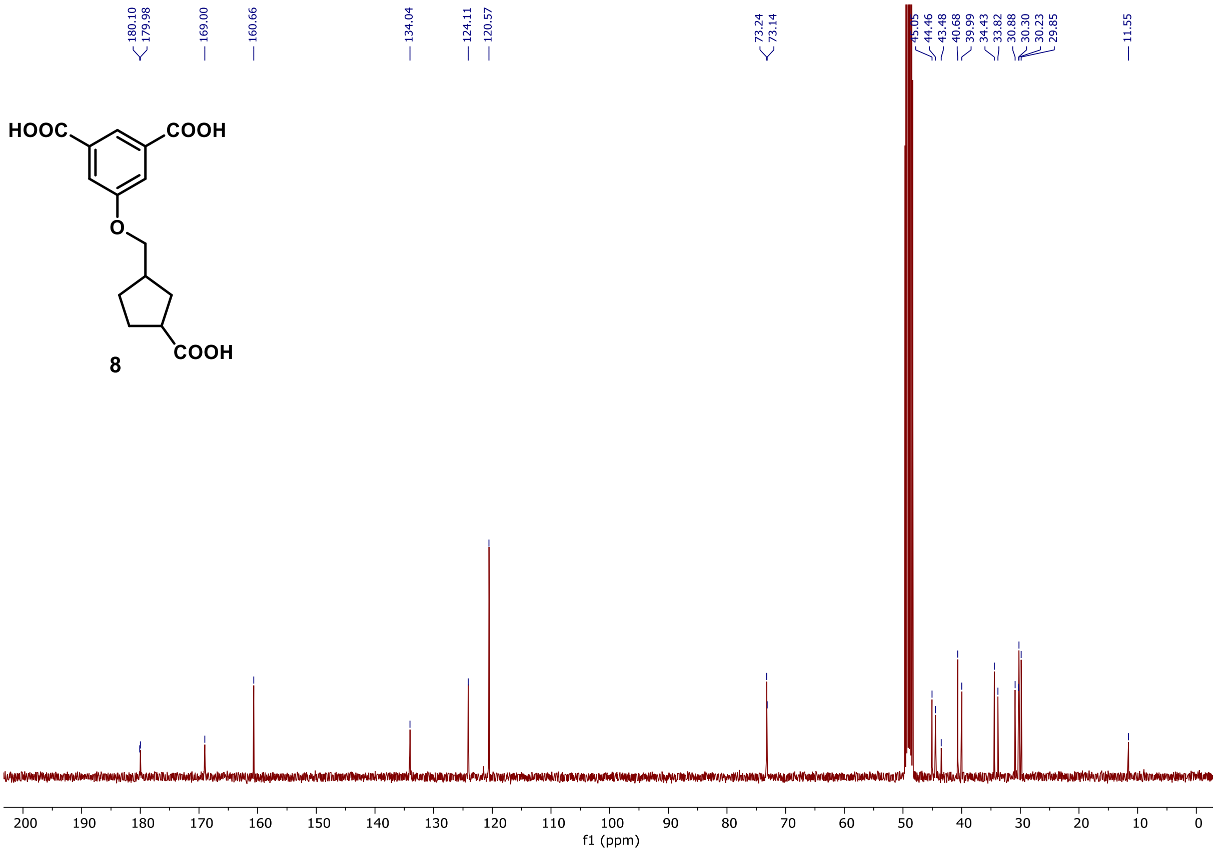

Figure S198: *¹³C NMR spectrum of tricarboxylic acid* ***8*** *(101 MHz, MeOD).*

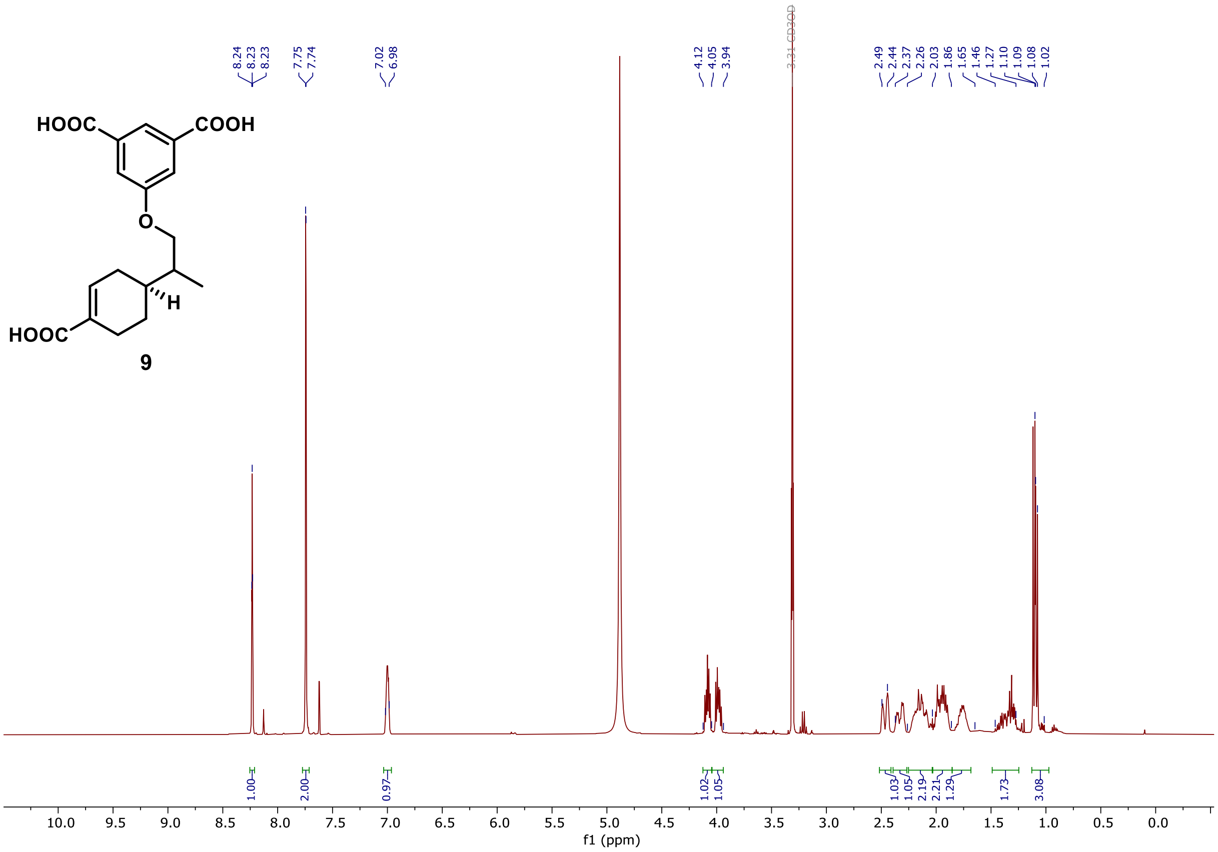

Figure S199: *^{1}H NMR spectra of tricarboxylic acid **9** (400 MHz, MeOD).*

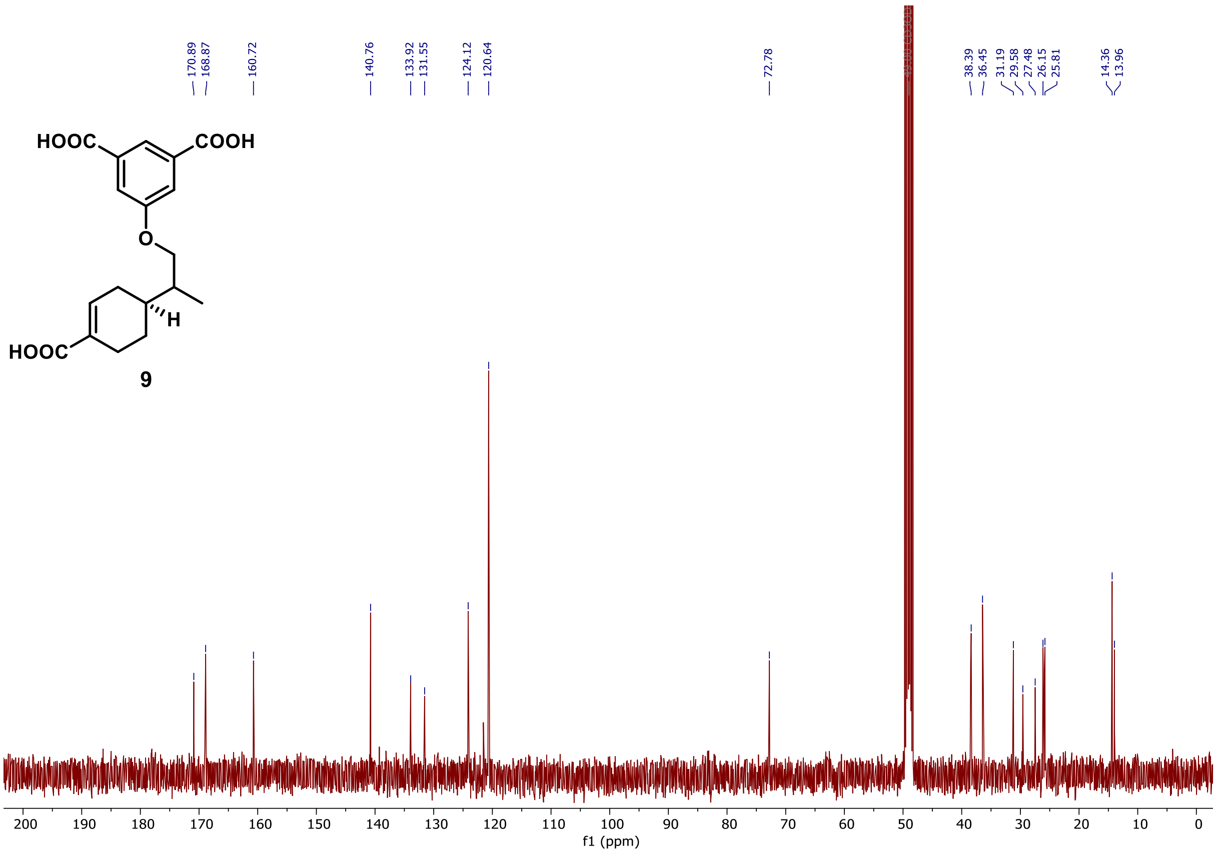

Figure S200: *^{13}C NMR spectrum of tricarboxylic acid **9** (101 MHz, MeOD).*

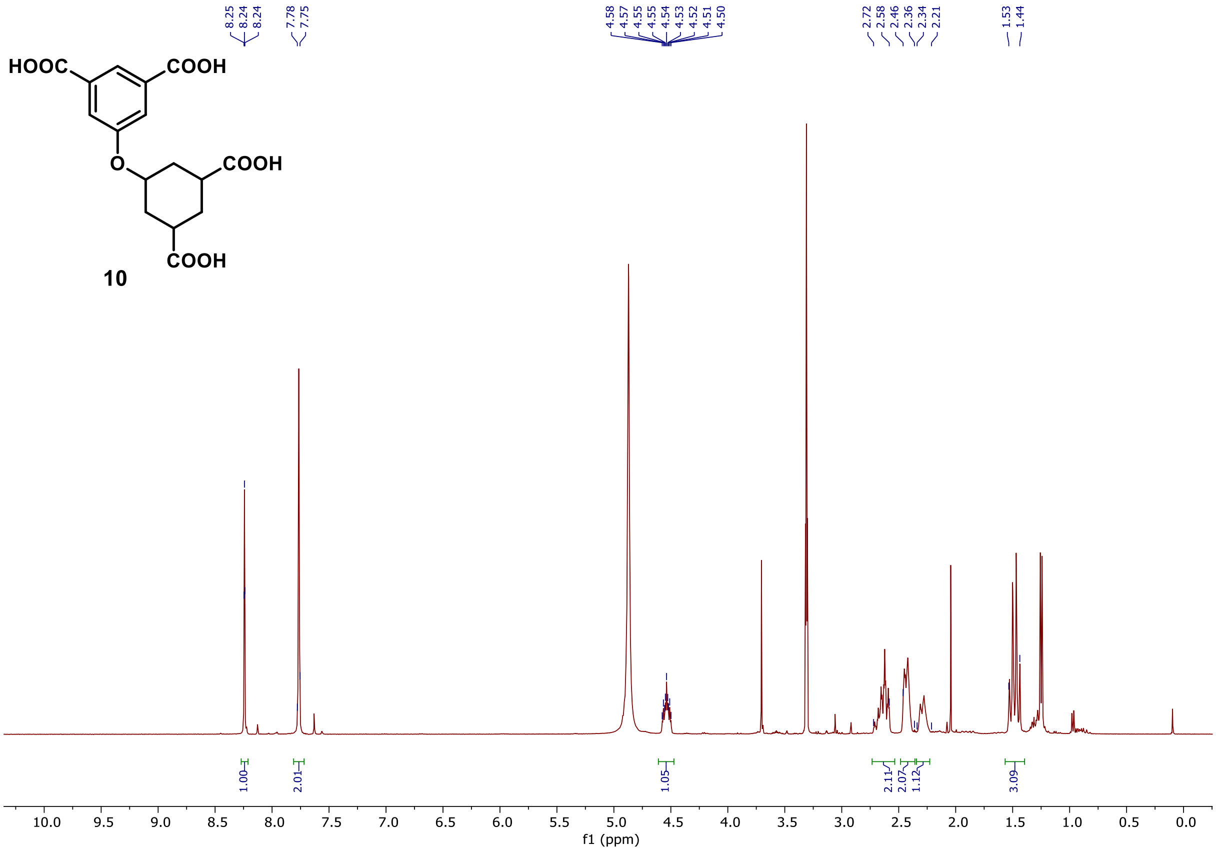

Figure S201: *^{1}H NMR spectra of tetracarboxylic acid* ***10*** *(400 MHz, MeOD).*

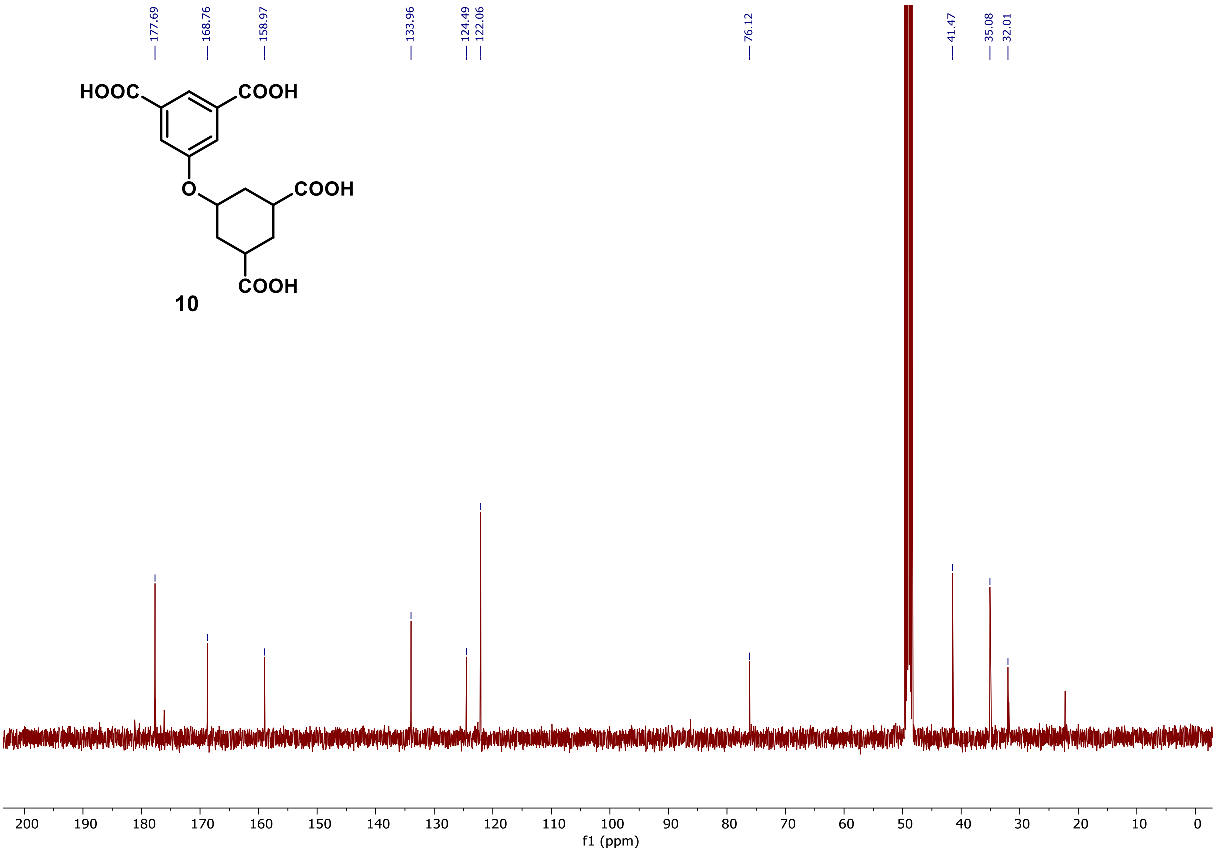

Figure S202: *^{13}C NMR spectrum of tetracarboxylic acid* ***10*** *(101 MHz, MeOD)****.***

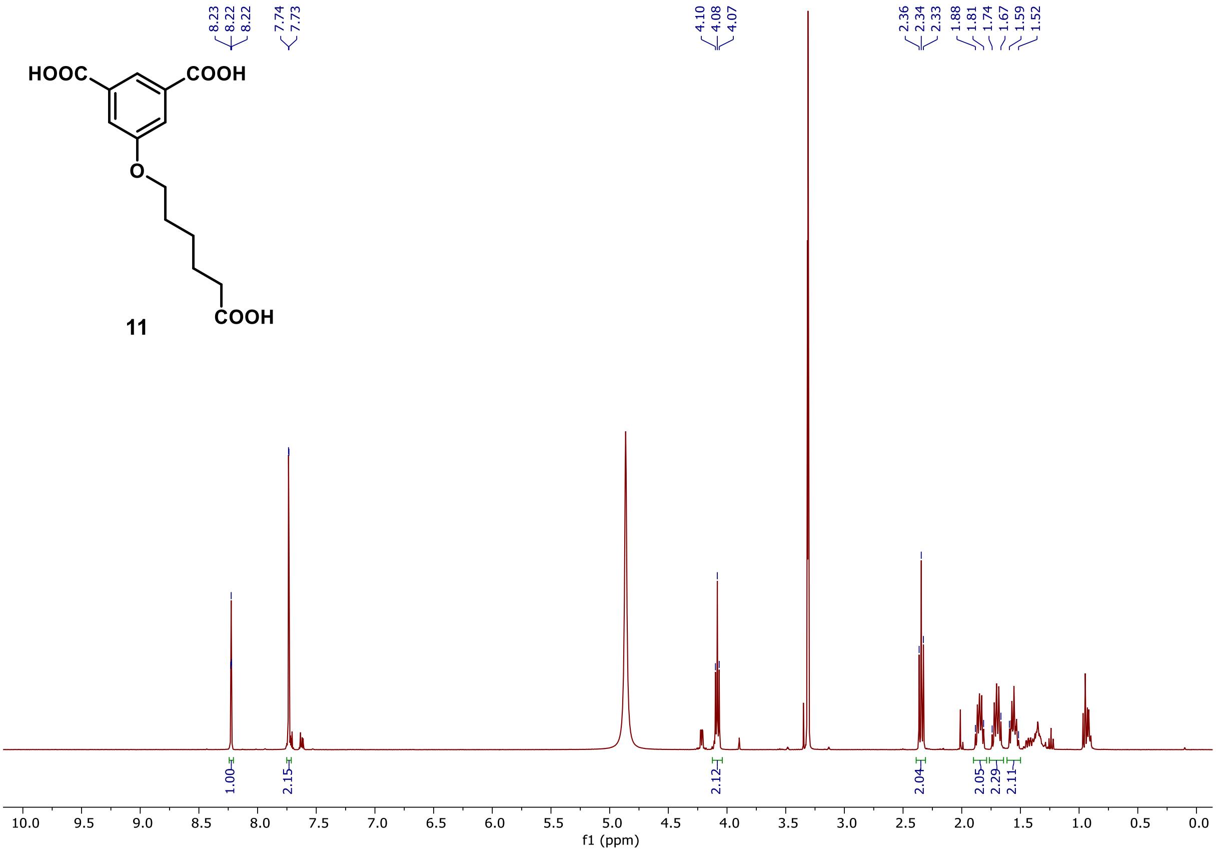

11

Figure S203: ^{1}H *NMR spectra of tricarboxylic acid* ***11*** *(400 MHz, MeOD).*

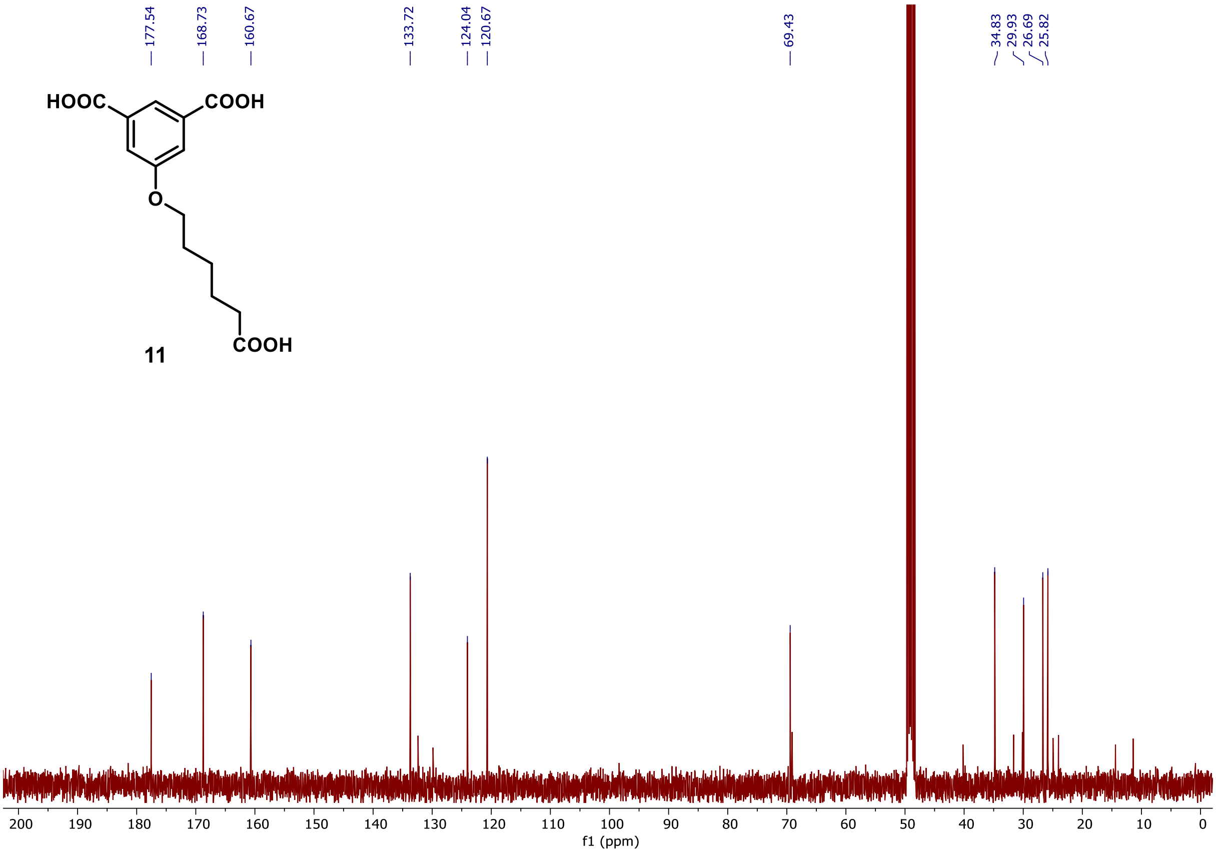

11

Figure S204: ^{13}C *NMR spectrum of tricarboxylic acid* ***11*** *(101 MHz, MeOD).*

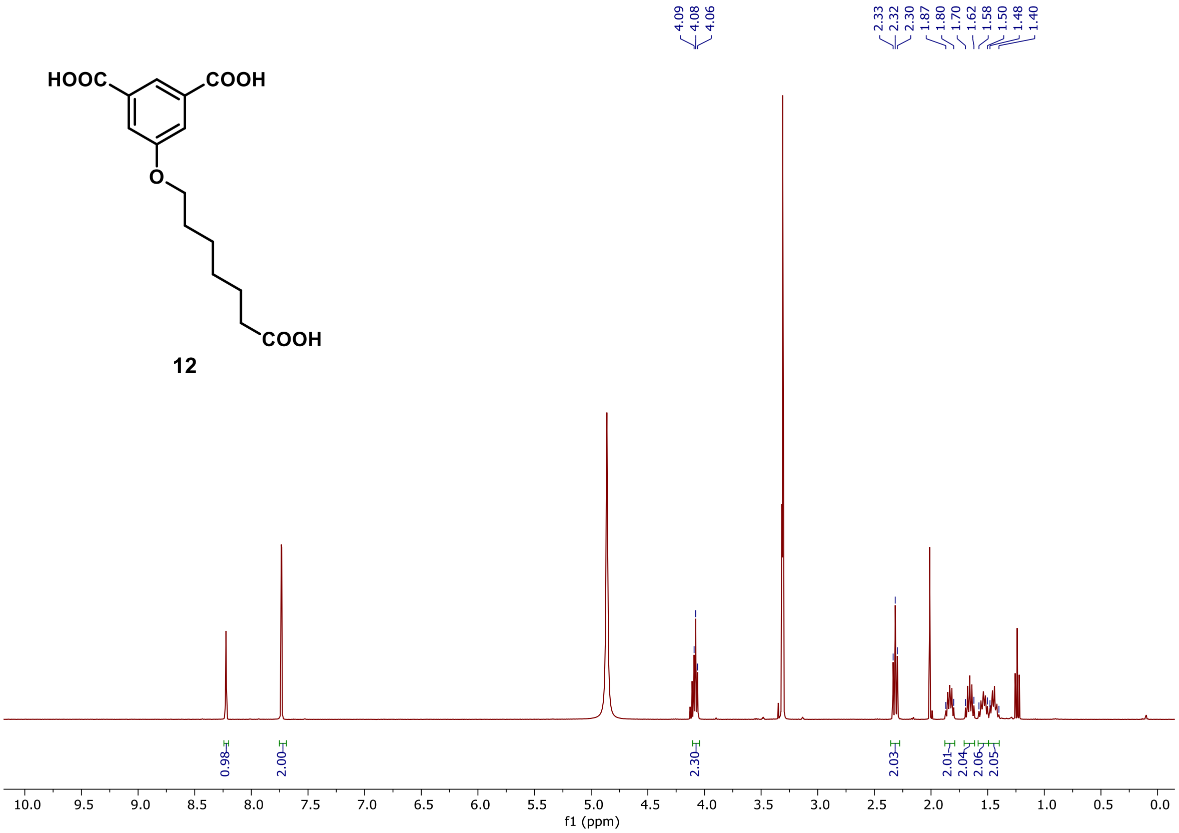

Figure S205: *^{1}H NMR spectra of tricarboxylic acid* ***12*** *(400 MHz, MeOD).*

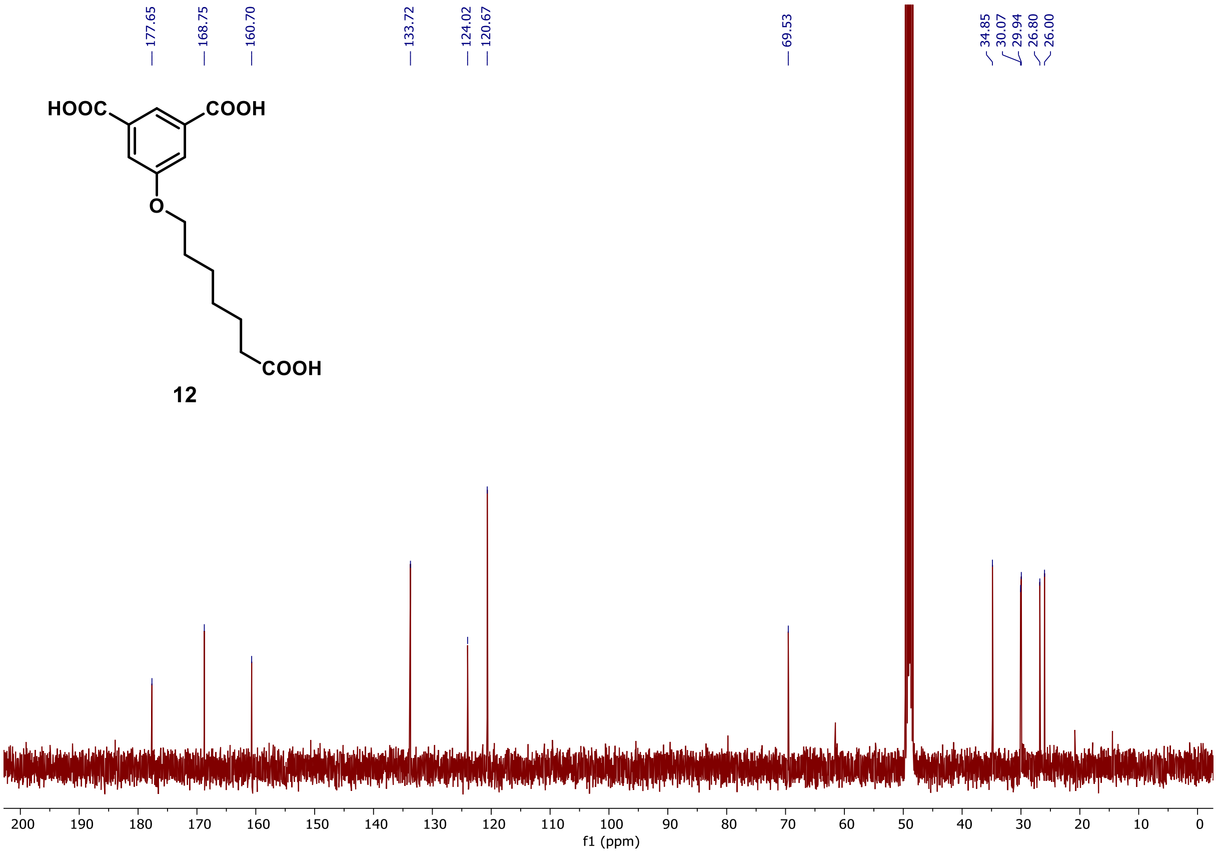

Figure S206: *^{13}C NMR spectrum of tricarboxylic acid* ***12*** *(101 MHz, MeOD).*

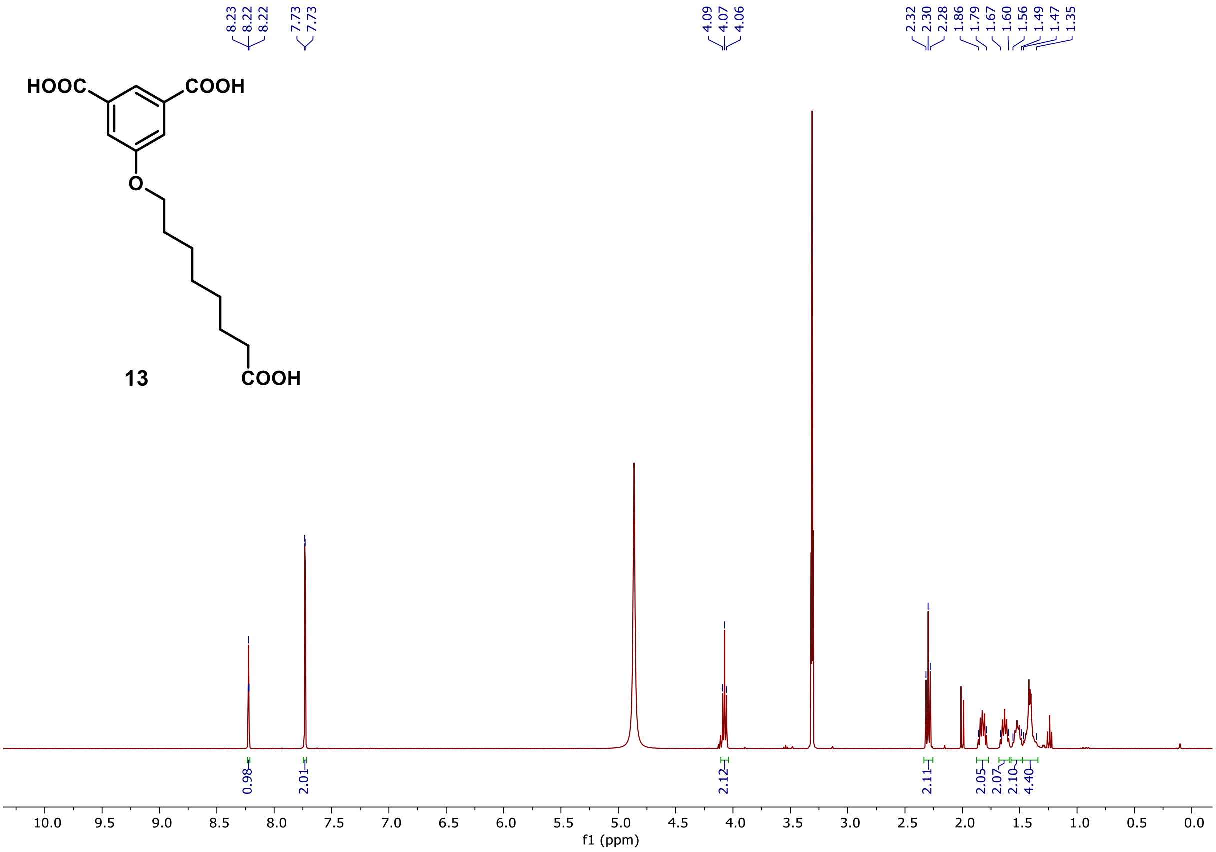

Figure S207: *^{1}H NMR spectra of tricarboxylic acid **13** (400 MHz, MeOD).*

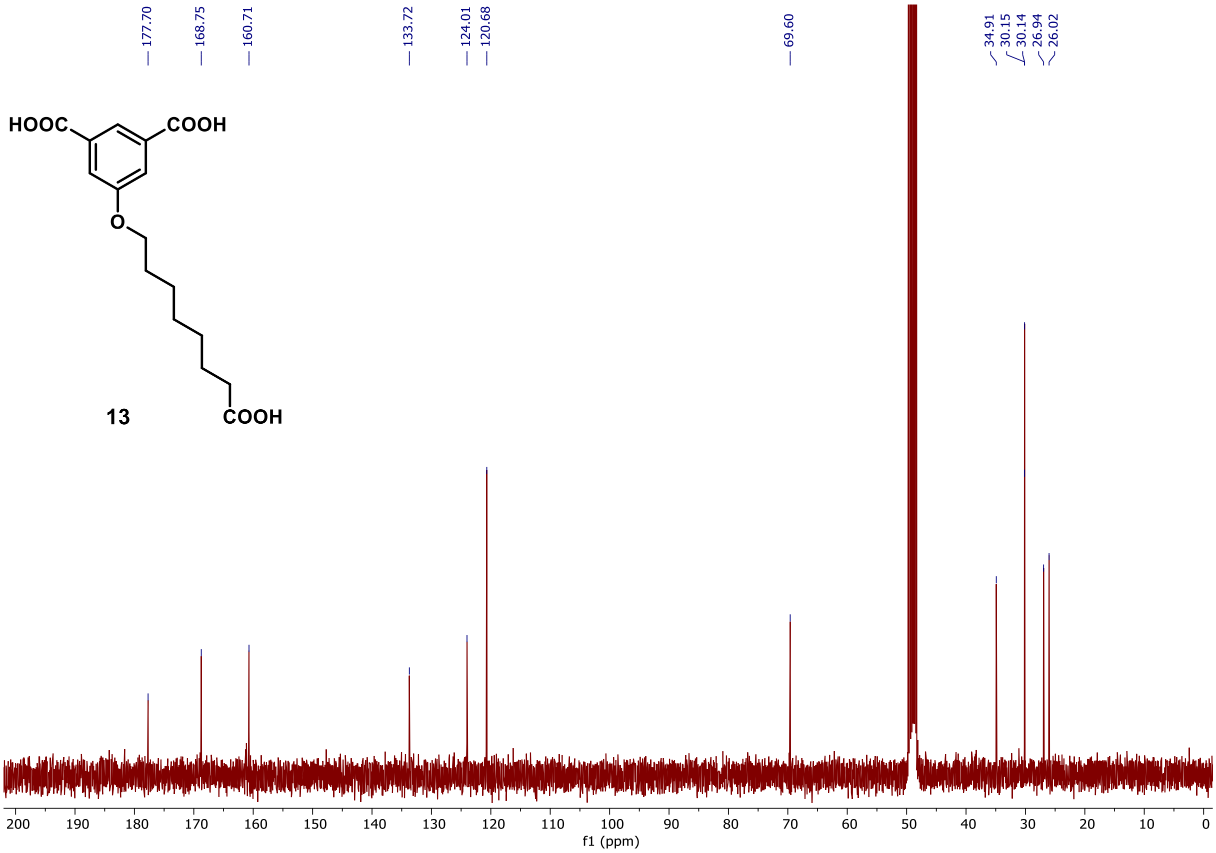

Figure S208: *^{13}C NMR spectrum of tricarboxylic acid **13** (101 MHz, MeOD).*

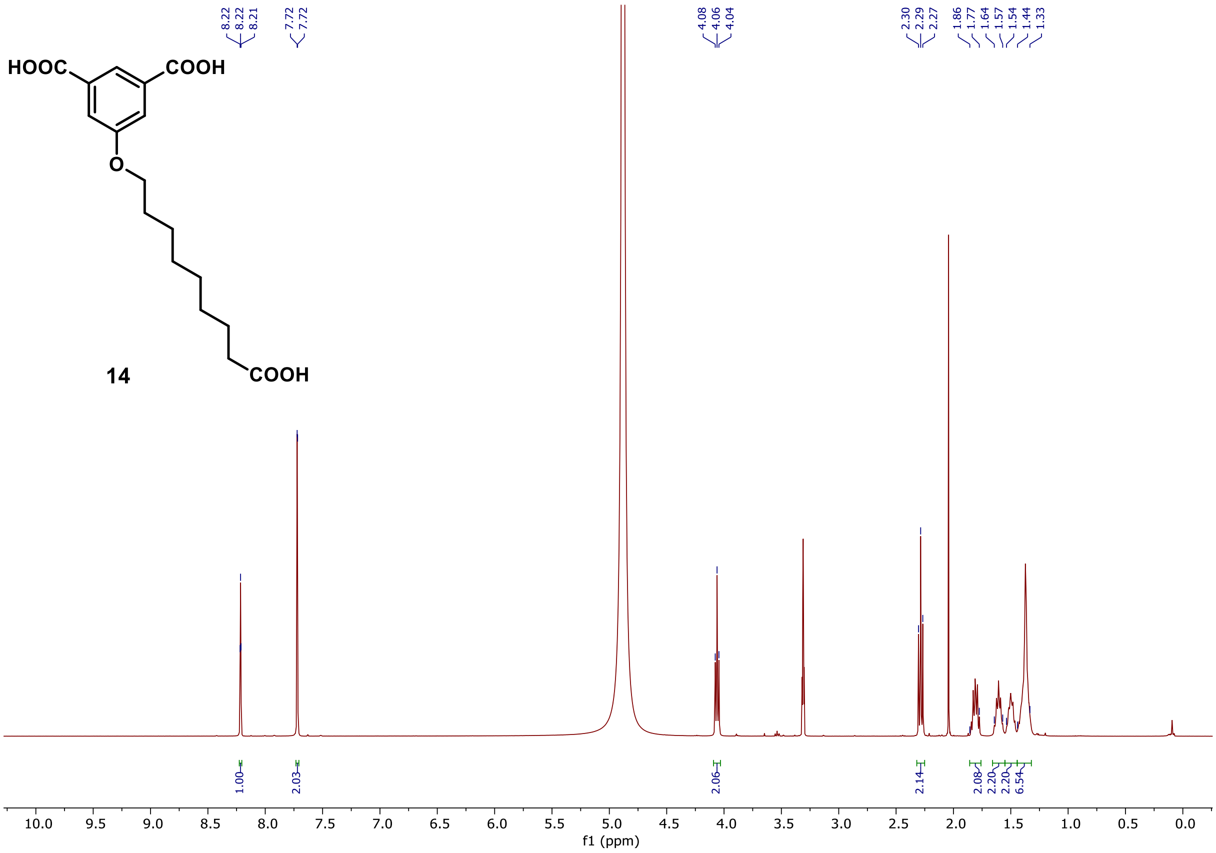

Figure S209: *^{1}H NMR spectra of tricarboxylic acid **14** (400 MHz, MeOD).*

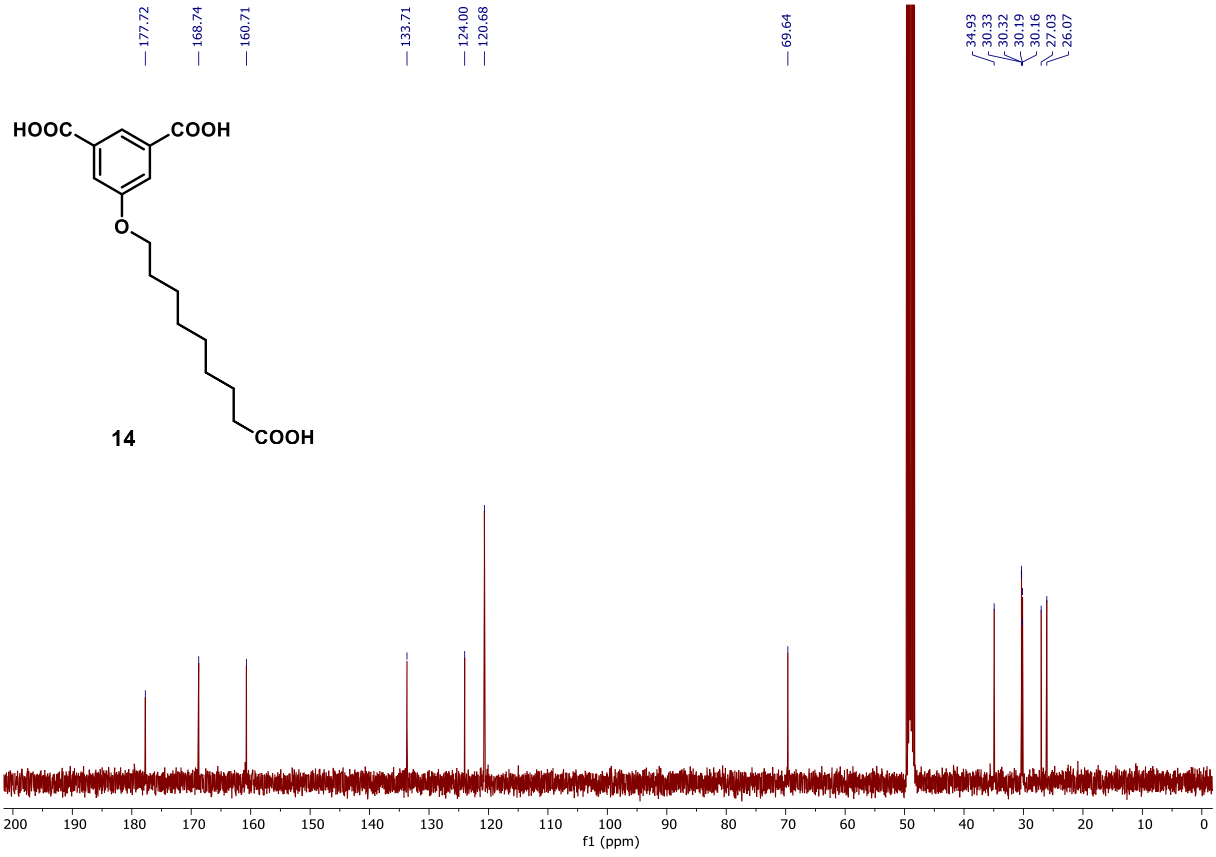

Figure S210: *^{13}C NMR spectrum of tricarboxylic acid **14** (101 MHz, MeOD).*

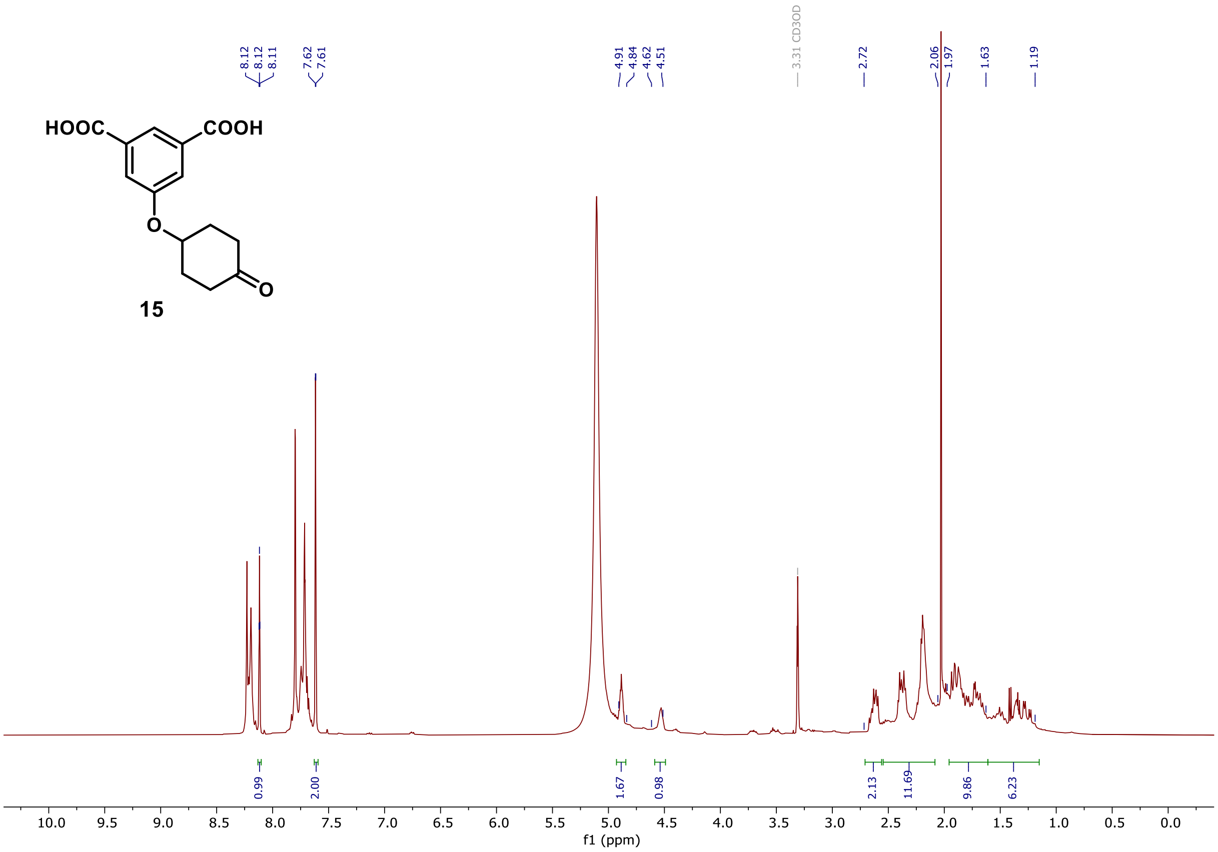

Figure S211: *^{1}H NMR spectra of ketone **15** (400 MHz, MeOD).*

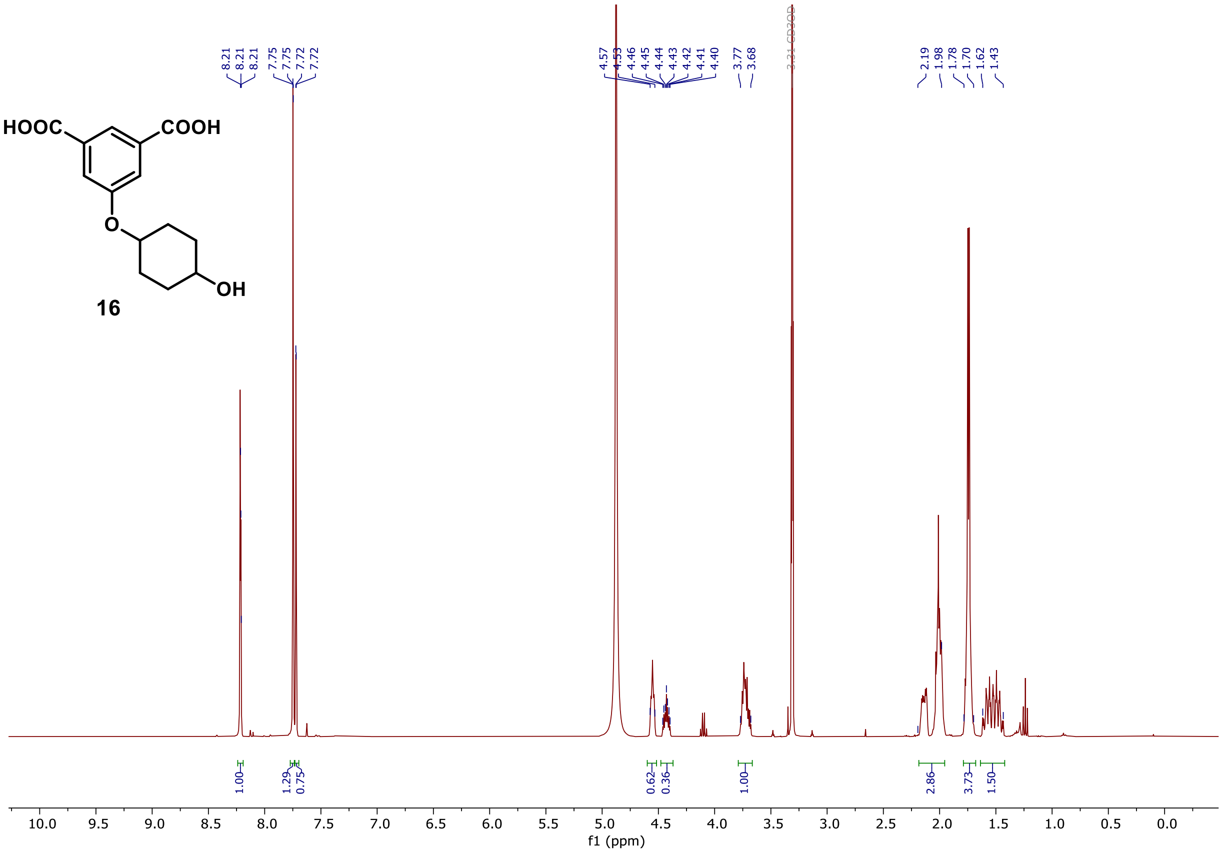

Figure S212: *^{1}H NMR spectra of alcohol **16** (400 MHz, MeOD).*

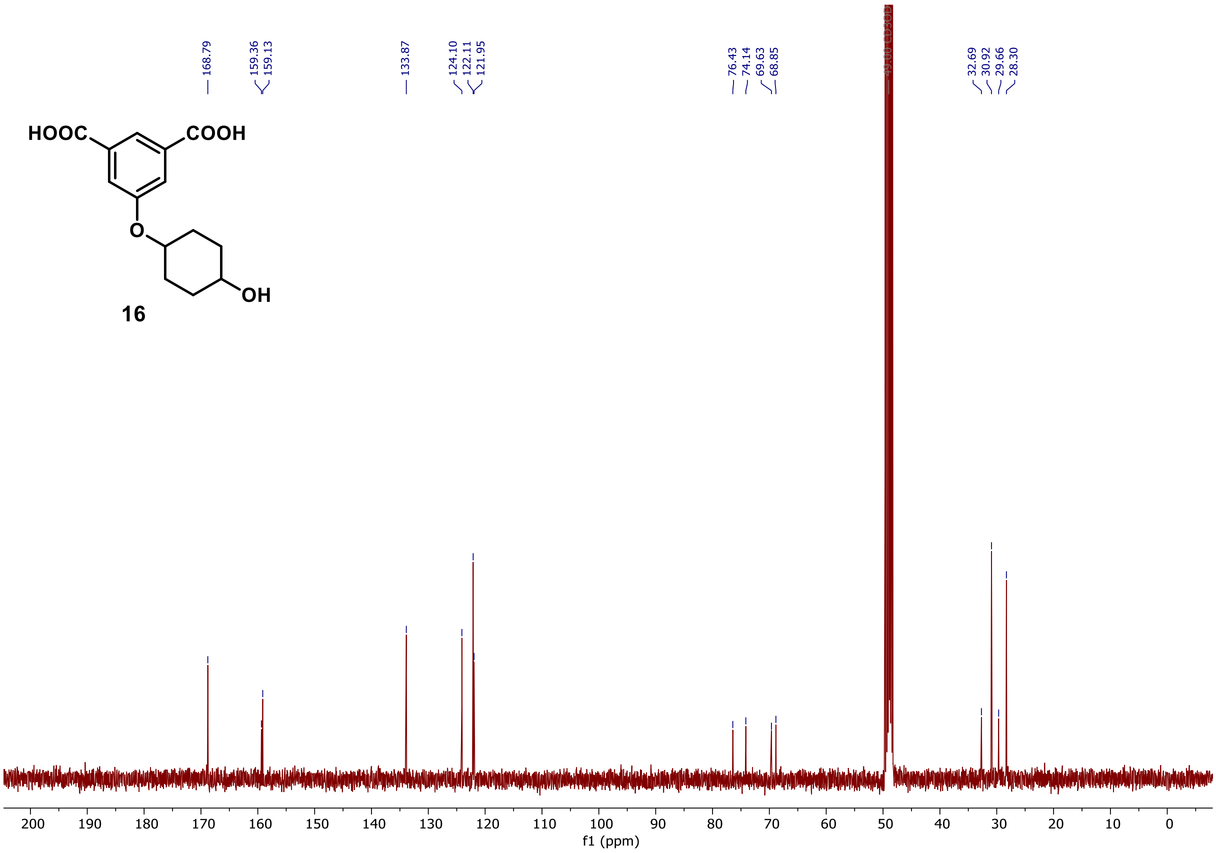

Figure S213: *^{13}C NMR spectrum of alcohol **16** (101 MHz, MeOD).*

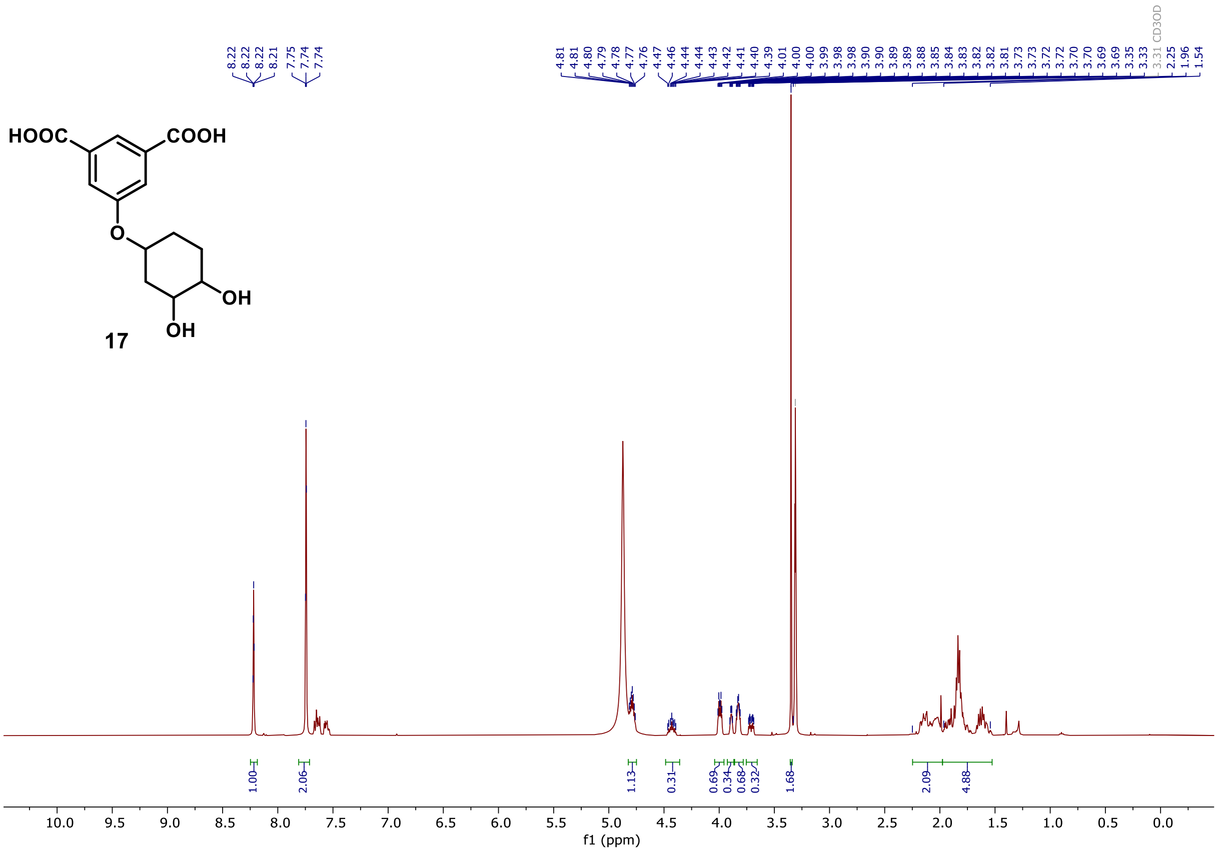

Figure S214: ¹H NMR spectra of diol **17** (400 MHz, MeOD).

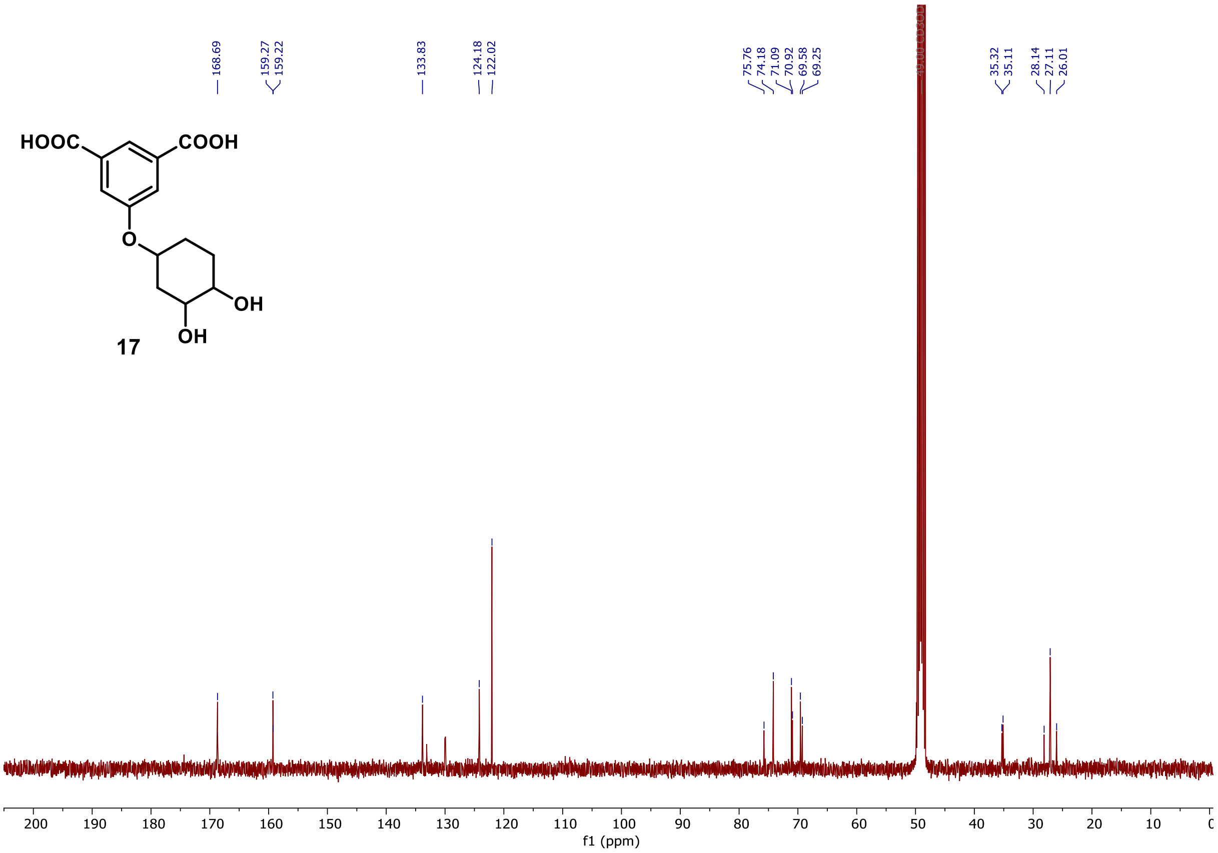

Figure S215: ¹³C NMR spectrum of diol **17** (101 MHz, MeOD).

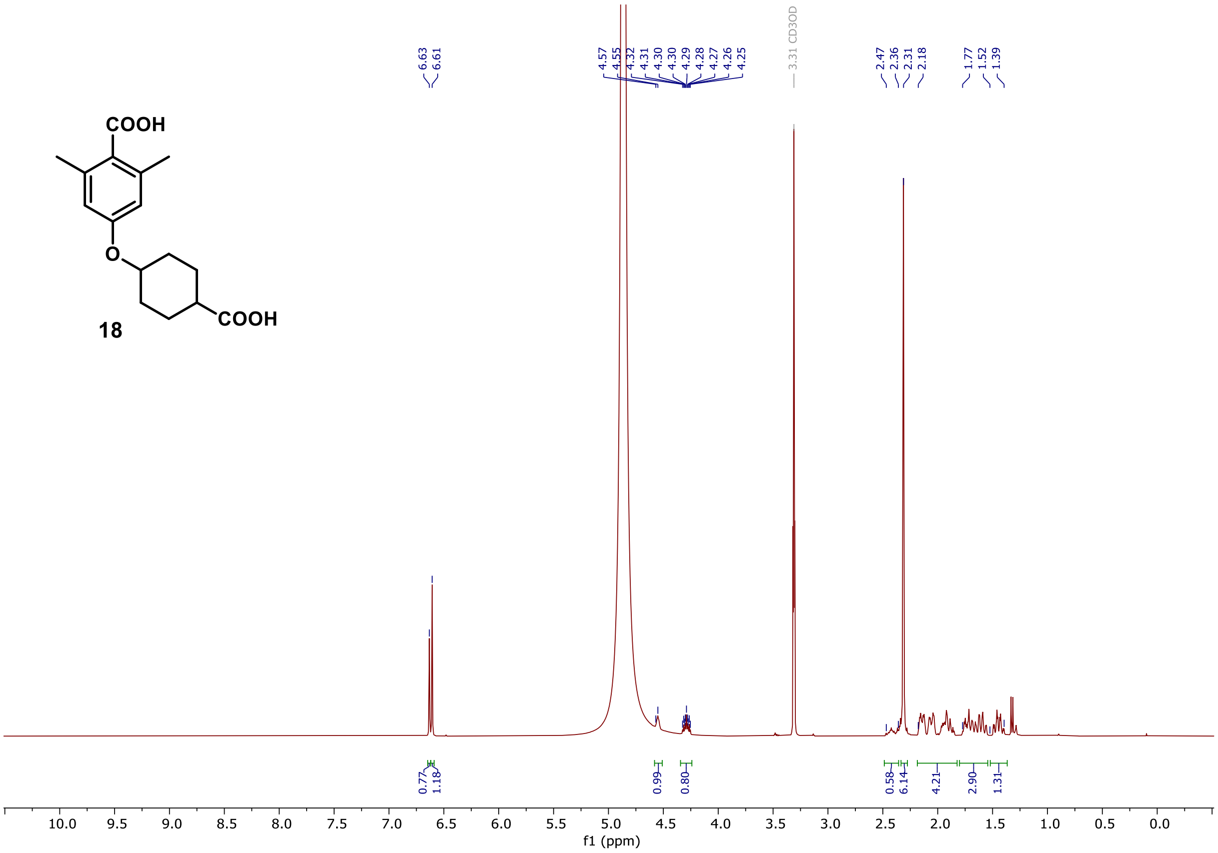

Figure S216: *^{1}H NMR spectra of dicarboxylic acid **18** (400 MHz, MeOD).*

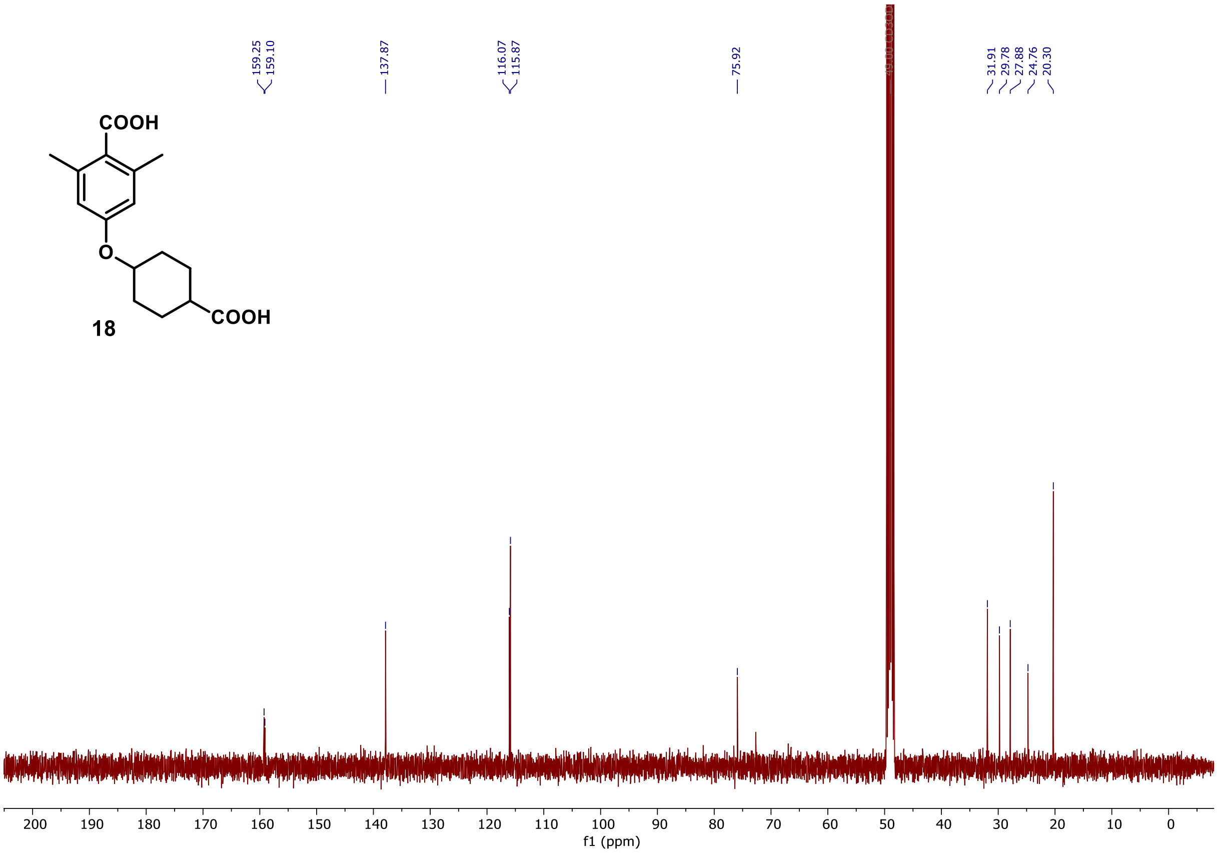

Figure S217: *^{13}C NMR spectrum of dicarboxylic acid **18** (101 MHz, MeOD).*

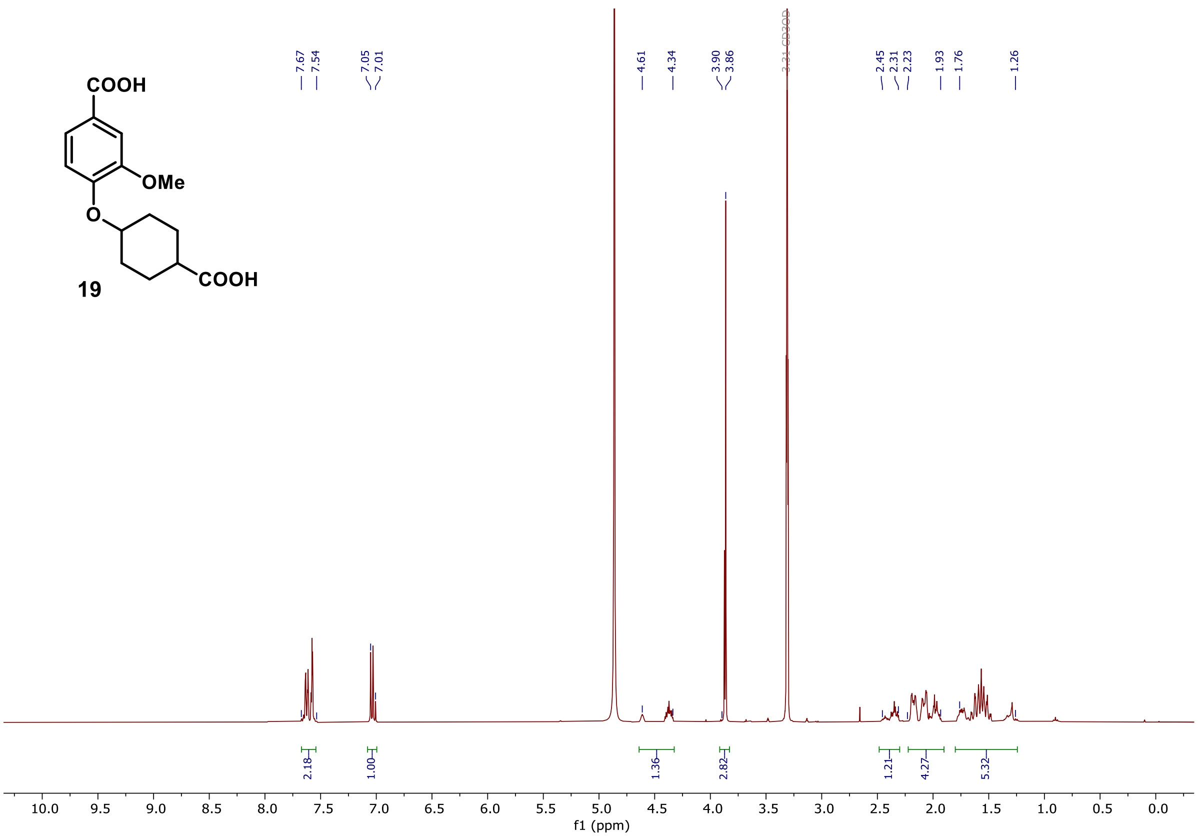

Figure S218: *^{1}H NMR spectra of methoxy ether **19** (400 MHz, MeOD).*

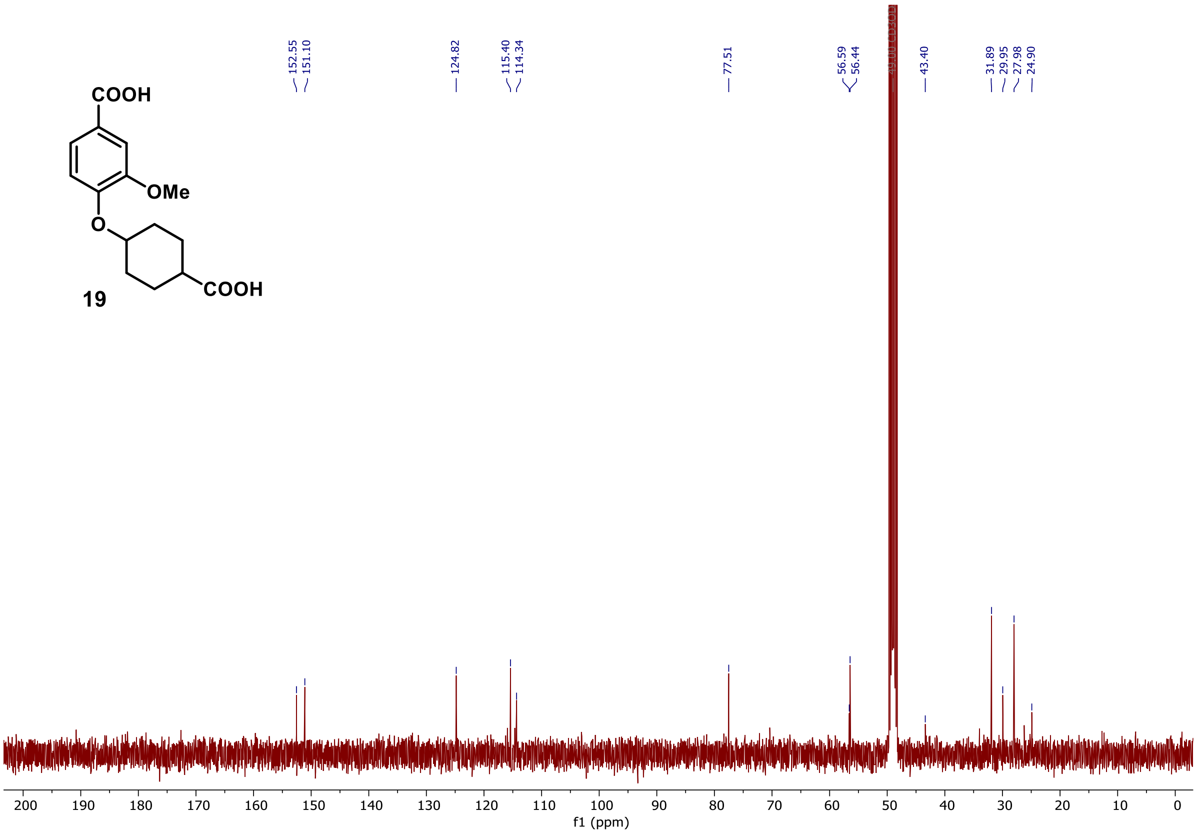

Figure S219: *^{13}C NMR spectrum of methoxy ether **19** (101 MHz, MeOD).*

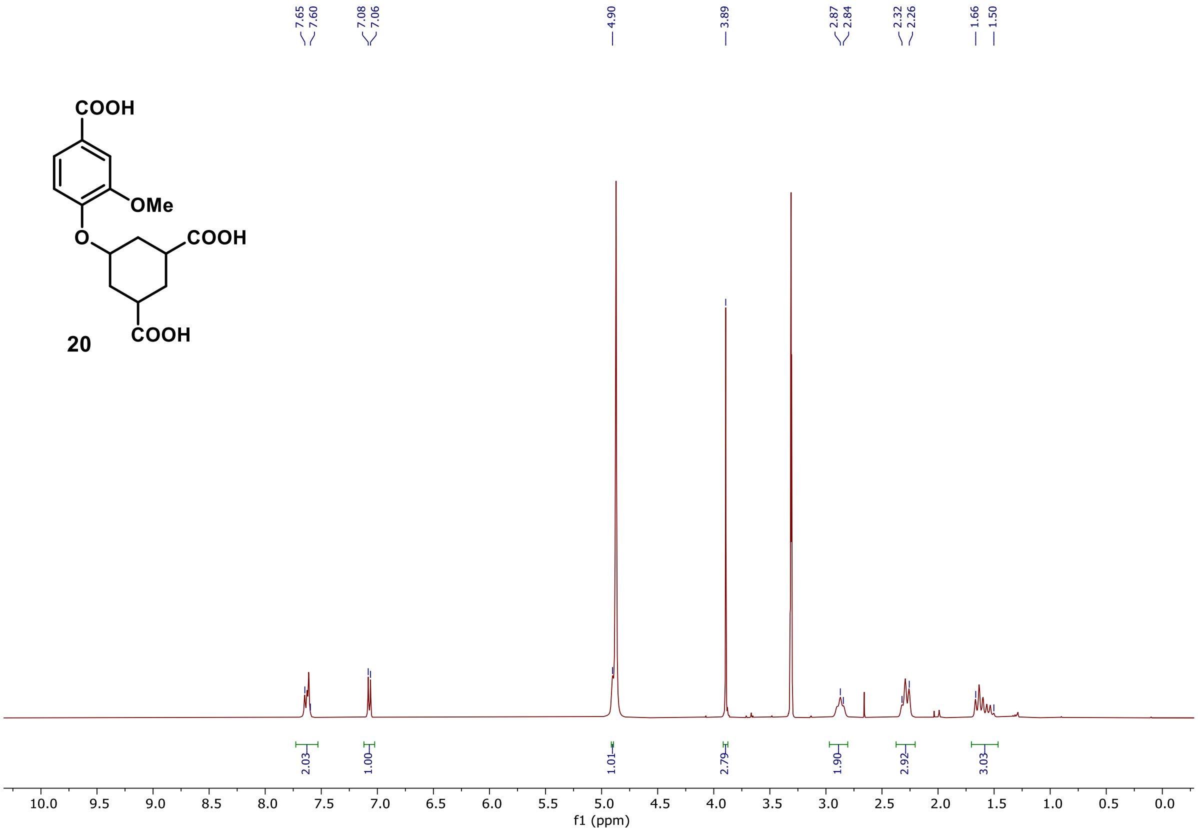

Figure S220: *^{1}H NMR spectra of methoxy ether* ***20*** *(400 MHz, MeOD).*

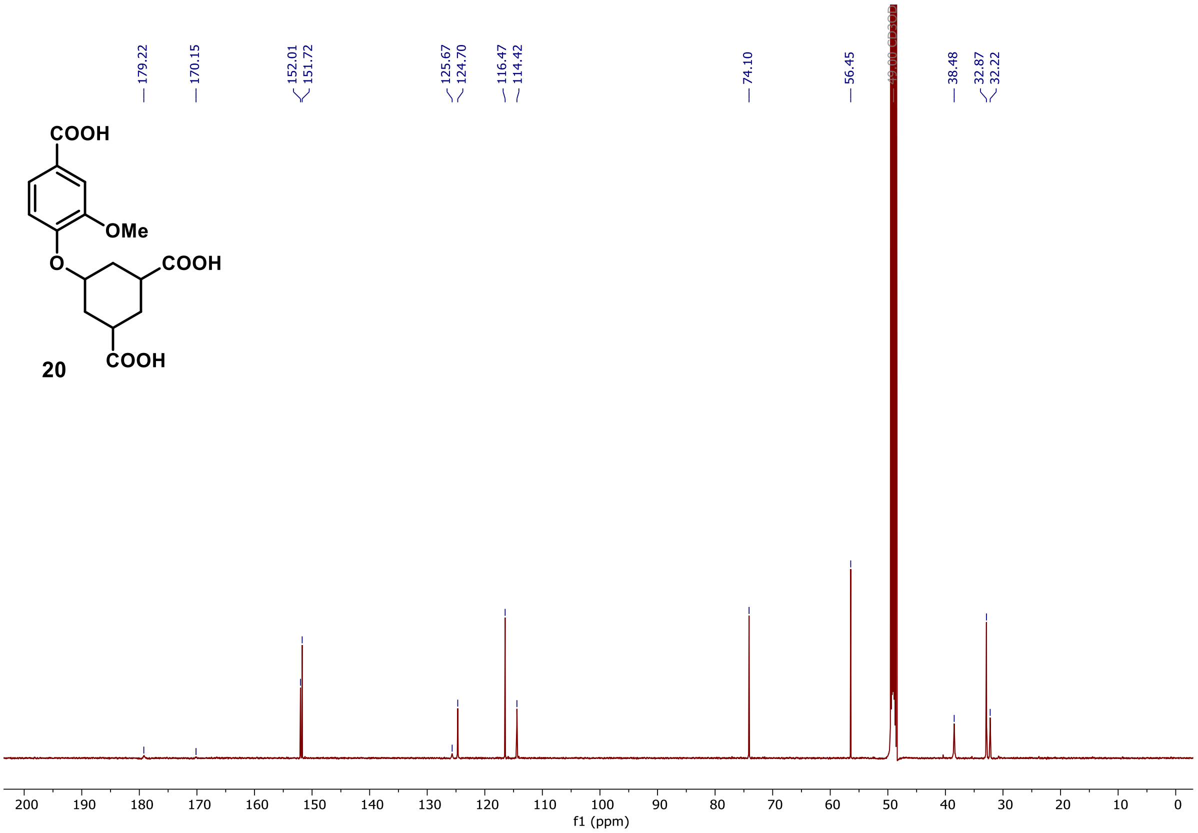

Figure S221: *^{13}C NMR spectrum of methoxy ether* ***20*** *(101 MHz, MeOD).*

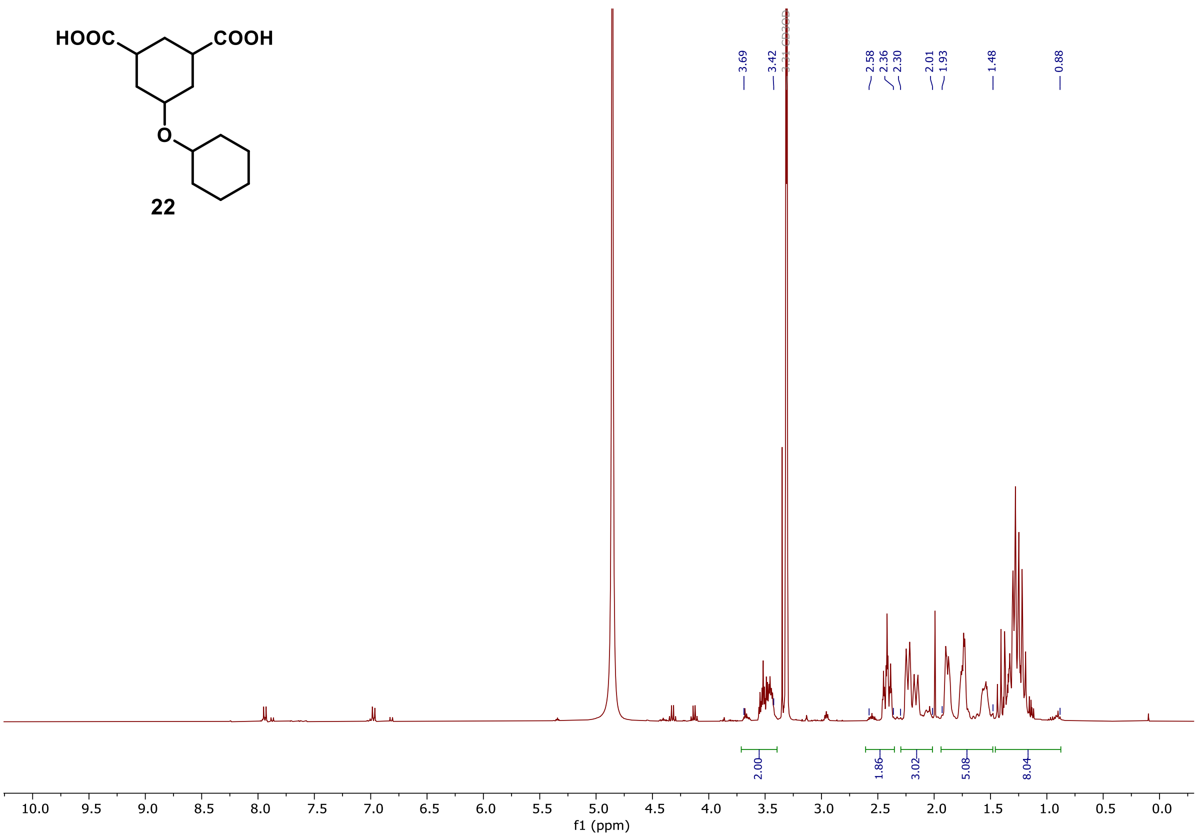

Figure S222: *^{1}H NMR spectra of dicarboxylic acid* ***22*** *(400 MHz, MeOD).*

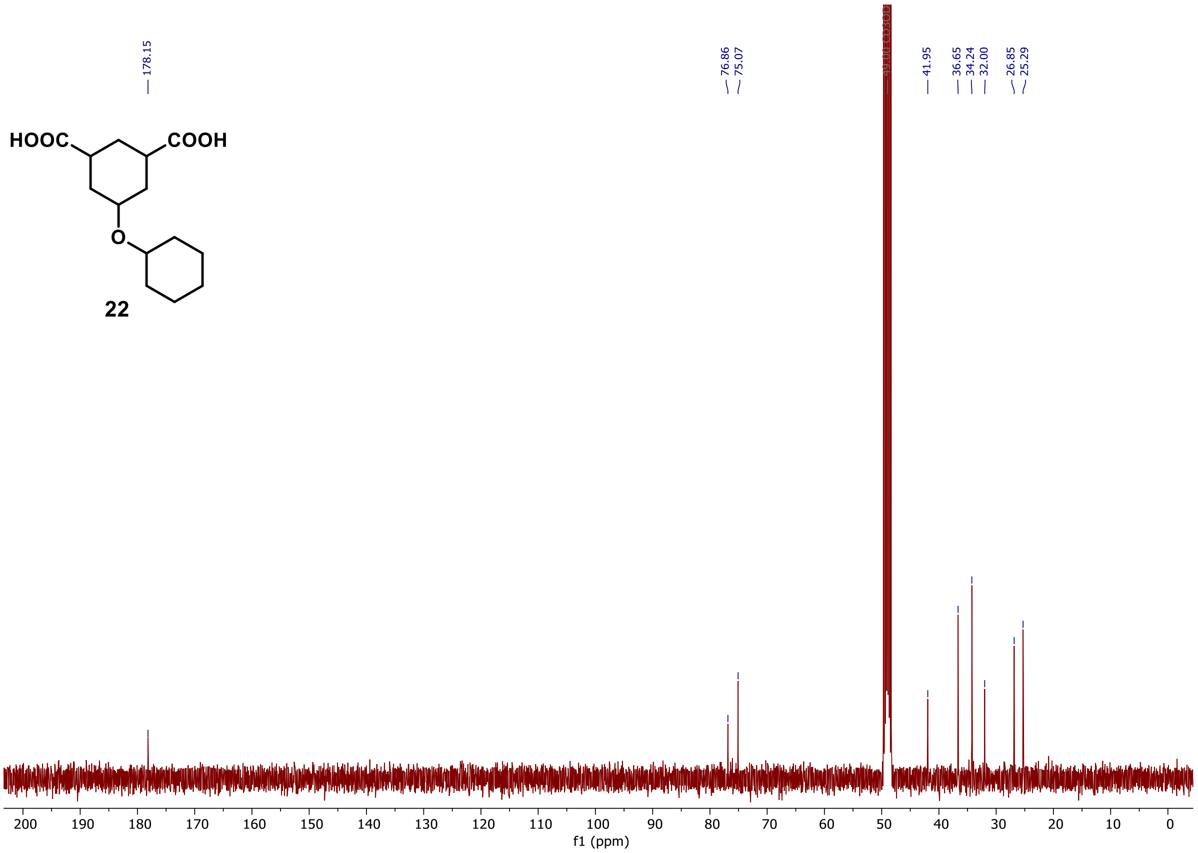

Figure S223: *^{13}C NMR spectrum of dicarboxylic acid* ***22*** *(101 MHz, MeOD).*

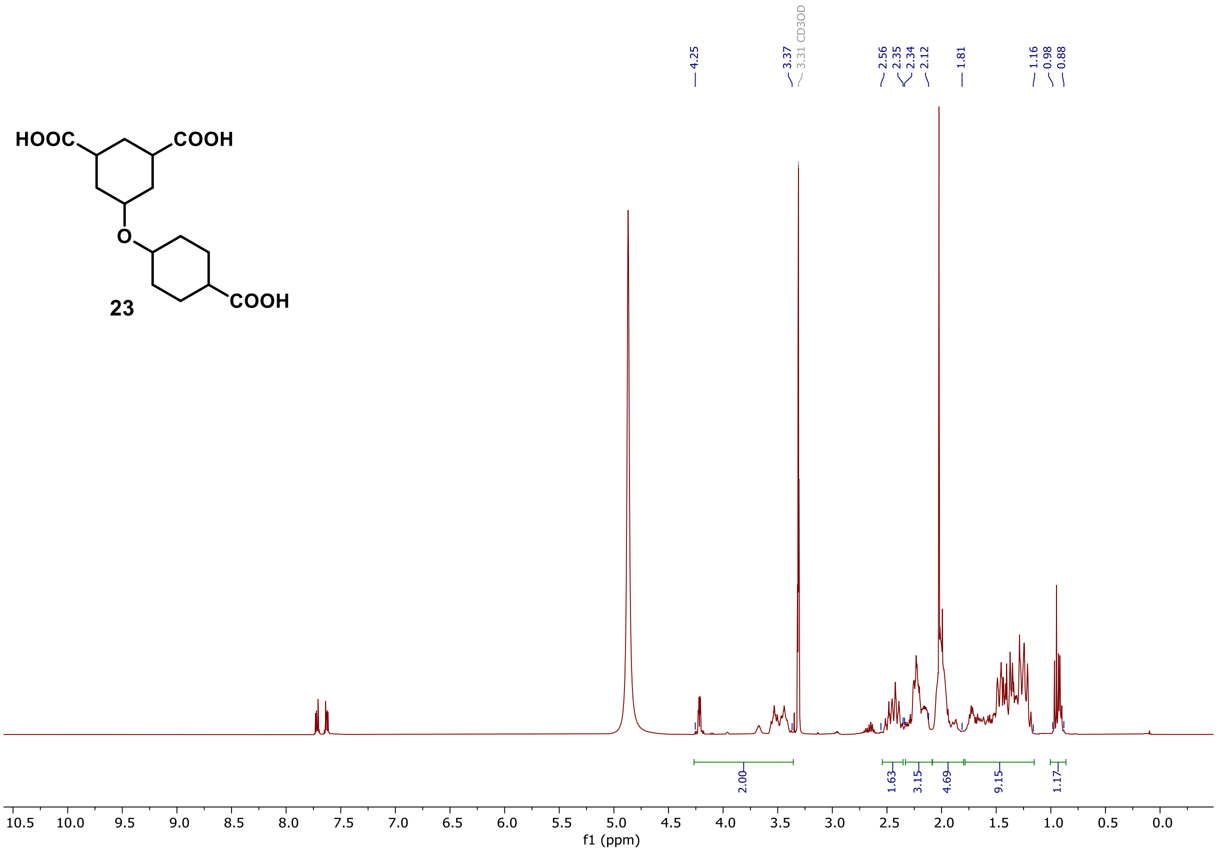

Figure S224: *^{1}H NMR spectra of tricarboxylic acid* ***23*** *(400 MHz, MeOD).*

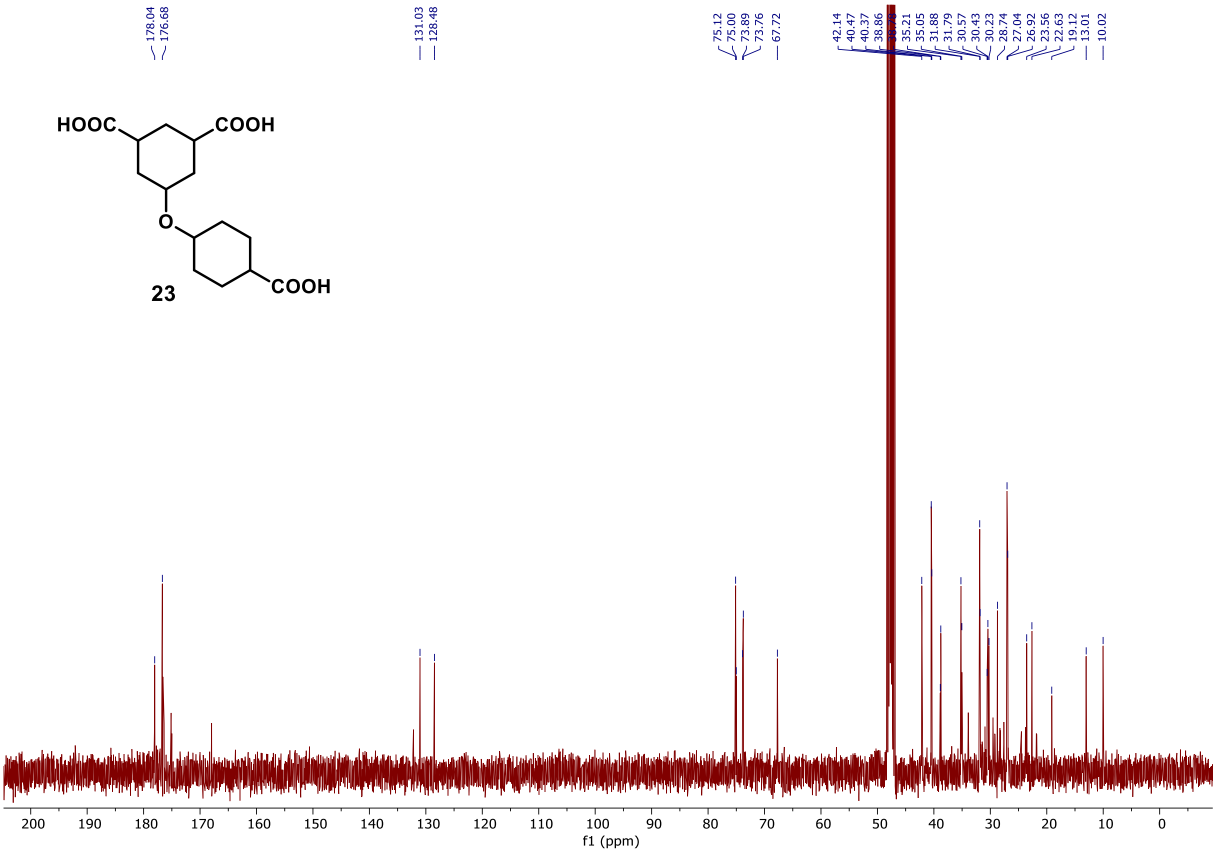

Figure S225: *^{13}C NMR spectrum of tricarboxylic acid* ***23*** *(101 MHz, MeOD).*

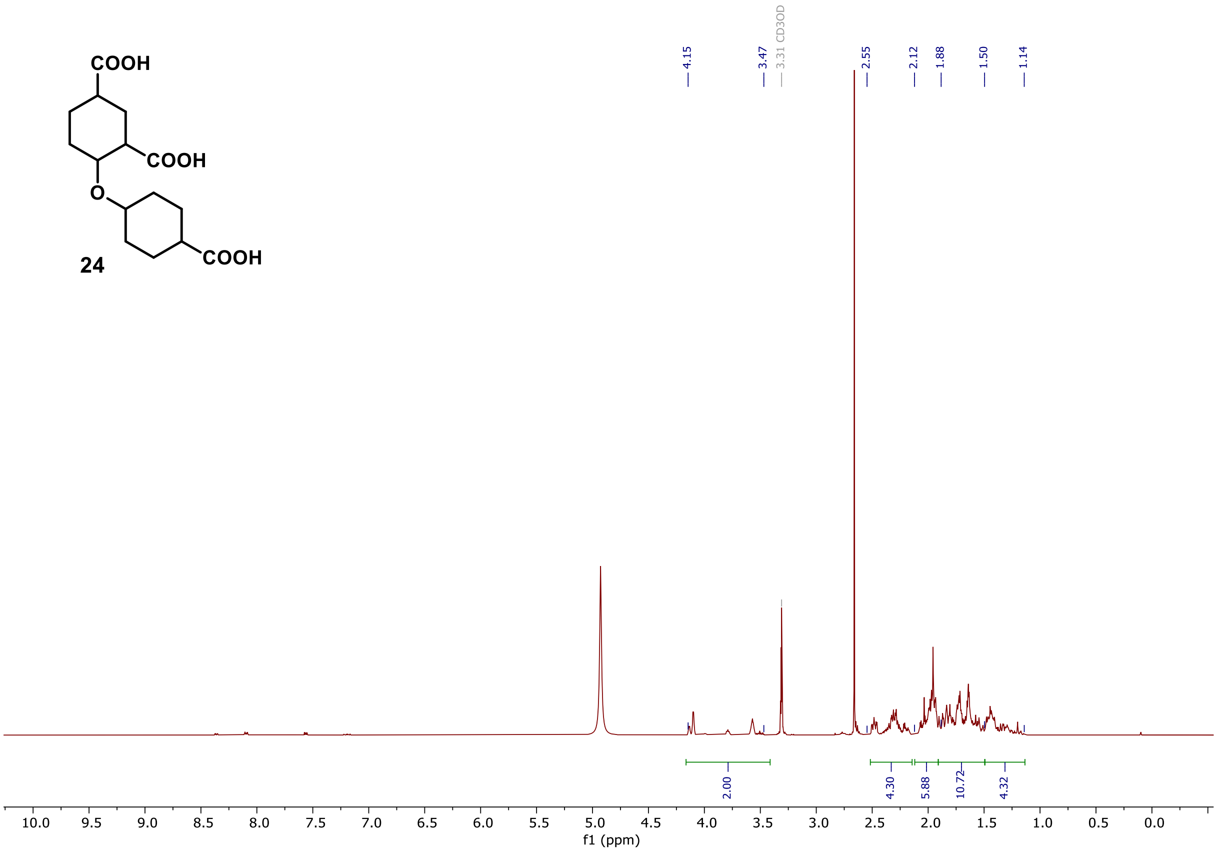

Figure S226: *^{1}H NMR spectra of tricarboxylic acid* ***24*** *(400 MHz, MeOD).*

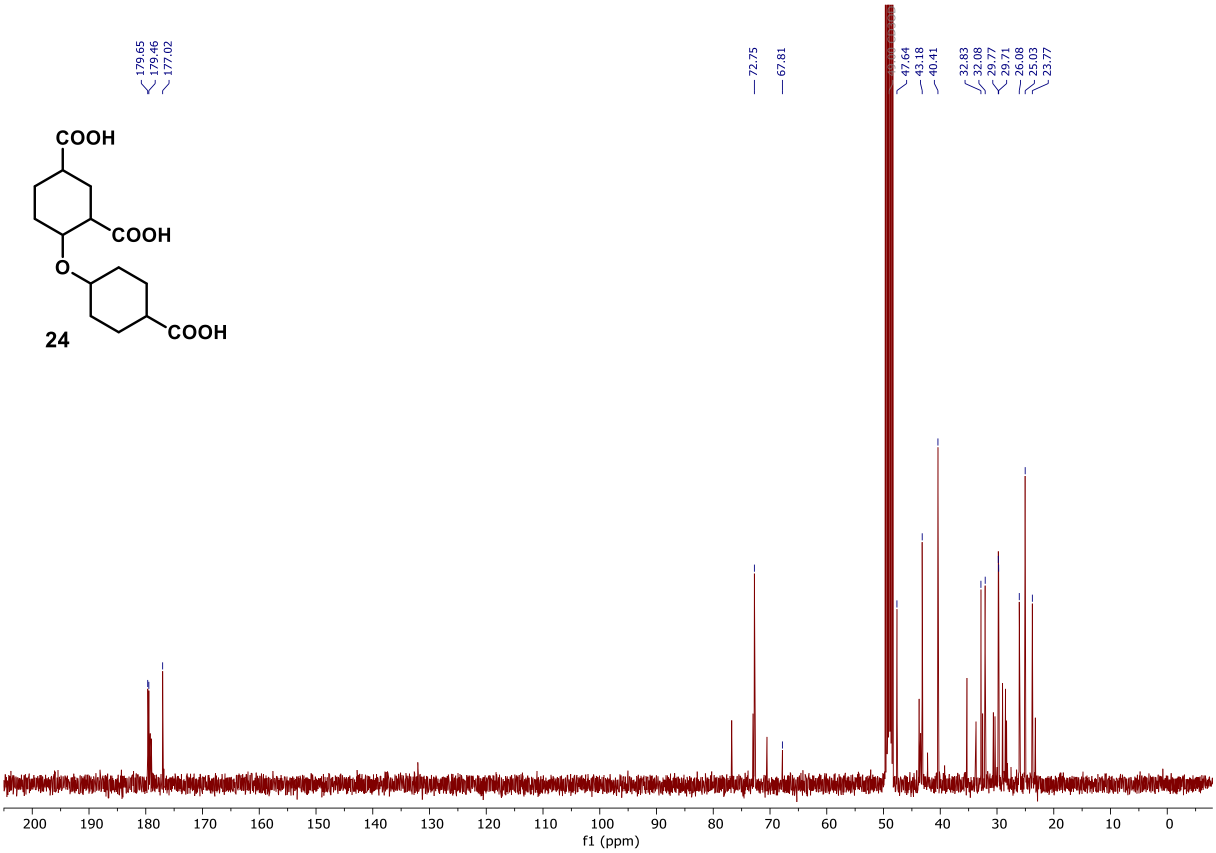

Figure S227: *^{13}C NMR spectrum of tricarboxylic acid* ***24*** *(101 MHz, MeOD).*

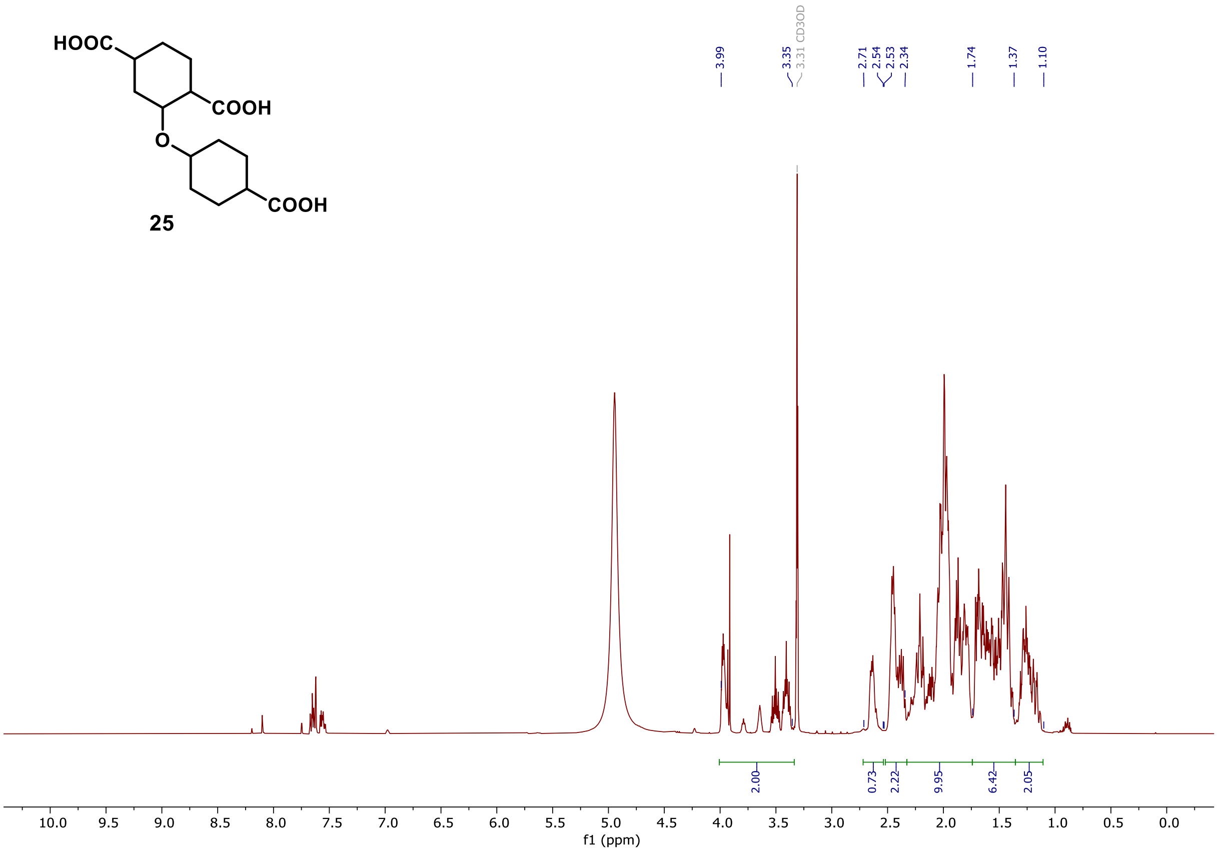

Figure S228: *^{1}H NMR spectra of tricarboxylic acid* ***25*** *(400 MHz, MeOD).*

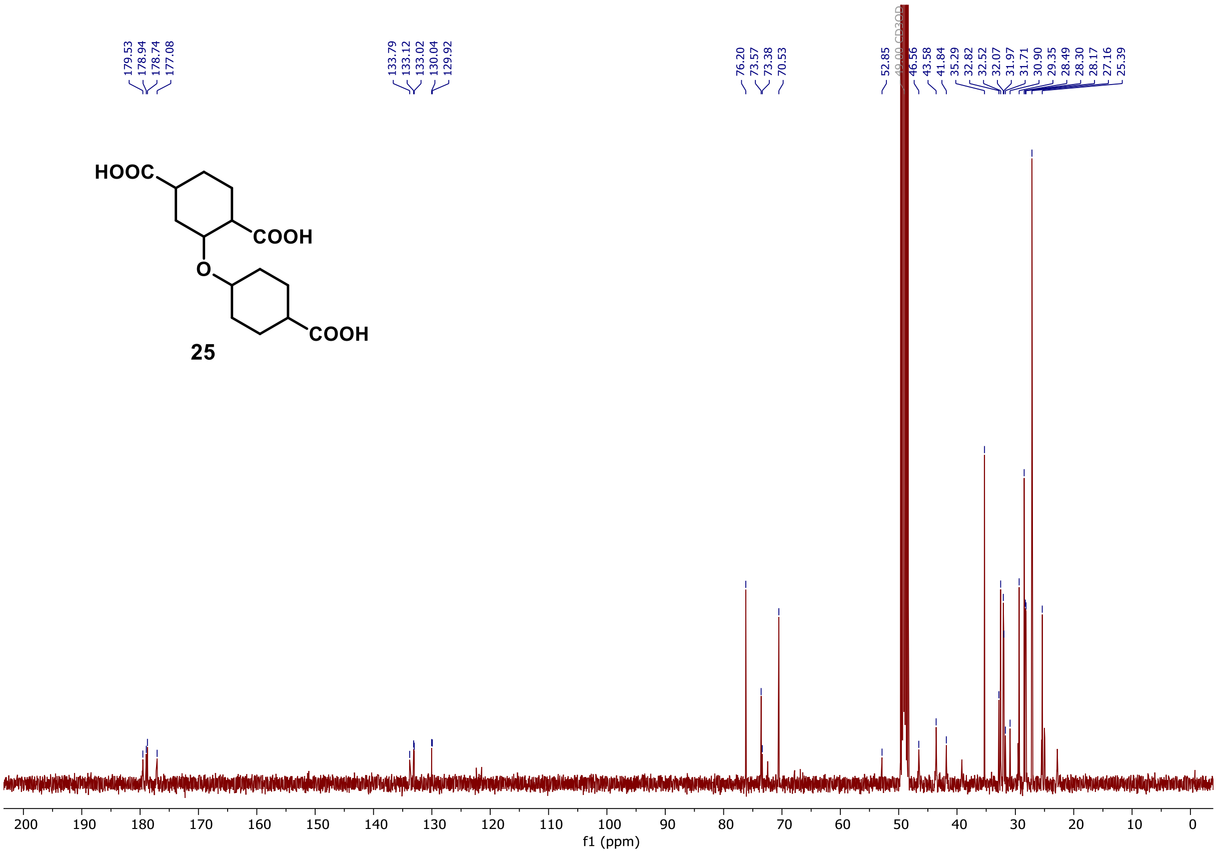

Figure S229: *^{13}C NMR spectrum of tricarboxylic acid* ***25*** *(101 MHz, MeOD).*

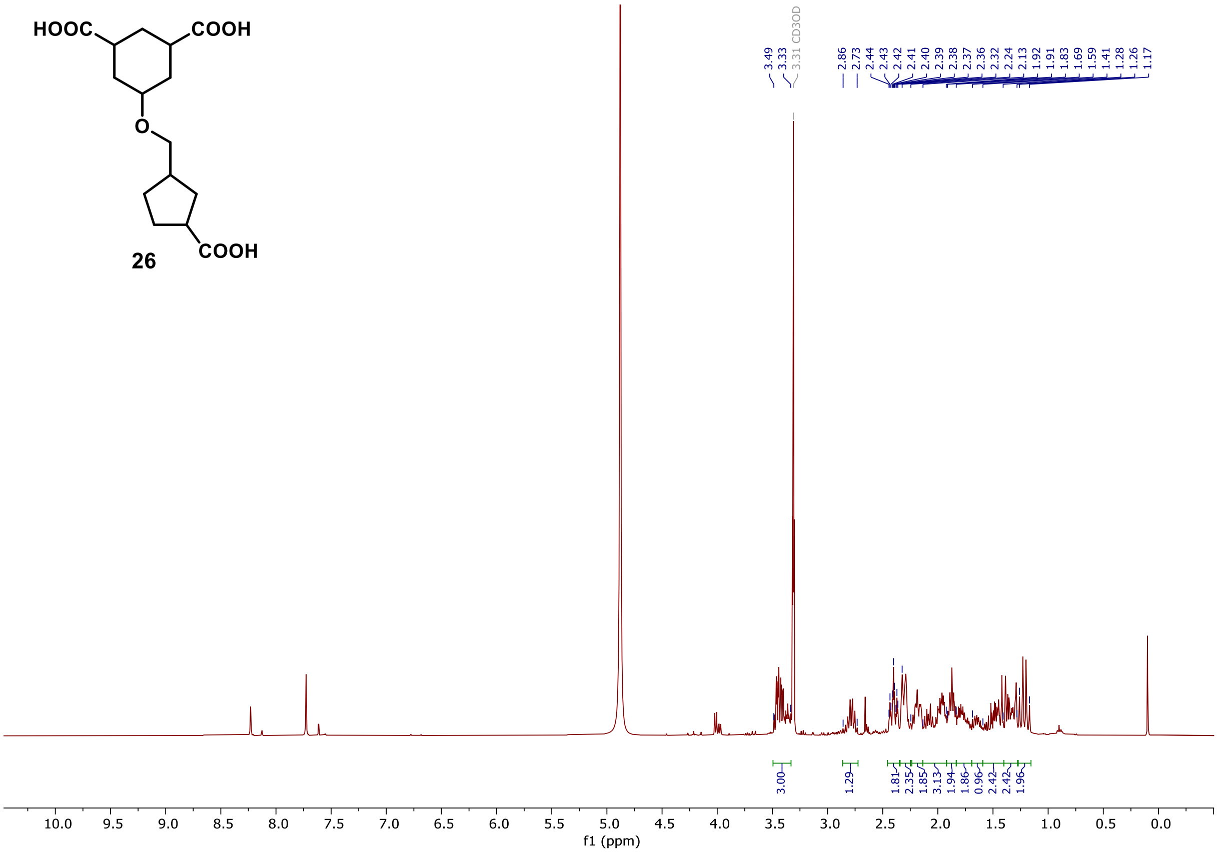

Figure S230: *^{1}H NMR spectra of tricarboxylic acid* ***26*** *(400 MHz, MeOD).*

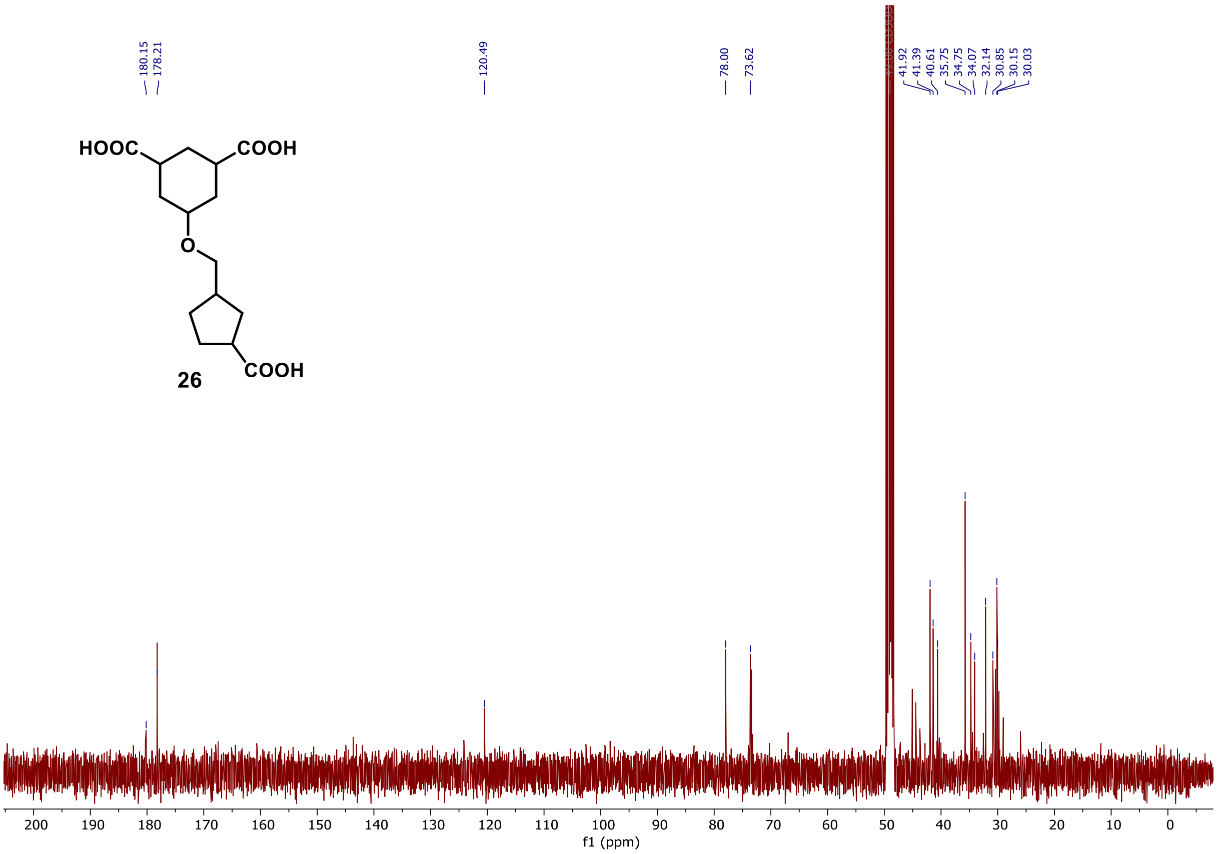

Figure S231: *^{13}C NMR spectrum of tricarboxylic acid* ***26*** *(101 MHz, MeOD).*

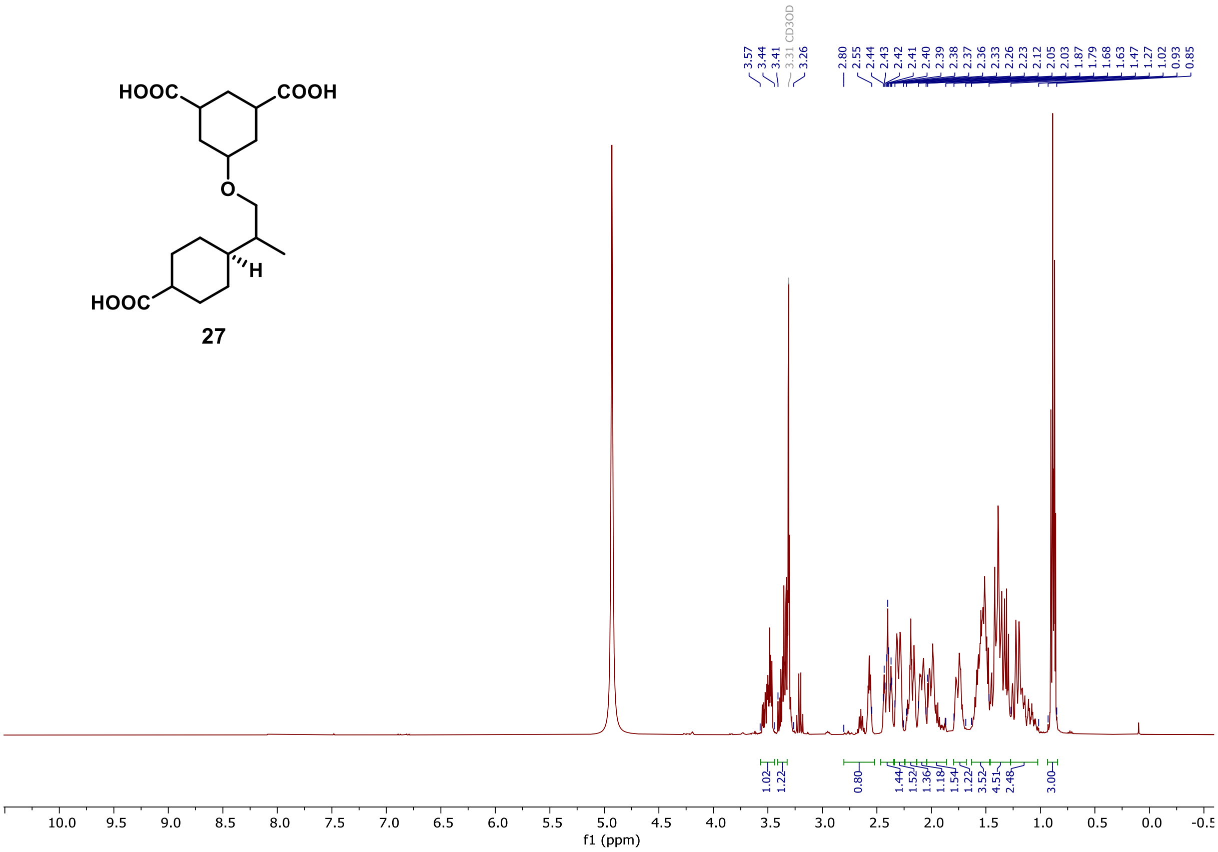

Figure S232: *^{1}H NMR spectra of tricarboxylic acid **27** (400 MHz, MeOD).*

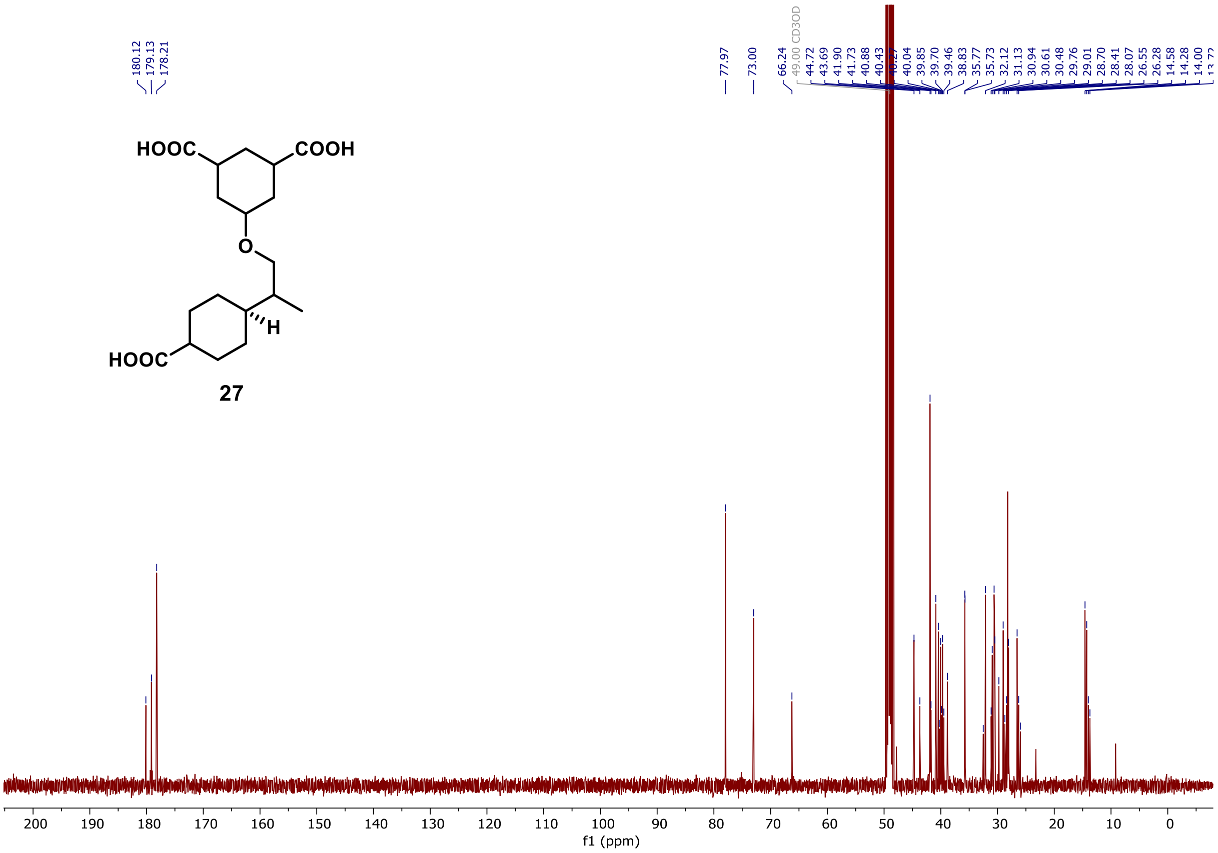

Figure S233: *^{13}C NMR spectrum of tricarboxylic acid **27** (101 MHz, MeOD).*

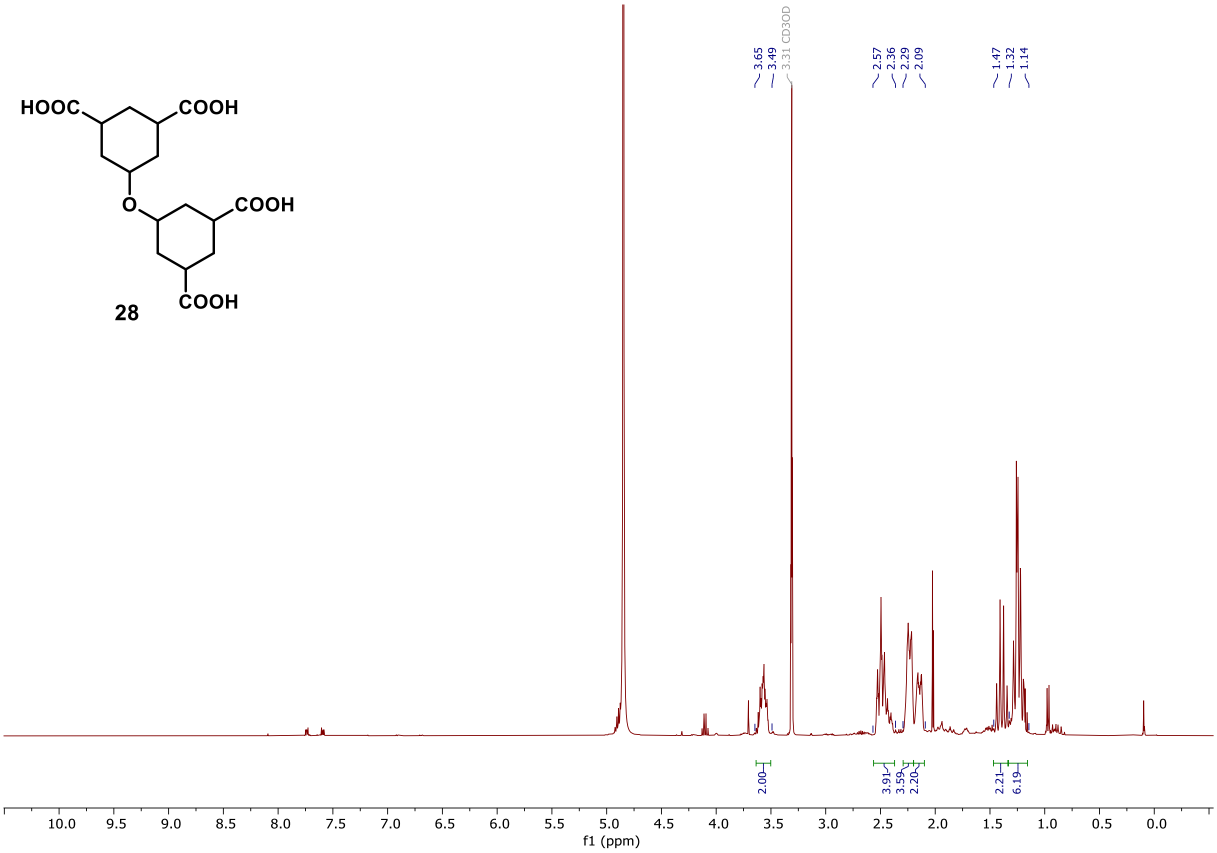

Figure S234: *^{1}H NMR spectra of tetracarboxylic acid* ***28*** *(400 MHz, MeOD).*

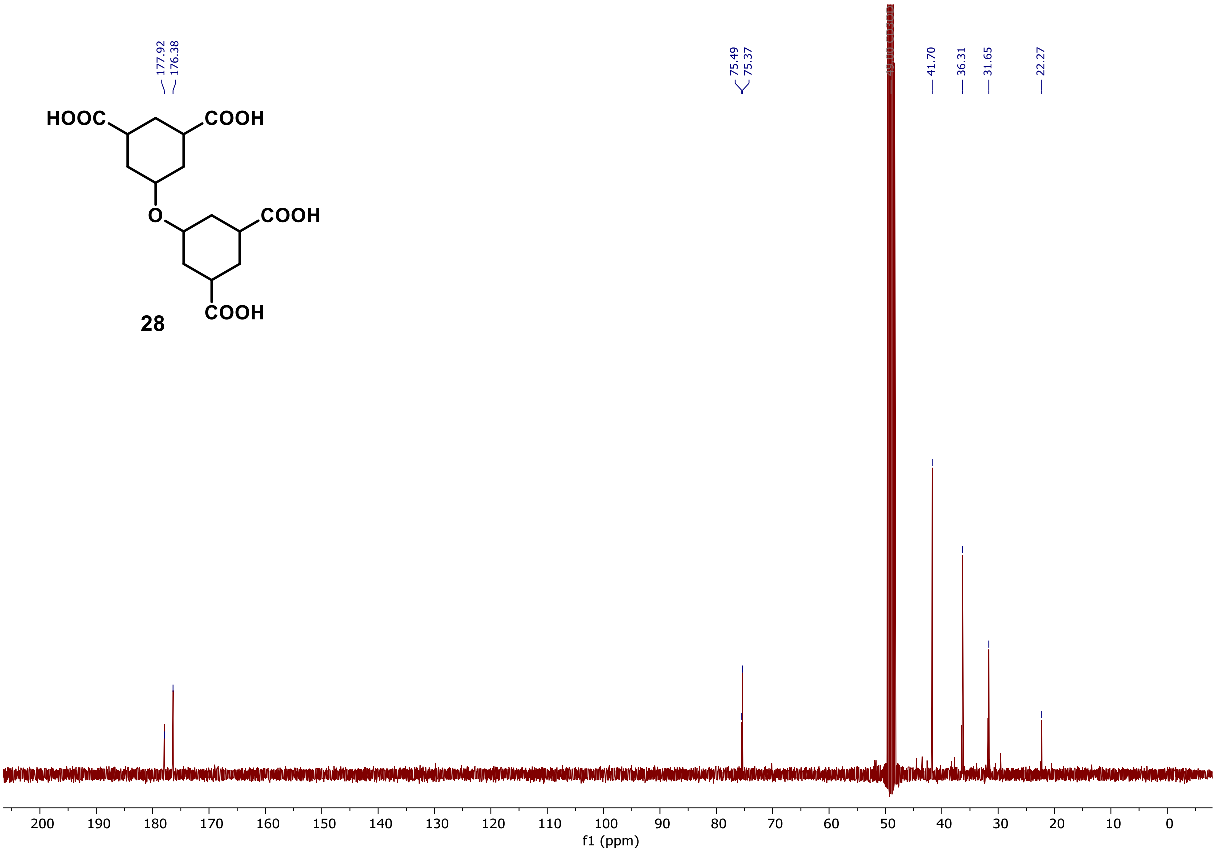

Figure S235: *^{13}C NMR spectrum of tetracarboxylic acid* ***28*** *(101 MHz, MeOD).*

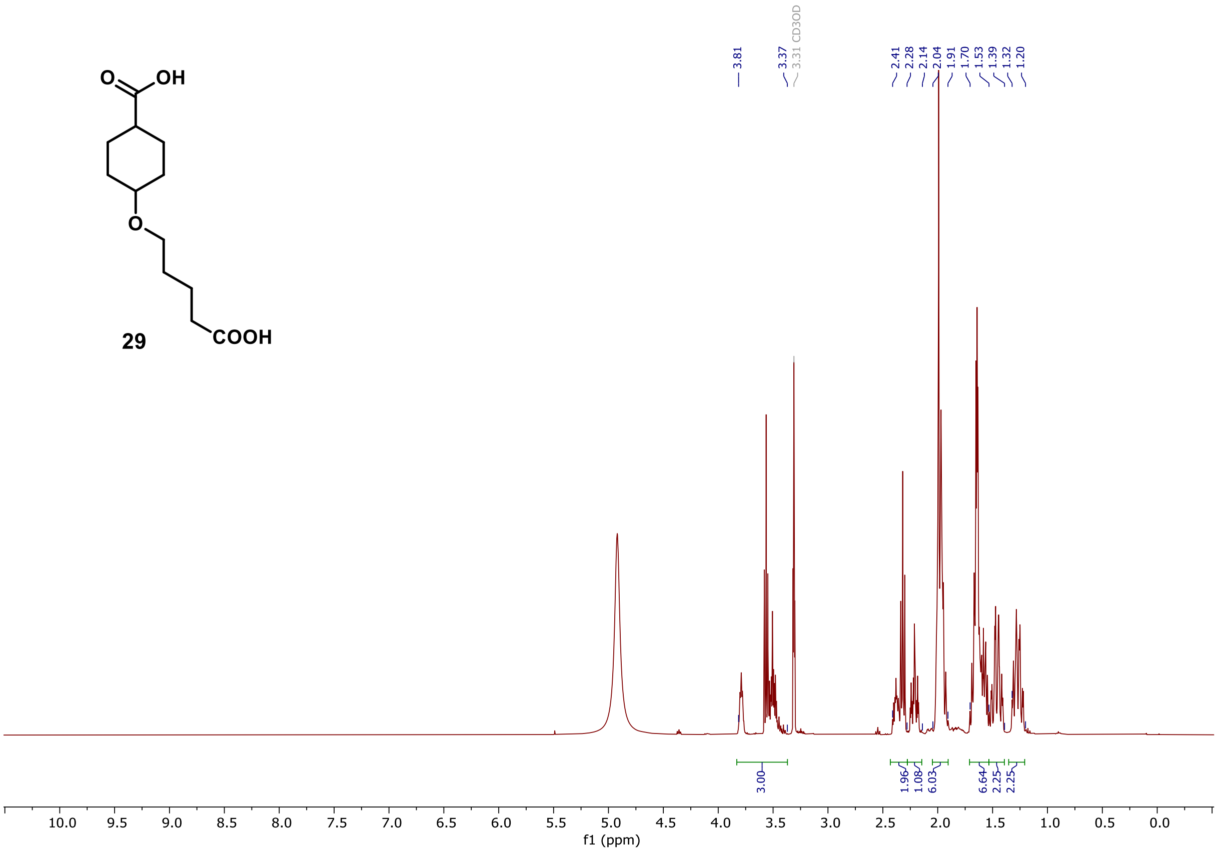

Figure S236: *^{1}H NMR spectra of dicarboxylic acid* ***29*** *(400 MHz, MeOD).*

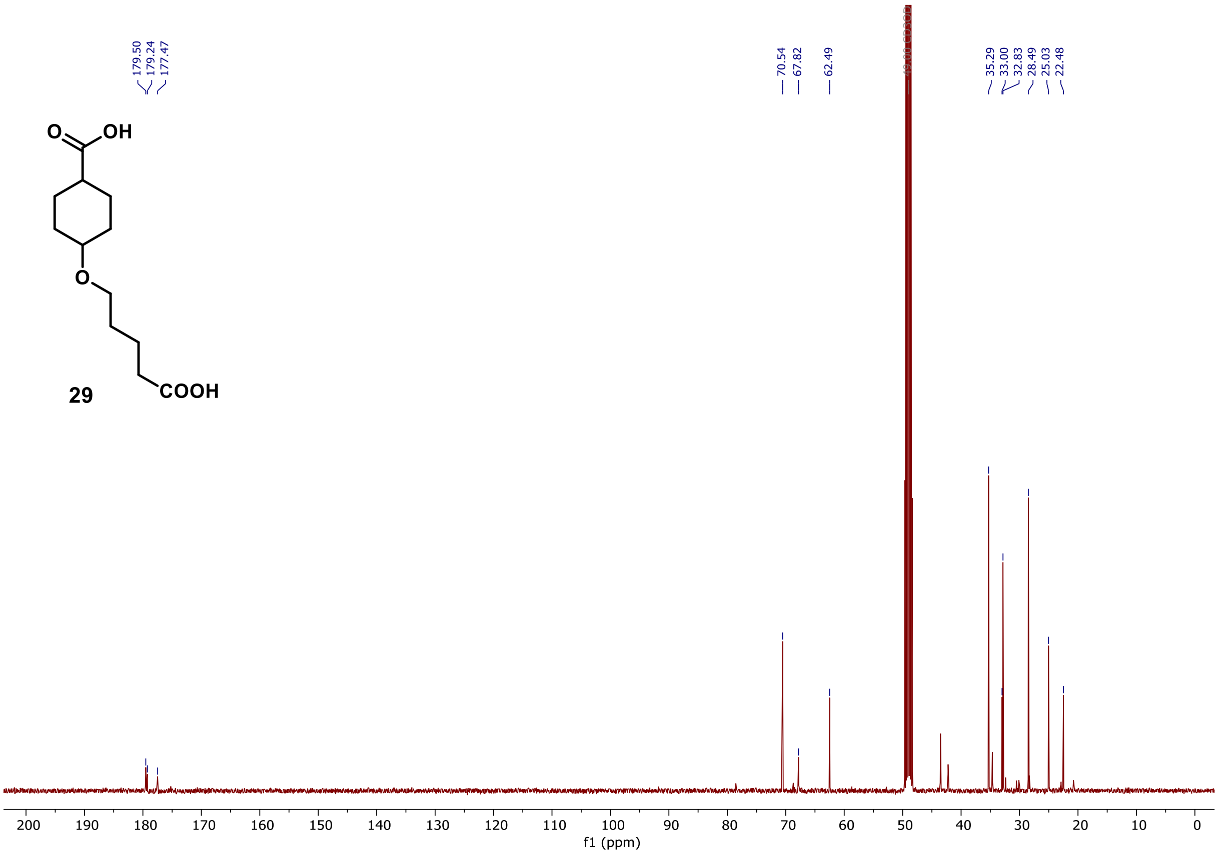

Figure S237: *^{13}C NMR spectrum of dicarboxylic acid* ***29*** *(101 MHz, MeOD).*

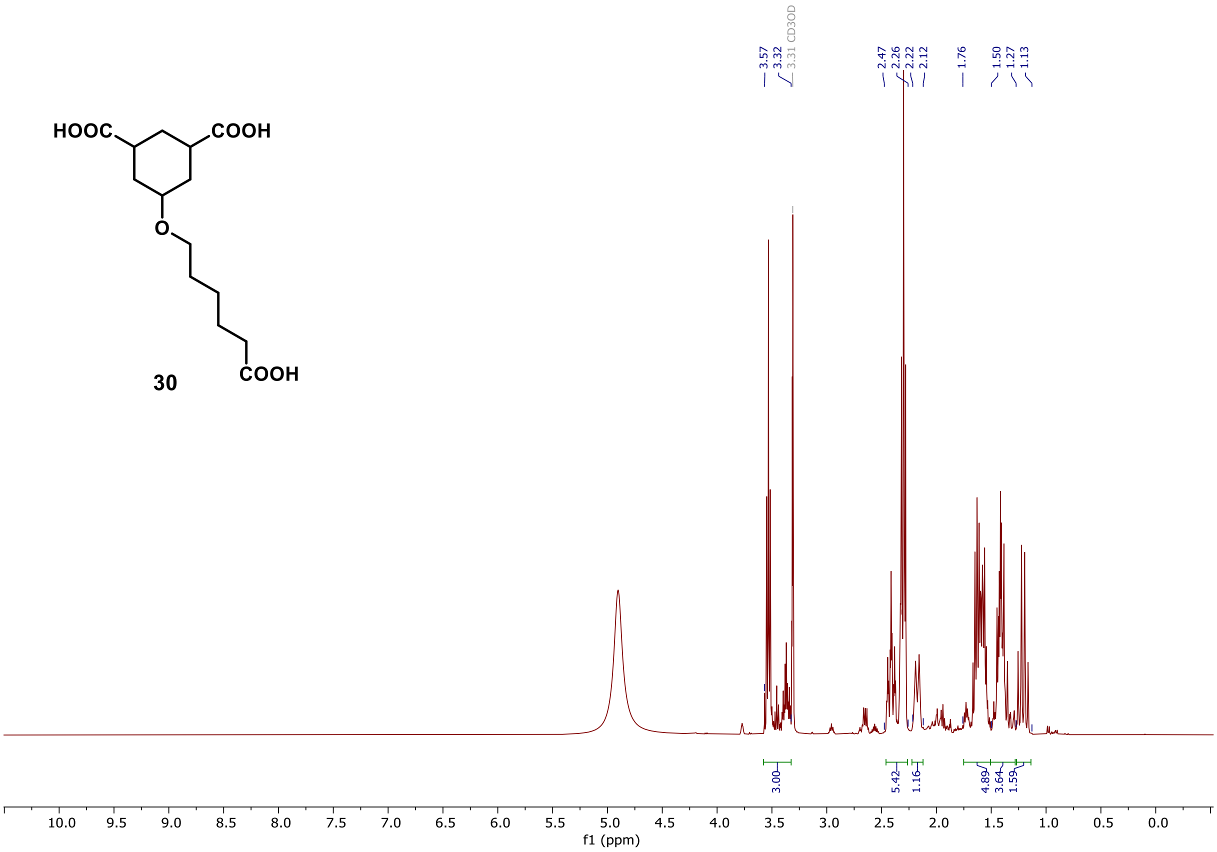

Figure S238: *^{1}H NMR spectra of tricarboxylic acid **30** (400 MHz, MeOD).*

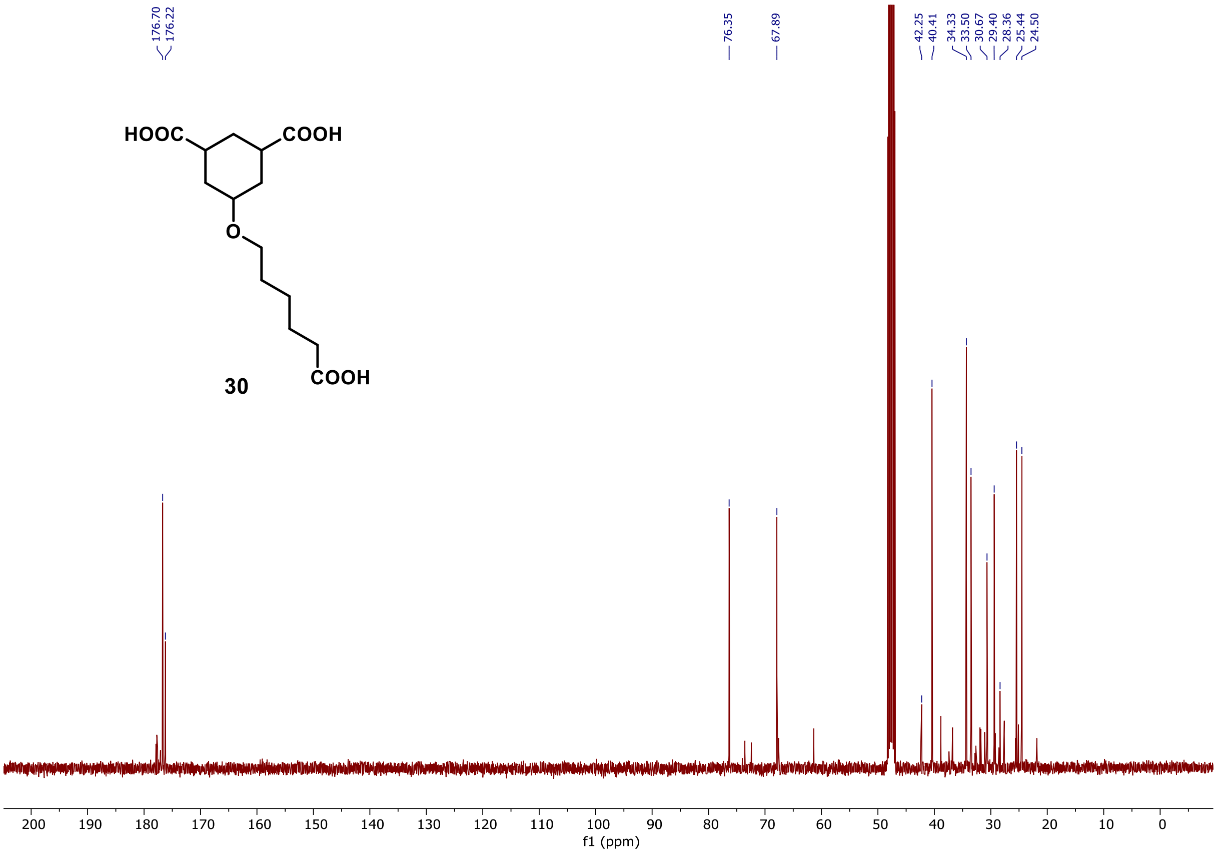

Figure S239: *^{13}C NMR spectrum of tricarboxylic acid **30** (101 MHz, MeOD).*

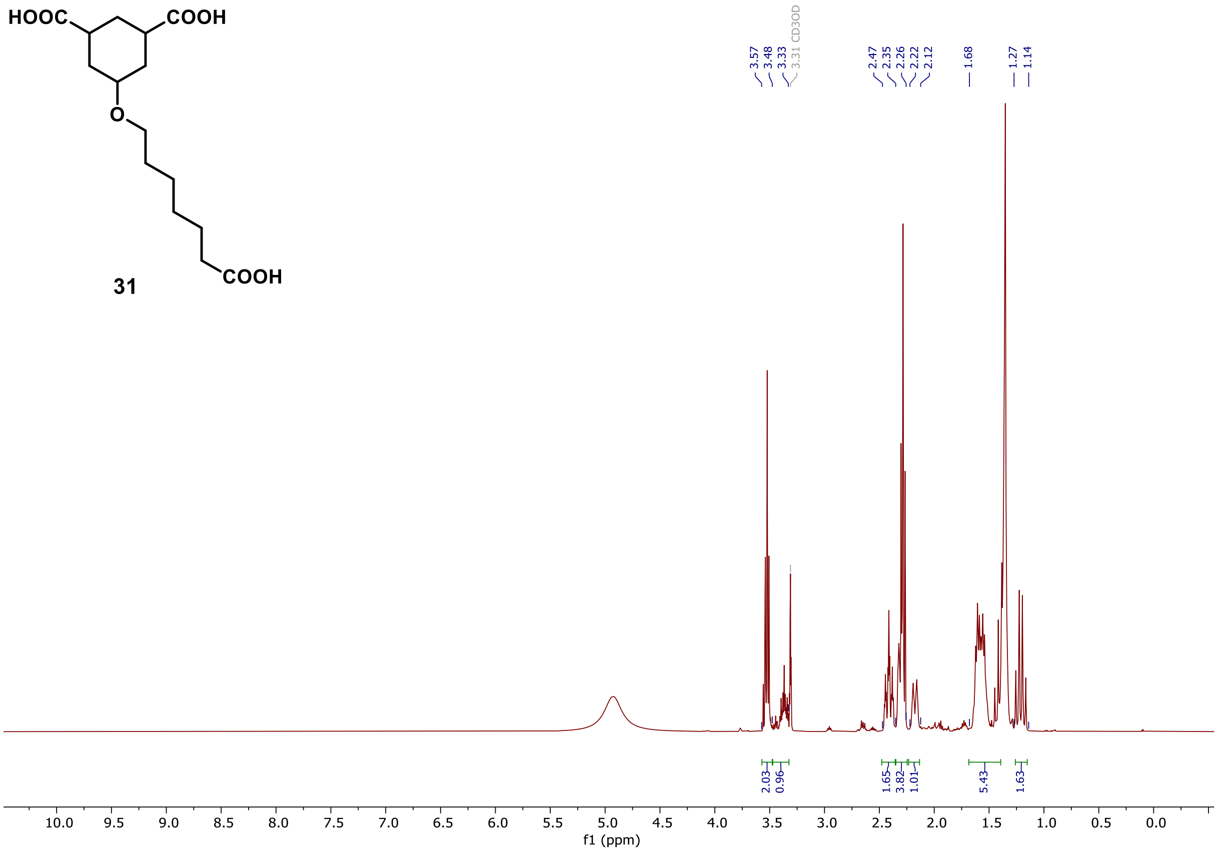

Figure S240: *^{1}H NMR spectra of tricarboxylic acid **31** (400 MHz, MeOD).*

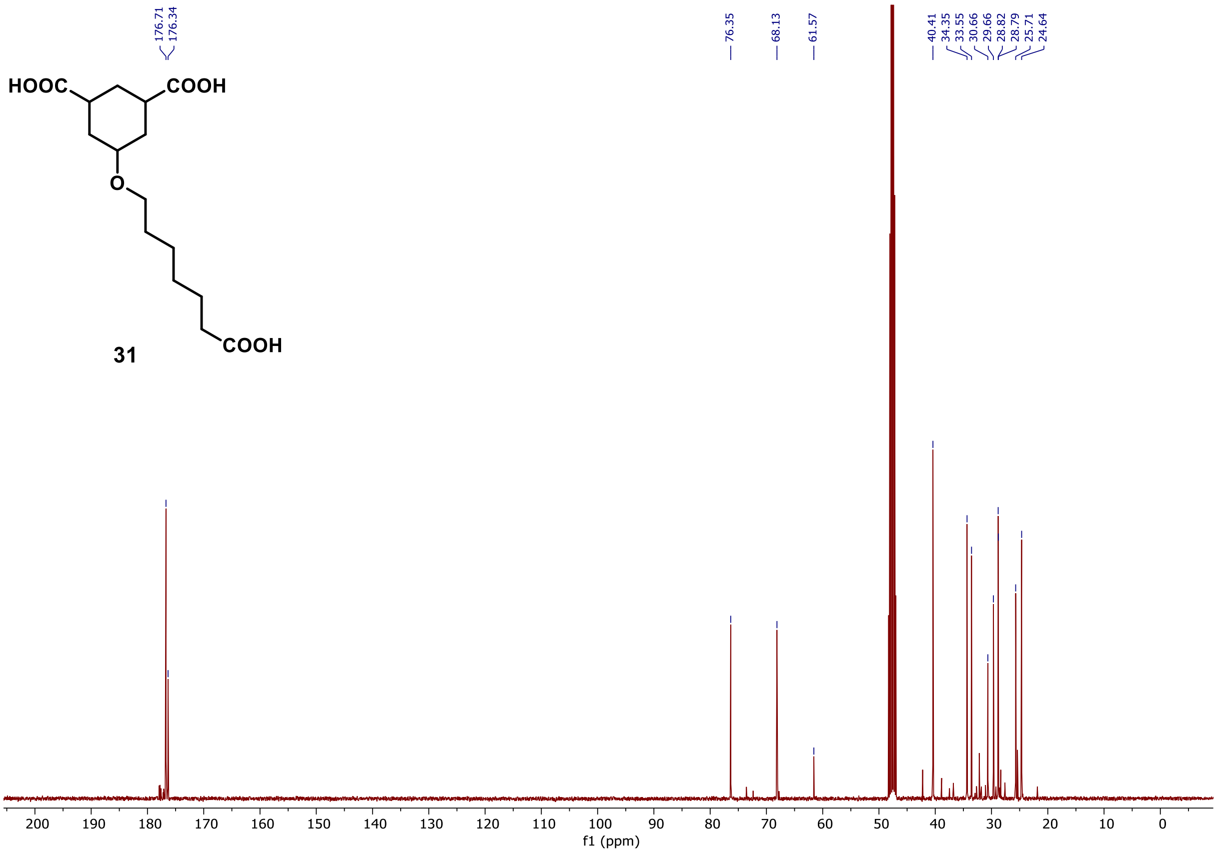

Figure S241: *^{13}C NMR spectrum of tricarboxylic acid **31** (101 MHz, MeOD).*

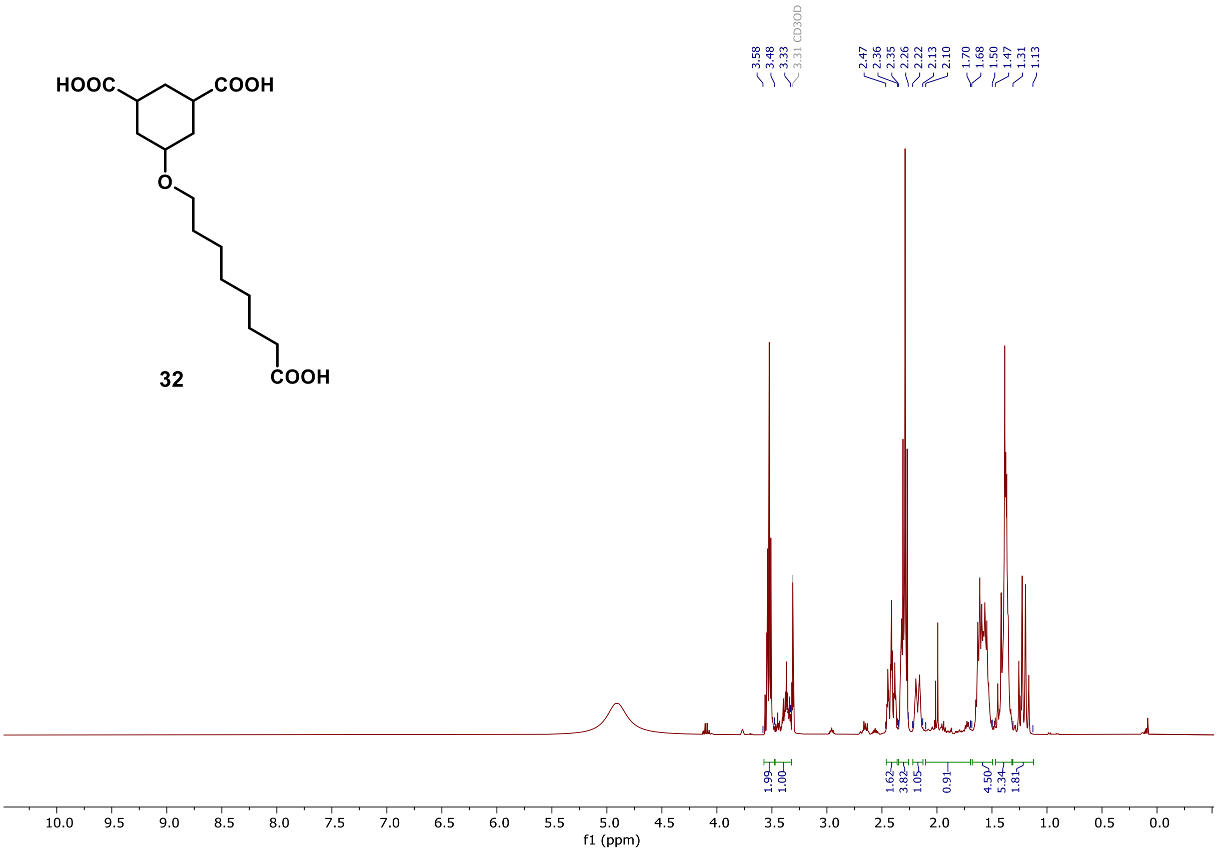

*Figure S242: ^{1}H NMR spectra of tricarboxylic acid **32** (400 MHz, MeOD).*

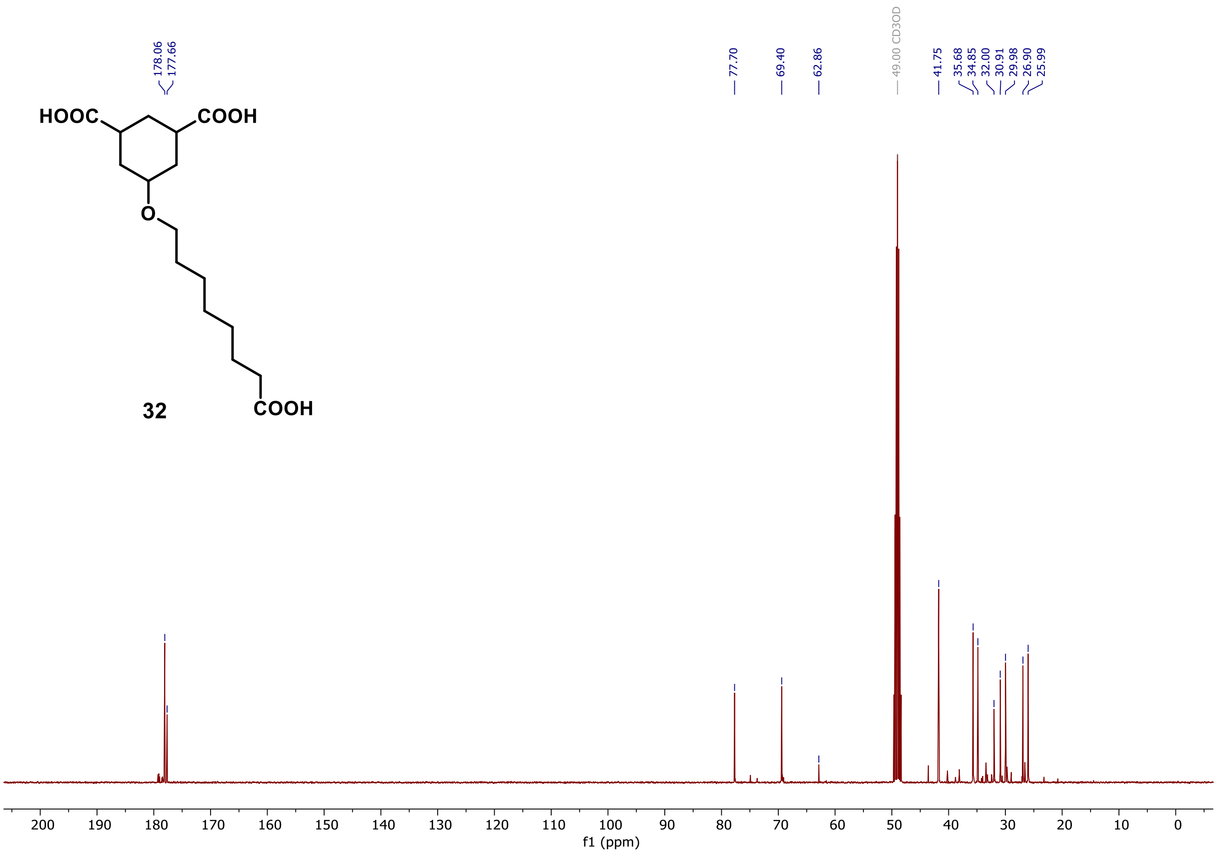

*Figure S243: ^{13}C NMR spectrum of tricarboxylic acid **32** (101 MHz, MeOD).*

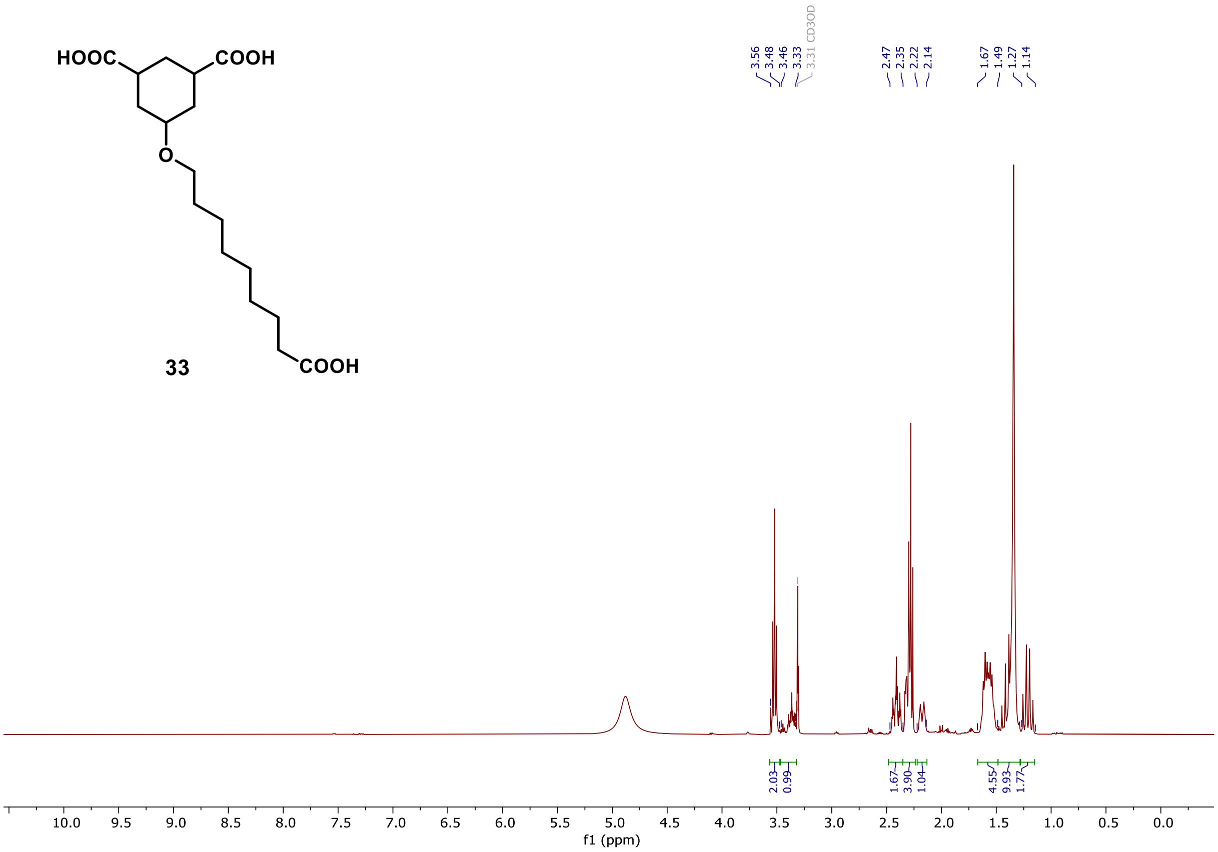

Figure S244: *^{1}H NMR spectra of tricarboxylic acid* ***33*** *(400 MHz, MeOD).*

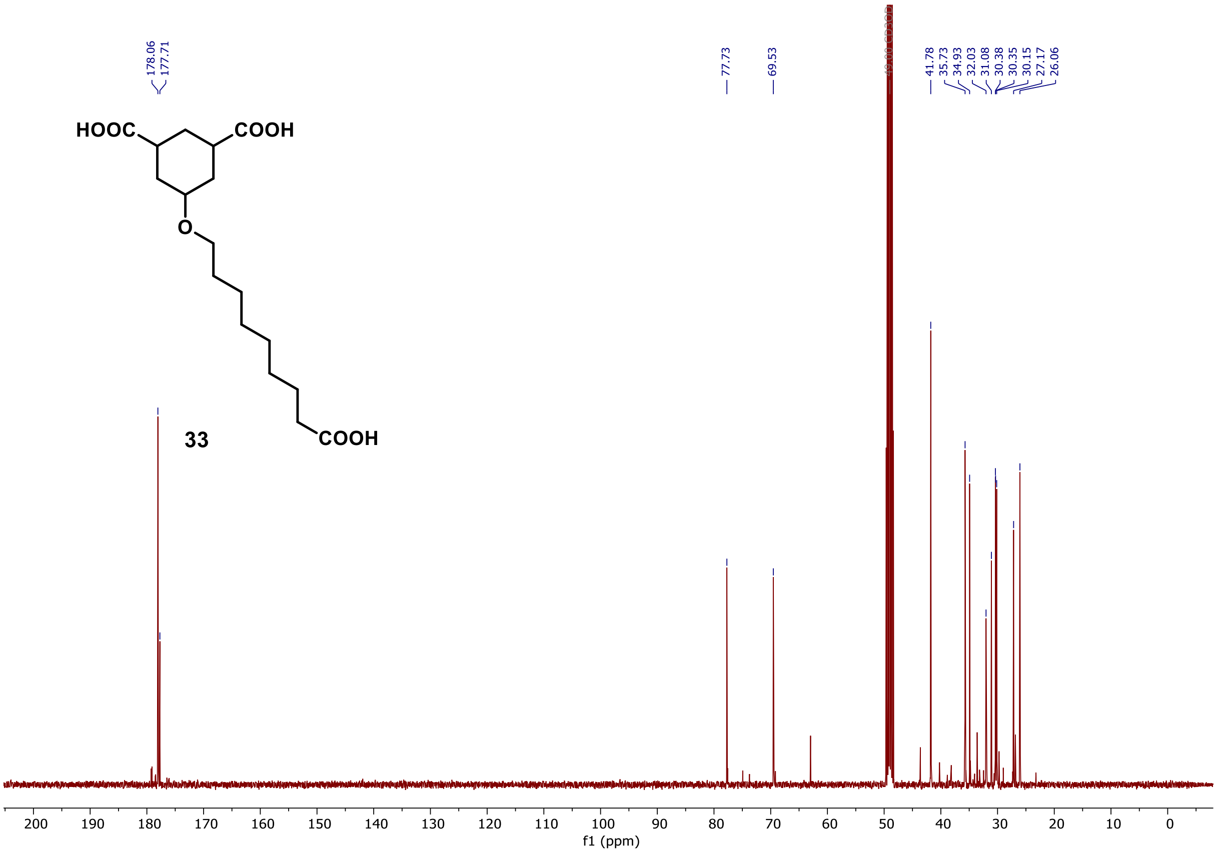

Figure S245: *^{13}C NMR spectrum of tricarboxylic acid* ***33*** *(101 MHz, MeOD).*

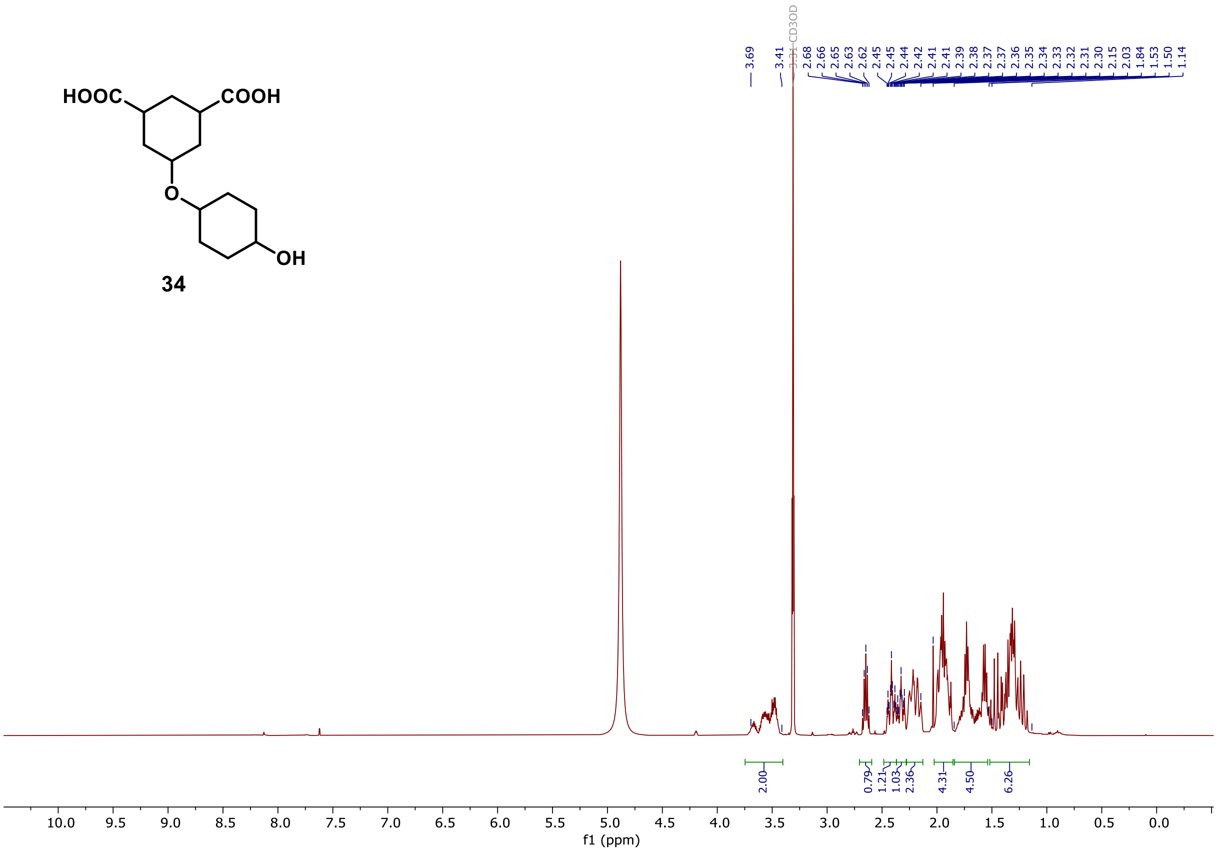

Figure S246: *^{1}H NMR spectra of alcohol* ***34*** *(400 MHz, MeOD).*

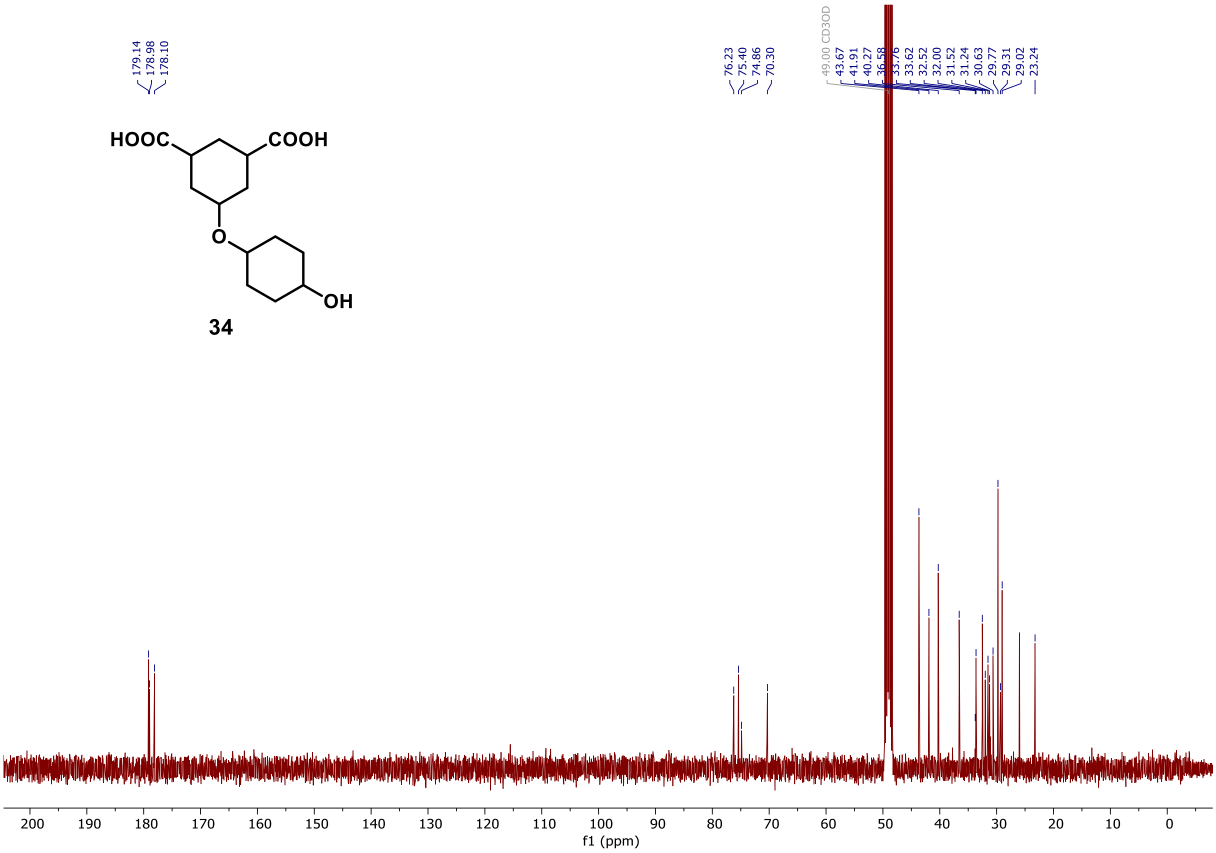

Figure S247: *^{13}C NMR spectrum of alcohol* ***34*** *(101 MHz, MeOD).*

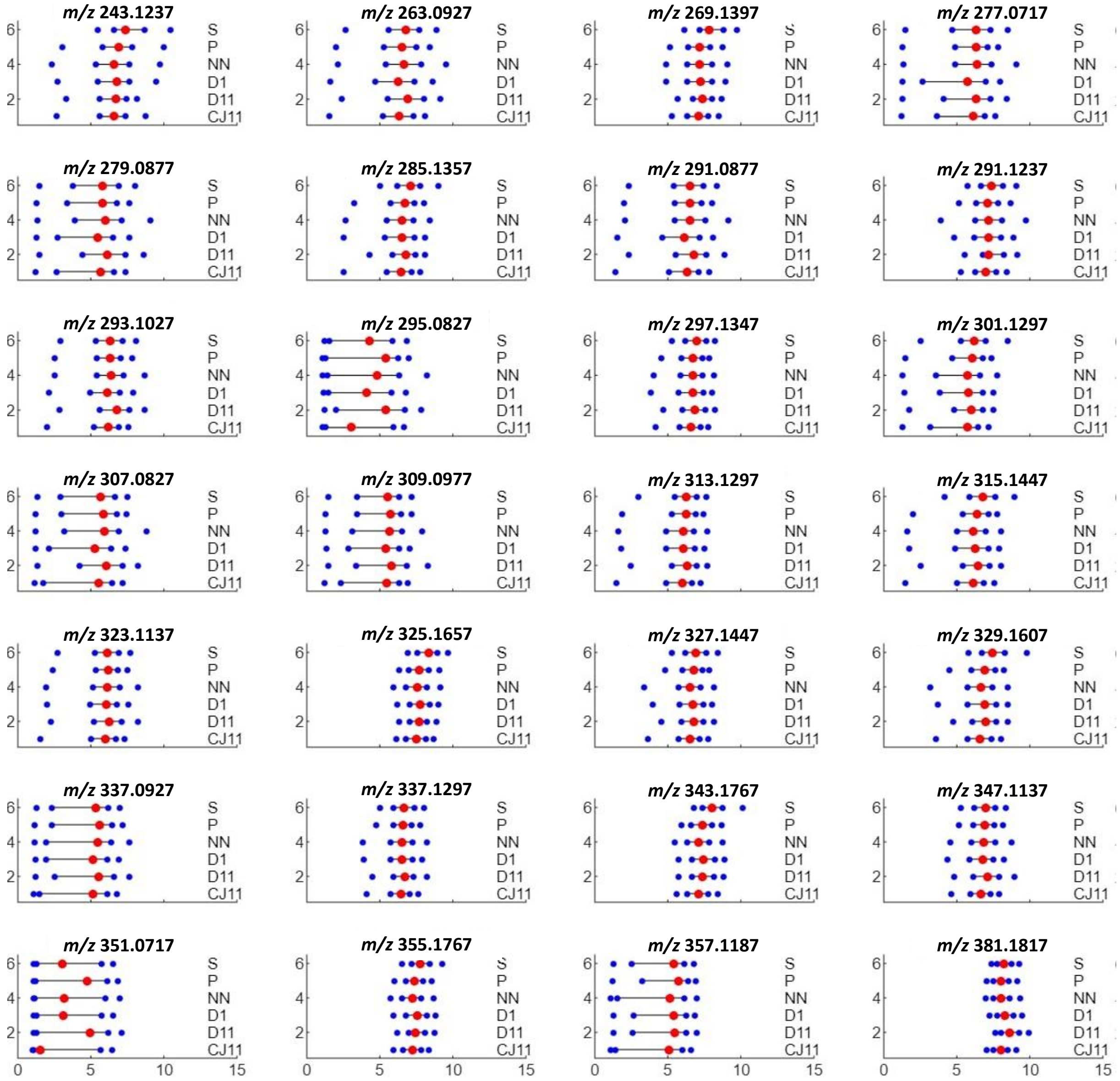

Figure S248: *Comparison plots for %CI values of DOM samples (S = SRFA, P = Plåten, NN = NRNOM, D1, D11, and CJ11) at different masses corresponding to synthetic compounds* ***1***-***38***. *Red dots indicate CI values of 50%, lines represent ranges from 25-50% and 50-75%, and outer blue dots represent CI values of 10% and 90%.*

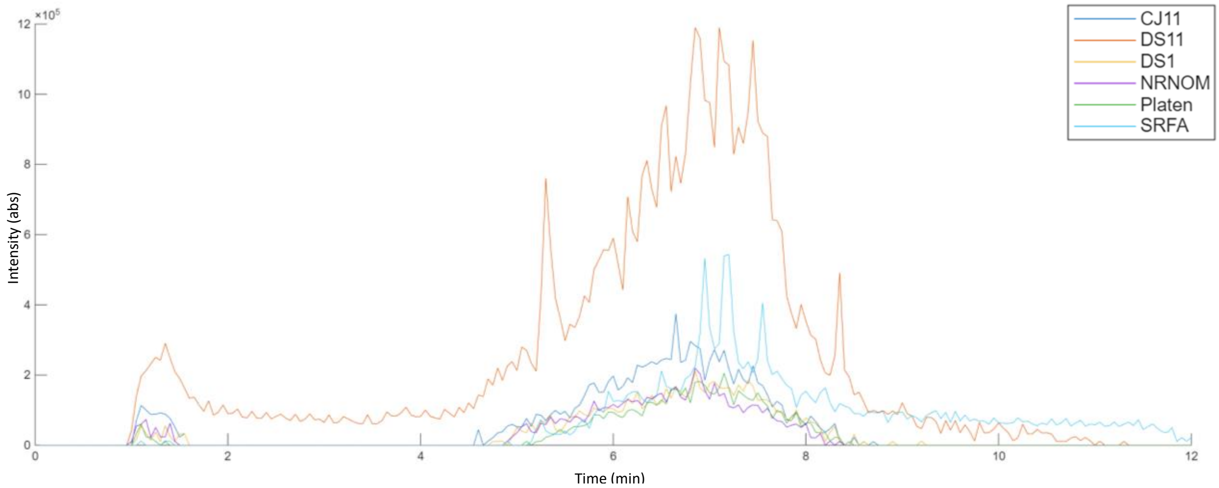

Figure S249: *XIC for 243.1237 m/z for CJ11 (dark blue), DS11 (orange), DS1 (yellow), NRNOM (purple), Plåten (green), and SRFA (light blue).*

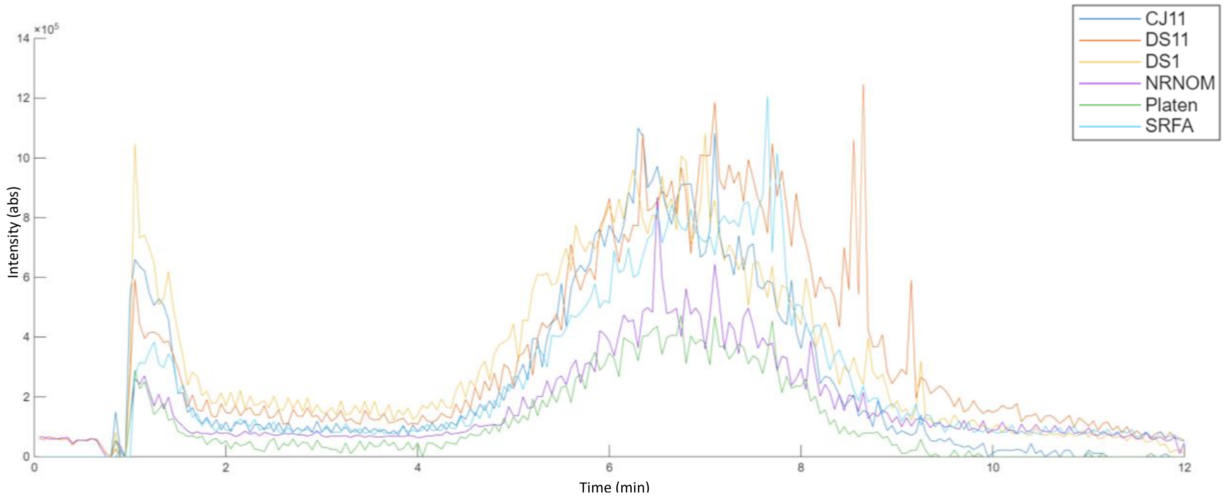

Figure S250: *XIC for 263.0927 m/z for CJ11 (dark blue), DS11 (orange), DS1 (yellow), NRNOM (purple), Plåten (green), and SRFA (light blue).*

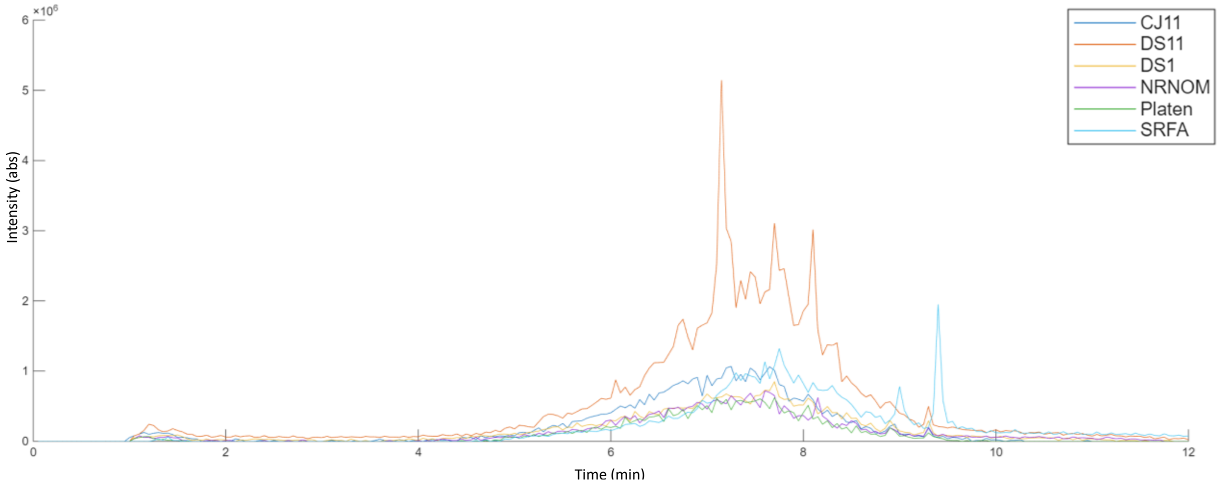

Figure S251: *XIC for 269.1397 m/z for CJ11 (dark blue), DS11 (orange), DS1 (yellow), NRNOM (purple), Plåten (green), and SRFA (light blue).*

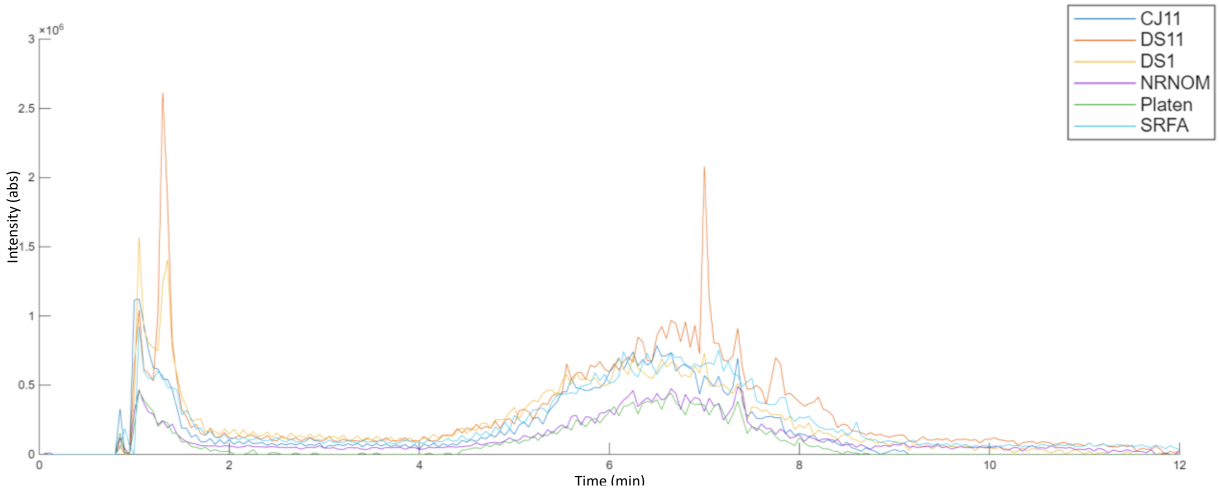

Figure S252: *XIC for 277.0717 m/z for CJ11 (dark blue), DS11 (orange), DS1 (yellow), NRNOM (purple), Plåten (green), and SRFA (light blue).*

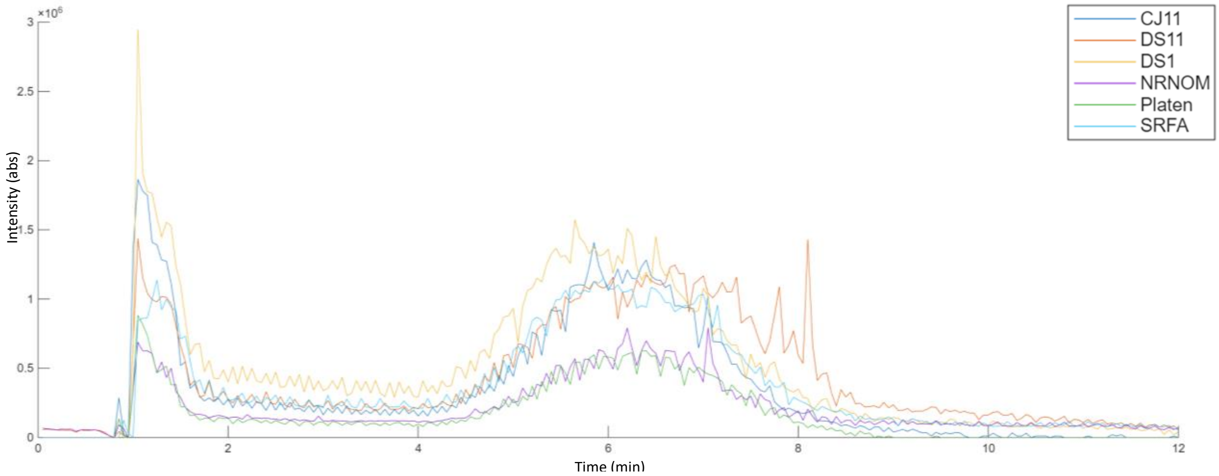

Figure S253: *XIC for 279.0877 m/z for CJ11 (dark blue), DS11 (orange), DS1 (yellow), NRNOM (purple), Plåten (green), and SRFA (light blue).*

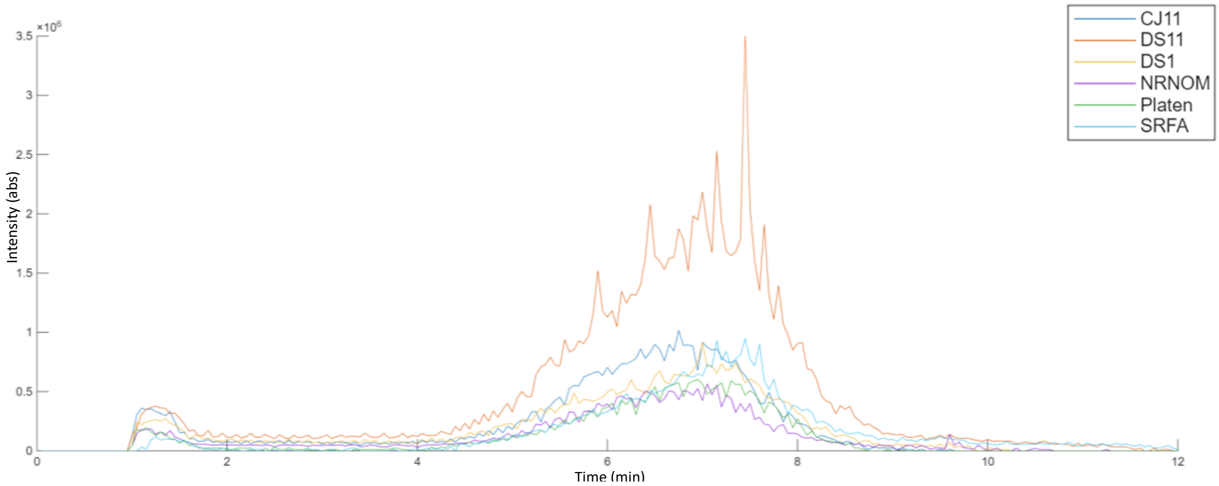

Figure S254: *XIC for 285.1357 m/z for CJ11 (dark blue), DS11 (orange), DS1 (yellow), NRNOM (purple), Plåten (green), and SRFA (light blue).*

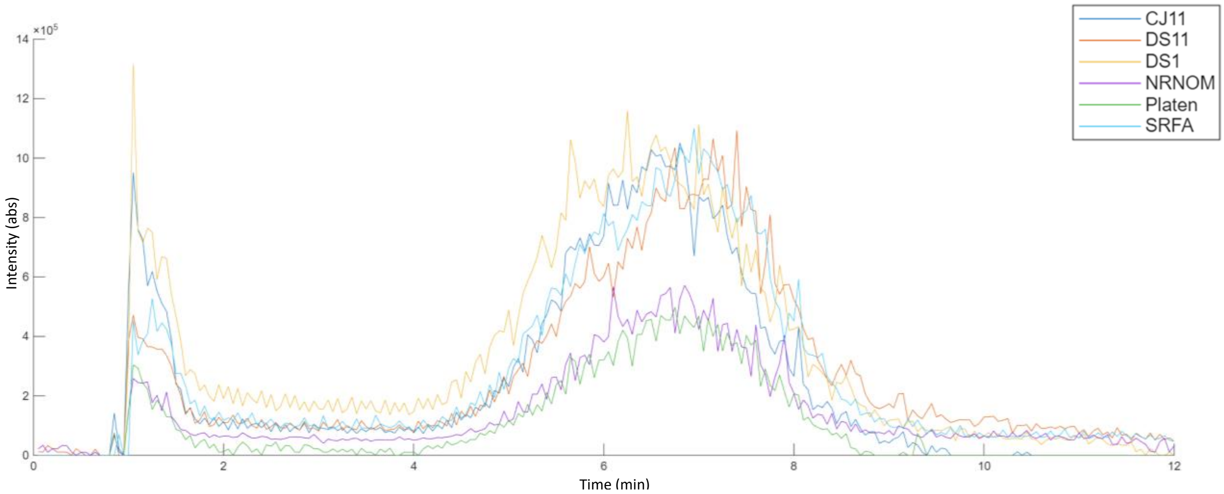

Figure S255: *XIC for 291.0877 m/z for CJ11 (dark blue), DS11 (orange), DS1 (yellow), NRNOM (purple), Plåten (green), and SRFA (light blue).*

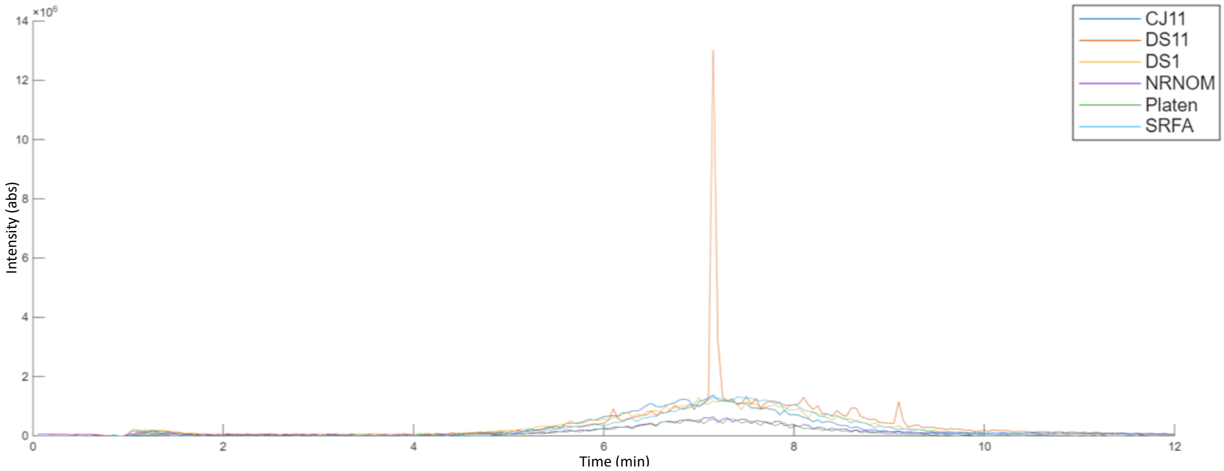

Figure S256: *XIC for 291.1237 m/z for CJ11 (dark blue), DS11 (orange), DS1 (yellow), NRNOM (purple), Plåten (green), and SRFA (light blue).*

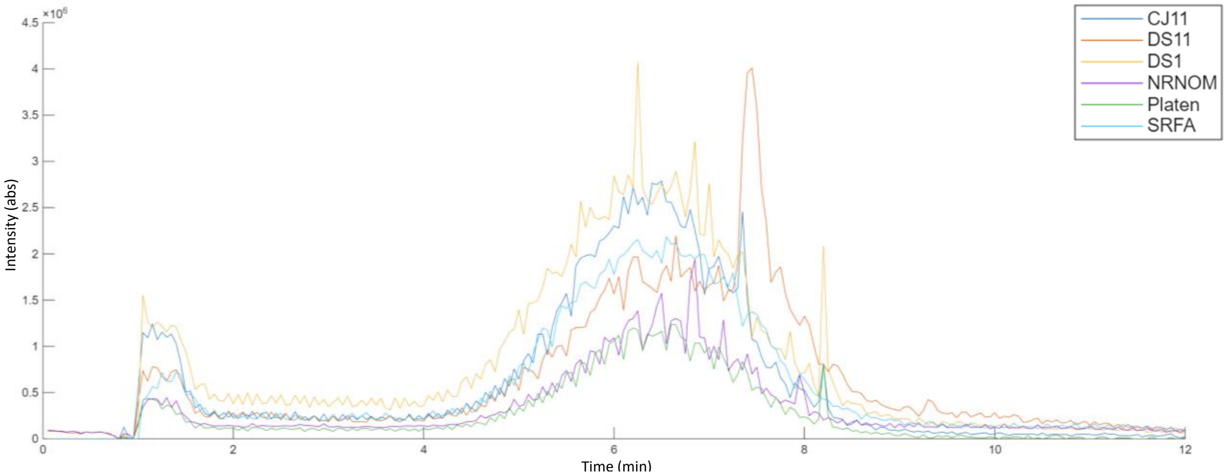

Figure S257: *XIC for 293.1027 m/z for CJ11 (dark blue), DS11 (orange), DS1 (yellow), NRNOM (purple), Plåten (green), and SRFA (light blue).*

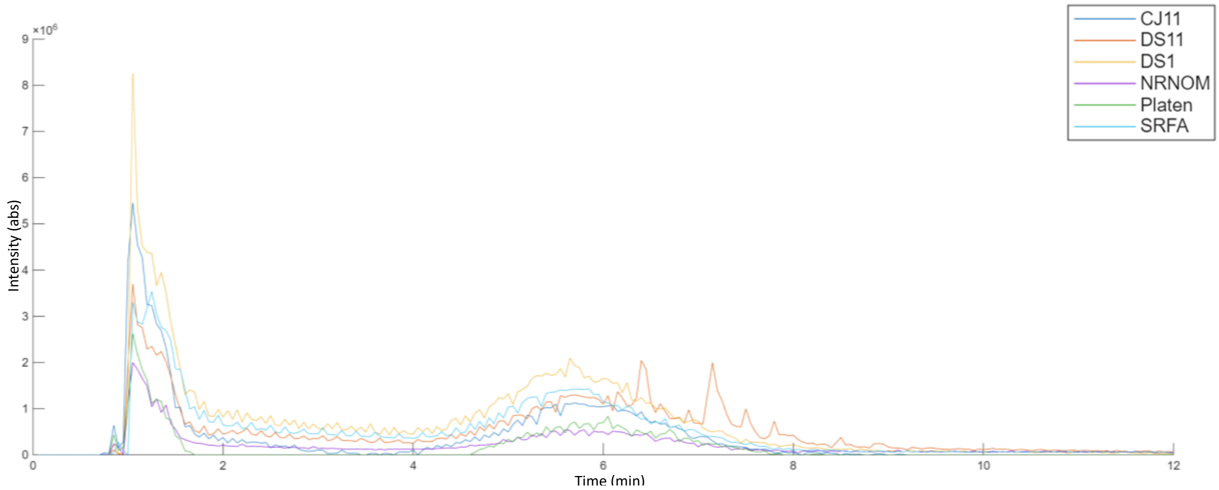

Figure S258: *XIC for 295.0827 m/z for CJ11 (dark blue), DS11 (orange), DS1 (yellow), NRNOM (purple), Plåten (green), and SRFA (light blue).*

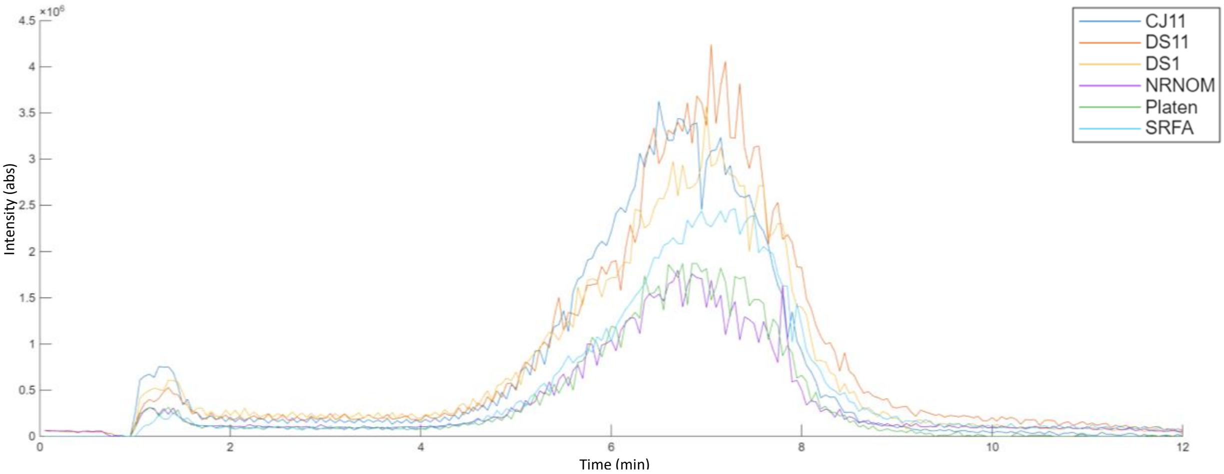

Figure S259: *XIC for 297.1347 m/z for CJ11 (dark blue), DS11 (orange), DS1 (yellow), NRNOM (purple), Plåten (green), and SRFA (light blue).*

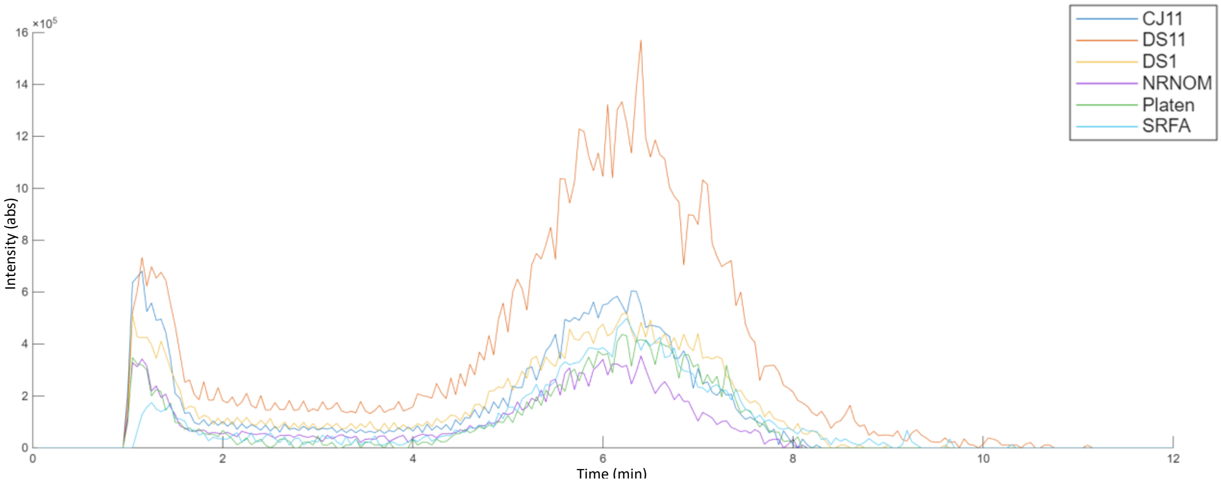

Figure S260: *XIC for 301.1297 m/z for CJ11 (dark blue), DS11 (orange), DS1 (yellow), NRNOM (purple), Plåten (green), and SRFA (light blue).*

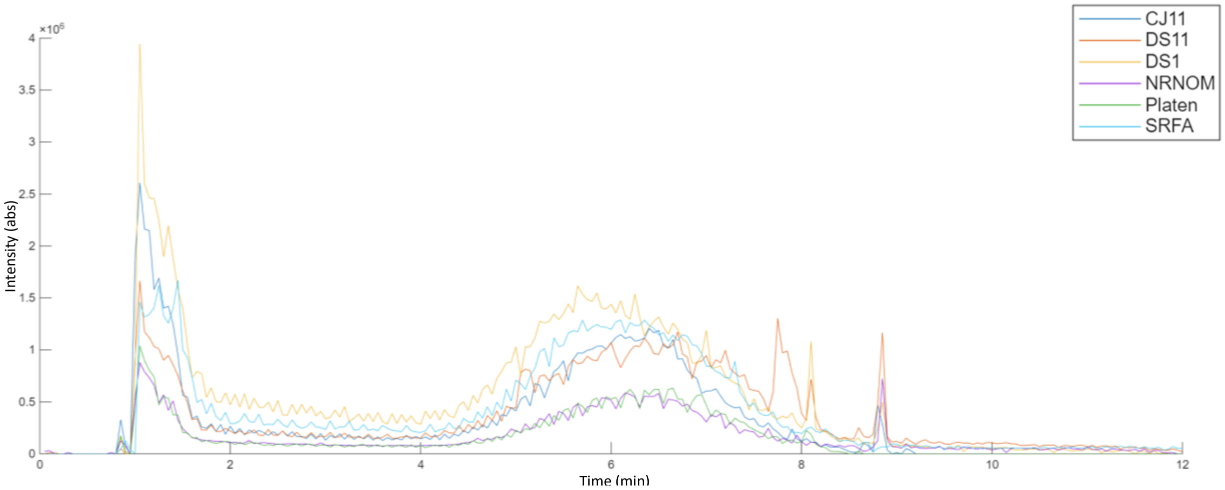

Figure S261: *XIC for 307.0827 m/z for CJ11 (dark blue), DS11 (orange), DS1 (yellow), NRNOM (purple), Plåten (green), and SRFA (light blue).*

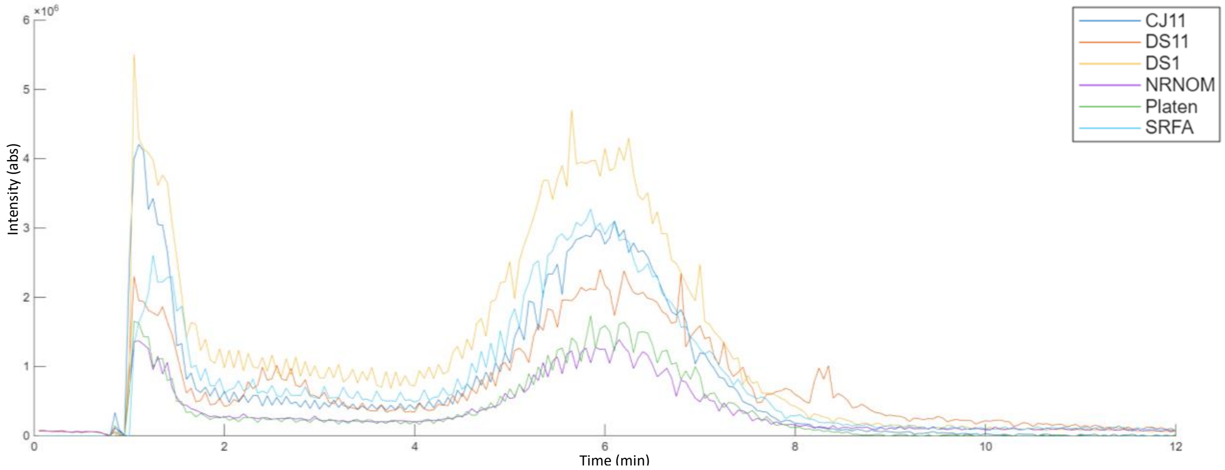

Figure S262: *XIC for 309.0977 m/z for CJ11 (dark blue), DS11 (orange), DS1 (yellow), NRNOM (purple), Plåten (green), and SRFA (light blue).*

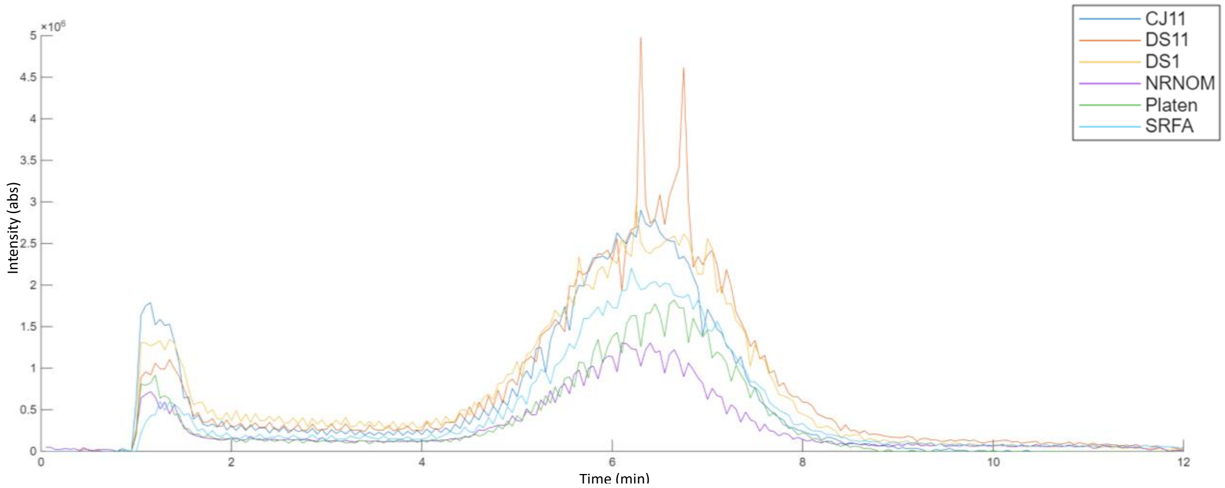

Figure S263: *XIC for 313.1297 m/z for CJ11 (dark blue), DS11 (orange), DS1 (yellow), NRNOM (purple), Plåten (green), and SRFA (light blue).*

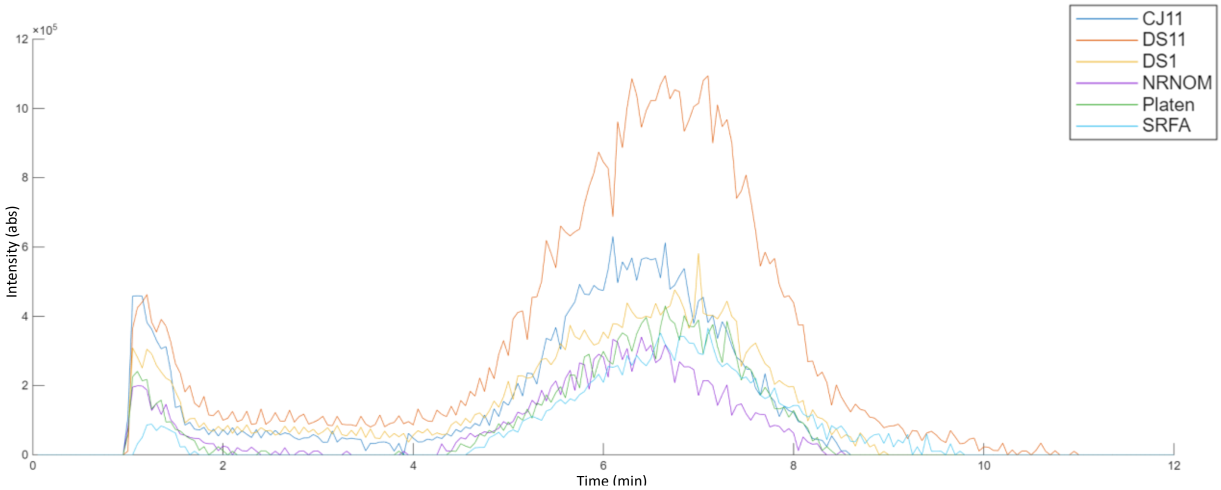

Figure S264: *XIC for 315.1447 m/z for CJ11 (dark blue), DS11 (orange), DS1 (yellow), NRNOM (purple), Plåten (green), and SRFA (light blue).*

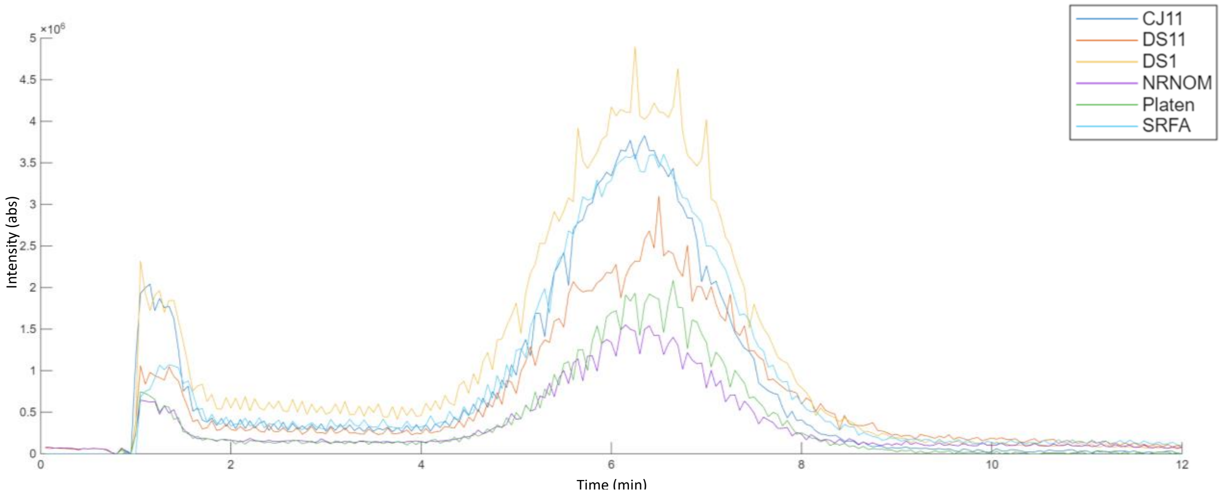

Figure S265: *XIC for 323.1137 m/z for CJ11 (dark blue), DS11 (orange), DS1 (yellow), NRNOM (purple), Plåten (green), and SRFA (light blue).*

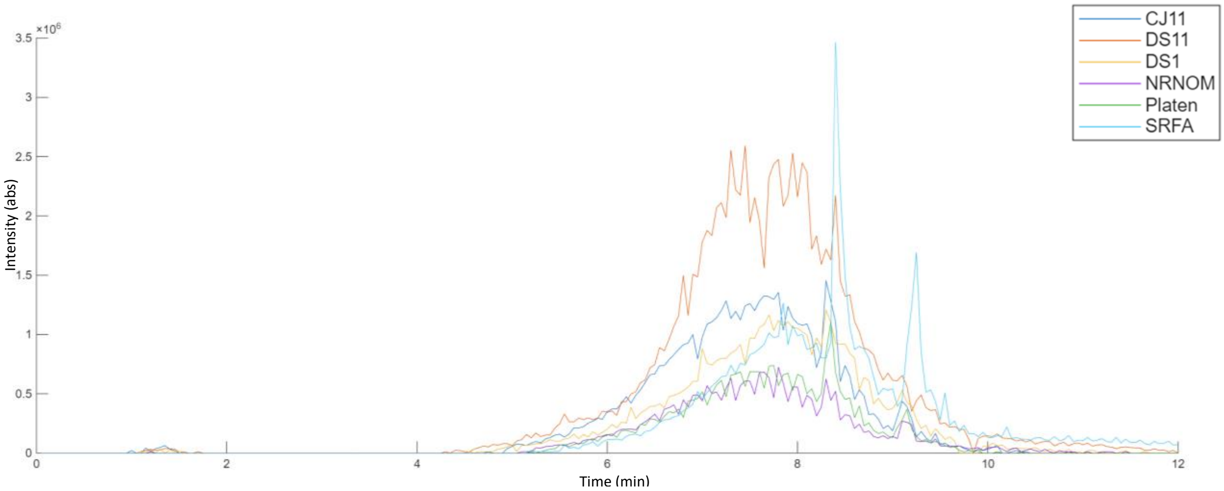

Figure S266: *XIC for 325.1657 m/z for CJ11 (dark blue), DS11 (orange), DS1 (yellow), NRNOM (purple), Plåten (green), and SRFA (light blue).*

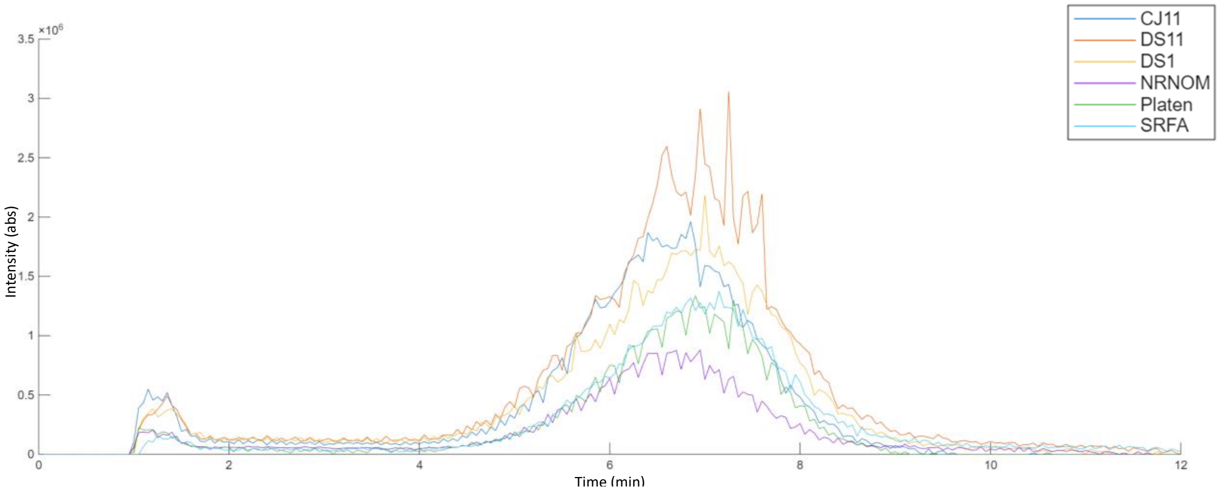

Figure S267: *XIC for 327.1447 m/z for CJ11 (dark blue), DS11 (orange), DS1 (yellow), NRNOM (purple), Plåten (green), and SRFA (light blue).*

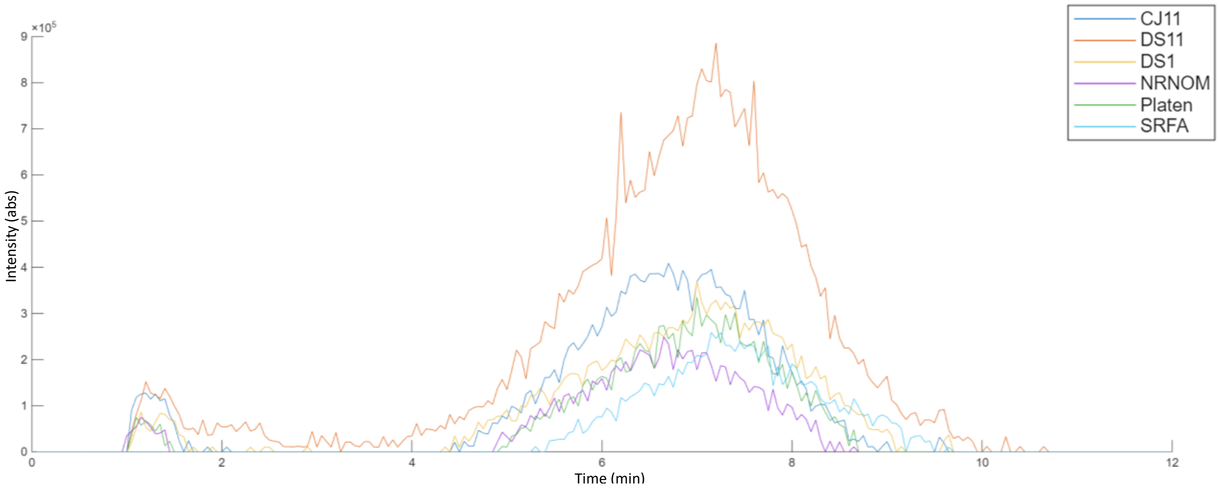

Figure S268: *XIC for 329.1607 m/z for CJ11 (dark blue), DS11 (orange), DS1 (yellow), NRNOM (purple), Plåten (green), and SRFA (light blue).*

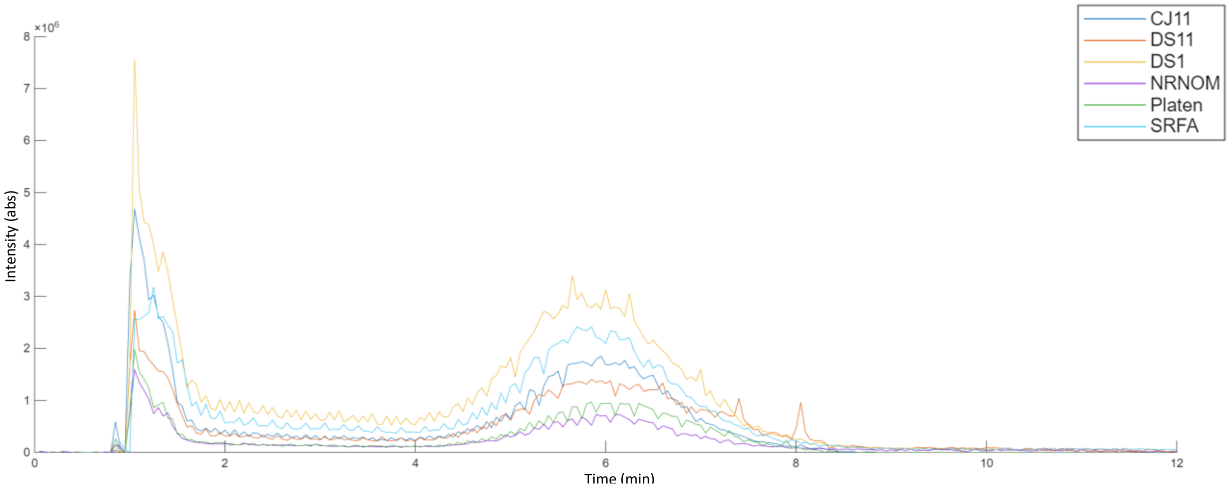

Figure S269: *XIC for 337.0927 m/z for CJ11 (dark blue), DS11 (orange), DS1 (yellow), NRNOM (purple), Plåten (green), and SRFA (light blue).*

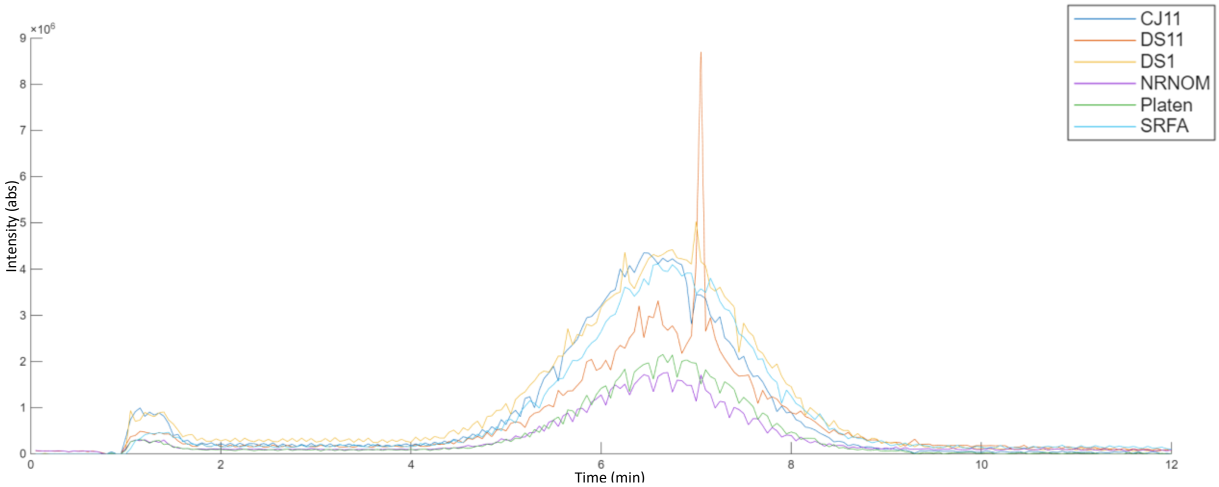

Figure S270: *XIC for 337.1297 m/z for CJ11 (dark blue), DS11 (orange), DS1 (yellow), NRNOM (purple), Plåten (green), and SRFA (light blue).*

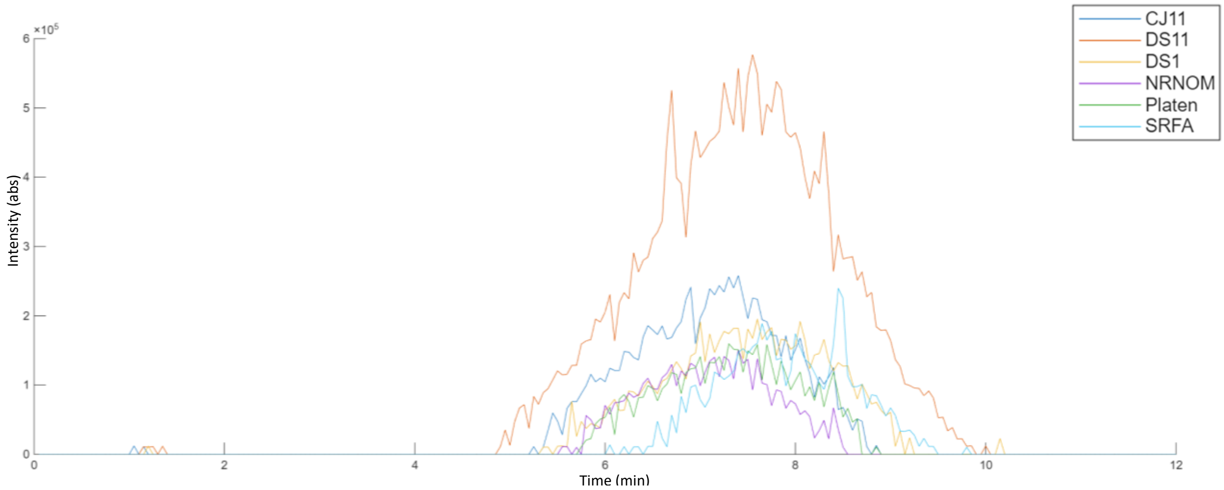

Figure S271: *XIC for 343.1767 m/z for CJ11 (dark blue), DS11 (orange), DS1 (yellow), NRNOM (purple), Plåten (green), and SRFA (light blue).*

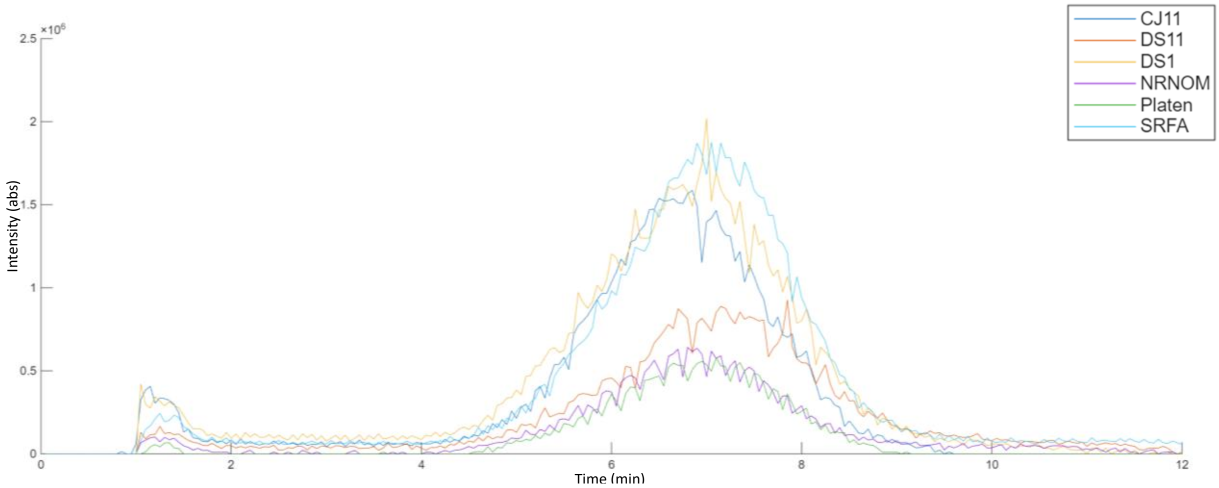

Figure S272: *XIC for 347.1137 m/z for CJ11 (dark blue), DS11 (orange), DS1 (yellow), NRNOM (purple), Plåten (green), and SRFA (light blue).*

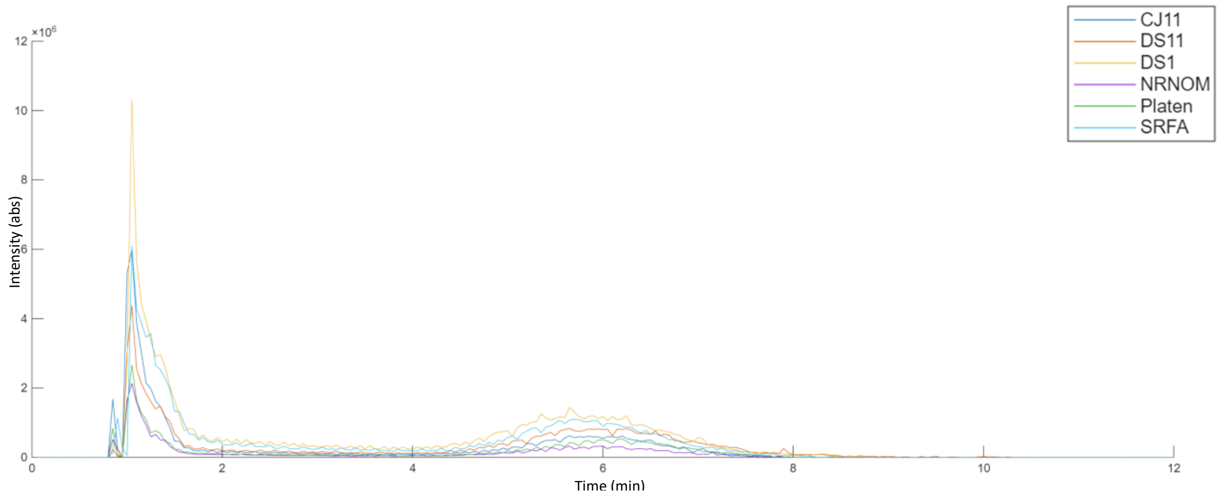

Figure S273: *XIC for 351.0717 m/z for CJ11 (dark blue), DS11 (orange), DS1 (yellow), NRNOM (purple), Plåten (green), and SRFA (light blue).*

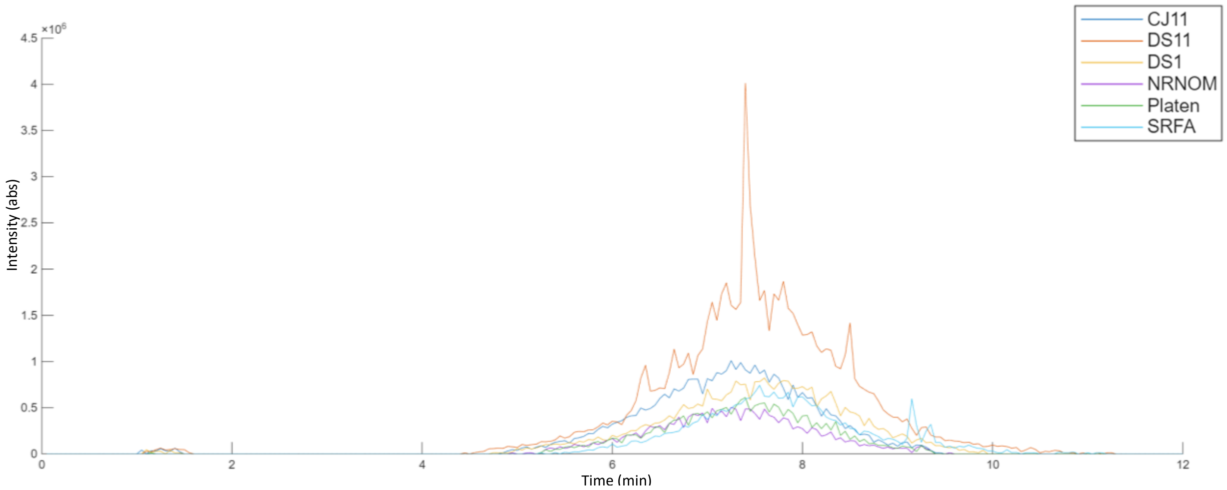

Figure S274: *XIC for 355.1767 m/z for CJ11 (dark blue), DS11 (orange), DS1 (yellow), NRNOM (purple), Plåten (green), and SRFA (light blue).*

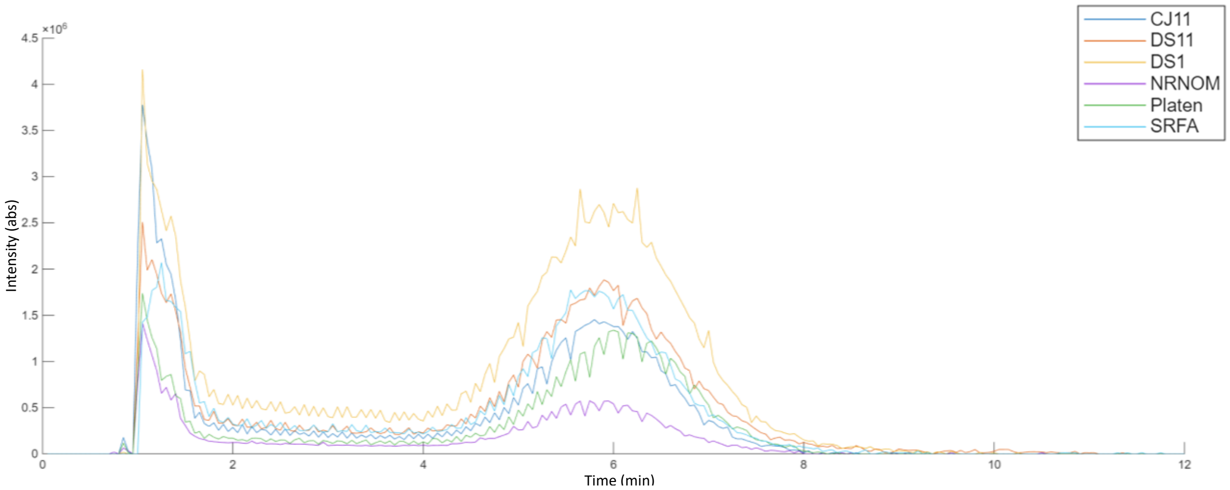

Figure S275: *XIC for 357.1187 m/z for CJ11 (dark blue), DS11 (orange), DS1 (yellow), NRNOM (purple), Plåten (green), and SRFA (light blue).*

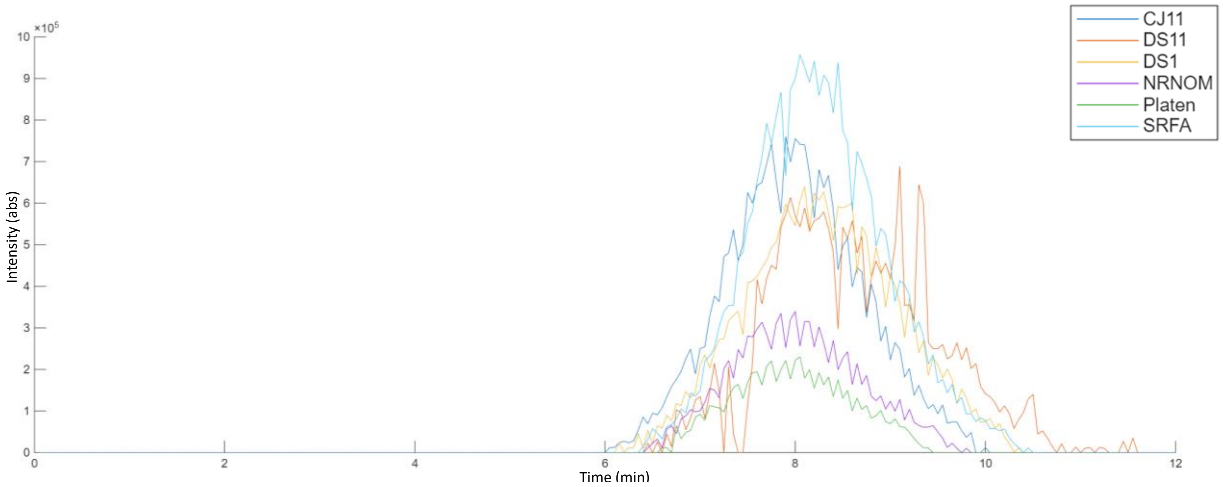

Figure S276: *XIC for 381.1817 m/z for CJ11 (dark blue), DS11 (orange), DS1 (yellow), NRNOM (purple), Plåten (green), and SRFA (light blue).*

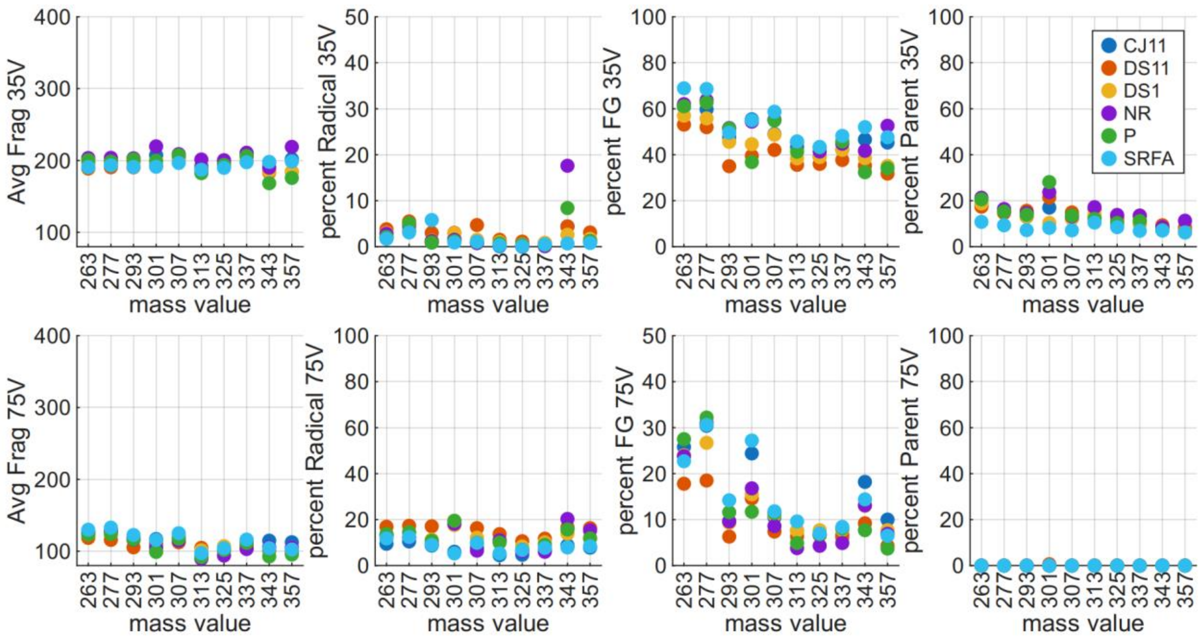

Figure S277*: 35V Avg Frag (**A**), %Rad (**B**), %FG (**C**), and %Parent (**D**), and 75V Avg Frag (**E**), %Rad (**F**), %FG (**G**), and %Parent (**H**) for DOM samples CJ11 (dark blue), DS11 (orange), DS1 (yellow), NRNOM (purple), Plåten (green), and SRFA (light blue), using metrics calculated with no negative mass defect cutoff.*
